# Scaffold hopping from (5-hydroxymethyl) isophthalates to multisubstituted pyrimidines diminishes binding affinity to the C1 domain of protein kinase C

**DOI:** 10.1371/journal.pone.0195668

**Published:** 2018-04-11

**Authors:** Riccardo Provenzani, Ilari Tarvainen, Giulia Brandoli, Antti Lempinen, Sanna Artes, Ainoleena Turku, Maria Helena Jäntti, Virpi Talman, Jari Yli-Kauhaluoma, Raimo K. Tuominen, Gustav Boije af Gennäs

**Affiliations:** 1 Drug Research Program, Division of Pharmaceutical Chemistry and Technology, Faculty of Pharmacy, University of Helsinki, Helsinki, Finland; 2 Drug Research Program, Division of Pharmacology and Pharmacotherapy, Faculty of Pharmacy, University of Helsinki, Helsinki, Finland; University of East Anglia, UNITED KINGDOM

## Abstract

Protein kinase C (PKC) isoforms play a pivotal role in the regulation of numerous cellular functions, making them extensively studied and highly attractive drug targets. Utilizing the crystal structure of the PKC*δ* C1B domain, we have developed hydrophobic isophthalic acid derivatives that modify PKC functions by binding to the C1 domain of the enzyme. In the present study, we aimed to improve the drug-like properties of the isophthalic acid derivatives by increasing their solubility and enhancing the binding affinity. Here we describe the design and synthesis of a series of multisubstituted pyrimidines as analogs of C1 domain–targeted isophthalates and characterize their binding affinities to the PKC*α* isoform. In contrast to our computational predictions, the scaffold hopping from phenyl to pyrimidine core diminished the binding affinity. Although the novel pyrimidines did not establish improved binding affinity for PKC*α* compared to our previous isophthalic acid derivatives, the present results provide useful structure-activity relationship data for further development of ligands targeted to the C1 domain of PKC.

## Introduction

Protein kinase C (PKC) comprises a family of ten phospholipid-dependent serine/threonine kinases [1, 2], which regulate several cellular processes including proliferation, migration, cell survival and apoptosis [3–5]. Due to its central position in intracellular signaling, PKC is also involved in the pathogenesis of various diseases, including diabetes, cancer, ischemic heart disease and heart failure, some autoimmune diseases, Parkinson’s disease and in Alzheimer’s disease [2]. The fact that PKC is linked with so many diseases makes it a very attractive subject of research and a potential target for therapeutic discoveries.

PKC consists of a single polypeptide chain that contains a conserved catalytic kinase domain and a more variable regulatory domain [2]. PKC isoforms are divided into three classes based on differences of their regulatory domain subunit structure and arrangement and the way in which the protein is activated. All conventional PKC isoforms (cPKCs: -*α*, -*β*I, -*β*II and -*γ*), novel PKCs (nPKCs: -*δ*, -*ε*, -*θ* and -*η*) and atypical PKCs (aPKCs: -*ζ*- and -*λ/ι*) require phosphatidylserine (PS) for their activation. In addition to PS, cPKCs require diacylglycerol (DAG) and Ca^2+^ to be activated, while nPKCs are activated in a DAG-dependent and Ca^2+^-independent manner. The structure of the regulatory domain of aPKCs however differs substantially from cPKCs and nPKCs, and therefore neither DAG nor Ca^2+^ is needed for their activation.

The C1 domain region in the regulatory domains of cPKC and nPKC isoforms mediates their translocation to cellular membranes [2]. It is the binding site for DAG and phorbol esters and thus the region of interest for developing PKC modulators. Moreover, as the ATP binding site in the catalytic domain is highly preserved throughout the human kinome, targeting the regulatory C1 domain greatly increases the selectivity for PKC over other kinases [6, 7]. In addition to PKCs, there are only six other protein families, compared to more than 500 protein kinases in the human genome, containing a DAG-responsive C1 domain [8–10]. Throughout the years, several PKC activators showing higher affinity than the natural DAG were described and they represent a significant class of PKC modulators [11]. Natural and semi-synthetic C1 domain ligands (including phorbol esters and bryostatins) are generally complex in their chemical structure, mostly due to the presence of stereocenters and macrocycles. Our group and several others pursued to design and synthesize structurally simpler C1 domain–targeted ligands.

Previously, we developed a set of dialkyl 5-(hydroxymethyl)isophthalate derivatives (HMIs) that modify PKC functions by binding to the C1 domain of the enzyme [12]. Compounds HMI-1a3 and -1b11 are examples of the most potent ligands for PKC*α* and -*δ* (*K*_i_ values in the range 205–915 nM) with marked effects on cultured cells in low micromolar concentrations. In HeLa cervical cancer cells, HMI-1a3 exhibited a marked antiproliferative effect and induced PKC-dependent ERK1/2 phosphorylation. These same effects are induced by both HMI-1a3 and -1b11 in SH-SY5Y neuroblastoma cell-line, together with induction of neurite growth and increased expression of GAP-43, which is a marker for neurite sprouting and neuronal differentiation [13, 14].

In the current work, we focused on improving the drug-likeness of the HMIs by increasing their solubility and enhancing the binding affinity. Hence, we chose to substitute the phenyl core of the HMIs with a heterocycle. In this study, we describe the design, synthesis and structure-activity relationships of novel multisubstituted pyrimidines as analogs of C1 domain–targeted isophthalates.

## Chemistry

### Design

C1 domains (C1a and C1b) function as an anchor stabilizing PKC on the cell membrane [15]. When binding to the C1 domains, phorbol esters contribute to the formation of a continuous hydrophobic surface, which allows the protein-ligand complex to anchor to membranes and stabilize the activated protein-ligand-membrane complex. From two studies on DAG lactones, it appears that the amphipathic properties and the log*P* of a C1 domain–targeted ligand substantially affect the affinity for the protein [16, 17].

**Fig 1:**
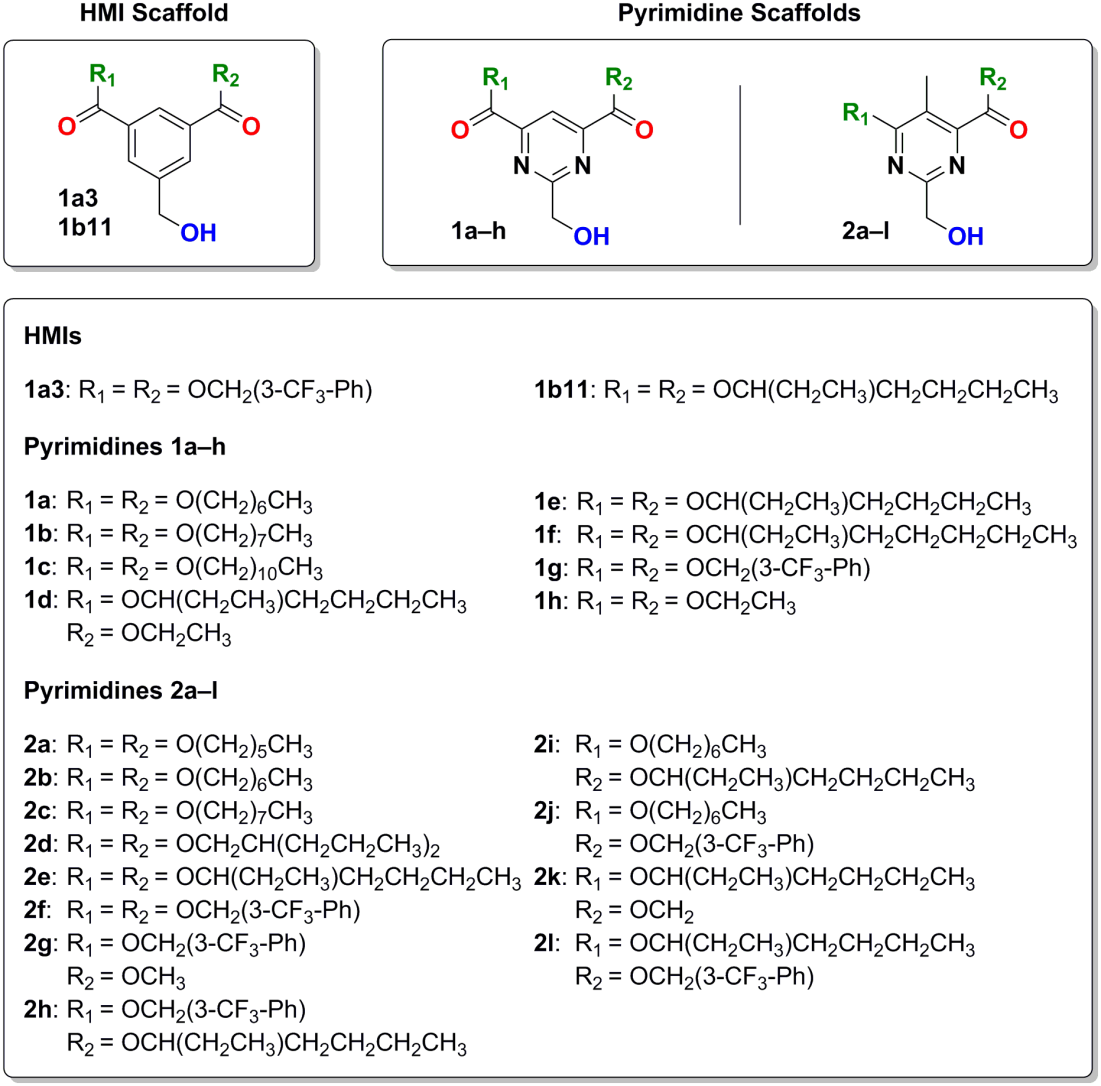
Scaffolds comparison. Comparison of the HMI scaffold (left) with the symmetrical 2,4,6-trisubstituted pyrimidine (center) and the asymmetrical 2,4,5,6-tetrasubstituted pyrimidine (right) scaffolds. Common moieties are color-coded: H-bond donor/acceptor hydroxy groups are shown in blue, carbonyl H-bond acceptor oxygens in red and hydrophobic substituents R in green.

In our previous work, the molecular modeling of the HMIs suggests their interaction with the PKC*δ*C1B domain occurring in a similar manner as for the phorbol esters. The clog*P* (calculated log*P*) of the best HMIs ranges between 6–7 and their affinity for PKC between 205–915 nM [12]. In this study, we substituted the phenyl core of the isophthalates with a pyrimidine moiety to investigate whether the activity of our ligands is affected by increasing their solubility in aqueous buffer but maintaining the amphipathic properties of the HMIs scaffold. We designed two new scaffolds (Fig 1), a symmetrical and an unsymmetrical one which allowed us also to explore different degrees of substitution obtaining 2,4,6-trisubstituted pyrimidines **1a-h** and 2,4,5,6-tetrasubstituted pyrimidines **2a–l**.

The derivatization of the ester moieties of the symmetrical 2,4,6-trisubstituted pyrimidines comprises substituents with increasing length of linear (**1a–c**) and branched (**1d–f**) alkyl chains and the benzylic **1g**, compounds **1g** and **1e** being the corresponding pyrimidine versions of HMI-1a3 and -1b11. We also kept short ethyl derivatives (**1d** and **1h**) to investigate eventual alkyl chain length-dependent loss of activity. The design of the unsymmetrical 2,4,5,6-tetrasubstituted pyrimidines instead focused a deeper investigation on the symmetry-related activity with compounds featuring the same substituents (**2a–f**) or different combinations (**2g–l**) switching them between the ether and ester moieties in positions C4 and C6, respectively.

### Modeling

**Fig 2:**
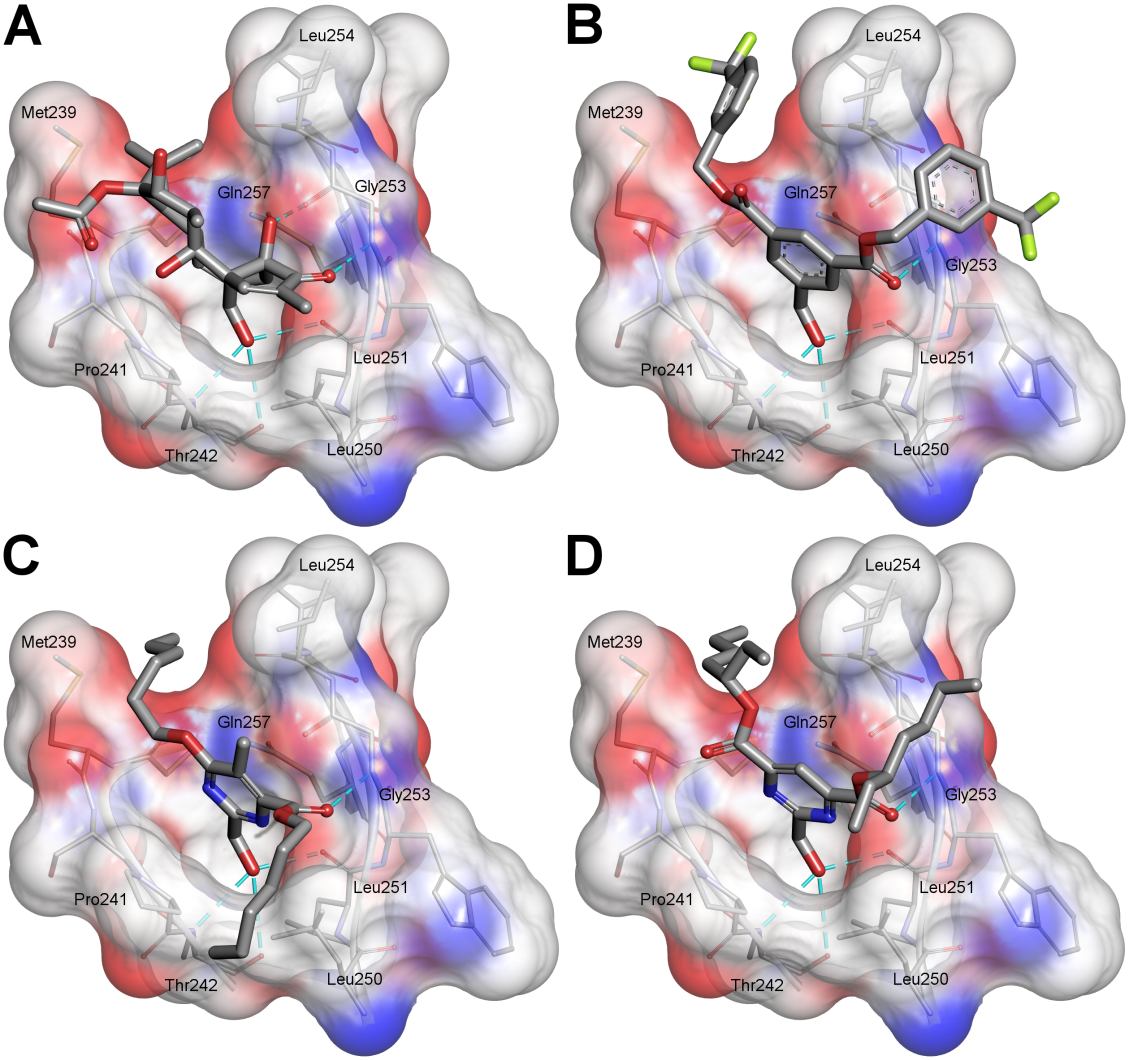
Comparison of phorbol 13-acetate, HMI-1a3, 2b and 1f docked into the PKC*δ*C1B domain (PDB: 1PTR). (A) Phorbol 13-acetate; (B) HMI-1a3; (C) **2b**; (D) **1f**. Color code: carbon atoms are shown in grey, oxygen atoms in red, nitrogen atoms in blue and fluorine atoms in lime. Hydrogen bonds are represented as cyan dashed lines. View from the top of the binding site.

To design a set of pyrimidines we referred to the crystal structure of the phorbol 13-acetate bound PKC*δ*C1B (Protein Data Bank code: 1PTR) [18] and to the knowledge of the key functional groups of the HMIs gained from our previous study [12]. The co-crystallized phorbol acetate forms hydrogen bonds with the amino acids Thr242, Leu251 and Gly253 in the hydrophilic pocket of the C1 domain while it completes the hydrophobic surface of the protein through hydrophobic interactions with Leu251, Leu254 and Met239 (Fig 2A). According to our previous docking study, the HMIs are able to bind to the active site in similar manner showing also a possible additional attractive interaction between Gln257 and the *π*-electrons of the aromatic core (Fig 2B).

When comparing the previous docking poses with those of the new pyrimidines, the interaction pattern between the ligands and the backbone amino acids of the polar pocket (i.e. Thr242, Leu251 and Gly253) (Fig 2) remain alike. The hydrophobic interactions, instead, show a bit more variation, as the pyrimidines may interact also with for instance Pro241 and Leu250 in addition to Leu251, Leu254 and Met239. (Fig 2C and 2D).

### Synthesis

**Fig 3:**
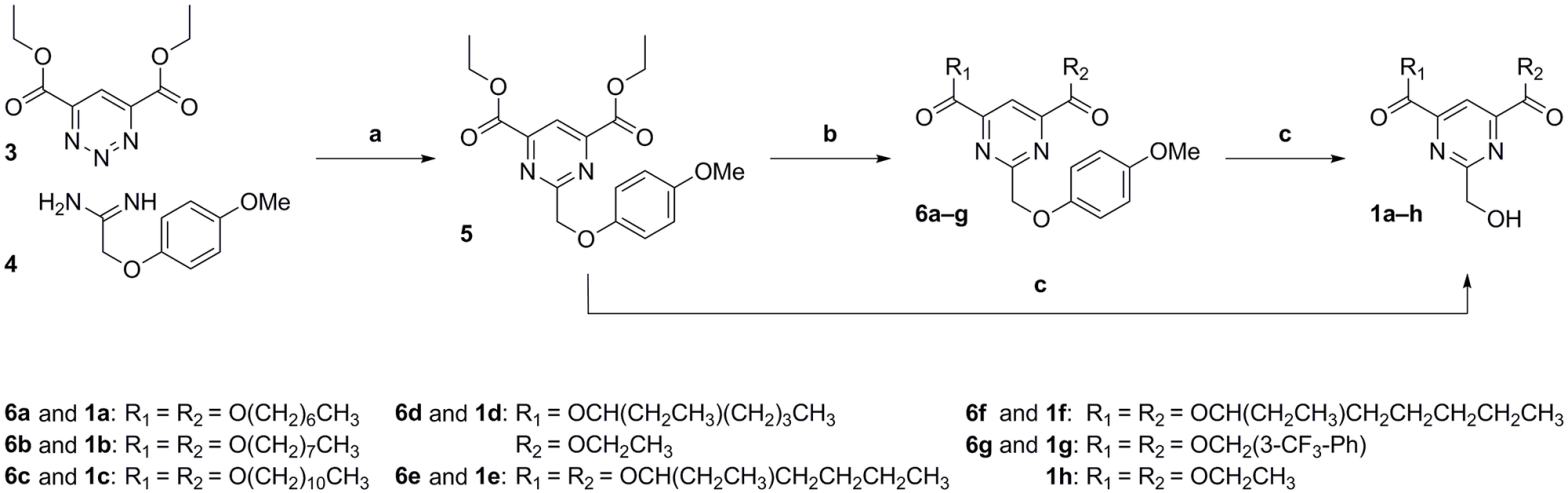
Synthesis and derivatization of the 2,4,6-trisubstituted pyrimidines 1a–h. Conditions: (a) MeCN/1,4-dioxane, rt, 24 h, 63%; (b) alcohol, H_2_SO_4_ (cat.), 100 °C, 3 h, 17–84%; (c) CAN, MeCN/H_2_O, -15 °C, 10 min, 49–80%.

We prepared the symmetrical 2,4,6-trisubstituted pyrimidines in a two to three-step synthesis (Fig 3). We started with an inverse electron demand Diels—Alder reaction reported on related compounds by Duerfeldt, Anderson and coworkers [19, 20]. A commercially available diethyl 1,2,3-triazine-4,6-dicarboxylate (**3**) was reacted with 2-(4-methoxyphenoxy)acetamidine (**4**) to obtain the 2,4,6-trisubstituted pyrimidine **5** containing a *p*-methoxyphenyl (PMP)–protected hydroxymethyl moiety at the C2-position. The PMP protection allows the treatment of **5** with different alcohols in the presence of a catalytic amount of sulfuric acid and transesterification of the esters in positions C4 and C6 to give the intermediates **6a–g**. Finally, the PMP was easily removed by an oxidative cleavage reaction applying conditions reported by Lee [21] with minor modifications. We treated the intermediates **6a–g** with ceric(IV) ammonium nitrate (CAN) to give the desired products **1a–g** while the same conditions applied directly to the intermediate **5** gave the final product **1h**.

**Fig 4:**
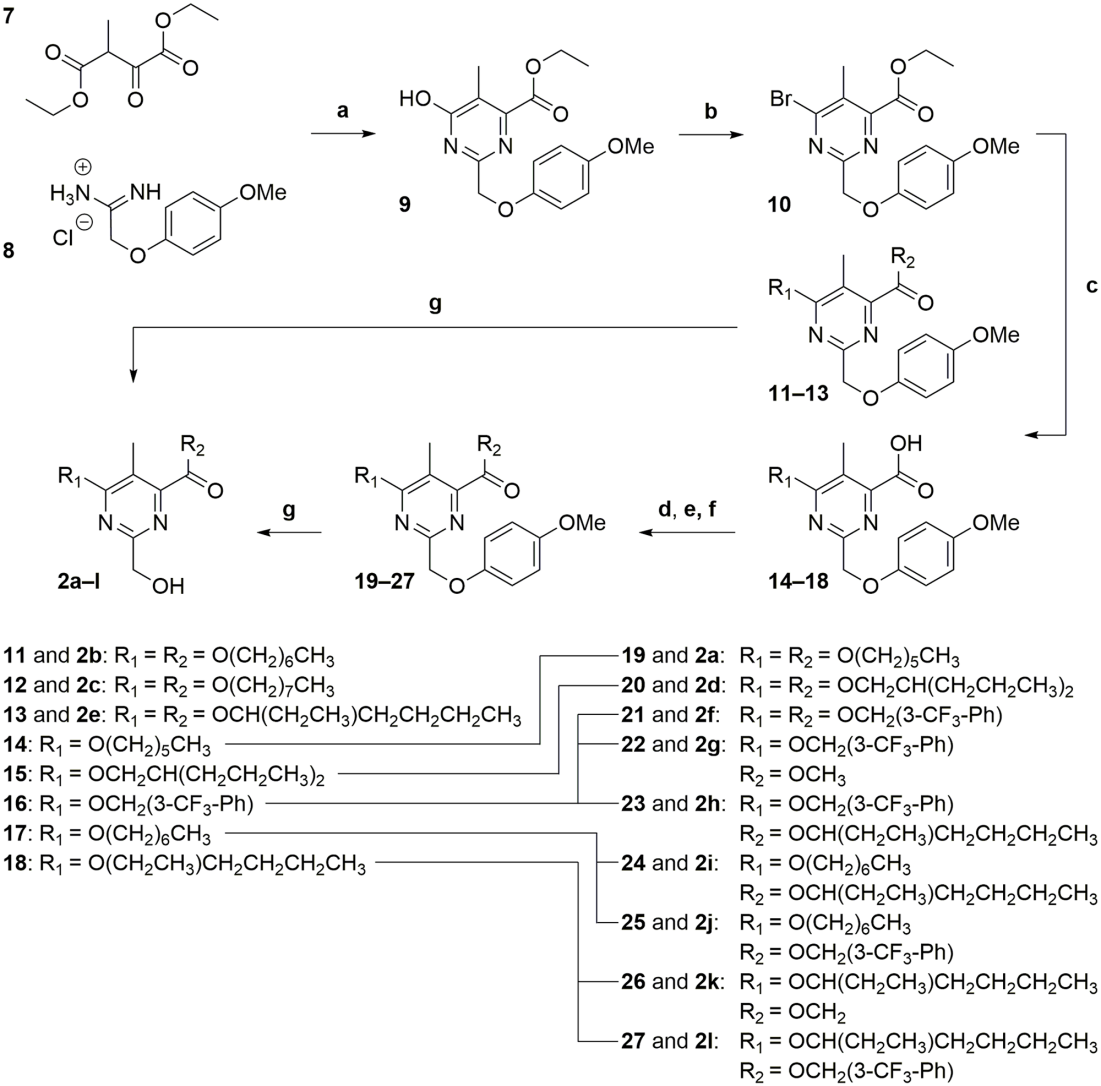
Synthesis and derivatization of the 2,4,5,6-tetrasubstituted pyrimidines 2a–l. Conditions: (a) TEA, EtOH, reflux, 2.5 h, 31%; (b) POBr_3_, DMF, MW 90 °C, 10 min, 75%; (c) alcohol, NaH, THF, 0 °C → rt, overnight (20–22 h), 5–68%; (d) SOCl_2_, alcohol, MW 90 °C, 1 h, 33–50%; (e) CDI, DBU, DMAP, DMF, MW 50 °C, 1 h, 51–74%; (f) 2 M Me_3_SiCHN_2_ in Et_2_O, CH_2_Cl_2_/MeOH, 0 °C, 30 min, 44–100%; (g) CAN, MeCN/H_2_O, -15 °C, 10 min, 17–76%.

We performed a four to five-step synthesis to obtain the unsymmetrical 2,4,5,6-tetrasubstituted pyrimidines (Fig 4). In the first step, reported on related compounds by Otsuka and coworkers [22], we reacted the commercially available diethyl oxalpropionate (**7**) and 2-(4-methoxyphenoxy)acetamidine hydrochloride (**8**) in the presence of triethylamine (TEA) in ethanol to obtain pyrimidine **9** containing a PMP-protected hydroxymethyl moiety in C2-position. The substituted pyrimidine **9** was treated with phosphoryl bromide in *N*,*N*-dimethylformamide (DMF) to give the aryl bromide **10** with the C4-position activated for the subsequent nucleophilic substitution. Different alcohols were treated with NaH to generate the respective alkoxides which reacted with intermediate **10** on both positions C4 and the carbonyl moiety to give pyrimidines **11–13** in low yields. Instead, the carboxylic acids **14–18** were formed during the reaction and were isolated for an esterification reaction to give compounds **19–27**. The carboxyl groups of **14–18** were esterified with different methods including: 1) treatment with SOCl_2_ in an alcohol as a solvent, 2) activation with 1,1’-carbonyldiimidazole (CDI) and treatment with different alcohols in the presence of 1,8-diazabicyclo[5.4.0]undec-7-ene (DBU) and 4-(dimethylamino)pyridine (DMAP) and 3) treatment with trimethylsilyldiazomethane. For the PMP-deprotection step, intermediates **11–13** and **19–27** were treated with CAN to give the final compounds **2a–l**.

### Chemography and ChemGPS-NP

**Fig 5:**
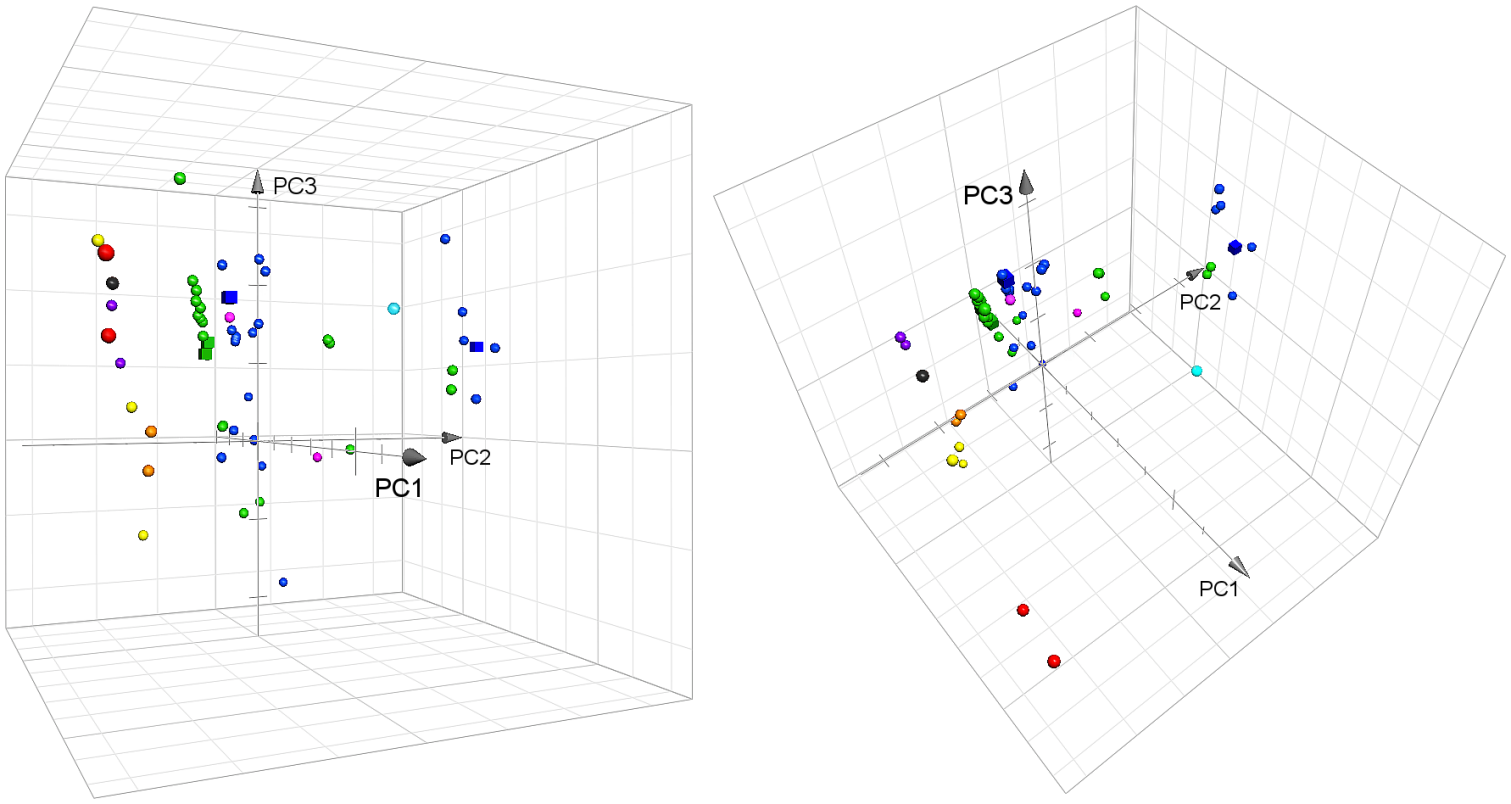
3D chemographic plot of PKC-targeted compounds from two different angles. Color code: Pyrimidines are shown in green; HMIs in blue; bryostatins in red; phorbol esters in yellow; DAG-lactones in purple; iripallidal in black, ingenol 3-angelate and prostratin in orange; mezerein in cyan; 9-decyl-benzolactam-V8 and indolactam-V in magenta. Pyrimidines **1e** and **2a**, HMI-1a3 (towards the PC2-boundary) and HMI-1b11 (central area) are represented as cubes. The full list of the compounds, ChemGPS-NP raw data, SMILES and structures are available in S1 File.

To compare the physicochemical properties of the novel pyrimidines with those of other PKC*α* ligands we carried out a chemographic mapping including also the HMIs and some of the most potent PKC*α* binders (for the complete list of the compounds see Materials and methods and S1 File). We used the ChemGPS-NP_Web_ tool [23–25], a principal component analysis–based chemical global positioning system, which allows to plot organic compounds in a two/three-dimensional chemical space assigning a position based on their structure-derived physicochemical properties. We converted the structures of the compounds into SMILES (simplified molecular-input line-entry system) and uploaded them to the ChemGPS-NP_Web_ server (http://chemgps.bmc.uu.se) which generated for each of them eight principal components (dimensions PC1–8). Each PC describes different physicochemical properties based on 35 descriptors and the four most significant PCs (PC1–4) represent 77% of data variance. PC1 accounts for size, shape and polarizability, PC2 comprises aromaticity and conjugation properties, PC3 includes lipophilicity, polarity, and H-bond capacity while PC4 represents flexibility and rigidity [24]. We plotted the ligands in a three-dimensional space setting PC1, PC2 and PC3 as the x, y and z axes, respectively, with conical arrows indicating the positive sides (Fig 5). The full list of the compounds, ChemGPS-NP raw data, SMILES and structures are available in S1 File.

The 3D-plot shows clearly how most of the best binders, the pyrimidines and HMIs are separated by PC2 in 4 bands, then distributed along PC1 by their size and along PC3 by their lipophilicity. In this analysis PC2 is the most significant dimension and, as explained previously, it represents aromatic and conjugation properties of the compounds: the more aromatic rings/conjugated systems feature in the structure of a compound the higher is the PC2-value the compound obtains. The structures of all the potent binders, except mezerein, 9-decyl-benzolactam-V8 and indolactam-V (Fig 5, cyan and magenta spheres respectively), feature only few *π*-conjugated systems and no aromatic moieties in both core structure and substituents. This explains why they obtained lower PC2-values compared to the other compounds and thus they aligned together on the most negative side of PC2. The aforementioned three potent binders, which instead did not align with the rest of the ligands with high affinity, present a non-aromatic core but some aromatic features in their substituents that explains their higher PC2-values. All the other compounds feature, instead, an aromatic core which increases their PC2-values to form the two central bands of pyrimidines/HMIs bearing aliphatic substituents while those with aromatic substituents clustered at the most positive side of PC2. Then PC2 highlights clearly the lower aromatic contribution of the pyrimidine ring compared to the phenyl ring with all the pyrimidines separated, with lower PC2-values, from their HMI analogs. The alignment of the pyrimidines, slightly closer to the most active compounds compared to the HMIs, suggested that even better activity might occur. Unfortunately, the biological data did not however support this hypothesis.

## Biology

**Fig 6:**
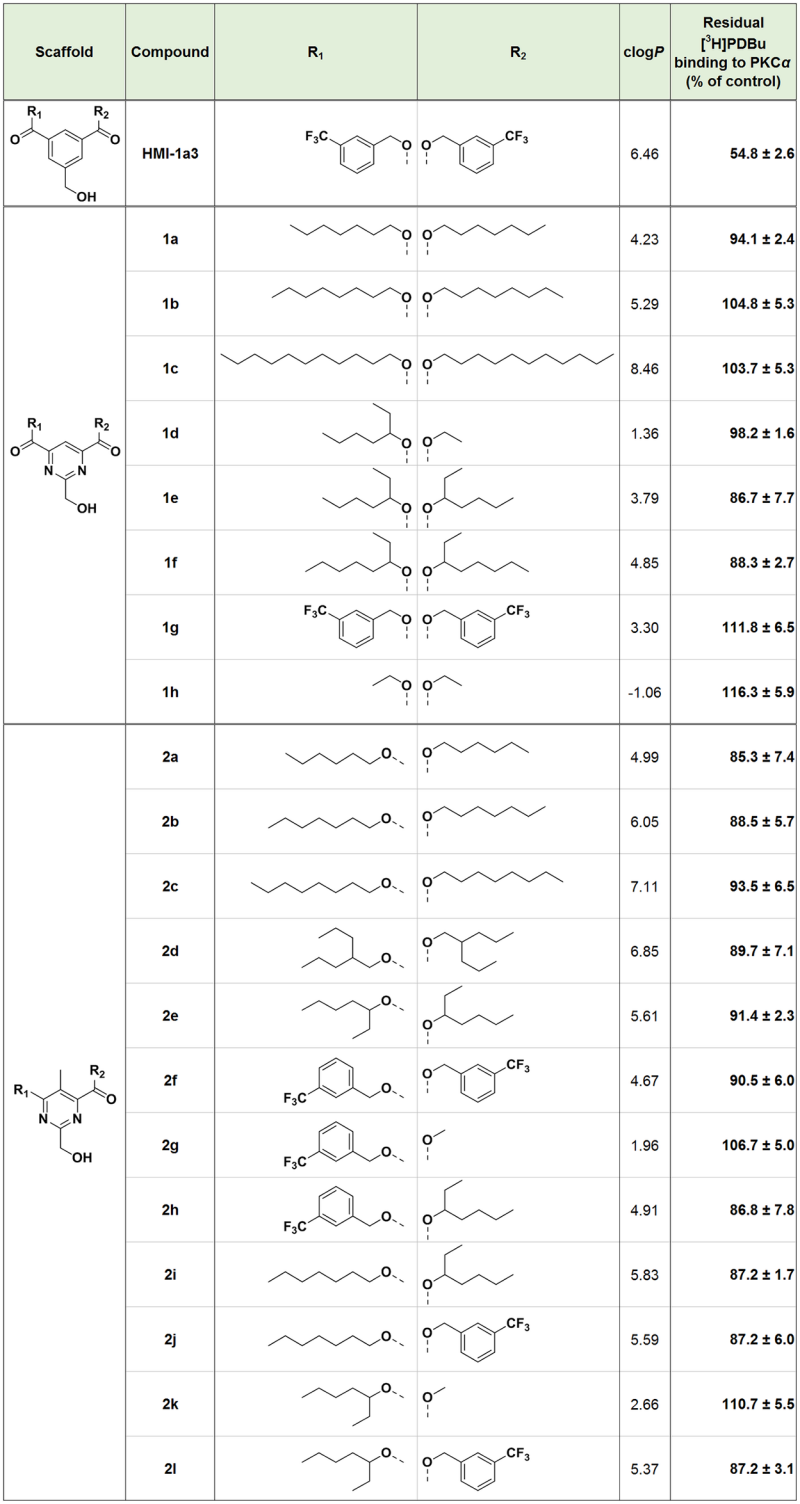
Data comparison for HMI-1a3, symmetrical pyrimidines 1a–h and unsymmetrical pyrimidines 2a–l. Binding affinity of pyrimidine derivatives expressed as the mean + standard error of the mean (SEM) (n = 2–8) of residual [^3^H]PDBu binding (% of control) at 20 μM compound concentration. The raw data of the displacement assay is available in S2 File.

**Fig 7:**
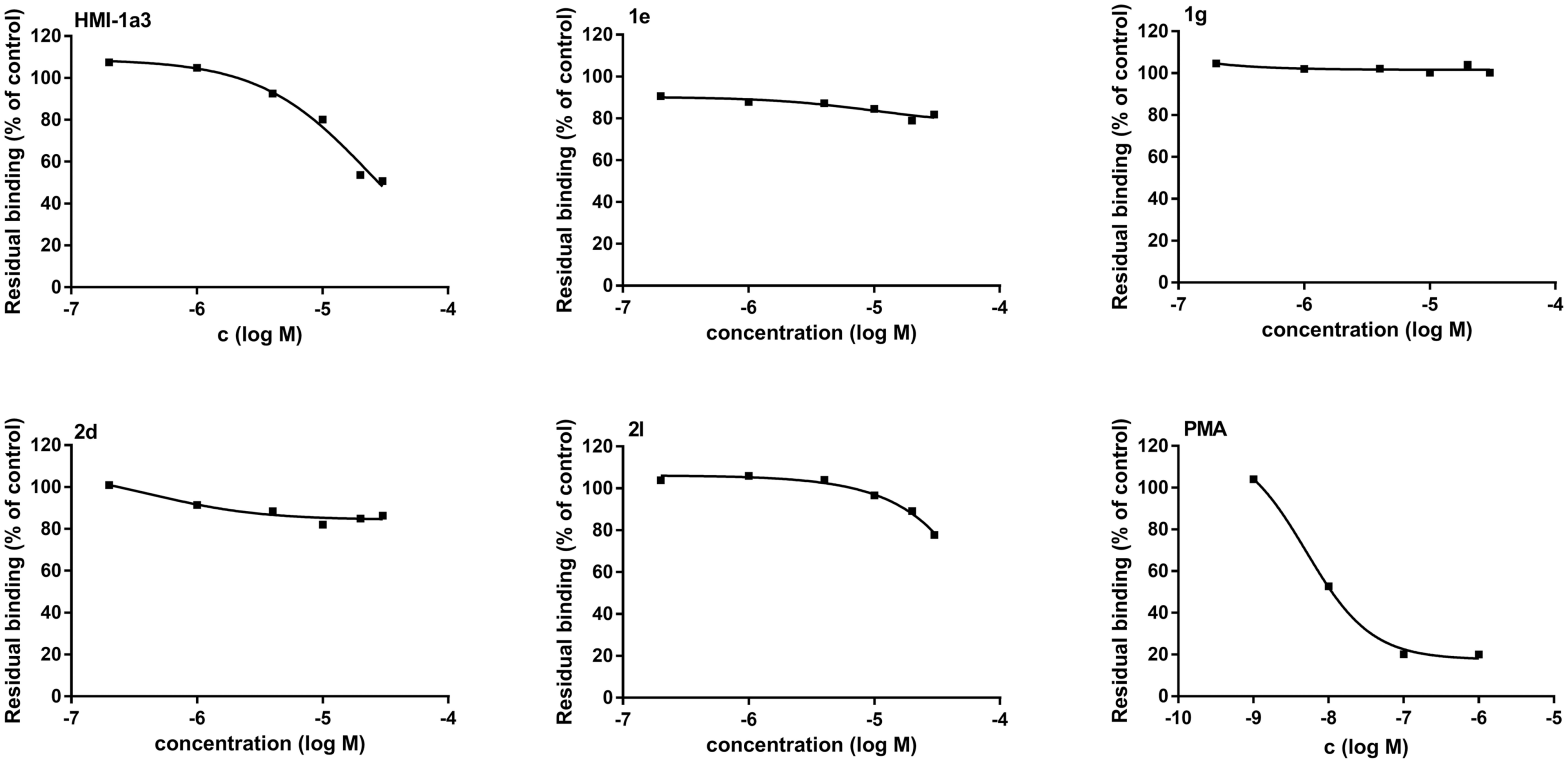
Representative binding curves for HMI 1a3, pyrimidines 1e, 1g, 2d, 2l and PMA. Binding of [^3^H]PDBu (10 nM) to purified PKC*α* measured in the presence of increasing concentrations of the tested compounds. The data is presented as mean of residual [^3^H]PDBu binding (% of control) from three parallel samples in a single representative experiment. The raw data of the displacement assays are available in S2 File.

We tested the compounds for binding to the C1 domains of PKC*α* with a 96-well plate filtration assay as described earlier, at a concentration range of 0.2–30 μM [12, 26]. To our surprise, none of the new compounds displaced [20-^3^H]phorbol-12,13-dibutyrate ([^3^H]PDBu) as efficiently as HMI-1a3. The comparison of the displacement ability between the compounds **1a–c**, **1e**, **1f** and **2a–c** aimed to reveal a correlation between the length of the linear side chain and the binding affinity (Fig 6) (raw data available in S2 File). The differences, however, were very low and no trend can be established. Compounds **1d**, **1h**, **2g**, and **2k** demonstrate that the core structure requires longer alkyl side chains on both sides to achieve detectable binding. Surprisingly, the corresponding pyrimidine version of HMI-1a3, **1g**, could not displace [^3^H]PDBu at the concentration range used (Fig 7) (raw data available in S2 File). However, the HMI-1b11 analog **1e** was one of the most effective novel compounds to displace [^3^H]PDBu from PKC*α*. Its affinity was however considerably lower than that of HMI-1b11 determined in our previous work [12]. Compounds **2a** and **2l** showed the strongest concentration dependence (Fig 7). In terms of lipophilicity, most of the novel compounds showed a lower clog*P* value compared to HMI-1a3 (Fig 6).

## Discussion and conclusion

The C1 domain of PKC represents a potential target for discovery of therapeutic drugs for diseases with unmet medical needs [6]. Plant and animal derived natural C1 domain ligands, such as phorbol esters and bryostatins, show high affinity and biological activity but they are not optimal drug candidates as their complex chemical structures make their synthesis tedious. In our previous work, we have demonstrated that simple 5-hydroxymethyl isophthalic acid derivatives exhibit promising biological activity [9, 10, 12, 13]. The lipophilicity values for the HMIs (clog*P* 6–7) are however higher than the Lipinski’s drug-like lipophilic property value (log*P* ≤5) [27] and therefore, we endeavored to synthesize a new set of compounds with reduced lipophilicity and retained/increased binding affinity.

In the present study, we designed and developed a novel set of PKC C1 domain–targeted pyrimidines. Despite their similarity to the HMIs in terms of structure and predicted binding mode, they were not able to displace [^3^H]PDBu from the C1 domain of PKC*α* at the concentration range tested. Surprisingly, not even **1e** and **1g** showed similar binding to PKC*α* as the corresponding HMI-1b11 and HMI-1a3, respectively. This overall outcome was not expected based on the docking model of the pyrimidines, which returned docking scores in the same range as for the HMIs and suggested the same binding interactions. In addition, the chemographic data from the ChemGPS-NP 3D-plot displayed the pyrimidines slightly closer to the more potent binding compounds suggesting, at first, a possible activity. In line with the negative feedback from the biological data, a reinterpretation of the chemographic study highlights that (1) the overall lower affinity of the HMIs, compared to more potent ligands, may be due to the aromatic/planar nature of the core structure; (2) the presence of aromatic substituents have no effect or may favor the affinity; and (3) the scaffold hopping towards a heterocycle, pyrimidine in this case, caused the loss of activity.

In addition to PKCα, the HMIs bind to PKC*δ* and other protein families containing a DAG-responsive C1 domain (e.g. β-chimaerin, protein kinase D1 and myotonic dystrophy kinase-related Cdc42-binding kinase [MRCK]) at comparable affinities [9]. The present work demonstrates the binding affinities of the pyrimidines only for PKC*α*. As many other C1 domain ligands, these compounds might show substantial differences in binding affinity towards different PKC isoforms or single C1 domains [28]. However, due to the analogy with the HMIs we expect that the almost complete lack of binding of the pyrimidines to PKC*α* may indicate only weak or no affinity to other C1 domains as well. This is why we did not proceed to characterize the binding of pyrimidines for those.

To improve the affinity and selectivity of C1 domain ligands Ohashi and coworkers recently presented a novel set of dimeric DAG-lactone derivatives [17]. These dimeric lactones showed no enhanced binding affinity to the full-length PKC*α* or -*δ* compared to their monomeric constructs, and they indicated higher lipophilicity (clog*P* values: 10.7–16.7). However, they showed stronger binding to the individual PKC*δ*C1B domain than the monomer. Physiological relevance of this finding is unclear, as affinity for the full-length protein was not increased. Elhalem and coworkers studied the C1 domain selectivity of indololactones, bearing a heterocyclic ring at the *sn-1* or *sn-2* position, for PKC*α*, -*δ* and Ras guanine nucleotide-releasing protein (RasGRP1) [29]. They demonstrated selectivity for RasGRP1 over PKC*α* when the indole ring is in the *sn-2* position of indololactones [30]. Binding affinity for PKC*α*, -*δ* and RasGRP1 as well as selectivity for RasGRP1 decreased when substituted at the *sn-1* position compared to the *sn-2* position. These results encourage a further pharmacophore optimization for the design and synthesis of novel C1 domain targeted ligands to achieve improved binding affinity and selectivity for PKCs and other C1 domain-containing targets.

Taken together, we demonstrated previously that the isophthalate derivatives show affinity for the C1 domains of cPKCs and nPKCs and possess promising biological activities in cell culture models related to cancer and Alzheimer’s disease. In an attempt to improve the aqueous solubility of the C1 domain ligands, we prepared a set of 2,4,6-trisubstituted–and 2,4,5,6-tetrasubstituted pyrimidines, bearing similar hydrophobic substituents as the isophthalates, and quantified their binding to PKC*α*. We can conclude that the novel pyrimidine analogs did not establish improved binding affinity for PKC*α* compared to the most promising isophthalates and the lower binding affinity of the isophthalates, compared to more potent ligands, may correlate to the aromatic/planar nature of their core structure. Results presented here, however, provide useful SAR data for further development of ligands targeted to the C1 domain of PKC.

## Materials and methods

### Modeling

We docked our 22 compounds to the crystal structure of the C1B domain of PKC*δ* (PDB ID: 1PTR) using Glide of Schrödinger Maestro [31] with SP parameters. The targeted binding site was defined by the mass center of the co-crystallized ligand, phorbol 13-acetate, which was also used as a reference compound in docking. Prior to the docking, the target protein was prepared with Maestro’s Protein preparation tool, and 3D coordinates of the compounds were calculated by Schrödinger’s LigPrep utilizing Epik to generate protonation states. For scoring, we used Glide’s “docking score”.

### Syntheses

All reagents were acquired from Sigma-Aldrich (Schnelldorf, Germany), Fluorochem (Hadfield, United Kingdom) and Fluka (Buchs, Switzerland), and were used without further purification. All reactions in anhydrous conditions were conducted using dry solvents in oven-dried glassware under an inert atmosphere of dry argon. The progress of chemical reactions was monitored by thin-layer chromatography on Silica Gel 60 F254 aluminum sheets acquired from Merck (Darmstadt, Germany), visualized under UV light (254/366 nm) and stained with phosphomolybdic acid (10% w/v in EtOH). Microwave reactions were performed with a Biotage Initiator^+^ SP *Wave* Microwave Synthesizer (Uppsala, Sweden). Flash SiO_2_ column chromatography was performed with an automated high performance flash chromatography Biotage Sp1-system equipped with a 0.1-mm path length flow cell UV-detector/recorder module (fixed wavelength 254 nm) or with a Biotage Isolera^™^ Spektra Systems with ACI^™^ and Assist (ISO-1SW Isolera One) equipped with a variable UV-VIS (200–800 nm) photodiode array (Uppsala, Sweden), and the indicated mobile phase gradient. ^1^H, ^13^C and ^19^F NMR spectra (also available in S1 Appendix including ^13^C HSQC, ^13^C HMBC and ^15^N HMBC 2D NMR spectra) were acquired on a Bruker Ascend 400 MHz—Avance III HD NMR spectrometer (Bruker Corporation, Billerica, MA, USA) as solutions in CDCl_3_. Chemical shifts (*δ*) are reported as parts per million (ppm) relative to the solvent peaks at 7.26 and 77.16 ppm for ^1^H and ^13^C NMR respectively. Multiplicities of peaks are represented by s (singlet), d (doublet), t (triplet), q (quartet), quint (quintet), and m (multiplet). Visual features of peaks including broad (br) or apparent (app) are also indicated. In ^13^C NMR data, peaks referring to two symmetrical carbons (sym, 2C) or two different carbons with overlapping signals (2C) are also indicated. All spectra were processed for recorded FID files with MestReNova 11.0.4 software (Mestrelab Research, Santiago de Compostela, Spain). Low resolution mass (MS-APCI) analyses were performed on a MS Advion expression^®^ CMS spectrometer equipped with an APCI ion source and an Atmospheric Solids Analysis Probe (ASAP) and the data was reported for the molecular ions [M+H]^+^. Exact mass and purity (>95%) of all tested compounds was confirmed by LC-MS analyses with a Waters Acquity^®^ UPLC system (Waters, Milford, MA, USA) equipped with an Acquity UPLC^®^ BEH C18 column (1.7 μm, 50 × 2.1 mm, Waters, Ireland), an Acquity PDA detector and a Waters Synapt G2 HDMS mass spectrometer (Waters, Milford, MA, USA) via an ESI ion source in positive mode. High resolution mass (HRMS-ESI) data was reported for the molecular ions [M+H]^+^. The clog*P* values of the compounds were calculated with ChemDraw Professional 16.0.0.82 software (PerkinElmer Informatics, Waltham, MA, USA).

#### Diethyl 2-[(4-methoxyphenoxy)methyl]pyrimidine-4,6-dicarboxylate (5)

Diethyl 1,2,3-triazine-4,6-dicarboxylate (**3**) (252 mg, 1.12 mmol, 2 equiv) was dissolved in dry MeCN (2.4 mL) and stirred with crushed molecular sieves (4 Å) under argon atmosphere. Meanwhile, a solution of the free base amidine **4** (101 mg, 0.560 mmol) in dry 1,4-dioxane (2 mL) and dry MeCN (2.4 mL) was added dropwise to the first mixture. Nitrogen and subsequent ammonia evolution occurred and the color of the mixture turned from orange to black. After stirring the reaction mixture for 24 h at rt the solvents were evaporated under reduced pressure. The black residue was taken up in EtOAc and washed with water. The organic layer was dried with anhydrous Na_2_SO_4_, filtrated and the solvent evaporated under reduced pressure. The residue was purified by flash column chromatography [cyclohexane (A), EtOAc (B); gradient: 6% → 30% B×10 CV] to give **5** (129 mg, 0.357 mmol, 63.7% yield) as a dark yellow solid. *R*_f_ 0.42 (cyclohexane/EtOAc 2:1 + 2% TEA). ^1^H NMR (400 MHz, CDCl_3_) *δ*_ppm_ 8.51 (app t, *J* = 0.6 Hz, 1H), 7.02–6.93 (m, 2H), 6.85–6.76 (m, 2H), 5.42 (s, 2H), 4.52 (q, *J* = 7.1 Hz, 4H), 3.75 (s, 3H), 1.45 (t, *J* = 7.1 Hz, 6H). ^13^C NMR (101 MHz, CDCl_3_) *δ*_ppm_ 167.9, 163.5 (sym, 2C), 158.1 (sym, 2C), 154.4, 152.6, 119.1, 116.4 (sym, 2C), 114.7 (sym, 2C), 71.5, 63.2 (sym, 2C), 55.8, 14.3 (sym, 2C). MS-APCI (*m/z*): [M+H]^+^ 361.1.

#### General procedure I: Acid-catalyzed transesterification

Compound **5** was dissolved in alcohol (13–16 equiv) and heated to 100 °C for 3 h in the presence of a catalytic amount of H_2_SO_4_ (0.1 equiv). Complete dissolution occurred while heating. The reaction was quenched by adding a saturated solution of NaHCO_3_ in water and the mixture was extracted with EtOAc. The organic layers were combined, and the solvent was evaporated under reduced pressure at 40 °C. The residual alcohol was removed by vacuum distillation. The crude residue was purified by flash column chromatography with appropriate eluents and a gradient.

#### Diheptyl 2-[(4-methoxyphenoxy)methyl]pyrimidine-4,6-dicarboxylate (6a)

General procedure I was followed. Compound **5** (73 mg, 0.20 mmol), 1-heptanol (0.50 mL, 3.2 mmol, 16 equiv), H_2_SO_4_ (1 μL, 0.03 mmol, 0.1 equiv). Flash chromatography eluents: *n*-hexane (A), Et_2_O (B); gradient: 8% → 66% B×10 CV. Compound **6a** was isolated as a dark yellow oil (81 mg, 0.16 mmol, 80% yield). *R*_f_ 0.20 (*n*-hexane/Et_2_O 2:1). ^1^H NMR (400 MHz, CDCl_3_) *δ*_ppm_ 8.48 (app t, *J* = 0.6 Hz, 1H), 7.02–6.95 (m, 2H), 6.85–6.77 (m, 2H), 5.42 (s, 2H), 4.44 (t, *J* = 6.9 Hz, 4H), 3.75 (s, 3H), 1.82 (quint, *J* = 6.9 Hz, 4H), 1.49–1.21 (m, 16H), 0.89 (app t, *J* = 7.0 Hz, 6H). ^13^C NMR (101 MHz, CDCl_3_) *δ*_ppm_ 168.0, 163.6 (sym, 2C), 158.1 (sym, 2C), 154.4, 152.6, 119.1, 116.4 (sym, 2C), 114.7 (sym, 2C), 71.5, 67.3 (sym, 2C), 55.8, 31.8 (sym, 2C), 29.0 (sym, 2C), 28.6 (sym, 2C), 25.9 (sym, 2C), 22.7 (sym, 2C), 14.2 (sym, 2C). MS-APCI (*m/z*): [M+H]^+^ 501.7.

#### Dioctyl 2-[(4-methoxyphenoxy)methyl]pyrimidine-4,6-dicarboxylate (6b)

General procedure I was followed. Compound **5** (0.100 g, 0.277 mmol), 1-octanol (0.700 mL, 4.44 mmol, 16.0 equiv), H_2_SO_4_ (2 μL, 0.04 mmol, 0.1 equiv). Flash chromatography eluents: *n*-hexane (A), EtOAc (B); gradient: 4% → 30% B×9 CV. Compound **6b** was isolated as a dark yellow oil (0.120 g, 0.227 mmol, 81.9% yield). *R*_f_ 0.41 (*n*-hexane/EtOAc 5:1). ^1^H NMR (400 MHz, CDCl_3_) *δ*_ppm_ 8.48 (app t, *J* = 0.6 Hz, 1H), 7.04–6.89 (m, 2H), 6.88–6.73 (m, 2H), 5.42 (s, 2H), 4.44 (t, *J* = 6.9 Hz, 4H), 3.75 (s, 3H), 1.82 (quint, *J* = 6.9 Hz, 4H), 1.49–1.14 (m, 20H), 0.88 (app t, *J* = 7.0 Hz, 6H). ^13^C NMR (101 MHz, CDCl_3_) *δ*_ppm_ 168.0, 163.6 (sym, 2C), 158.1 (sym, 2C), 154.4, 152.6, 119.1, 116.4 (sym, 2C), 114.7 (sym, 2C), 71.5, 67.3 (sym, 2C), 55.8, 31.9 (sym, 2C), 29.32 (sym, 2C), 29.28 (sym, 2C), 28.6 (sym, 2C), 26.0 (sym, 2C), 22.8 (sym, 2C), 14.2 (sym, 2C). MS-APCI (*m/z*): [M+H]^+^ 529.6.

#### Diundecyl 2-[(4-methoxyphenoxy)methyl]pyrimidine-4,6-dicarboxylate (6c)

General procedure I was followed. Compound **5** (73.0 mg, 0.202 mmol), 1-undecanol (673 μL, 3.24 mmol, 16.0 equiv), H_2_SO_4_ (1 μL, 0.03 mmol, 0.1 equiv). Flash chromatography eluents: cyclohexane (A), Et_2_O (B); gradient: 6% → 50% B×10 CV + 50% B×5 CV. Compound **6c** was isolated as a yellow oil (65.2 mg, 0.106 mmol, 52.5% yield). *R*_f_ 0.25 (cyclohexane/Et_2_O 3:1). ^1^H NMR (400 MHz, CDCl_3_) *δ*_ppm_ 8.48 (s, 1H), 7.02–6.95 (m, 2H), 6.85–6.78 (m, 2H), 5.43 (s, 2H), 4.44 (t, *J* = 6.9 Hz, 4H), 3.76 (s, 3H), 1.82 (quint, *J* = 6.9 Hz, 4H), 1.48–1.17 (m, 32H), 0.87 (app t, *J* = 6.7 Hz, 6H). ^13^C NMR (101 MHz, CDCl_3_) *δ*_ppm_ 168.0, 163.6 (sym, 2C), 158.1 (sym, 2C), 154.4, 152.6, 119.1, 116.4 (sym, 2C), 114.7 (sym, 2C), 71.5, 67.3 (sym, 2C), 55.8, 32.0 (sym, 2C), 29.74 (sym, 2C), 29.72 (sym, 2C), 29.6 (sym, 2C), 29.5 (sym, 2C), 29.4 (sym, 2C), 28.6 (sym, 2C), 26.0 (sym, 2C), 22.8 (sym, 2C), 14.3 (sym, 2C). MS-APCI (*m/z*): [M+H]^+^ 613.5.

#### 4-ethyl 6-(heptan-3-yl) 2-[(4-methoxyphenoxy)methyl]pyrimidine-4,6-dicarboxylate (6d) and di(heptan-3-yl) 2-[(4-methoxyphenoxy)methyl]pyrimidine-4,6-dicarboxylate (6e)

General procedure I was followed. Compound **5** (58 mg, 0.16 mmol), 3-heptanol (0.301 mL, 2.12 mmol, 13.1 equiv), H_2_SO_4_ (1 μL, 0.02 mmol, 0.1 equiv). Flash chromatography eluents: cyclohexane (A), EtOAc (B); gradient: 8% → 52% B×8 CV. Compound **6d** was isolated as a yellow oil (12 mg, 0.027 mmol, 17% yield). *R*_f_ 0.55 (cyclohexane/EtOAc 2:1). ^1^H NMR (400 MHz, CDCl_3_) *δ*_ppm_ 8.47 (app t, *J* = 0.6 Hz, 1H), 7.05–6.90 (m, 2H), 6.88–6.76 (m, 2H), 5.43 (s, 2H), 5.17 (app quint, *J* = 6.1 Hz, 1H), 4.53 (q, *J* = 7.1 Hz, 2H), 3.75 (s, 3H), 1.85–1.57 (m, 4H), 1.46 (t, *J* = 7.1 Hz, 3H), 1.45–1.21 (m, 4H), 0.95 (t, *J* = 7.4 Hz, 3H), 0.89 (app t, *J* = 7.0 Hz, 3H). ^13^C NMR (101 MHz, CDCl_3_) *δ*_ppm_ 168.0, 163.7, 163.2, 158.4, 158.0, 154.4, 152.7, 119.0, 116.5 (sym, 2C), 114.7 (sym, 2C), 79.2, 71.5, 63.2, 55.8, 33.3, 27.6, 27.1, 22.7, 14.3, 14.1, 9.8. MS-APCI (m/z): [M+H]^+^ 431.4. Compound **6e** was isolated as a yellow oil (41 mg, 0.081 mmol, 51% yield). *R*_f_ 0.75 (cyclohexane/EtOAc 2:1). ^1^H NMR (400 MHz, CDCl_3_) *δ*_ppm_ 8.42 (app t, *J* = 0.6 Hz, 1H), 7.07–6.92 (m, 2H), 6.88–6.70 (m, 2H), 5.44 (s, 2H), 5.16 (app quint, *J* = 6.1 Hz, 2H), 3.75 (s, 3H), 1.92–1.55 (m, 8H), 1.42–1.22 (m, 8H), 0.95 (t, *J* = 7.4 Hz, 6H), 0.89 (app t, *J* = 7.1 Hz, 6H). ^13^C NMR (101 MHz, CDCl_3_) *δ*_ppm_ 168.0, 163.4 (sym, 2C), 158.3 (sym, 2C), 154.4, 152.7, 118.8, 116.6 (sym, 2C), 114.6 (sym, 2C), 79.1 (sym, 2C), 71.5, 55.8, 33.3 (sym, 2C), 27.6 (sym, 2C), 27.0 (sym, 2C), 22.7 (sym, 2C), 14.1 (sym, 2C), 9.8 (sym, 2C). MS-APCI (*m/z*): [M+H]^+^ 501.6.

#### Di(octan-3-yl) 2-[(4-methoxyphenoxy)methyl]pyrimidine-4,6-dicarboxylate (6f)

General procedure I was followed. Compound **5** (73 mg, 0.20 mmol), 3-octanol (0.516 mL, 3.24 mmol, 16.0 equiv), H_2_SO_4_ (1 μL, 0.03 mmol, 0.1 equiv). Flash chromatography eluents: *n*-hexane (A), Et_2_O (B); gradient: 8% → 66% B×10 CV + 66% B×2 CV. Compound **6f** was isolated as a dark yellow oil (85 mg, 0.17 mmol, 84% yield). *R*_f_ 0.37 (*n*-hexane/Et_2_O 3:1). ^1^H NMR (400 MHz, CDCl_3_) *δ*_ppm_ 8.42 (app t, *J* = 0.6 Hz, 1H), 7.03–6.95 (m, 2H), 6.84–6.77 (m, 2H), 5.44 (s, 2H), 5.16 (app quint, *J* = 6.2 Hz, 2H), 3.75 (s, 3H), 1.83–1.59 (m, 8H), 1.46–1.21 (m, 12H), 0.95 (t, *J* = 7.4 Hz, 6H), 0.87 (app t, *J* = 6.8 Hz, 6H). ^13^C NMR (101 MHz, CDCl_3_) *δ*_ppm_ 168.0, 163.4 (sym, 2C), 158.3 (sym, 2C), 154.4, 152.7, 118.8, 116.6 (sym, 2C), 114.6 (sym, 2C), 79.1 (sym, 2C), 71.6, 55.8, 33.6 (sym, 2C), 31.8 (sym, 2C), 27.0 (sym, 2C), 25.1 (sym, 2C), 22.6 (sym, 2C), 14.1 (sym, 2C), 9.8 (sym, 2C). MS-APCI (*m/z*): [M+H]^+^ 529.1.

#### Bis[3-(trifluoromethyl)benzyl] 2-[(4-methoxyphenoxy)methyl]pyrimidine-4,6-dicarboxylate (6g)

General procedure I was followed. Compound **5** (52 mg, 0.14 mmol), 3-(trifluoromethyl)benzyl alcohol (0.300 mL, 2.21 mmol, 15.3 equiv), H_2_SO_4_ (1 μL, 0.02 mmol, 0.1 equiv). Flash chromatography eluents: cyclohexane (A), acetone (B); gradient: 8% → 38% B×10 CV. Compound **6g** was isolated as a dark yellow oil (57 mg, 0.092 mmol, 64% yield). *R*_f_ 0.25 (cyclohexane/acetone 2:1). ^1^H NMR (400 MHz, CDCl_3_) *δ*_ppm_ 8.53 (app t, *J* = 0.6 Hz, 1H), 7.73 (s, 2H), 7.69–7.60 (m, 4H), 7.52 (t, *J* = 7.7 Hz, 2H), 7.02–6.89 (m, 2H), 6.83–6.74 (m, 2H), 5.51 (s, 4H), 5.43 (s, 2H), 3.74 (s, 3H). ^13^C NMR (101 MHz, CDCl_3_) *δ*_ppm_ 168.3, 163.2 (sym, 2C), 157.6 (sym, 2C), 154.5, 152.5, 135.7 (sym, 2C), 132.1 (app q, *J* = 1.1 Hz, sym, 2C), 131.3 (q, *J* = 32.5 Hz, sym, 2C), 129.5 (sym, 2C), 125.8 (q, *J* = 3.7 Hz, sym, 2C), 125.5 (q, *J* = 3.8 Hz, sym, 2C), 124.0 (q, *J* = 272.3 Hz, sym, 2C), 119.4, 116.4 (sym, 2C), 114.7 (sym, 2C), 71.3, 67.7 (sym, 2C), 55.8. MS-APCI (*m/z*): [M+H]^+^ 621.6.

#### General procedure II: *p*-Methoxyphenyl deprotection

Ceric(IV) ammonium nitrate (3 equiv) was added to a cooled (-15 °C) solution of a PMP-protected compound in CH_3_CN/H_2_O 4:1 (0.4–2.8 mL) and stirred for 10 min. The mixture was diluted with water and extracted with EtOAc. The combined organic layers were washed with brine, dried with anhydrous Na_2_SO_4_ and filtrated. The solvent was evaporated under reduced pressure at 40 °C. The residual hydroquinone was removed by high vacuum. The crude residue was purified by flash column chromatography with appropriate eluents and a gradient.

#### Diheptyl 2-(hydroxymethyl)pyrimidine-4,6-dicarboxylate (1a)

General procedure II was followed. Compound **6a** (62 mg, 0.12 mmol), CH_3_CN/H_2_O 4:1 (1.8 mL). Flash chromatography eluents: *n*-hexane (A), Et_2_O (B); gradient: 12% → 100% B×10 CV. Compound **1a** was isolated as an orange oil (29 mg, 0.074 mmol, 60% yield). *R*_f_ 0.35 (*n*-hexane/Et_2_O 1:1). ^1^H NMR (400 MHz, CDCl_3_) *δ*_ppm_ 8.46 (app t, *J* = 0.8 Hz, 1H), 5.03 (app d, *J* = 0.8 Hz, 2H), 4.45 (t, *J* = 6.9 Hz, 4H), 3.16 (br s, 1H), 1.82 (quint, *J* = 6.8 Hz, 4H), 1.55–1.16 (m, 16H), 0.88 (app t, *J* = 6.8 Hz, 6H). ^13^C NMR (101 MHz, CDCl_3_) *δ*_ppm_ 170.4, 163.4 (sym, 2C), 157.7 (sym, 2C), 118.7, 67.3 (sym, 2C), 64.9, 31.8 (sym, 2C), 29.0 (sym, 2C), 28.6 (sym, 2C), 25.9 (sym, 2C), 22.7 (sym, 2C), 14.2 (sym, 2C). MS-APCI (*m/z*): [M+H]^+^ 395.3. HRMS-ESI (*m/z*): [M+H]^+^ calcd for C_21_H_35_N_2_O_5_ 395.2546; found 395.2545.

#### Dioctyl 2-(hydroxymethyl)pyrimidine-4,6-dicarboxylate (1b)

General procedure II was followed. Compound **6b** (0.100 g, 0.189 mmol), CH_3_CN/H_2_O 4:1 (2.8 mL). Flash chromatography eluents: *n*-hexane (A), EtOAc (B); gradient: 6% → 44% B×13 CV. Compound **1b** was isolated as an orange oil (57.9 mg, 0.137 mmol, 72.4% yield). *R*_f_ 0.42 (*n*-hexane/EtOAc 5:1). ^1^H NMR (400 MHz, CDCl_3_) *δ*_ppm_ 8.46 (app t, *J* = 0.8 Hz, 1H), 5.04 (d, *J* = 4.5 Hz, 2H), 4.45 (t, *J* = 6.8 Hz, 4H), 3.62 (t, *J* = 5.4 Hz, 1H), 1.82 (quint, *J* = 7.2 Hz, 4H), 1.51–1.18 (m, 20H), 0.88 (app t, *J* = 6.8 Hz, 6H). ^13^C NMR (101 MHz, CDCl_3_) *δ*_ppm_ 170.4, 163.4 (sym, 2C), 157.7 (sym, 2C), 118.7, 67.3 (sym, 2C), 64.9, 31.9 (sym, 2C), 29.3 (sym, 2C), 29.3 (sym, 2C), 28.6 (sym, 2C), 26.0 (sym, 2C), 22.8 (sym, 2C), 14.2 (sym, 2C). MS-APCI (*m/z*): [M+H]^+^ 423.3. HRMS-ESI (*m/z*): [M+H]^+^ calcd for C_23_H_38_N_2_O_5_ 423.2859; found 423.2858.

#### Diundecyl 2-(hydroxymethyl)pyrimidine-4,6-dicarboxylate (1c)

General procedure II was followed. Compound **6c** (50.0 mg, 0.0816 mmol), CH_3_CN/H_2_O 4:1 (1.2 mL). Flash chromatography eluents: *n*-hexane (A), Et_2_O (B); gradient: 12% → 100% B×10 CV. Compound **1c** was isolated as an orange oil (25.8 mg, 0.0509 mmol, 62.4% yield). *R*_f_ 0.19 (*n*-hexane/Et_2_O 1:1). ^1^H NMR (400 MHz, CDCl_3_) *δ*_ppm_ 8.46 (app t, *J* = 0.7 Hz, 1H), 5.04 (s, 2H), 4.45 (t, *J* = 6.8 Hz, 4H), 3.59 (br s, 1H), 1.82 (quint, *J* = 7.1 Hz, 4H), 1.54–1.15 (m, 32H), 0.87 (t, *J* = 7.2 Hz, 6H). ^13^C NMR (101 MHz, CDCl_3_) *δ*_ppm_ 170.4, 163.4 (sym, 2c), 157.7 (sym, 2c), 118.8, 67.3 (sym, 2c), 64.9, 32.0 (sym, 2c), 29.73 (sym, 2c), 29.71 (sym, 2c), 29.6 (sym, 2c), 29.5 (sym, 2c), 29.4 (sym, 2c), 28.6 (sym, 2c), 26.0 (sym, 2c), 22.8 (sym, 2c), 14.3 (sym, 2c). MS-APCI (*m/z*): [M+H]^+^ 507.7. HRMS-ESI (*m/z*): [M+Na]^+^ calcd for C_29_H_50_N_2_O_5_Na 529.3618; found 529.3619.

#### 4-ethyl 6-(heptan-3-yl) 2-(hydroxymethyl)pyrimidine-4,6-dicarboxylate (1d)

General procedure II was followed. Compound **6d** (11 mg, 0.026 mmol), CH_3_CN/H_2_O 4:1 (0.4 mL). Flash chromatography eluents: cyclohexane (A), EtOAc (B); gradient: 12% → 92% B×9 CV. Compound **1d** was isolated as a pale yellow oil (4.8 mg, 0.014 mmol, 53% yield). *R*_f_ 0.4 (cyclohexane/EtOAc 1:1). ^1^H NMR (400 MHz, CDCl_3_) *δ*_ppm_^1^H NMR (400 MHz, CDCl_3_) δ 8.46 (app t, *J* = 0.8 Hz, 1H), 5.18 (app quint, *J* = 6.1 Hz, 1H), 5.04 (app d, *J* = 0.8 Hz, 2H), 4.53 (q, *J* = 7.1 Hz, 2H), 3.55 (br s, 1H), 1.84–1.59 (m, 4H), 1.46 (t, *J* = 7.1 Hz, 3H), 1.42–1.27 (m, 4H), 0.97 (t, *J* = 7.4 Hz, 3H), 0.90 (app t, *J* = 6.9 Hz, 3H). ^13^C NMR (101 MHz, CDCl_3_) *δ*_ppm_ 170.3, 163.5, 163.1, 157.9, 157.7, 118.7, 79.3, 64.9, 63.2, 33.4, 27.6, 27.1, 22.7, 14.3, 14.1, 9.8. MS-APCI (*m/z*): [M+H]^+^ 325.2. HRMS-ESI (*m/z*): [M+H]^+^ calcd for C_16_H_25_N_2_O_5_ 325.1764; found 325.1766.

#### Di(heptan-3-yl) 2-(hydroxymethyl)pyrimidine-4,6-dicarboxylate (1e)

General procedure II was followed. Compound **6e** (0.040 g, 0.080 mmol), CH_3_CN/H_2_O 4:1 (1.2 mL). Flash chromatography eluents: cyclohexane (A), EtOAc (B); gradient: 8% → 60% B×9 CV. Compound **1e** was isolated as an orange oil (25 mg, 0.064 mmol, 80% yield). *R*_f_ 0.5 (cyclohexane/EtOAc 2:1). ^1^H NMR (400 MHz, CDCl_3_) *δ*_ppm_^1^H NMR (400 MHz, CDCl_3_) δ 8.41 (app t, *J* = 0.8 Hz, 1H), 5.16 (app quint, *J* = 6.2 Hz, 2H), 5.02 (d, *J* = 5.3 Hz, 2H), 3.71 (t, *J* = 5.3 Hz, 1H), 1.84–1.59 (m, 8H), 1.43–1.23 (m, 8H), 0.95 (t, *J* = 7.4 Hz, 6H), 0.88 (app t, *J* = 7.1 Hz, 6H). ^13^C NMR (101 MHz, CDCl_3_) *δ*_ppm_ 170.3, 163.2 (sym, 2C), 157.9 (sym, 2C), 118.5, 79.2 (sym, 2C), 64.8, 33.3 (sym, 2C), 27.6 (sym, 2C), 27.0 (sym, 2C), 22.6 (sym, 2C), 14.1 (sym, 2C), 9.8 (sym, 2C). MS-APCI (*m/z*): [M+H]^+^ 395.3. HRMS-ESI (*m/z*): [M+H]^+^ calcd for C_21_H_35_N_2_O_5_ 395.2546; found 395.2544.

#### Di(octan-3-yl) 2-(hydroxymethyl)pyrimidine-4,6-dicarboxylate (1f)

General procedure II was followed. Compound **6f** (63.1 mg, 0.126 mmol), CH_3_CN/H_2_O 4:1 (1.8 mL). Flash chromatography eluents: *n*-hexane (A), Et_2_O (B); gradient: 12% → 100% B×10 CV. Compound **1f** was isolated as an orange oil (41.1 mg, 0.0973 mmol, 77.2% yield). *R*_f_ 0.35 (*n*-hexane/Et_2_O 1:1). ^1^H NMR (400 MHz, CDCl_3_) *δ*_ppm_^1^H NMR (400 MHz, CDCl_3_) δ 8.42 (app t, *J* = 0.7 Hz, 1H), 5.18 (app quint, *J* = 6.1 Hz, 2H), 5.03 (d, *J* = 0.6 Hz, 2H), 3.33 (s, 1H), 2.05–1.51 (m, 8H), 1.50–1.10 (m, 12H), 0.96 (t, *J* = 7.4 Hz, 6H), 0.87 (app t, *J* = 7.0 Hz, 6H). ^13^C NMR (101 MHz, CDCl_3_) *δ*_ppm_ 170.3, 163.3 (sym, 2C), 157.9 (sym, 2C), 118.6, 79.3 (sym, 2C), 64.9, 33.6 (sym, 2C), 31.7 (sym, 2C), 27.1 (sym, 2C), 25.1 (sym, 2C), 22.6 (sym, 2C), 14.1 (sym, 2C), 9.8 (sym, 2C). MS-APCI (*m/z*): [M+H]^+^ 423.3. HRMS-ESI (*m/z*): [M+H]^+^ calcd for C_23_H_39_N_2_O_5_ 423.2859; found 423.2857.

#### Bis[3-(trifluoromethyl)benzyl] 2-(hydroxymethyl)pyrimidine-4,6-dicarboxylate (1g)

General procedure II was followed. Compound **6g** (56 mg, 0.090 mmol), CH_3_CN/H_2_O 4:1 (1.3 mL). Flash chromatography eluents: cyclohexane (A), EtOAc (B); gradient: 12% → 100% B×10 CV. Compound **1f** was isolated as a yellow oil (30 mg, 0.047 mmol, 52% yield). *R*_f_ 0.25 (cyclohexane/EtOAc 1:1). ^1^H NMR (400 MHz, CDCl_3_) *δ*_ppm_ 8.50 (app t, *J* = 0.7 Hz, 1H), 7.72 (s, 2H), 7.66 (d, *J* = 7.6 Hz, 2H), 7.63 (d, *J* = 7.9 Hz, 2H), 7.53 (t, *J* = 7.7 Hz, 2H), 5.51 (s, 4H), 5.03 (s, 2H), 3.60 (br s, 1H). ^13^C NMR (101 MHz, CDCl_3_) *δ*_ppm_ 170.7, 163.0 (sym, 2C), 157.2 (sym, 2C), 135.6 (sym, 2C), 132.1 (app q, *J* = 1.1 Hz, sym, 2C), 131.4 (q, *J* = 32.6 Hz, sym, 2C), 129.5 (sym, 2C), 125.9 (q, *J* = 3.7 Hz, sym, 2C), 125.6 (q, *J* = 3.9 Hz, sym, 2C), 123.9 (q, *J* = 272.4 Hz, sym, 2C), 119.0, 67.7 (sym, 2C), 64.9. ^19^F NMR (376 MHz, CDCl_3_) *δ*_ppm_ -62.73. MS-APCI (*m/z*): [M+H]^+^ 515.1. HRMS-ESI (*m/z*): [M+H]^+^ calcd for C_23_H_17_N_2_O_5_F_6_ 515.1042; found 515.1042.

#### Diethyl 2-(hydroxymethyl)pyrimidine-4,6-dicarboxylate (1h)

General procedure II was followed. Compound **6h** (16 mg, 0.043 mmol), CH_3_CN/H_2_O 4:1 (0.6 mL). Flash chromatography eluents: cyclohexane (A), EtOAc (B); gradient: 20% → 35% B×15 CV + 35% → 100% B×15 CV. Compound **1h** was isolated as a yellow oil (5.4 mg, 0.021 mmol, 49% yield). *R*_f_ 0.15 (cyclohexane/EtOAc 3:2). ^1^H NMR (400 MHz, CDCl_3_) *δ*_ppm_ 8.49 (app q, *J* = 0.7 Hz, 1H), 5.04 (s, 2H), 4.52 (q, *J* = 7.1 Hz, 4H), 3.61 (s, 1H), 1.46 (t, *J* = 7.1 Hz, 6H). ^13^C NMR (101 MHz, CDCl_3_) *δ*_ppm_ 170.4, 163.4 (sym, 2C), 157.6 (sym, 2C), 118.8, 64.9, 63.2 (sym, 2C), 14.3 (sym, 2C). HRMS-ESI (*m/z*): [M+H]^+^ calcd for C_11_H_15_N_2_O_5_ 255.0981; found 255.0981.

#### Ethyl 6-hydroxy-2-[(4-methoxyphenoxy)methyl]-5-methylpyrimidine-4-carboxylate (9)

Diethyl oxalpropionate (0.932 mL, 4.95 mmol) was added to a solution of 2-(4-methoxyphenoxy)acetamidine hydrochloride (1.18 g, 5.44 mmol, 1.1 equiv) in ethanol (20 mL) and triethylamine (1.52 mL, 10.9 mmol, 2.2 equiv) and refluxed for 2.5 h under argon atmosphere. The yellow solution turned brown approaching the reflux point. The solvent was evaporated under reduced pressure at 40 °C. The residue was taken up with EtOAc, water (15 mL) was added and the mixture was extracted with EtOAc (3×25 mL). The combined organic layers were washed with brine (3×25 mL) and the solvent was evaporated under reduced pressure at 40 °C. The residue was purified by flash column chromatography [cyclohexane (A), EtOAc (B); gradient: 25% → 40% B×7 CV + 40% → 100% B×7 CV] to give **9** (488 mg, 1.53 mmol, 30.9% yield) as a yellow solid. *R*_f_ 0.45 (cyclohexane/EtOAc 1:1). ^1^H NMR (400 MHz, CDCl_3_) *δ*_ppm_ 11.05 (br s, 1H), 6.98–6.89 (m, 2H), 6.89–6.79 (m, 2H), 4.98 (s, 2H), 4.43 (q, *J* = 7.1 Hz, 2H), 3.76 (s, 3H), 2.25 (s, 3H), 1.41 (t, *J* = 7.1 Hz, 3H). ^13^C NMR (101 MHz, CDCl_3_) *δ*_ppm_ 165.5, 163.5, 155.1, 154.7, 151.1, 150.0, 125.3, 115.9 (sym, 2C), 115.0 (sym, 2C), 67.3, 62.4, 55.8, 14.3, 11.8. MS-APCI (*m/z*): [M+H]^+^ 319.3. HRMS-ESI (*m/z*): [M+H]^+^ calcd for C_16_H_19_N_2_O_5_ 319.1294; found 319.1296.

#### Ethyl 6-bromo-2-[(4-methoxyphenoxy)methyl]-5-methylpyrimidine-4-carboxylate (10)

Phosphoryl bromide (1.80 g, 6.28 mmol, 2 equiv) was added to a solution of **9** (1.00 g, 3.14 mmol) in DMF (10 mL) and the mixture was microwave irradiated for 10 min at 90 °C. The yellow color of the mixture turned dark brown. The reaction was quenched by adding ice water (25 mL) and the mixture was extracted with EtOAc (3×25 mL). The combined organic layers were washed with brine (3×25 mL) and the solvent was evaporated under reduced pressure at 50 °C. The residue was purified by flash column chromatography [cyclohexane (A), EtOAc (B); gradient: 8% → 66% B×10] to give **10** (0.900 g, 2.36 mmol, 75.1% yield) as a yellow solid. *R*_f_ 0.65 (cyclohexane/EtOAc 2:1). ^1^H NMR (400 MHz, CDCl_3_) *δ*_ppm_^1^H NMR (400 MHz, CDCl_3_) δ 7.04–6.88 (m, 2H), 6.88–6.73 (m, 2H), 5.20 (s, 2H), 4.47 (q, *J* = 7.2 Hz, 2H), 3.76 (s, 3H), 2.50 (s, 3H), 1.42 (t, *J* = 7.2 Hz, 3H). ^13^C NMR (101 MHz, CDCl_3_) *δ*_ppm_ 164.6, 164.0, 158.2, 156.9, 154.5, 152.6, 130.2, 116.4 (sym, 2C), 114.7 (sym, 2C), 71.0, 62.9, 55.8, 17.8, 14.2. MS-APCI (*m/z*): [M+H]^+^ 381.2. HRMS-ESI (*m/z*): [M+H]^+^ calcd for C_16_H_18_N_2_O_4_Br 381.0450; found 381.0450.

#### General procedure III: Nucleophilic substitutions on 10 by alkoxides

An alcohol (2.25–6.1 equiv) was added dropwise to a cooled suspension of NaH (60% dispersion in mineral oil; 2.2–6 equiv) in dry THF (0.5–1.5 mL) at 0 °C and the mixture was stirred for 1 h under argon atmosphere. A solution of **10** in dry THF (0.5–1.5 mL) was added dropwise and mixture was stirred overnight letting to warm up to rt. The reaction was quenched with ice water and acidified with a solution of KHSO_4_ until pH≈3. The aqueous phase was extracted with EtOAc. The combined organic layers were washed with brine and the solvent was evaporated under reduced pressure at 40 °C. The crude residue was purified by flash column chromatography with appropriate eluents and a gradient.

#### Heptyl 6-(heptyloxy)-2-[(4-methoxyphenoxy)methyl]-5-methylpyrimidine-4-carboxylate (11)

General procedure III was followed except that the mixture containing the alkoxide was added dropwise to the solution of **10**. NaH (60% in mineral oil; 69.2 mg, 1.73 mmol, 2.2 equiv), dry THF (1.25 mL), 1-heptanol (0.250 mL, 1.77 mmol, 2.25 equiv); compound **10** (0.300 g, 0.787 mmol), dry THF (1.25 mL). Flash chromatography eluents: cyclohexane (A), EtOAc (B); gradient: 10% → 30% B×15 CV. Compound **11** was isolated as an orange oil (46.6 mg, 0.0958 mmol, 12.2% yield). *R*_f_ 0.9 (cyclohexane/EtOAc 2:1). ^1^H NMR (400 MHz, CDCl_3_) *δ*_ppm_ 6.98–6.86 (m, 2H), 6.86–6.75 (m, 2H), 5.14 (s, 2H), 4.37 (t, *J* = 6.9 Hz, 2H), 4.35 (t, *J* = 6.7 Hz, 2H), 3.75 (s, 3H), 2.27 (s, 3H), 1.78 (quint, *J* = 6.9 Hz, 2H), 1.71 (quint, *J* = 7.2 Hz, 2H), 1.47–1.17 (m, 16H), 0.89 (app t, *J* = 6.9 Hz, 3H), 0.88 (app t, *J* = 6.8 Hz, 3H). ^13^C NMR (101 MHz, CDCl_3_) *δ*_ppm_ 169.3, 165.8, 163.1, 155.0, 154.1, 152.9, 116.6, 116.1 (sym, 2C), 114.6 (sym, 2C), 71.2, 67.7, 66.4, 55.8, 31.9, 31.8, 29.1, 29.0, 28.74, 28.67, 26.04, 25.98, 22.73, 22.71, 14.22, 14.20, 11.1. MS-APCI (*m/z*): [M+H]^+^ 487.5.

#### Octyl 2-[(4-methoxyphenoxy)methyl]-5-methyl-6-(octyloxy)pyrimidine-4-carboxylate (12)

General procedure III was followed except that the mixture containing the alkoxide was added dropwise to the solution of **10**. NaH (60% in mineral oil; 46.7 mg, 1.18 mmol, 2.2 equiv), dry THF (1 mL), 1-octanol (187 μL, 1.18 mmol, 2.25 equiv); compound **10** (0.200 g, 0.525 mmol), dry THF (1 mL). Flash chromatography eluents: cyclohexane (A), EtOAc (B); gradient: 10% → 30% B×15 CV. Compound **12** was isolated as an orange oil (0.040 g, 0.078 mmol, 15% yield). *R*_f_ 0.9 (cyclohexane/EtOAc 2:1). ^1^H NMR (400 MHz, CDCl_3_) *δ*_ppm_ 7.02–6.86 (m, 2H), 6.86–6.69 (m, 2H), 5.13 (s, 2H), 4.37 (t, *J* = 6.8 Hz, 2H), 4.35 (t, *J* = 6.6 Hz, 2H), 3.75 (s, 3H), 2.27 (s, 3H), 1.77 (quint, *J* = 7.0 Hz, 2H), 1.71 (quint, *J* = 7.1 Hz, 2H), 1.47–1.33 (m, 4H), 1.35–1.19 (m, 16H), 0.88 (app t, *J* = 6.7 Hz, 3H), 0.88 (app t, *J* = 6.9 Hz, 3H). ^13^C NMR (101 MHz, CDCl_3_) *δ*_ppm_ 169.3, 165.9, 163.1, 155.1, 154.1, 152.9, 116.6, 116.1 (sym, 2C), 114.6 (sym, 2C), 71.2, 67.7, 66.4, 55.8, 31.94, 31.91, 29.39, 29.35, 29.33, 29.30, 28.74, 28.66, 26.1, 26.0, 22.79, 22.77, 14.24, 14.23, 11.1.

#### Heptan-3-yl 6-(heptan-3-yloxy)-2-[(4-methoxyphenoxy)methyl]-5-methylpyrimidine-4-carboxylate (13) and 6-(heptan-3-yloxy)-2-[(4-methoxyphenoxy)methyl]-5-methylpyrimidine-4-carboxylic acid (18)

General procedure III was followed except that the alcohol was used as solvent. NaH (60% in mineral oil; 80.8 mg, 2.02 mmol, 2.2 equiv), 3-heptanol (1.5 mL); compound **10** (0.350 g, 0.918 mmol), 3-heptanol (2.5 mL). Flash chromatography eluents: cyclohexane (A), EtOAc (B); gradient: 3% → 28% B×15 CV + 28% B×4 CV. Compound **13** was isolated as a dark red oil (23 mg, 0.047 mmol, 5.1% yield). *R*_f_ 0.9 (cyclohexane/EtOAc 2:1). ^1^H NMR (400 MHz, CDCl_3_) *δ*_ppm_ 6.98–6.84 (m, 2H), 6.84–6.69 (m, 2H), 5.19 (quint, *J* = 5.9 Hz, 1H), 5.13 (s, 2H), 5.12 (app quint, *J* = 6.0 Hz, 1H), 3.74 (s, 3H), 2.22 (s, 3H), 1.79–1.63 (m, 4H), 1.67–1.51 (m, 4H), 1.47–1.29 (m, 4H), 1.32–1.13 (m, 4H), 0.98 (t, *J* = 7.4 Hz, 3H), 0.91 (app t, *J* = 7.0 Hz, 3H), 0.85 (app t, *J* = 6.8 Hz, 3H), 0.84 (t, *J* = 7.4 Hz, 3H).^13^C NMR (101 MHz, CDCl_3_) *δ*_ppm_ 169.2, 166.1, 163.1, 156.2, 154.0, 153.0, 116.0 (sym, 2C), 115.5, 114.6 (sym, 2C), 78.2, 78.1, 70.9, 55.8, 33.3, 33.0, 27.6, 27.4, 27.0, 26.6, 22.74, 22.72, 14.1 (2C), 11.1, 9.8, 9.5. Compound **18** was isolated as an orange solid (115 mg, 0.296 mmol, 32.2% yield). *R*_f_ 0.1 (cyclohexane/EtOAc 3:1 + 2% AcOH). ^1^H NMR (400 MHz, CDCl_3_) *δ*_ppm_ 9.04 (br s, 1H), 6.98–6.85 (m, 2H), 6.85–6.71 (m, 2H), 5.25 (quint, *J* = 5.9 Hz, 1H), 5.11 (s, 2H), 3.76 (s, 3H), 2.57 (s, 3H), 1.78–1.59 (m, 4H), 1.38–1.17 (m, 4H), 0.97–0.80 (m, 6H). ^13^C NMR (101 MHz, CDCl_3_) *δ*_ppm_ 170.6, 163.7, 161.6, 154.4, 152.6, 148.6, 120.9, 115.9 (sym, 2C), 114.8 (sym, 2C), 79.5, 70.2, 55.8, 32.9, 27.5, 26.6, 22.7, 14.1, 10.7, 9.6.

#### 6-(hexyloxy)-2-[(4-methoxyphenoxy)methyl]-5-methylpyrimidine-4-carboxylic acid (14)

General procedure III was followed. NaH (60% in mineral oil; 189 mg, 4.72 mmol, 6 equiv), dry THF (1.5 mL), 1-hexanol (603 μL, 4.80 mmol, 6.1 equiv); compound **10** (0.300 g, 0.786 mmol), dry THF (1.5 mL). Flash chromatography eluents: CHCl_3_ (A), CHCl_3_/MeOH 20:1 + 1% AcOH (B); gradient: 0% → 20% B×15 CV. Compound **14** was isolated as a pale yellow solid (65 mg, 0.17 mmol, 22% yield). *R*_f_ 0.5 (CHCl_3_/MeOH 20:1 + 1% AcOH). ^1^H NMR (400 MHz, CDCl_3_) *δ*_ppm_ 9.25 (br s, 1H), 7.02–6.85 (m, 2H), 6.85–6.68 (m, 2H), 5.12 (s, 2H), 4.40 (t, *J* = 6.6 Hz, 2H), 3.76 (s, 3H), 2.57 (s, 3H), 1.77 (quint, *J* = 6.8 Hz, 2H), 1.53–1.20 (m, 6H), 0.90 (app t, *J* = 6.8 Hz, 3H). ^13^C NMR (101 MHz, CDCl_3_) *δ*_ppm_ 170.6, 163.6, 161.7, 154.4, 152.5, 148.4, 120.8, 115.9 (sym, 2C), 114.8 (sym, 2C), 70.3, 68.5, 55.8, 31.6, 28.6, 25.7, 22.7, 14.1, 10.7.

#### 2-[(4-methoxyphenoxy)methyl]-5-methyl-6-[(2-propylpentyl)oxy]pyrimidine-4-carboxylic acid (15)

General procedure III was followed. NaH (60% in mineral oil; 177 mg, 4.42 mmol, 6 equiv), dry THF (1.5 mL), 2-propyl-1-pentanol (706 μL, 4.49 mmol, 6.1 equiv); compound **10** (281 mg, 0.737 mmol), dry THF (1.5 mL). Flash chromatography eluents: cyclohexane (A), EtOAc (B); gradient: 0% → 100% B×20 CV. Compound **15** was isolated as a pale yellow solid (79 mg, 0.19 mmol, 26% yield). *R*_f_ 0.2 (cyclohexane/EtOAc 3:1 + 2% AcOH). ^1^H NMR (400 MHz, CDCl_3_) *δ*_ppm_ 8.90 (br s, 1H), 6.94–6.86 (m, 2H), 6.85–6.77 (m, 2H), 5.12 (s, 2H), 4.30 (d, *J* = 5.6 Hz, 2H), 3.76 (s, 3H), 2.57 (s, 3H), 1.91–1.75 (m, 1H), 1.46–1.28 (m, 8H), 0.96–0.86 (m, 6H). ^13^C NMR (101 MHz, CDCl_3_) *δ*_ppm_ 170.7, 163.8, 161.7, 154.4, 152.5, 148.7, 120.8, 116.0 (sym, 2C), 114.8 (sym, 2C), 71.1, 70.3, 55.8, 37.1, 33.8 (sym, 2C), 20.1 (sym, 2C), 14.5 (sym, 2C), 10.7.

#### 2-[(4-methoxyphenoxy)methyl]-5-methyl-6-[[3-(trifluoromethyl)benzyl]oxy]pyrimidine-4-carboxylic acid (16)

General procedure III was followed. NaH (60% in mineral oil; 54.2 mg, 2.26 mmol, 2.2 equiv), dry THF (0.5 mL), 3-(trifluoromethyl)benzyl alcohol (419 μL, 3.08 mmol, 5 equiv); compound **10** (235 mg, 0.616 mmol), dry THF (0.5 mL). Flash chromatography eluents: cyclohexane (A), EtOAc (B); gradient: 0% → 100% B×20 CV. Compound **16** was isolated as a yellow solid (160 mg, 0.357 mmol, 57.9% yield). *R*_f_ 0.2 (cyclohexane/EtOAc 3:1 + 2% AcOH). ^1^H NMR (400 MHz, CDCl_3_) *δ*_ppm_ 9.47 (br s, 1H), 7.71 (s, 1H), 7.62 (d, *J* = 7.6 Hz, 2H), 7.50 (t, *J* = 7.7 Hz, 1H), 6.99–6.86 (m, 2H), 6.86–6.76 (m, 2H), 5.52 (s, 2H), 5.16 (s, 2H), 3.77 (s, 3H), 2.63 (s, 3H). ^13^C NMR (101 MHz, CDCl_3_) *δ*_ppm_ 170.0, 163.2, 161.8, 154.5, 152.5, 149.0, 136.6, 131.7 (d, *J* = 1.1 Hz), 129.3, 125.4 (d, *J* = 3.9 Hz), 125.2 (d, *J* = 3.7 Hz), 121.1, 115.9 (sym, 2C), 114.9 (sym, 2C), 70.2, 68.8, 55.8, 10.8 (the quartets of C-CF_3_ quaternary carbons with *J* ≈ 32 Hz and *J* ≈ 273 Hz could not be identified due to a low signal/noise ratio; for the same reason, the quartets of the adjacent carbons with *J* = 3.9 Hz, *J* = 3.8 and *J* = 1.1 Hz are indicated as doublets). ^19^F NMR (376 MHz, CDCl_3_) *δ*_ppm_ -62.69. MS-APCI (*m/z*): [M+H]^+^ 449.3.

#### 6-(heptyloxy)-2-[(4-methoxyphenoxy)methyl]-5-methylpyrimidine-4-carboxylic acid (17)

General procedure III was followed except that the alcohol was used as solvent and THF as cosolvent. NaH (60% in mineral oil; 57.7 mg, 1.44 mmol, 2.2 equiv), 1-heptanol (1.5 mL); compound **10** (0.250 g, 0.656 mmol), 1-heptanol (1.3 mL), dry THF (1.5 mL). Flash chromatography eluents: cyclohexane (A), EtOAc (B) + 2% AcOH; gradient: 3% → 100% B×10 CV. Compound **17** was isolated as a pale yellow solid (172 mg, 0.444 mmol, 67.7% yield). *R*_f_ 0.5 (EtOAc + 2% AcOH). ^1^H NMR (400 MHz, CDCl_3_) *δ*_ppm_ 9.37 (br s, 1H), 6.93–6.87 (m, 2H), 6.85–6.78 (m, 2H), 5.13 (s, 2H), 4.41 (t, *J* = 6.6 Hz, 2H), 3.76 (s, 3H), 2.58 (s, 3H), 1.78 (quint, *J* = 7.2 Hz, 2H), 1.49–1.21 (m, 8H), 0.90 (app t, *J* = 7.0 Hz, 3H). ^13^C NMR (101 MHz, CDCl_3_) *δ*_ppm_ 170.6, 163.5, 161.7, 154.4, 152.6, 148.4, 120.9, 116.0 (sym, 2C), 114.8 (sym, 2C), 70.3, 68.5, 55.8, 31.9, 29.1, 28.7, 26.0, 22.7, 14.2, 10.7. MS-APCI (*m/z*): [M+H]^+^ 389.2.

#### Hexyl 6-(hexyloxy)-2-[(4-methoxyphenoxy)methyl]-5-methylpyrimidine-4-carboxylate (19)

To a solution of **14** (64 mg, 0.17 mmol) in 1-hexanol (1 mL), SOCl_2_ (37 μL, 0.51 mmol, 3 equiv) was added under argon atmosphere. The mixture was microwave irradiated for 1 h at 90 °C. The reaction was quenched by adding a saturated solution of NaHCO_3_ in water (10 mL) and the mixture was extracted with EtOAc (3×15 mL). The combined organic layers were washed with brine (3×15 mL) and the solvent was evaporated under reduced pressure at 40 °C. Residual 1-hexanol was removed by vacuum distillation. The residue was purified by flash column chromatography [cyclohexane (A), EtOAc (B); gradient: 10% → 13% B×10 CV] to give **19** as a brown oil (26 mg, 0.056 mmol, 33% yield). *R*_f_ 0.7 (cyclohexane/EtOAc 5:1). ^1^H NMR (400 MHz, CDCl_3_) *δ*_ppm_ 67.00–6.86 (m, 2H), 6.86–6.72 (m, 2H), 5.13 (s, 2H), 4.37 (t, *J* = 6.9 Hz, 2H), 4.35 (t, *J* = 6.7 Hz, 2H), 3.75 (s, 3H), 2.26 (s, 3H), 1.77 (quint, *J* = 7.2 Hz, 2H), 1.71 (quint, *J* = 7.1 Hz, 2H), 1.48–1.36 (m, 4H), 1.36–1.24 (m, 8H), 0.89 (app t, *J* = 7.2 Hz, 6H). ^13^C NMR (101 MHz, CDCl_3_) *δ*_ppm_ 169.3, 165.9, 163.1, 155.1, 154.1, 153.0, 116.6, 116.1 (sym, 2C), 114.6 (sym, 2C), 71.2, 67.6, 66.4, 55.8, 31.6, 31.5, 28.7, 28.6, 25.73, 25.68, 22.7, 22.6, 14.1 (2C), 11.1.

#### 2-propylpentyl 2-[(4-methoxyphenoxy)methyl]-5-methyl-6-[(2-propylpentyl)oxy]pyrimidine-4-carboxylate (20)

To a solution of **15** (69 mg, 0.17 mmol) in 1-hexanol (1 mL), SOCl_2_ (37 μL, 0.51 mmol, 3 equiv) was added under argon atmosphere. The mixture was microwave irradiated for 1 h at 90 °C. The reaction was quenched by adding a saturated solution of NaHCO_3_ in water (10 mL) and the mixture was extracted with EtOAc (3×15 mL). The combined organic layers were washed with brine (3×15 mL) and the solvent was evaporated under reduced pressure at 40 °C. Residual 1-hexanol was removed by vacuum distillation. The residue was purified by flash column chromatography [cyclohexane (A), EtOAc (B); gradient: 10% → 13% B×10 CV] to give **19** as a brown oil (44 mg, 0.085 mmol, 50% yield). *R*_f_ 0.75 (cyclohexane/EtOAc 5:1). ^1^H NMR (400 MHz, CDCl_3_) *δ*_ppm_ 6.98–6.86 (m, 2H), 6.86–6.74 (m, 2H), 5.13 (s, 2H), 4.29 (d, *J* = 5.8 Hz, 2H), 4.25 (d, *J* = 5.6 Hz, 2H), 3.75 (s, 3H), 2.26 (s, 3H), 1.93–1.67 (m, 2H), 1.44–1.24 (m, 16H), 0.98–0.81 (m, 12H). ^13^C NMR (101 MHz, CDCl_3_) *δ*_ppm_^13^C NMR (101 MHz, CDCl_3_) δ 169.4, 166.1, 163.1, 155.3, 154.1, 153.0, 116.3, 116.2 (sym, 2C), 114.6 (sym, 2C), 71.3, 70.2, 69.0, 55.8, 37.1, 37.0, 33.9 (sym, 2C), 33.6 (sym, 2C), 20.1 (sym, 2C), 20.0 (sym, 2C), 14.54 (sym, 2C), 14.50 (sym, 2C), 11.1. MS-APCI (*m/z*): [M+H]^+^ 515.2.

#### General procedure IV: CDI-promoted esterification of carboxylic acids

To a solution of a carboxylic acid in dry DMF (0.2–1.6 mL), CDI (2–2.2 equiv) was added and the mixture was stirred at rt for 1 h under argon atmosphere. An alcohol (1.1–2 equiv), DBU (0.5–2 equiv) and DMAP (0.1 equiv) were subsequently added and the reaction mixture was microwave irradiated at 50 °C for 1 h. The reaction was quenched with ice water and extracted with EtOAc. The combined organic layers were washed with a saturated solution of NaHCO_3_ in water, brine and the solvent was evaporated under reduced pressure at 40 °C. The crude residue was purified by flash column chromatography with appropriate eluents and a gradient.

#### 3-(trifluoromethyl)benzyl 2-[(4-methoxyphenoxy)methyl]-5-methyl-6-[[3-(trifluoromethyl)benzyl]oxy]pyrimidine-4-carboxylate (21)

General procedure IV was followed. Compound **16** (0.060 g, 0.13 mmol), dry DMF (0.2 mL), CDI (43 mg, 0.27 mmol, 2 equiv), 3-(trifluoromethyl)benzyl alcohol (36 μL, 0.27 mmol, 2 equiv), DBU (0.010 mL, 0.067 mmol, 0.5 equiv), DMAP (1.6 mg, 0.013 mmol, 0.1 equiv). Flash chromatography eluents: cyclohexane (A), EtOAc (B); gradient: 6% → 45% B×10 CV. Compound **21** was isolated as a yellow oil (49 mg, 0.081 mmol, 62% yield). *R*_f_ 0.5 (cyclohexane/EtOAc 4:1). ^1^H NMR (400 MHz, CDCl_3_) *δ*_ppm_ 7.72 (s, 1H), 7.68 (s, 1H), 7.67–7.48 (m, 4H), 7.51 (t, *J* = 7.7 Hz, 1H), 7.45 (t, *J* = 7.7 Hz, 1H), 7.00–6.85 (m, 2H), 6.85–6.70 (m, 2H), 5.47 (s, 2H), 5.46 (s, 2H), 5.17 (s, 2H), 3.75 (s, 3H), 2.31 (s, 3H). ^13^C NMR (101 MHz, CDCl_3_) *δ*_ppm_ 168.7, 165.1, 163.3, 154.7, 154.2, 152.8, 137.0, 136.3, 131.7 (2C), 129.4, 129.2, 125.6–125.3 (m, 2C), 125.3–125.0 (m, 2C), 117.5, 116.0 (sym, 2C), 114.7 (sym, 2C), 70.9, 68.1, 66.8, 55.8, 11.1 (the quartets of C-CF_3_ quaternary carbons with *J* ≈ 32 Hz and *J* ≈ 273 Hz could not be identified due to a low signal/noise ratio).

#### Methyl 2-[(4-methoxyphenoxy)methyl)]-5-methyl-6-[[3-(trifluoromethyl)benzyl]oxy]pyrimidine-4-carboxylate (22)

A 2-molar solution of (trimethylsilyl)diazomethane in Et_2_O (134 μL, 0.268 mmol, 2 equiv) was added to a solution of **16** (60.0 mg, 0.134 mmol) in dry CH_2_Cl_2_/MeOH (200 μL, 1:1) at 0 °C under argon atmosphere. The mixture was stirred for 30 min letting the temperature to rise to rt. The reaction was quenched by adding water (10 mL) and the mixture was extracted with EtOAc (3×10 mL). The combined organic layers were washed with brine (2×10 mL) and the solvent was evaporated under reduced pressure at 40 °C. The residue was purified by flash column chromatography [cyclohexane (A), EtOAc (B); gradient: 8% → 30% B×14 CV] to give **22** as a pale yellow oil (27.0 mg, 0.0584 mmol, 43.6% yield). *R*_f_ 0.3 (cyclohexane/EtOAc 4:1). ^1^H NMR (400 MHz, CDCl_3_) *δ*_ppm_ 7.69 (s, 1H), 7.64–7.51 (m, 2H), 7.45 (t, *J* = 7.7 Hz, 1H), 6.97–6.86 (m, 2H), 6.85–6.75 (m, 2H), 5.47 (s, 2H), 5.17 (s, 2H), 3.98 (s, 3H), 3.76 (s, 3H), 2.36 (s, 3H). ^13^C NMR (101 MHz, CDCl_3_) *δ*_ppm_ 168.8, 165.7, 163.1, 154.7, 154.2, 152.8, 137.1, 131.7 (app q, *J* = 1.2 Hz), 131.1 (d, *J* = 32.4 Hz), 129.2, 126.8 (d, *J* = 271.1 Hz), 125.2 (q, *J* = 3.9 Hz), 125.1 (q, *J* = 3.9 Hz), 117.7, 116.0 (sym, 2C), 114.7 (sym, 2C), 71.0, 68.1, 55.8, 53.2, 11.2 (the quartets of C-CF_3_ quaternary carbons with *J* ≈ 32.4 Hz and *J* ≈ 271.1 Hz are indicated as doublets because the lower intensity peaks could not be identified due to a low signal/noise ratio).

#### Heptan-3-yl 2-[(4-methoxyphenoxy)methyl]-5-methyl-6-[[3-(trifluoromethyl)benzyl]oxy]pyrimidine-4-carboxylate (23)

General procedure IV was followed. Compound **16** (0.060 g, 0.13 mmol), dry DMF (0.3 mL), CDI (43 mg, 0.27 mmol, 2 equiv), 3-heptanol (38 μL, 0.27 mmol, 2 equiv), DBU (0.010 mL, 0.067 mmol, 0.5 equiv), DMAP (1.6 mg, 0.013 mmol, 0.1 equiv). Flash chromatography eluents: cyclohexane (A), EtOAc (B); gradient: 5% → 40% B×10 CV. Compound **23** was isolated as a yellow oil (37 mg, 0.068 mmol, 52% yield). *R*_f_ 0.85 (cyclohexane/EtOAc 4:1). ^1^H NMR (400 MHz, CDCl_3_) *δ*_ppm_ 7.69 (s, 1H), 7.61–7.52 (m, 2H), 7.45 (t, *J* = 7.7 Hz, 1H), 6.99–6.88 (m, 2H), 6.86–6.75 (m, 2H), 5.46 (s, 2H), 5.16 (s, 2H), 5.13 (app quint, *J* = 6.5 Hz, 1H), 3.76 (s, 3H), 2.30 (s, 3H), 1.81–1.59 (m, 4H), 1.45–1.23 (m, 4H), 0.97 (t, *J* = 7.4 Hz, 3H), 0.91 (app t, *J* = 7.1 Hz, 3H). ^13^C NMR (101 MHz, CDCl_3_) *δ*_ppm_ 168.6, 165.6, 163.2, 156.6, 154.1, 152.9, 137.2, 131.7 (app q, *J* = 1.3, 0.9 Hz), 129.1, 125.2–125.0 (m, 2C), 116.1 (sym, 2C), 115.8, 114.6 (sym, 2C), 78.3, 71.0, 67.9, 55.8, 33.3, 27.6, 27.0, 22.7, 14.1, 11.1, 9.8 (the quartets of C-CF_3_ quaternary carbons with *J* ≈ 32 Hz and *J* ≈ 273 Hz respectively could not be identified due to low signal/noise ratio).

#### Heptan-3-yl 6-(heptyloxy)-2-[(4-methoxyphenoxy)methyl]-5-methylpyrimidine-4-carboxylate (24)

General procedure IV was followed. Compound **17** (70.0 mg, 0.180 mmol), dry DMF (1.6 mL), CDI (64 mg, 0.40 mmol, 2.2 equiv), 3-heptanol (28 μL, 0.20 mmol, 1.1 equiv), DBU (54 μL, 0.36 mmol, 2.2 equiv), DMAP (2.2 mg, 0.018 mmol, 0.1 equiv). Flash chromatography eluents: *n*-hexane (A), EtOAc (B); gradient: 3% → 28% B×10 CV. Compound **24** was isolated as a pale yellow oil (65.0 mg, 0.134 mmol, 74.4% yield). *R*_f_ 0.75 (*n*-hexane/EtOAc 5:1). ^1^H NMR (400 MHz, CDCl_3_) *δ*_ppm_ 6.98–6.89 (m, 2H), 6.84–6.74 (m, 2H), 5.13 (s, 2H), 5.12 (app quint, *J* = 6.1 Hz, 1H), 4.34 (t, *J* = 6.6 Hz, 2H), 3.75 (s, 3H), 2.24 (s, 3H), 1.79–1.59 (m, 6H), 1.45–1.20 (m, 12H), 0.97 (t, *J* = 7.4 Hz, 3H), 0.90 (app t, *J* = 6.6 Hz, 3H), 0.89 (app t, *J* = 6.7 Hz, 3H). ^13^C NMR (101 MHz, CDCl_3_) *δ*_ppm_ 169.2, 165.9, 163.1, 156.0, 154.1, 153.0, 116.2 (sym, 2C), 115.7, 114.6 (sym, 2C), 78.1, 71.2, 67.6, 55.8, 33.3, 31.9, 29.1, 28.8, 27.6, 27.0, 26.0, 22.74, 22.72, 14.2, 14.1, 11.1, 9.8. MS-APCI (*m/z*): [M+H]^+^ 487.2.

#### 3-(trifluoromethyl)benzyl 6-(heptyloxy)-2-[(4-methoxyphenoxy)methyl]-5-methylpyrimidine-4-carboxylate (25)

General procedure IV was followed. Compound **17** (0.070 g, 0.18 mmol), dry DMF (1.6 mL), CDI (64 mg, 0.40 mmol, 2.2 equiv), 3-(trifluoromethyl)benzyl alcohol (27 μL, 0.20 mmol, 1.1 equiv), DBU (54 μL, 0.36 mmol, 2.2 equiv), DMAP (2.2 mg, 0.018 mmol, 0.1 equiv). Flash chromatography eluents: *n*-hexane (A), EtOAc (B); gradient: 4% → 34% B×10 CV. Compound **25** was isolated as a yellow oil (0.060 g, 0.11 mmol, 61% yield). *R*_f_ 0.75 (*n*-hexane/EtOAc 5:1). ^1^H NMR (400 MHz, CDCl_3_) *δ*_ppm_ 7.72 (s, 1H), 7.65–7.58 (m, 2H), 7.50 (t, *J* = 7.7 Hz, 1H), 6.96–6.89 (m, 2H), 6.83–6.75 (m, 2H), 5.46 (s, 2H), 5.14 (s, 2H), 4.36 (t, *J* = 6.6 Hz, 2H), 3.75 (s, 3H), 2.26 (s, 3H), 1.72 (quint, *J* = 6.7 Hz, 2H), 1.45–1.16 (m, 8H), 0.89 (app t, *J* = 7.0 Hz, 3H). ^13^C NMR (101 MHz, CDCl_3_) *δ*_ppm_ 169.4, 165.3, 163.2, 154.2, 154.1, 152.9, 136.4, 131.7 (app q, *J* = 1.5 Hz), 131.2 (q, *J* = 32.6 Hz), 129.3, 125.4 (q, *J* = 3.6 Hz), 125.1 (q, *J* = 3.7 Hz), 124.3 (q, *J* = 272.5 Hz), 117.4, 116.1 (sym, 2C), 114.6 (sym, 2C), 71.2, 67.8, 66.7, 55.8, 31.9, 29.1, 28.7, 26.0, 22.7, 14.2, 11.1. MS-APCI (*m/z*): [M+H]^+^ 547.1.

#### Methyl 6-(heptan-3-yloxy)-2-[(4-methoxyphenoxy)methyl]-5-methylpyrimidine-4-carboxylate (26)

A 2 M solution of (trimethylsilyl)diazomethane in Et_2_O (128 μL, 0.257 mmol, 2 equiv) was added to a solution of **18** in dry CH_2_Cl_2_/MeOH (200 μL, 1:1) at 0 °C under argon atmosphere. The mixture was stirred for 30 min letting the temperature to rise to rt and the solvent was evaporated under reduced pressure at 40 °C. The residue was used without further purification. ^1^H NMR (400 MHz, CDCl_3_) *δ*_ppm_ 6.93–6.84 (m, 2H), 6.82–6.73 (m, 2H), 5.19 (quint, *J* = 6.0 Hz, 1H), 5.12 (s, 2H), 3.96 (s, 3H), 3.73 (s, 3H), 2.28 (s, 3H), 1.76–1.48 (m, 4H), 1.34–1.12 (m, 4H), 0.92–0.77 (m, 6H). ^13^C NMR (101 MHz, CDCl_3_) *δ*_ppm_ 169.4, 166.1, 163.0, 154.3, 154.0, 152.9, 117.4, 115.9 (sym, 2C), 114.6 (sym, 2C), 78.4, 70.9, 55.8, 53.0, 32.9, 27.4, 26.6, 22.7, 14.1, 11.1, 9.5.

#### 3-(trifluoromethyl)benzyl 6-(heptan-3-yloxy)-2-[(4-methoxyphenoxy)methyl]-5-methylpyrimidine-4-carboxylate (27)

General procedure IV was followed. Compound **18** (50.0 mg, 0.128 mmol), dry DMF (0.2 mL), CDI (42 mg, 0.26 mmol, 2 equiv), 3-(trifluoromethyl)benzyl alcohol (35 μL, 0.26 mmol, 2 equiv), DBU (0.010 mL, 0.064 mmol, 0.5 equiv), DMAP (1.6 mg, 0.013 mmol, 0.1 equiv). Flash chromatography eluents: cyclohexane (A), EtOAc (B); gradient: 4% → 21% B×6 CV. Compound **27** was isolated as a transparent oil (45 mg, 0.082 mmol, 64% yield). *R*_f_ 0.45 (*n*-hexane/EtOAc 5:1). ^1^H NMR (400 MHz, CDCl_3_) *δ*_ppm_ 7.72 (s, 1H), 7.64 (d, *J* = 7.6 Hz, 1H), 7.61 (d, *J* = 7.9 Hz, 1H), 7.51 (t, *J* = 7.7 Hz, 1H), 6.97–6.84 (m, 2H), 6.84–6.71 (m, 2H), 5.46 (s, 2H), 5.21 (quint, *J* = 5.9 Hz, 1H), 5.14 (s, 2H), 3.74 (s, 3H), 2.23 (s, 3H), 1.76–1.48 (m, 4H), 1.33–1.11 (m, 4H), 0.92–0.74 (m, 6H). ^13^C NMR (101 MHz, CDCl_3_) *δ*_ppm_ 169.4, 165.5, 163.2, 154.3, 154.1, 152.9, 136.4, 131.7 (app q, *J* = 0.9 Hz), 129.3, 125.4 (q, *J* = 3.8 Hz), 125.2 (q, *J* = 3.8 Hz), 117.2, 116.0 (sym, 2C), 114.6 (sym, 2C), 78.5, 70.9, 66.7, 55.8, 32.9, 27.4, 26.6, 22.7, 14.1, 11.1, 9.5 (the quartets of C-CF_3_ quaternary carbons with *J* ≈ 32 Hz and *J* ≈ 273 Hz could not be identified due to a low signal/noise ratio).

#### Hexyl 6-(hexyloxy)-2-(hydroxymethyl)-5-methylpyrimidine-4-carboxylate (2a)

General procedure II was followed. Compound **19** (26 mg, 0.055 mmol), CH_3_CN/H_2_O 4:1 (660 μL). Flash chromatography eluents: cyclohexane (A), EtOAc (B); gradient: 13% → 25% B×15 CV. Compound **2a** was isolated as an orange oil (11 mg, 0.031 mmol, 56% yield). *R*_f_ 0.3 (cyclohexane/EtOAc 6:1). ^1^H NMR (400 MHz, CDCl_3_) *δ*_ppm_ 4.69 (s, 2H), 4.40 (t, *J* = 5.9 Hz, 2H), 4.37 (t, *J* = 6.1 Hz, 2H), 3.40 (br s, 1H), 2.28 (s, 3H), 1.85–1.65 (m, 4H), 1.49–1.38 (m, 4H), 1.37–1.30 (m, 8H), 1.02–0.74 (m, 6H). ^13^C NMR (101 MHz, CDCl_3_) *δ*_ppm_ 169.2, 165.8, 165.3, 154.5, 116.3, 67.7, 66.4, 64.2, 31.6, 31.5, 28.7, 28.6, 25.8, 25.7, 22.69, 22.65, 14.13, 14.11, 11.1. HRMS-ESI (*m/z*): [M+H]^+^ calcd for C_19_H_33_N_2_O_4_ 353.2440; found 353.2445.

#### Heptyl 6-(heptyloxy)-2-(hydroxymethyl)-5-methylpyrimidine-4-carboxylate (2b)

General procedure II was followed. Compound **11** (0.040 g, 0.082 mmol), CH_3_CN/H_2_O 4:1 (1.2 mL). Flash chromatography eluents: cyclohexane (A), EtOAc (B); gradient: 15% → 18% B×10 CV. Compound **2b** was isolated as an orange oil (21 mg, 0.055 mmol, 67% yield). *R*_f_ 0.67 (cyclohexane/EtOAc 4:1). ^1^H NMR (400 MHz, CDCl_3_) *δ*_ppm_ 4.69 (s, 2H), 4.39 (t, *J* = 6.1 Hz, 2H), 4.36 (t, *J* = 6.3 Hz, 2H), 3.55 (br s, 1H), 2.27 (s, 3H), 1.91–1.65 (m, 4H), 1.52–1.36 (m, 4H), 1.38–1.17 (m, 12H), 0.89 (app t, *J* = 6.7 Hz, 3H), 0.88 (app t, *J* = 6.7 Hz, 3H). ^13^C NMR (101 MHz, CDCl_3_) *δ*_ppm_ 169.2, 165.7, 165.3, 154.5, 116.3, 67.7, 66.4, 64.2, 31.9, 31.8, 29.1, 29.0, 28.8, 28.7, 26.1, 26.0, 22.72, 22.69, 14.19, 14.18, 11.0. HRMS-ESI (*m/z*): [M+H]^+^ calcd for C_21_H_37_N_2_O_4_ 381.2753; found 381.2751.

#### Octyl 2-(hydroxymethyl)-5-methyl-6-(octyloxy)pyrimidine-4-carboxylate (2c)

General procedure II was followed. Compound **12** (29 mg, 0.056 mmol), CH_3_CN/H_2_O 4:1 (0.83 mL). Flash chromatography eluents: cyclohexane (A), EtOAc (B); gradient: 20% → 100% B×15 CV. Compound **2c** was isolated as a brown oil (14 mg, 0.035 mmol, 62% yield). *R*_f_ 0.8 (cyclohexane/EtOAc 4:1). ^1^H NMR (400 MHz, CDCl_3_) *δ*_ppm_ 4.69 (s, 2H), 4.39 (t, *J* = 6.3 Hz, 2H), 4.36 (t, *J* = 6.4 Hz, 2H), 3.22 (br s, 1H), 2.27 (s, 3H), 1.85–1.70 (m, 4H), 1.50–1.36 (m, 4H), 1.38–1.19 (m, 16H), 0.88 (app t, *J* = 6.7 Hz, 3H), 0.87 (app t, *J* = 7.1 Hz, 3H). ^13^C NMR (101 MHz, CDCl_3_) *δ*_ppm_ 169.2, 165.7, 165.3, 154.5, 116.3, 67.8, 66.4, 64.2, 31.91, 31.88, 29.4, 29.33, 29.30, 29.28, 28.74, 28.66, 26.1, 26.0, 22.78, 22.76, 14.22, 14.21, 11.1. HRMS-ESI (*m/z*): [M+H]^+^ calcd for C_23_H_41_N_2_O_4_ 409.3066; found 409.3067.

#### 2-propylpentyl 2-(hydroxymethyl)-5-methyl-6-[(2-propylpentyl)oxy]pyrimidine-4-carboxylate (2d)

General procedure II was followed. Compound **20** (44 mg, 0.085 mmol), CH_3_CN/H_2_O 4:1 (1 mL). Flash chromatography eluents: cyclohexane (A), EtOAc (B); gradient: 14% → 20% B×10 CV. Compound **2d** was isolated as an orange oil (23 mg, 0.055 mmol, 65% yield). *R*_f_ 0.3 (cyclohexane/EtOAc 6:1). ^1^H NMR (400 MHz, CDCl_3_) *δ*_ppm_ 4.68 (s, 2H), 4.29 (d, *J* = 4.4 Hz, 2H), 4.28 (d, *J* = 4.7 Hz, 2H), 3.59 (br s, 1H), 2.28 (s, 3H), 1.93–1.70 (m, 2H), 1.46–1.28 (m, 16H), 1.01–0.77 (m, 12H). ^13^C NMR (101 MHz, CDCl_3_) *δ*_ppm_ 169.3, 165.9, 165.3, 154.5, 116.2, 70.4, 69.0, 64.1, 37.1, 37.0, 33.9 (sym, 2C), 33.7 (sym, 2C), 20.1 (sym, 2C), 20.0 (sym, 2C), 14.51 (sym, 2C), 14.48 (sym, 2C), 11.1. HRMS-ESI (*m/z*): [M+H]^+^ calcd for C_23_H_41_N_2_O_4_ 409.3066; found 409.3068.

#### Heptan-3-yl 6-(heptan-3-yloxy)-2-(hydroxymethyl)-5-methylpyrimidine-4-carboxylate (2e)

General procedure II was followed. Compound **13** (22 mg, 0.046 mmol), CH_3_CN/H_2_O 4:1 (0.54 mL). Flash chromatography eluents: cyclohexane (A), EtOAc (B); gradient: 6% → 20% B×10 CV. Compound **2e** was isolated as an orange oil (12 mg, 0.032 mmol, 69% yield). *R*_f_ 0.7 (cyclohexane/EtOAc 3:1). ^1^H NMR (400 MHz, CDCl_3_) *δ*_ppm_ 5.27 (quint, *J* = 5.9 Hz, 1H), 5.13 (quint, *J* = 6.1 Hz, 1H), 4.66 (s, 2H), 3.62 (br s, 1H), 2.24 (s, 3H), 1.84–1.52 (m, 8H), 1.47–1.18 (m, 8H), 0.98 (t, *J* = 7.4 Hz, 3H), 0.91 (t, *J* = 7.4 Hz, 3H), 0.91 (app t, *J* = 7.0 Hz, 3H), 0.89 (app t, *J* = 6.9 Hz, 3H). ^13^C NMR (101 MHz, CDCl_3_) *δ*_ppm_ 169.1, 165.9, 165.1, 155.4, 115.5, 78.4, 78.1, 64.1, 33.4, 33.1, 27.7, 27.5, 27.1, 26.7, 22.8, 22.7, 14.1 (2C), 11.1, 9.8, 9.6. HRMS-ESI (*m/z*): [M+H]^+^ calcd for C_21_H_37_N_2_O_4_ 381.2753; found 381.2753.

#### 3-(trifluoromethyl)benzyl 2-(hydroxymethyl)-5-methyl-6-[[3-(trifluoromethyl)benzyl]oxy]pyrimidine-4-carboxylate (2f)

General procedure II was followed. Compound **21** (48 mg, 0.079 mmol), CH_3_CN/H_2_O 4:1 (0.92 mL). Flash chromatography eluents: cyclohexane (A), EtOAc (B); gradient: 12% → 100% B×10 CV. Compound **2f** was isolated as a yellow oil (6.7 mg, 0.013 mmol, 17% yield). *R*_f_ 0.18 (cyclohexane/EtOAc 4:1). ^1^H NMR (400 MHz, CDCl_3_) *δ*_ppm_ 7.71 (s, 2H), 7.68–7.58 (m, 4H), 7.52 (t, *J* = 7.7 Hz, 2H), 5.52 (s, 2H), 5.46 (s, 2H), 4.73 (s, 2H), 3.42 (br s, 1H), 2.33 (s, 3H). ^13^C NMR (101 MHz, CDCl_3_) *δ*_ppm_ 168.68, 165.49, 164.96, 154.06, 136.87, 136.23, 131.69 (app q, *J* = 1.1 Hz), 131.36 (app q, *J* = 1.0 Hz), 131.31 (d, *J* = 32.7 Hz), 131.25 (d, *J* = 32.4 Hz), 129.41, 129.34, 125.56 (q, *J* = 3.8 Hz), 125.37 (q, *J* = 3.9 Hz), 125.16 (q, *J* = 3.5 Hz), 124.92 (q, *J* = 3.7 Hz), 117.19, 68.29, 66.84, 64.24, 11.11 (the quartets with *J* ≈ 32 Hz of the quaternary carbons bearing a -CF_3_ group are indicated as doublets because the lower intensity peaks could not be identified due to low signal/noise ratio; the quartets with *J* ≈ 273 Hz of the -CF_3_ quaternary carbons could not be identified due to low signal/noise ratio). ^19^F NMR (376 MHz, CDCl_3_) *δ*_ppm_ -62.73, -62.74. HRMS-ESI (*m/z*): [M+H]^+^ calcd for C_23_H_19_N_2_O_4_F_6_ 501.1249; found 501.1250.

#### Methyl 2-(hydroxymethyl)-5-methyl-6-[[3-(trifluoromethyl)benzyl]oxy]pyrimidine-4-carboxylate (2g)

General procedure II was followed. Compound **22** (27 mg, 0.058 mmol), CH_3_CN/H_2_O 4:1 (0.68 mL). Flash chromatography eluents: cyclohexane (A), EtOAc (B); gradient: 8% → 60% B×15 CV. Compound **2g** was isolated as a yellow oil (11 mg, 0.029 mmol, 50% yield). *R*_f_ 0.15 (cyclohexane/EtOAc 2:1). ^1^H NMR (400 MHz, CDCl_3_) *δ*_ppm_ 7.71 (s, 1H), 7.67–7.58 (m, 2H), 7.52 (t, *J* = 7.7 Hz, 1H), 5.53 (s, 2H), 4.74 (app d, *J* = 0.5 Hz, 2H), 3.98 (s, 3H), 3.06 (s, 1H), 2.37 (s, 3H). ^13^C NMR (101 MHz, CDCl_3_) *δ*_ppm_ 168.7, 165.7, 165.4, 154.3, 136.9, 131.3 (app q, *J* = 1.0 Hz), 131.3 (q, *J* = 32.5 Hz), 129.3, 125.3 (q, *J* = 3.9 Hz), 124.9 (q, *J* = 3.8 Hz), 124.1 (q, *J* = 272.4 Hz), 117.2, 68.3, 64.3, 53.1, 11.1. HRMS-ESI (*m/z*): [M+H]^+^ calcd for C_16_H_16_N_2_O_4_F_3_ 357.1062; found 357.1063.

#### Heptan-3-yl 2-(hydroxymethyl)-5-methyl-6-[[3-(trifluoromethyl)benzyl]oxy]pyrimidine-4-carboxylate (2h)

General procedure II was followed. Compound **23** (25 mg, 0.046 mmol), CH_3_CN/H_2_O 4:1 (0.54 mL). Flash chromatography eluents: cyclohexane (A), EtOAc (B); gradient: 8% → 30% B×12 CV. Compound **2h** was isolated as a yellow oil (11 mg, 0.025 mmol, 54% yield). *R*_f_ 0.25 (cyclohexane/EtOAc 4:1). ^1^H NMR (400 MHz, CDCl_3_) *δ*_ppm_ 7.71 (s, 1H), 7.66–7.58 (m, 2H), 7.52 (t, *J* = 7.7 Hz, 1H), 5.52 (s, 2H), 5.13 (quint, *J* = 6.1 Hz, 1H), 4.71 (s, 2H), 3.30 (br s, 1H), 2.32 (s, 3H), 1.81–1.59 (m, 4H), 1.44–1.27 (m, 4H), 0.98 (t, *J* = 7.4 Hz, 3H), 0.91 (app t, *J* = 7.0 Hz, 3H). ^13^C NMR (101 MHz, CDCl_3_) *δ*_ppm_ 168.5, 165.4, 165.3, 155.7, 137.0, 131.3 (app q, *J* = 1.3 Hz), 131.2 (q, *J* = 32.6 Hz), 129.3, 125.3 (q, *J* = 3.8 Hz), 124.9 (q, *J* = 3.8 Hz), 124.1 (q, *J* = 272.4 Hz), 115.7, 78.3, 68.1, 64.1, 33.4, 27.6, 27.1, 22.7, 14.1, 11.1, 9.8. HRMS-ESI (*m/z*): [M+H]^+^ calcd for C_22_H_28_N_2_O_4_F_3_ 441.2001; found 441.2003.

#### Heptan-3-yl 6-(heptyloxy)-2-(hydroxymethyl)-5-methylpyrimidine-4-carboxylate (2i)

General procedure II was followed. Compound **24** (50.7 mg, 0.104 mmol), CH_3_CN/H_2_O 4:1 (1.25 mL). Flash chromatography eluents: *n*-hexane (A), EtOAc (B); gradient: 3% → 28% B×10 CV. Compound **2i** was isolated as a yellow oil (30.3 mg, 0.0796 mmol, 76.5% yield). *R*_f_ 0.4 (*n*-hexane/EtOAc 6:1). ^1^H NMR (400 MHz, CDCl_3_) *δ*_ppm_ 5.12 (quint, *J* = 6.1 Hz, 1H), 4.67 (s, 2H), 4.39 (t, *J* = 6.6 Hz, 2H), 3.62 (br s, 1H), 2.26 (s, 3H), 1.84–1.60 (m, 6H), 1.50–1.21 (m, 12H), 0.98 (t, *J* = 7.4 Hz, 3H), 0.90 (app t, *J* = 7.0 Hz, 3H), 0.89 (app t, *J* = 6.9 Hz, 3H). ^13^C NMR (101 MHz, CDCl_3_) *δ*_ppm_ 169.1, 165.7, 165.3, 155.2, 115.5, 78.1, 67.7, 64.1, 33.4, 31.9, 29.1, 28.8, 27.6, 27.1, 26.1, 22.73, 22.69, 14.2, 14.1, 11.0, 9.8. MS-APCI (*m/z*): [M+H]^+^ 381.3. HRMS-ESI (*m/z*): [M+H]^+^ calcd for C_21_H_37_N_2_O_4_ 381.2753; found 381.2756.

#### 3-(trifluoromethyl)benzyl 6-(heptyloxy)-2-(hydroxymethyl)-5-methylpyrimidine-4-carboxylate (2j)

General procedure II was followed. Compound **25** (46 mg, 0.085 mmol), CH_3_CN/H_2_O 4:1 (1.25 mL). Flash chromatography eluents: *n*-hexane (A), EtOAc (B); gradient: 5% → 40% B×10 CV. Compound **2j** was isolated as a yellow oil (9.0 mg, 0.020 mmol, 24% yield). *R*_f_ 0.4 (*n*-hexane/EtOAc 4:1). ^1^H NMR (400 MHz, CDCl_3_) *δ*_ppm_ 7.71 (s, 1H), 7.64 (d, *J* = 7.6 Hz, 1H), 7.61 (d, *J* = 7.9 Hz, 1H), 7.52 (t, *J* = 7.7 Hz, 1H), 5.45 (s, 2H), 4.70 (s, 2H), 4.40 (t, *J* = 6.6 Hz, 2H), 3.50 (s, 1H), 2.27 (s, 3H), 1.79 (quint, *J* = 6.7 Hz, 2H), 1.49–1.22 (m, 8H), 0.89 (app t, *J* = 6.9 Hz, 3H). ^13^C NMR (101 MHz, CDCl_3_) *δ*_ppm_ 169.3, 165.4, 165.2, 153.5, 136.3, 131.7 (app q, *J* = 1.3 Hz), 131.3 (q, *J* = 32.8 Hz), 129.4, 125.5 (q, *J* = 3.8 Hz), 125.1 (q, *J* = 3.9 Hz), 124.0 (q, *J* = 272.2 Hz), 117.1, 67.9, 66.7, 64.2, 31.9, 29.1, 28.7, 26.1, 22.7, 14.2, 11.1. MS-APCI (*m/z*): [M+H]^+^ 441.2. HRMS-ESI (*m/z*): [M+H]^+^ calcd for C_22_H_28_N_2_O_4_F_3_ 441.2001; found 441.2007.

#### Methyl 6-(heptan-3-yloxy)-2-(hydroxymethyl)-5-methylpyrimidine-4-carboxylate (2k)

General procedure II was followed. Compound **26** (52 mg, 0.13 mmol), CH_3_CN/H_2_O 4:1 (1.5 mL). Flash chromatography eluents: cyclohexane (A), EtOAc (B); gradient: 12% → 100% B×10 CV. Compound **2k** was isolated as a brown oil (22 mg, 0.075 mmol, 58% yield). *R*_f_ 0.65 (cyclohexane/EtOAc 1:1). ^1^H NMR (400 MHz, CDCl_3_) *δ*_ppm_ 5.26 (quint, *J* = 5.8 Hz, 1H), 4.68 (s, 2H), 3.96 (s, 3H), 3.45 (br s, 1H), 2.29 (s, 3H), 1.76–1.60 (m, 4H), 1.39–1.20 (m, 4H), 0.91 (t, *J* = 7.4 Hz, 3H), 0.88 (app t, *J* = 7.1 Hz, 3H). ^13^C NMR (101 MHz, CDCl_3_) *δ*_ppm_ 169.3, 166.0, 165.1, 153.9, 117.1, 78.7, 64.3, 53.0, 33.0, 27.5, 26.7, 22.7, 14.1, 11.1, 9.6. HRMS-ESI (*m/z*): [M+H]^+^ calcd for C_15_H_25_N_2_O_4_ 297.1814; found 297.1814.

#### 3-(trifluoromethyl)benzyl 6-(heptan-3-yloxy)-2-(hydroxymethyl)-5-methylpyrimidine-4-carboxylate (2l)

General procedure II was followed. Compound **27** (44 mg, 0.080 mmol), CH_3_CN/H_2_O 4:1 (0.94 mL). Flash chromatography eluents: cyclohexane (A), EtOAc (B); gradient: 6% → 46% B×9 CV. Compound **2l** was isolated as an orange oil (0.010 g, 0.023 mmol, 29% yield). *R*_f_ 0.4 (cyclohexane/EtOAc 3:1). ^1^H NMR (400 MHz, CDCl_3_) *δ*_ppm_ 7.72 (s, 1H), 7.65 (d, *J* = 7.7 Hz, 1H), 7.61 (d, *J* = 7.8 Hz, 1H), 7.52 (t, *J* = 7.8 Hz, 1H), 5.46 (s, 2H), 5.27 (quint, *J* = 5.9 Hz, 1H), 4.68 (s, 2H), 3.02 (br s, 1H), 2.26 (s, 3H), 1.82–1.52 (m, 4H), 1.42–1.17 (m, 4H), 0.91 (t, *J* = 7.1 Hz, 3H), 0.88 (app t, *J* = 7.1 Hz, 3H). ^13^C NMR (101 MHz, CDCl_3_) *δ*_ppm_ 169.3, 165.3 (2C), 153.6, 136.3, 131.7 (app q, *J* = 1.1 Hz), 131.3 (q, *J* = 32.4 Hz), 129.4, 125.5 (q, *J* = 3.8 Hz), 125.2 (q, *J* = 3.8 Hz), 124.0 (q, *J* = 272.3 Hz), 117.1, 78.8, 66.7, 64.2, 33.0, 27.5, 26.7, 22.7, 14.1, 11.1, 9.6. HRMS-ESI (*m/z*): [M+H]^+^ calcd for C_22_H_8_N_2_O_4_ 441.2001; found 441.2003.

### ChemGPS-NP

All the structures included in the 3D-plot were converted into SMILES using ChemDraw Professional 16.0.0.82 and uploaded to the ChemGPS-NP_Web_ tool (http://chemgps.bmc.uu.se) [25]. The resulting coordinates were plotted using Grapher 2.5 distributed together with MacOS X. All the pyrimidines reported in this article were included. The following list comprises all the other compounds in alphabetical order and relevant/available *K*_i_ values for PKC*α* are indicated in parenthesis: 9-decyl-benzolactam-V8 (3.8 nM) [32]; bryostatin-1 (1.35 nM) [33], bryostatin-18 (4.8 nM) [34]; (*E*)-DAG-lactone 31 (2.7 nM) [16], (*Z*)-DAG-lactone 9 (11 nM) [35]; HMI-1a1 and -1a2, HMI-1a3 (205 nM), HMI-1b1–1b10, HMI-1b11 (319 nM), HMI-1b12–1b21, HMI-15e, -22c and -24a [12]; indolactam-V (11 nM) [36]; ingenol 3-angelate (0.1 nM) [37]; iripallidal (75.6 nM) [38]; mezerein (0.27 nM) [36]; phorbol 13-acetate (120 μM) [39], phorbol 12,13-dibutyrate (0.3 nM) [37], phorbol 12-myristate-13-acetate (2 nM) [39]; prostratin (4.83 nM) [36]. The full list of the compounds, ChemGPS-NP raw data, SMILES and structures are available in S1 File.

### Biological assay

Materials: [20-^3^H]Phorbol-12,13-dibutyrate ([^3^H]PDBu) (20 Ci/mmol) was acquired from American Radiolabeled Chemicals Inc. (Saint Louis, MO). Phorbol 12-myristate-13-acetate (PMA) and phosphatidyl-L-serine (PS; product number: P6641) and bovine immunoglobulin G (IgG) were purchased from Sigma-Aldrich (Steinheim, Germany). Protease inhibitors (Complete Protease Inhibitor Cocktail Tablets) were from Roche (Mannheim, Germany) And the Optiphase SuperMix liquid scintillant was from PerkinElmer (Groningen, Netherlands).

Method: PKC*α* protein was produced in recombinant baculovirus-infected Sf9 cells as described previously [40]. The cells were harvested two days after infection, washed with PBS, and the resultant cell pellets were frozen. Subsequently the cells were suspended in buffer containing 25 mM Tris-HCl (pH 7.5), 0.5 mM EGTA, 0.1% Triton X-100, and protease inhibitors to prepare a crude cell lysate. Following a 30-min incubation on ice, the lysate was centrifuged at 16000g for 15 min at 4 °C and the supernatant representing the soluble (cytosolic) fraction was collected. The protein content of the supernatant was determined with a Bradford assay.

The ability of the compounds to compete in binding to the regulatory domain of PKC*α* with radioactively labeled phorbol ester [^3^H]PDBu was determined according to Gopalakrishna et al. [26]. First, 20 μg of protein/well from the supernatant was incubated with the test compounds and [^3^H]PDBu for 10 min at room temperature in a 96-well Durapore filter plate (Millipore, cat. no. MSHVN4B50, Carrigtwohill, Ireland) in a total volume of 125 μL. The final concentrations in the assay were as follows: 20 mM Tris-HCl (pH 7.5), 40 μM CaCl_2_, 10 mM MgCl_2_, 400 μg/ mL bovine IgG, 25 nM [^3^H]PDBu, and 0.1 mg/mL phosphatidyl-L-serine (1,2-diacyl-*sn*-glycero-3-phospho-L-serine). Proteins were then precipitated by the addition of 125 μL of cold 20% poly(ethylene glycol) 6000, and after 15 min of incubation on a plate shaker at room temperature the filters were washed six times using a vacuum manifold with buffer containing 20 mM Tris-HCl (pH 7.5), 100 μM CaCl_2_, and 5 mM MgCl_2_. The plates were dried and 25 μL of Optiphase SuperMix liquid scintillant was added to each well. Radioactivity was measured using Wallac Microbeta Trilux microplate liquid scintillation counter (PerkinElmer, Waltham, MA, USA) after an equilibration period of three hours. All tested compounds were diluted in DMSO to give the same final DMSO concentration in the binding assay (4%) in each well. PMA (1 μM) was used as a positive control in all assays and as the nonspecific binding was around 6%, only the total binding was measured. The results were calculated as a percentage of control (4% DMSO) from the same plate. The graphs were created using GraphPad Prism version 5.02 for Windows (GraphPad Software, La Jolla, CA, www.graphpad.com).

## Supporting information

S1 File ChemGPS-NP raw data, SMILES and structures. (XLSX)

S2 File Displacement assays raw data. (XLSX)

S1 Appendix NMR spectra of all target compounds and representative intermediates. (PDF)
