# Supplementary material for: Scaffold hopping from (5-hydroxymethyl) isophthalates to multisubstituted pyrimidines diminishes binding affinity to the C1 domain of protein kinase C
Source: PLoS One. 2018 Apr 11;13(4):e0195668. doi: 10.1371/journal.pone.0195668 (PMC5895059; doi:10.1371/journal.pone.0195668)
Supplement: S1 Appendix — (PDF) [file pone.0195668.s003.pdf]

# – Supporting Information –

S1 Appendix

Pyrimidine analogs of C1 domain–targeted isophthalates as protein kinase C ligands: Structure-activity relationships and biological activities.

Riccardo Provenzani[1], Ilari Tarvainen[2], Giulia Brandoli[1], Antti Lempinen[1], Sanna Artes[1], Ainoleena Turku[1], Maria Helena Jäntti[2], Virpi Talman[2], Jari Yli-Kauhaluoma[1], Raimo Kalevi Tuominen[2], Gustav Boije af Gennäs[1]

[1]Drug Research Program, Division of Pharmaceutical Chemistry and Technology, Faculty of Pharmacy, University of Helsinki, Helsinki, Finland

[2]Drug Research Program, Division of Pharmacology and Pharmacotherapy, Faculty of Pharmacy, University of Helsinki, Helsinki, Finland

NMR Spectra

Peak reports and multiplet assignments are provided for all ^{1}H, ^{13}C and ^{19}F NMR spectra. Compound peaks are highlighted in **green** and solvent peaks in **yellow**. All 2D–NMR spectra are edited to highlight the correlation peaks. ^{13}C-HSQC spectra include DEPT-135 information with –CH– and $–CH_3$ correlation peaks in positive phase (**red**) while $–CH_2–$ correlation peaks in negative phase (**blue**). For some compounds also ^{15}N-HMBC NMR spectrum is available.

Table of contents

**Compounds**			**Page**
Compound	**1a**	....	S005
Compound	**1b**	....	S011
Compound	**1c**	....	S017
Compound	**1d**	....	S022
Compound	**1e**	....	S026
Compound	**1f**	....	S032
Compound	**1g**	....	S038
Compound	**1h**	....	S045
Compound	**2a**	....	S049
Compound	**2b**	....	S053

Compound **2c** S057
Compound **2d** S061
Compound **2e** S065
Compound **2f** S069
Compound **2g** S075
Compound **2h** S080
Compound **2i** S085
Compound **2j** S090
Compound **2k** S096
Compound **2l** S100
Compound **5** S105
Compound **6a** S111
Compound **6b** S117
Compound **6c** S123
Compound **6d** S129
Compound **6e** S133
Compound **6f** S136
Compound **6g** S142
Compound **9** S149

Compound **10** S153
Compound **11** S159
Compound **12** S163
Compound **13** S167
Compound **14** S171
Compound **15** S175
Compound **16** S179
Compound **17** S184
Compound **18** S188
Compound **19** S192
Compound **20** S196
Compound **21** S200
Compound **22** S204
Compound **23** S209
Compound **24** S213
Compound **25** S219
Compound **26** S226
Compound **27** S230

1H ^{1}H NMR (400 MHz, $CDCl_3$) δ 8.46 (app t, *J* = 0.8 Hz, 1H), 5.03 (app d, *J* = 0.8 Hz, 2H), 4.45 (t, *J* = 6.9 Hz, 4H), 3.16 (br s, 1H), 1.82 (quint, *J* = 6.8 Hz, 4H), 1.55 – 1.16 (m, 16H), 0.88 (app t, *J* = 6.8 Hz, 6H).

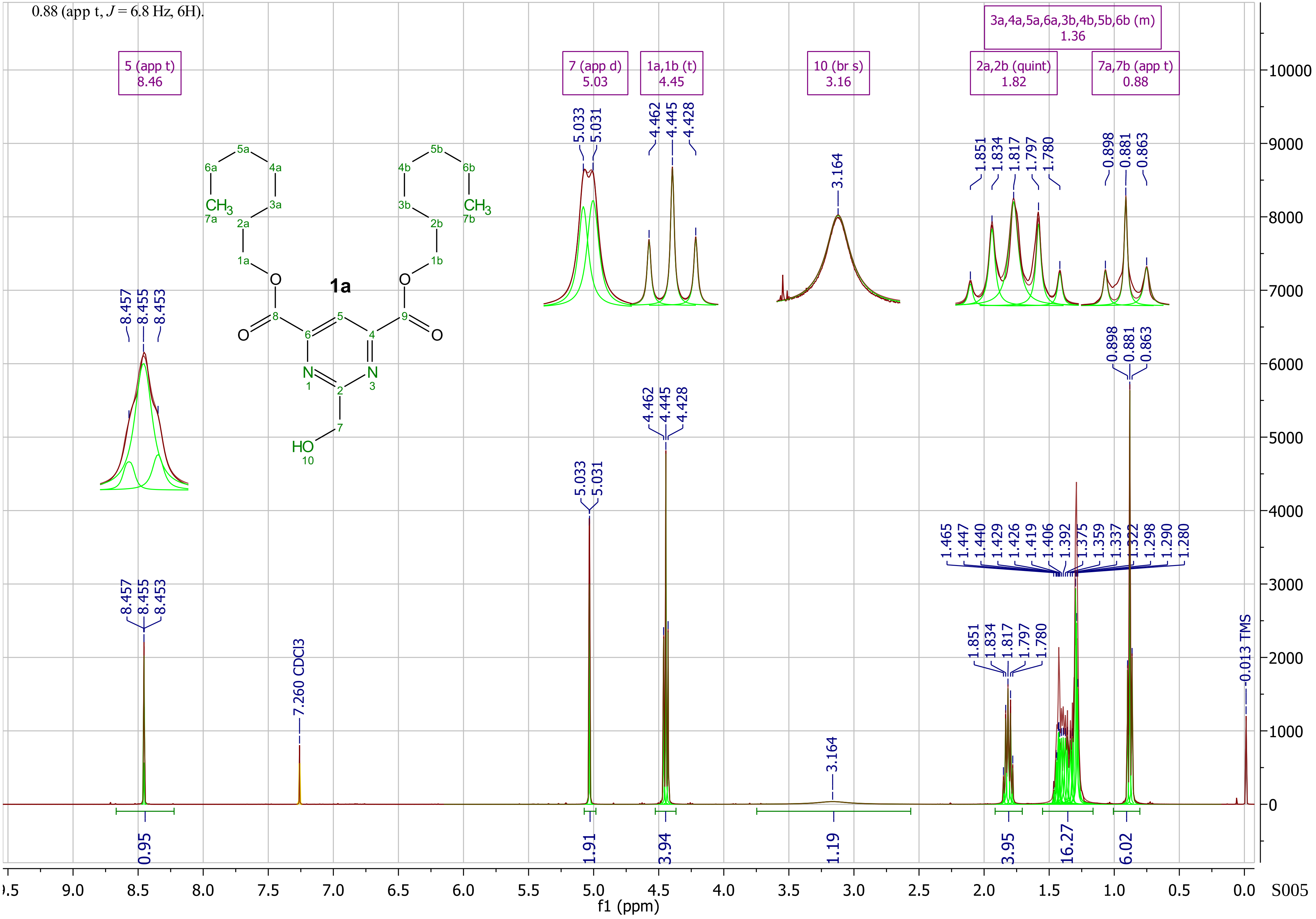

13C ^{13}C NMR (101 MHz, $CDCl_3$) δ 170.4, 163.4 (sym, 2C), 157.7 (sym, 2C), 118.7, 67.3 (sym, 2C), 64.9, 31.8 (sym, 2C), 29.0 (sym, 2C), 28.6 (sym, 2C), 25.9 (sym, 2C), 22.7 (sym, 2C), 14.2 (sym, 2C).

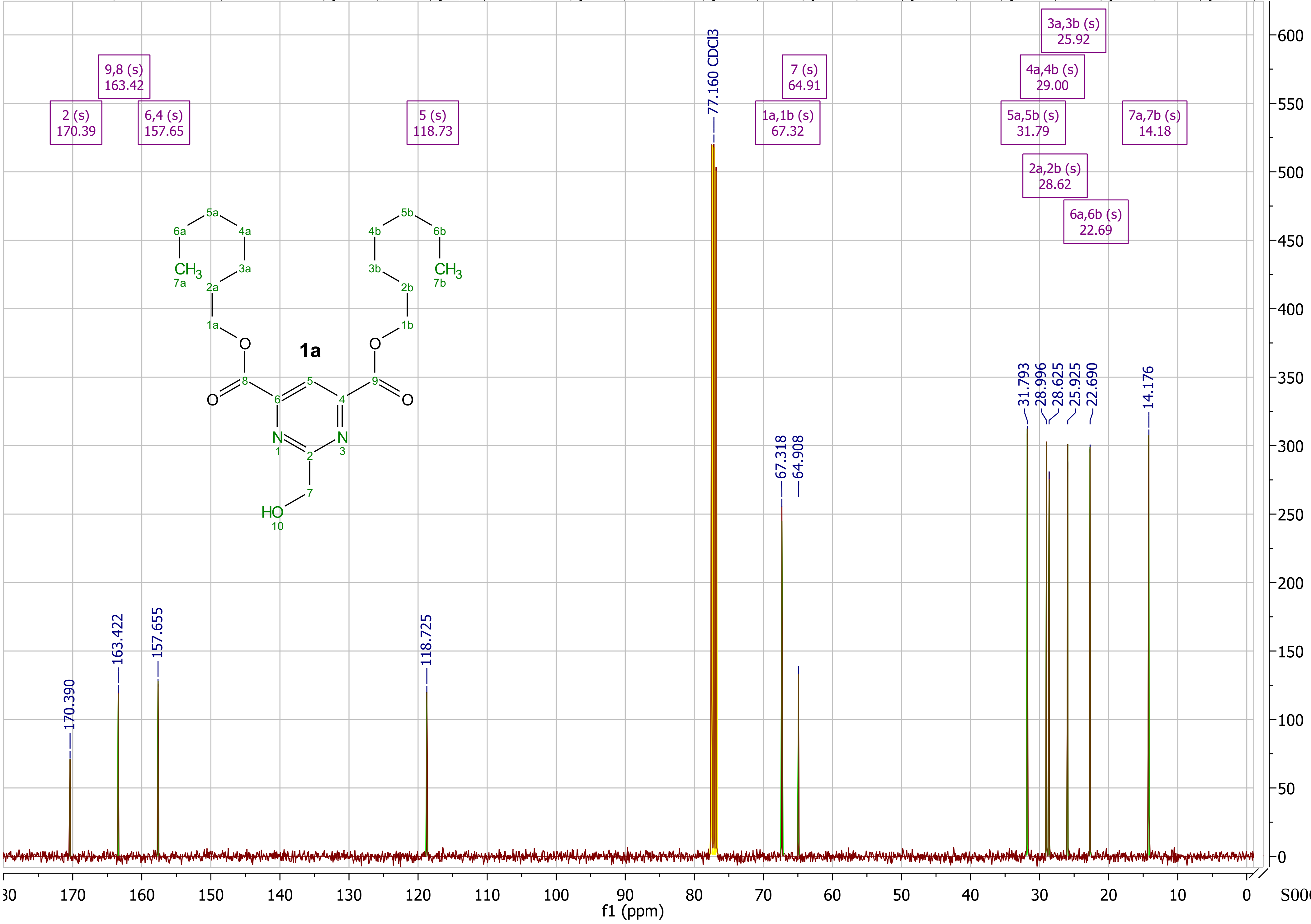

S006

13C HSQC

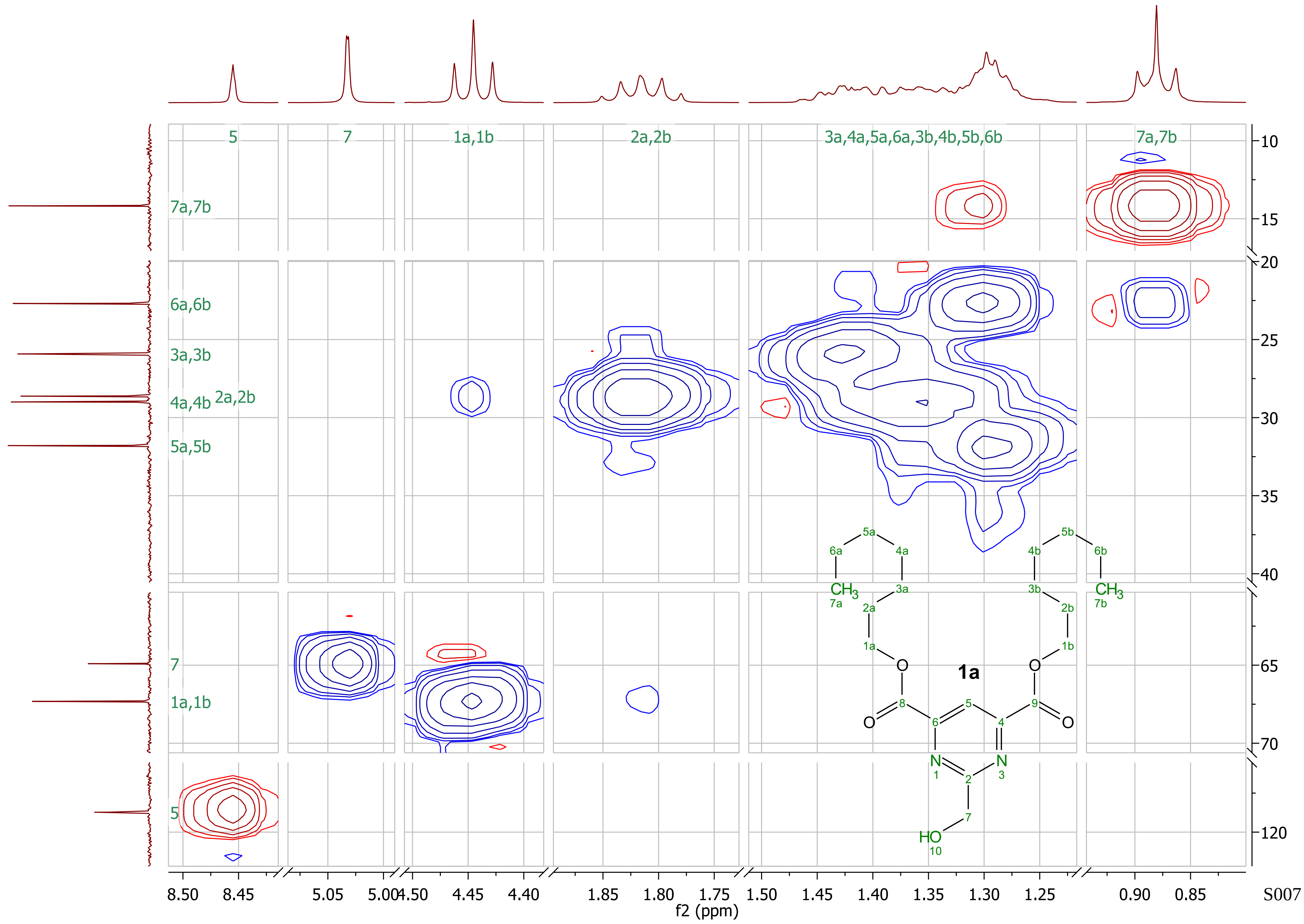
5
7
1a,1b
2a,2b
3a,4a,5a,6a,3b,4b,5b,6b
7a,7b
6a,6b
3a,3b
4a,4b
5a,5b
1a,1b
1a
f1 (ppm)
f2 (ppm)

13C HMBC

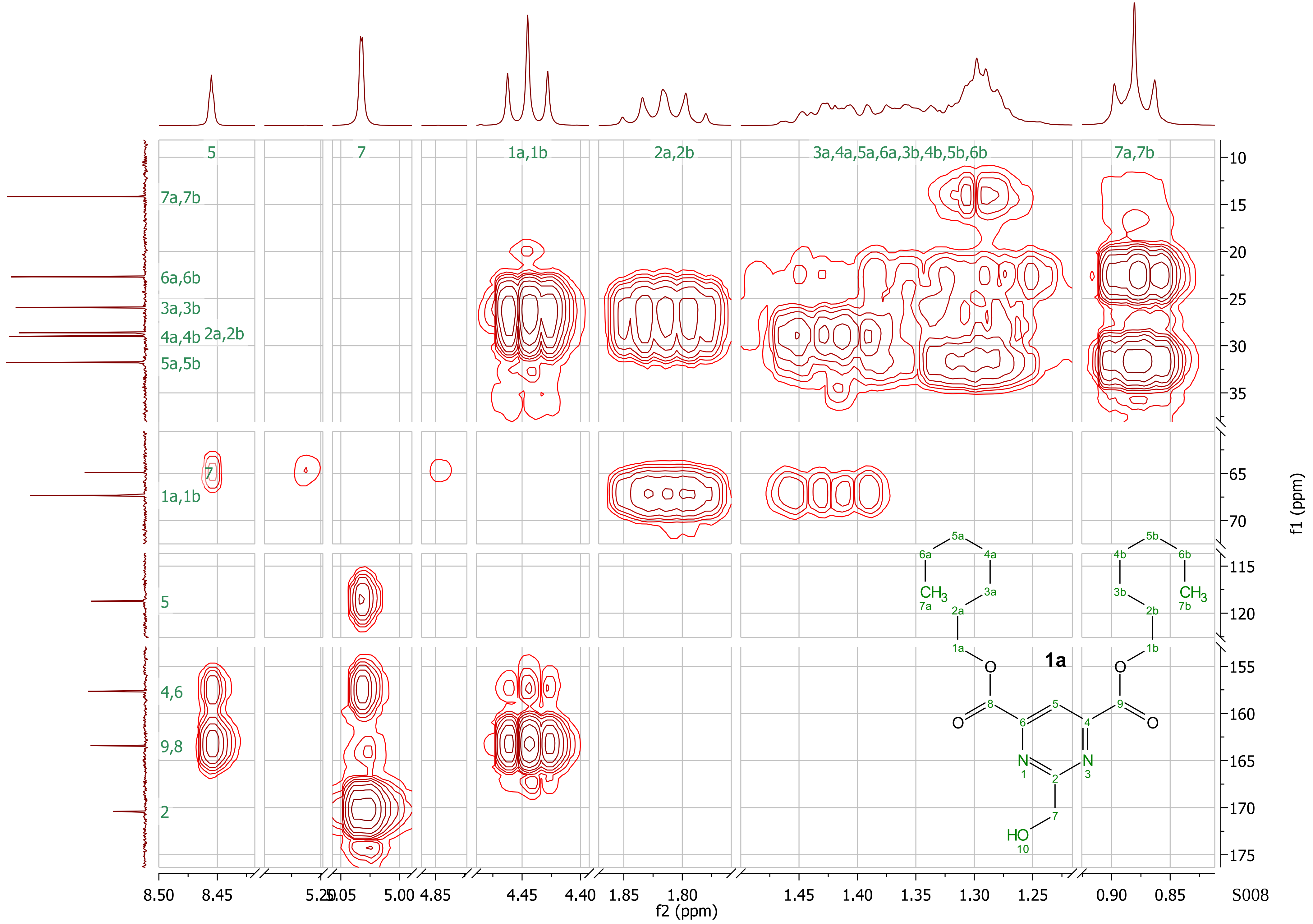

S008

15N HMBC

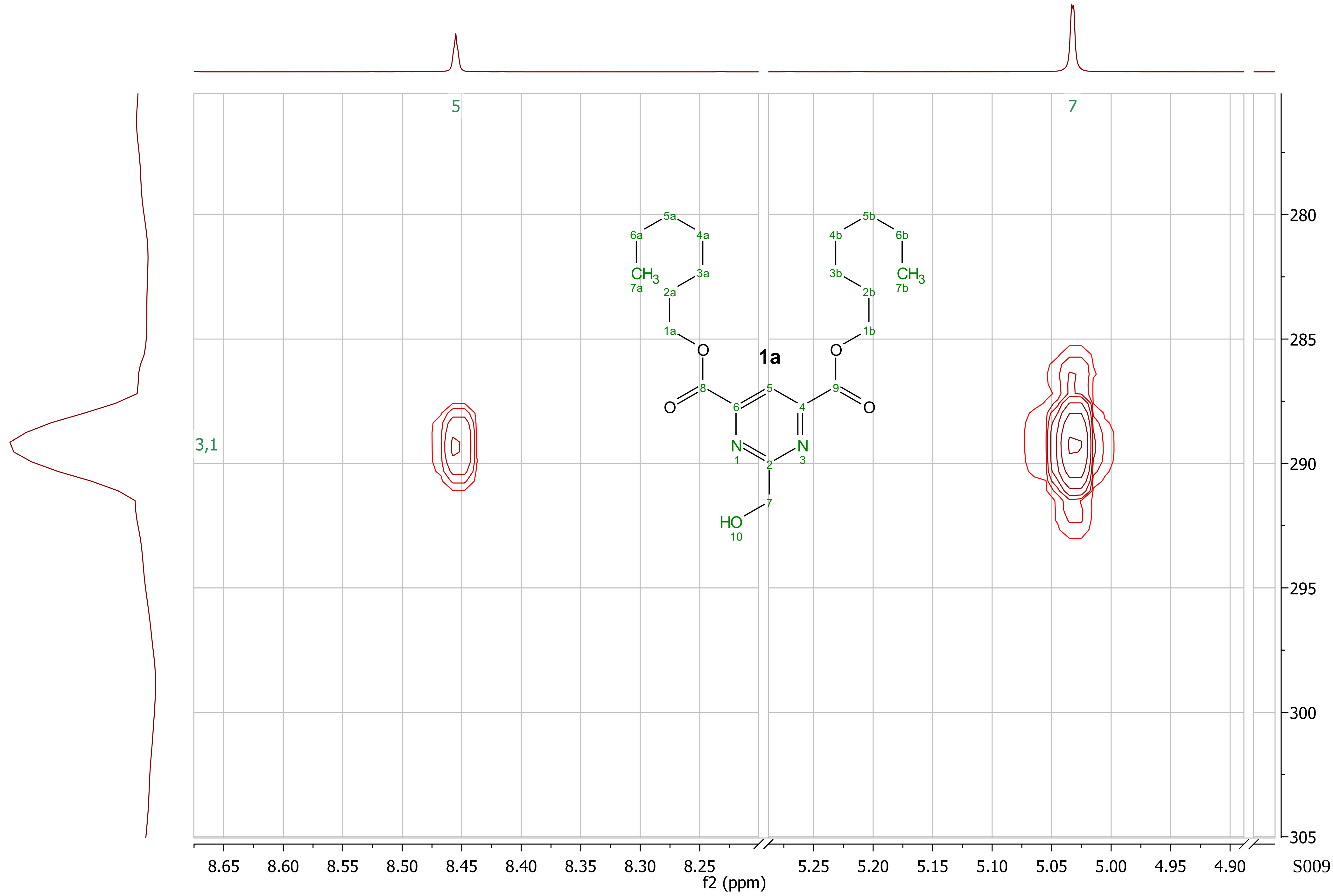

S009

15N HMBC f1 Projection ^{15}N NMR (41 MHz, $CDCl_3$) δ 289.21.

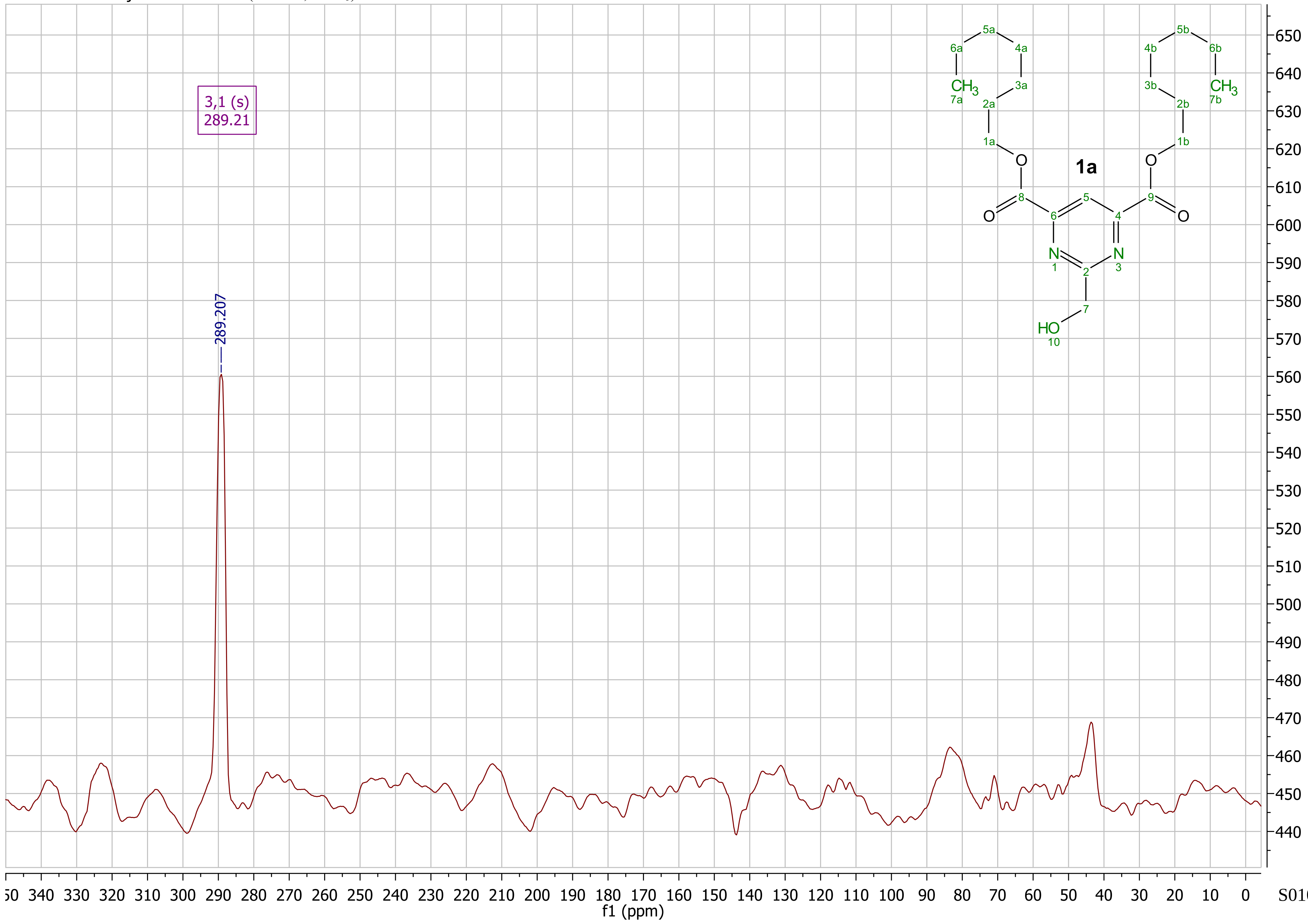

S010

1H ^{1}H NMR (400 MHz, $CDCl_3$) δ 8.46 (app t, J = 0.8 Hz, 1H), 5.04 (d, J = 4.5 Hz, 2H), 4.45 (t, J = 6.8 Hz, 4H), 3.62 (t, J = 5.4 Hz, 1H), 1.82 (quint, J = 7.2 Hz, 4H), 1.51 – 1.18 (m, 20H), 0.88 (app t, J = 6.8 Hz, 6H).

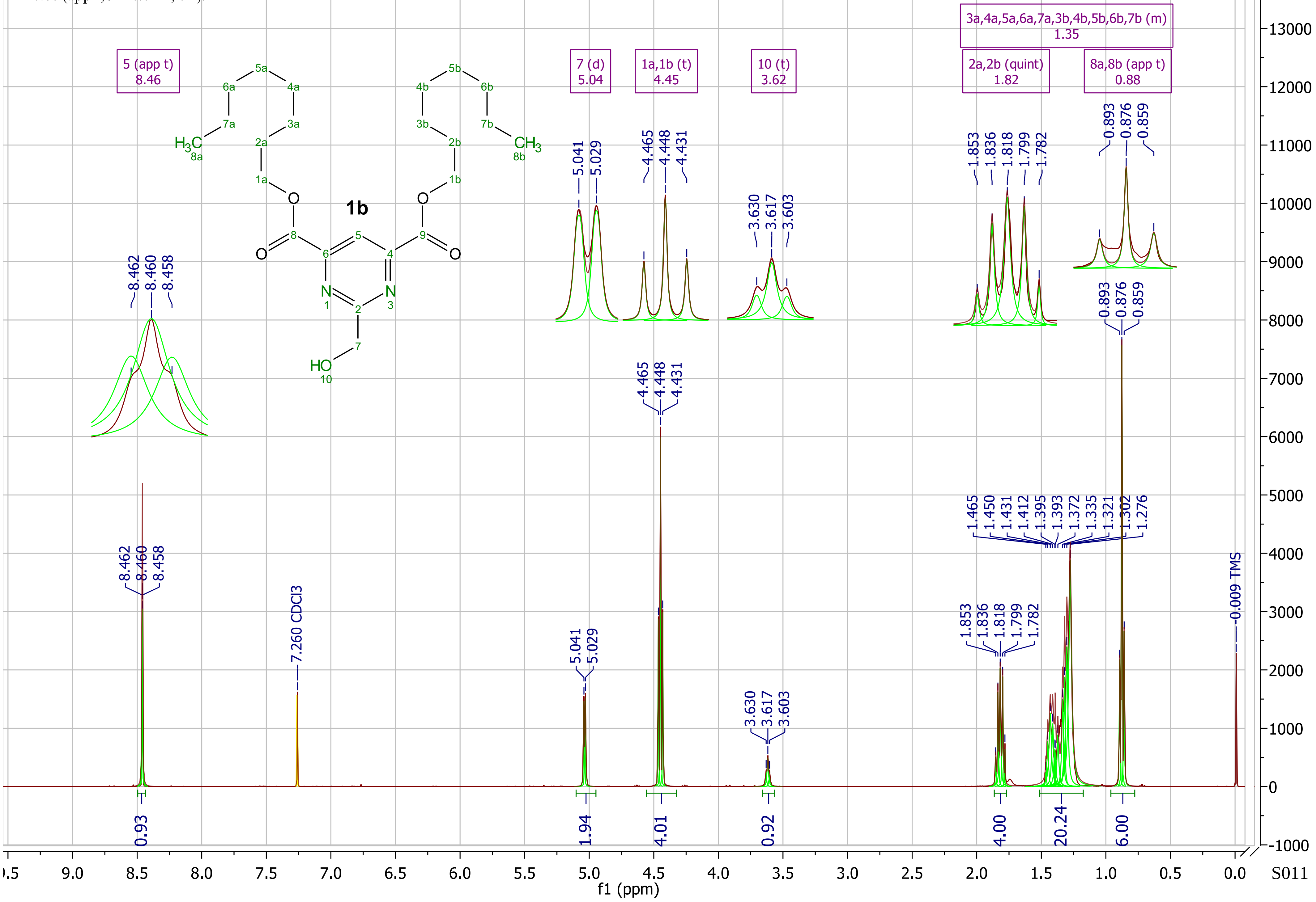

13C ^{13}C NMR (101 MHz, $CDCl_3$) δ 170.4, 163.4 (sym, 2C), 157.7 (sym, 2C), 118.7, 67.3 (sym, 2C), 64.9, 31.9 (sym, 2C), 29.3 (sym, 2C), 29.3 (sym, 2C), 28.6 (sym, 2C), 26.0 (sym, 2C), 22.8 (sym, 2C), 14.2 (sym, 2C).

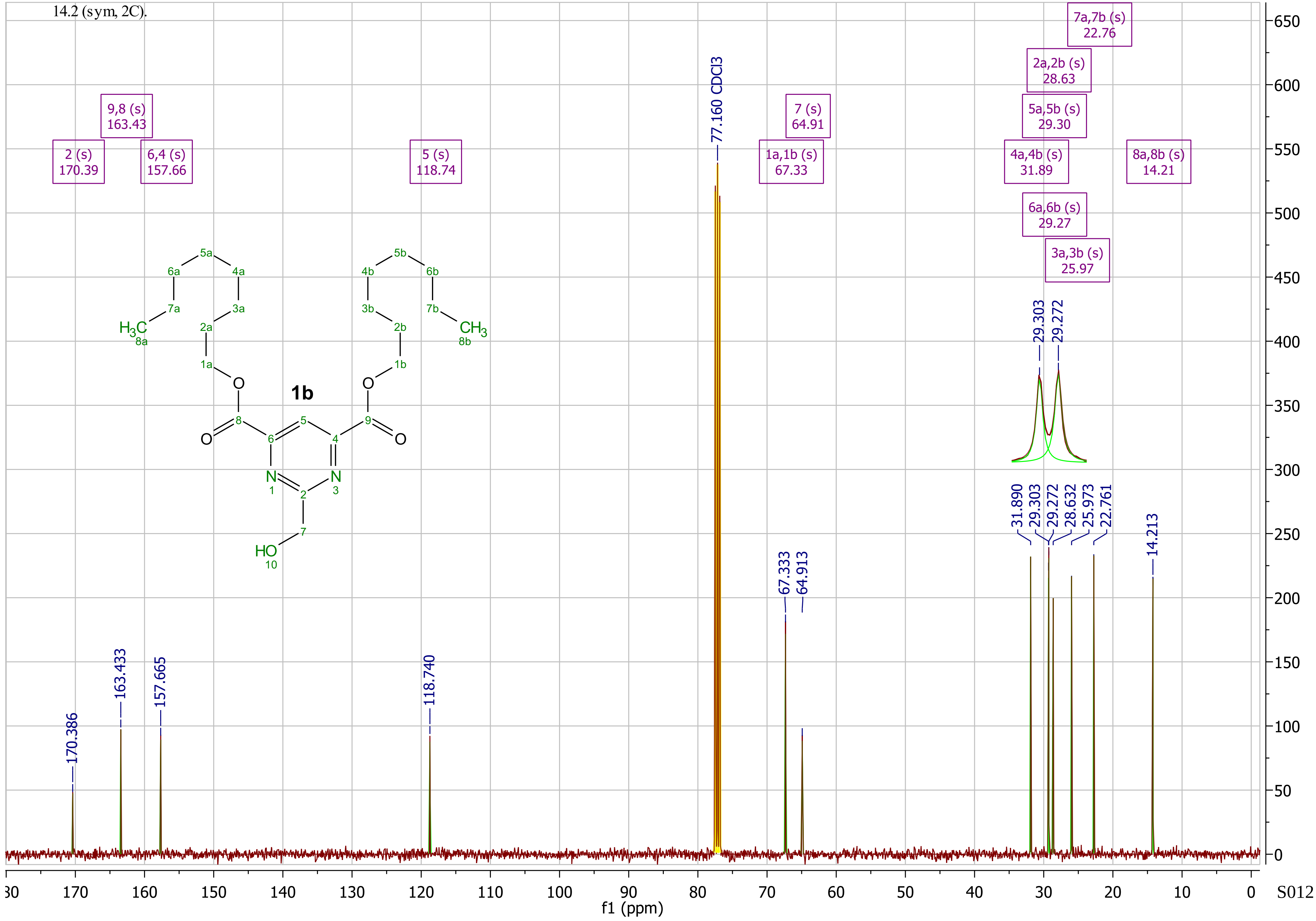

S012

13C HSQC

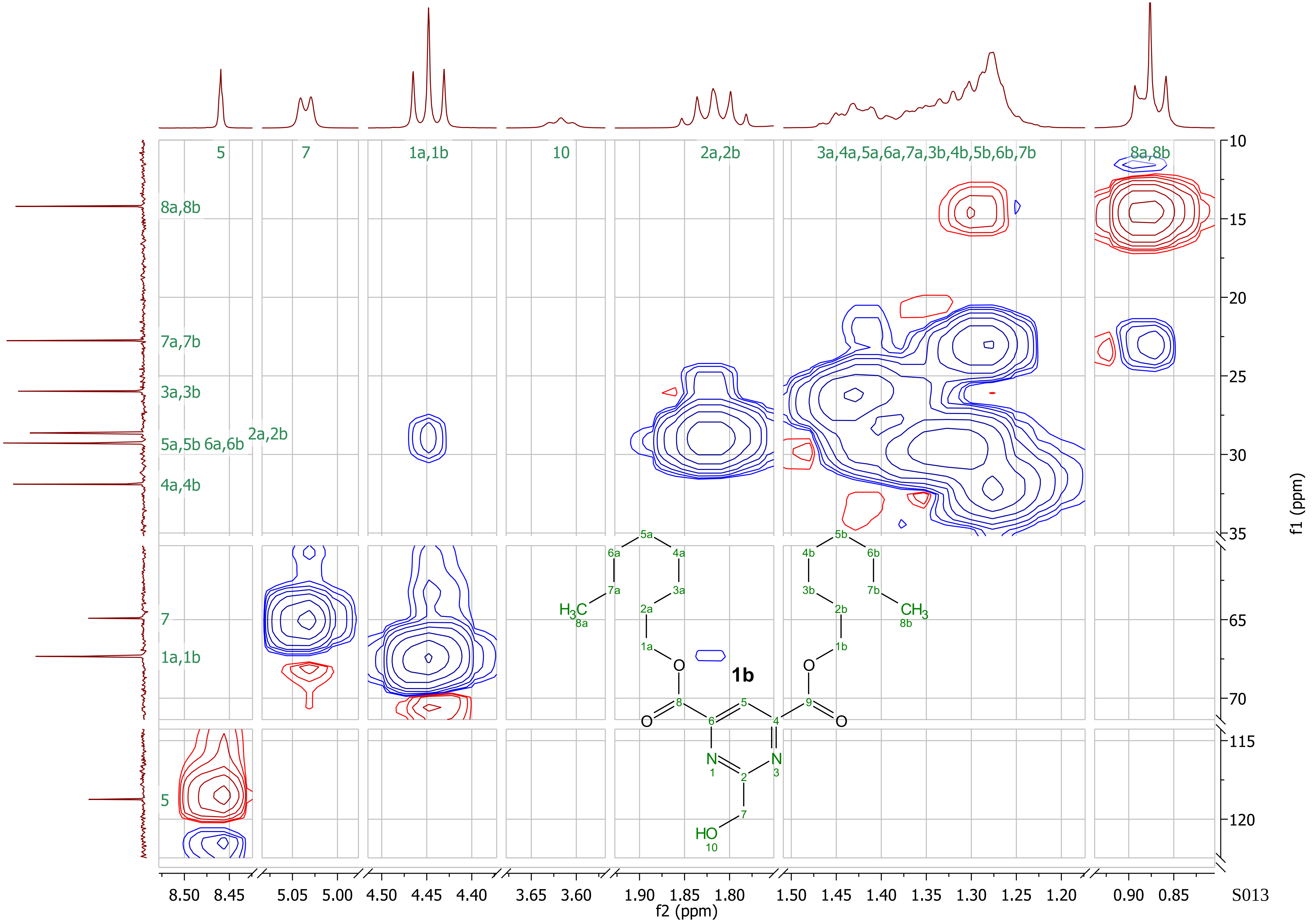

S013

13C HMBC

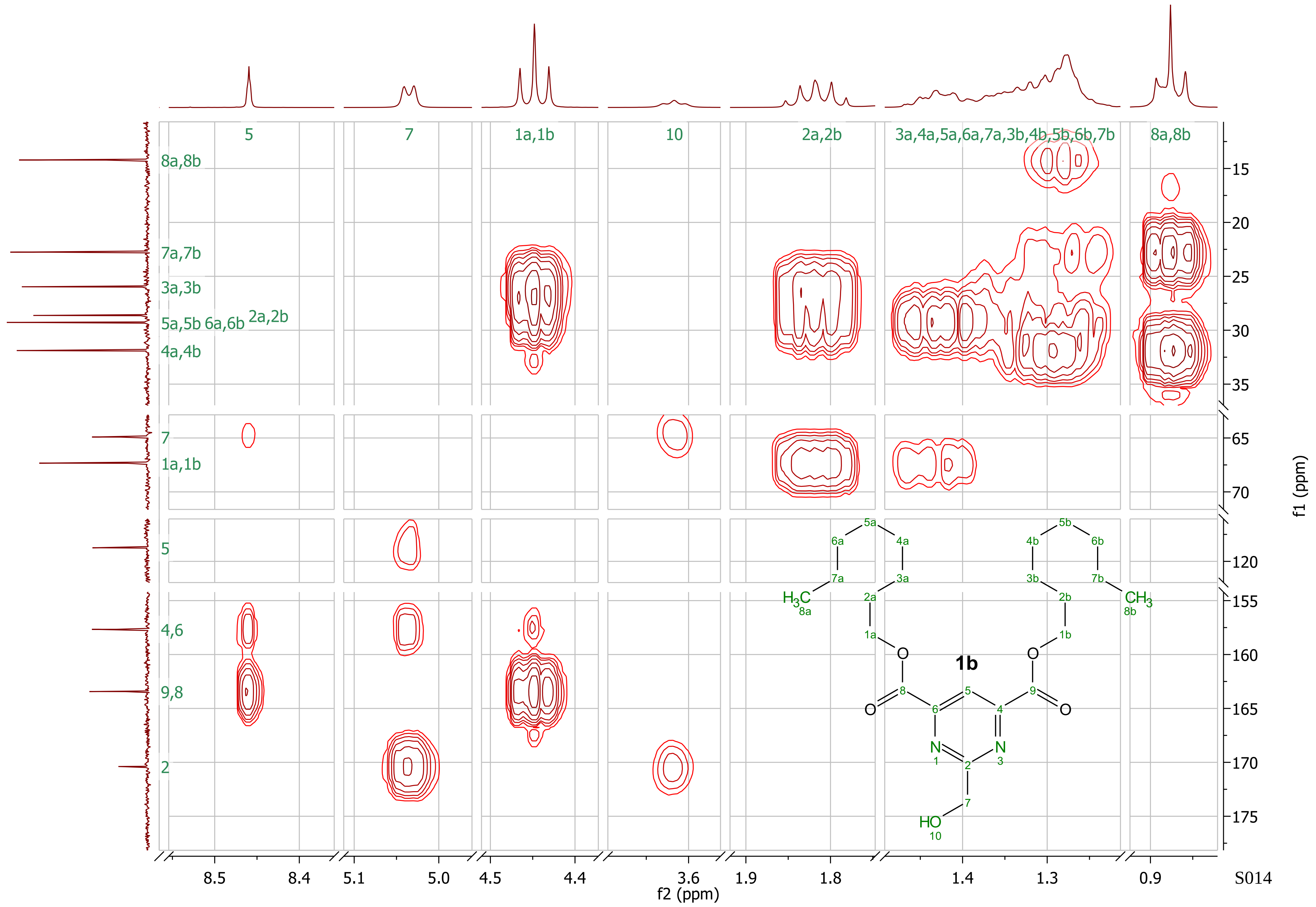
5
7
1a,1b
10
2a,2b
3a,4a,5a,6a,7a,3b,4b,5b,6b,7b
8a,8b
8a,8b
7a,7b
3a,3b
5a,5b 6a,6b 2a,2b
4a,4b
7
1a,1b
5
4,6
9,8
2
1b
f1 (ppm)
f2 (ppm)

15N HMBC

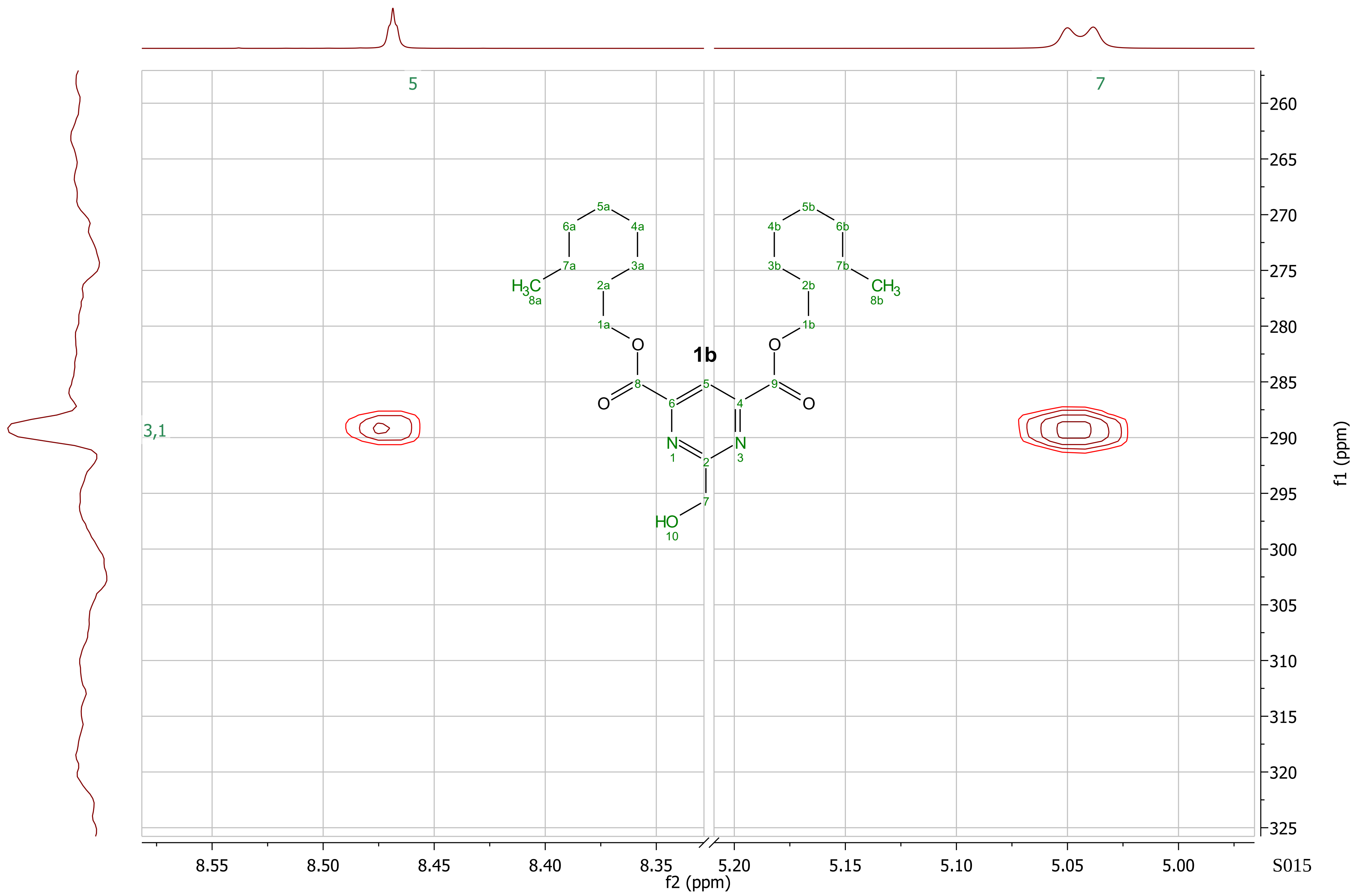

S015

15N HMBC f1 Projection ^{15}N NMR (41 MHz, $CDCl_3$) δ 289.29.

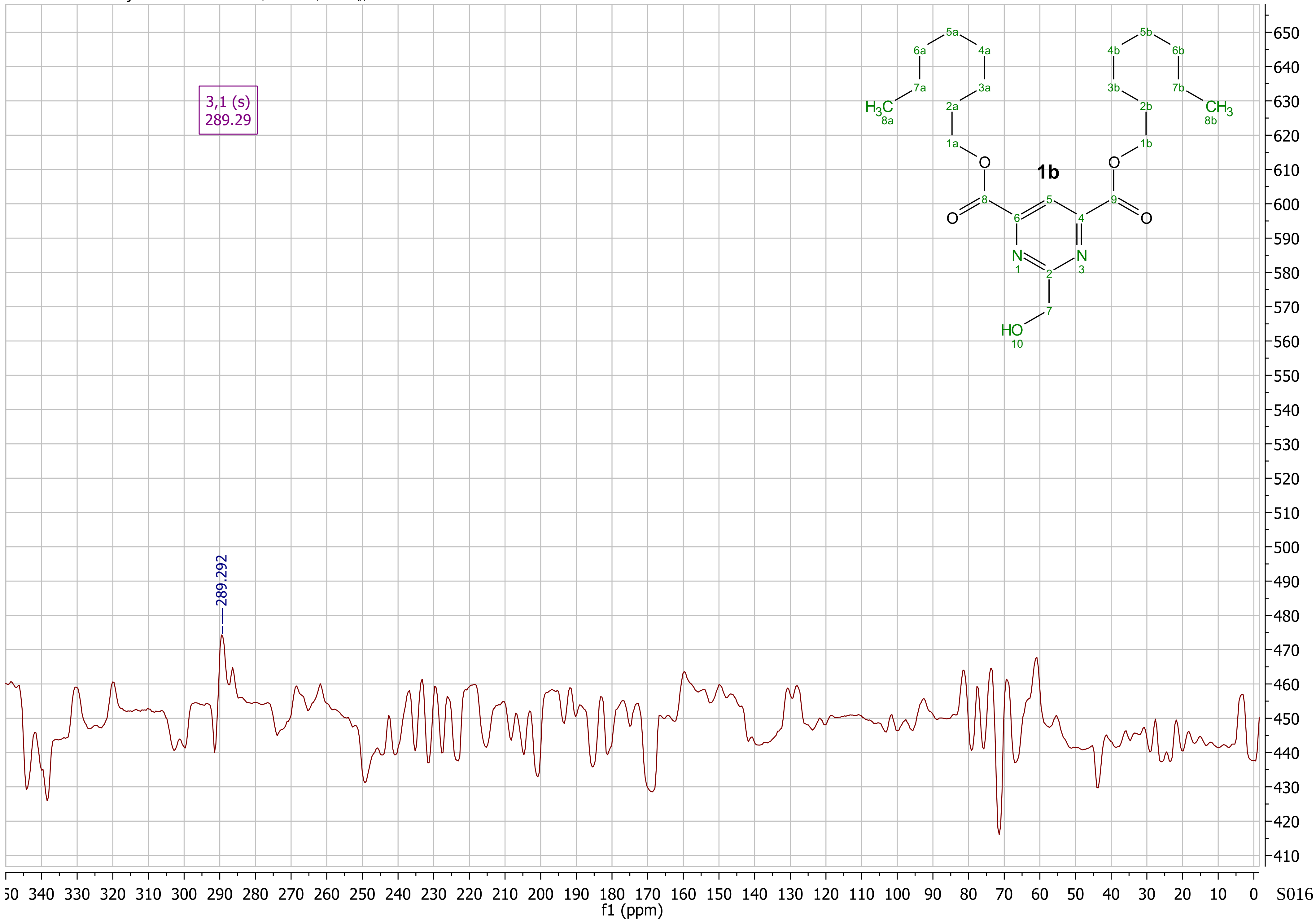

1H ^{1}H NMR (400 MHz, $CDCl_3$) δ 8.46 (app t, J = 0.7 Hz, 1H), 5.04 (s, 2H), 4.45 (t, J = 6.8 Hz, 4H), 3.59 (br s, 1H), 1.82 (quint, J = 7.1 Hz, 4H), 1.54 – 1.15 (m, 32H), 0.87 (t, J = 7.2 Hz, 6H).

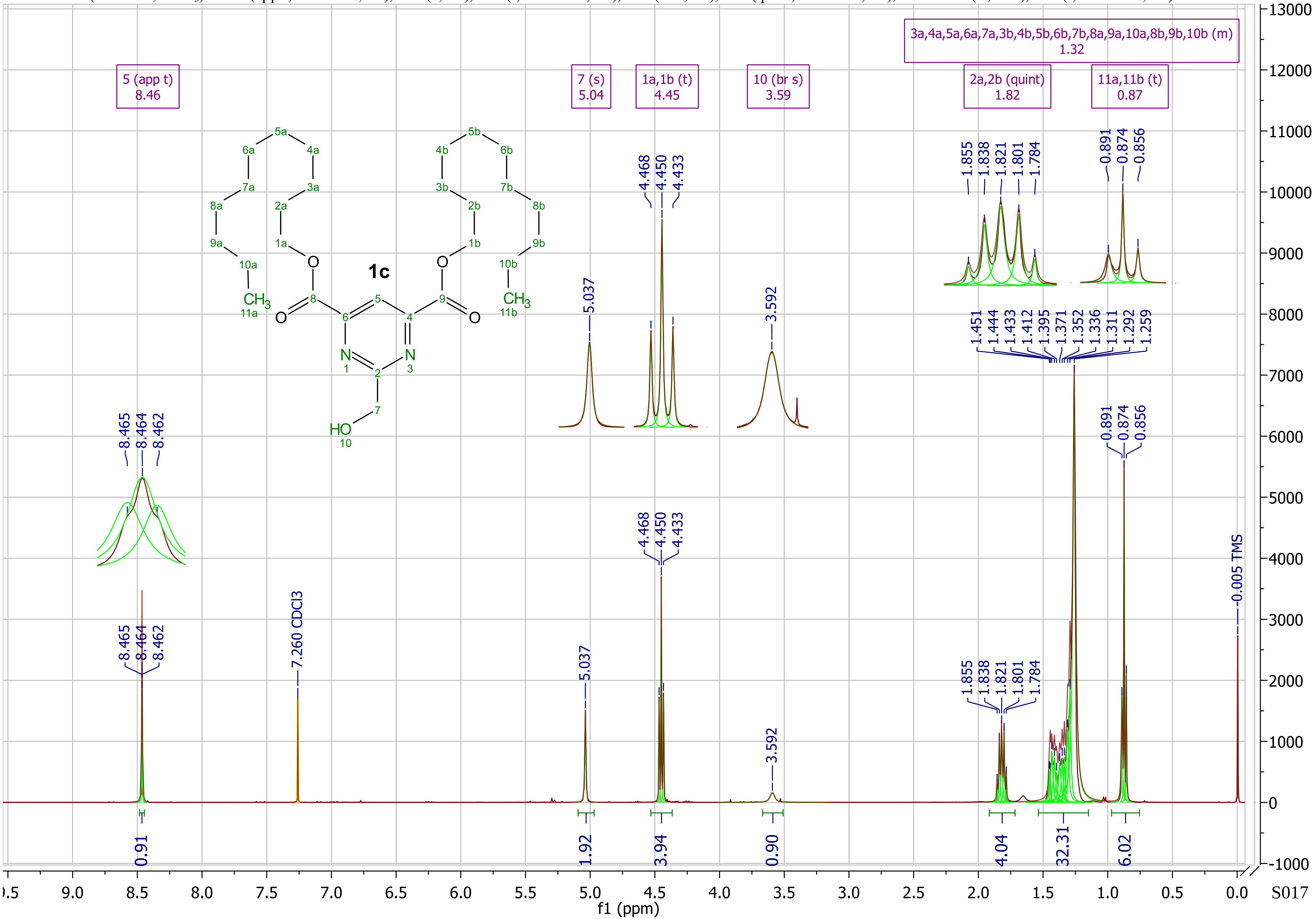

13C ^{13}C NMR (101 MHz, $CDCl_3$) δ 170.4, 163.4 (sym, 2c), 157.7 (sym, 2c), 118.8, 67.3 (sym, 2c), 64.9, 32.0 (sym, 2c), 29.73 (sym, 2c), 29.71 (sym, 2c), 29.6 (sym, 2c), 29.5 (sym, 2c), 29.4 (sym, 2c), 28.6 (sym, 2c), 26.0 (sym, 2c), 22.8 (sym, 2c), 14.3 (sym, 2c).

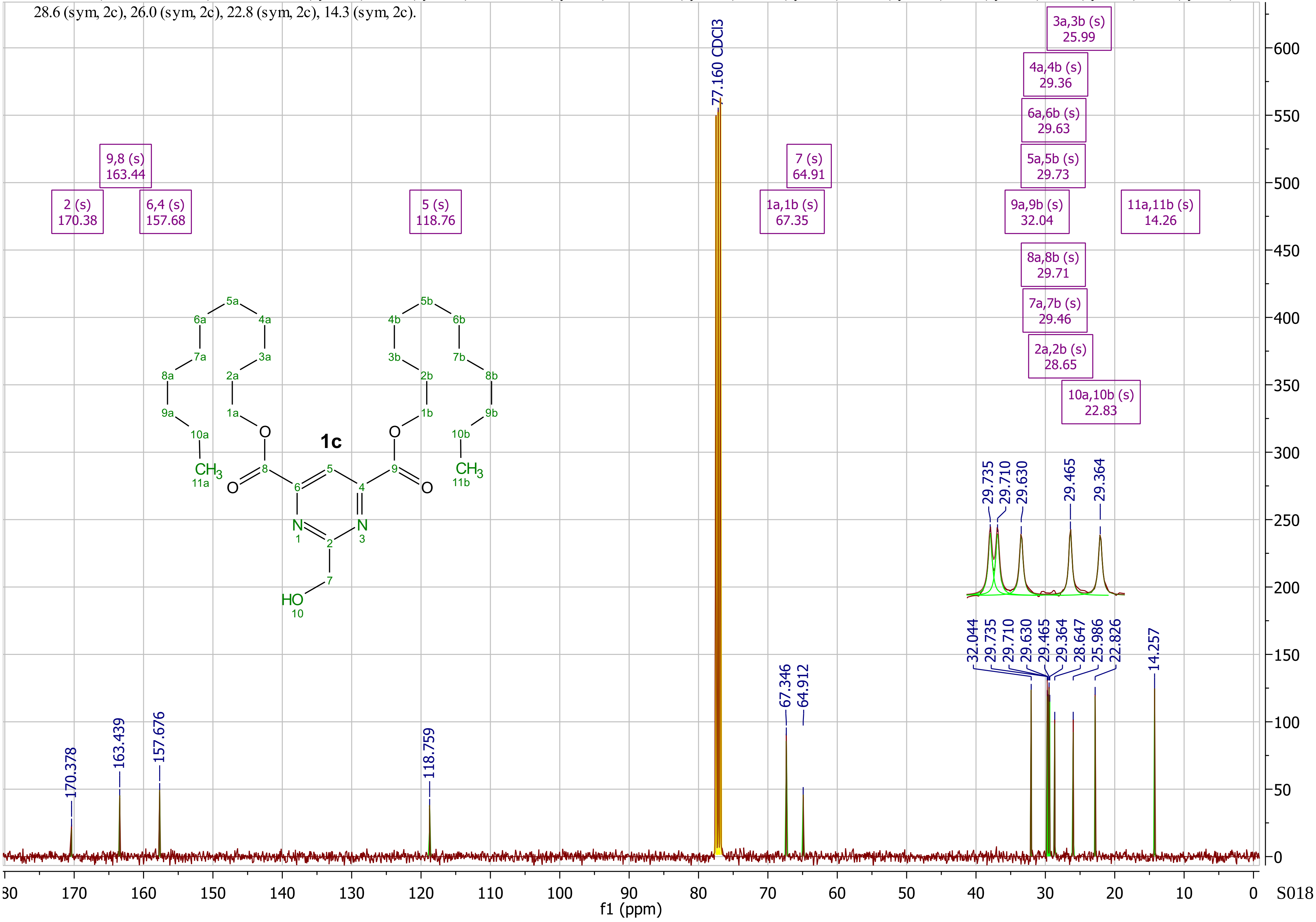

13C HSQC

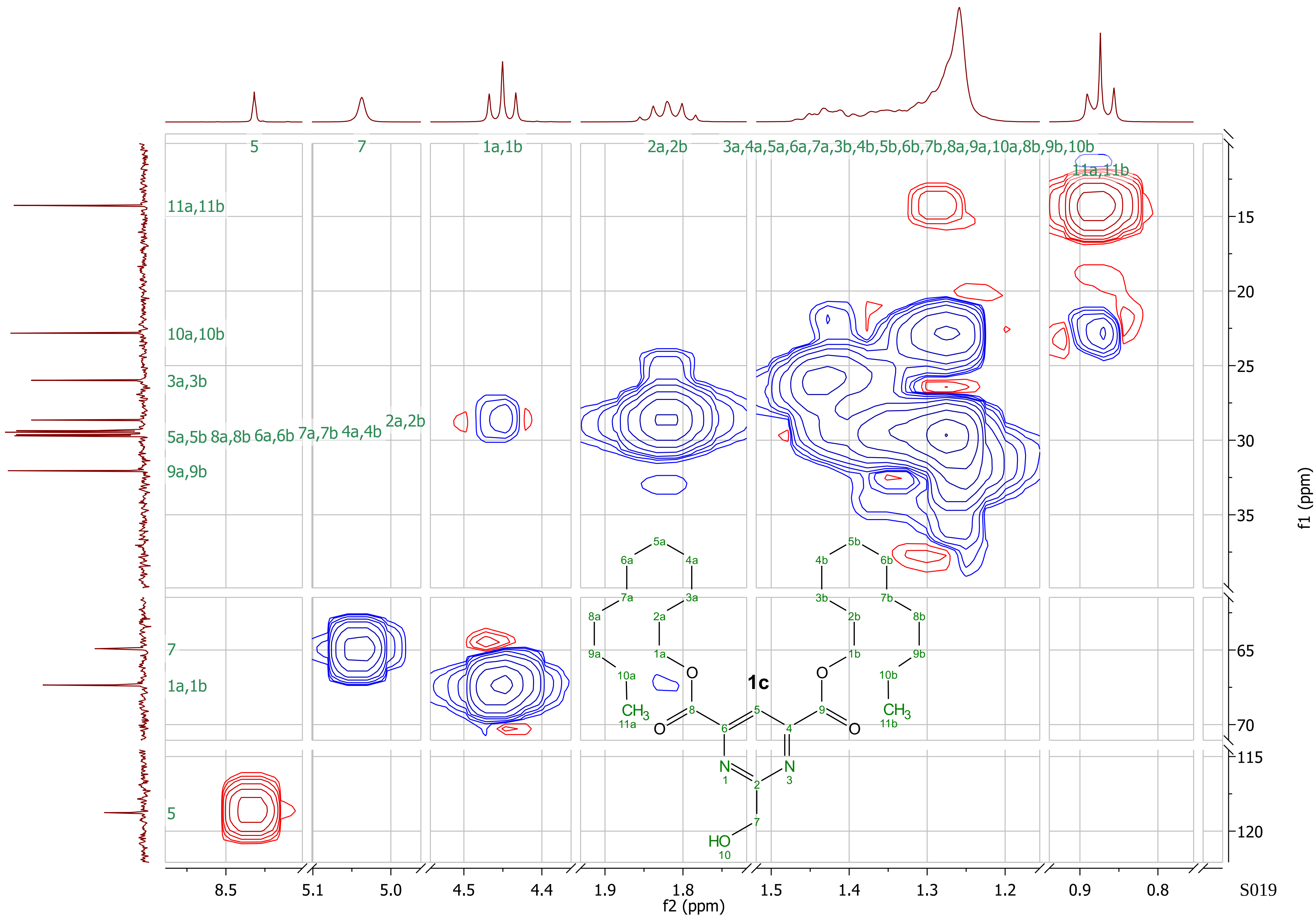

S019

13C HMBC

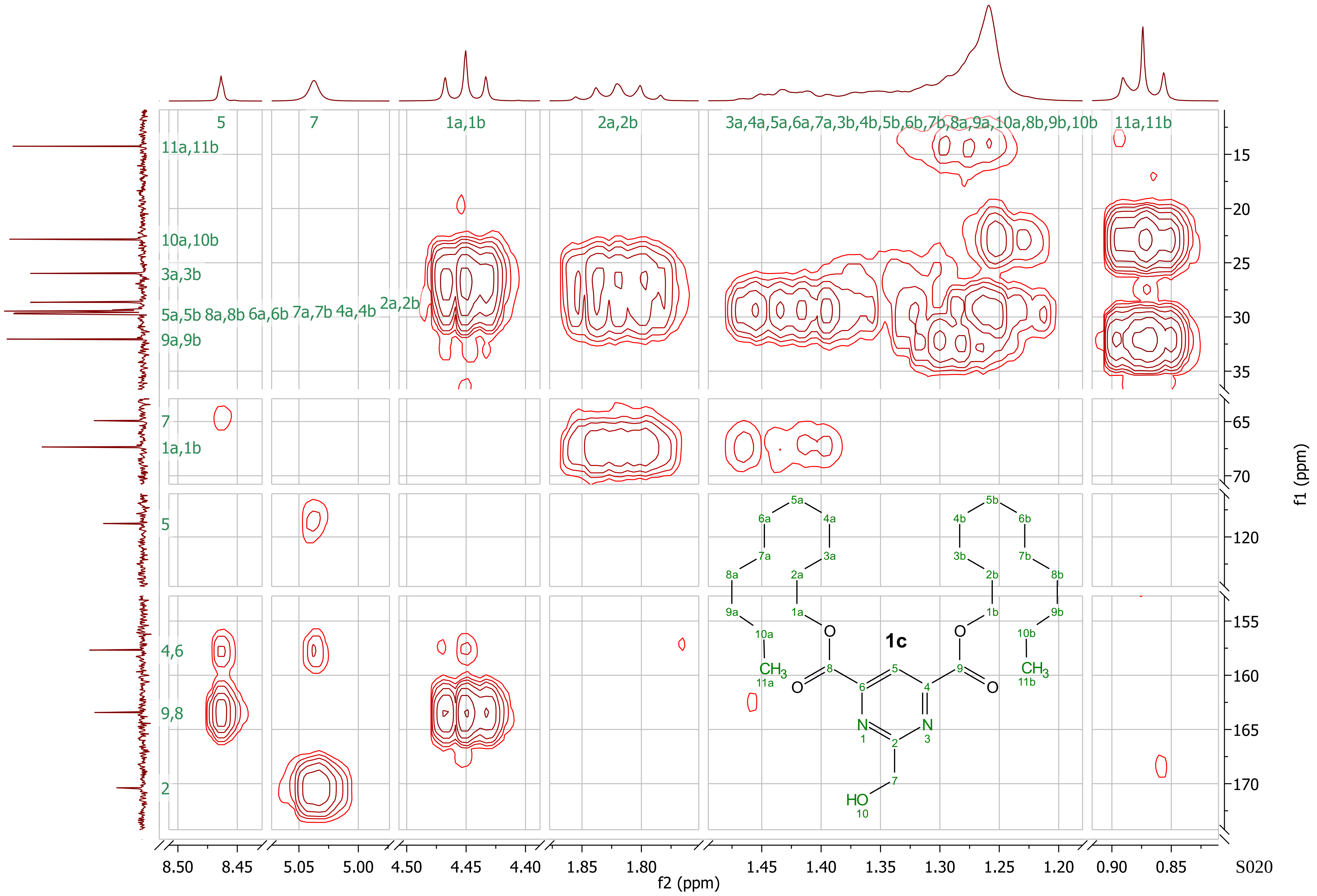

S020

15N HMBC ^{15}N NMR (41 MHz, $CDCl_3$) δ 289.20.

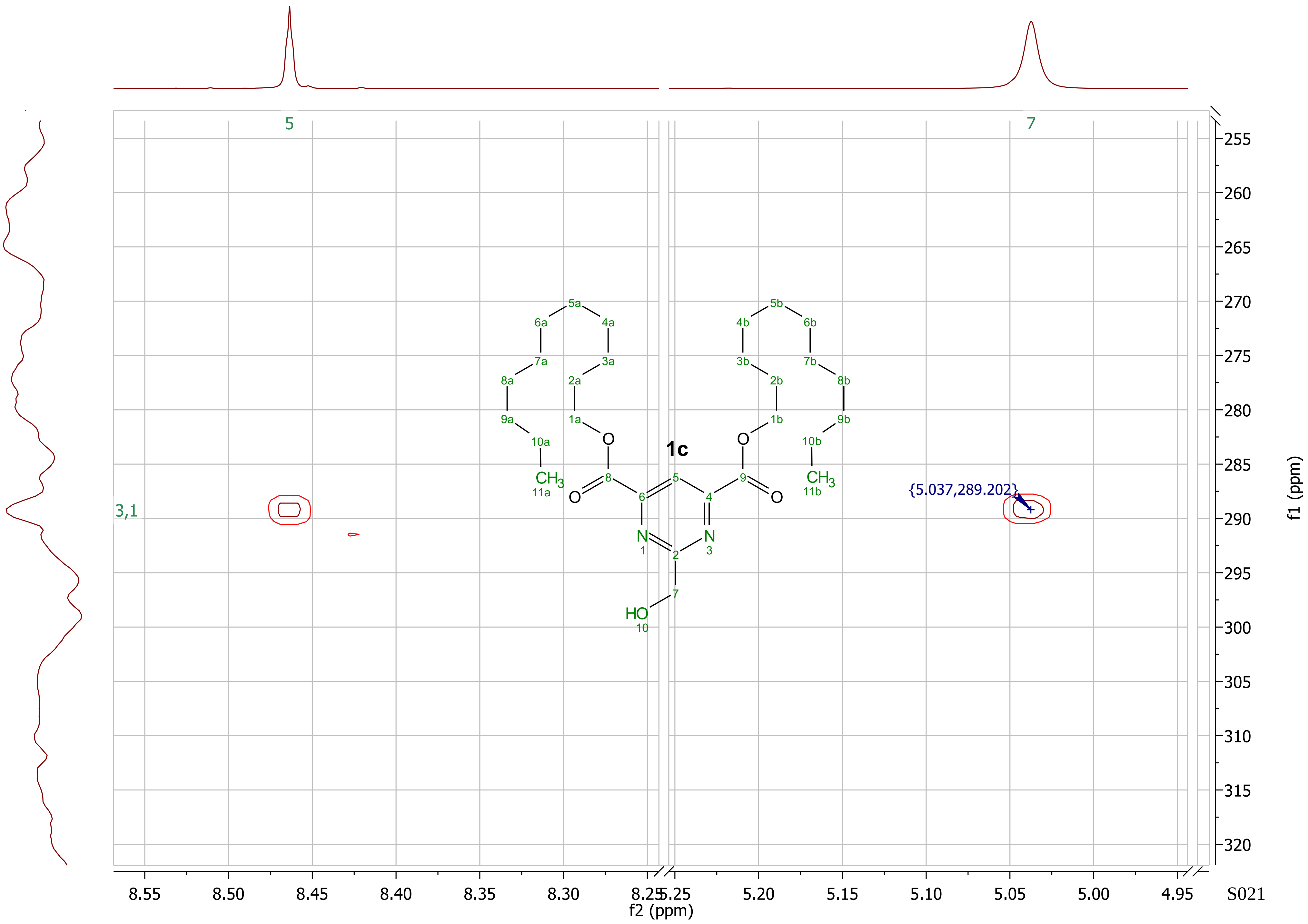

S021

1H ^{1}H NMR (400 MHz, $CDCl_3$) δ 8.46 (app t, J = 0.8 Hz, 1H), 5.18 (app quint, J = 6.1 Hz, 1H), 5.04 (app d, J = 0.8 Hz, 2H), 4.53 (q, J = 7.1 Hz, 2H), 3.55 (br s, 1H), 1.84 – 1.59 (m, 4H), 1.46 (t, J = 7.1 Hz, 3H), 1.42 – 1.27 (m, 4H), 0.97 (t, J = 7.4 Hz, 3H), 0.90 (app t, J = 6.9 Hz, 3H).

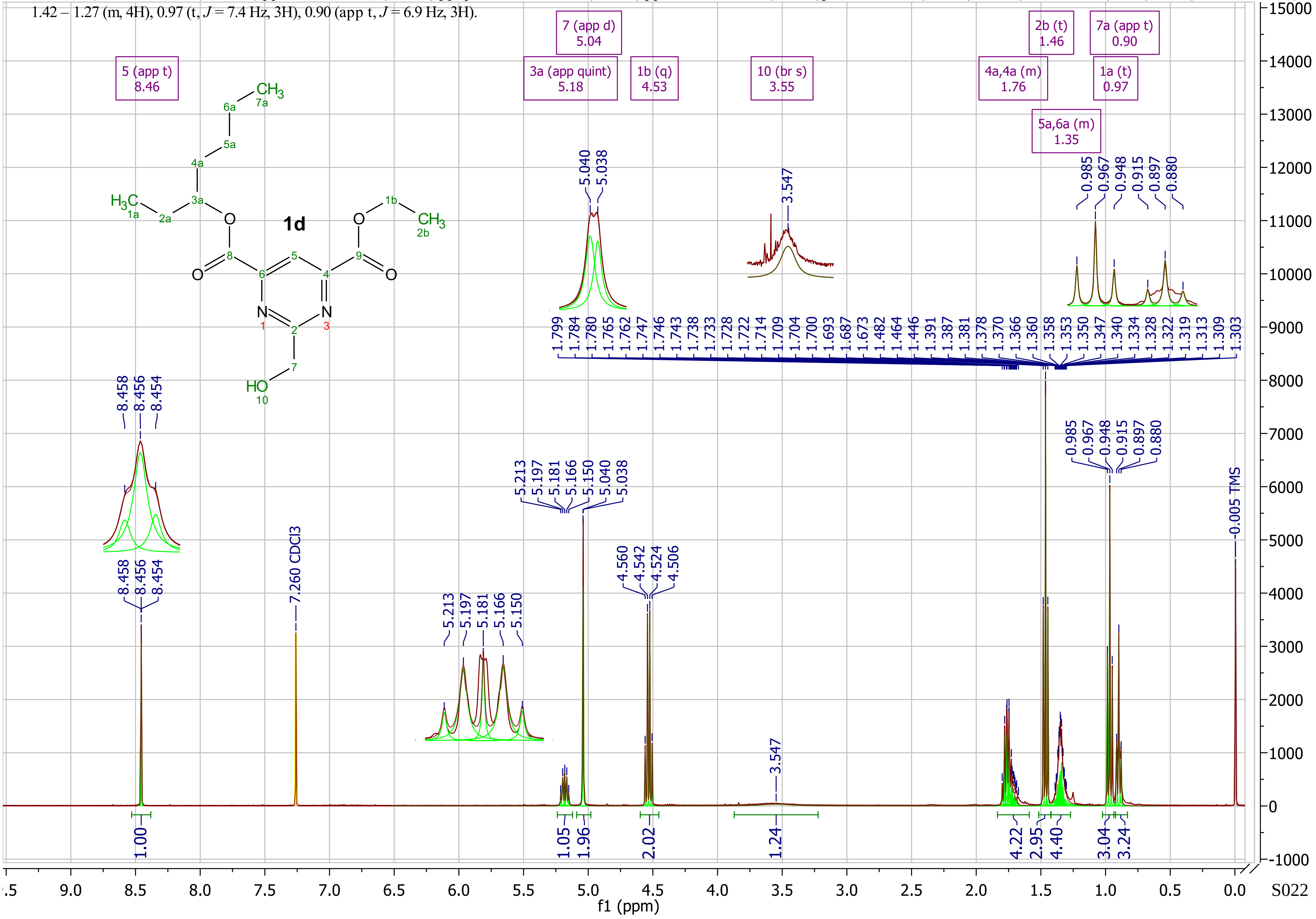

13C ^{13}C NMR (101 MHz, $CDCl_3$) δ 170.3, 163.5, 163.1, 157.9, 157.7, 118.7, 79.3, 64.9, 63.2, 33.4, 27.6, 27.1, 22.7, 14.3, 14.1, 9.8.

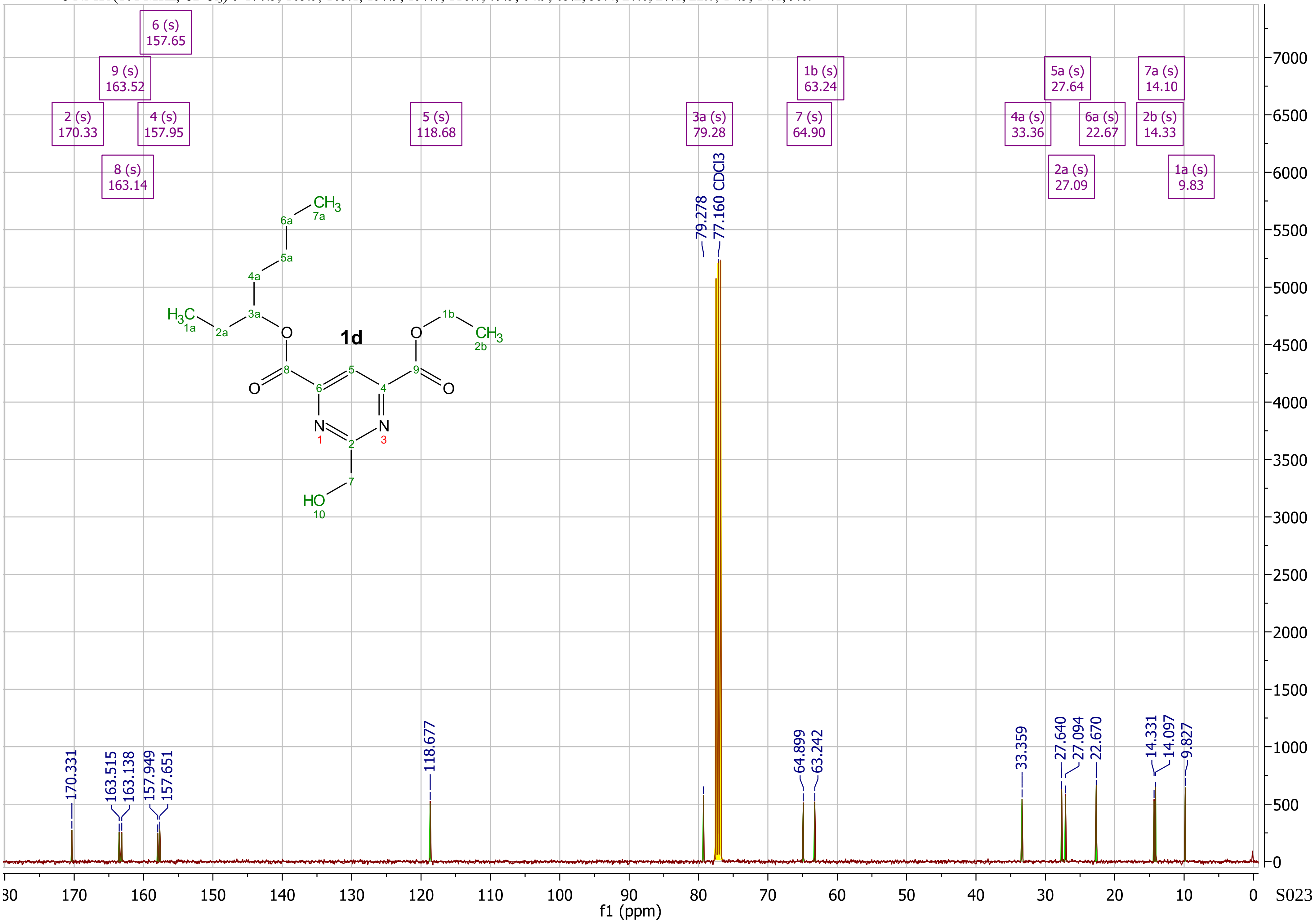

S023

13C HSQC

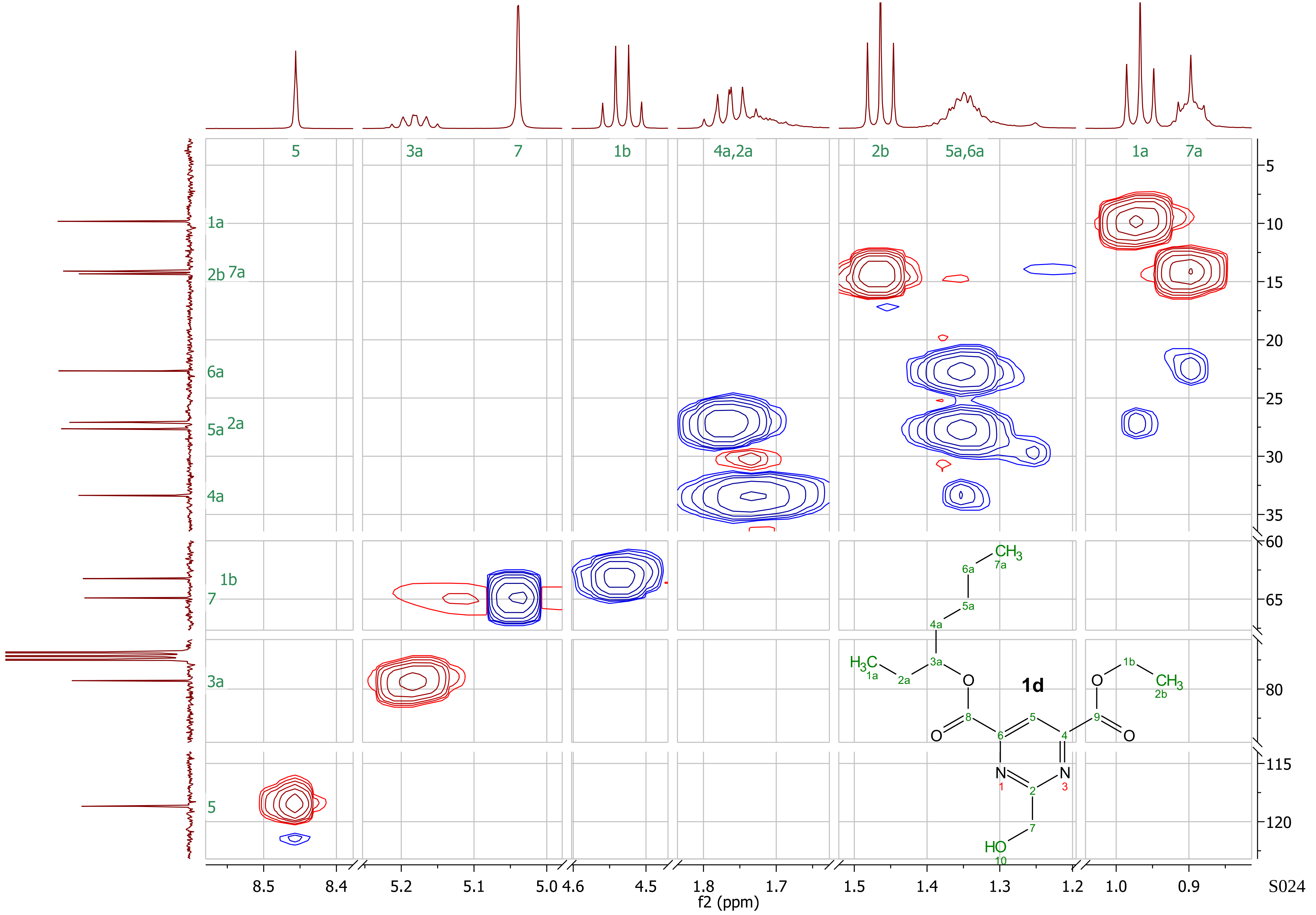

S024

13C HMBC

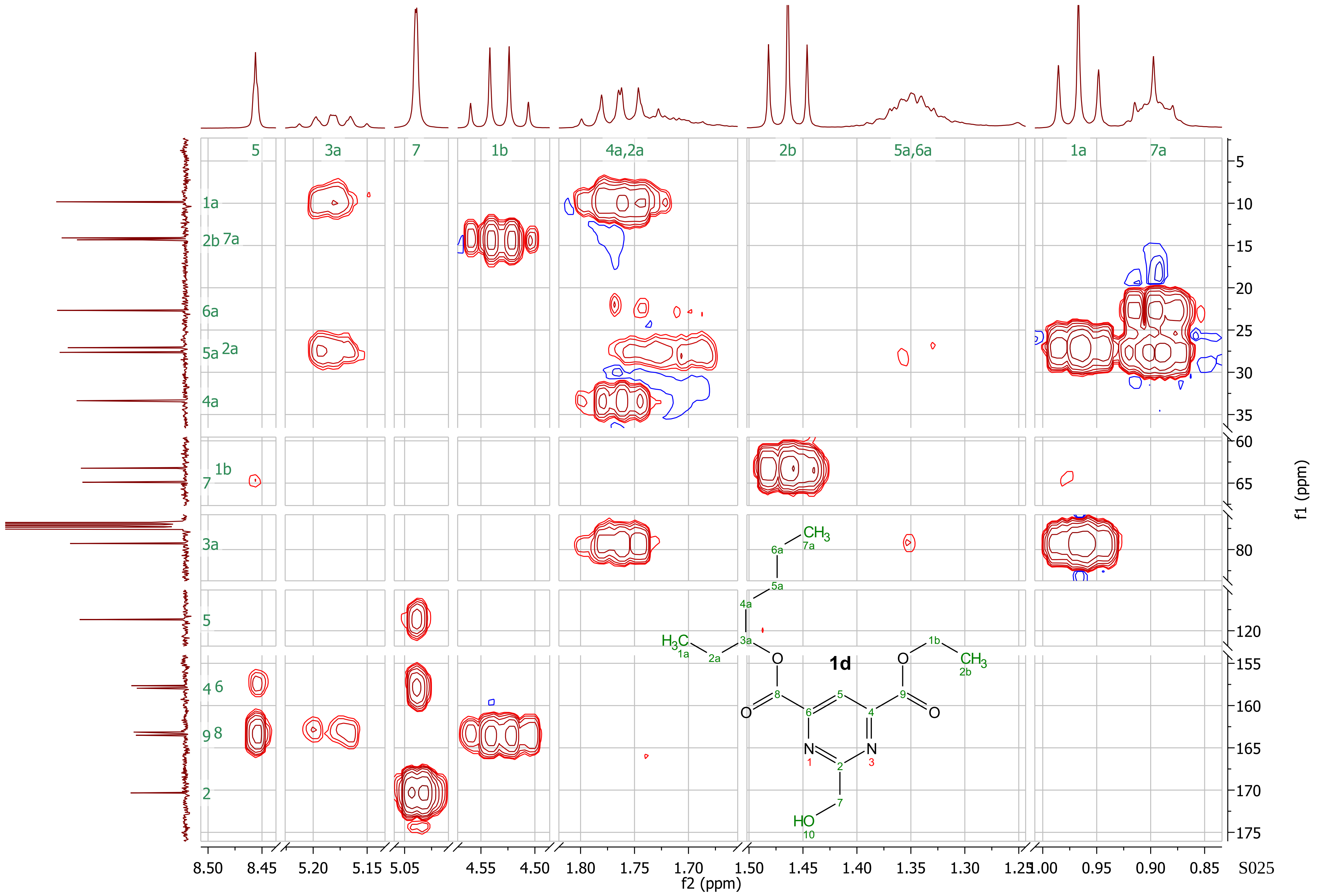

5
3a
7
1b
4a,2a
2b
5a,6a
1a
7a
1a
2b 7a
6a
5a 2a
4a
1b
7
3a
5
4 6
9 8
2
1d
f2 (ppm)
f1 (ppm)

1H ^{1}H NMR (400 MHz, $CDCl_3$) δ 8.41 (app t, J = 0.8 Hz, 1H), 5.16 (app quint, J = 6.2 Hz, 2H), 5.02 (d, J = 5.3 Hz, 2H), 3.71 (t, J = 5.3 Hz, 1H), 1.84 – 1.59 (m, 8H), 1.43 – 1.23 (m, 8H), 0.95 (t, J = 7.4 Hz, 6H), 0.88 (app t, J = 7.1 Hz, 6H).

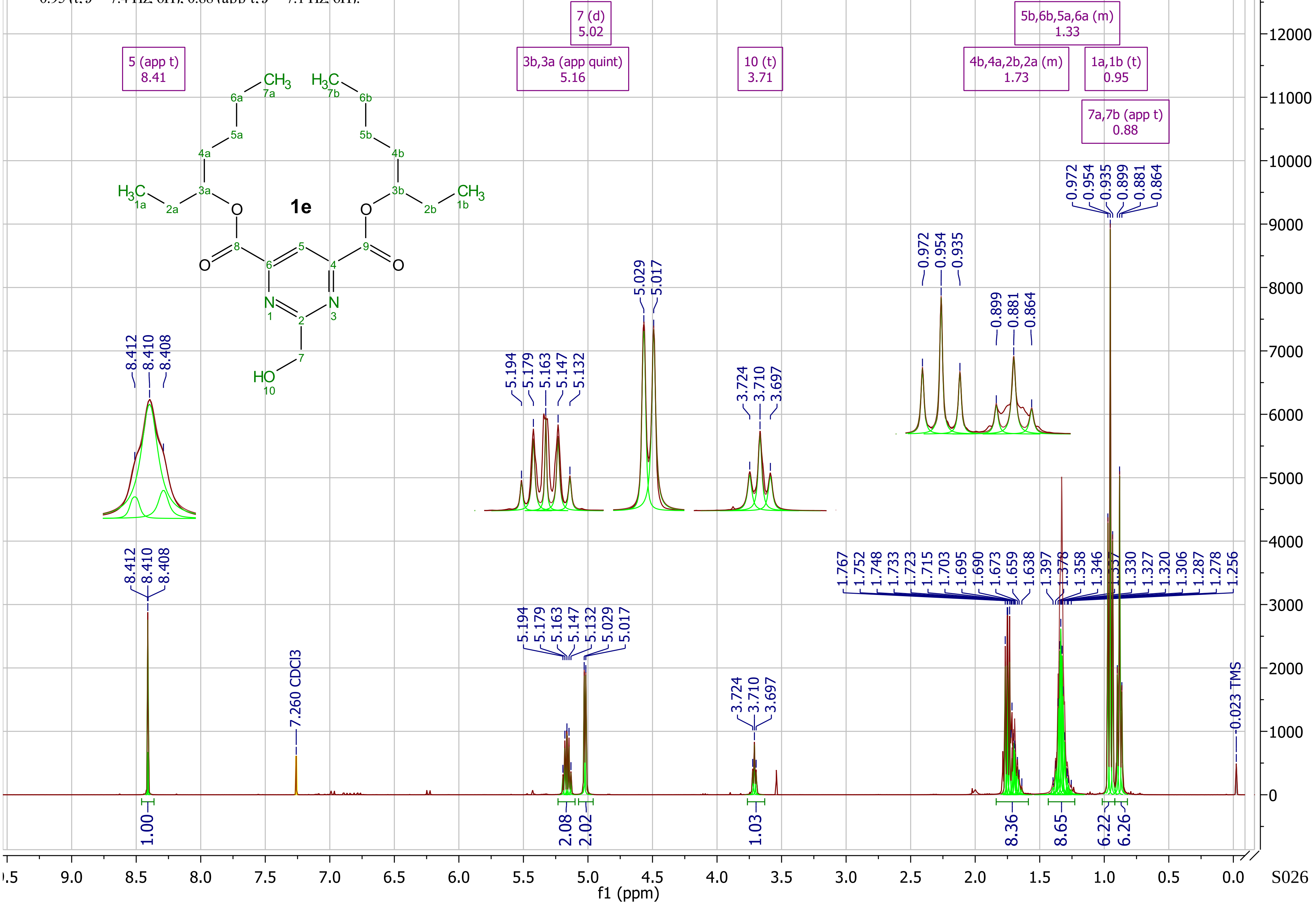

13C ^{13}C NMR (101 MHz, $CDCl_3$) δ 170.3, 163.2 (sym, 2C), 157.9 (sym, 2C), 118.5, 79.2 (sym, 2C), 64.8, 33.3 (sym, 2C), 27.6 (sym, 2C), 27.0 (sym, 2C), 22.6 (sym, 2C), 14.1 (sym, 2C), 9.8 (sym, 2C).

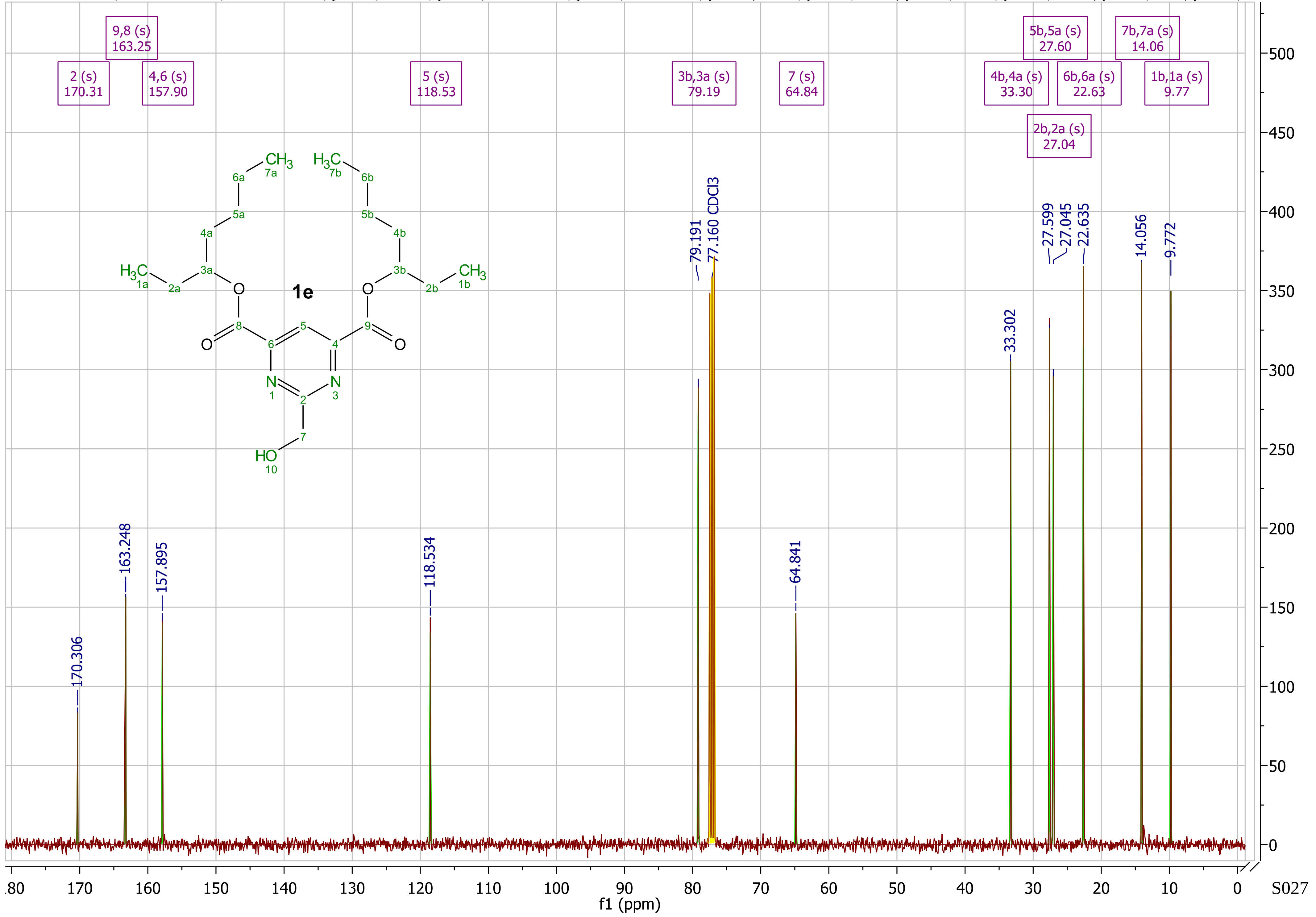

S027

13C HSQC

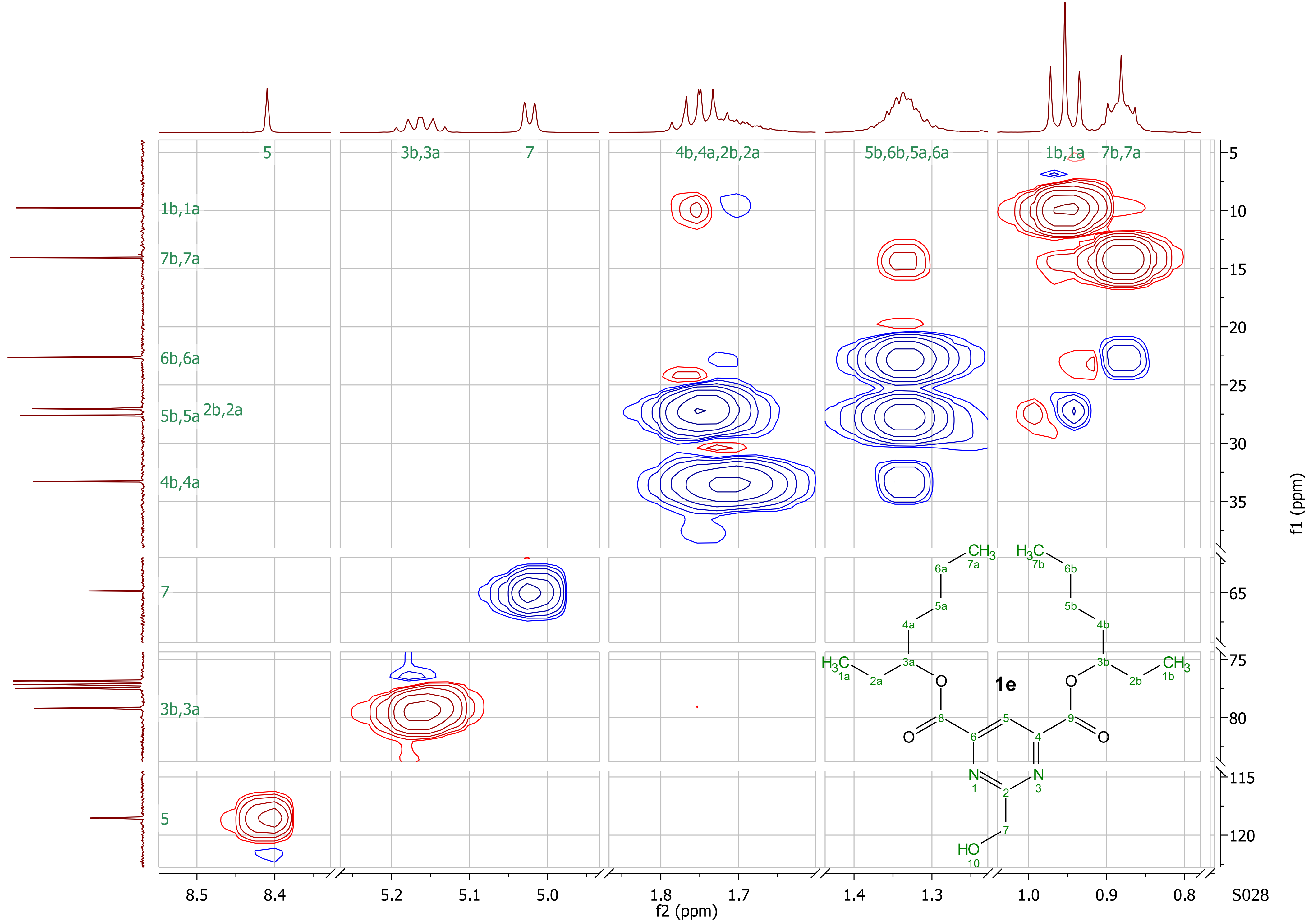

5
3b,3a
7
4b,4a,2b,2a
5b,6b,5a,6a
1b,1a
7b,7a
6b,6a
5b,5a
2b,2a
4b,4a
1e
HO
f2 (ppm)
f1 (ppm)
S028

13C HMBC

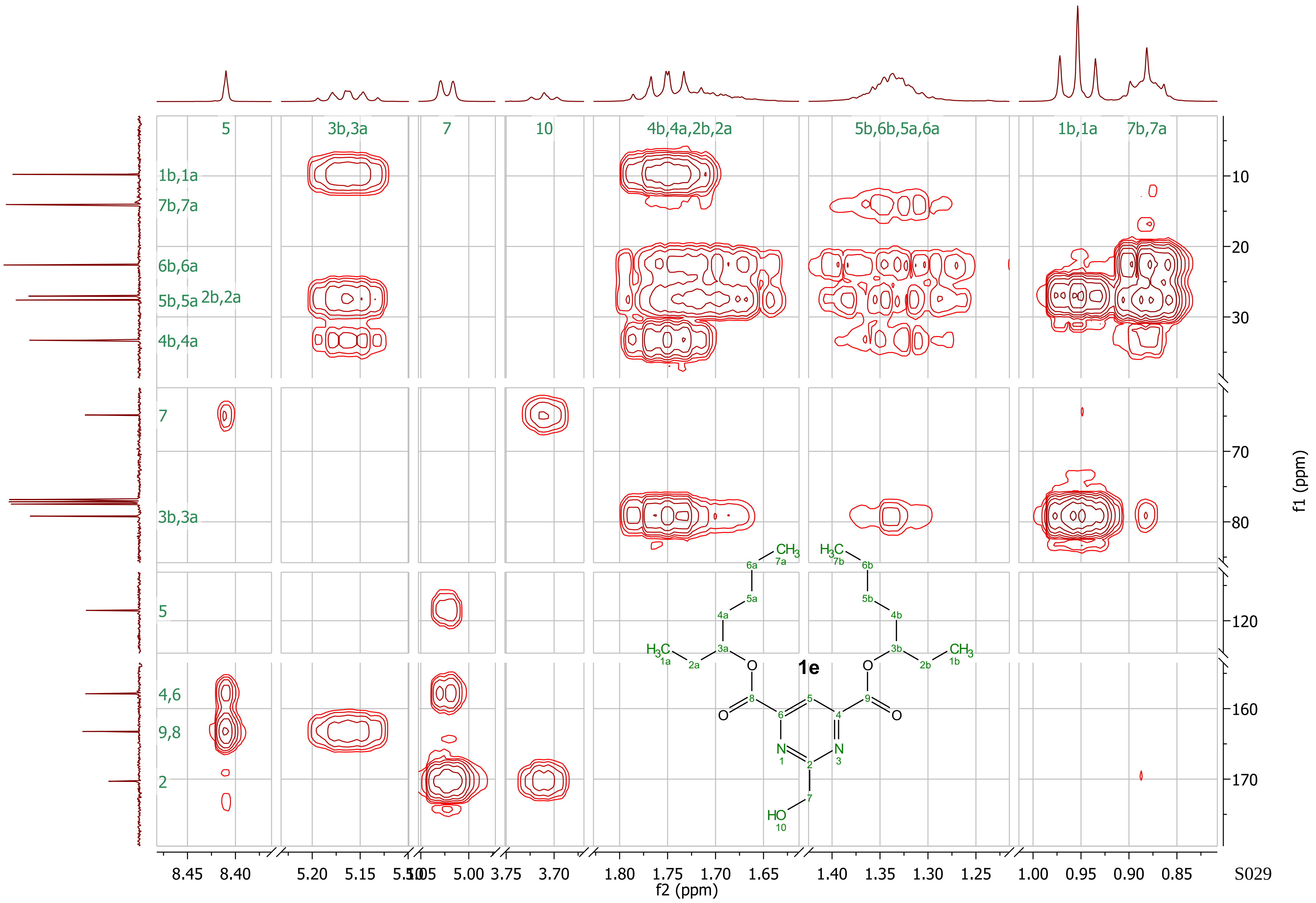

S029

15N HMBC

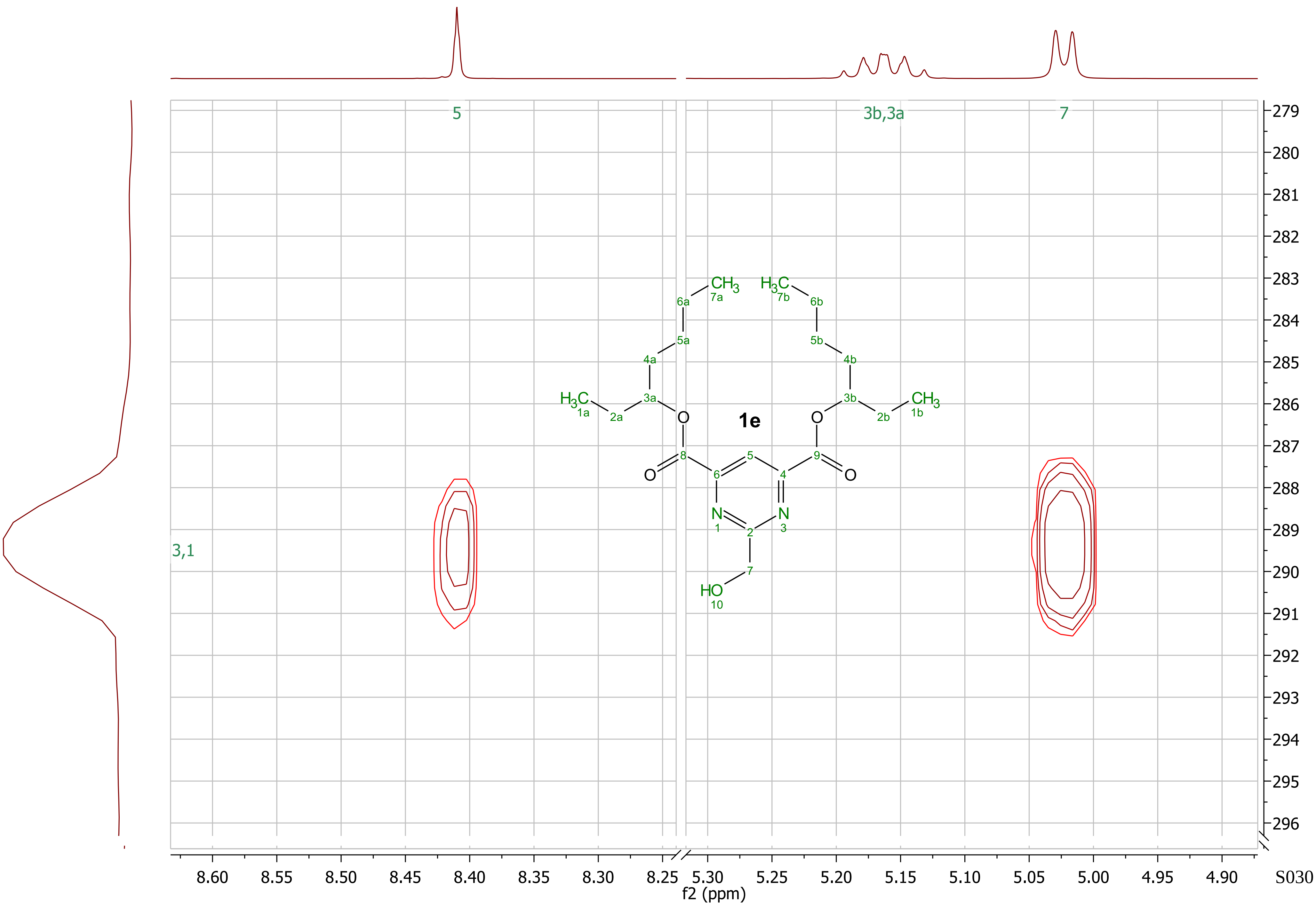

S030

15N HMBC f1 Projection ^{15}N NMR (41 MHz, $CDCl_3$) δ 289.47.

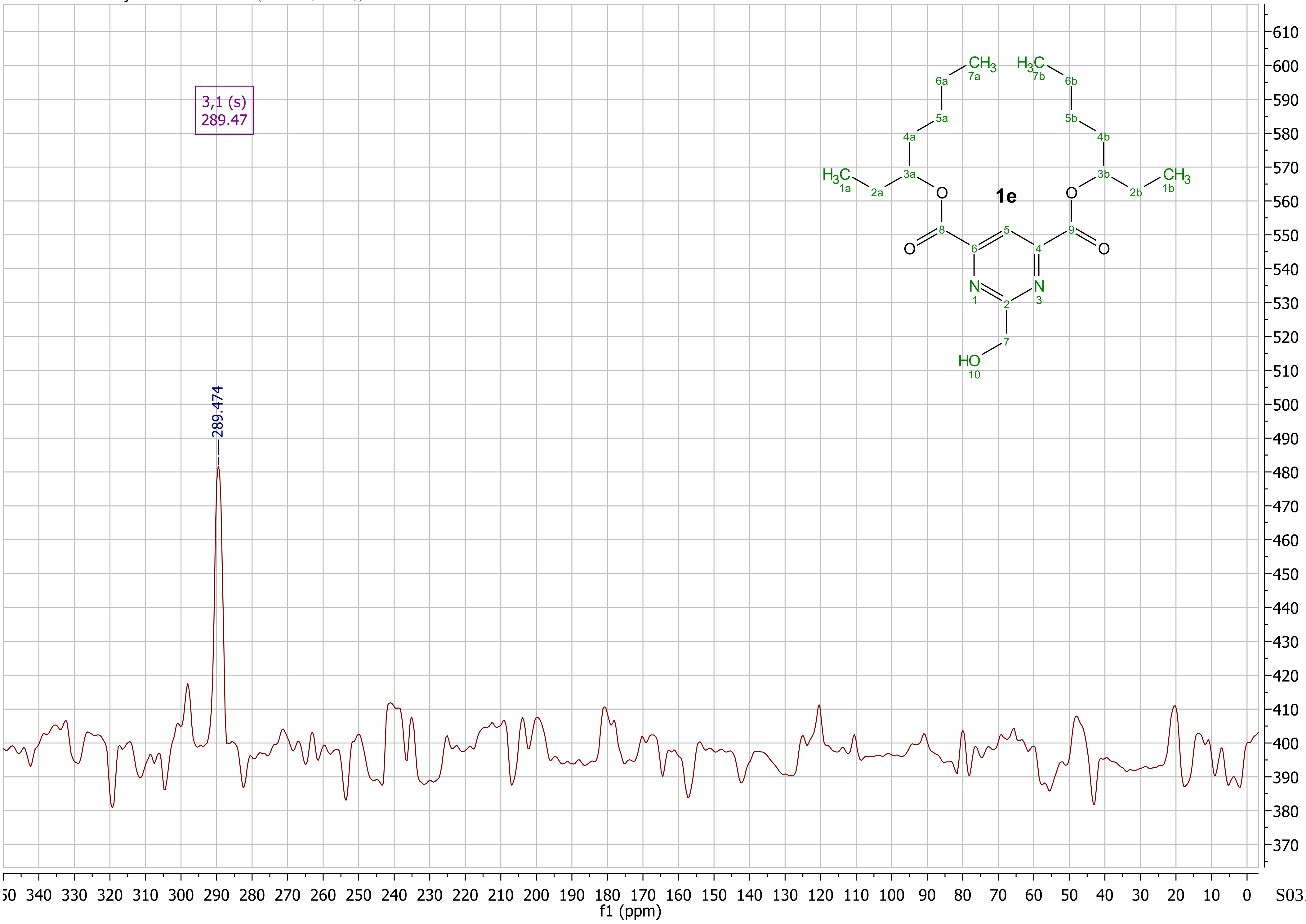

1H ^{1}H NMR (400 MHz, $CDCl_3$) δ 8.42 (app t, J = 0.7 Hz, 1H), 5.18 (app quint, J = 6.1 Hz, 2H), 5.03 (d, J = 0.6 Hz, 2H), 3.33 (s, 1H), 2.05 – 1.51 (m, 8H), 1.50 – 1.10 (m, 12H), 0.96 (t, J = 7.4 Hz, 6H), 0.87 (app t, J = 7.0 Hz, 6H).

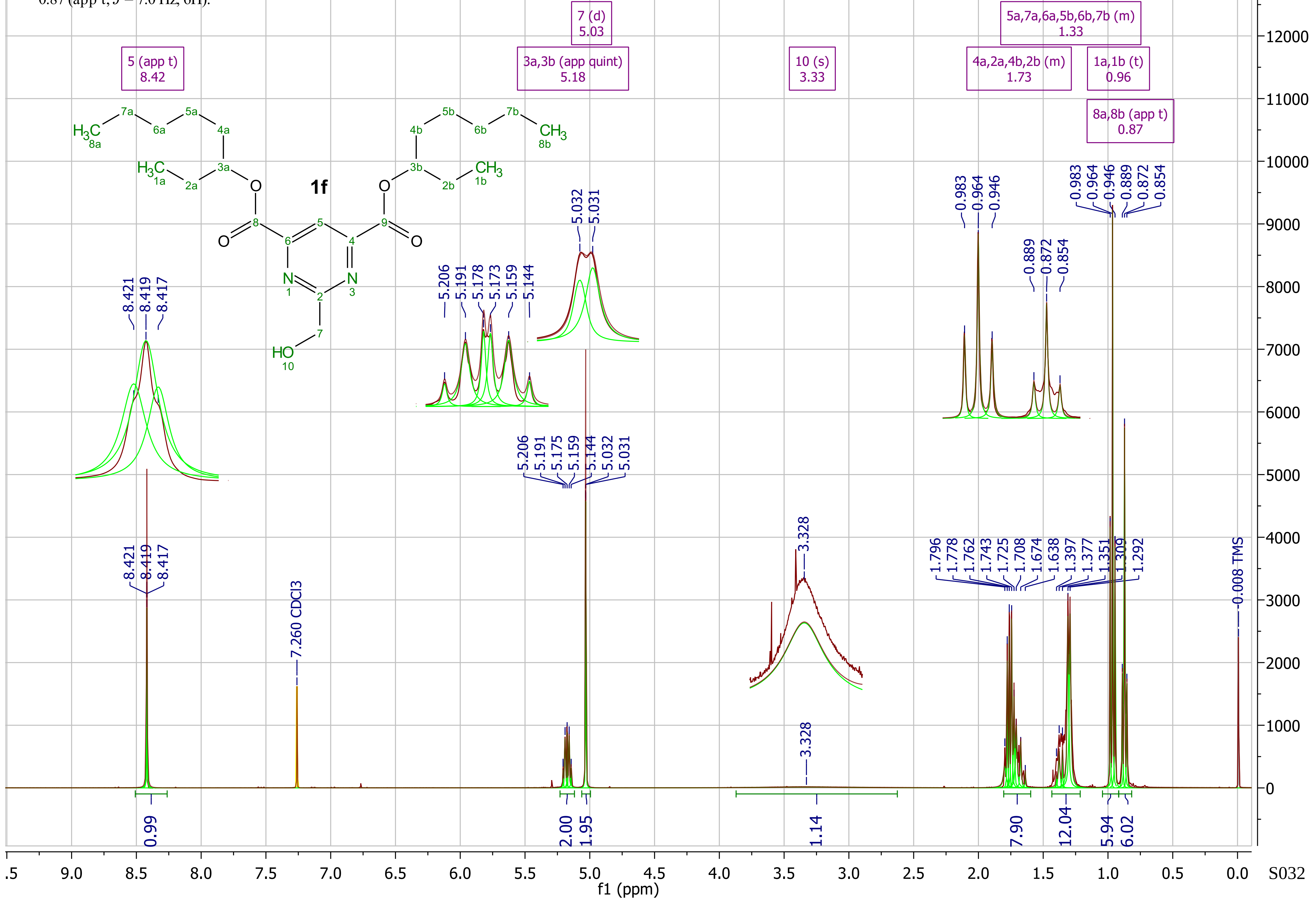

13C ^{13}C NMR (101 MHz, $CDCl_3$) δ 170.3, 163.3 (sym, 2C), 157.9 (sym, 2C), 118.6, 79.3 (sym, 2C), 64.9, 33.6 (sym, 2C), 31.7 (sym, 2C), 27.1 (sym, 2C), 25.1 (sym, 2C), 22.6 (sym, 2C), 14.1 (sym, 2C), 9.8 (sym, 2C).

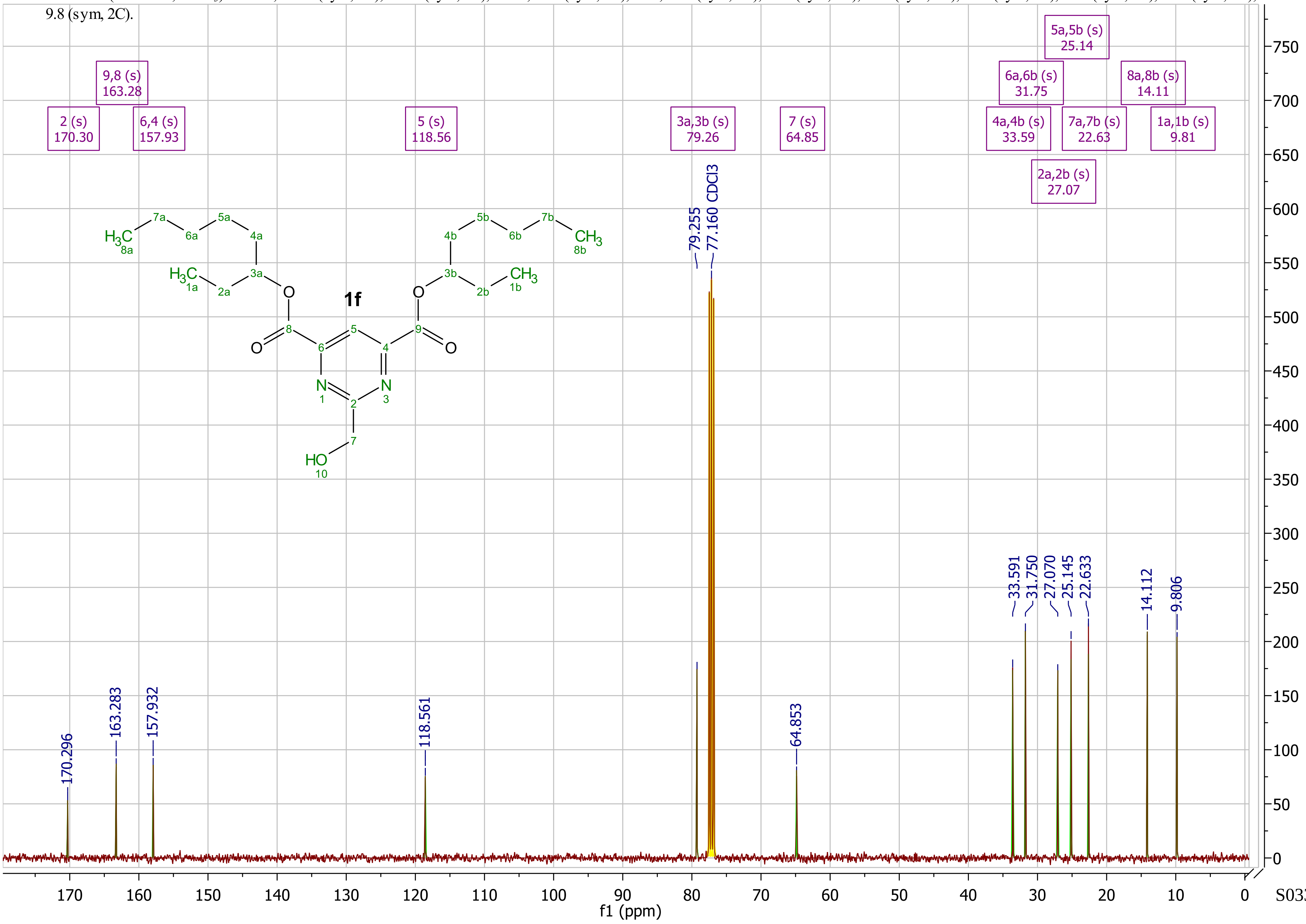

13C HSQC

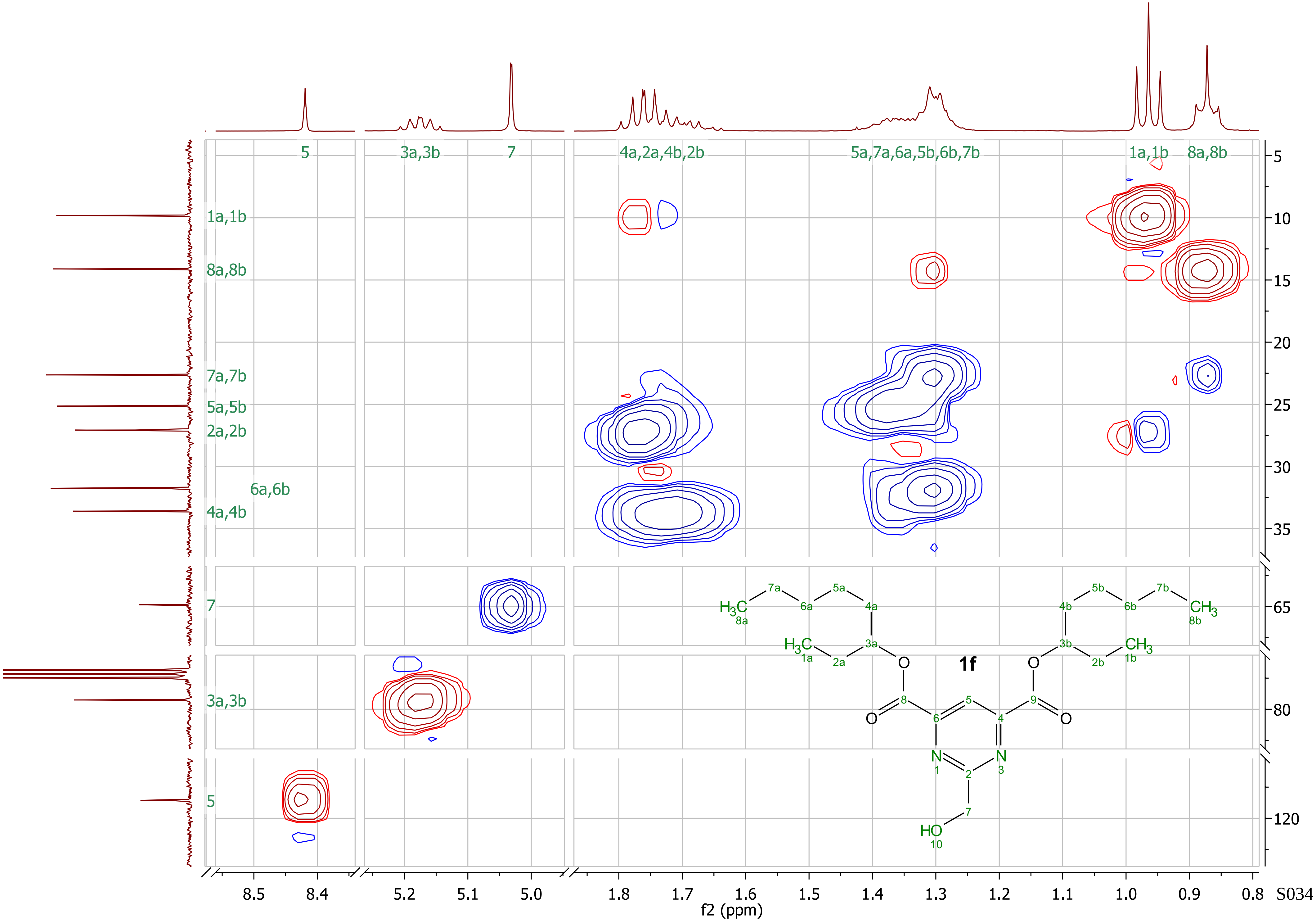

S034

13C HMBC

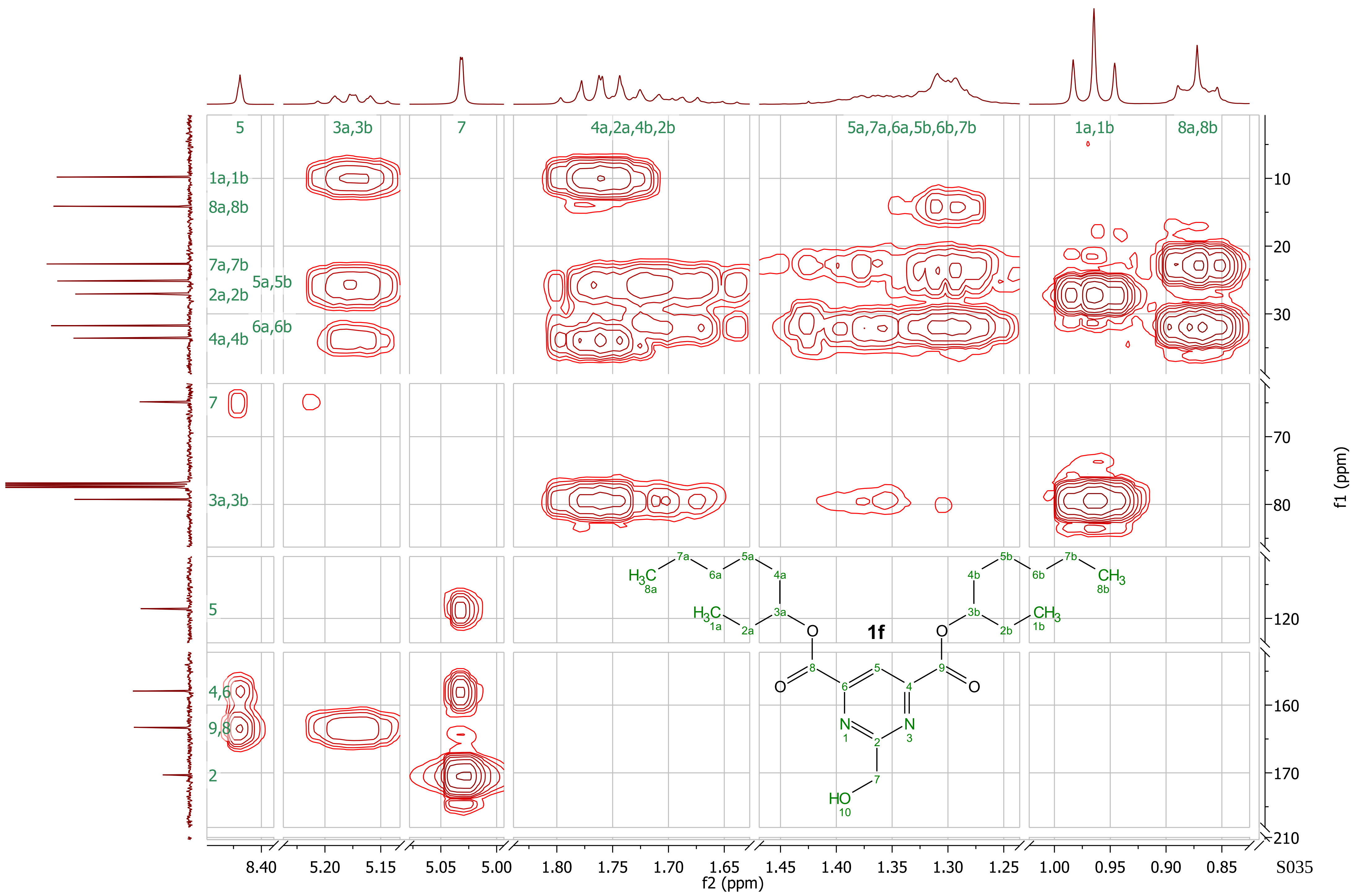

5
3a,3b
7
4a,2a,4b,2b
5a,7a,6a,5b,6b,7b
1a,1b
8a,8b
7a,7b
5a,5b
2a,2b
6a,6b
4a,4b
3a,3b
4,6
9,8
2
1f
f2 (ppm)
f1 (ppm)
S035

15N HMBC

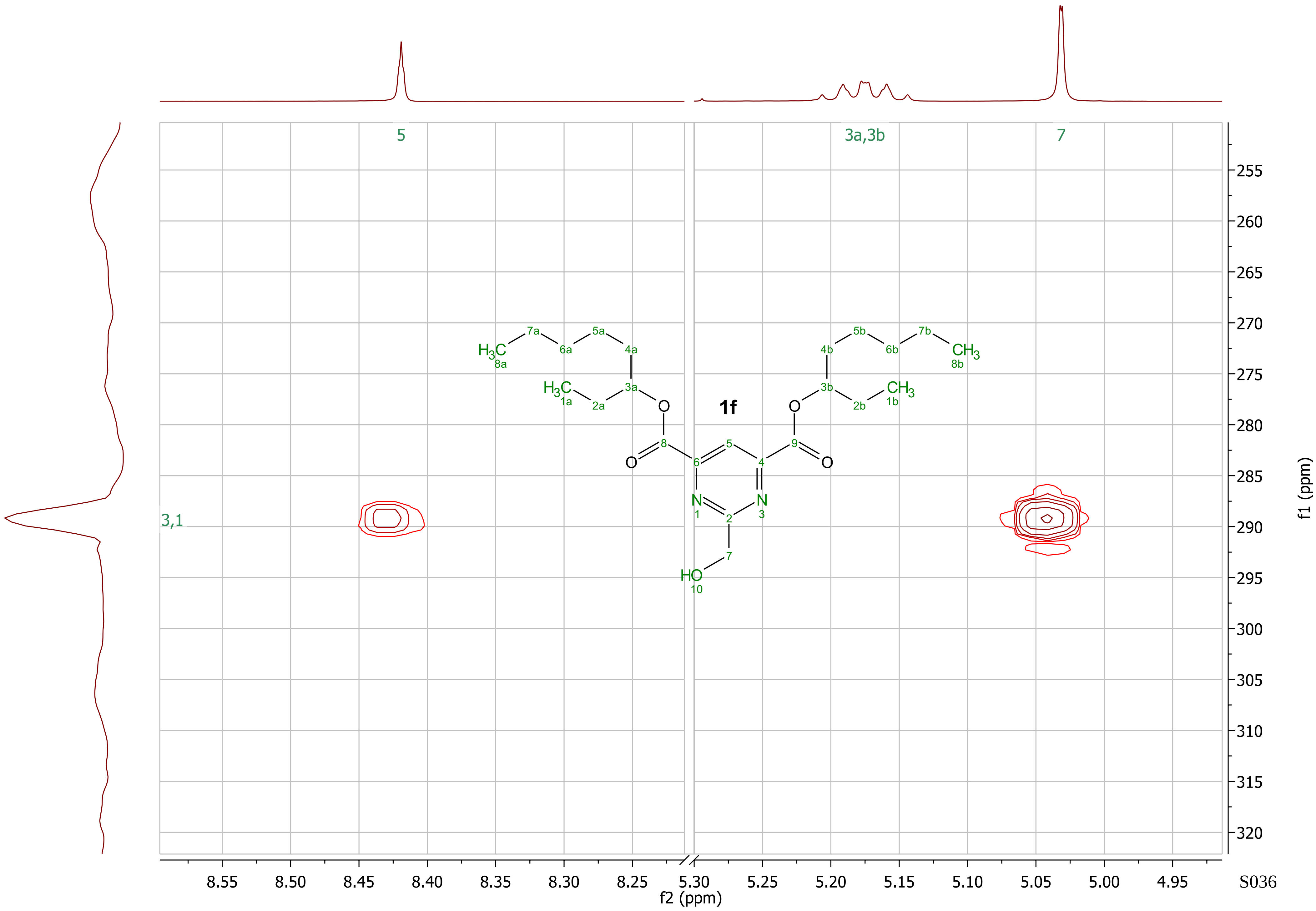

S036

15N HMBC f1 Projection ^{15}N NMR (41 MHz, $CDCl_3$) δ 289.28.

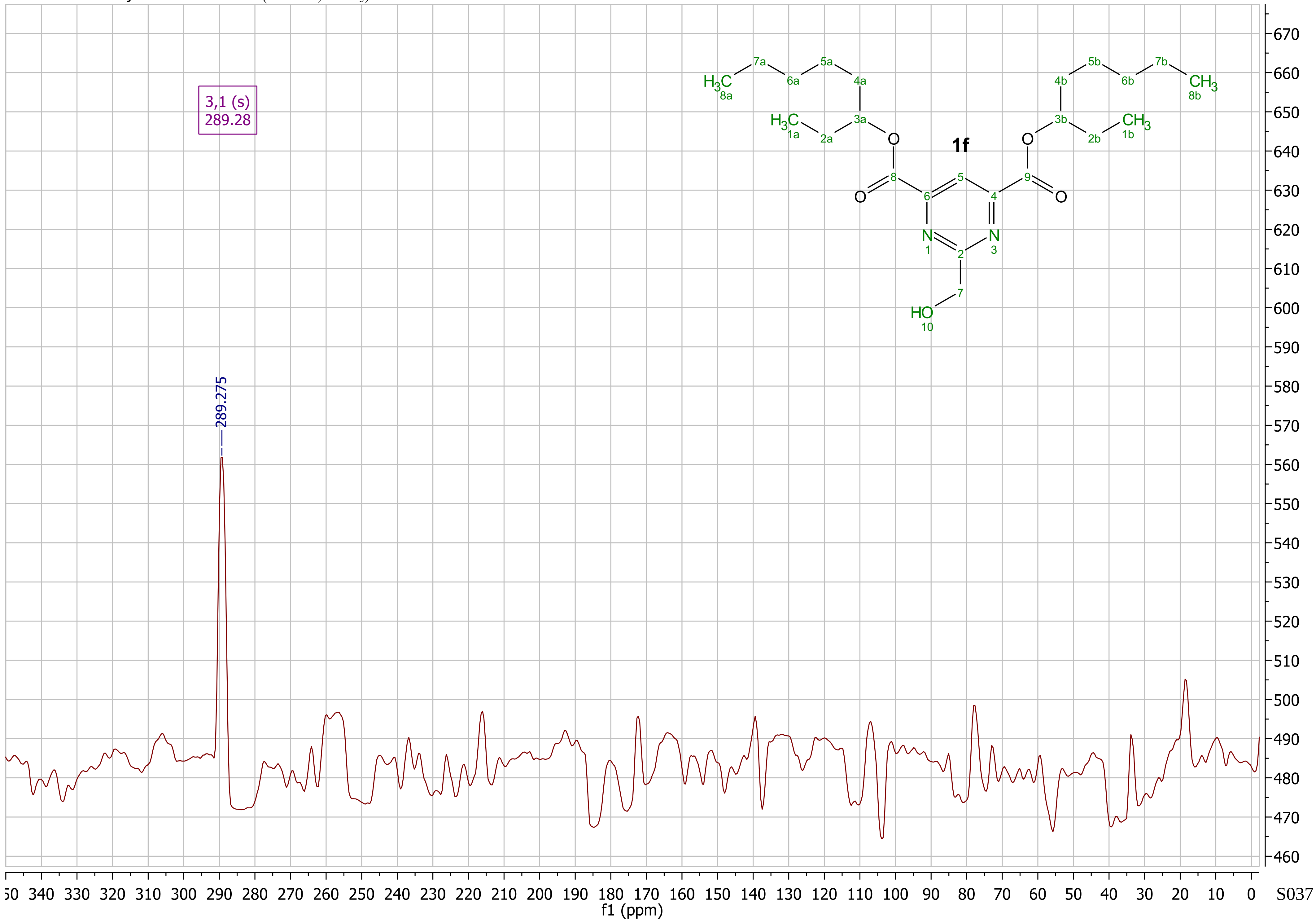

1H ^{1}H NMR (400 MHz, $CDCl_3$) δ 8.50 (app t, *J* = 0.7 Hz, 1H), 7.72 (s, 2H), 7.66 (d, *J* = 7.6 Hz, 2H), 7.63 (d, *J* = 7.9 Hz, 2H), 7.53 (t, *J* = 7.7 Hz, 2H), 5.51 (s, 4H), 5.03 (s, 2H), 3.60 (br s, 1H).

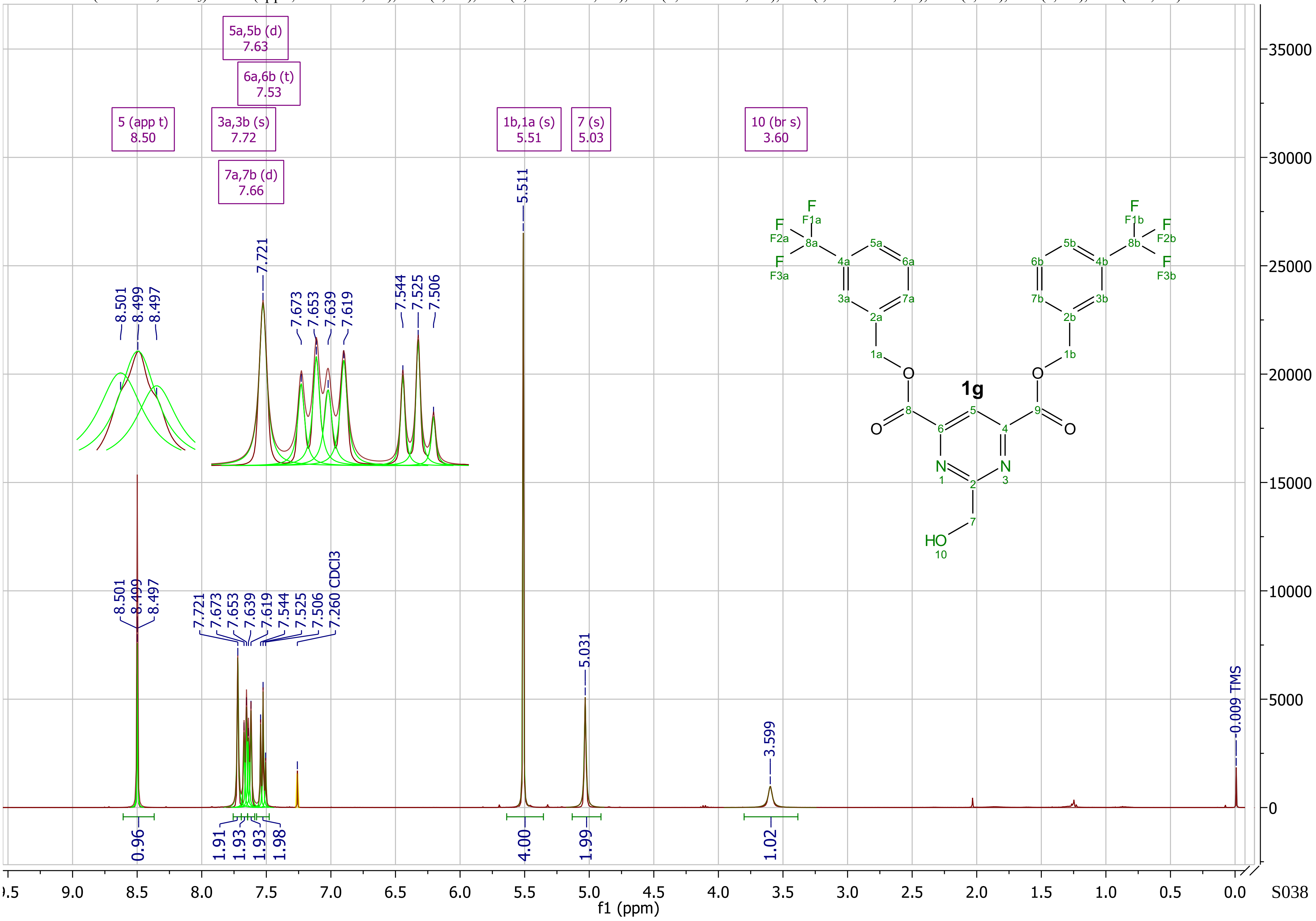

^{13}C NMR (101 MHz, CDCl$_3$) δ 170.7, 163.0 (sym, 2C), 157.2 (sym, 2C), 135.6 (sym, 2C), 132.1 (app q, *J* = 1.1 Hz, sym, 2C), 131.4 (q, *J* = 32.6 Hz, sym, 2C), 129.5 (sym, 2C), 125.9 (q, *J* = 3.7 Hz, sym, 2C), 125.6 (q, *J* = 3.9 Hz, sym, 2C), 123.9 (q, *J* = 272.4 Hz, sym, 2C), 119.0, 67.7 (sym, 2C), 64.9.

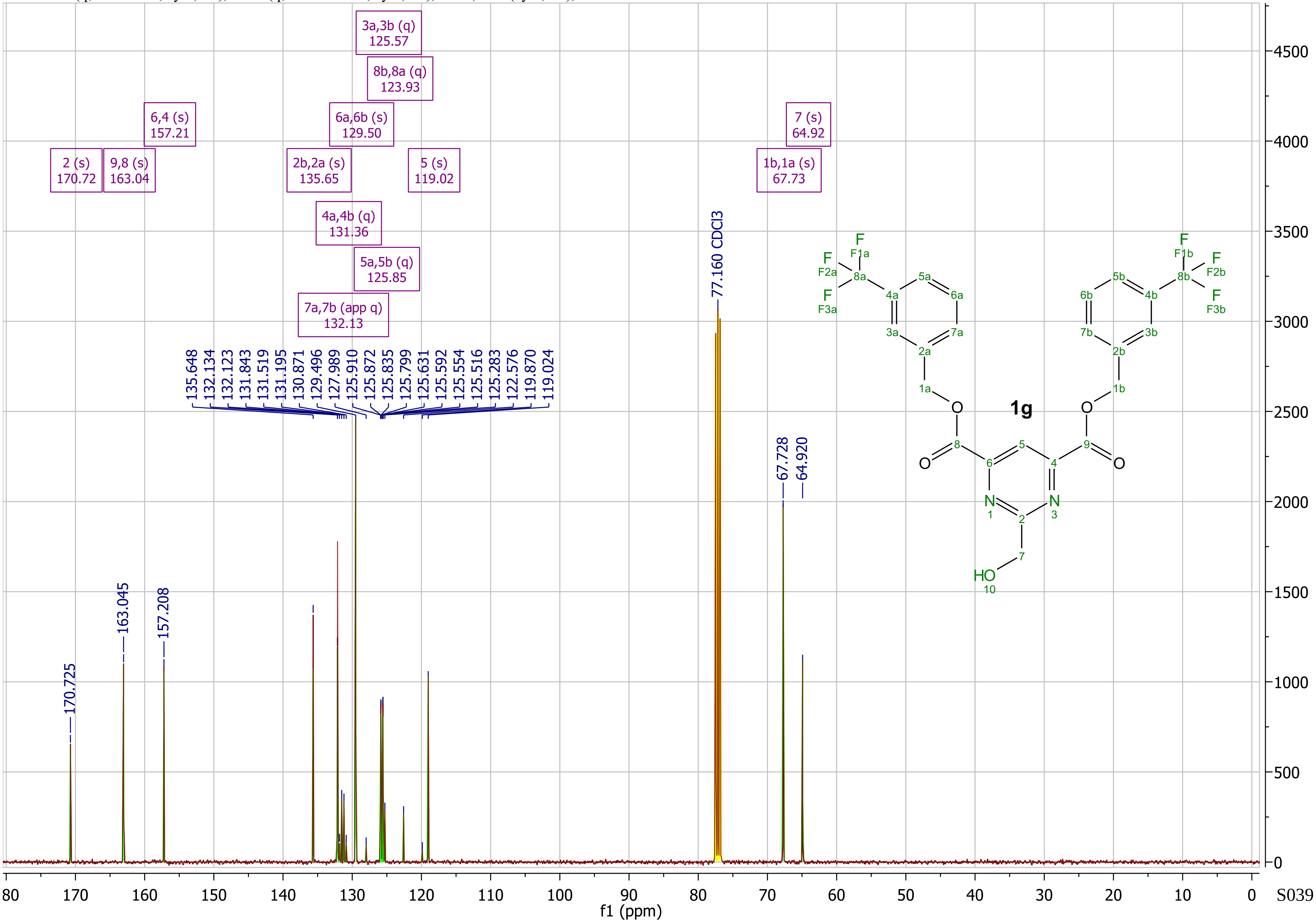

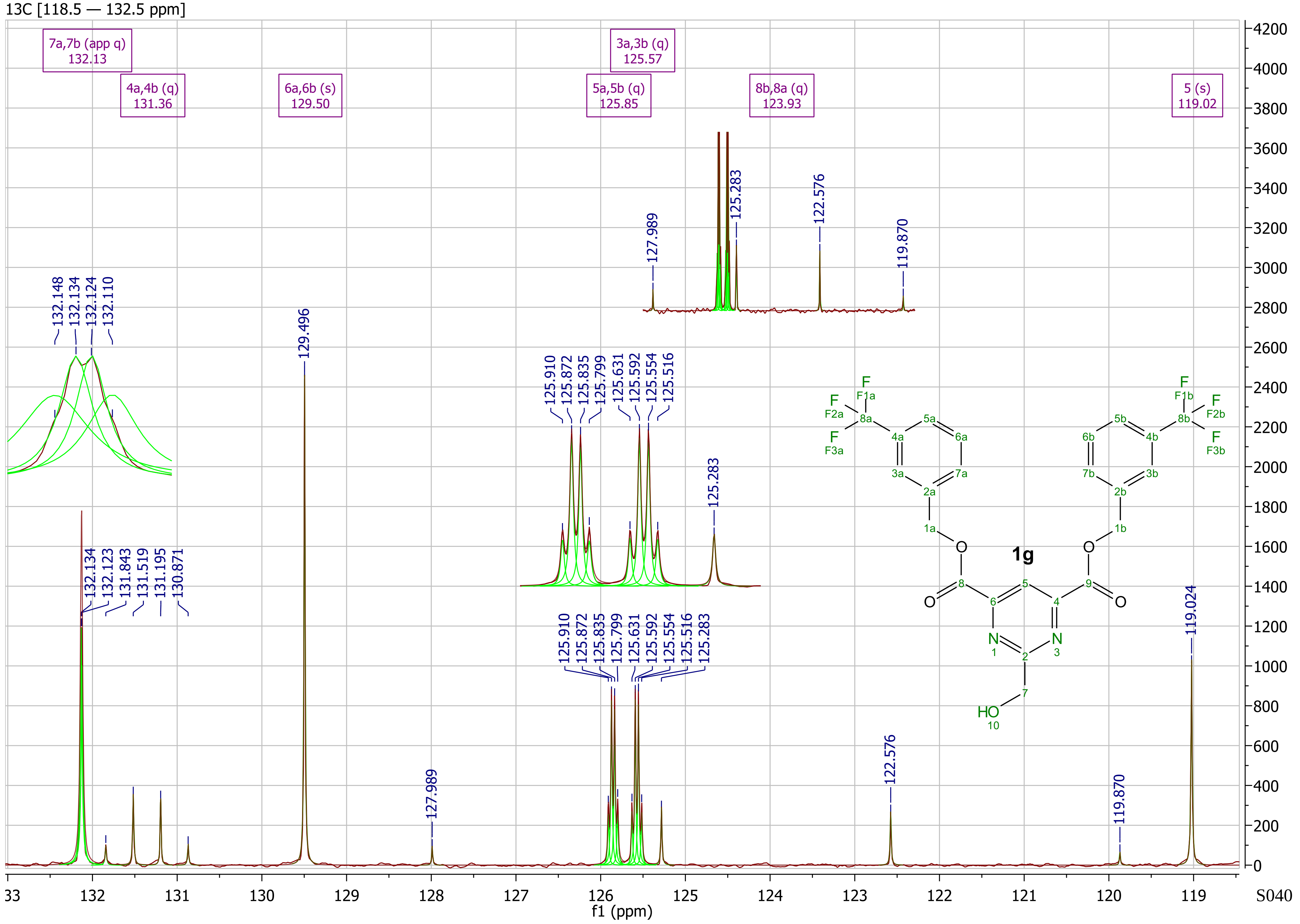

13C [118.5 — 132.5 ppm]
7a,7b (app q)
132.13
4a,4b (q)
131.36
6a,6b (s)
129.50
5a,5b (q)
125.85
3a,3b (q)
125.57
8b,8a (q)
123.93
5 (s)
119.02
132.148
132.134
132.124
132.110
129.496
125.910
125.872
125.835
125.799
125.631
125.592
125.554
125.516
125.283
127.989
122.576
119.870
132.134
132.123
131.843
131.519
131.195
130.871
119.024
1g
HO
f1 (ppm)
S040

13C HSQC

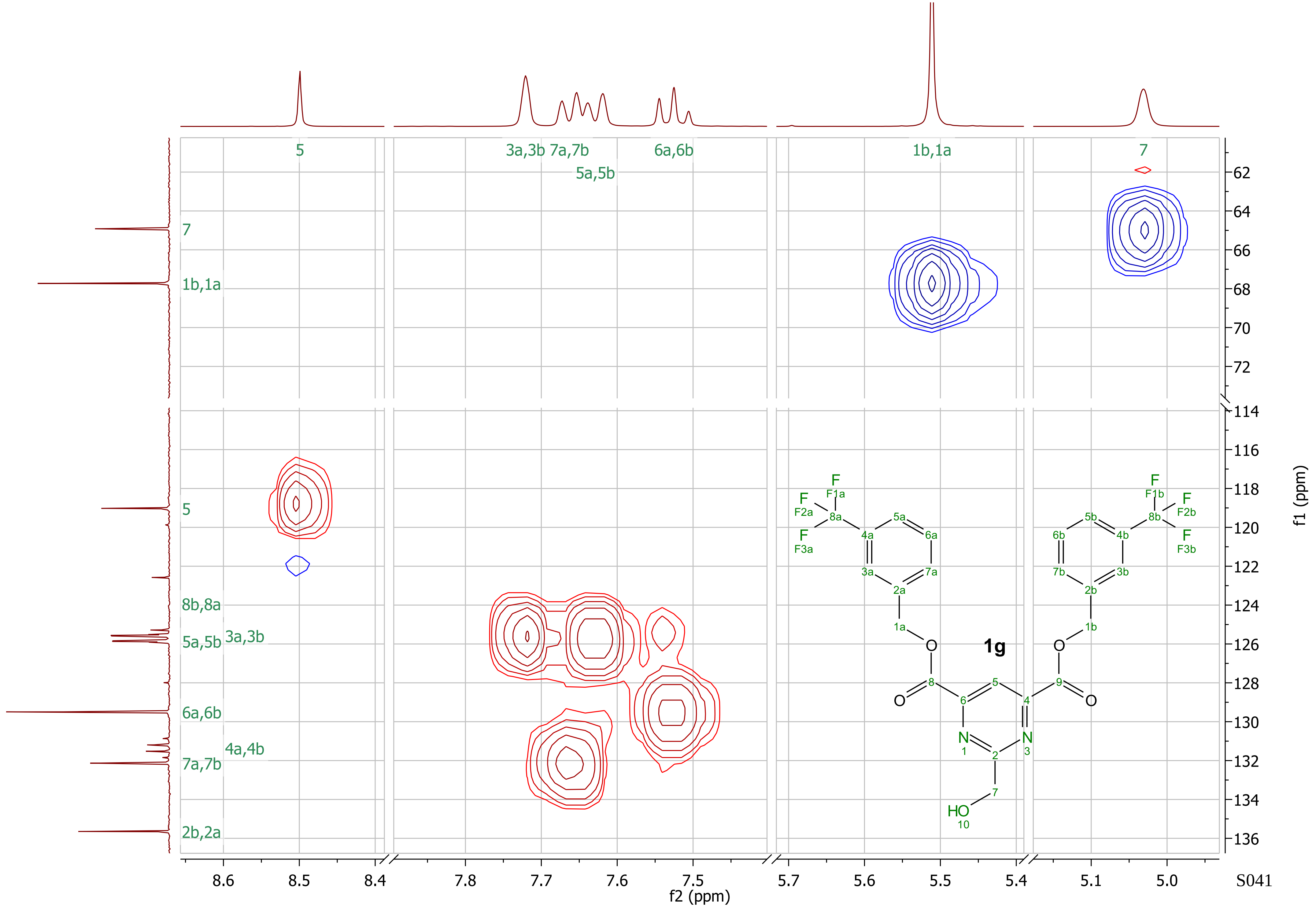

5
3a,3b 7a,7b
5a,5b
6a,6b
1b,1a
7
1b,1a
5
8b,8a
5a,5b 3a,3b
6a,6b
4a,4b
7a,7b
2b,2a
1g
HO
f2 (ppm)
f1 (ppm)

13C HMBC

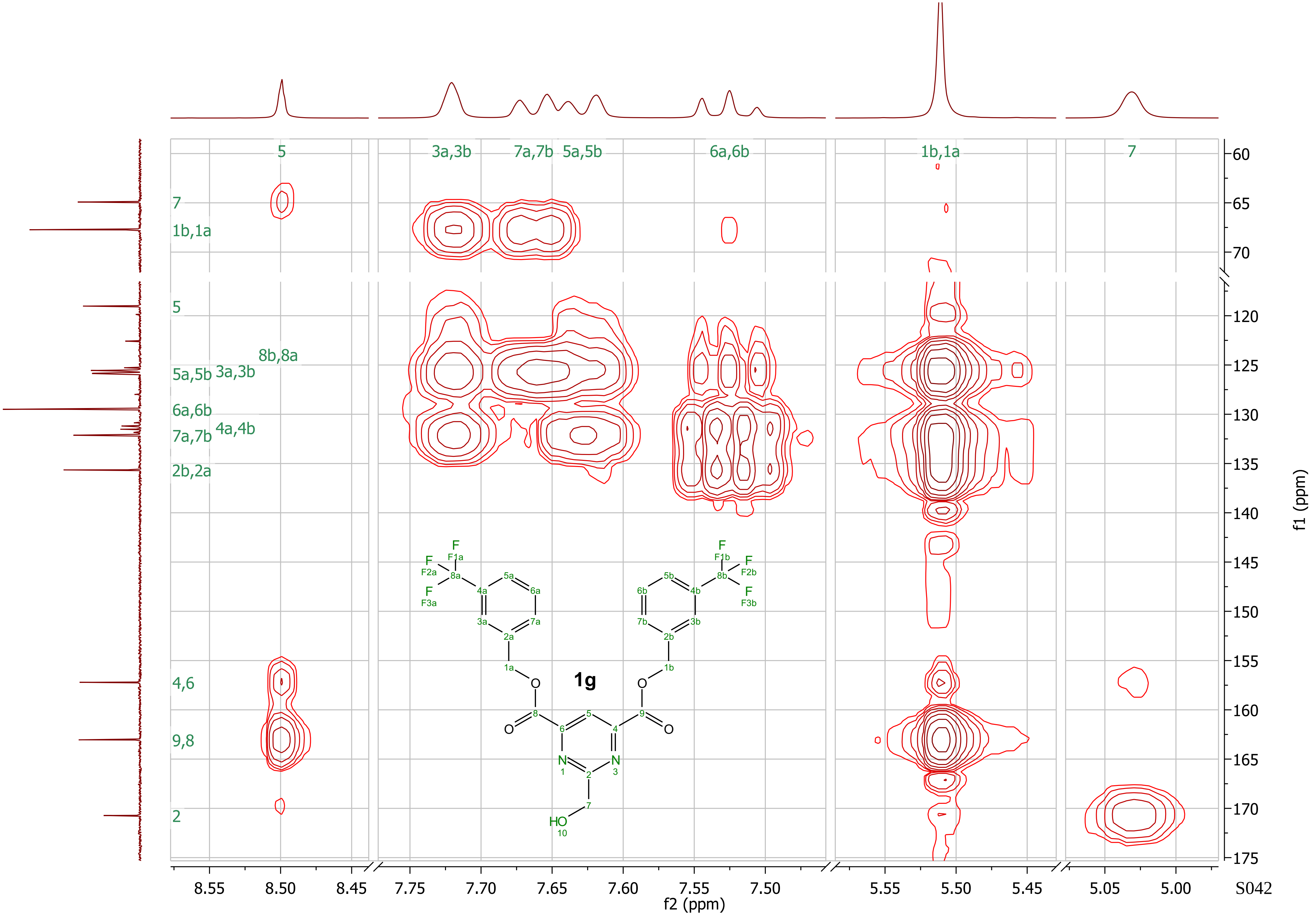

S042

15N HMBC ^{15}N NMR (41 MHz, $CDCl_3$) δ 290.66.

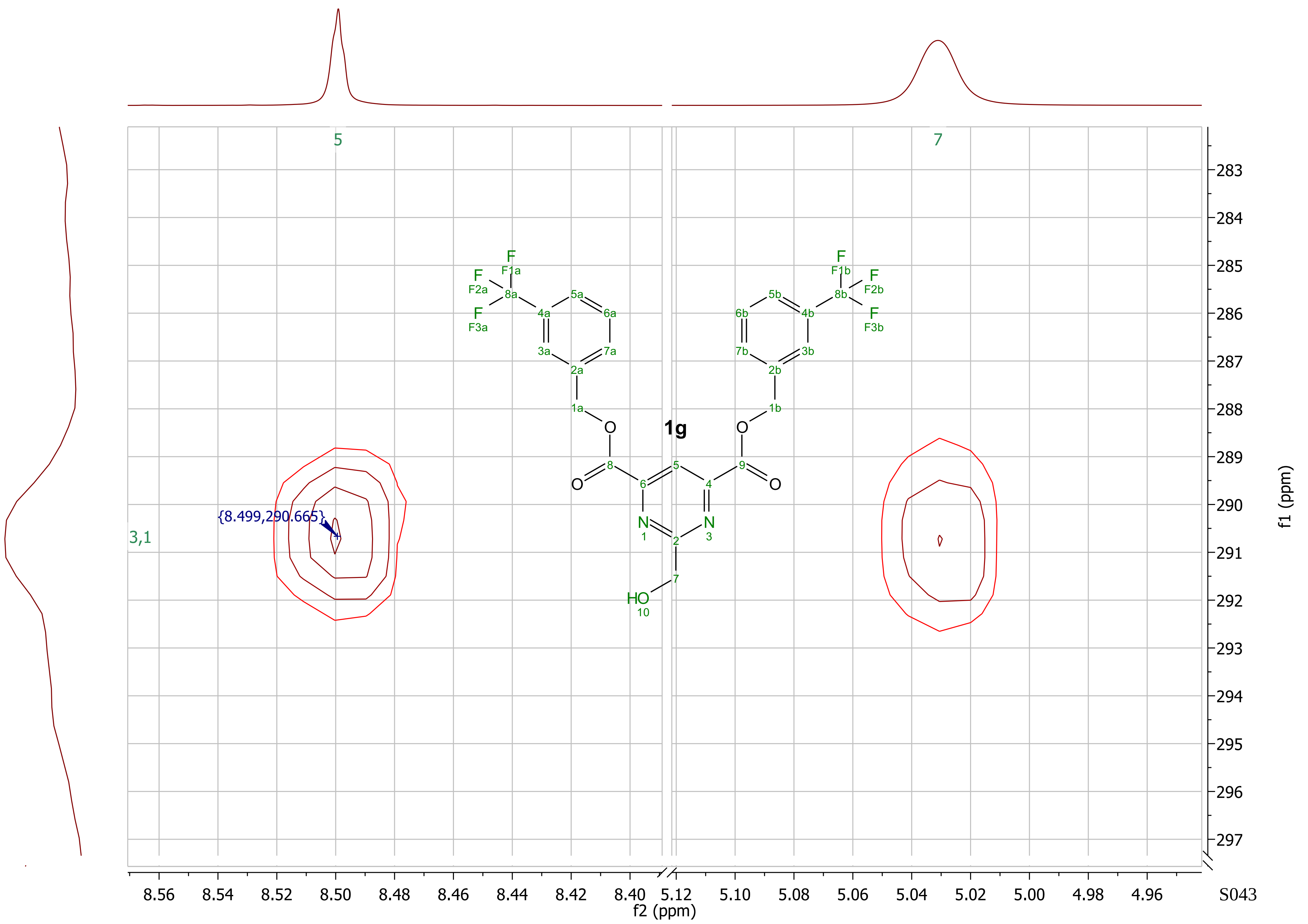

S043

19F ^{19}F NMR (376 MHz, $CDCl_3$) δ -62.73.

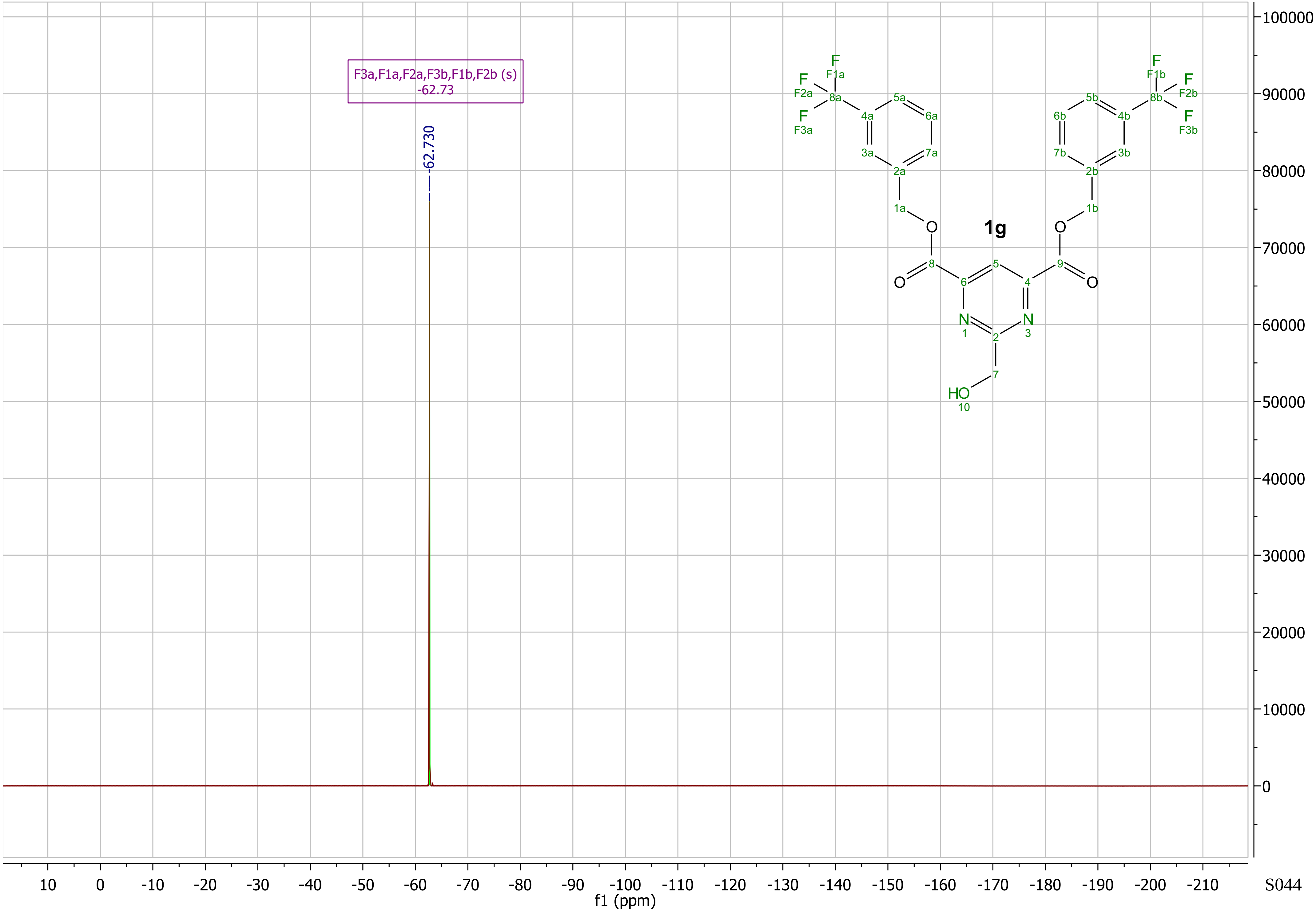

1H ^{1}H NMR (400 MHz, $CDCl_3$) δ 8.49 (app q, *J* = 0.7 Hz, 1H), 5.04 (s, 2H), 4.52 (q, *J* = 7.1 Hz, 4H), 3.61 (s, 1H), 1.46 (t, *J* = 7.1 Hz, 6H).

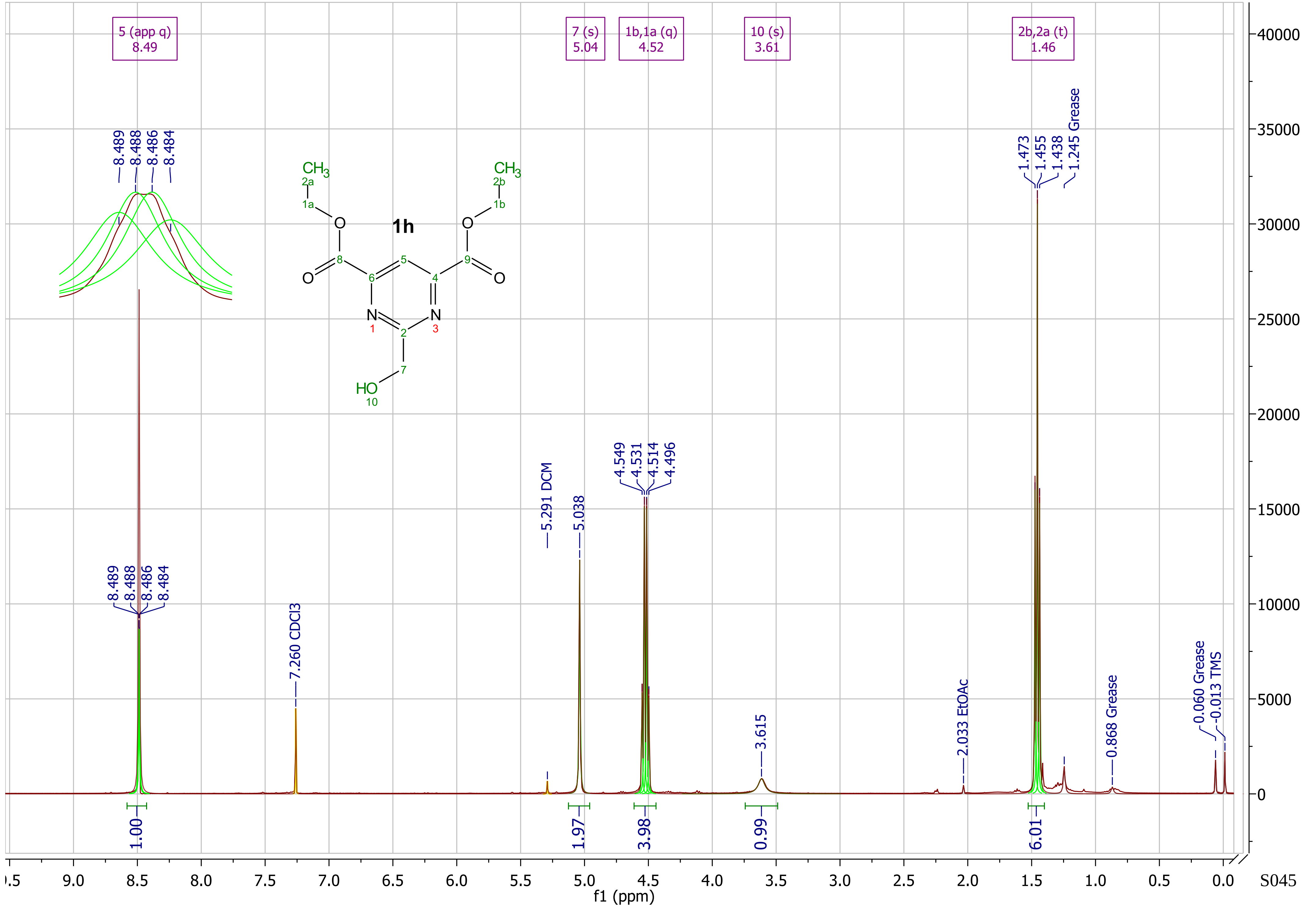

S045

13C ^{13}C NMR (101 MHz, $CDCl_3$) δ 170.4, 163.4 (sym, 2C), 157.6 (sym, 2C), 118.8, 64.9, 63.2 (sym, 2C), 14.3 (sym, 2C).

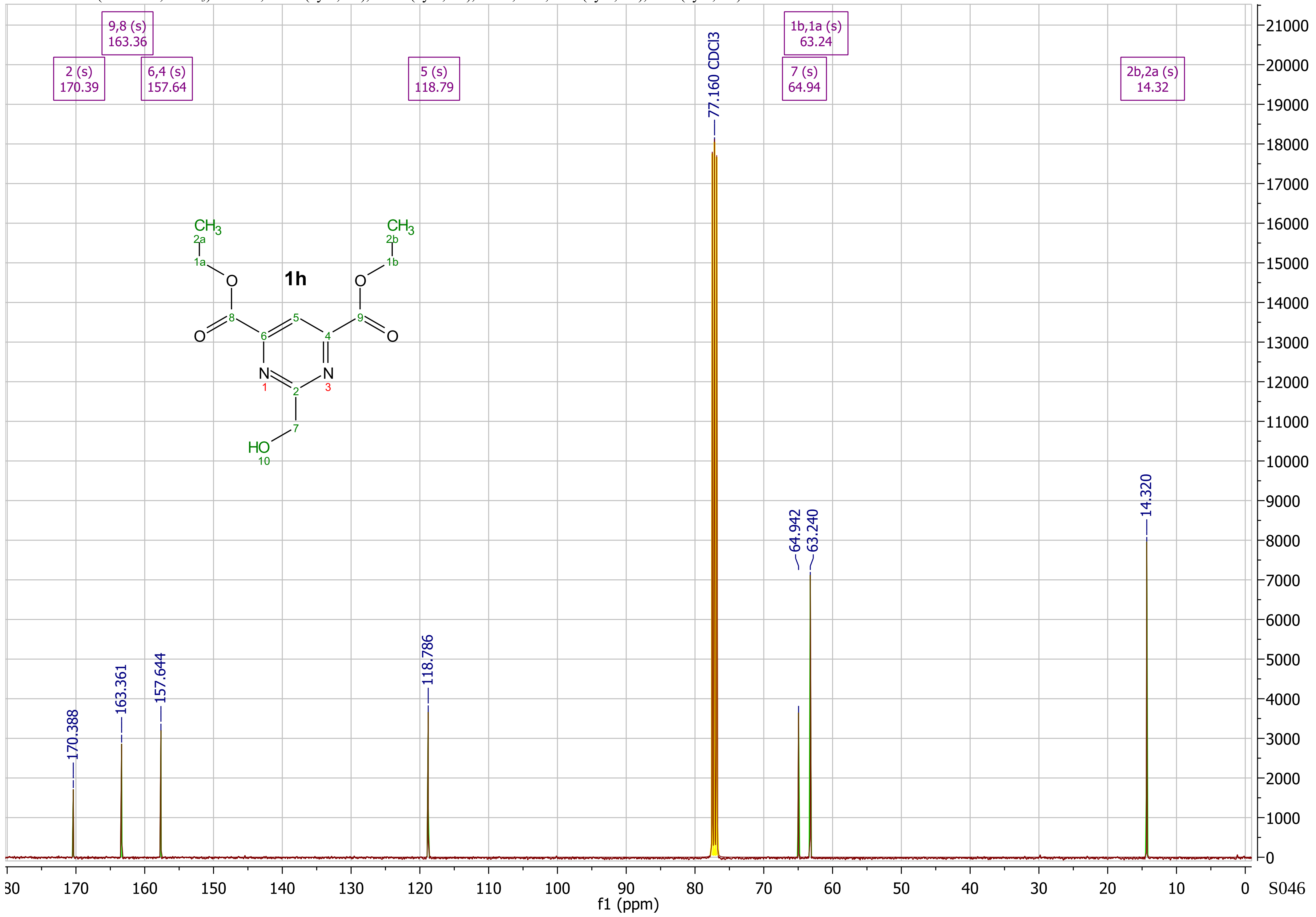

S046

13C HSQC

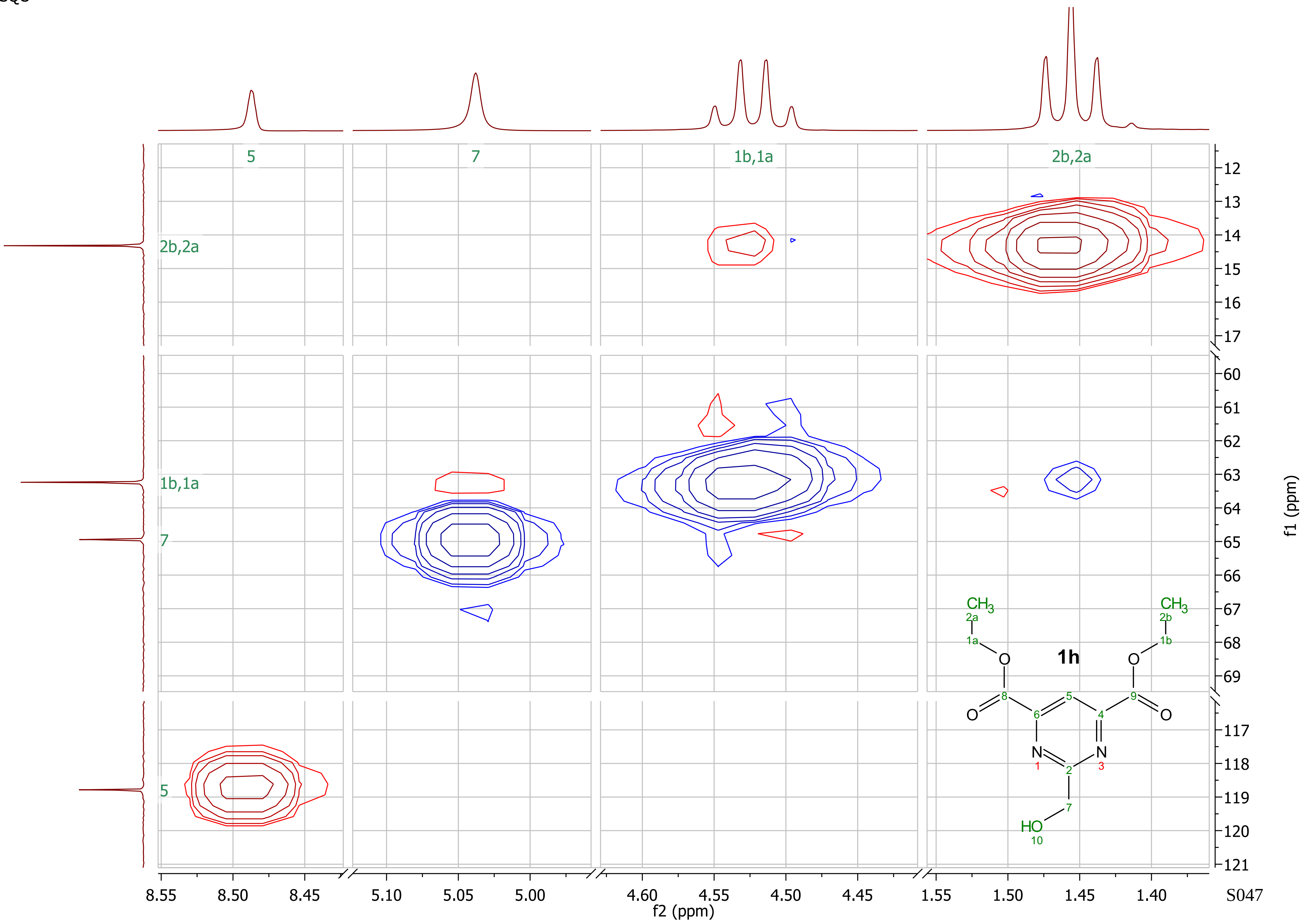

S047

13C HMBC

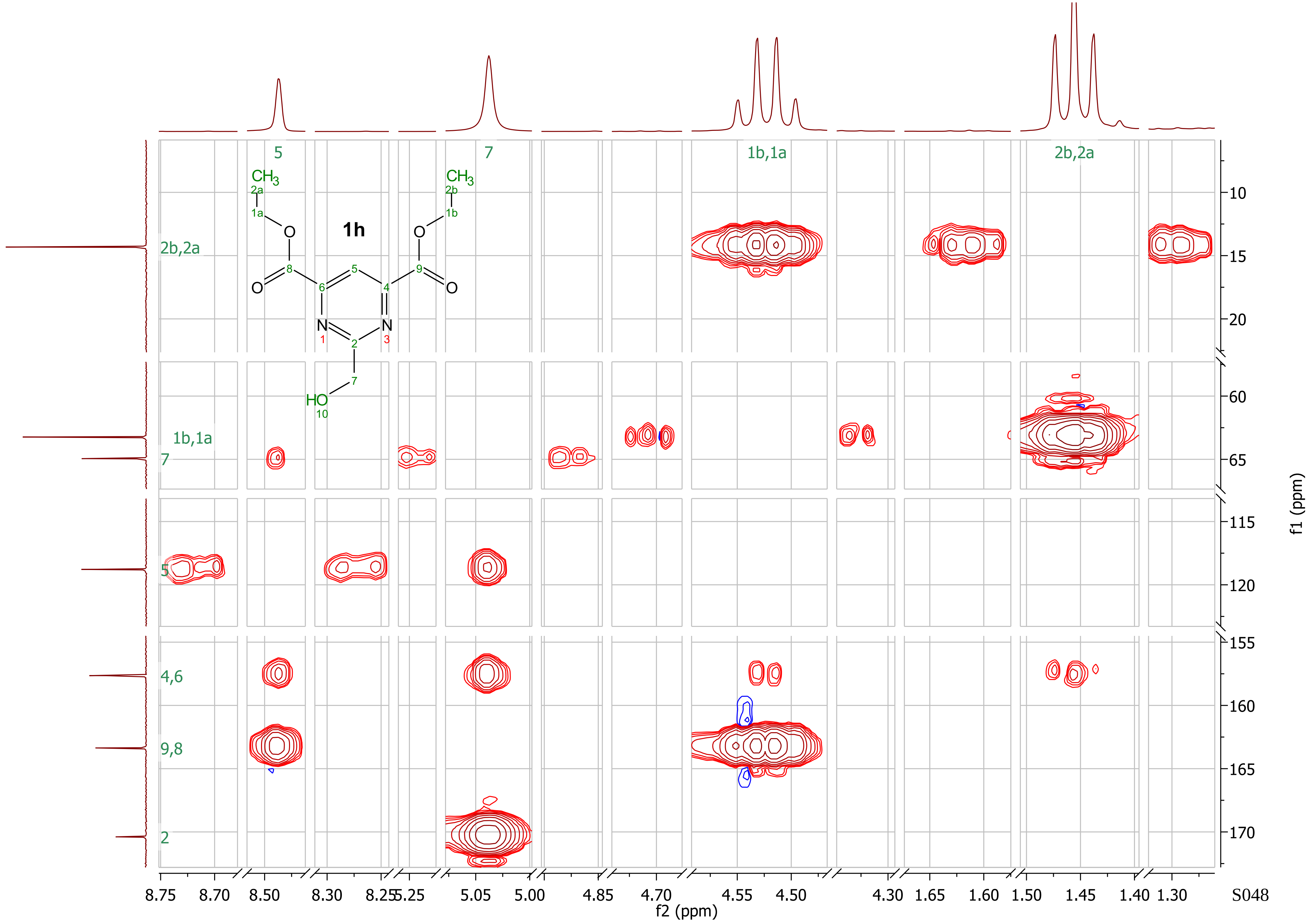

S048

1H ^{1}H NMR (400 MHz, $CDCl_3$) δ ^{1}H NMR (400 MHz, $CDCl_3$) δ 4.69 (s, 2H), 4.40 (t, J = 5.9 Hz, 2H), 4.37 (t, J = 6.1 Hz, 2H), 3.40 (br s, 1H), 2.28 (s, 3H), 1.85 – 1.65 (m, 4H), 1.49 – 1.38 (m, 4H), 1.37 – 1.30 (m, 8H), 1.02 – 0.74 (m, 6H).

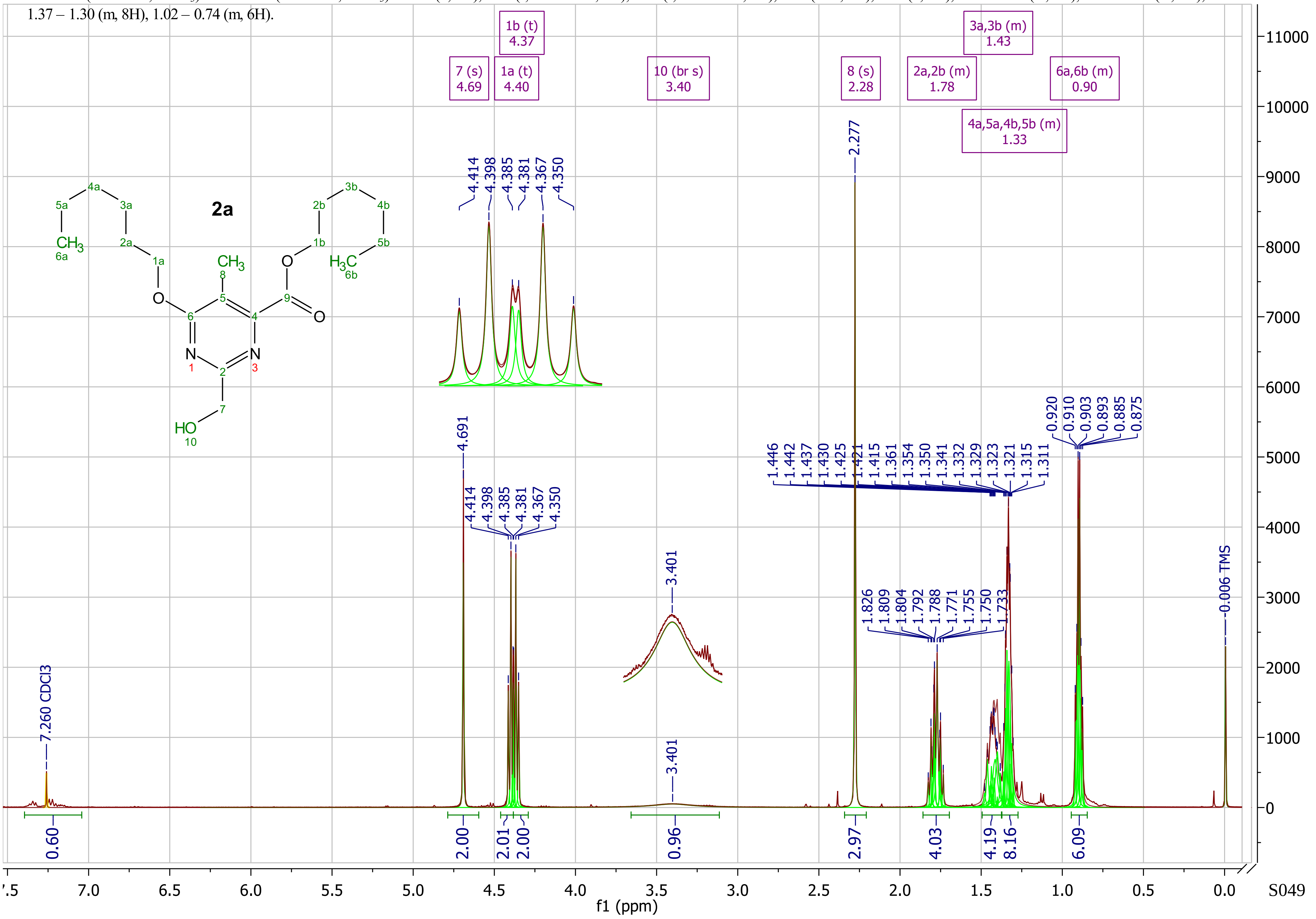

13C ^{13}C NMR (101 MHz, $CDCl_3$) δ 169.2, 165.8, 165.3, 154.5, 116.3, 67.7, 66.4, 64.2, 31.6, 31.5, 28.7, 28.6, 25.8, 25.7, 22.69, 22.65, 14.13, 14.11, 11.1.

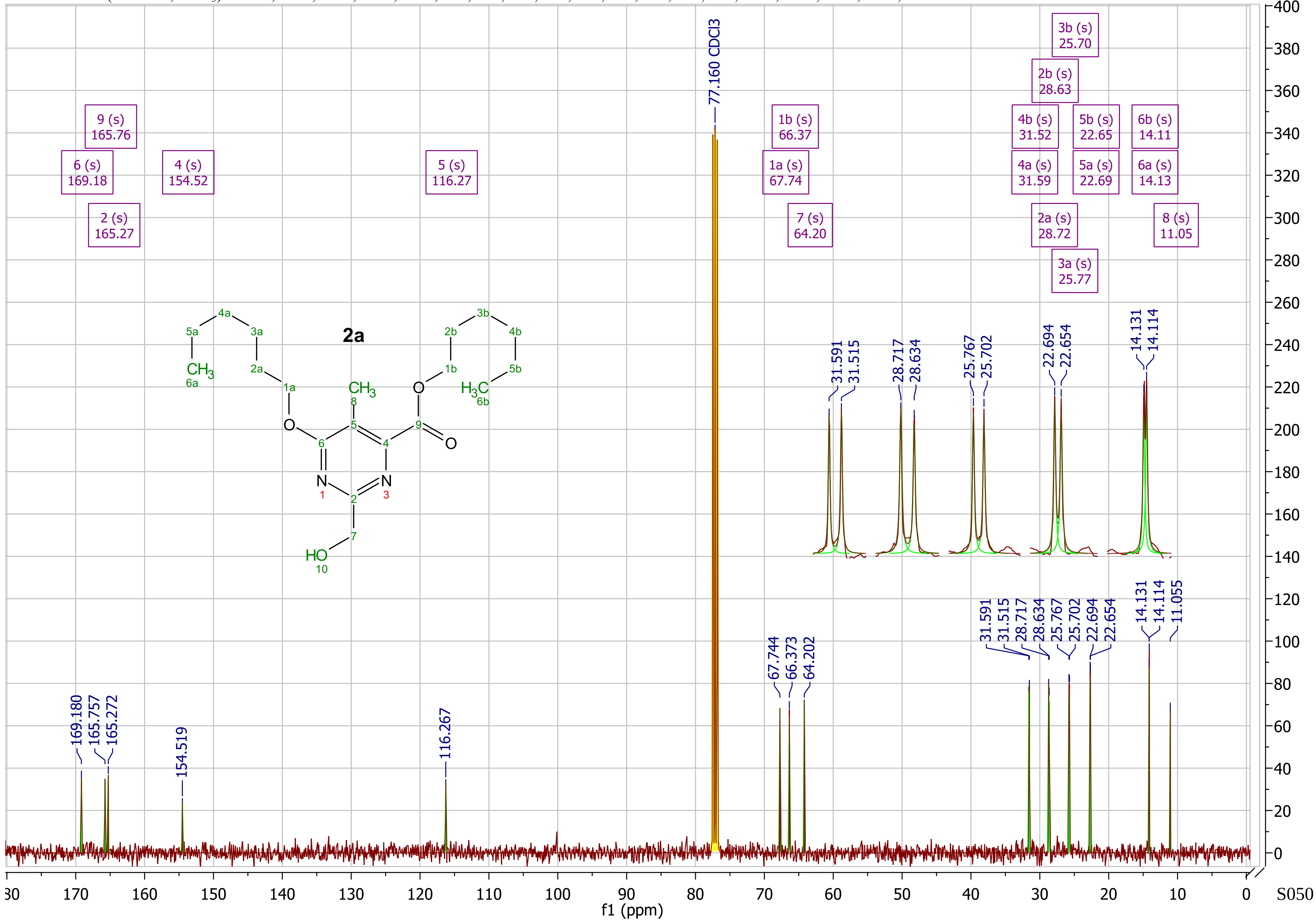

S050

13C HSQC

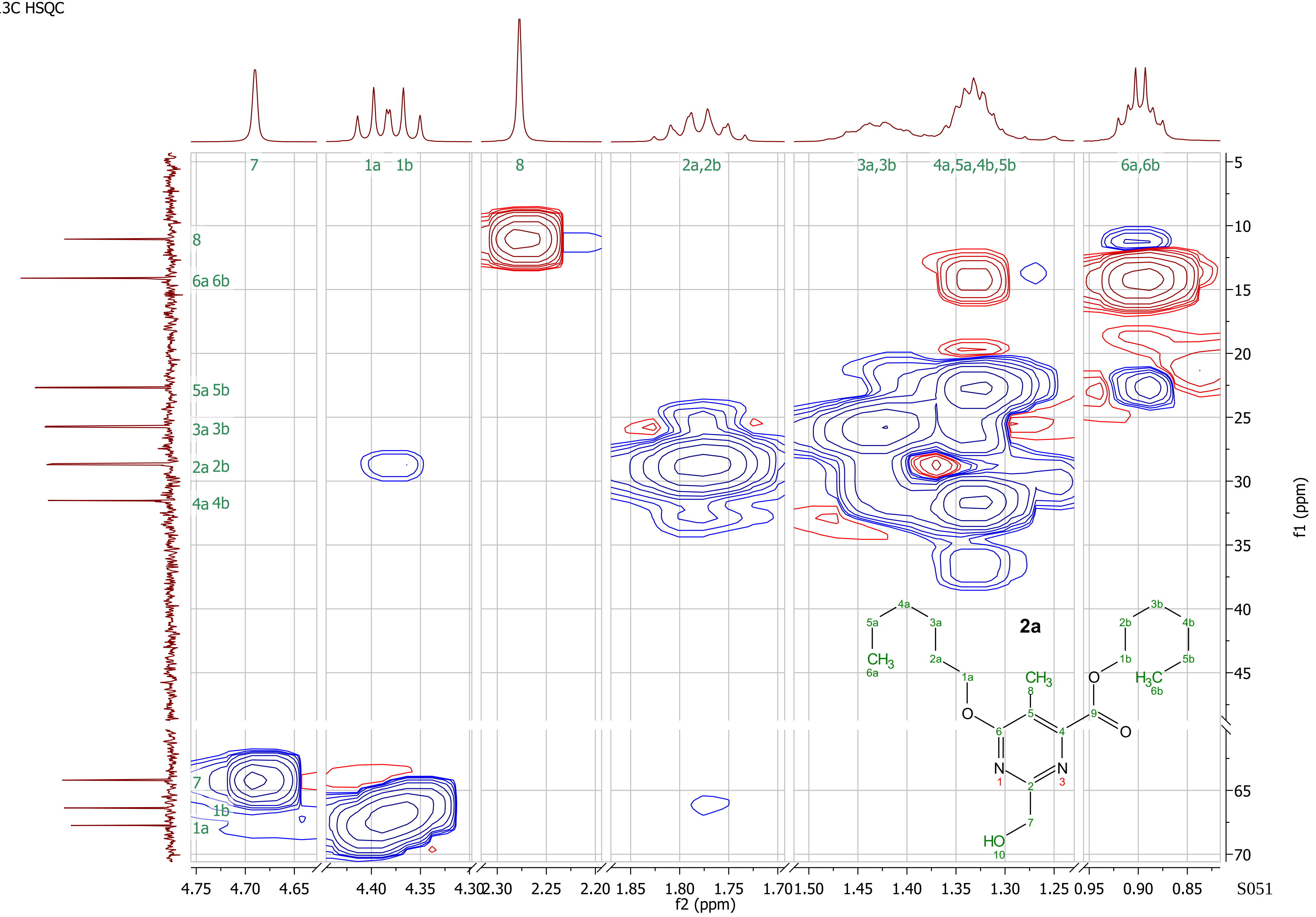

7
1a
1b
8
2a,2b
3a,3b
4a,5a,4b,5b
6a,6b
8
6a 6b
5a 5b
3a 3b
2a 2b
4a 4b
7
1b
1a
2a
f2 (ppm)
f1 (ppm)

13C HMBC

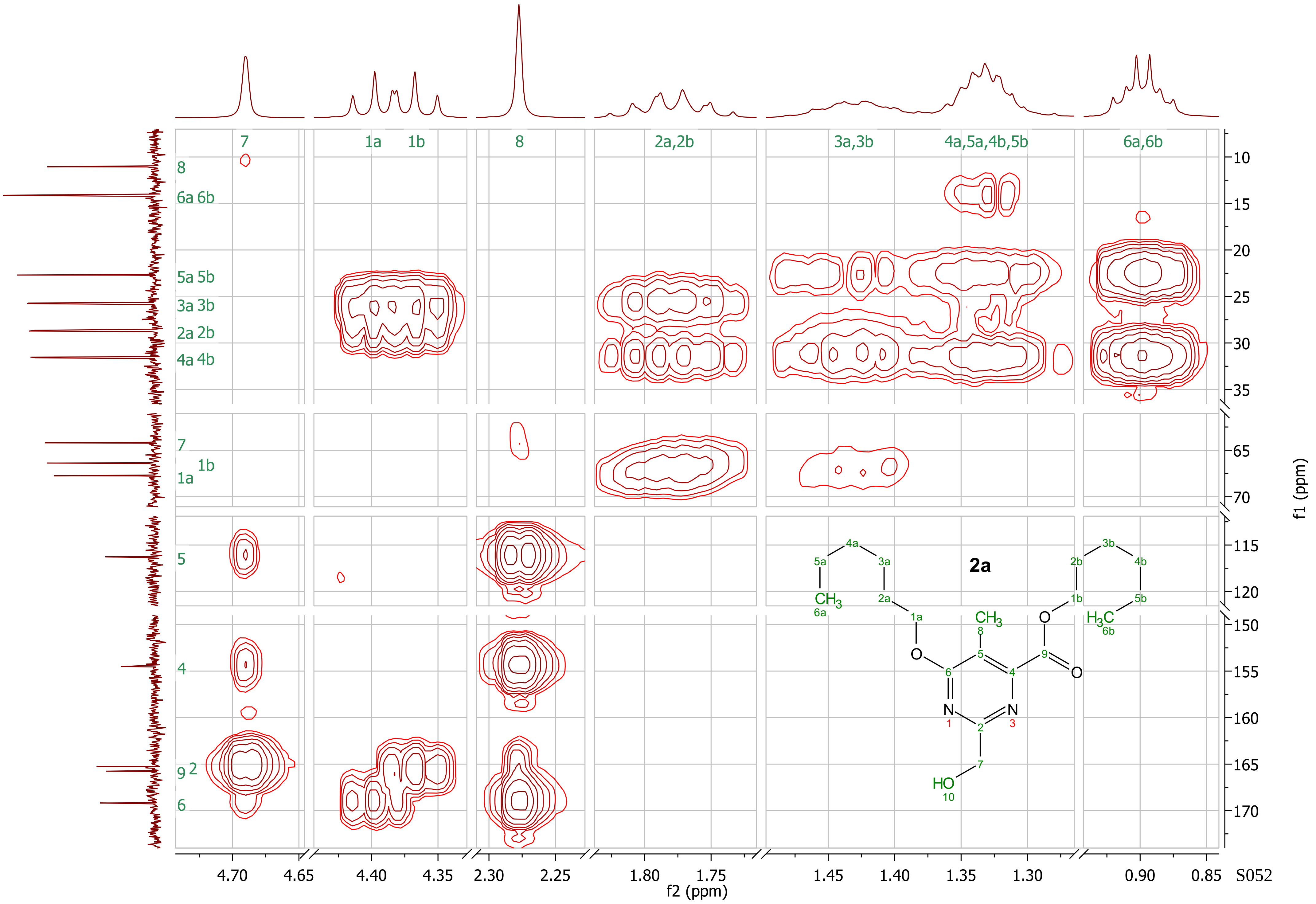

S052

1H ^{1}H NMR (400 MHz, $CDCl_3$) δ 4.69 (s, 2H), 4.39 (t, J = 6.1 Hz, 2H), 4.36 (t, J = 6.3 Hz, 2H), 3.55 (br s, 1H), 2.27 (s, 3H), 1.91 – 1.65 (m, 4H), 1.52 – 1.36 (m, 4H), 1.38 – 1.17 (m, 12H), 0.89 (app t, J = 6.7 Hz, 3H), 0.88 (app t, J = 6.7 Hz, 3H).

2b

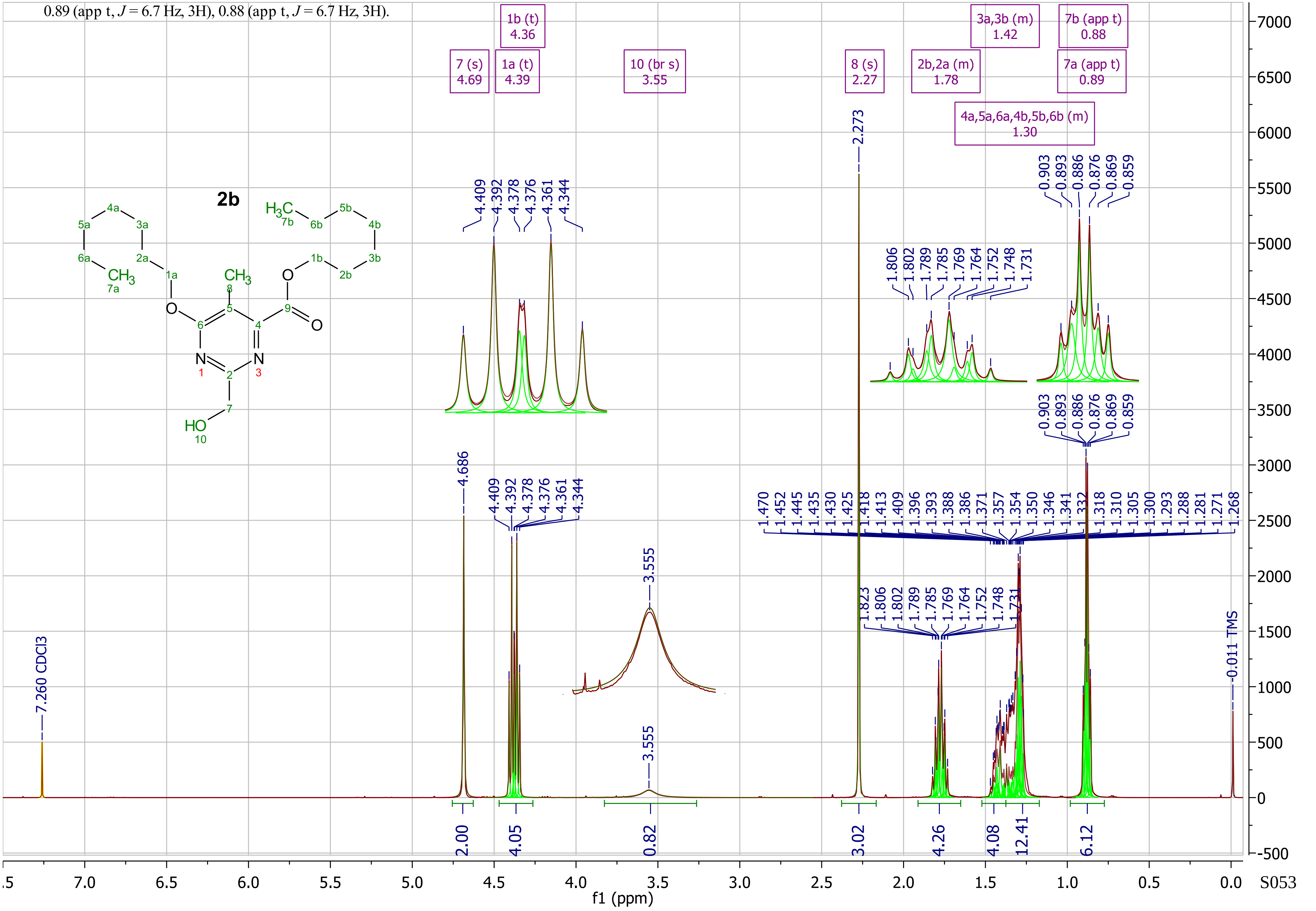

S053

13C ^{13}C NMR (101 MHz, $CDCl_3$) δ 169.2, 165.7, 165.3, 154.5, 116.3, 67.7, 66.4, 64.2, 31.9, 31.8, 29.1, 29.0, 28.8, 28.7, 26.1, 26.0, 22.72, 22.69, 14.19, 14.18, 11.0.

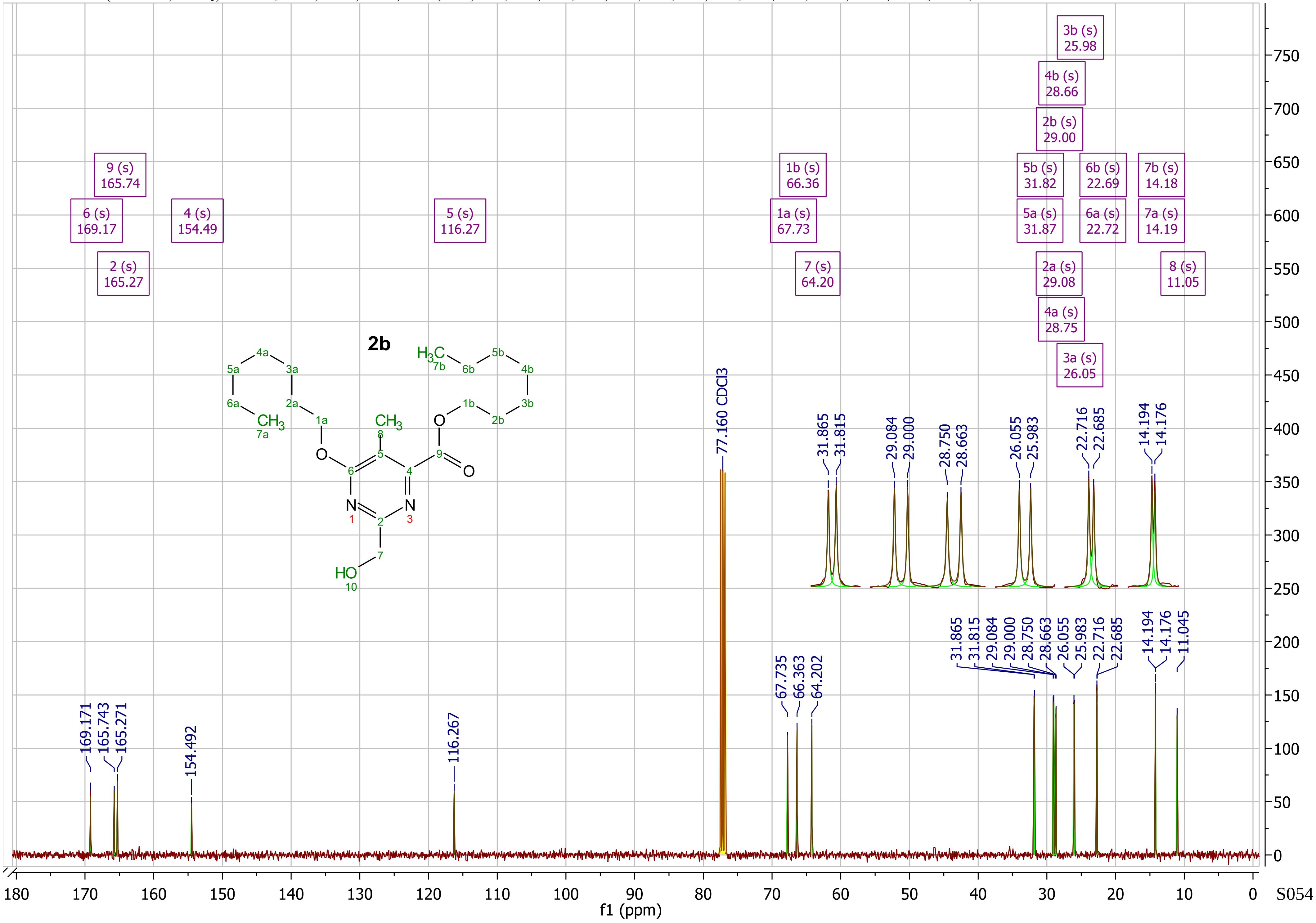

13C HSQC

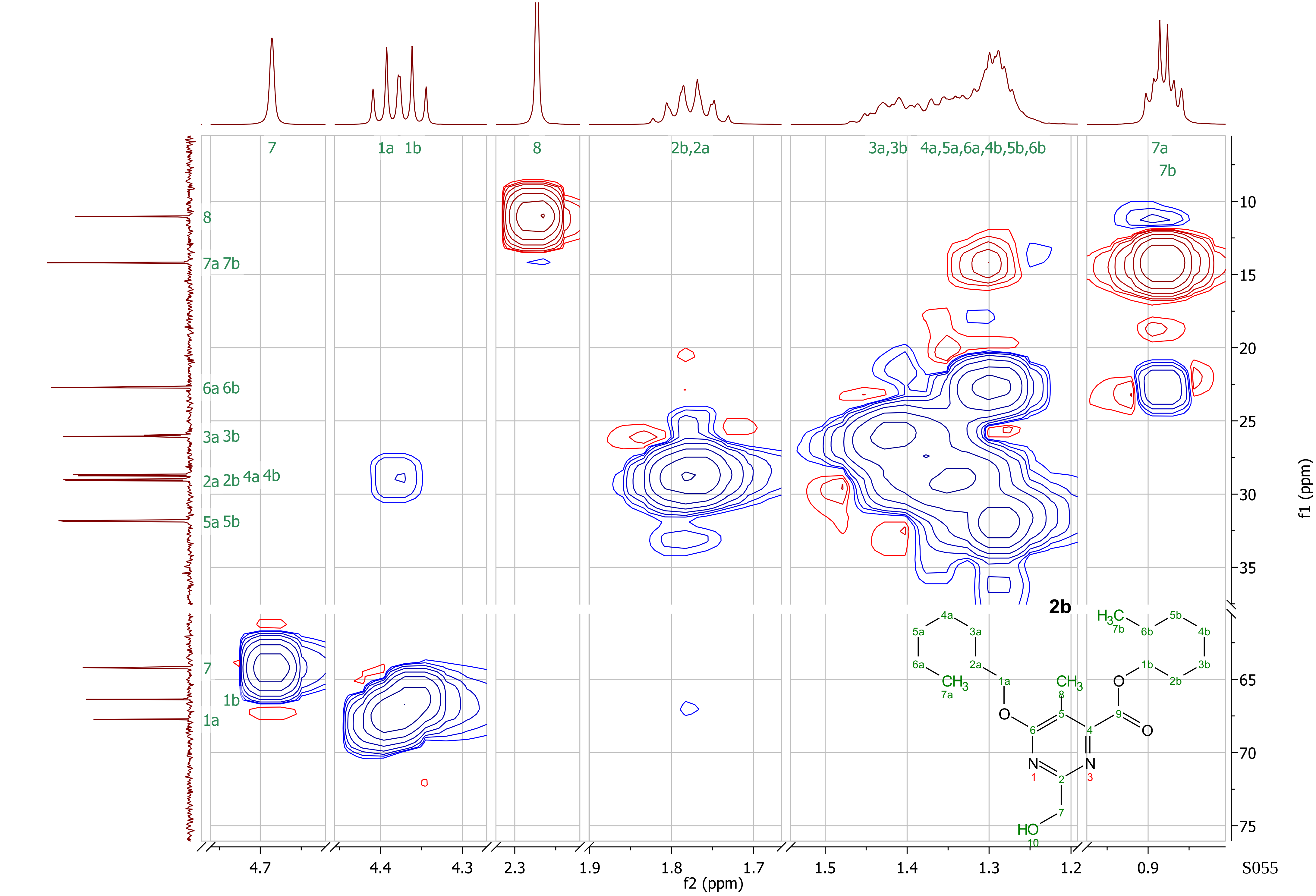

S055

13C HMBC

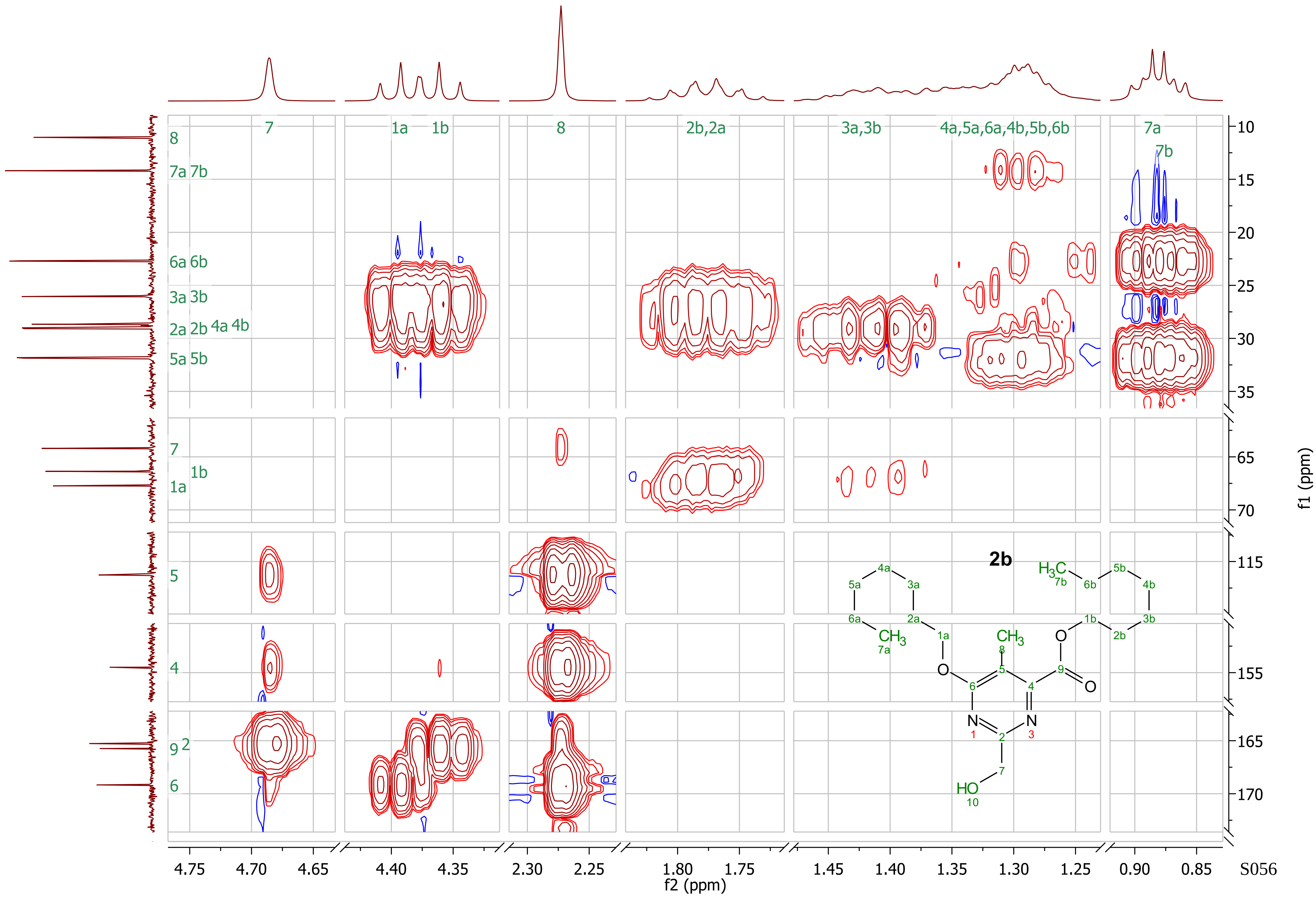

S056

1H ^{1}H NMR (400 MHz, $CDCl_3$) δ 4.69 (s, 2H), 4.39 (t, *J* = 6.3 Hz, 2H), 4.36 (t, *J* = 6.4 Hz, 2H), 3.22 (br s, 1H), 2.27 (s, 3H), 1.85 – 1.70 (m, 4H), 1.50 – 1.36 (m, 4H), 1.38 – 1.19 (m, 16H), 0.88 (app t, *J* = 6.7 Hz, 3H), 0.87 (app t, *J* = 7.1 Hz, 3H).

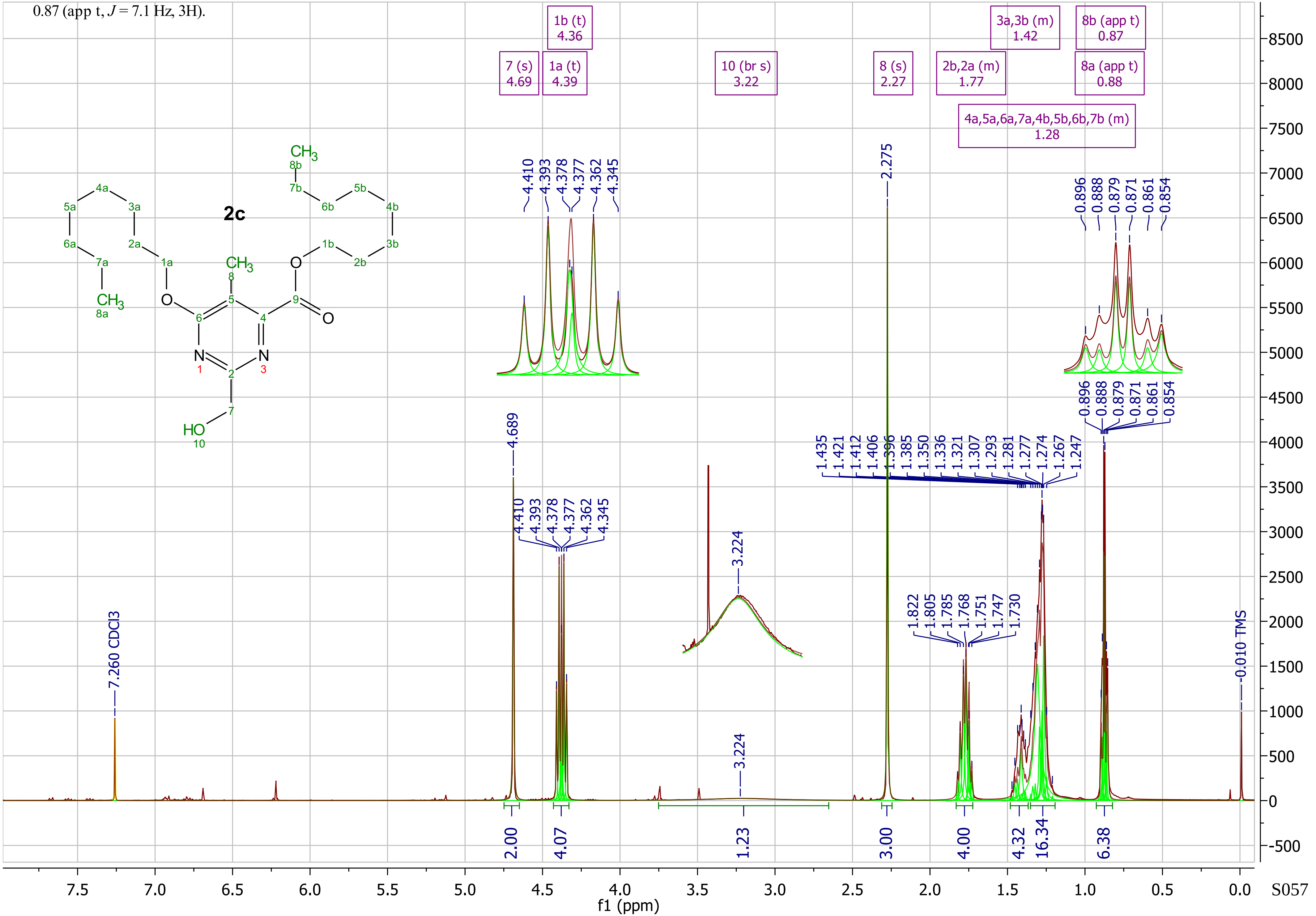

13C ^{13}C NMR (101 MHz, $CDCl_3$) δ 169.2, 165.7, 165.3, 154.5, 116.3, 67.8, 66.4, 64.2, 31.91, 31.88, 29.4, 29.33, 29.30, 29.28, 28.74, 28.66, 26.1, 26.0, 22.78, 22.76, 14.22, 14.21, 11.1.

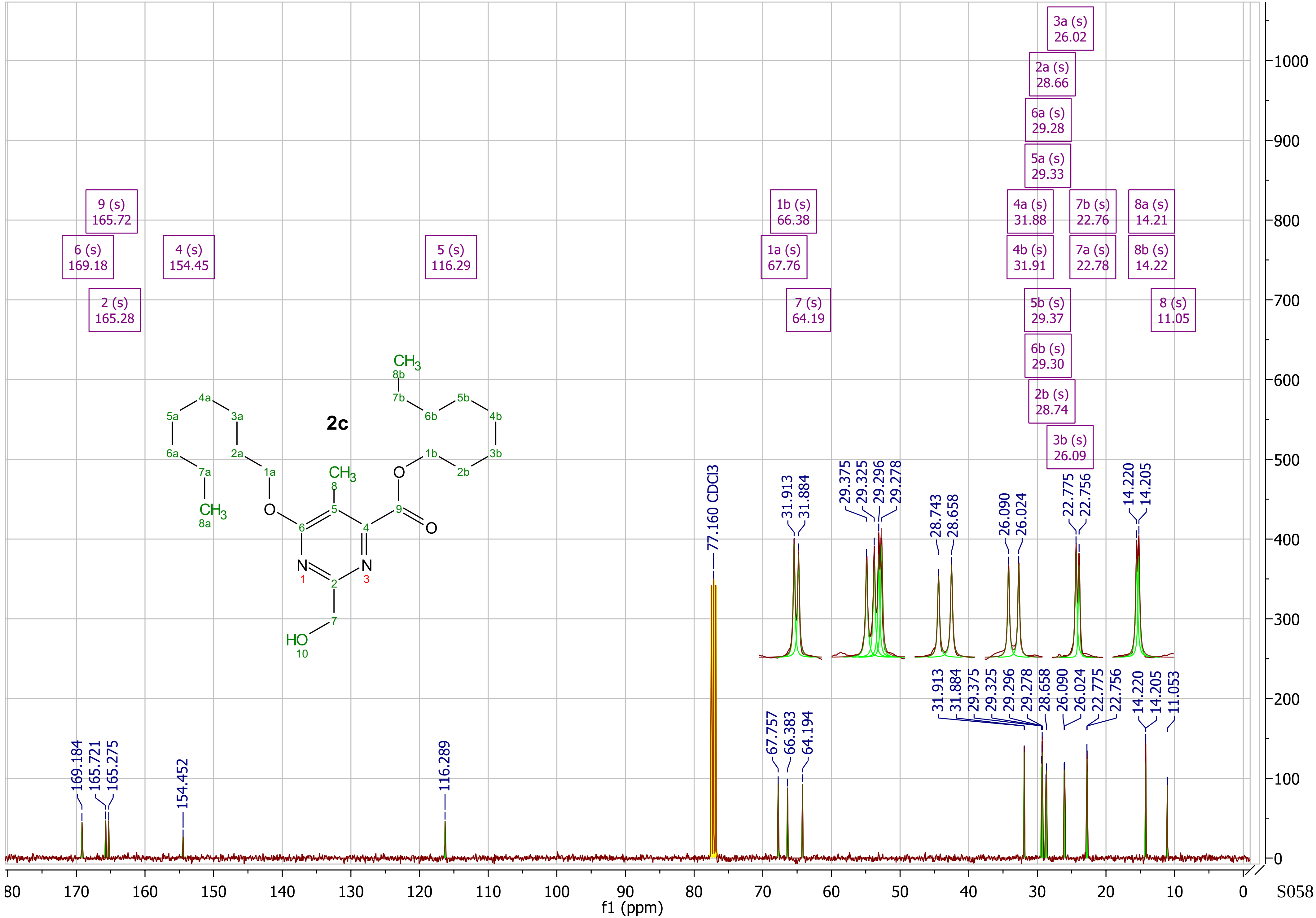

S058

13C HSQC

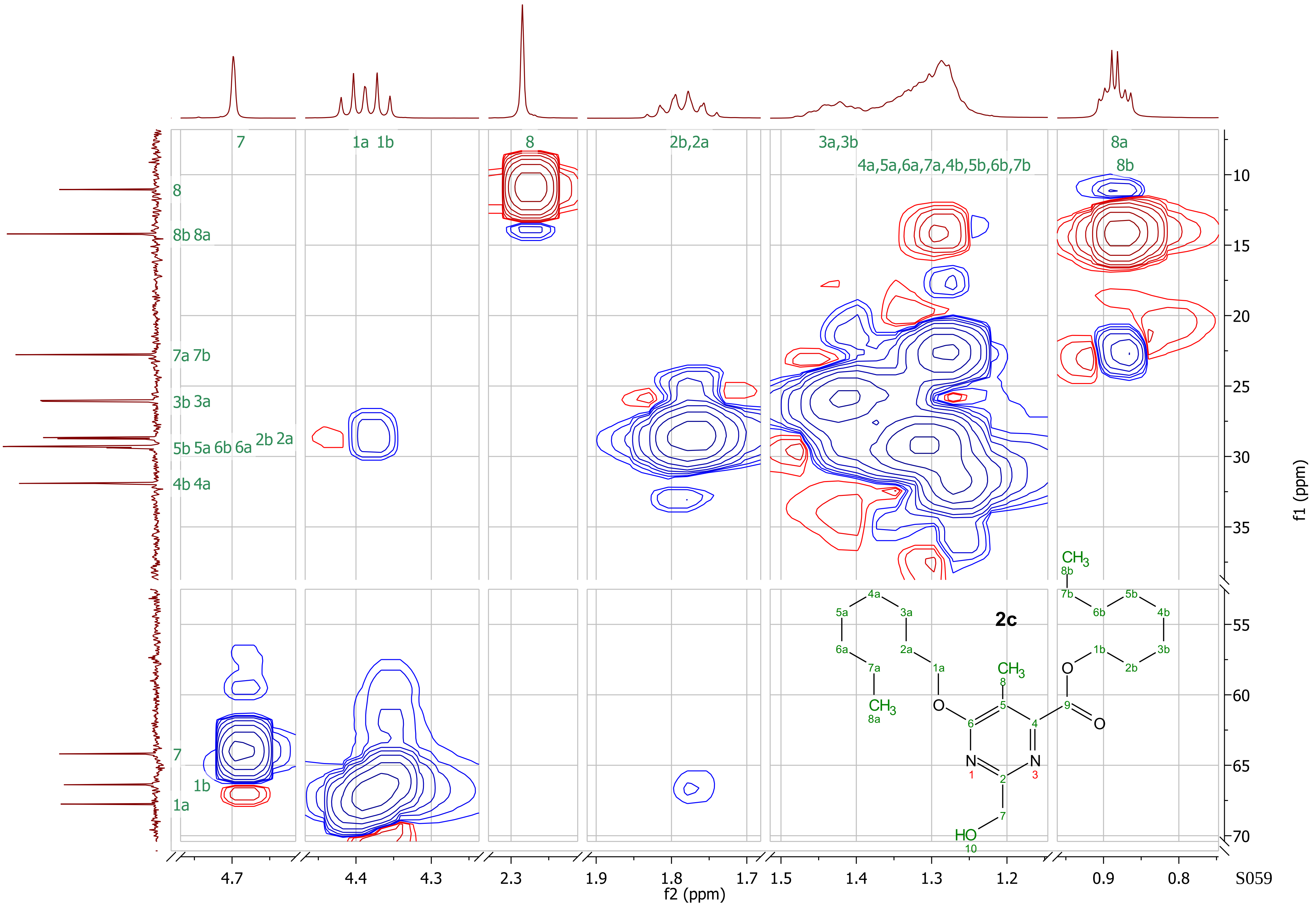

7
1a 1b
8
2b,2a
3a,3b
4a,5a,6a,7a,4b,5b,6b,7b
8a
8b
8
8b 8a
7a 7b
3b 3a
5b 5a 6b 6a 2b 2a
4b 4a
7
1b
1a
2c
f1 (ppm)
f2 (ppm)

13C HMBC

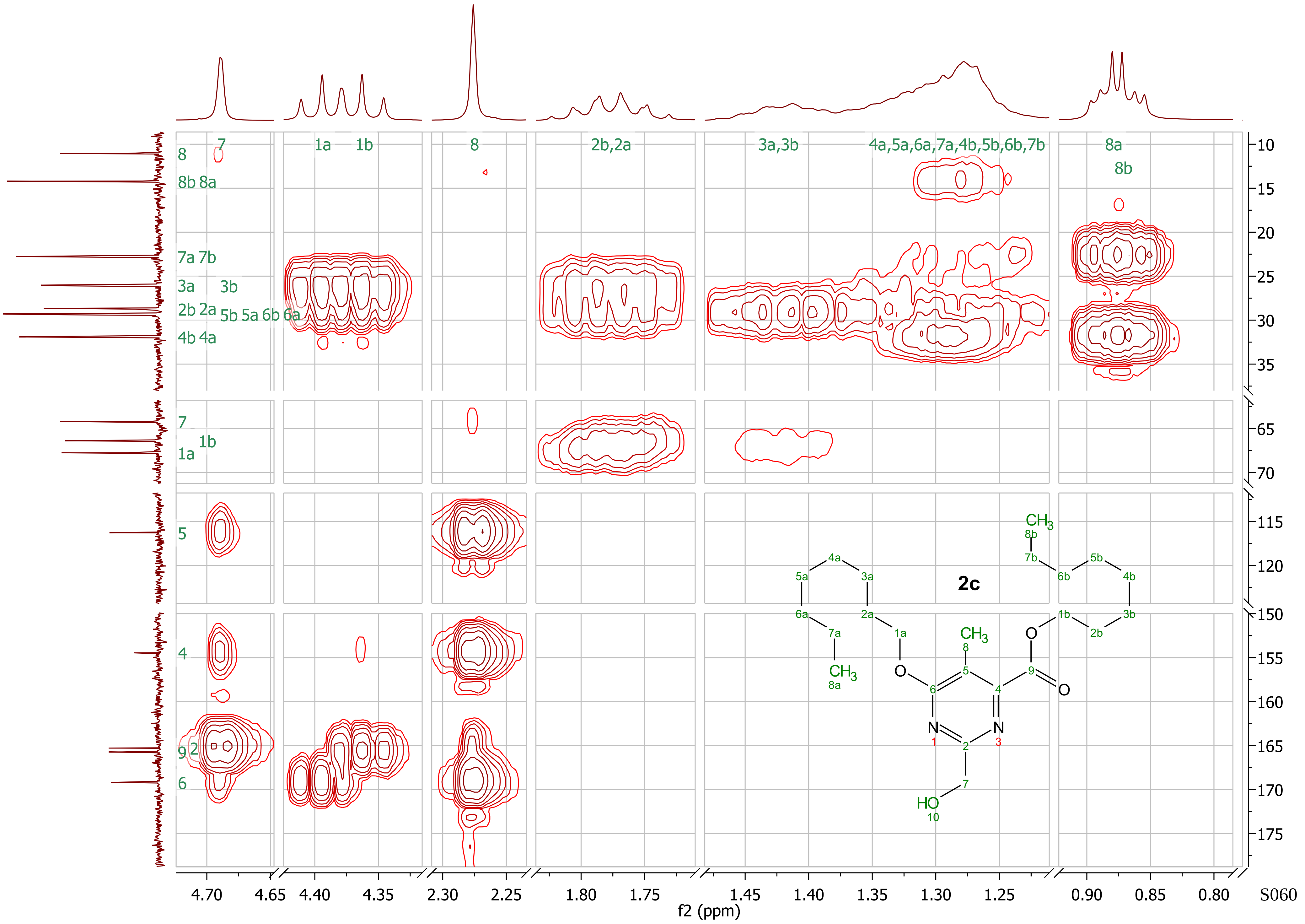

S060

1H ^{1}H NMR (400 MHz, $CDCl_3$) δ 4.68 (s, 2H), 4.29 (d, J = 4.4 Hz, 2H), 4.28 (d, J = 4.7 Hz, 2H), 3.59 (br s, 1H), 2.28 (s, 3H), 1.93 – 1.70 (m, 2H), 1.46 – 1.28 (m, 16H), 1.01 – 0.77 (m, 12H).

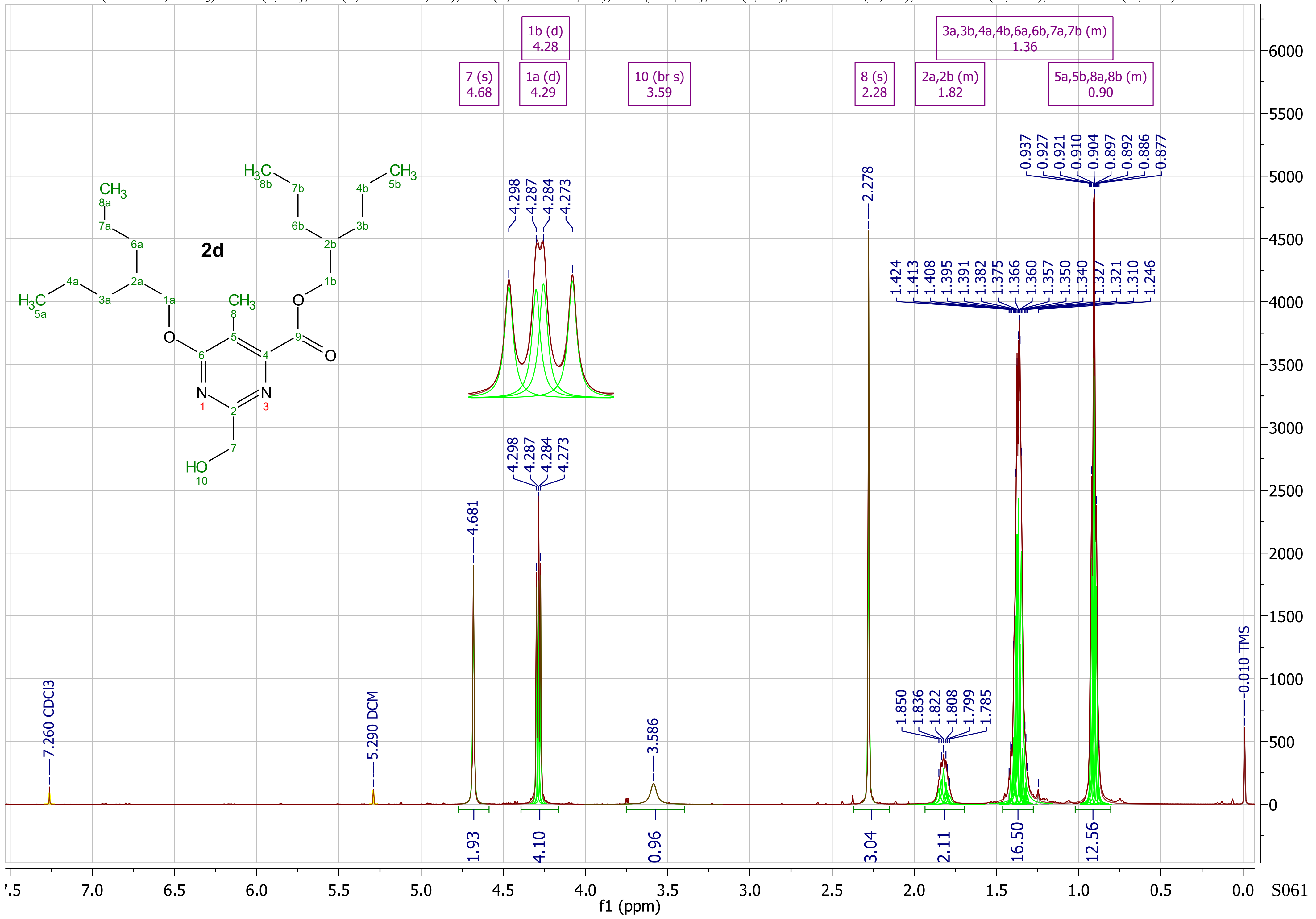

S061

13C ^{13}C NMR (101 MHz, $CDCl_3$) δ 169.3, 165.9, 165.3, 154.5, 116.2, 70.4, 69.0, 64.1, 37.1, 37.0, 33.9 (sym, 2C), 33.7 (sym, 2C), 20.1 (sym, 2C), 20.0 (sym, 2C), 14.51 (sym, 2C), 14.48 (sym, 2C), 11.1.

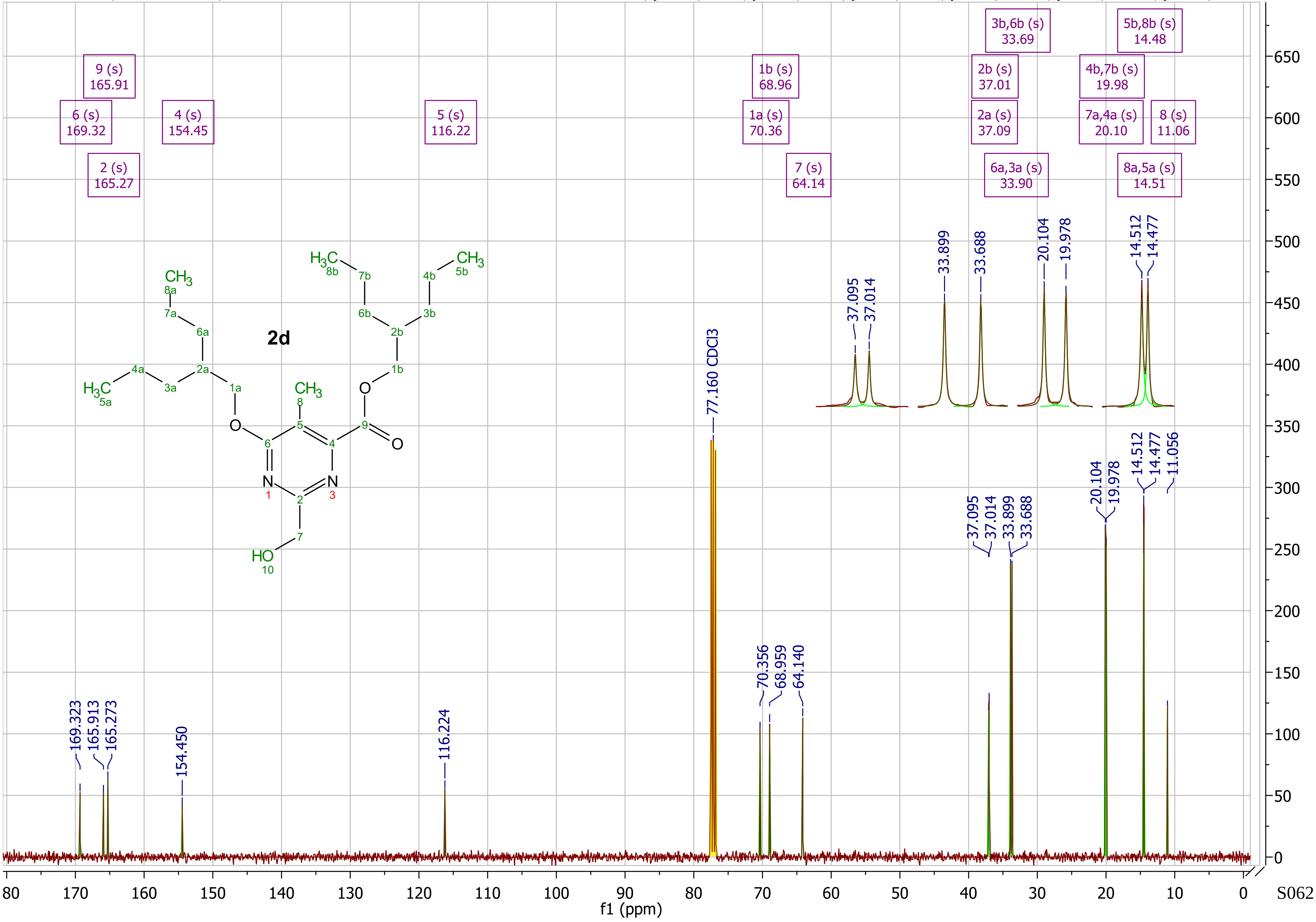

S062

13C HSQC

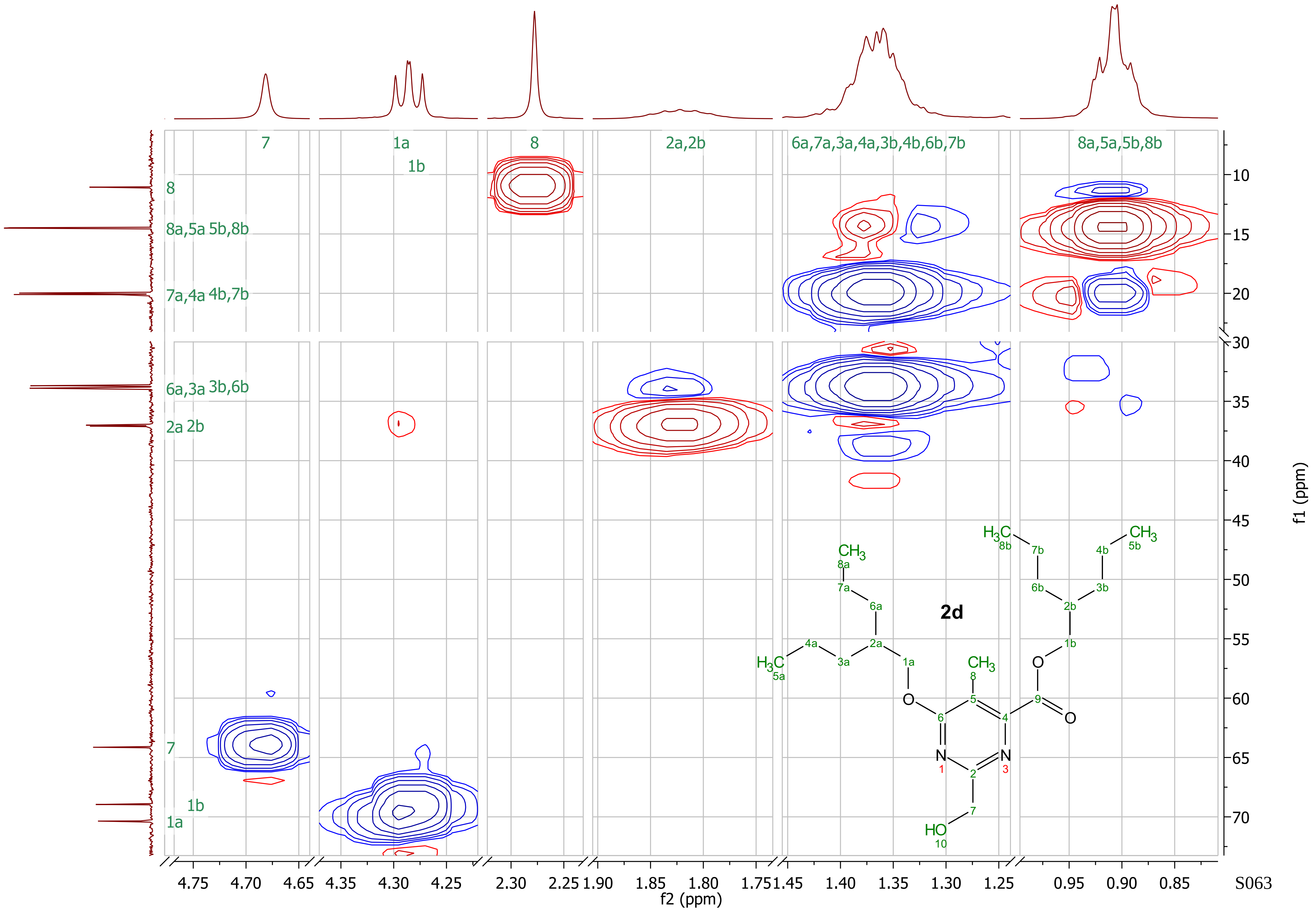

S063

13C HMBC

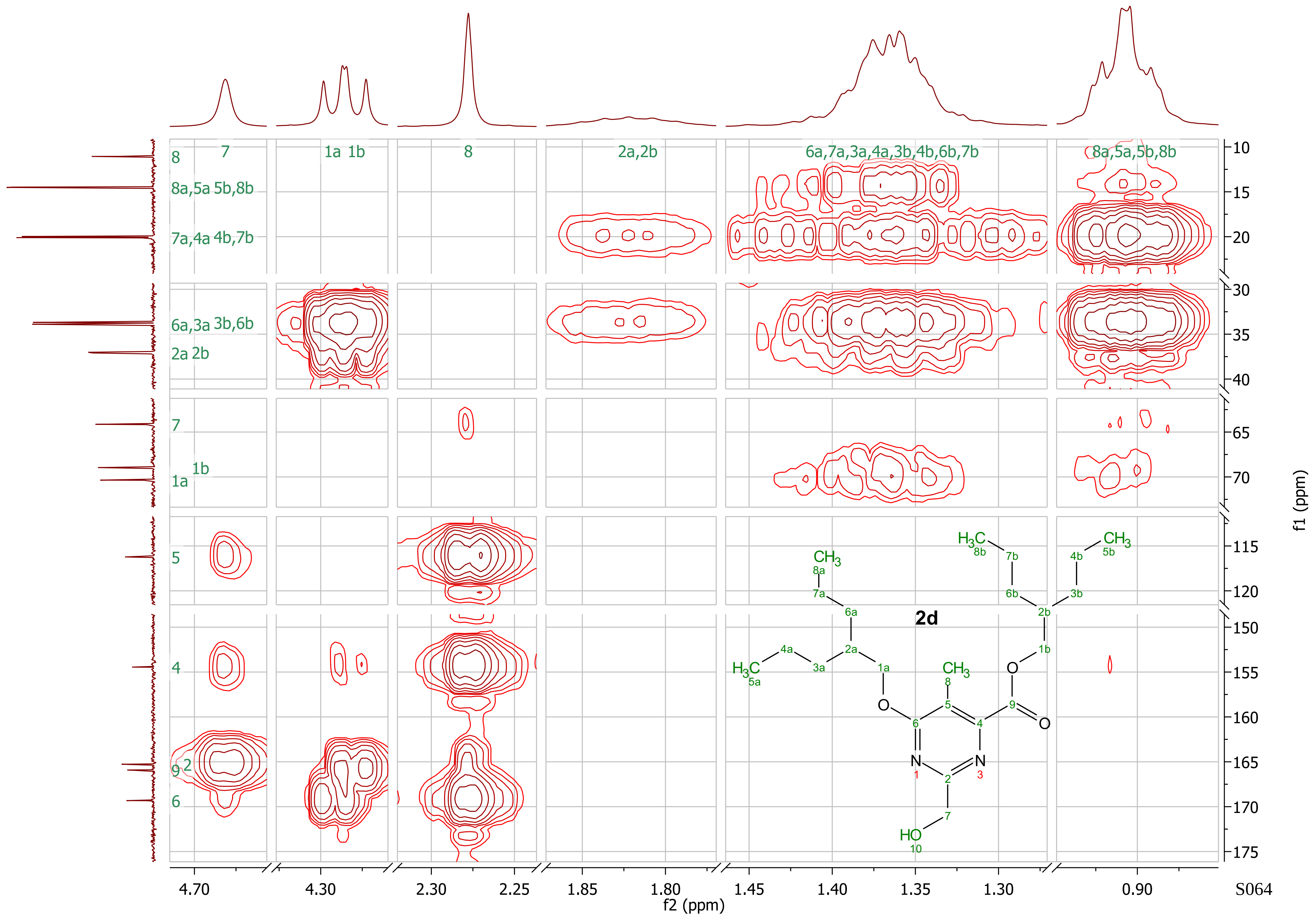

S064

1H ^{1}H NMR (400 MHz, $CDCl_3$) δ 5.27 (quint, J = 5.9 Hz, 1H), 5.13 (quint, J = 6.1 Hz, 1H), 4.66 (s, 2H), 3.62 (br s, 1H), 2.24 (s, 3H), 1.84 – 1.52 (m, 8H), 1.47 – 1.18 (m, 8H), 0.98 (t, J = 7.4 Hz, 3H), 0.91 (t, J = 7.4 Hz, 3H), 0.91 (app t, J = 7.0 Hz, 3H), 0.89 (app t, J = 6.9 Hz, 3H).

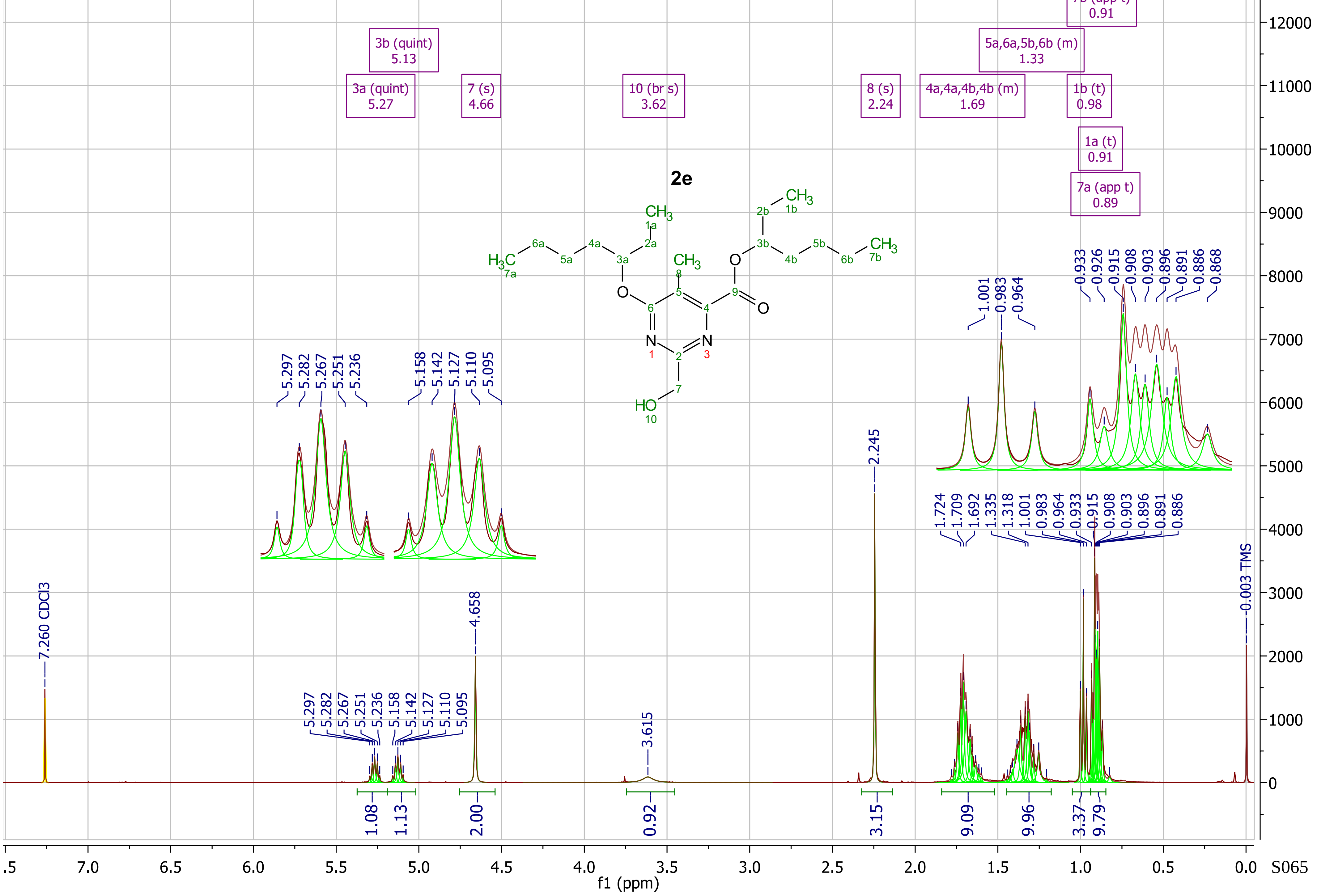

13C ^{13}C NMR (101 MHz, $CDCl_3$) δ 169.1, 165.9, 165.1, 155.4, 115.5, 78.4, 78.1, 64.1, 33.4, 33.1, 27.7, 27.5, 27.1, 26.7, 22.8, 22.7, 14.1 (2C), 11.1, 9.8, 9.6.

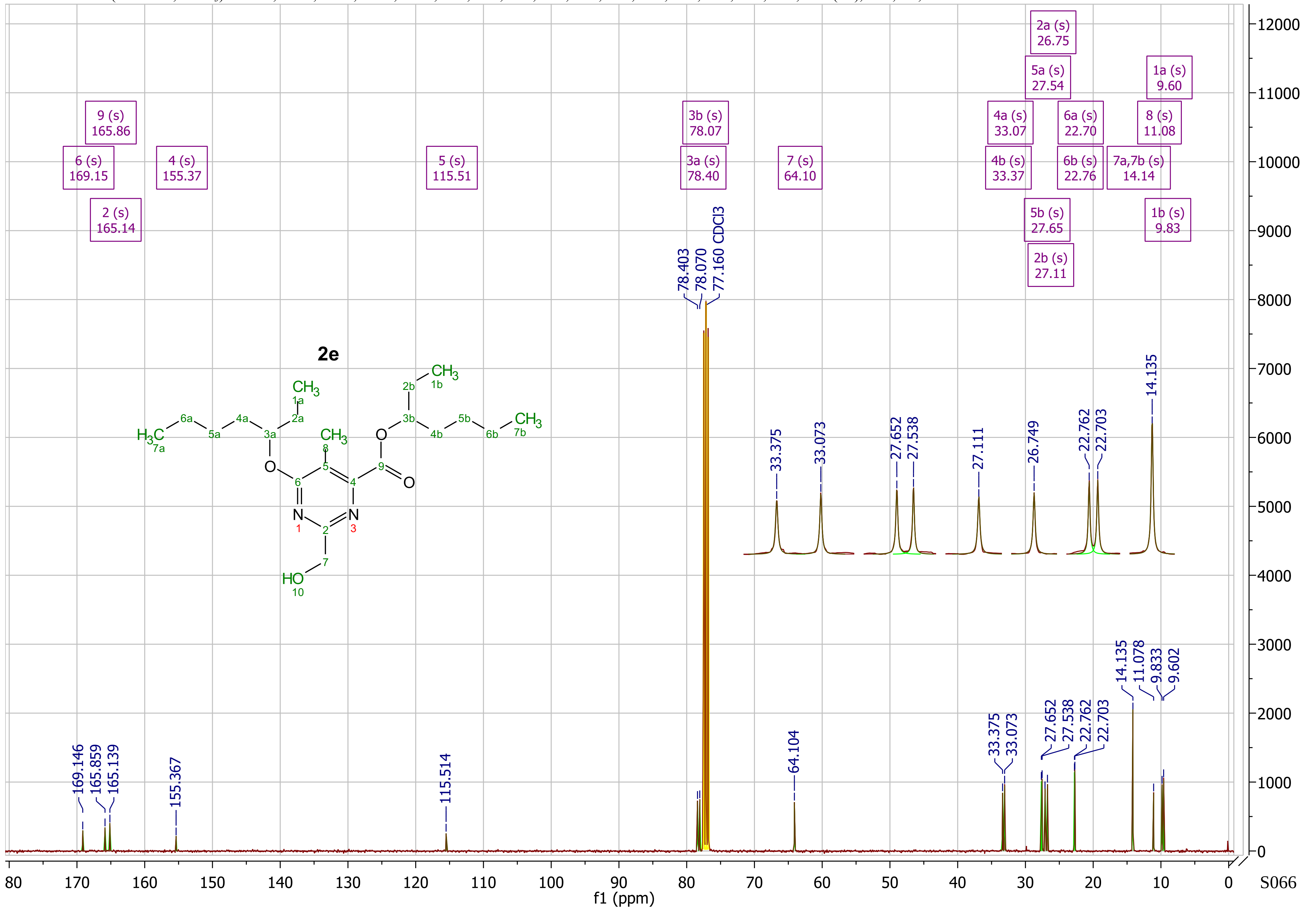

S066

13C HSQC

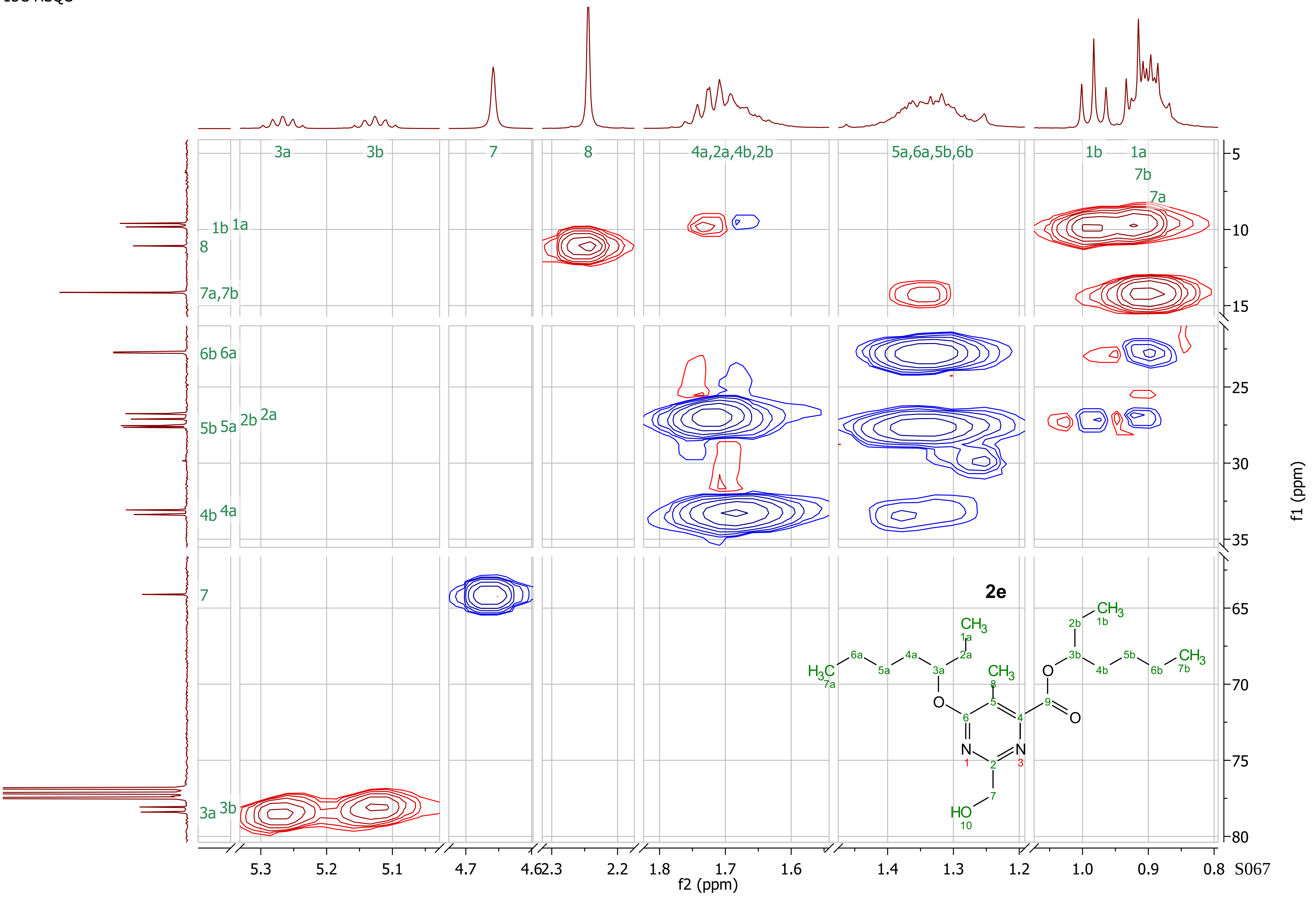

S067

13C HMBC

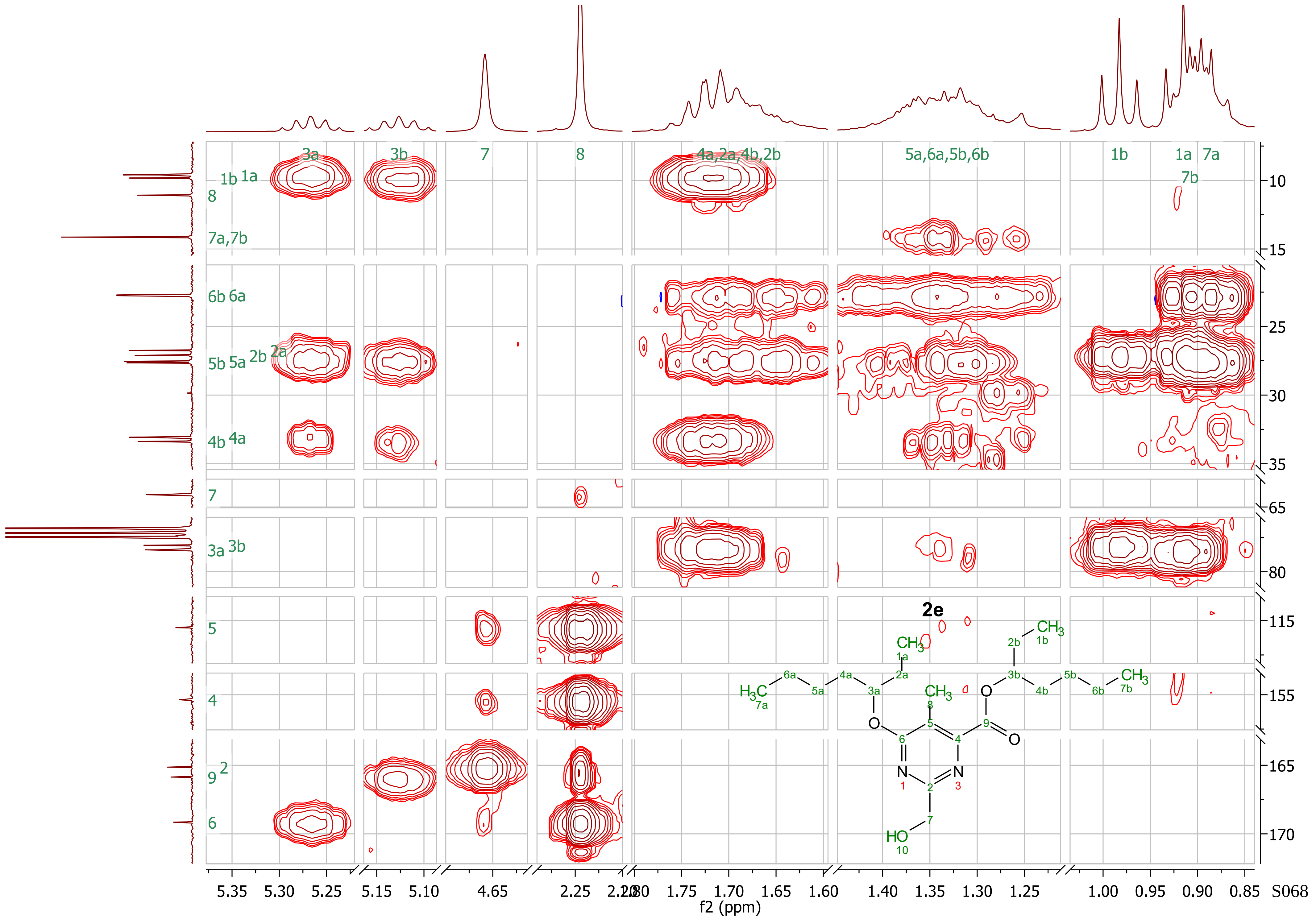

3a
3b
7
8
4a,2a,4b,2b
5a,6a,5b,6b
1b
1a
7a
7b
8
1b 1a
7a,7b
6b 6a
5b 5a 2b 2a
4b 4a
7
3a 3b
5
4
9 2
6
2e
f2 (ppm)
f1 (ppm)
S068

1H ^{1}H NMR (400 MHz, $CDCl_3$) δ 7.71 (s, 2H), 7.68 – 7.58 (m, 4H), 7.52 (t, *J* = 7.7 Hz, 2H), 5.52 (s, 2H), 5.46 (s, 2H), 4.73 (s, 2H), 3.42 (br s, 1H), 2.33 (s, 3H).

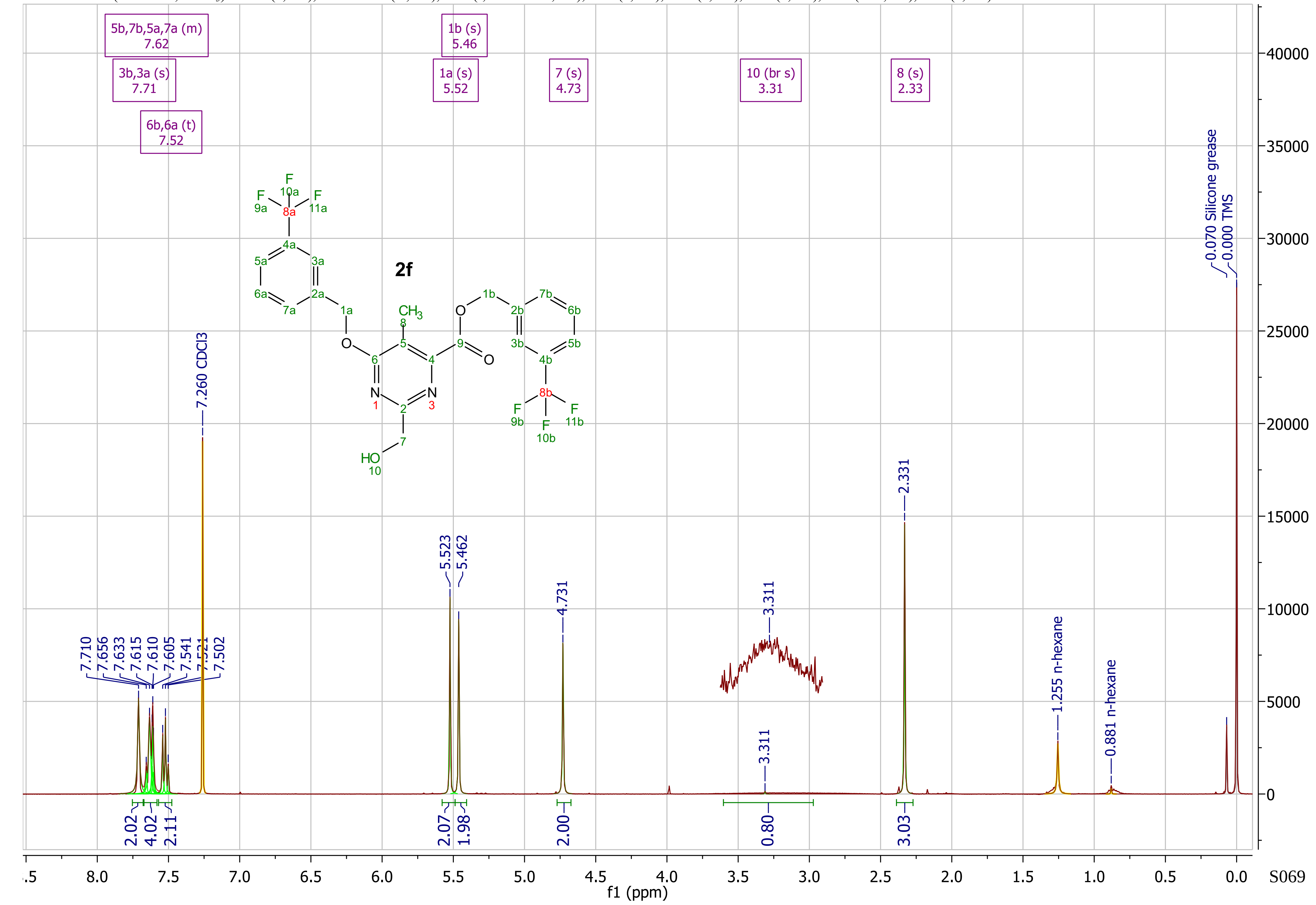

^{13}C NMR (101 MHz, $CDCl_3$) δ 168.68, 165.49, 164.96, 154.06, 136.87, 136.23, 131.69 (app q, J = 1.1 Hz), 131.36 (app q, J = 1.0 Hz), 131.31 (d, J = 32.7 Hz), 131.25 (d, J = 32.4 Hz), 129.41, 129.34, 125.56 (q, J = 3.8 Hz), 125.37 (q, J = 3.9 Hz), 125.16 (q, J = 3.5 Hz), 124.92 (q, J = 3.7 Hz), 117.19, 68.29, 66.84, 64.24, 11.11.

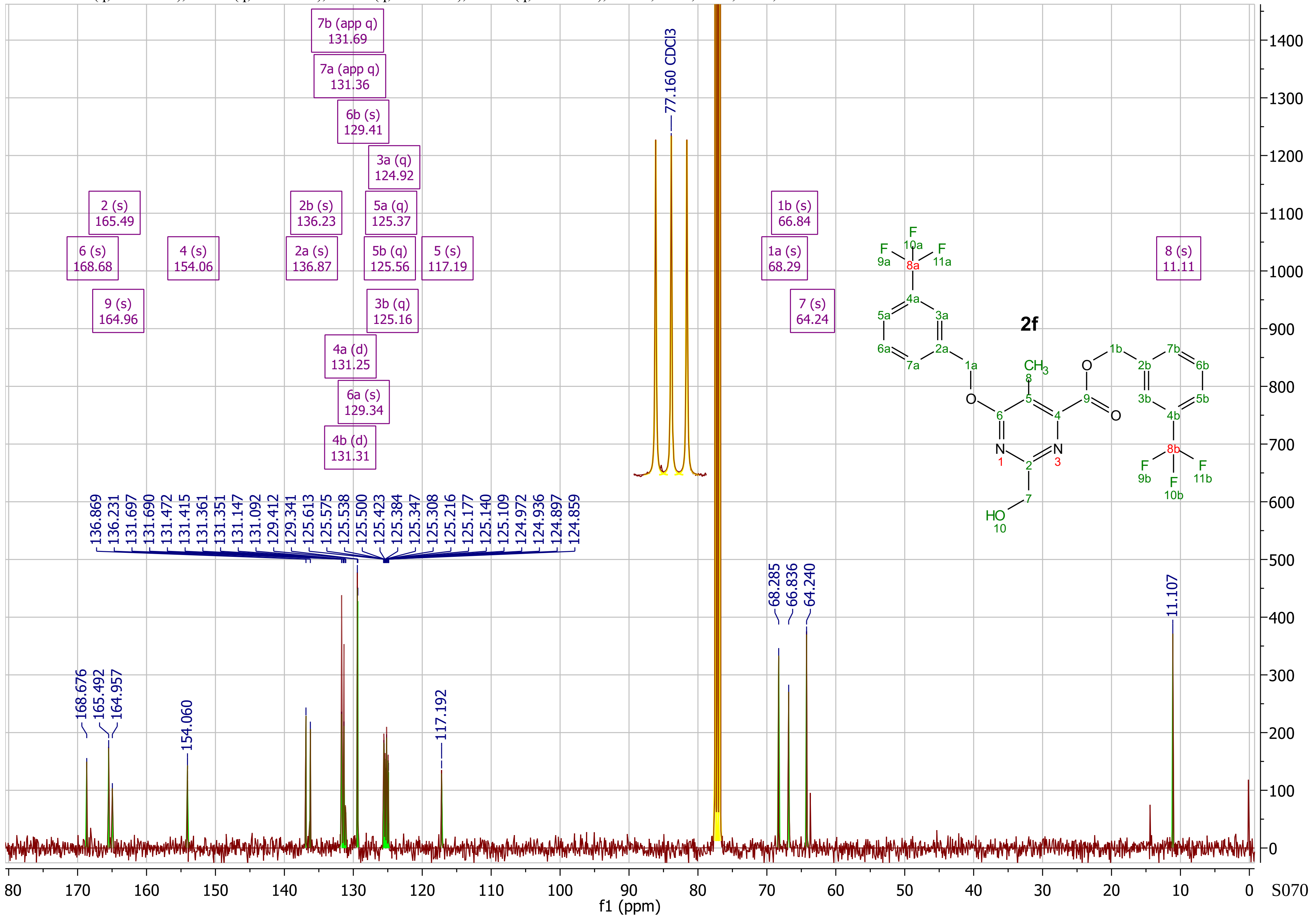

13C [124 — 132.5 ppm]

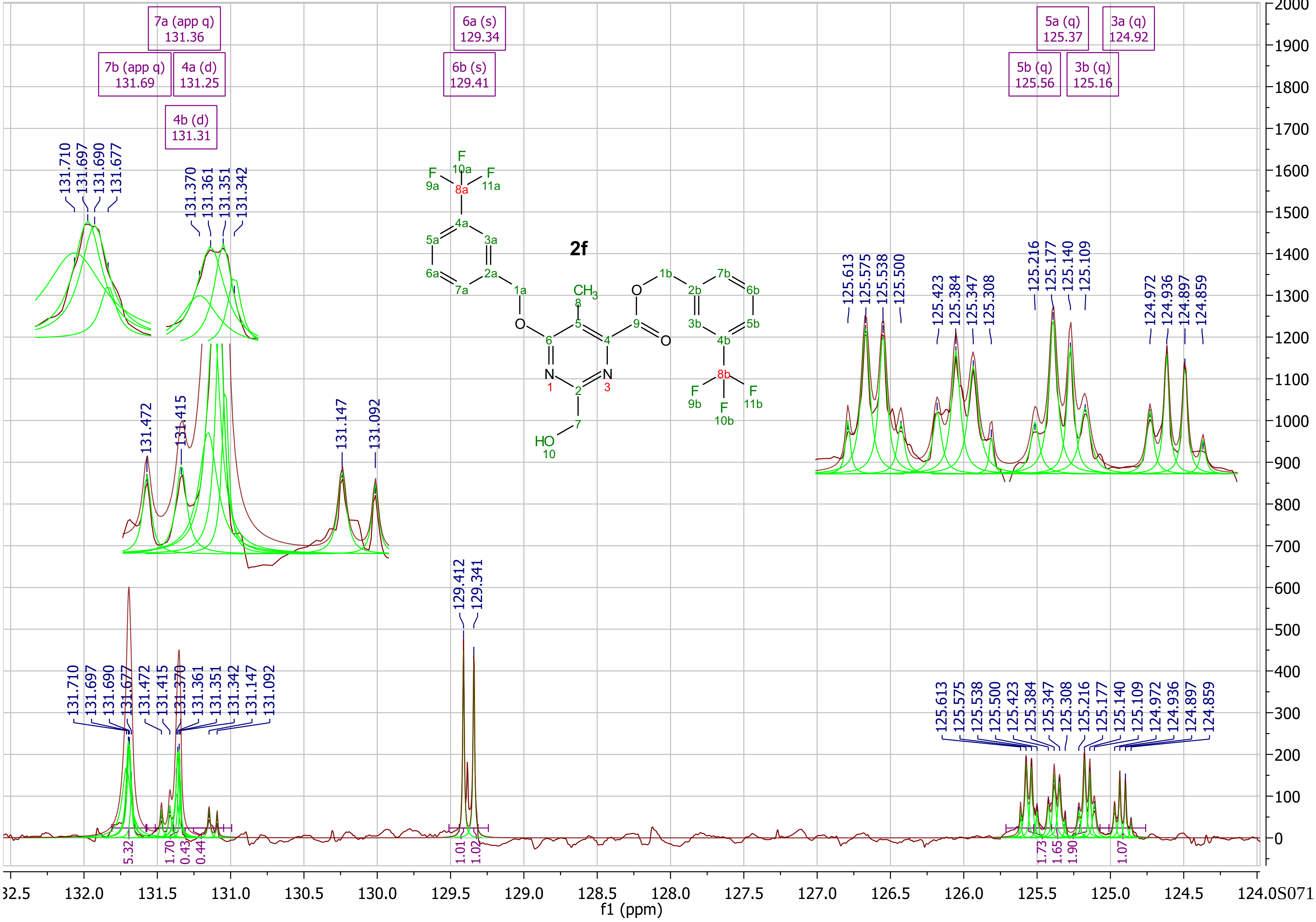

13C HSQC

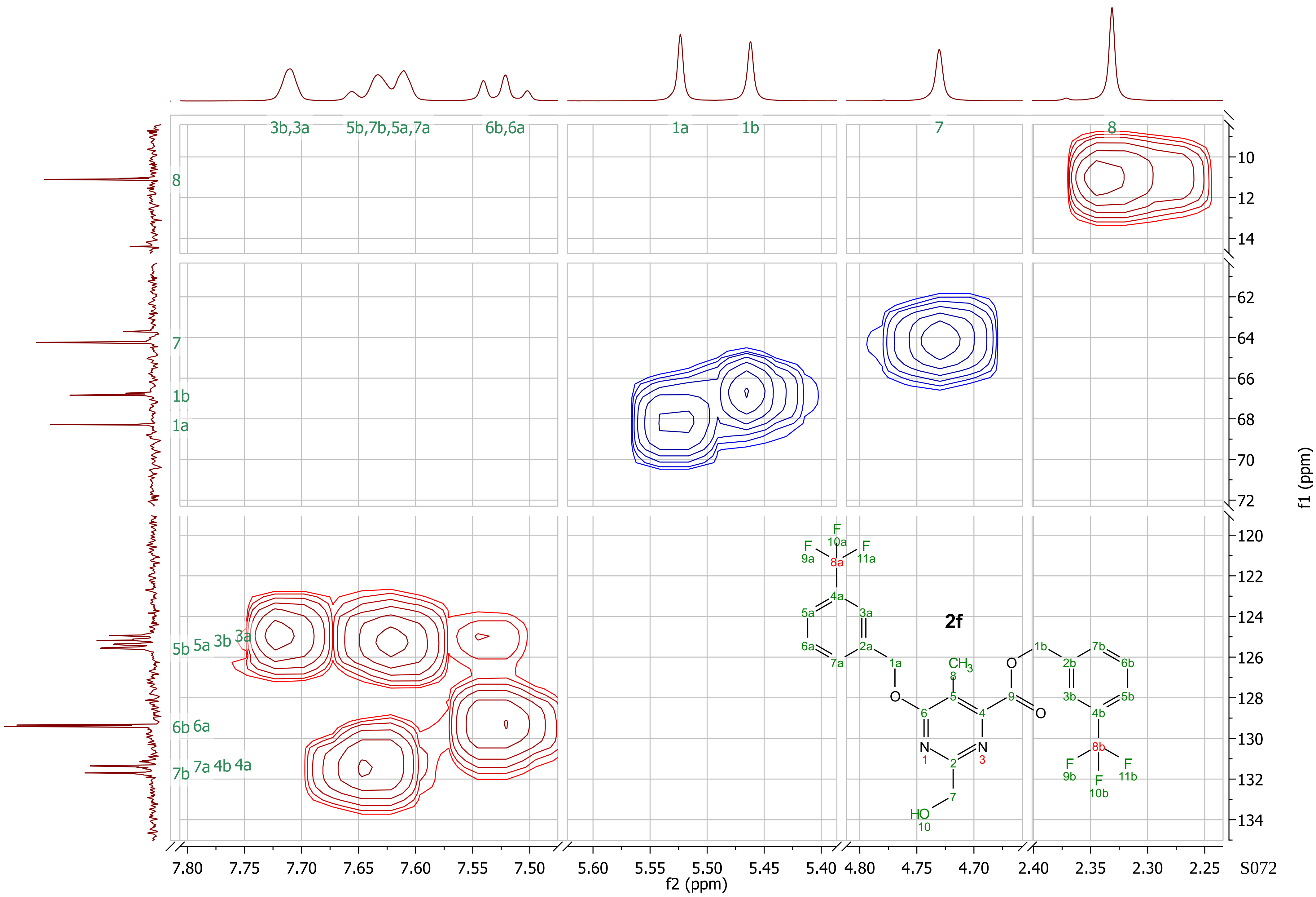

13C HMBC

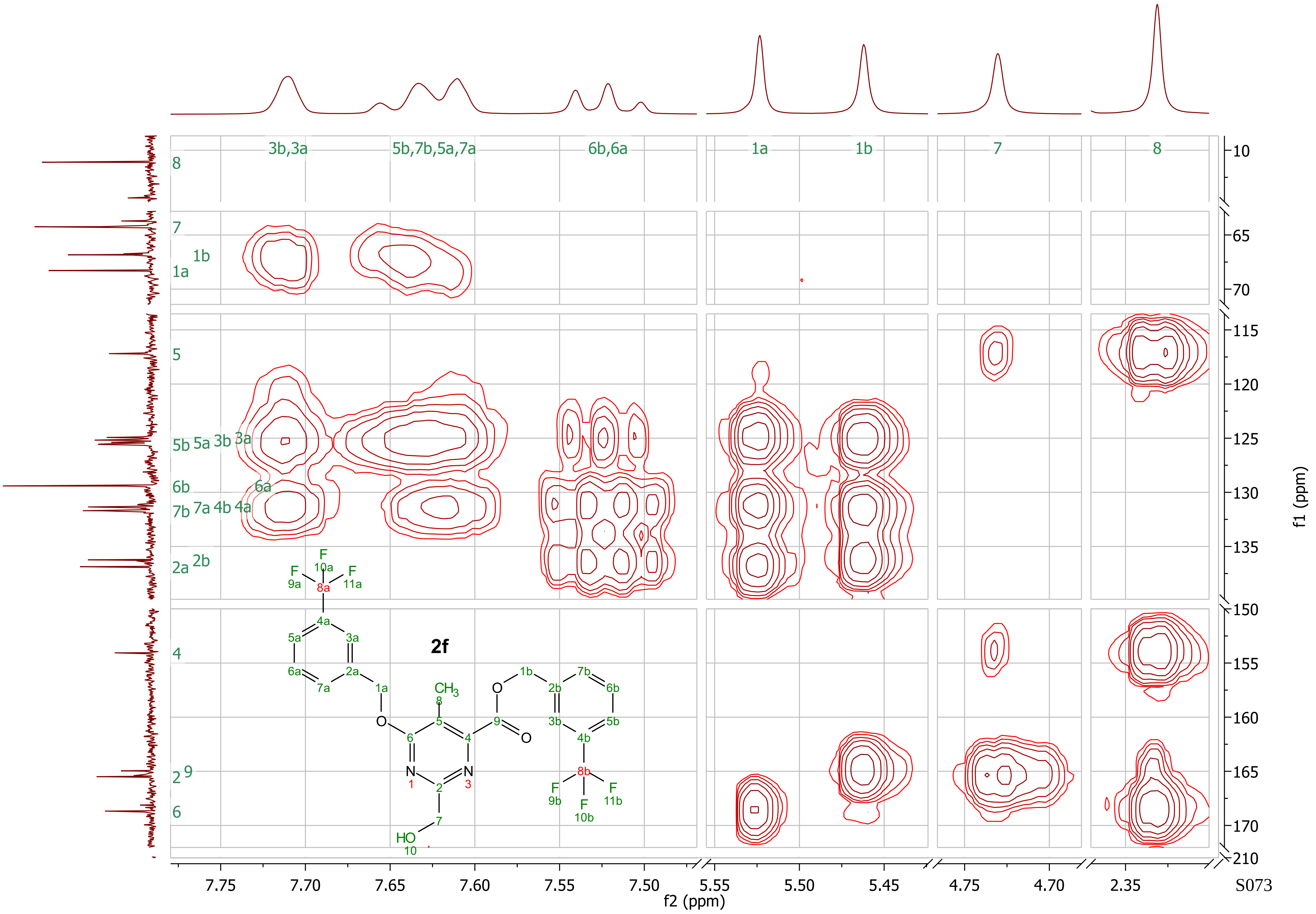

S073

19F ^{19}F NMR (376 MHz, $CDCl_3$) δ -62.73, -62.74.

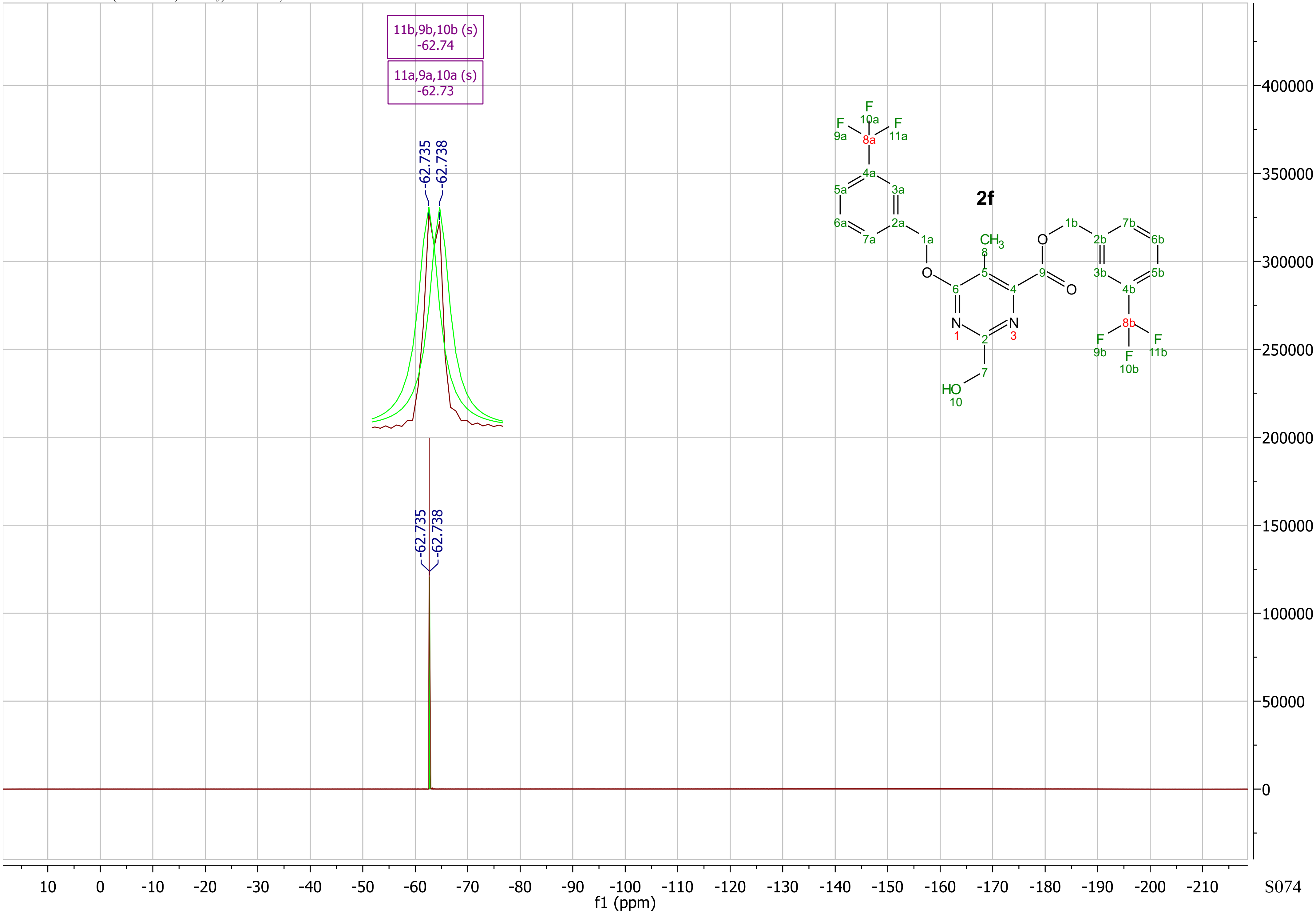

1H ^{1}H NMR (400 MHz, $CDCl_3$) δ 7.71 (s, 1H), 7.67 – 7.58 (m, 2H), 7.52 (t, J = 7.7 Hz, 1H), 5.53 (s, 2H), 4.74 (app d, J = 0.5 Hz, 2H), 3.98 (s, 3H), 3.06 (s, 1H), 2.37 (s, 3H).

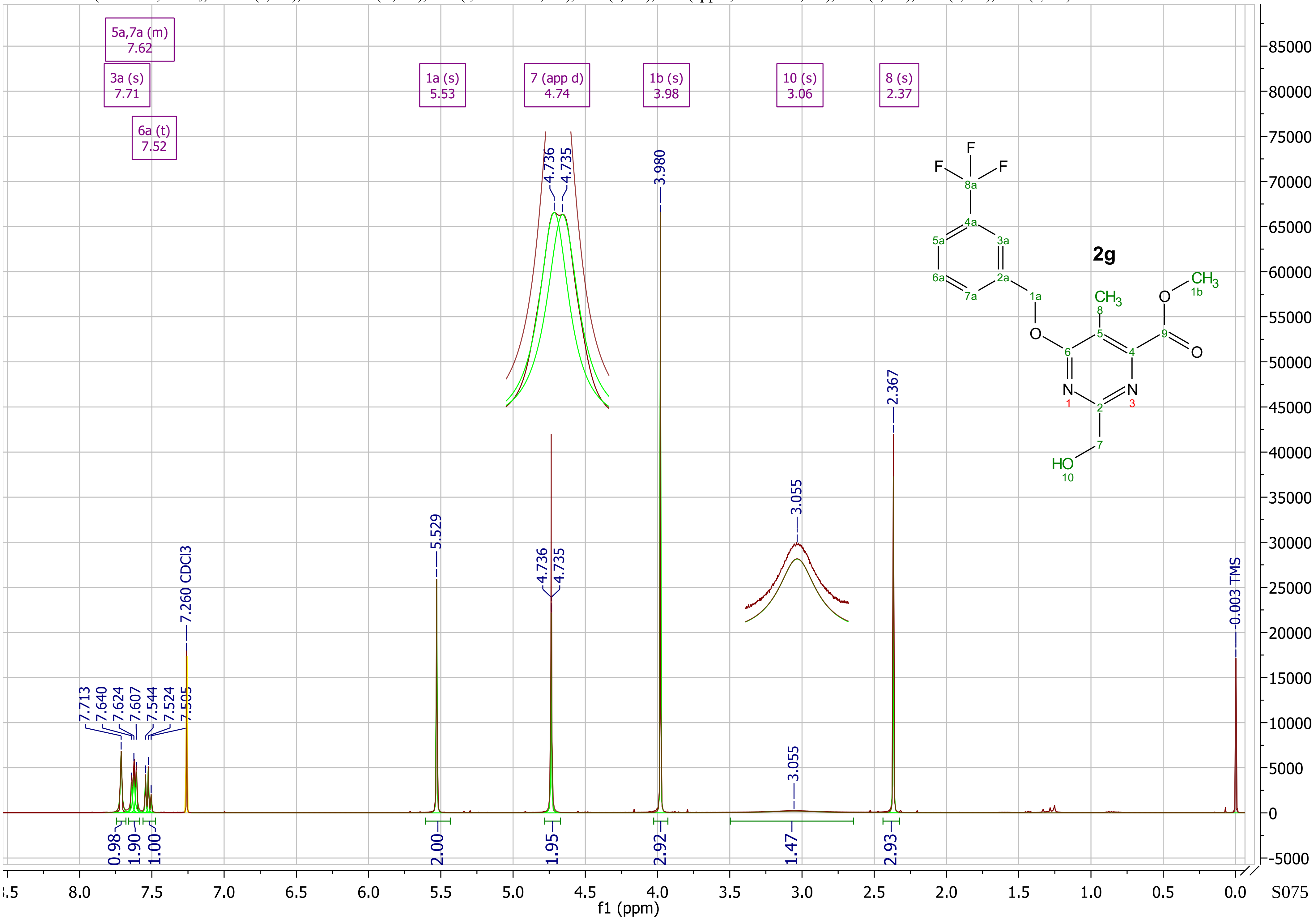

13C ^{13}C NMR (101 MHz, $CDCl_3$) δ 168.7, 165.7, 165.4, 154.3, 136.9, 131.3 (app q, J = 1.0 Hz), 131.3 (q, J = 32.5 Hz), 129.3, 125.3 (q, J = 3.9 Hz), 124.9 (q, J = 3.8 Hz), 124.1 (q, J = 272.4 Hz), 117.2, 68.3, 64.3, 53.1, 11.1.

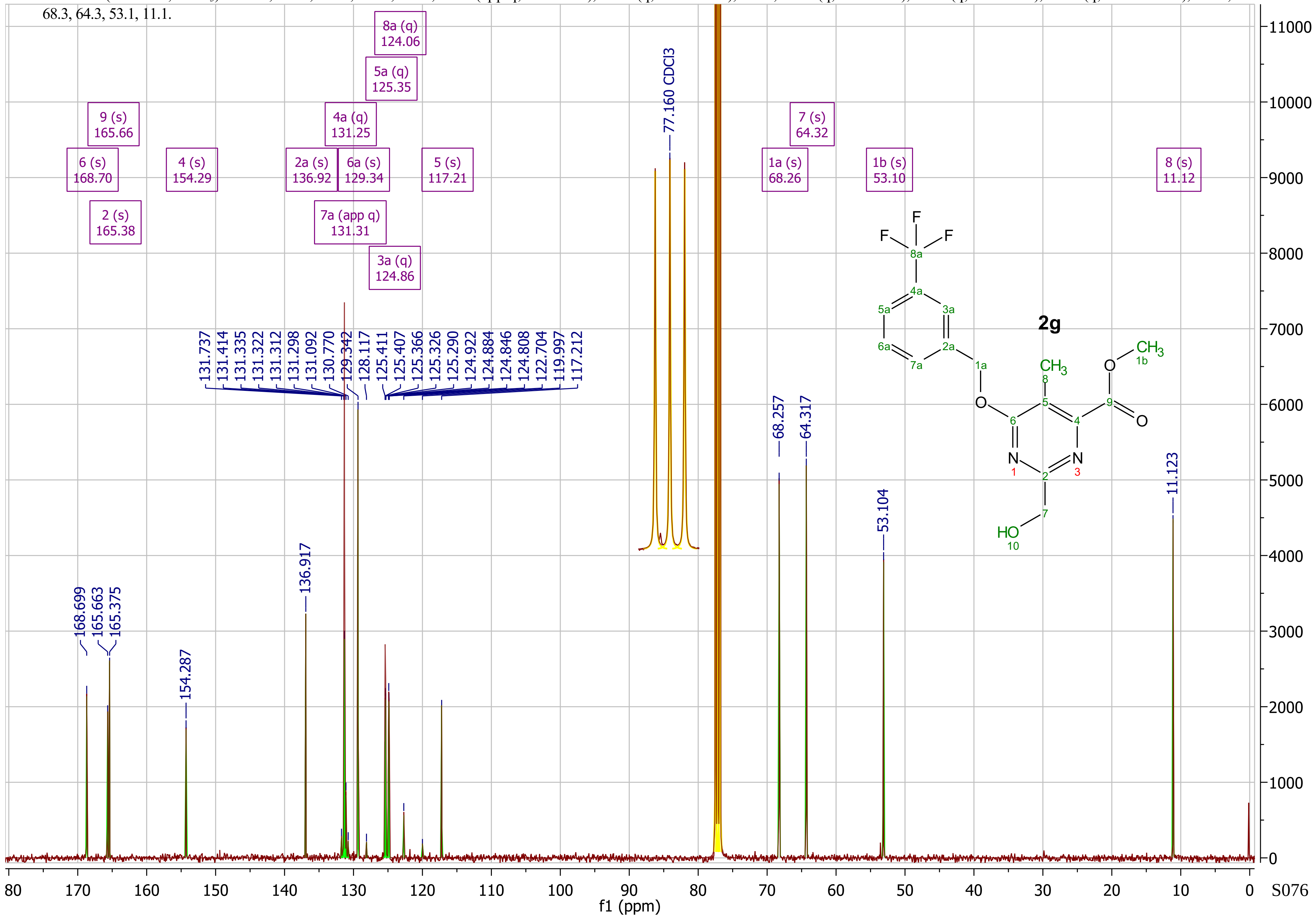

13C [118.5 — 132.5 ppm]

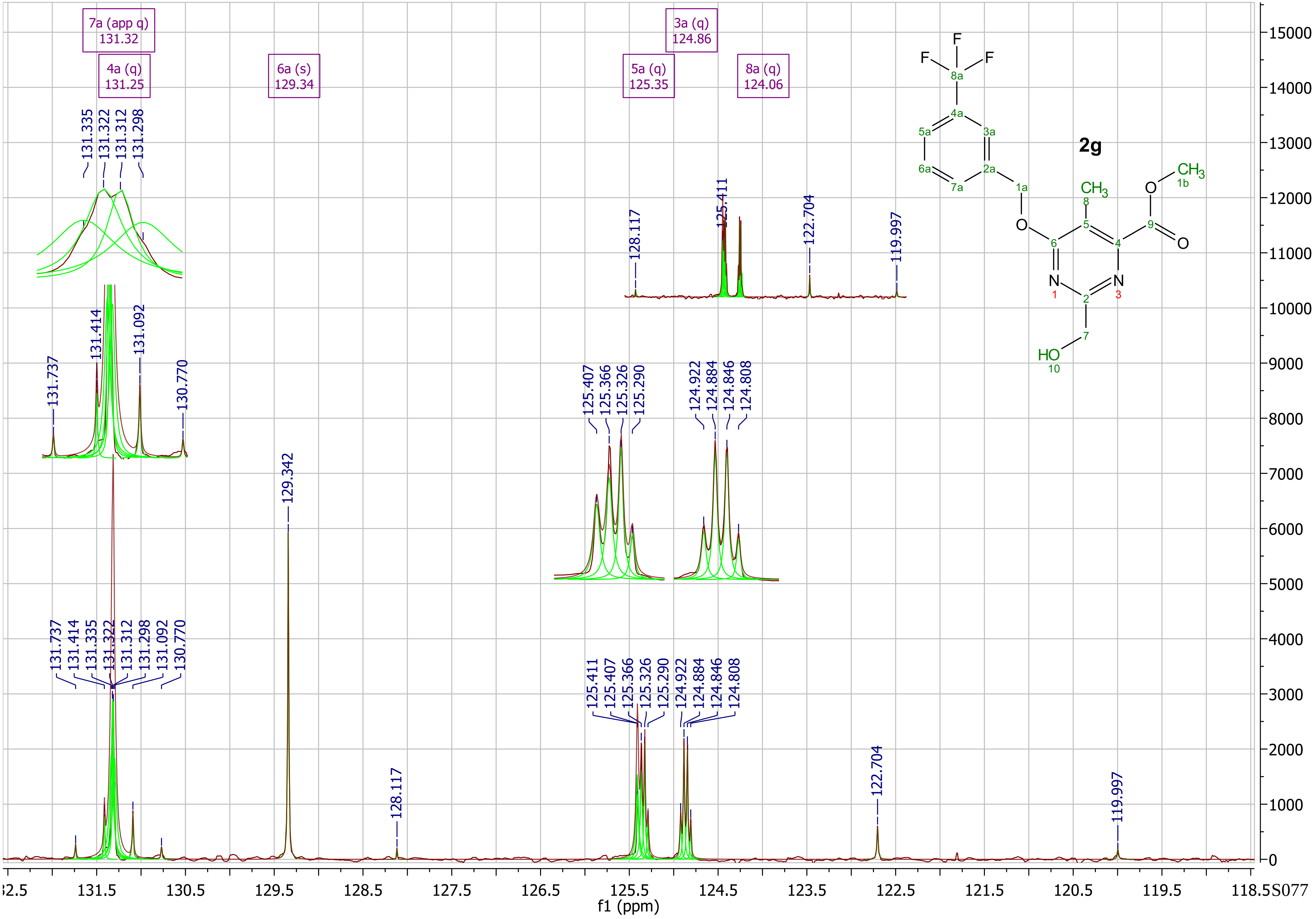

S077

13C HSQC

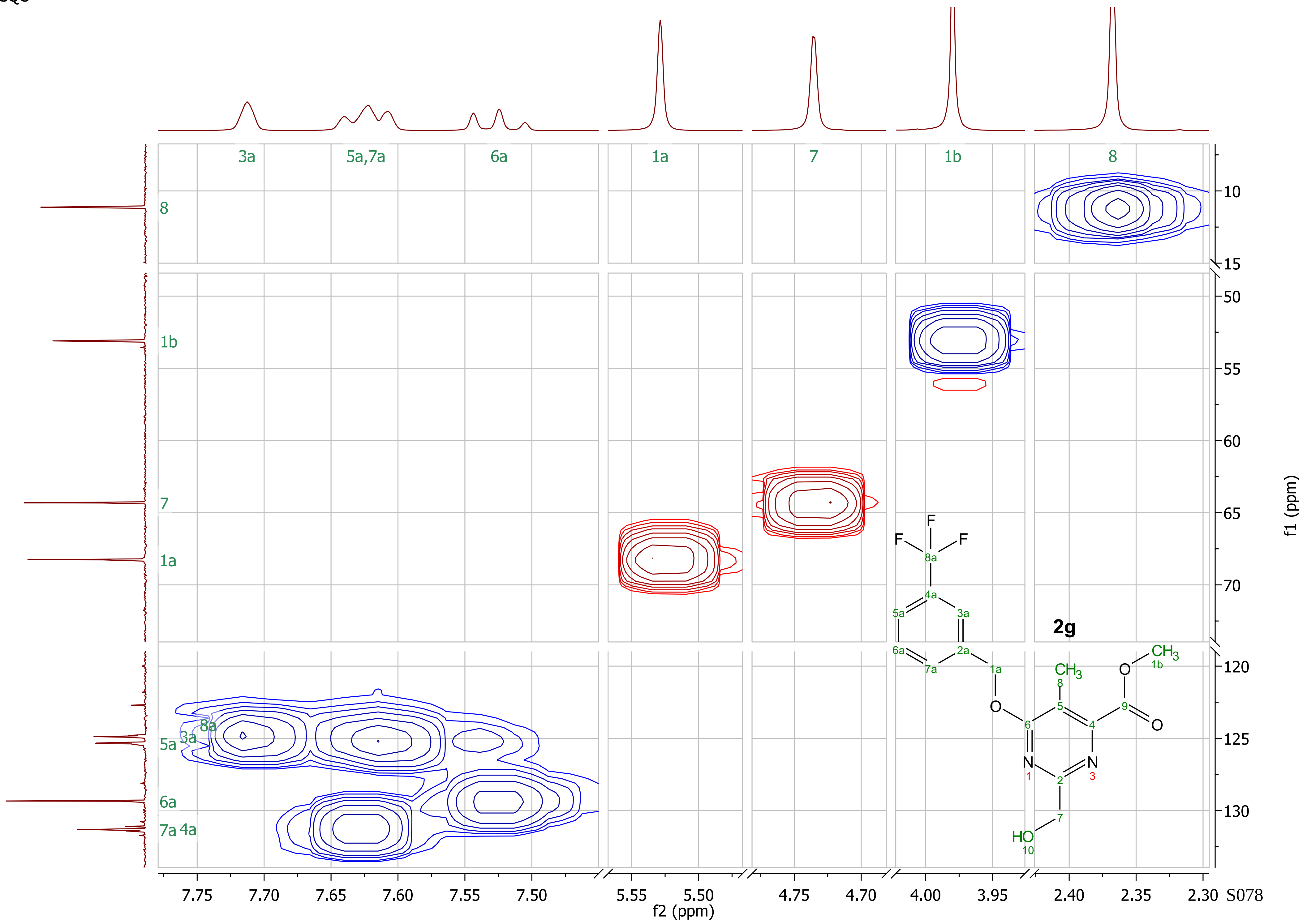

13C HMBC

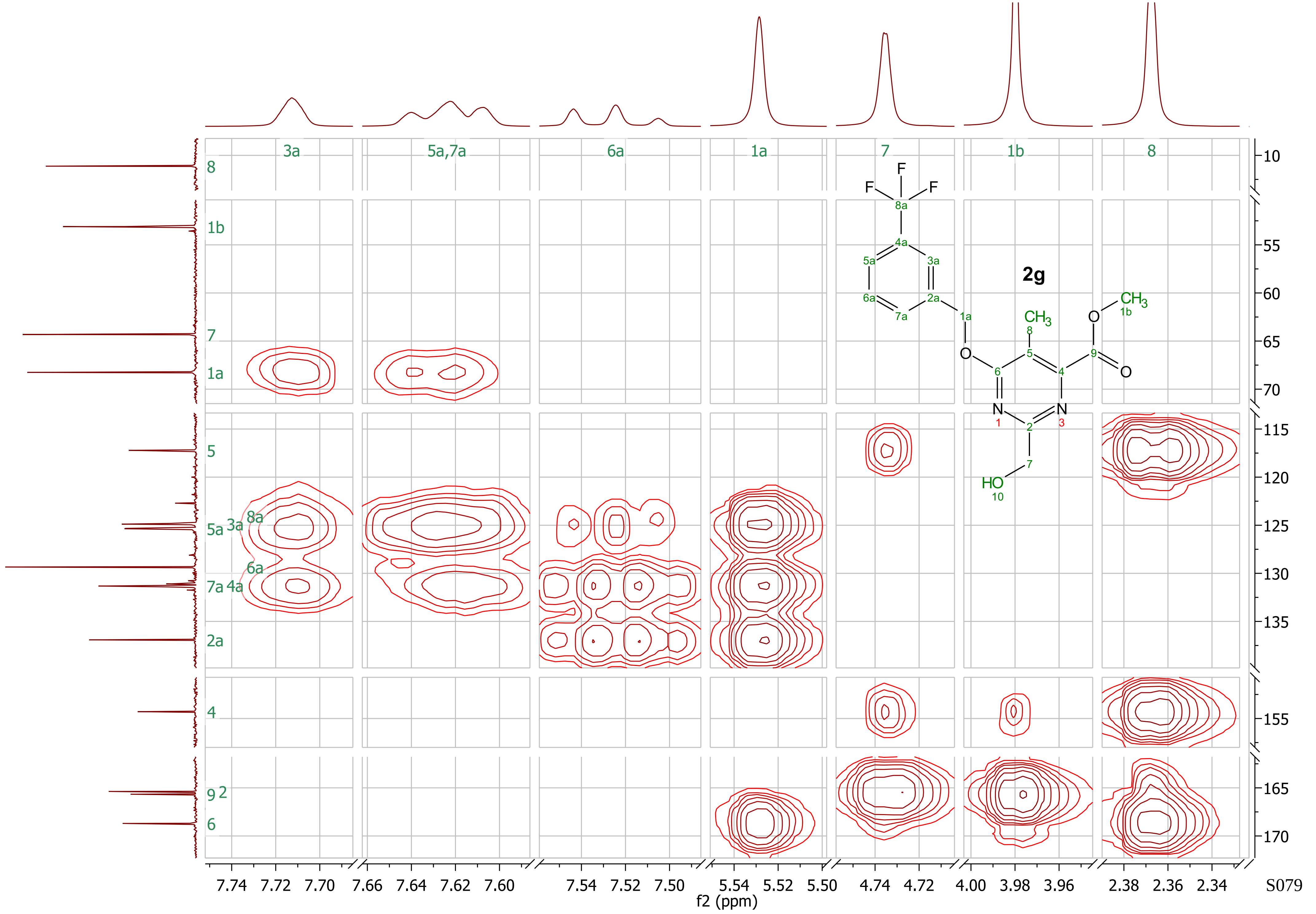

S079

^{1}H NMR (400 MHz, $CDCl_3$) δ 7.71 (s, 1H), 7.66 – 7.58 (m, 2H), 7.52 (t, *J* = 7.7 Hz, 1H), 5.52 (s, 2H), 5.13 (quint, *J* = 6.1 Hz, 1H), 4.71 (s, 2H), 3.30 (br s, 1H), 2.32 (s, 3H), 1.81 – 1.59 (m, 4H), 1.44 – 1.27 (m, 4H), 0.98 (t, *J* = 7.4 Hz, 3H), 0.91 (app t, *J* = 7.0 Hz, 3H).

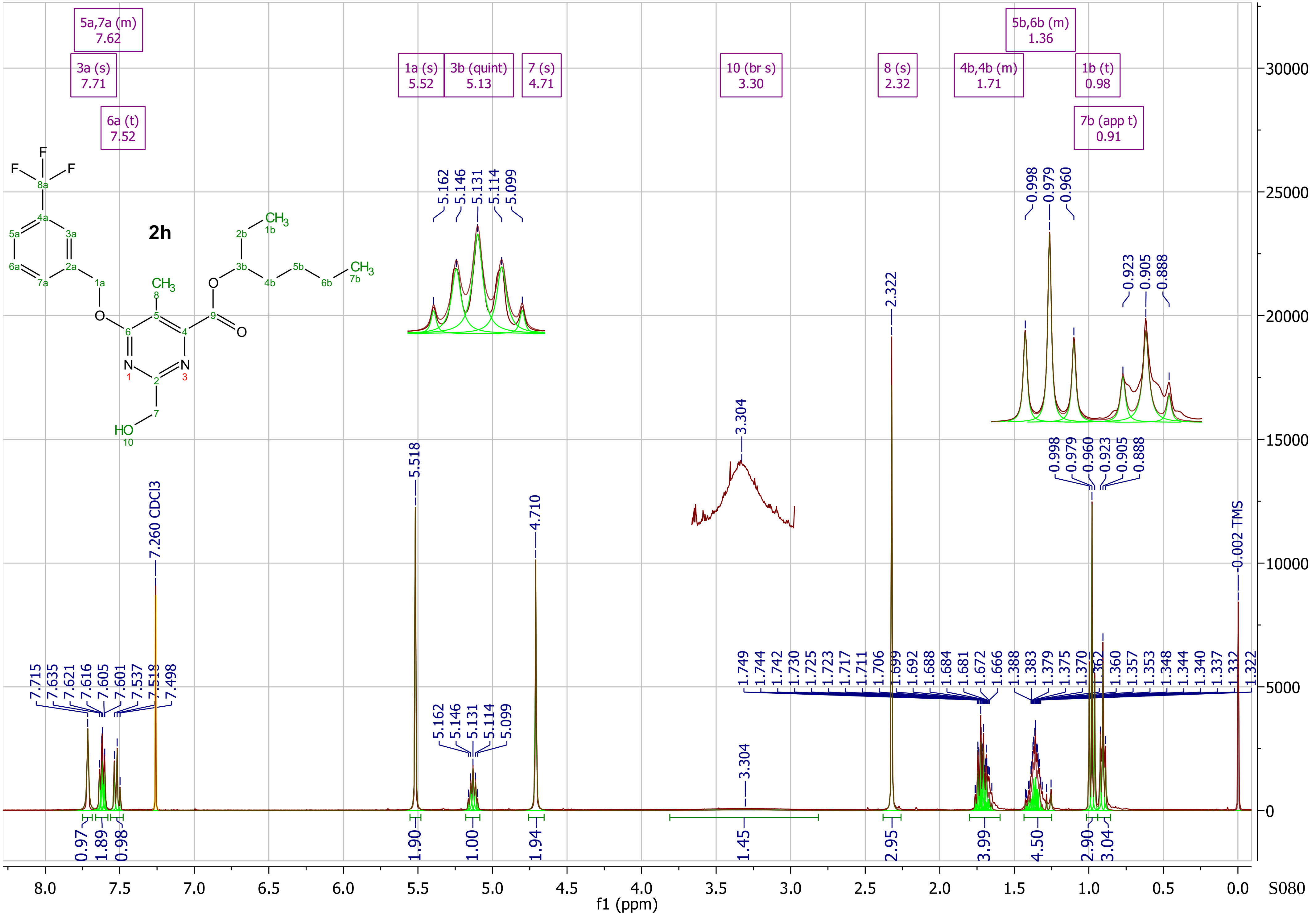

^{13}C NMR (101 MHz, $CDCl_3$) δ 168.5, 165.4, 165.3, 155.7, 137.0, 131.3 (app q, J = 1.3 Hz), 131.2 (q, J = 32.6 Hz), 129.3, 125.3 (q, J = 3.8 Hz), 124.9 (q, J = 3.8 Hz), 124.1 (q, J = 272.4 Hz), 115.7, 78.3, 68.1, 64.1, 33.4, 27.6, 27.1, 22.7, 14.1, 11.1, 9.8.

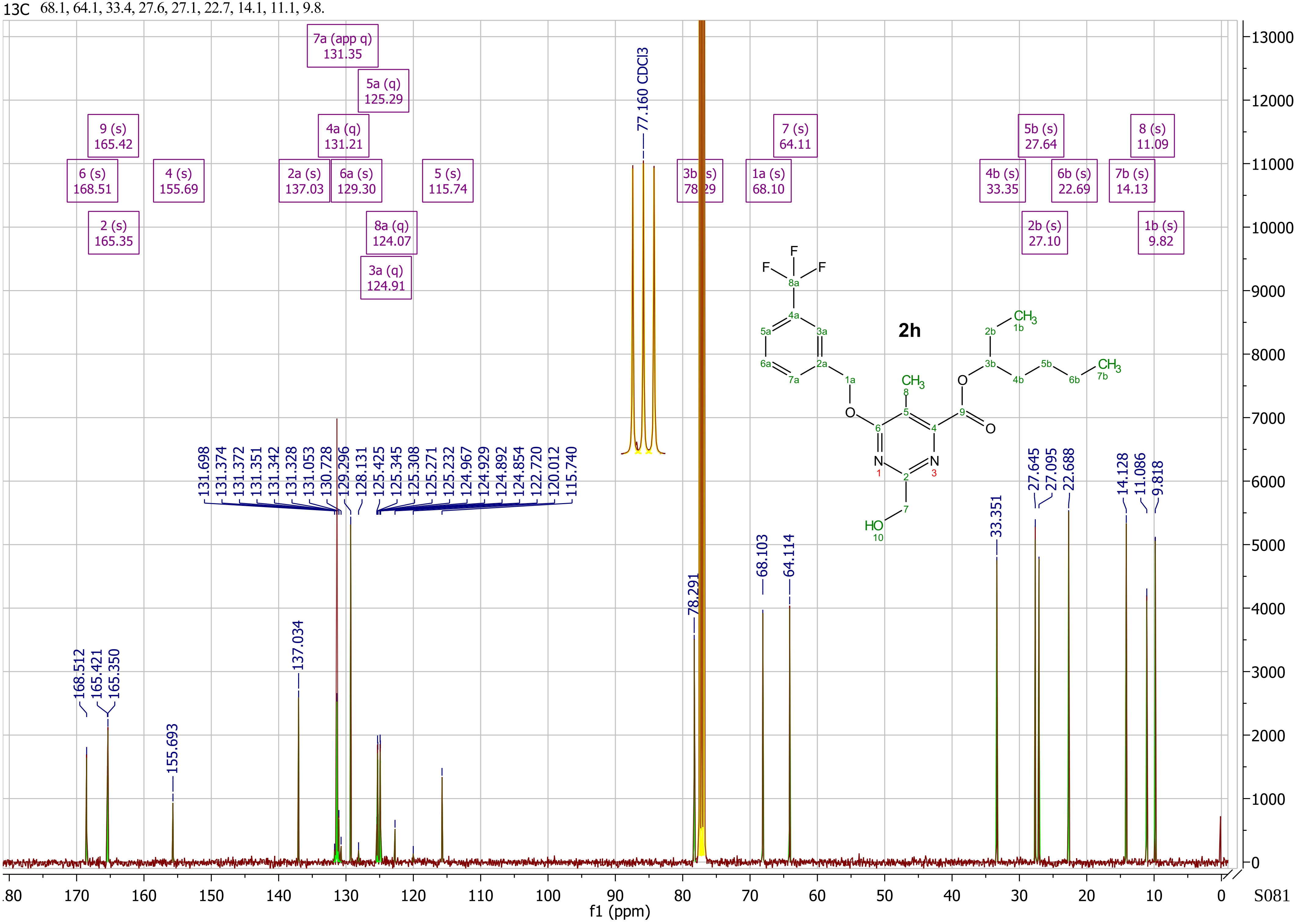

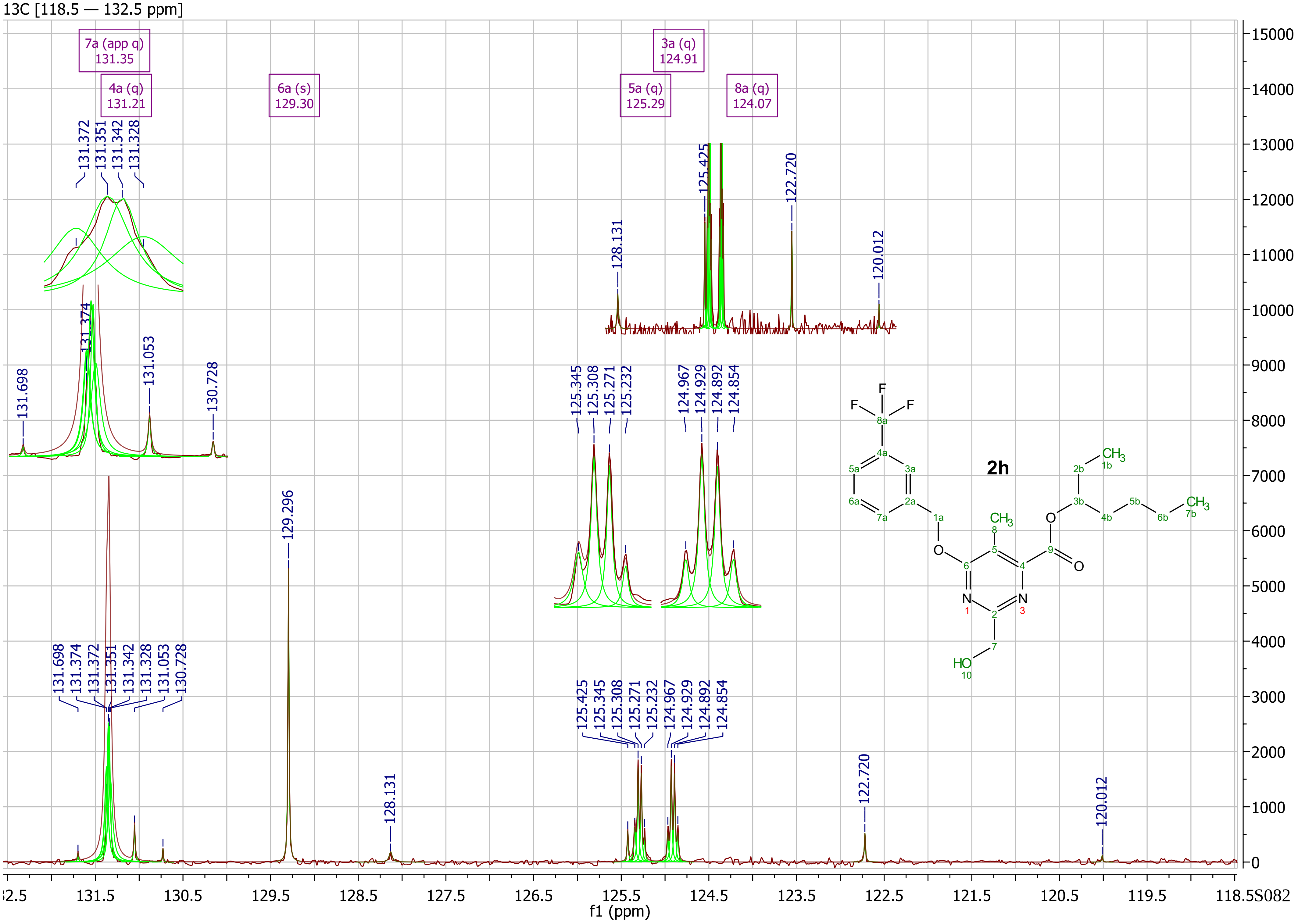
13C [118.5 — 132.5 ppm]
7a (app q)
131.35
4a (q)
131.21
6a (s)
129.30
3a (q)
124.91
5a (q)
125.29
8a (q)
124.07
131.372
131.351
131.342
131.328
131.698
131.374
131.053
130.728
129.296
128.131
125.425
125.345
125.308
125.271
125.232
124.967
124.929
124.892
124.854
122.720
120.012
2h
F
8a
4a
5a
3a
6a
2a
7a
1a
O
CH3
8
5
6
4
9
O
O
3b
2b
CH3
1b
4b
5b
6b
CH3
7b
N
1
2
N
3
7
HO
10
f1 (ppm)

S082

13C HSQC

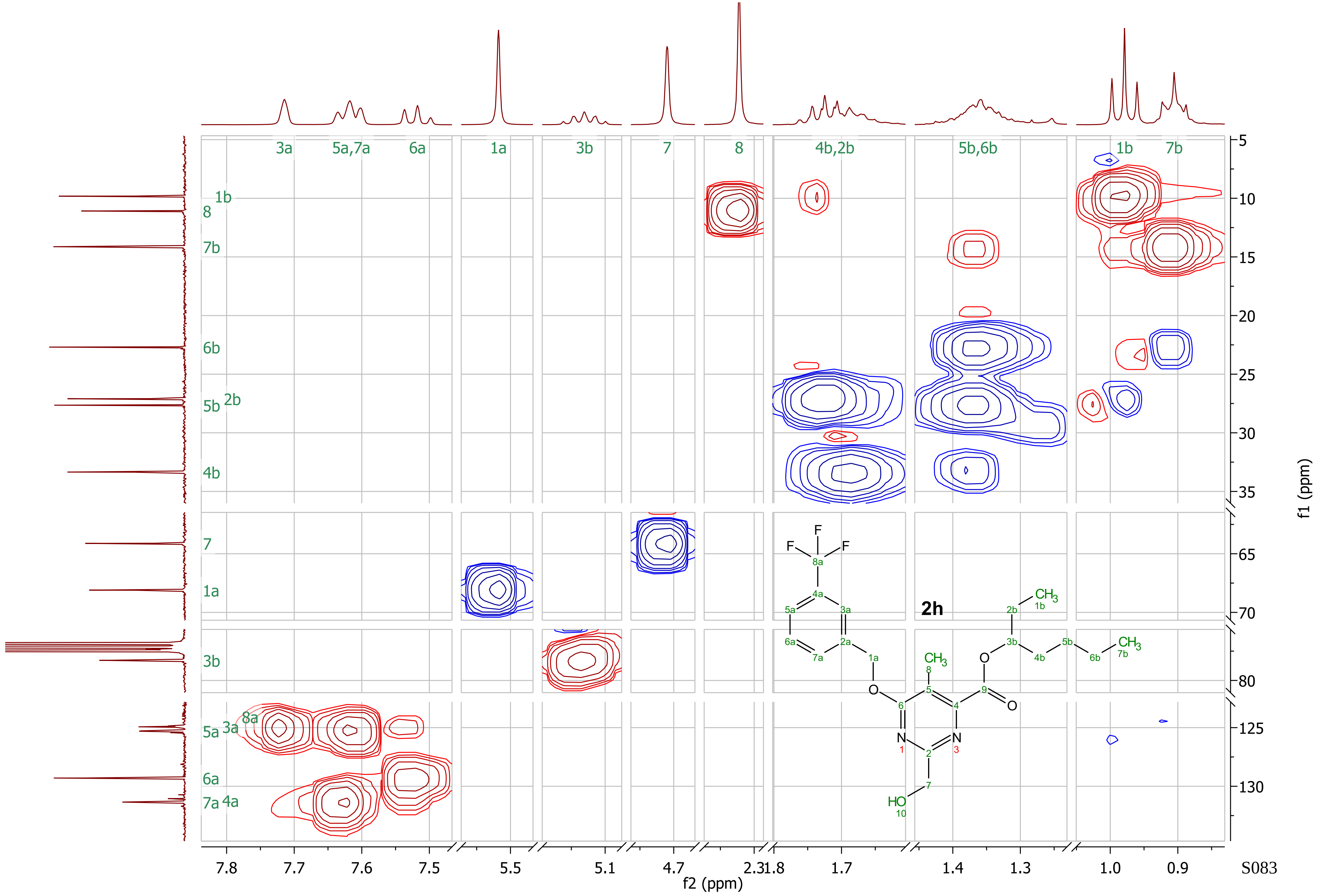

S083

13C HMBC

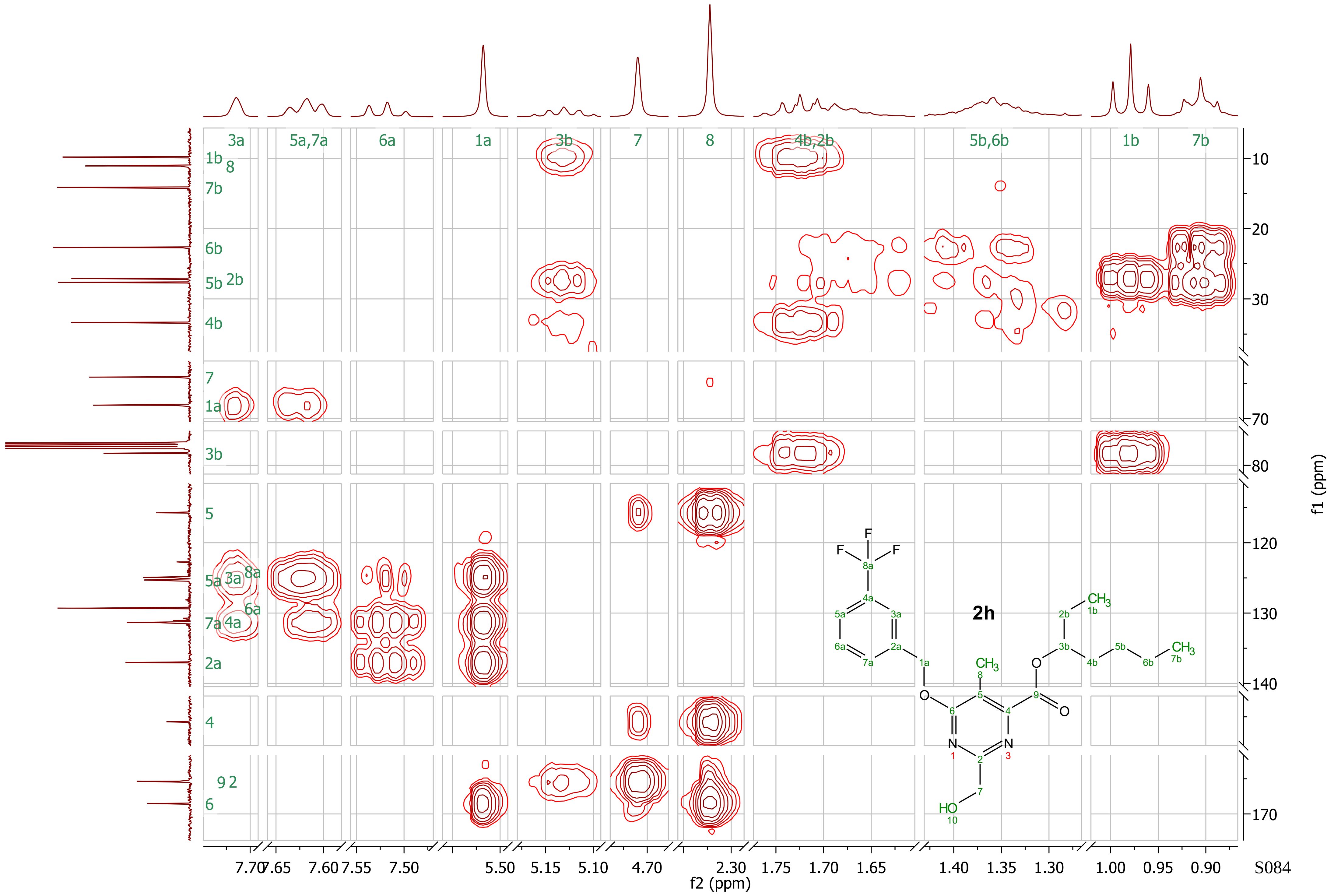

S084

1H ^{1}H NMR (400 MHz, $CDCl_3$) δ 5.12 (quint, *J* = 6.1 Hz, 1H), 4.67 (s, 2H), 4.39 (t, *J* = 6.6 Hz, 2H), 3.62 (br s, 1H), 2.26 (s, 3H), 1.84 – 1.60 (m, 6H), 1.50 – 1.21 (m, 12H), 0.98 (t, *J* = 7.4 Hz, 3H), 0.90 (app t, *J* = 7.0 Hz, 3H), 0.89 (app t, *J* = 6.9 Hz, 3H).

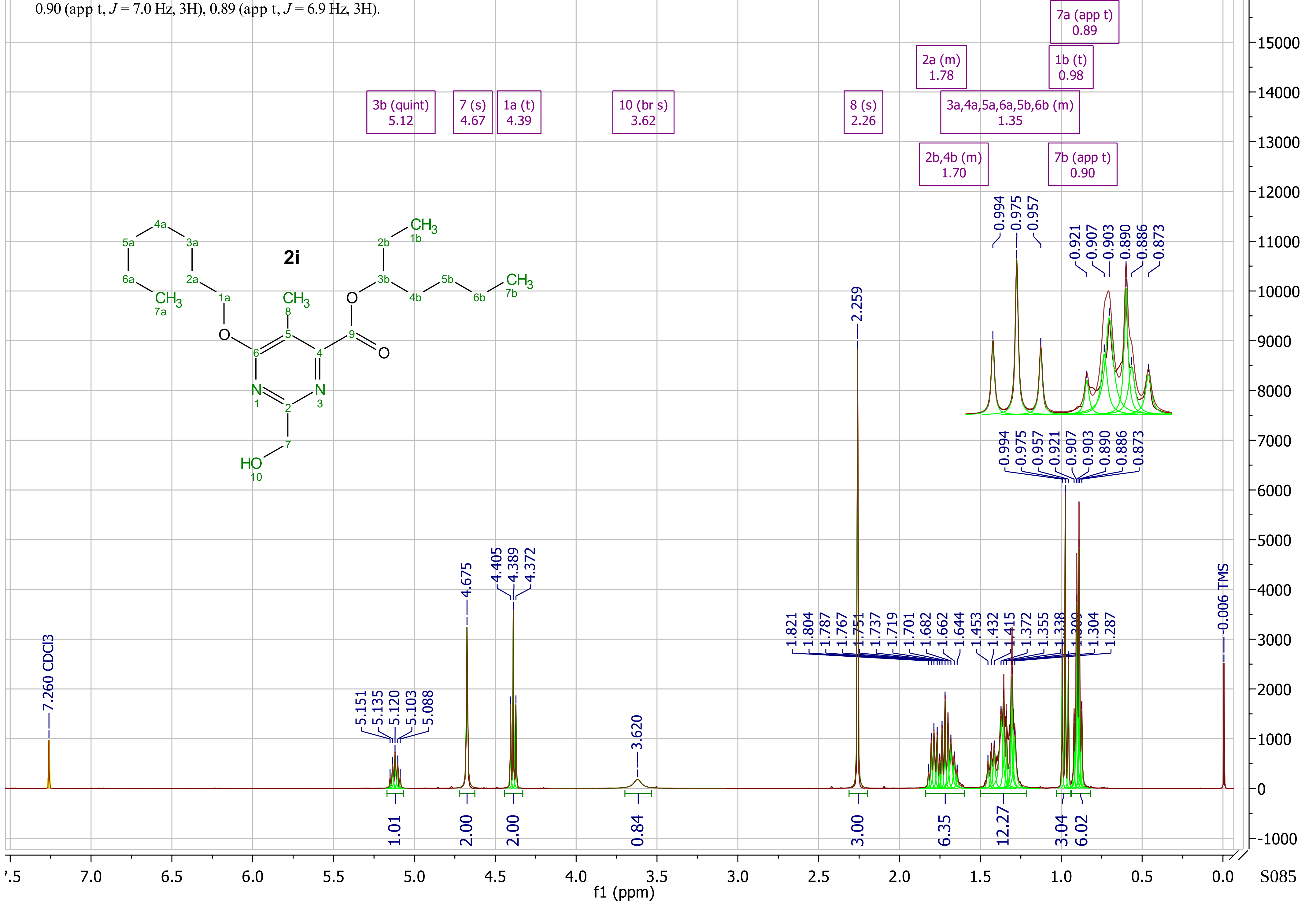

13C ^{13}C NMR (101 MHz, $CDCl_3$) δ 169.1, 165.7, 165.3, 155.2, 115.5, 78.1, 67.7, 64.1, 33.4, 31.9, 29.1, 28.8, 27.6, 27.1, 26.1, 22.73, 22.69, 14.2, 14.1, 11.0, 9.8.

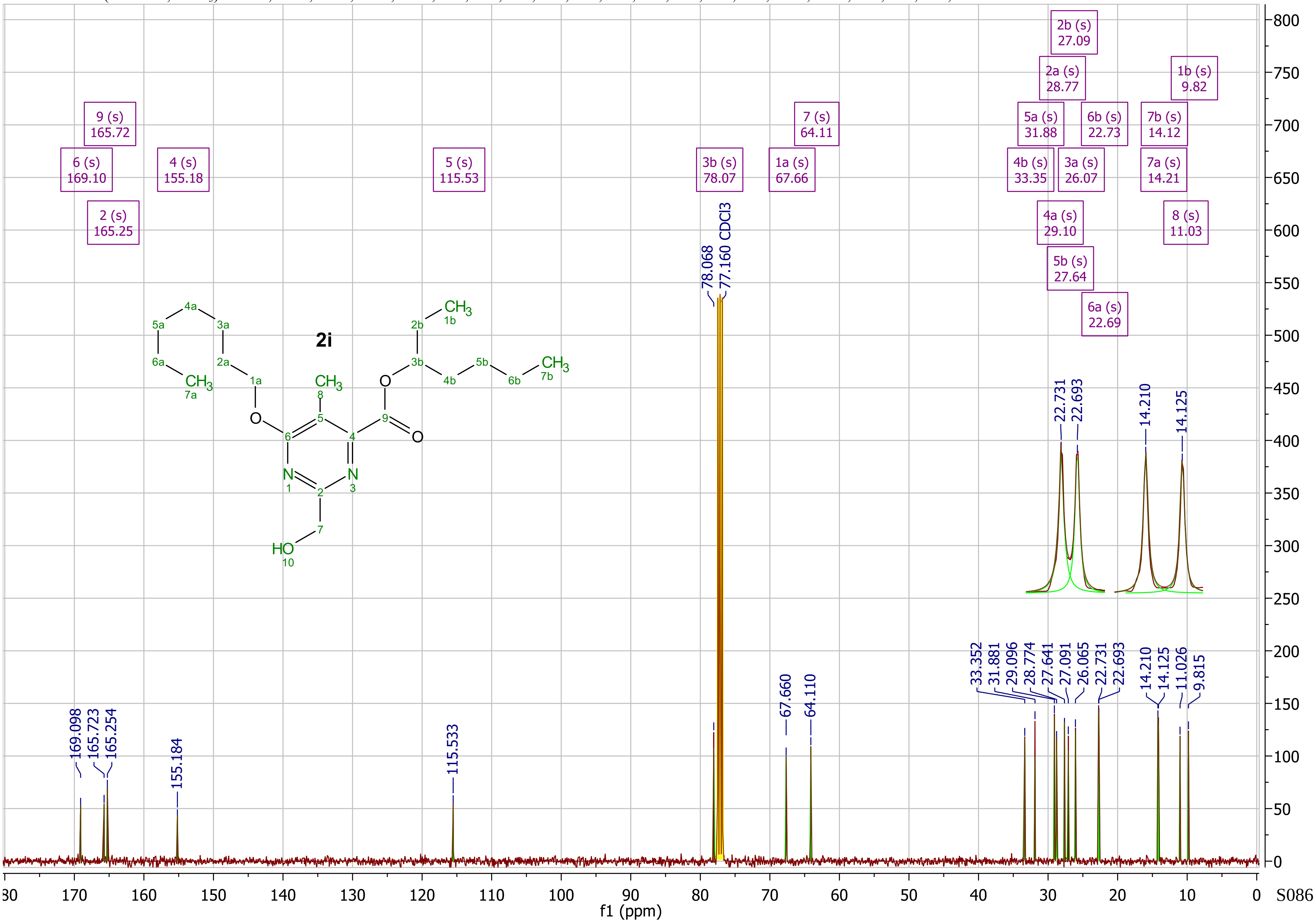

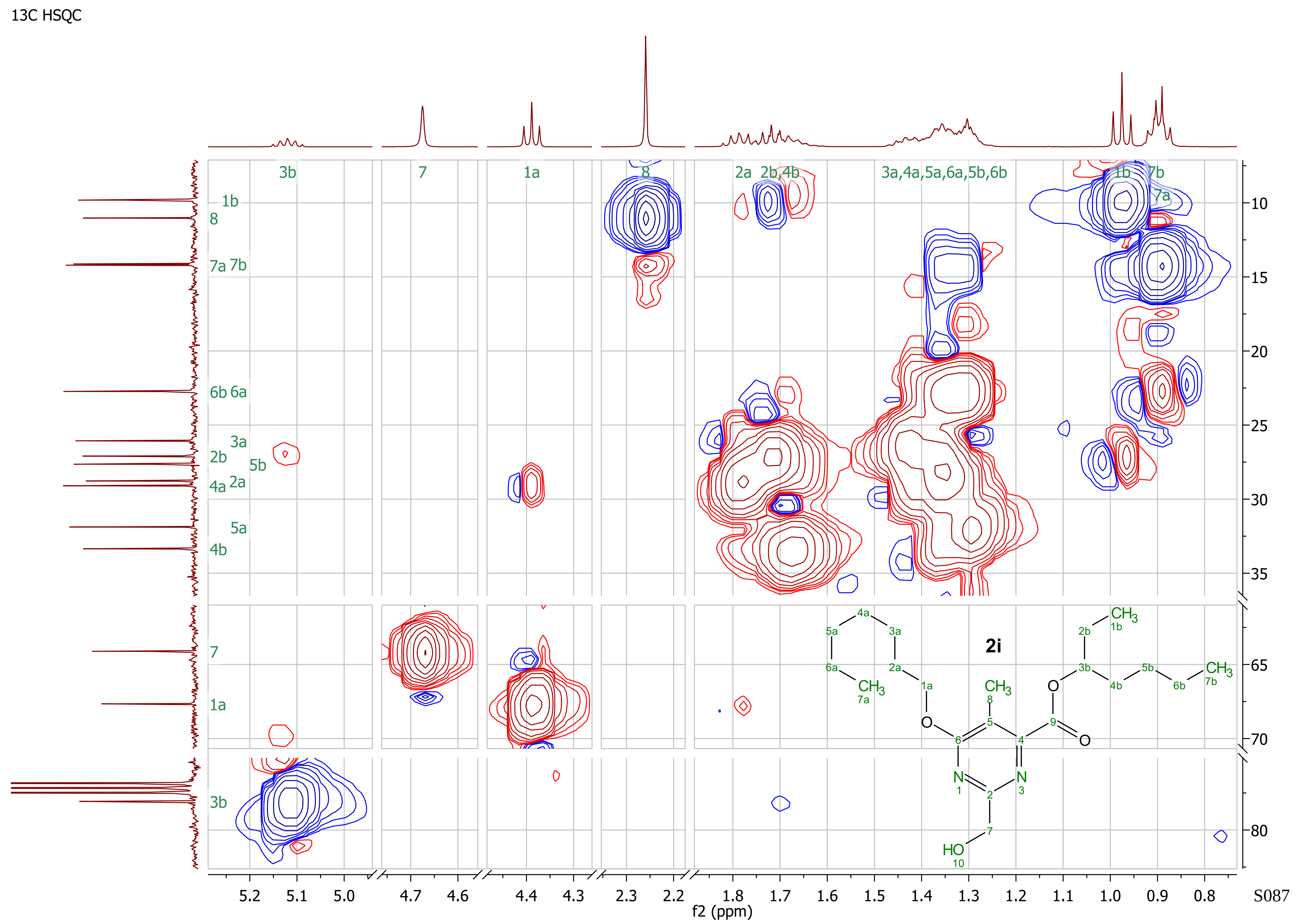
13C HSQC
3b
7
1a
8
2a 2b,4b
3a,4a,5a,6a,5b,6b
1b 7b
7a
1b
8
7a 7b
6b 6a
3a
2b
5b
4a 2a
5a
4b
7
1a
3b
2i
HO
f2 (ppm)
f1 (ppm)
S087

13C HMBC

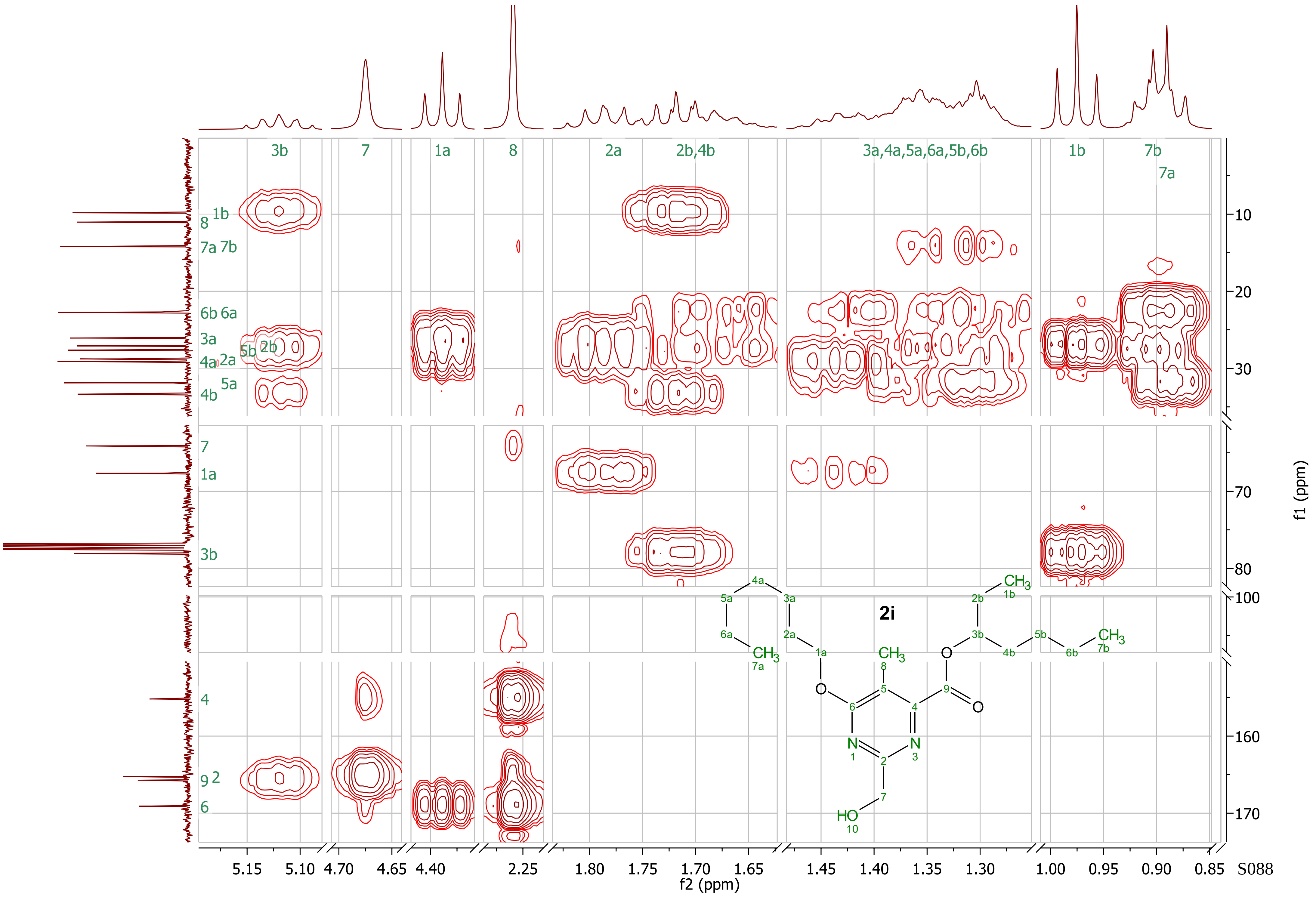
3b
7
1a
8
2a
2b,4b
3a,4a,5a,6a,5b,6b
1b
7b
7a
8 1b
7a 7b
6b 6a
3a
5b 2b
4a 2a
5a
4b
7
1a
3b
4
9 2
6
2i
f2 (ppm)
f1 (ppm)
S088

15N HMBC ^{15}N NMR (41 MHz, $CDCl_3$) δ 252.76, 243.69.

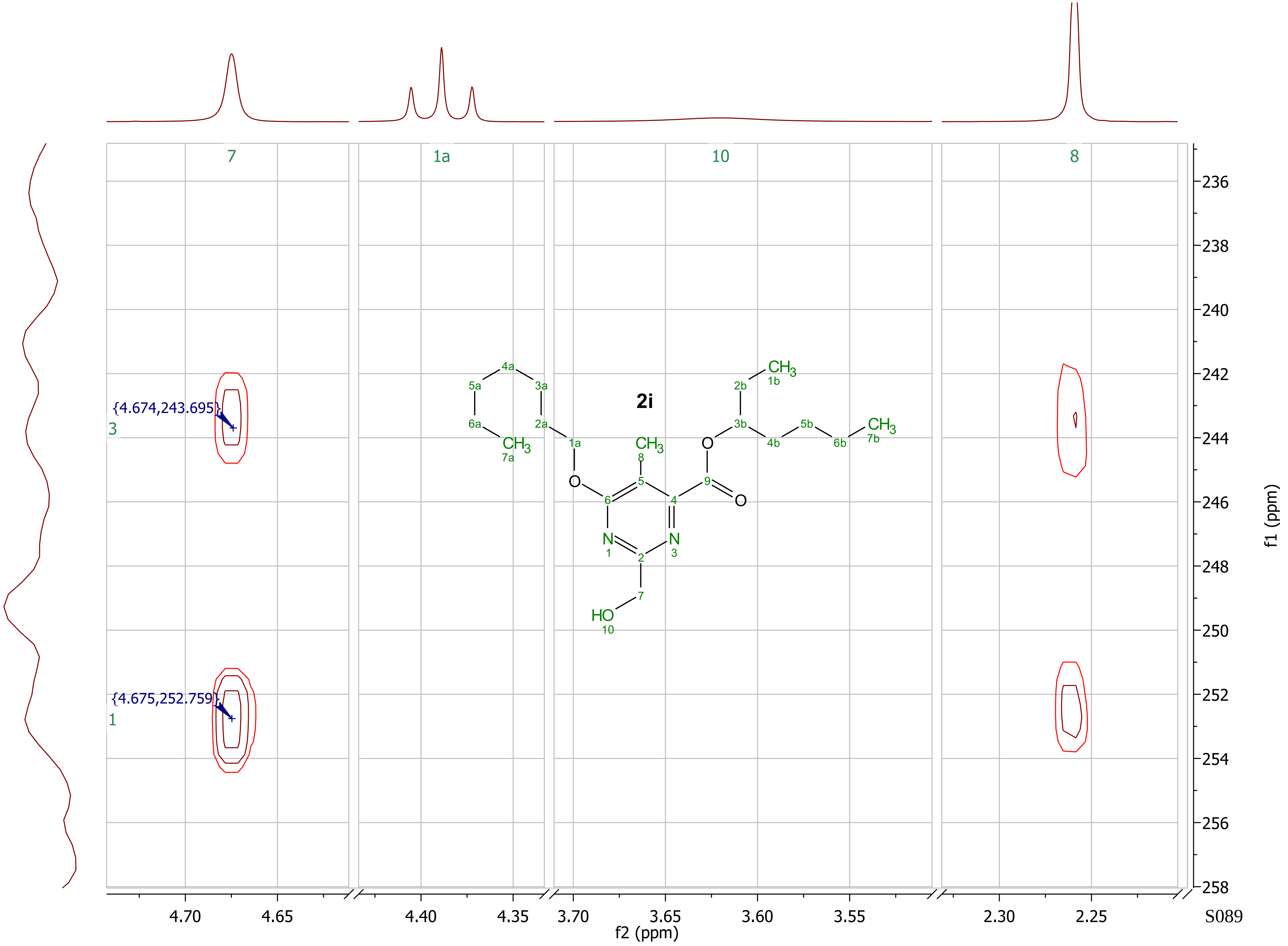

^{1}H NMR (400 MHz, $CDCl_3$) δ 7.71 (s, 1H), 7.64 (d, *J* = 7.6 Hz, 1H), 7.61 (d, *J* = 7.9 Hz, 1H), 7.52 (t, *J* = 7.7 Hz, 1H), 5.45 (s, 2H), 4.70 (s, 2H), 4.40 (t, *J* = 6.6 Hz, 2H), 3.50 (s, 1H), 2.27 (s, 3H),
1H 1.79 (quint, *J* = 6.7 Hz, 2H), 1.49 – 1.22 (m, 8H), 0.89 (app t, *J* = 6.9 Hz, 3H).

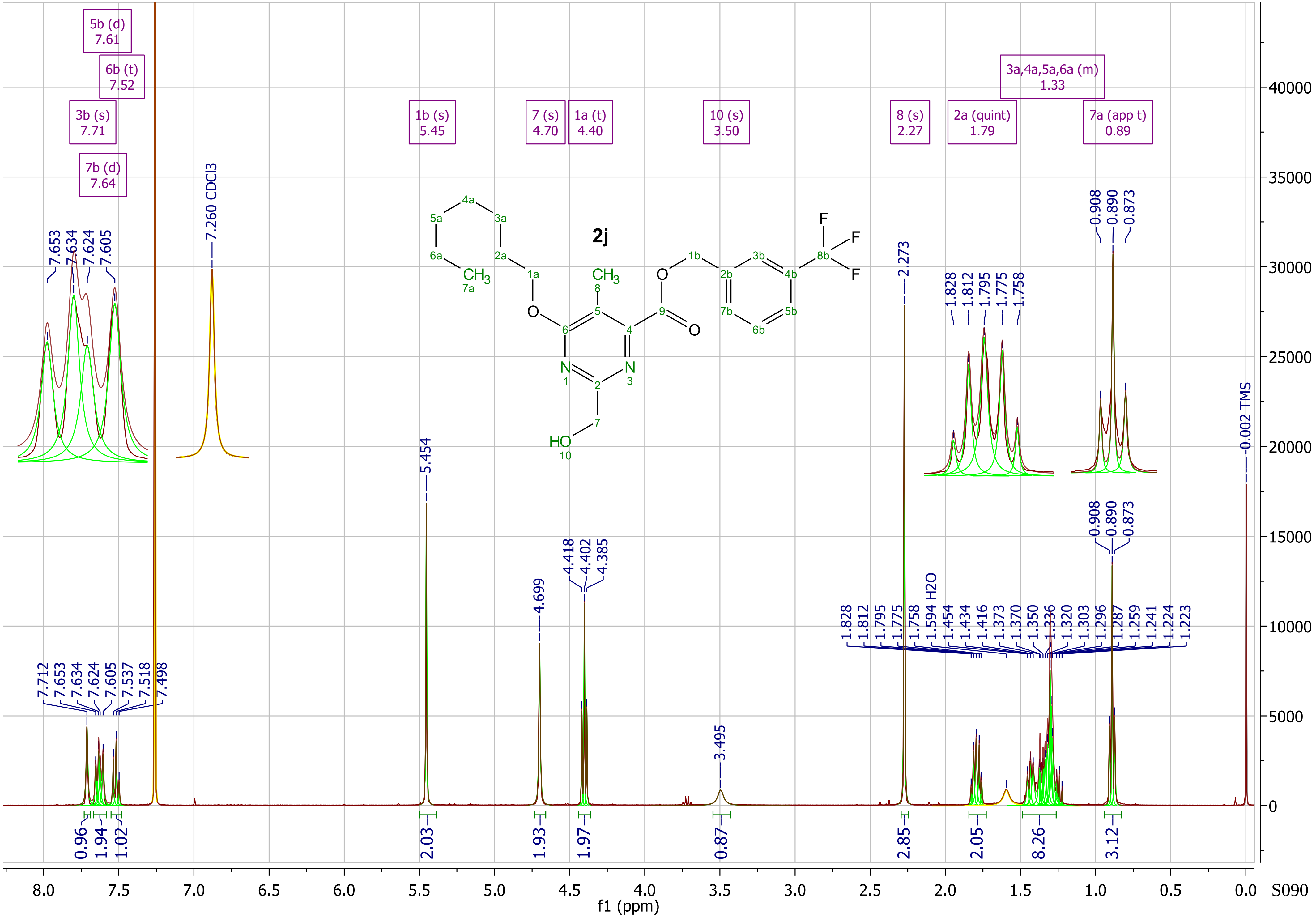

^{13}C NMR (101 MHz, $CDCl_3$) δ 169.3, 165.4, 165.2, 153.5, 136.3, 131.7 (app q, J = 1.3 Hz), 131.3 (q, J = 32.8 Hz), 129.4, 125.5 (q, J = 3.8 Hz), 125.1 (q, J = 3.9 Hz), 124.0 (q, J = 272.2 Hz), 117.1, 67.9, 66.7, 64.2, 31.9, 29.1, 28.7, 26.1, 22.7, 14.2, 11.1.

13C

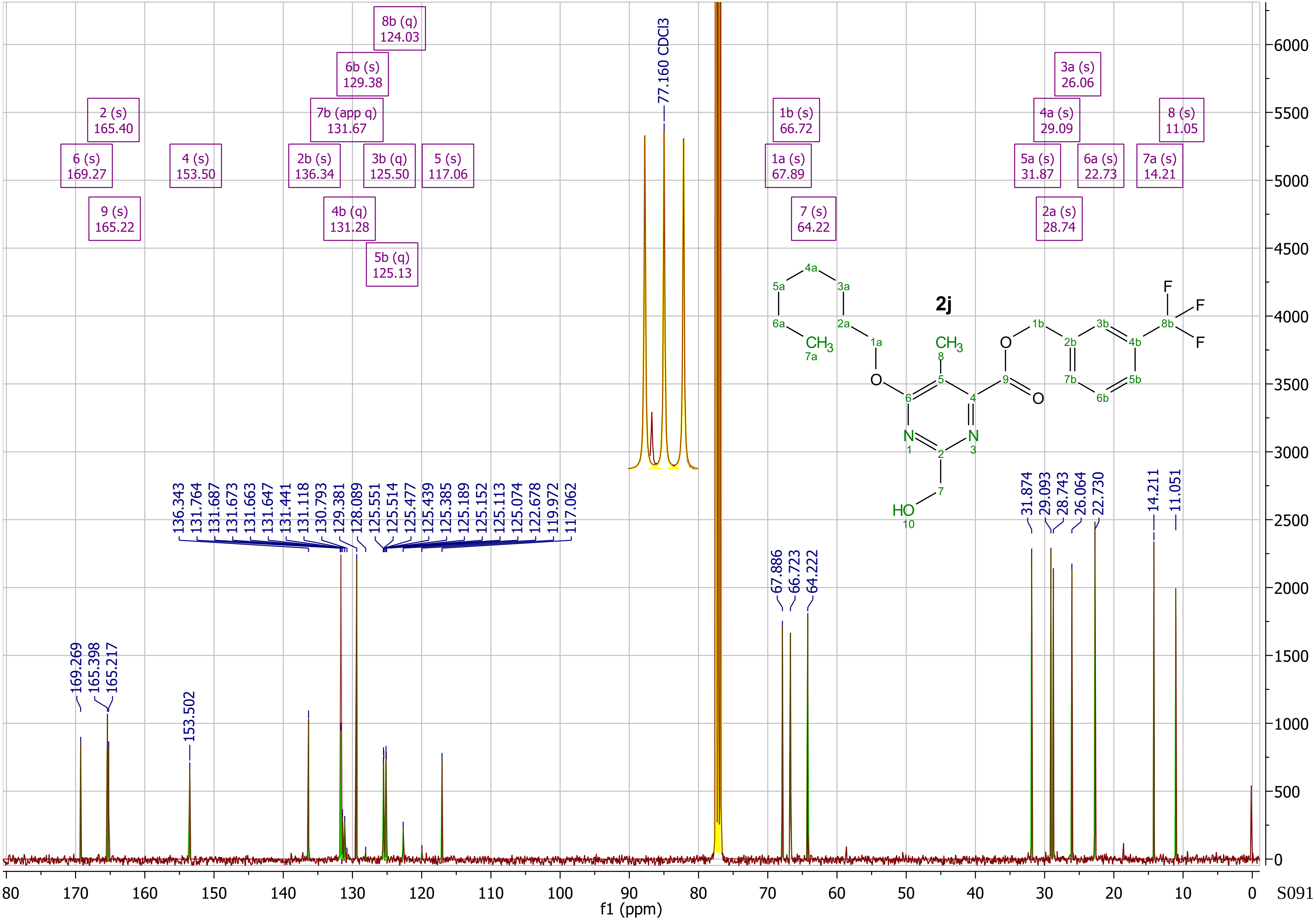

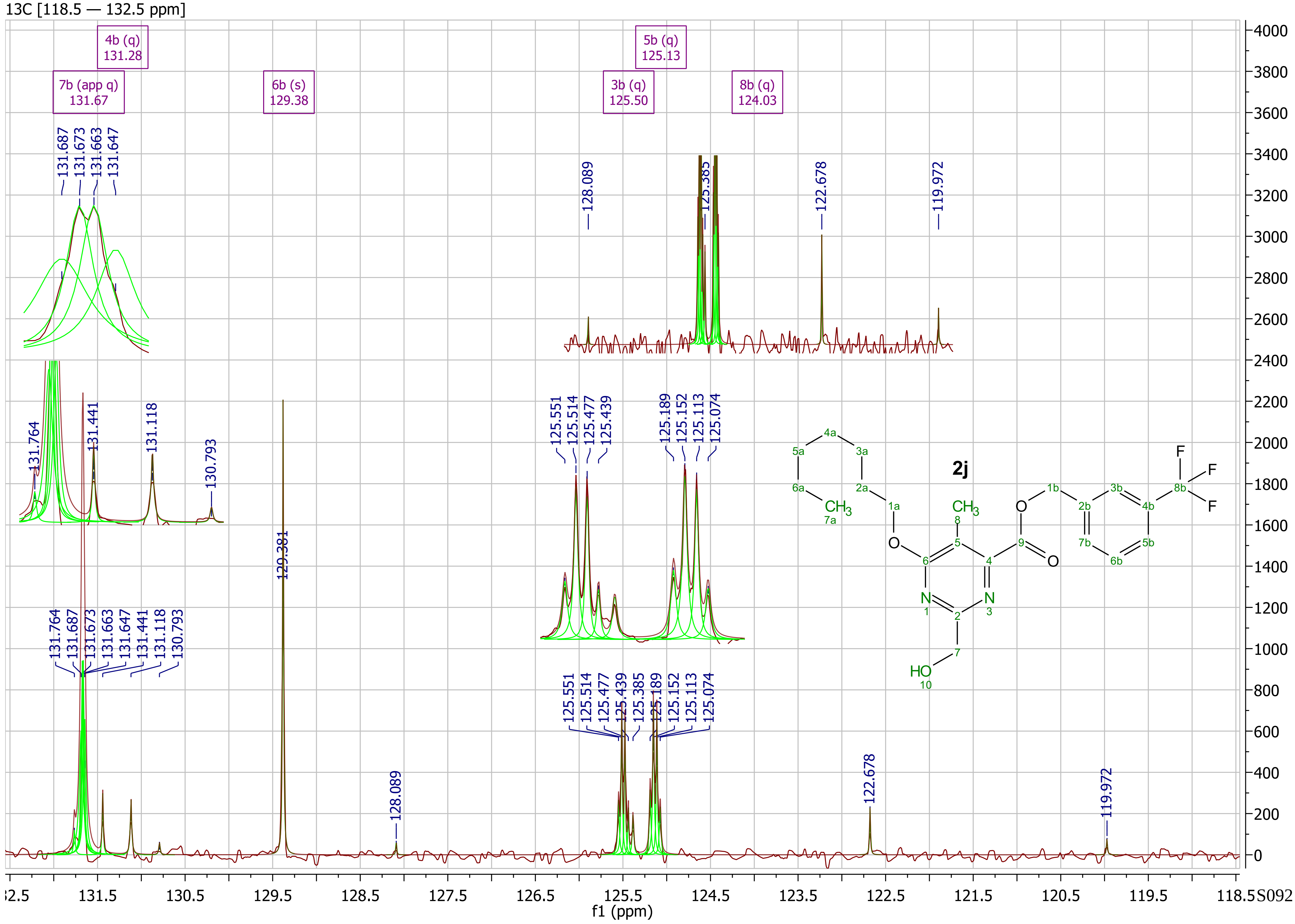
13C [118.5 — 132.5 ppm]
4b (q) 131.28
7b (app q) 131.67
6b (s) 129.38
5b (q) 125.13
3b (q) 125.50
8b (q) 124.03
131.687
131.673
131.663
131.647
128.089
125.385
122.678
119.972
131.764
131.441
131.118
130.793
125.551
125.514
125.477
125.439
125.189
125.152
125.113
125.074
129.381
2j
f1 (ppm)
S092

13 HSQC

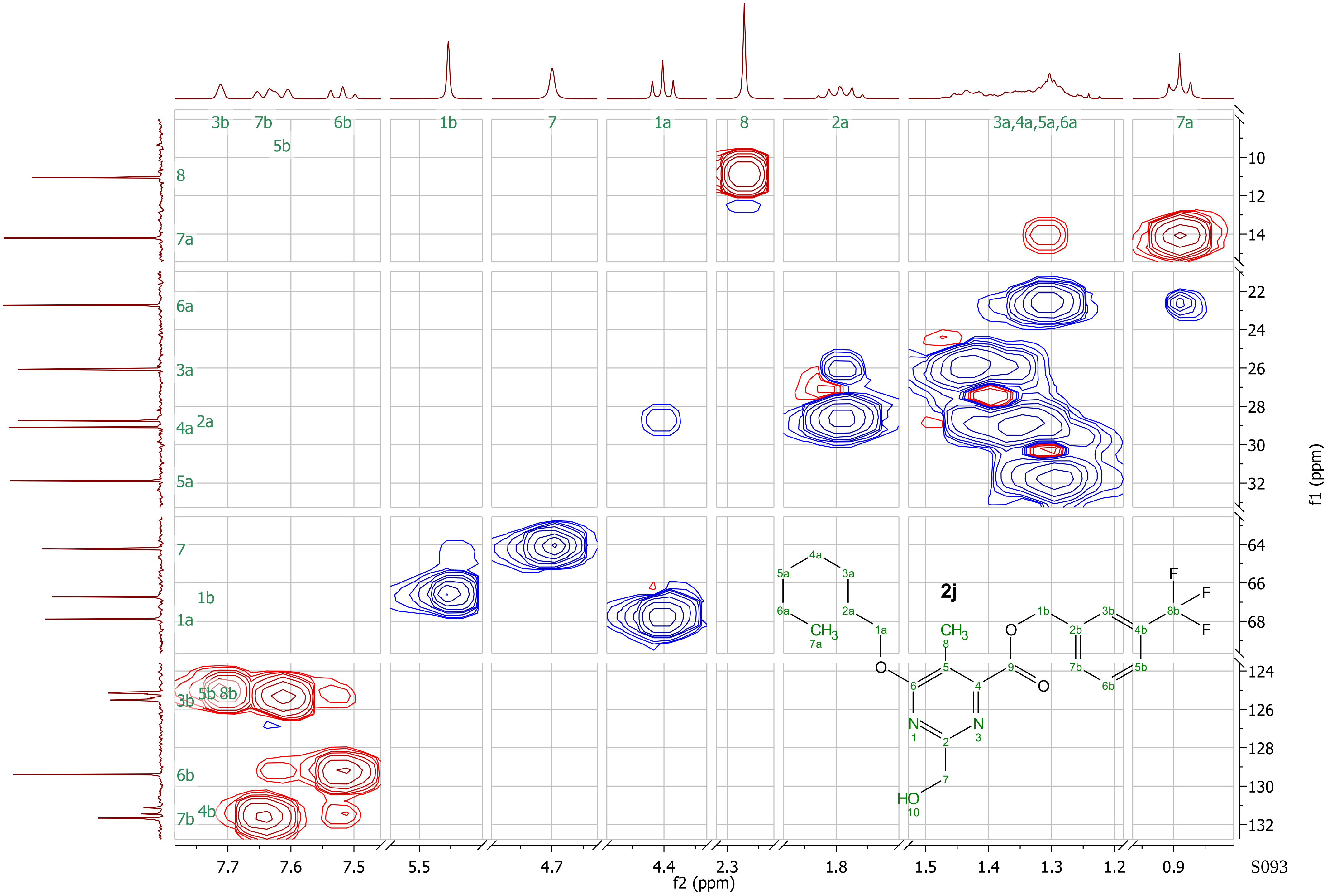

3b 7b 6b 1b 7 1a 8 2a 3a,4a,5a,6a 7a
5b
8
7a
6a
3a
4a 2a
5a
7
1b
1a
3b 5b 8b
6b
7b 4b
2j
f1 (ppm)
f2 (ppm)

S093

13 HMBC

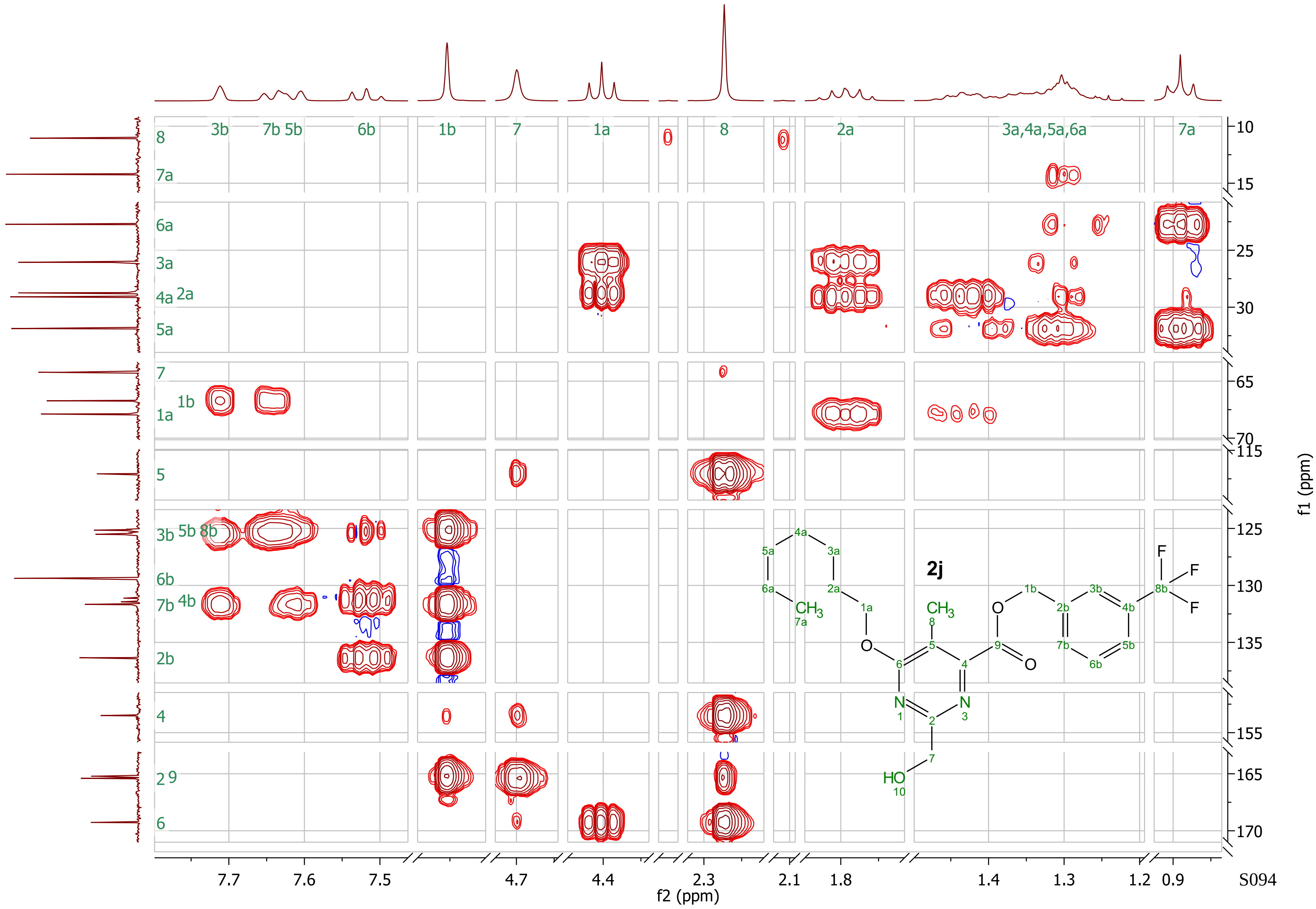

3b
7b 5b
6b
1b
7
1a
8
2a
3a,4a,5a,6a
7a
8
7a
6a
3a
4a 2a
5a
7
1b
1a
5
3b 5b 8b
6b
7b 4b
2b
4
2 9
6
2j
f1 (ppm)
f2 (ppm)
S094

15N HMBC ^{15}N NMR (41 MHz, $CDCl_3$) δ 253.62, 244.66.

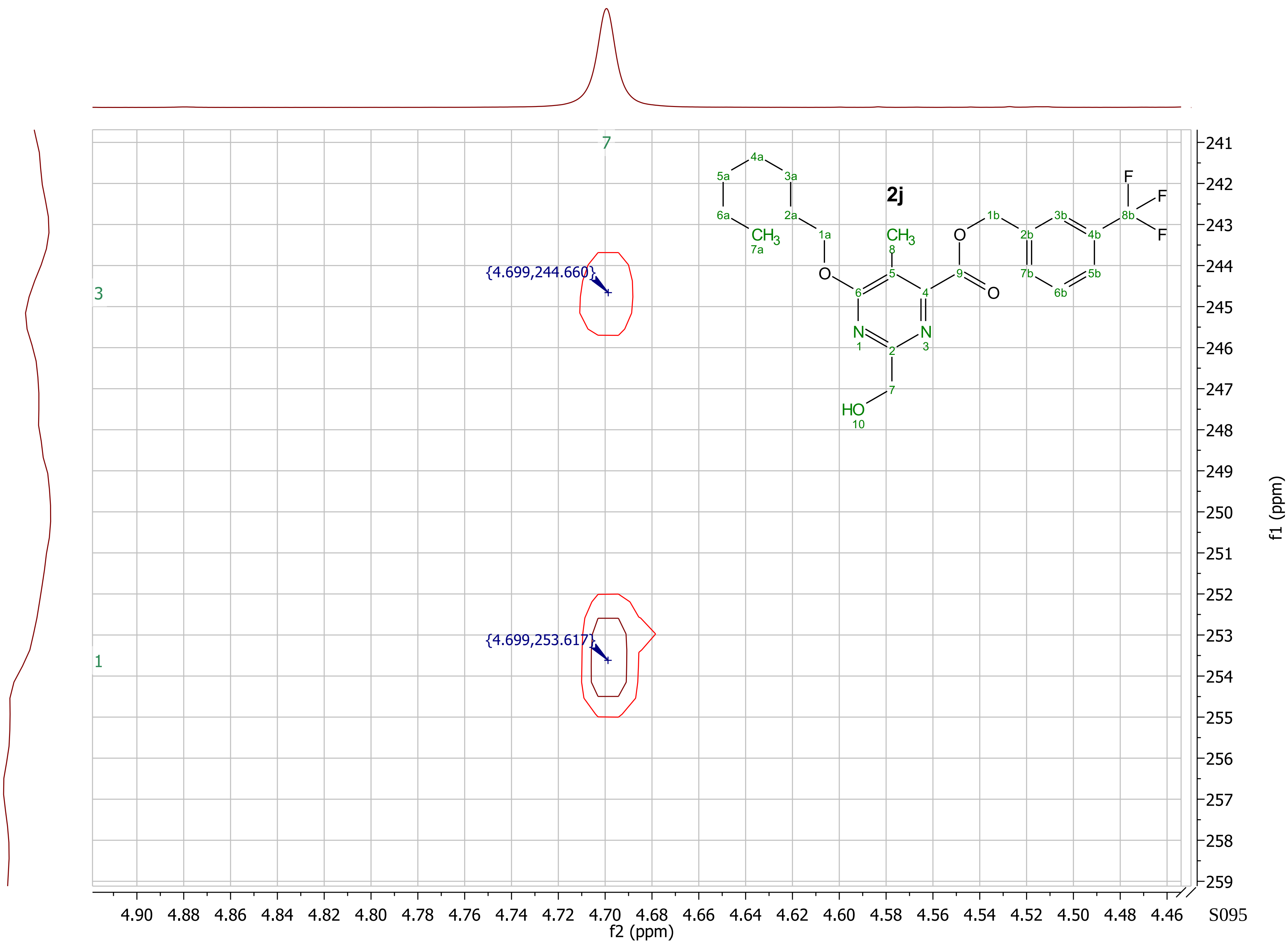

1H ^{1}H NMR (400 MHz, $CDCl_3$) δ 5.26 (quint, J = 5.8 Hz, 1H), 4.68 (s, 2H), 3.96 (s, 3H), 3.45 (br s, 1H), 2.29 (s, 3H), 1.76 – 1.60 (m, 4H), 1.39 – 1.20 (m, 4H), 0.91 (t, J = 7.4 Hz, 3H), 0.88 (app t, J = 7.1 Hz, 3H).

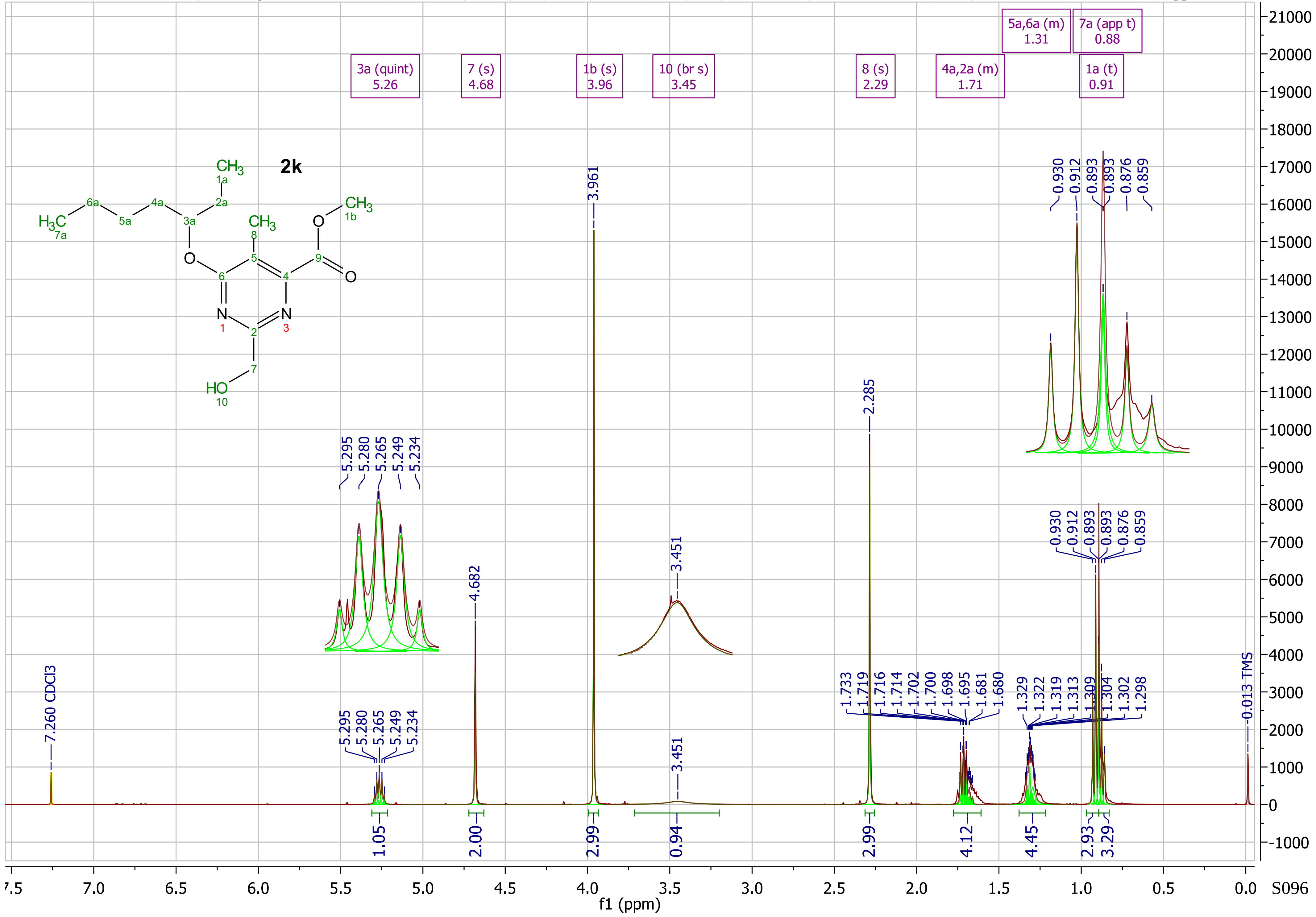

13C ^{13}C NMR (101 MHz, $CDCl_3$) δ 169.3, 166.0, 165.1, 153.9, 117.1, 78.7, 64.3, 53.0, 33.0, 27.5, 26.7, 22.7, 14.1, 11.1, 9.6.

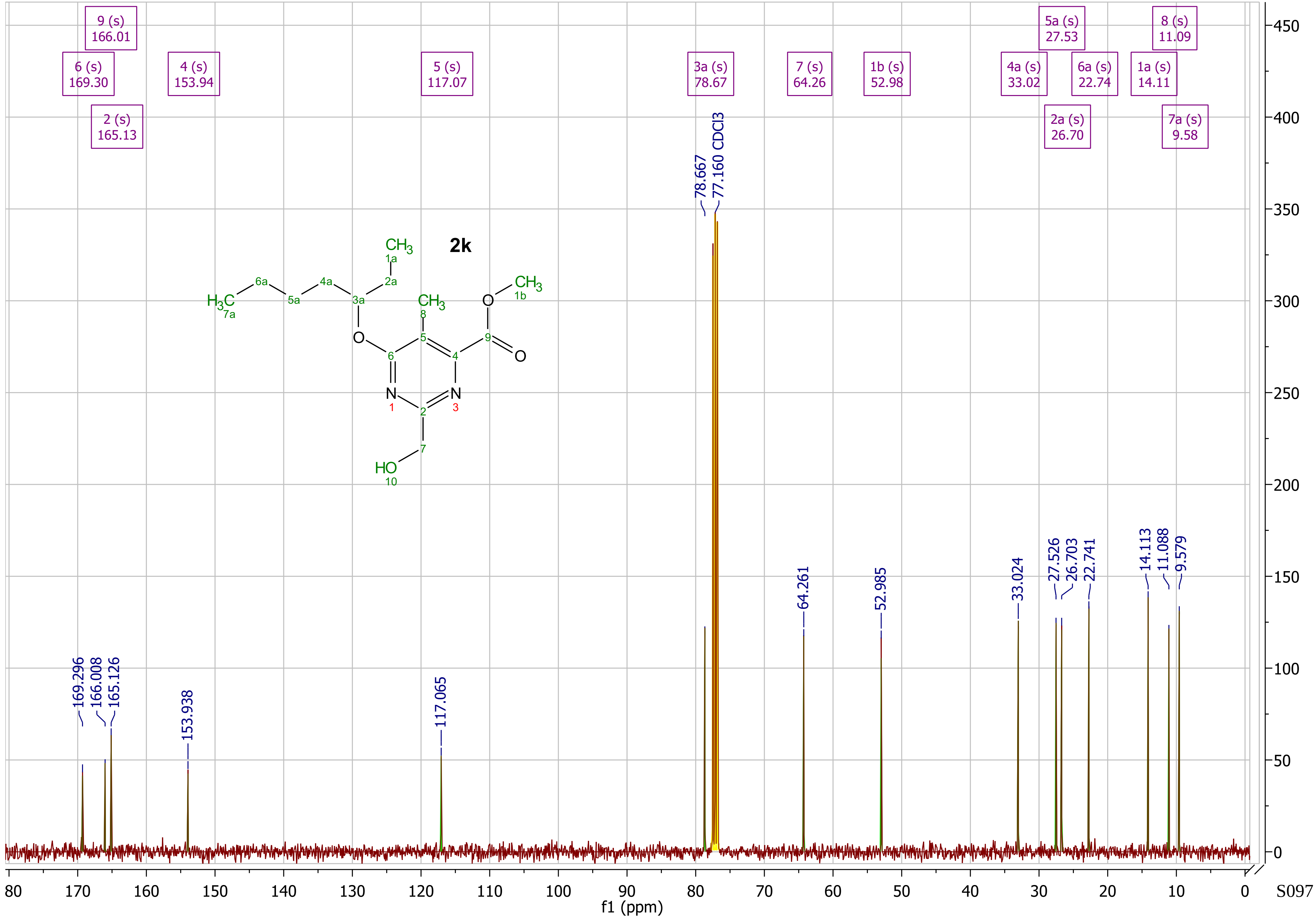

13C HSQC

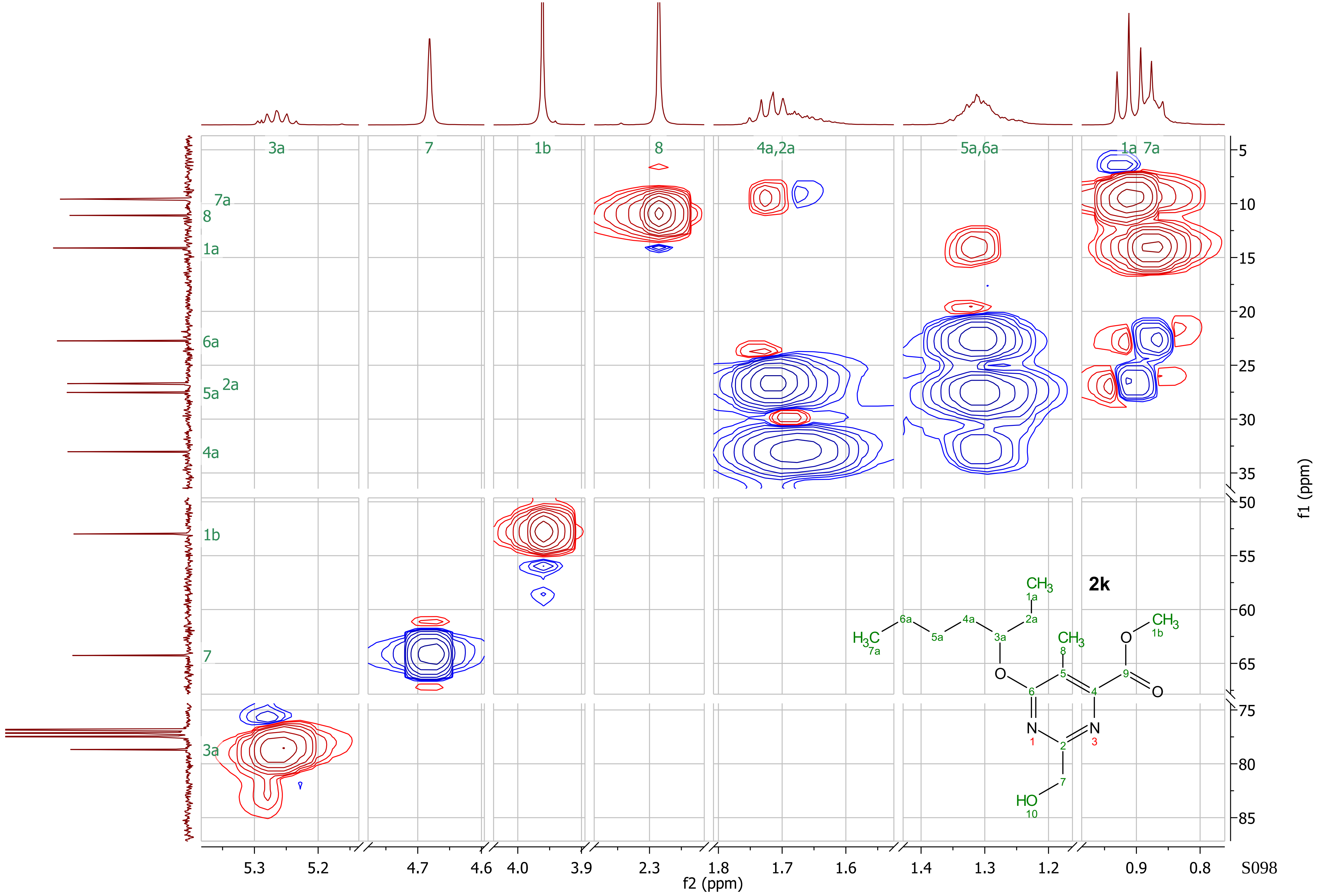

3a
7
1b
8
4a,2a
5a,6a
1a 7a
7a
8
1a
6a
2a
5a
4a
1b
7
3a
2k
f1 (ppm)
f2 (ppm)
S098

13C HMBC

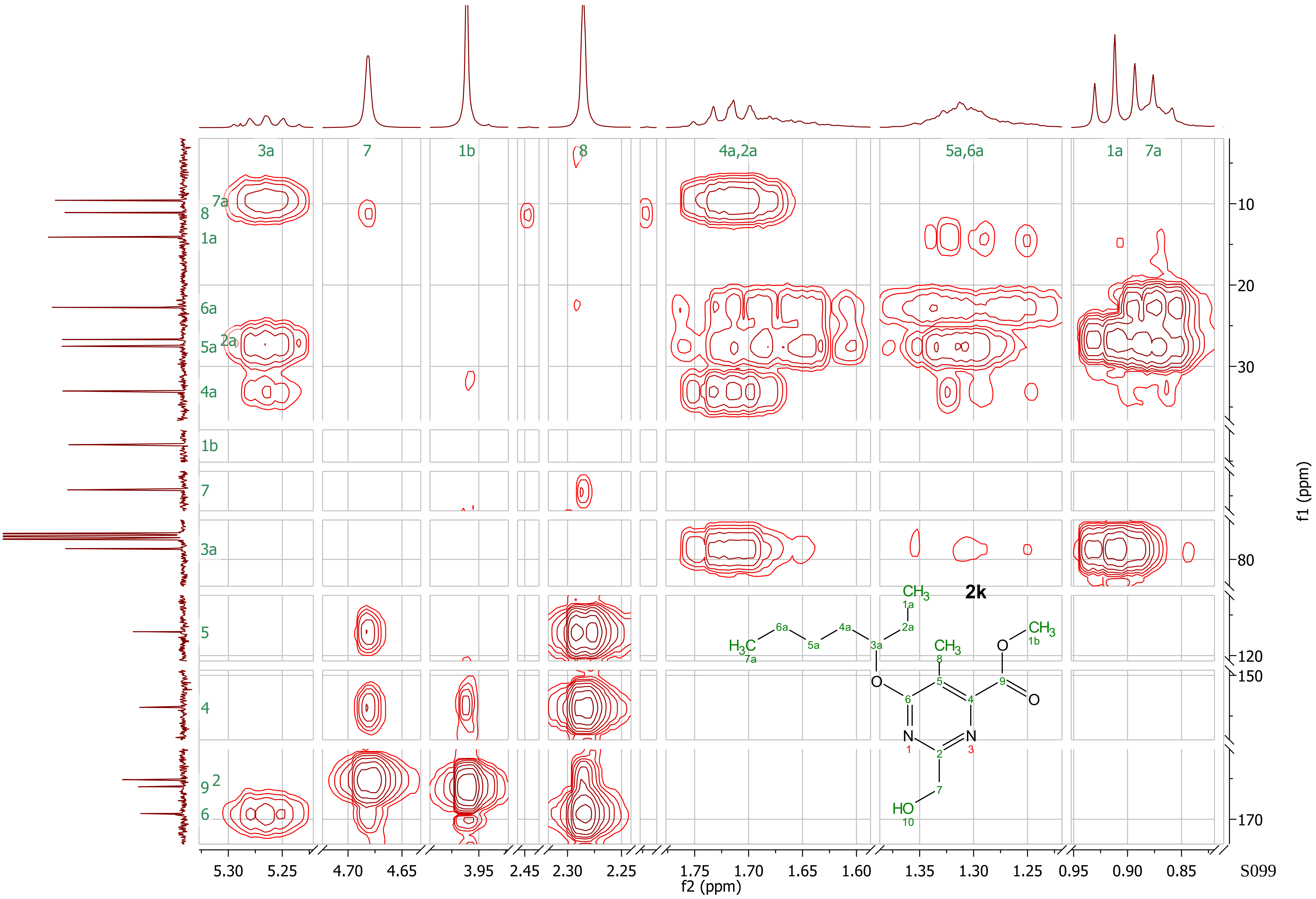

3a
7
1b
8
4a,2a
5a,6a
1a
7a
8
1a
6a
5a
2a
4a
1b
7
3a
5
4
9
2
6
f2 (ppm)
f1 (ppm)
2k
CH3
1a
2a
6a
4a
5a
3a
H3C
7a
CH3
8
O
1b
O
9
O
5
6
4
N
N
1
3
2
7
HO
10

^{1}H NMR (400 MHz, $CDCl_3$) δ 7.72 (s, 1H), 7.65 (d, J = 7.7 Hz, 1H), 7.61 (d, J = 7.8 Hz, 1H), 7.52 (t, J = 7.8 Hz, 1H), 5.46 (s, 2H), 5.27 (quint, J = 5.9 Hz, 1H), 4.68 (s, 2H), 3.02 (br s, 1H), 2.26 (s, 3H), 1.82 – 1.52 (m, 4H), 1.42 – 1.17 (m, 4H), 0.91 (t, J = 7.1 Hz, 3H), 0.88 (app t, J = 7.1 Hz, 3H).

1H

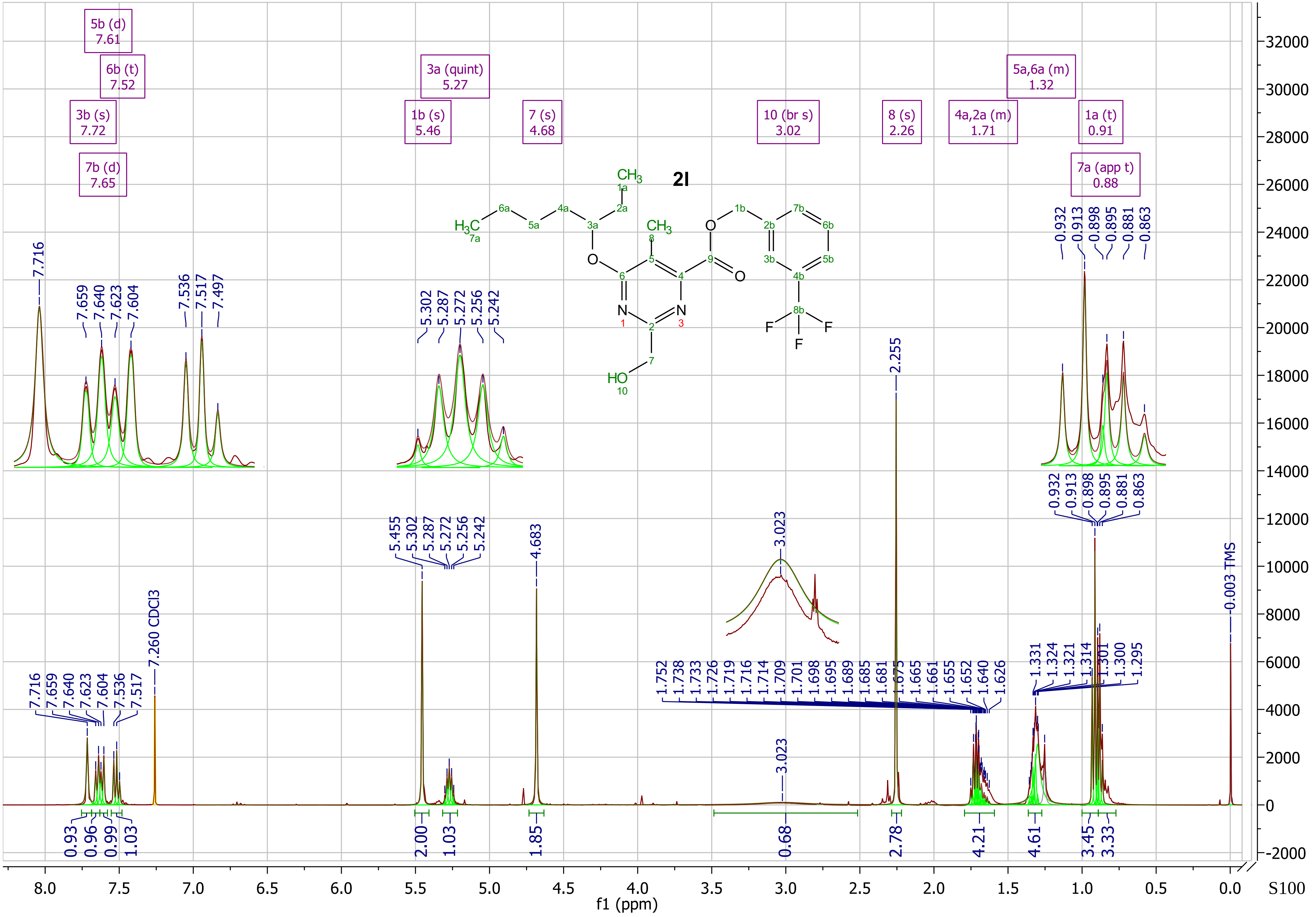

^{13}C NMR (101 MHz, $CDCl_3$) δ 169.3, 165.3 (2C), 153.6, 136.3, 131.7 (app q, J = 1.1 Hz), 131.3 (q, J = 32.4 Hz), 129.4, 125.5 (q, J = 3.8 Hz), 125.2 (q, J = 3.8 Hz), 124.0 (q, J = 272.3 Hz), 117.1, 78.8, 66.7, 64.2, 33.0, 27.5, 26.7, 22.7, 14.1, 11.1, 9.6.

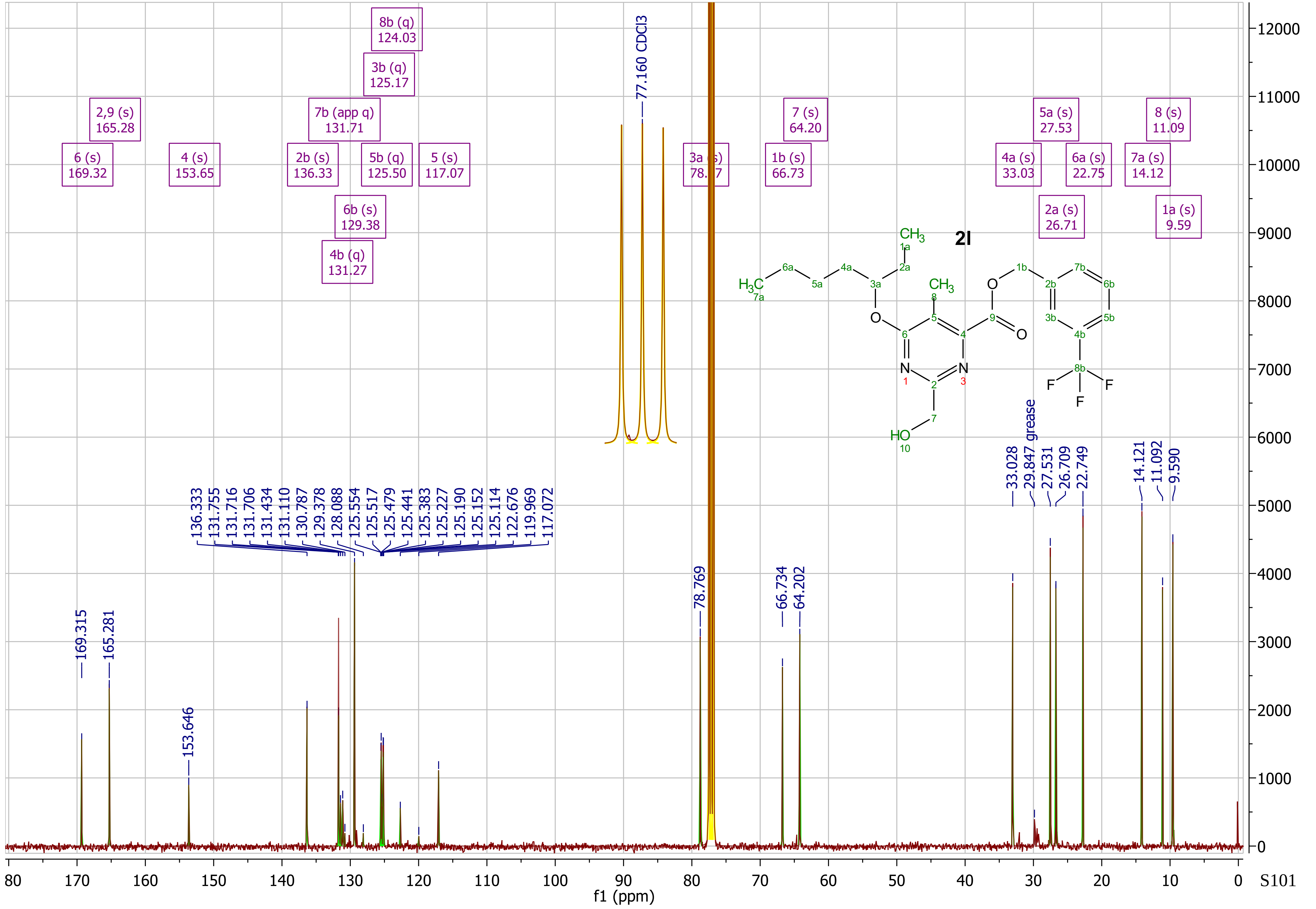

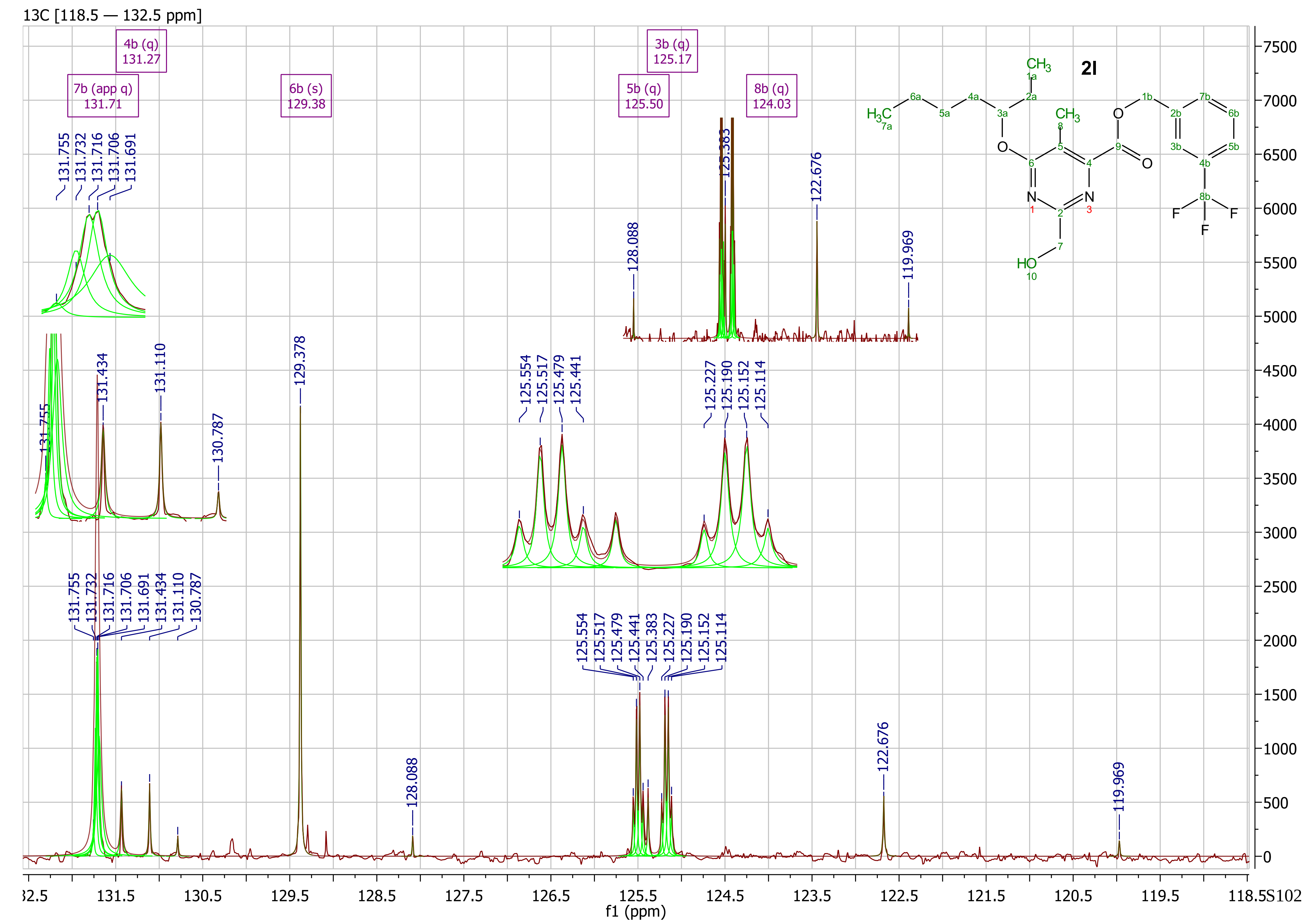
13C [118.5 — 132.5 ppm]
4b (q) 131.27
7b (app q) 131.71
6b (s) 129.38
3b (q) 125.17
5b (q) 125.50
8b (q) 124.03
2l
131.755
131.732
131.716
131.706
131.691
131.434
131.110
130.787
129.378
128.088
125.554
125.517
125.479
125.441
125.383
125.227
125.190
125.152
125.114
122.676
119.969
f1 (ppm)

13C HSQC

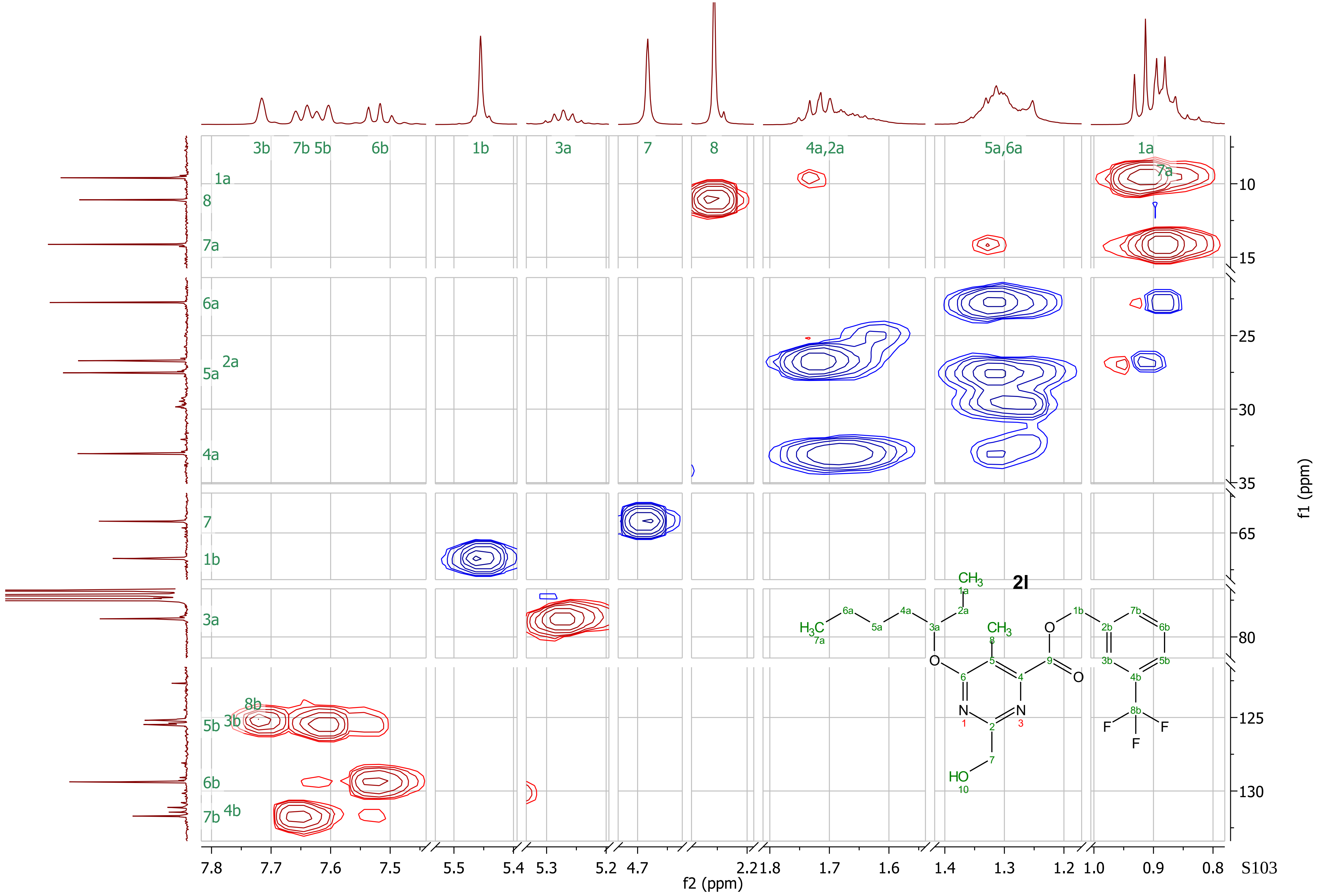

3b
7b 5b
6b
1b
3a
7
8
4a,2a
5a,6a
1a
7a
6a
2a
5a
4a
1b
8b
5b 3b
6b
7b 4b
2l
f2 (ppm)
f1 (ppm)

13C HMBC

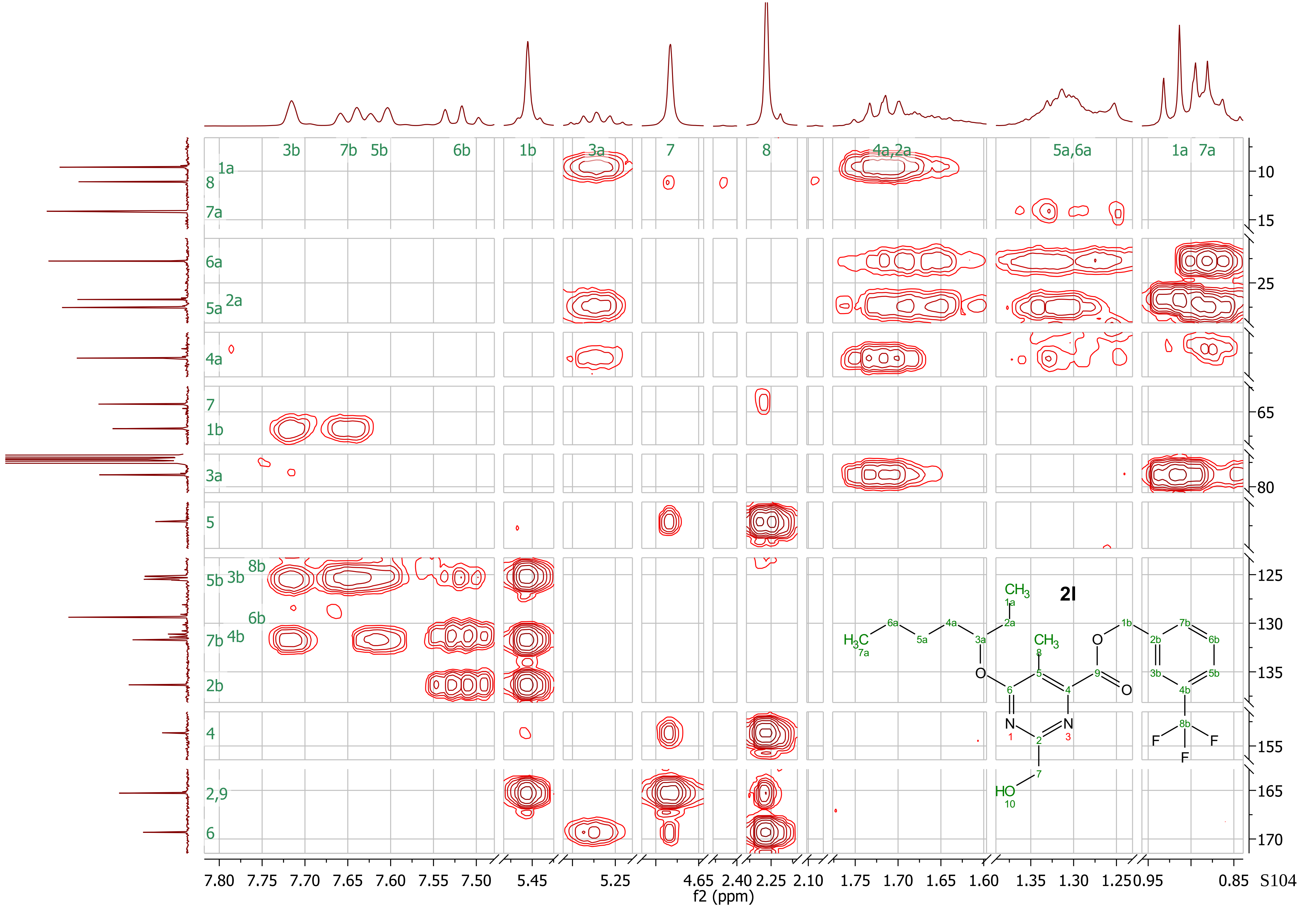

S104

1H ^{1}H NMR (400 MHz, $CDCl_3$) δ 8.51 (app t, *J* = 0.6 Hz, 1H), 7.02 – 6.93 (m, 2H), 6.85 – 6.76 (m, 2H), 5.42 (s, 2H), 4.52 (q, *J* = 7.1 Hz, 4H), 3.75 (s, 3H), 1.45 (t, *J* = 7.1 Hz, 6H).

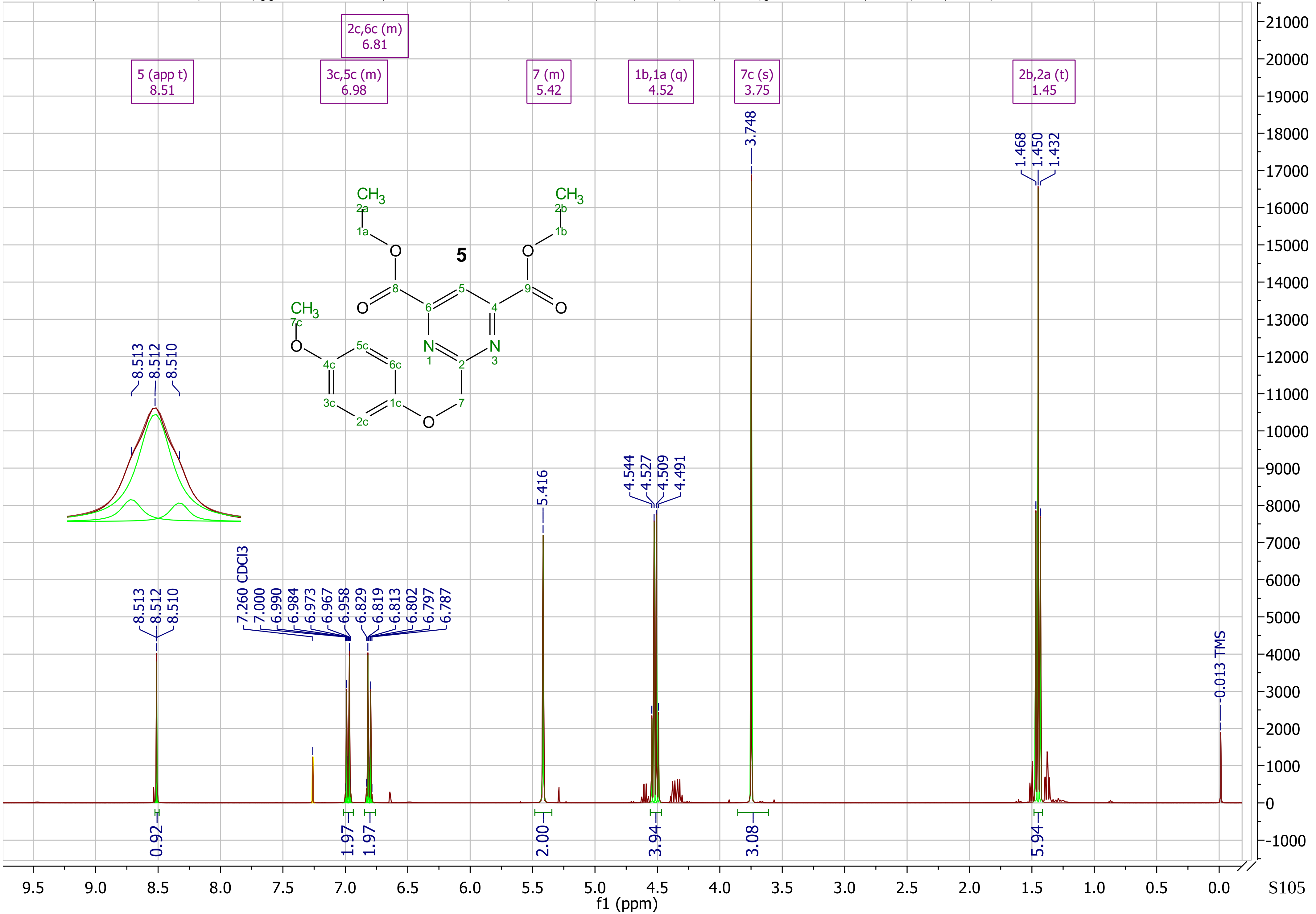

13C ^{13}C NMR (101 MHz, $CDCl_3$) δ 167.9, 163.5 (sym, 2C), 158.1 (sym, 2C), 154.4, 152.6, 119.1, 116.4 (sym, 2C), 114.7 (sym, 2C), 71.5, 63.2 (sym, 2C), 55.8, 14.3 (sym, 2C).

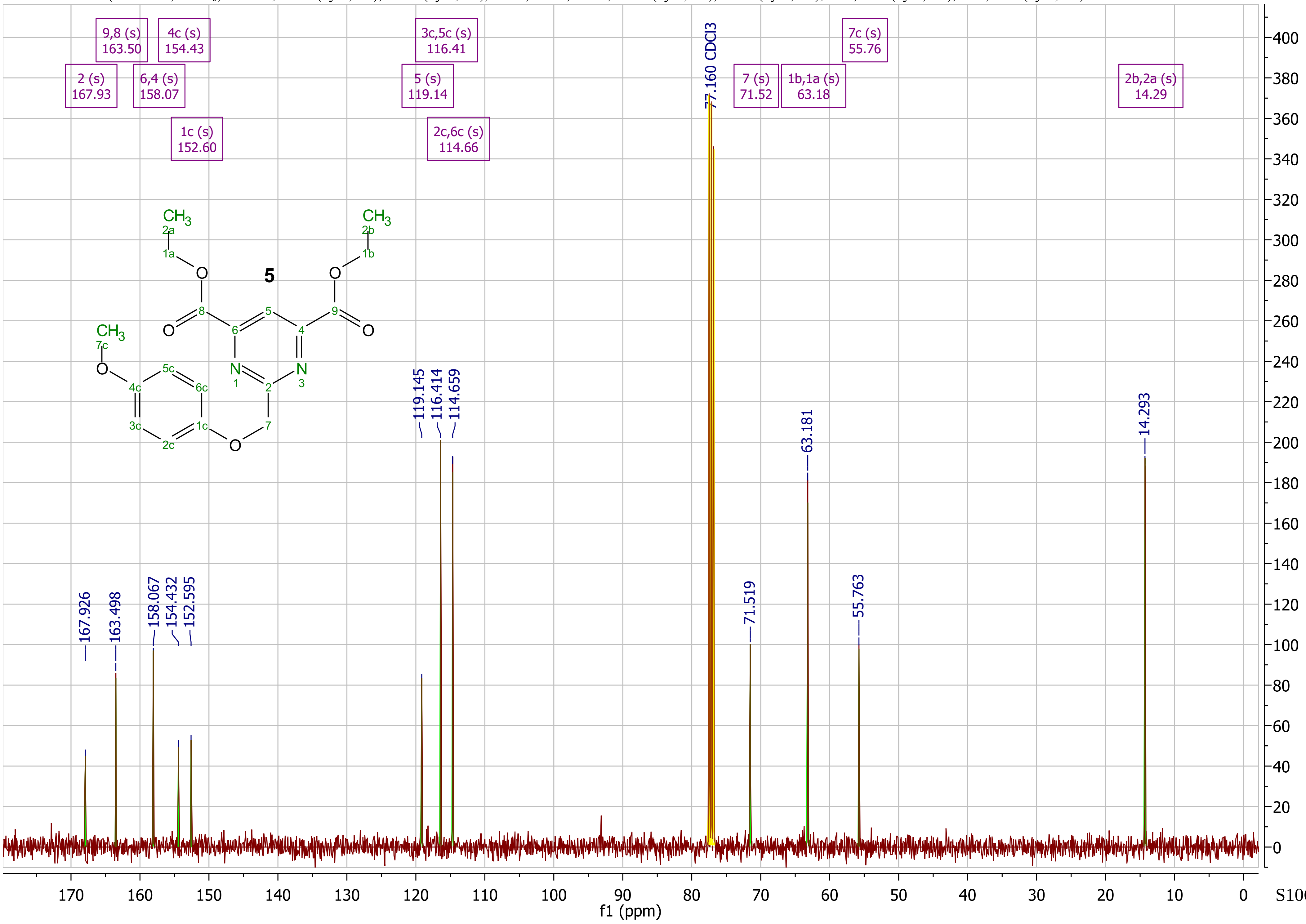

13C HSQC

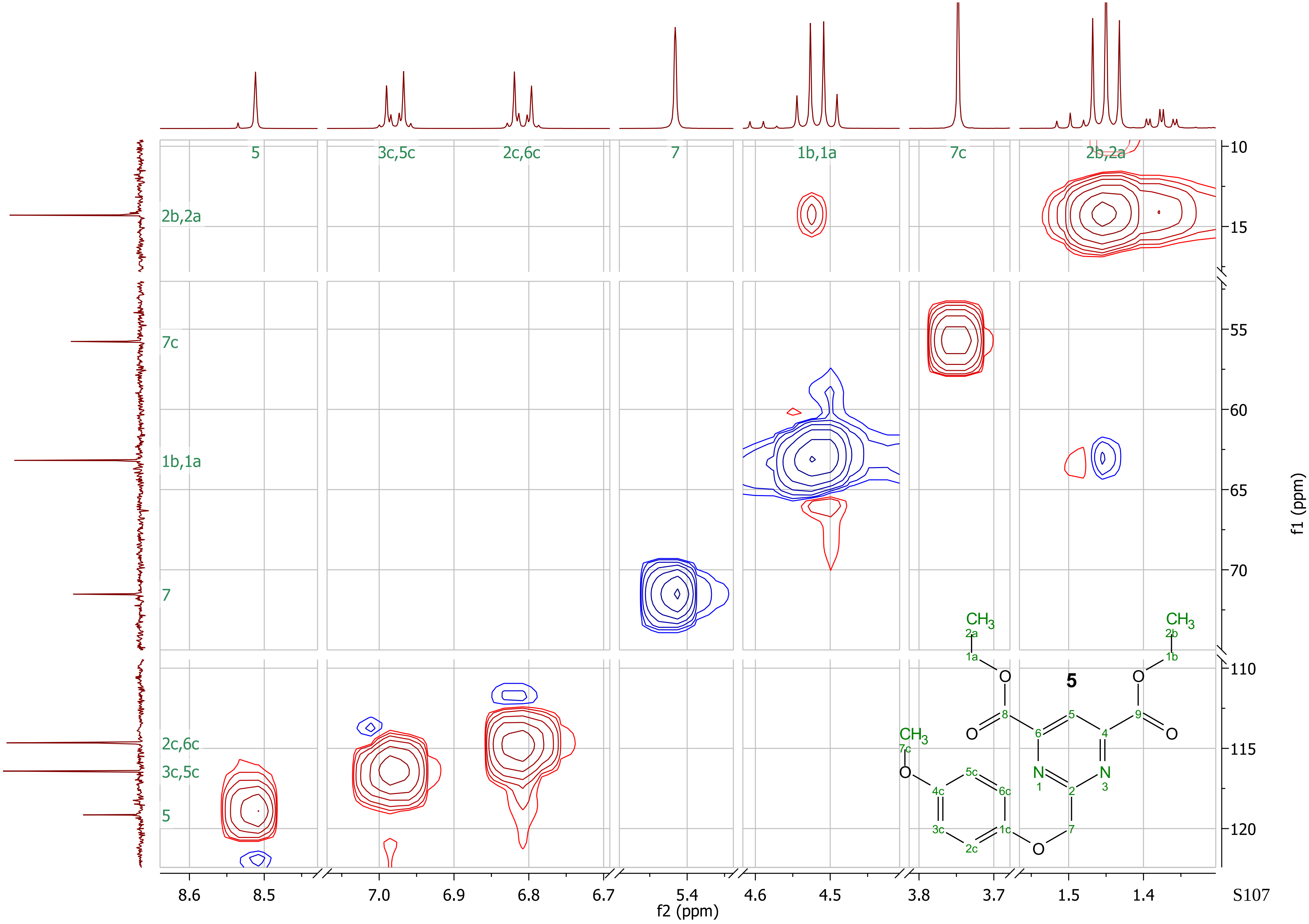

5
3c,5c
2c,6c
7
1b,1a
7c
2b,2a
2b,2a
7c
1b,1a
7
2c,6c
3c,5c
5
f1 (ppm)
f2 (ppm)
10
15
55
60
65
70
110
115
120
8.6
8.5
7.0
6.9
6.8
6.7
5.4
4.6
4.5
3.8
3.7
1.5
1.4

13C HMBC

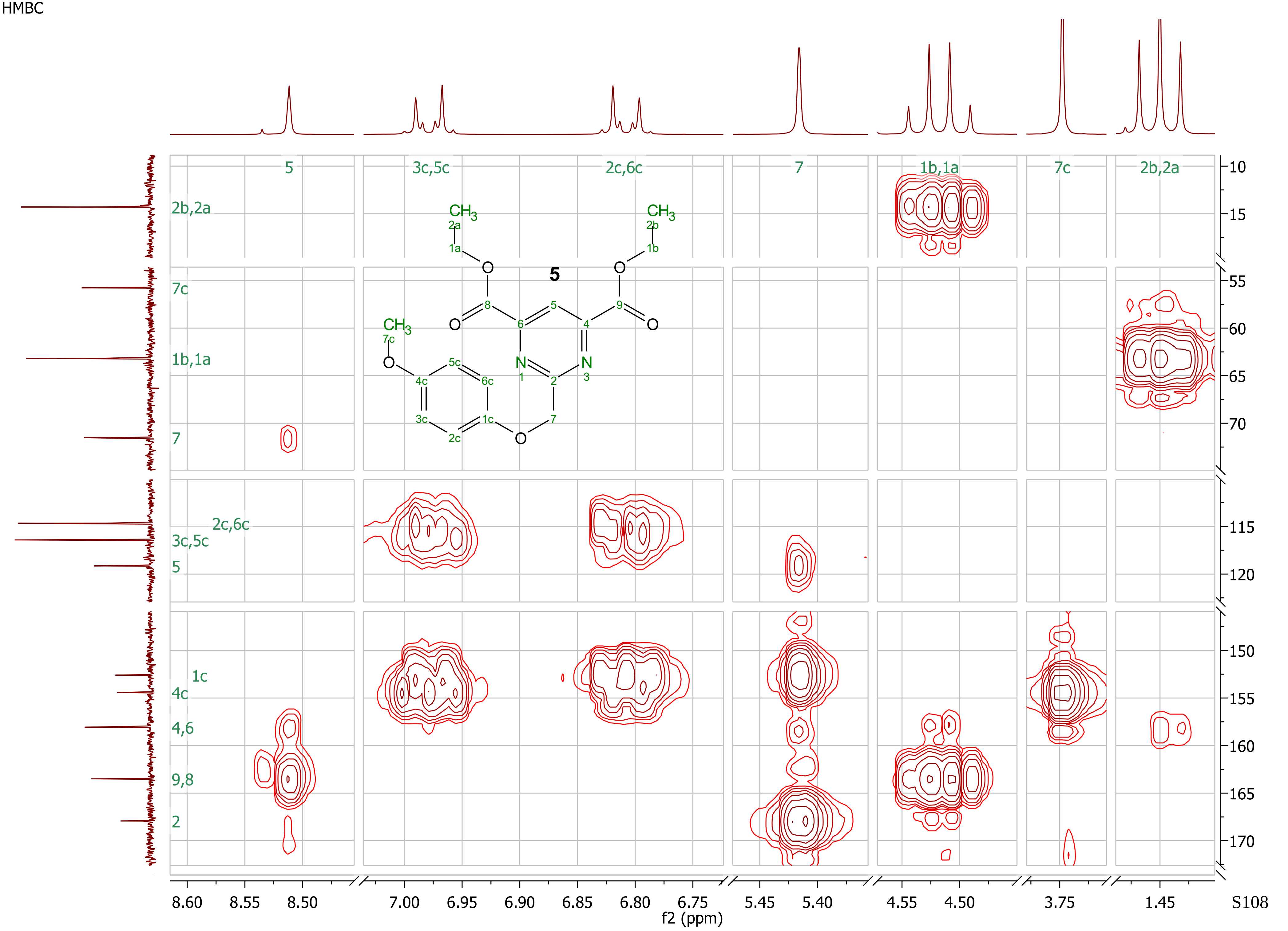
5
CH3
2a
1a
O
8
O
6
5
4
9
O
O
CH3
2b
1b
CH3
7c
O
4c
5c
6c
3c
2c
1c
O
7
2
N
1
N
3
2b,2a
7c
1b,1a
7
3c,5c
2c,6c
5
1c
4c
4,6
9,8
2
f2 (ppm)
f1 (ppm)

15N HMBC

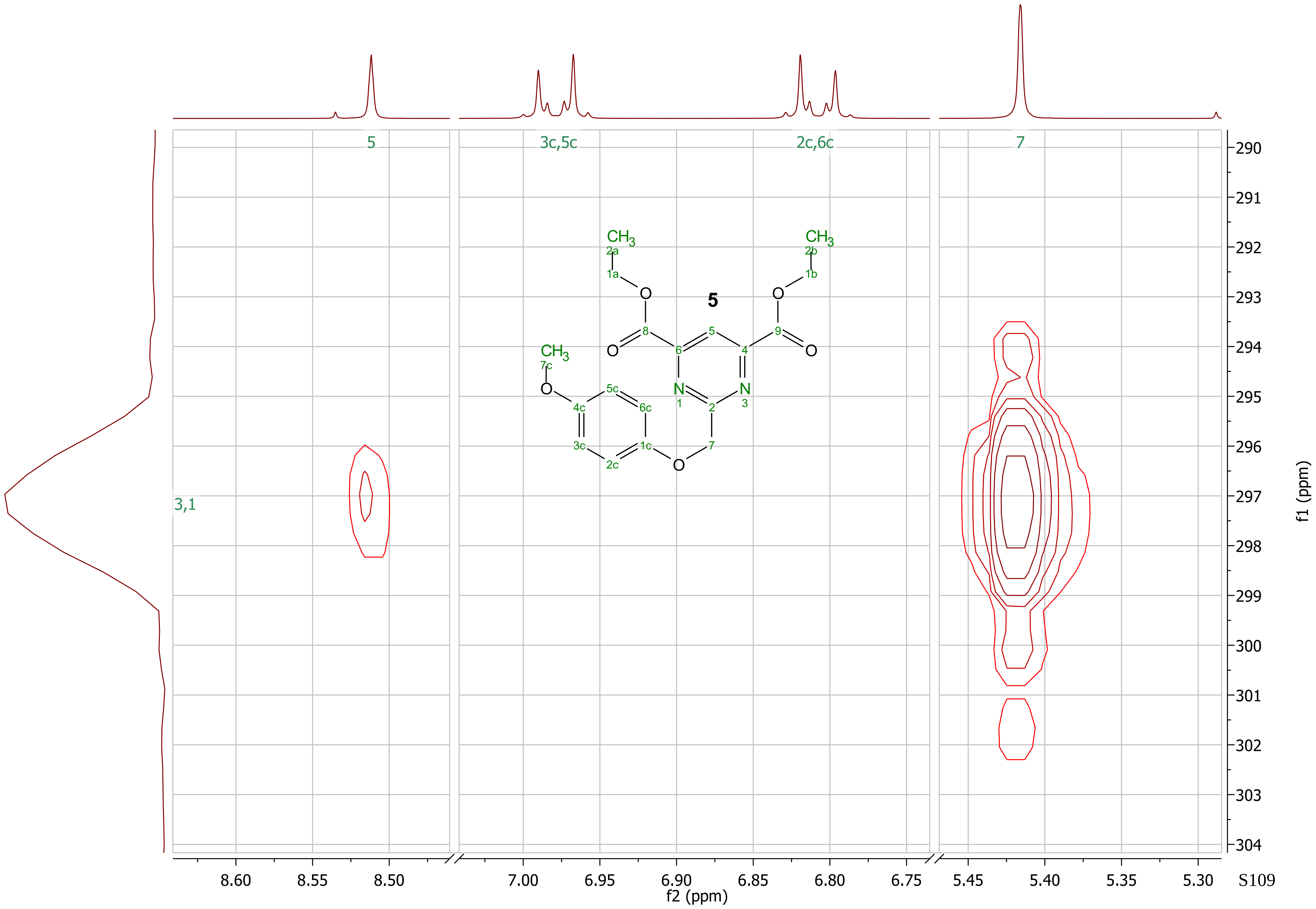

5
3c,5c
2c,6c
7
3,1
5
CH3
2a
1a
O
8
O
6
5
4
9
O
O
CH3
2b
1b
CH3
7c
O
4c
5c
6c
N
1
2
N
3
3c
1c
2c
O
7
8.60
8.55
8.50
7.00
6.95
6.90
6.85
6.80
6.75
5.45
5.40
5.35
5.30
f2 (ppm)
290
291
292
293
294
295
296
297
298
299
300
301
302
303
304
f1 (ppm)

15N HMBC f1 Projection ^{15}N NMR (41 MHz, $CDCl_3$) δ 297.15.

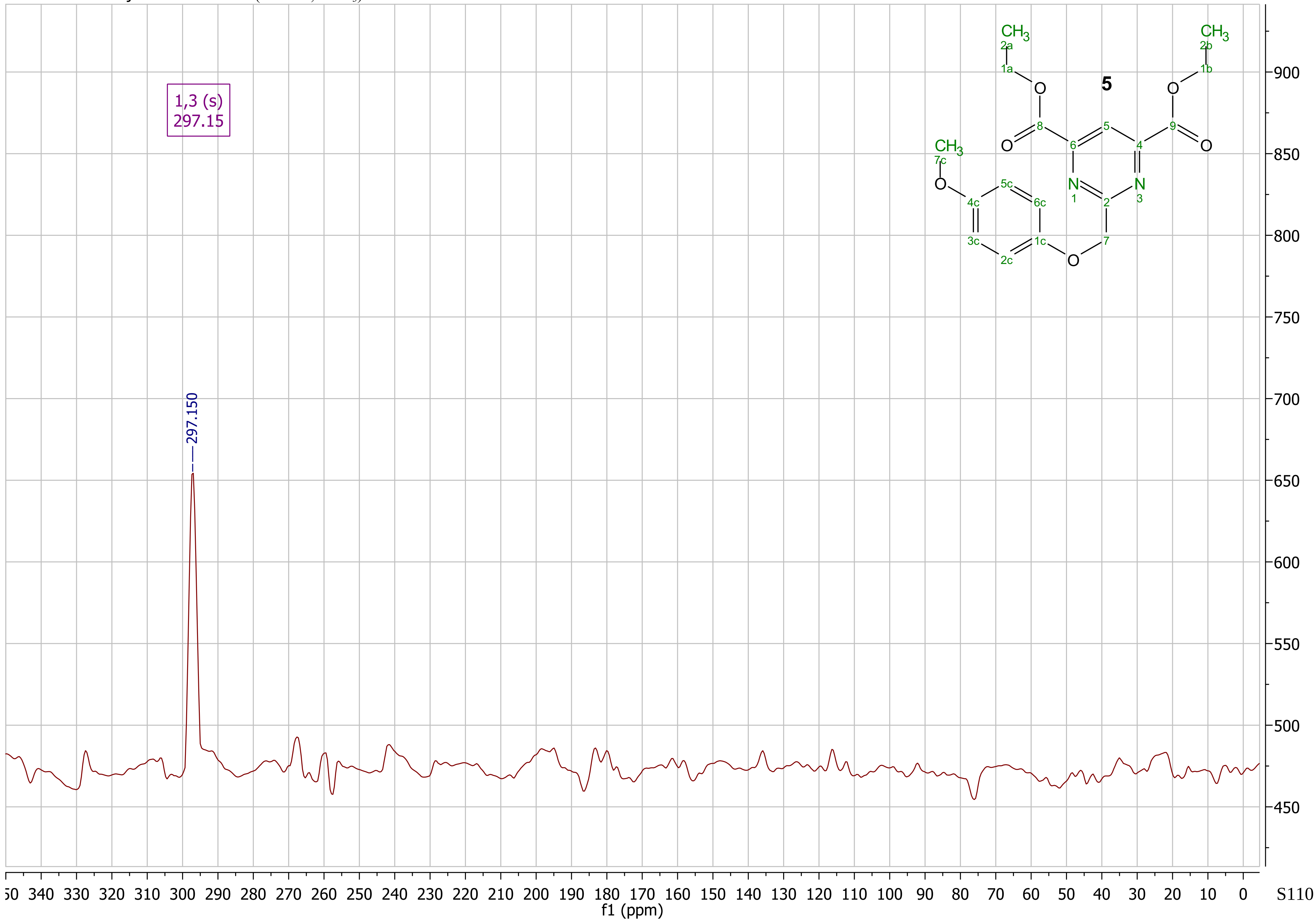

1H ^{1}H NMR (400 MHz, $CDCl_3$) δ 8.48 (app t, J = 0.6 Hz, 1H), 7.02 – 6.95 (m, 2H), 6.85 – 6.77 (m, 2H), 5.42 (s, 2H), 4.44 (t, J = 6.9 Hz, 4H), 3.75 (s, 3H), 1.82 (quint, J = 6.9 Hz, 4H), 1.49 – 1.21 (m, 16H), 0.89 (app t, J = 7.0 Hz, 6H).

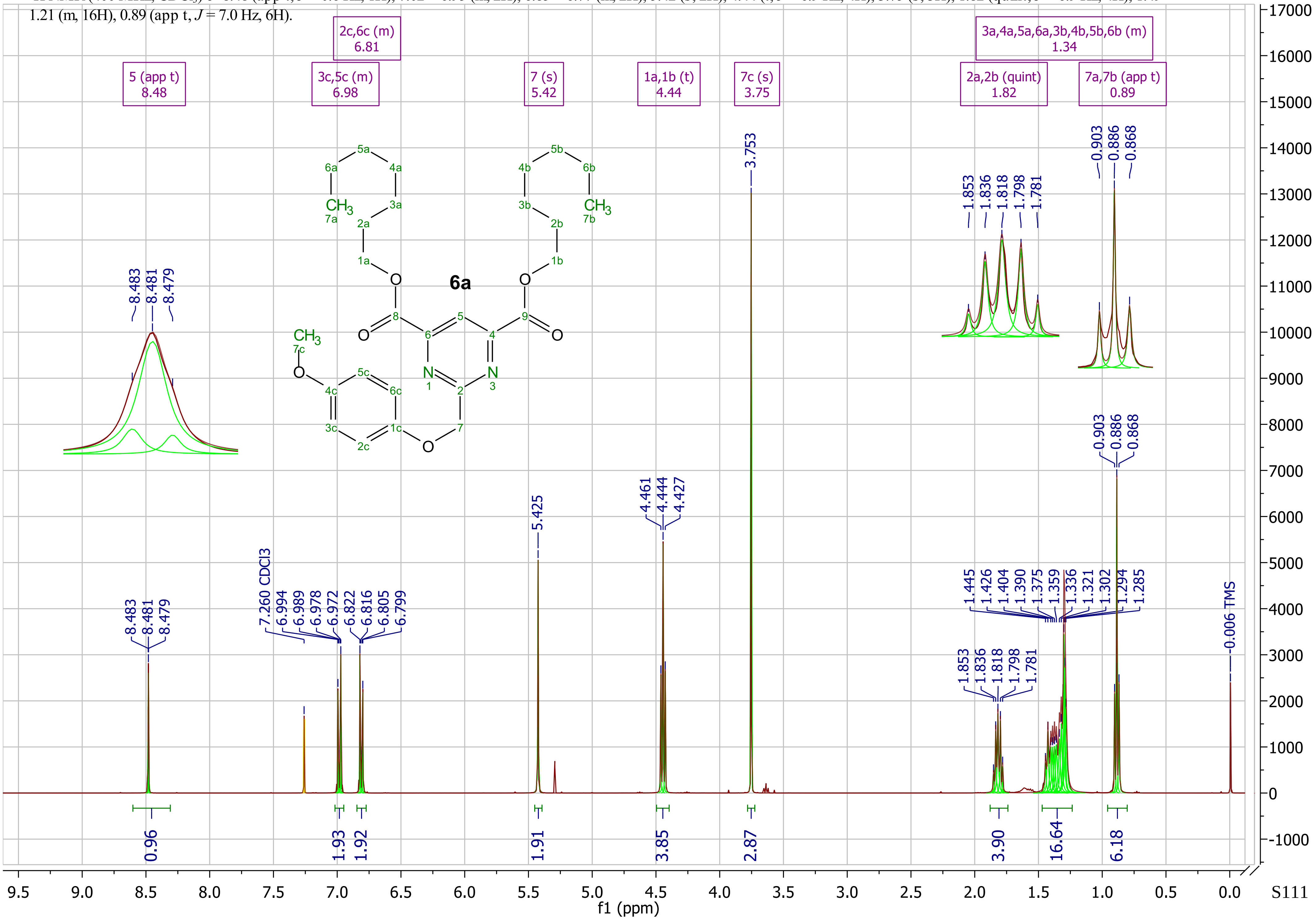

13C ^{13}C NMR (101 MHz, $CDCl_3$) δ 168.0, 163.6 (sym, 2C), 158.1 (sym, 2C), 154.4, 152.6, 119.1, 116.4 (sym, 2C), 114.7 (sym, 2C), 71.5, 67.3 (sym, 2C), 55.8, 31.8 (sym, 2C), 29.0 (sym, 2C), 28.6 (sym, 2C), 25.9 (sym, 2C), 22.7 (sym, 2C), 14.2 (sym, 2C).

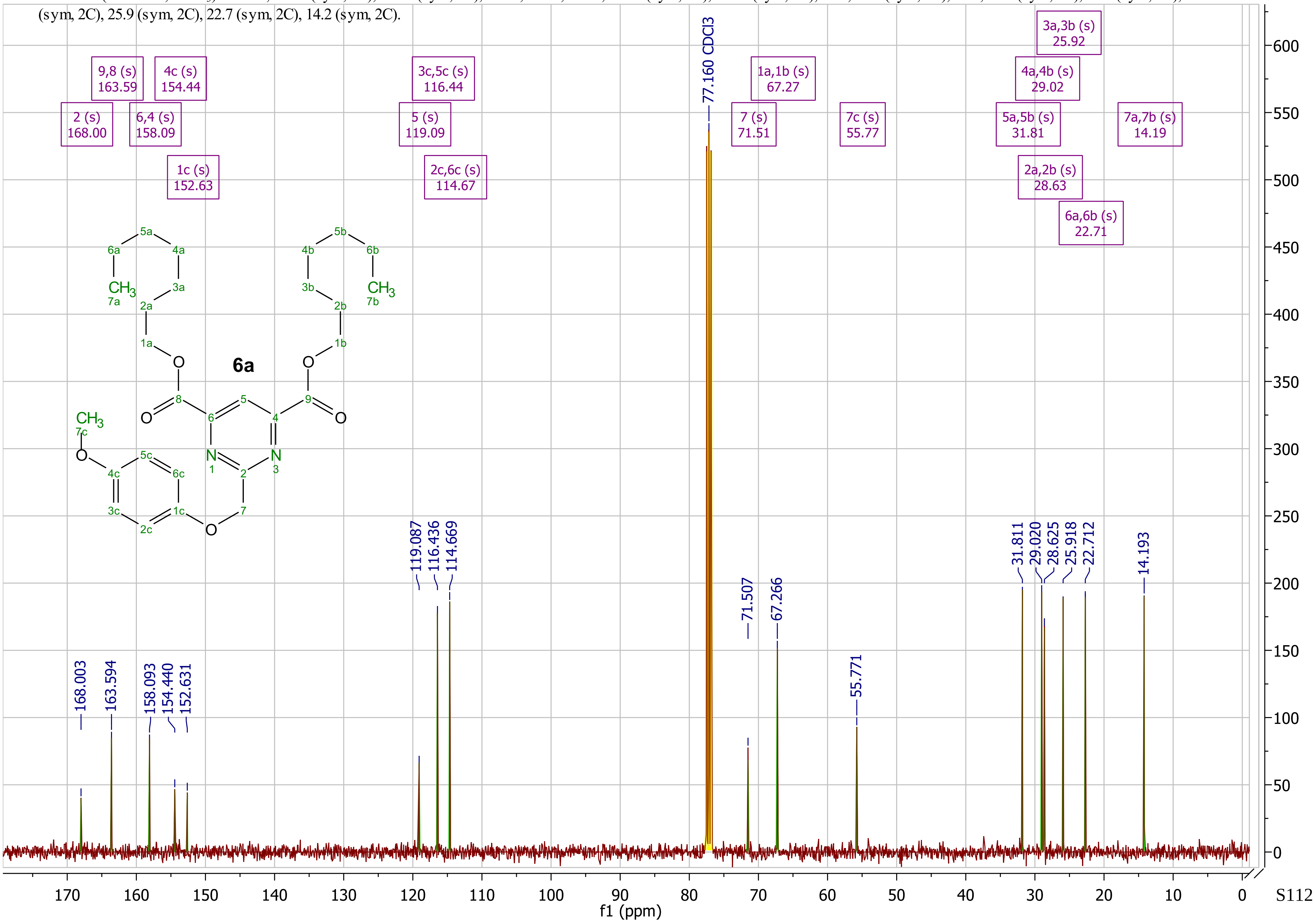

13C HSQC

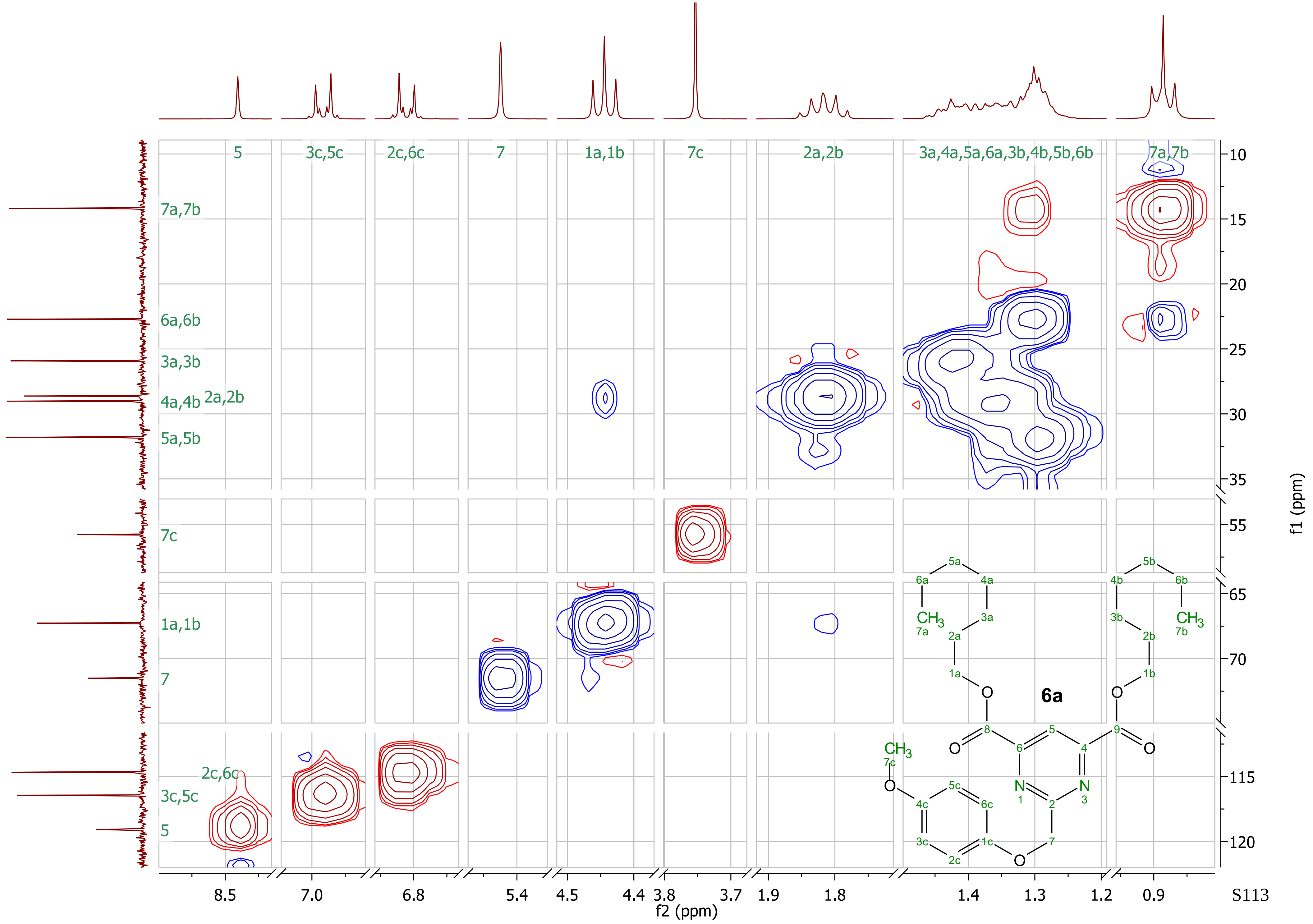

5
3c,5c
2c,6c
7
1a,1b
7c
2a,2b
3a,4a,5a,6a,3b,4b,5b,6b
7a,7b
6a,6b
3a,3b
4a,4b
5a,5b
f1 (ppm)
f2 (ppm)
6a

13C HMBC

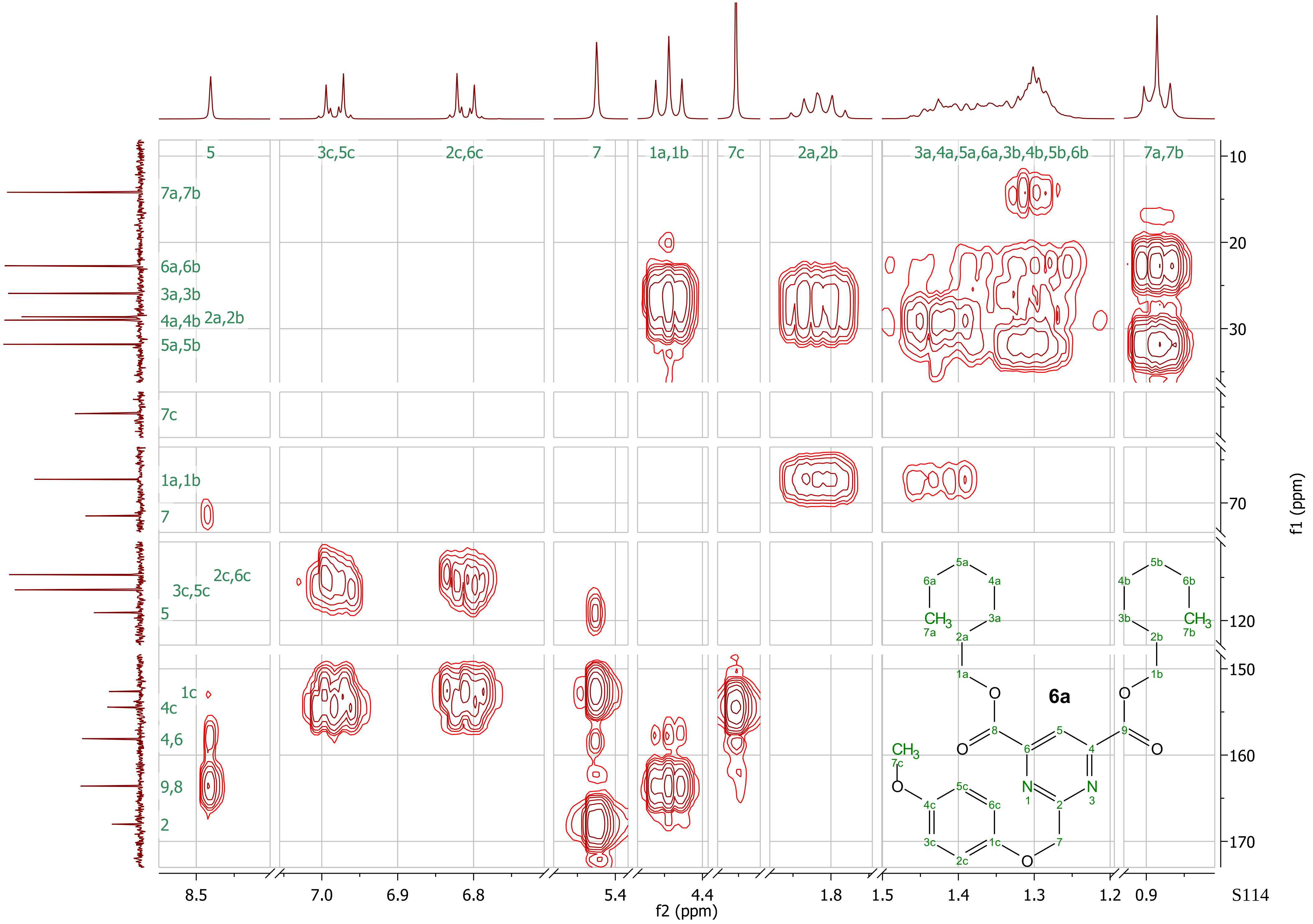

5
3c,5c
2c,6c
7
1a,1b
7c
2a,2b
3a,4a,5a,6a,3b,4b,5b,6b
7a,7b
6a,6b
3a,3b
4a,4b 2a,2b
5a,5b
7c
1a,1b
7
3c,5c
2c,6c
5
1c
4c
4,6
9,8
2
6a
f2 (ppm)
f1 (ppm)

15N HMBC

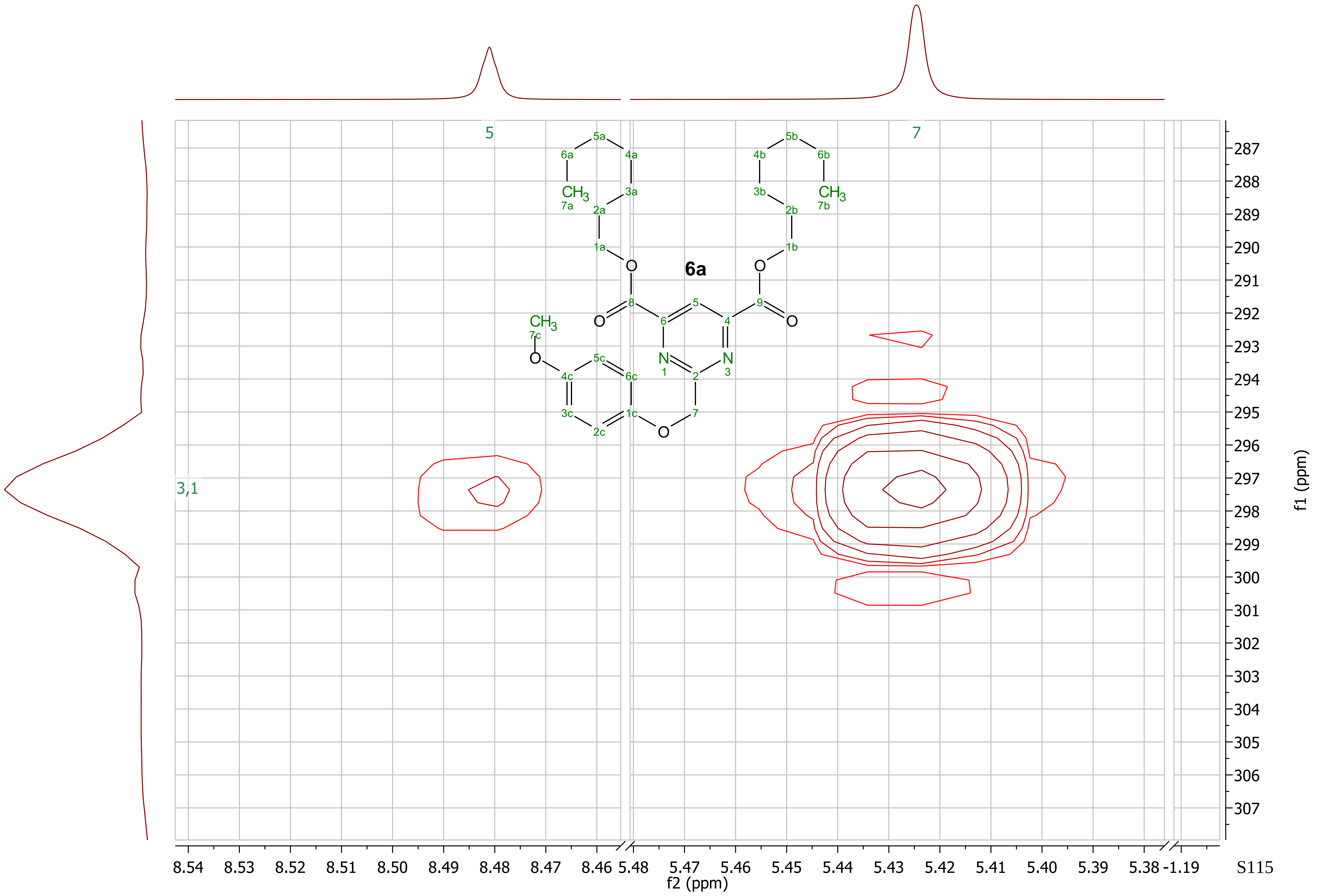

15N HMBC f1 Projection ^{15}N NMR (41 MHz, $CDCl_3$) δ 297.28.

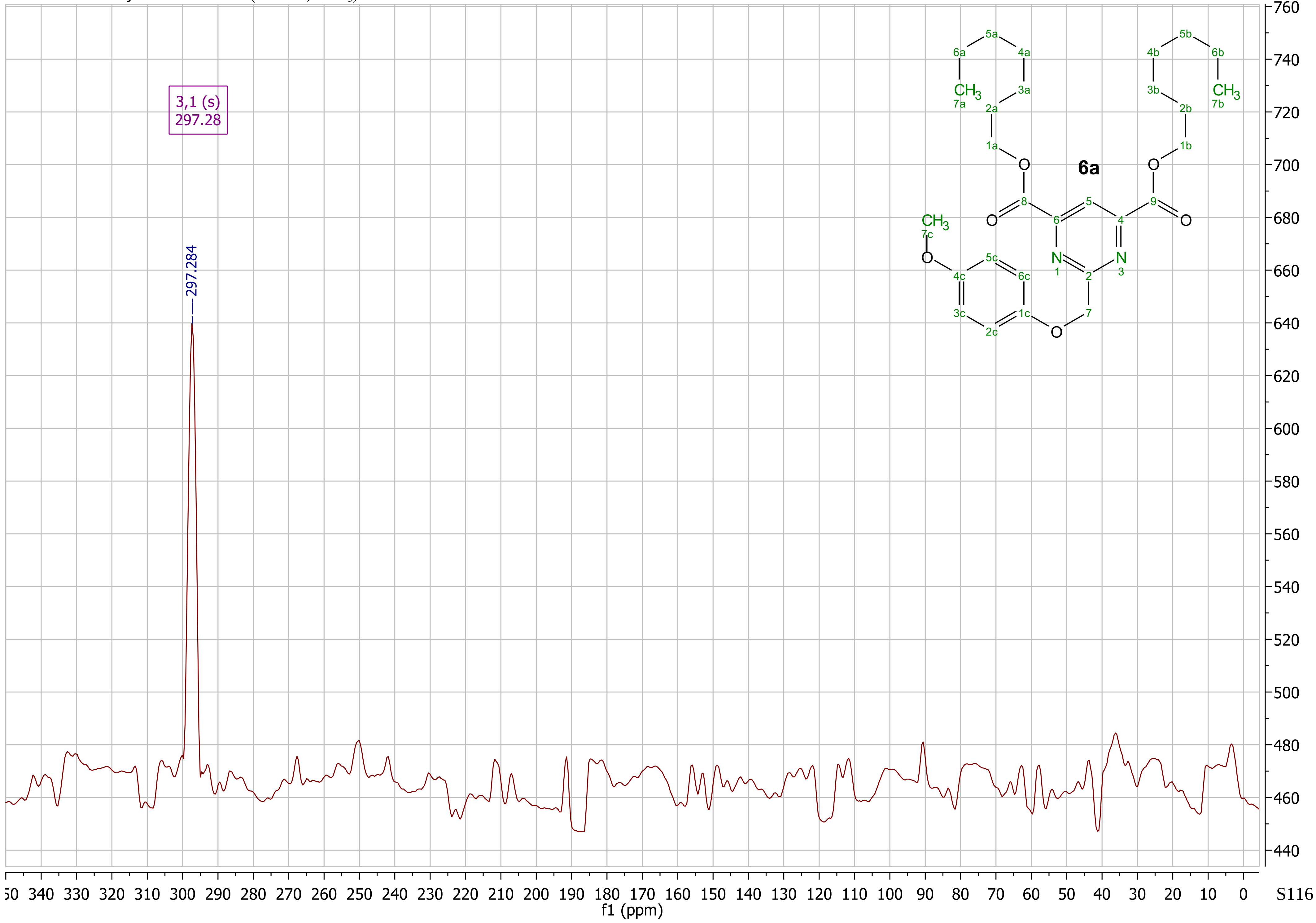

^{1}H NMR (400 MHz, $CDCl_3$) δ 8.48 (app t, J = 0.6 Hz, 1H), 7.04 – 6.89 (m, 2H), 6.88 – 6.73 (m, 2H), 5.42 (s, 2H), 4.44 (t, J = 6.9 Hz, 4H), 3.75 (s, 3H), 1.82 (quint, J = 6.9 Hz, 4H), 1.49 – 1.14 (m, 20H), 0.88 (app t, J = 7.0 Hz, 6H).

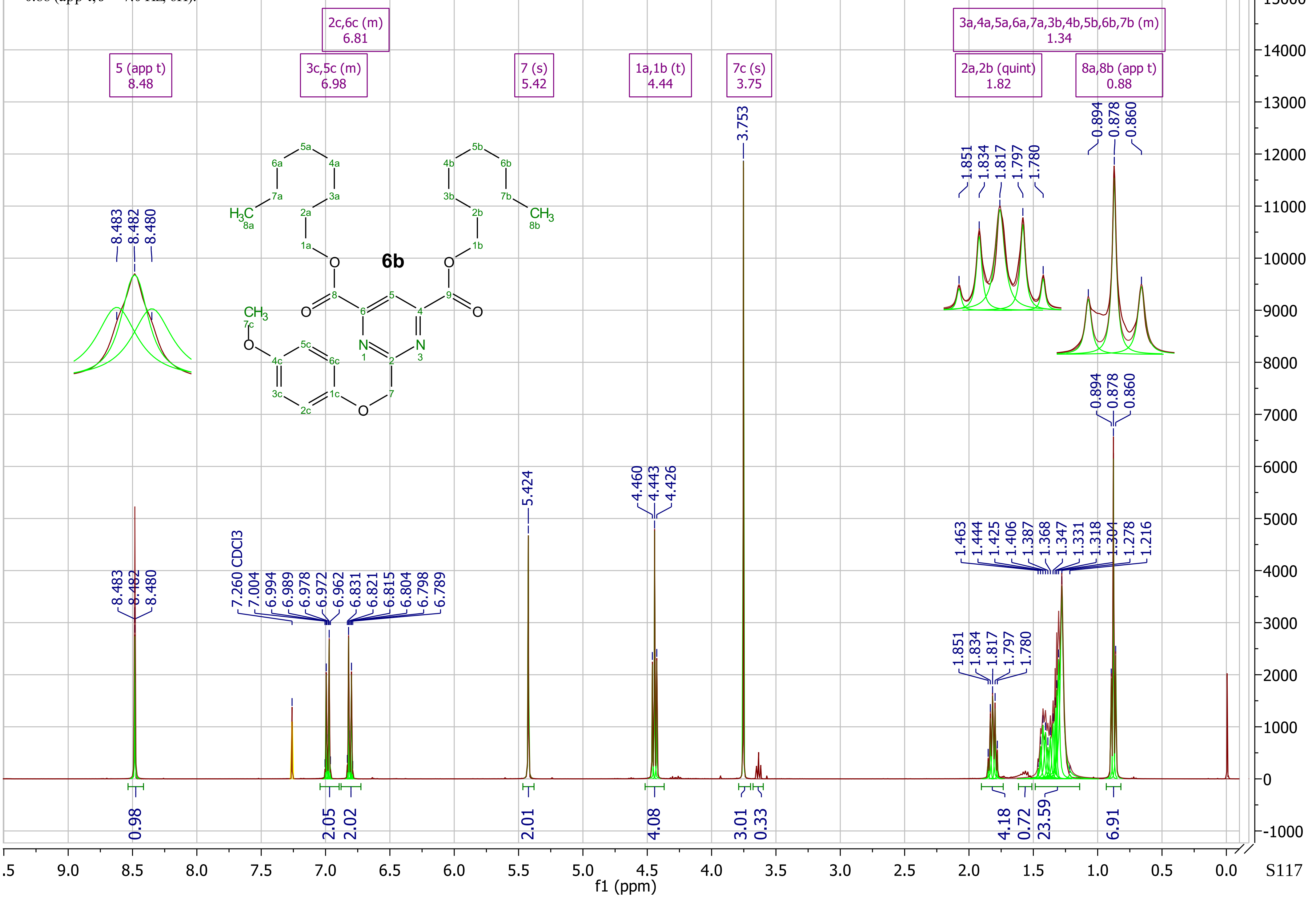

13C ^{13}C NMR (101 MHz, $CDCl_3$) δ 168.0, 163.6 (sym, 2C), 158.1 (sym, 2C), 154.4, 152.6, 119.1, 116.4 (sym, 2C), 114.7 (sym, 2C), 71.5, 67.3 (sym, 2C), 55.8, 31.9 (sym, 2C), 29.32 (sym, 2C), 29.28 (sym, 2C), 28.6 (sym, 2C), 26.0 (sym, 2C), 22.8 (sym, 2C), 14.2 (sym, 2C).

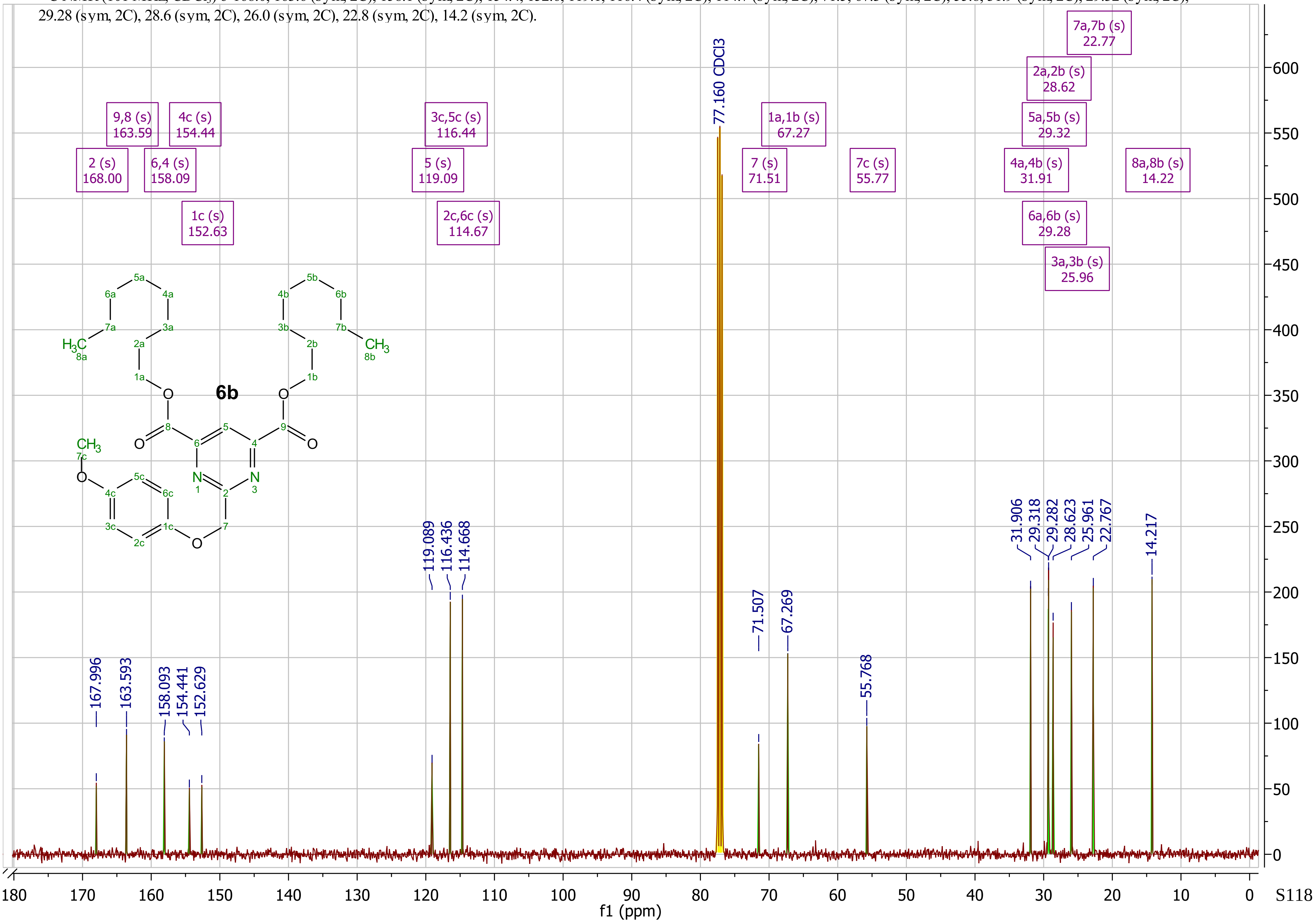

13C HSQC

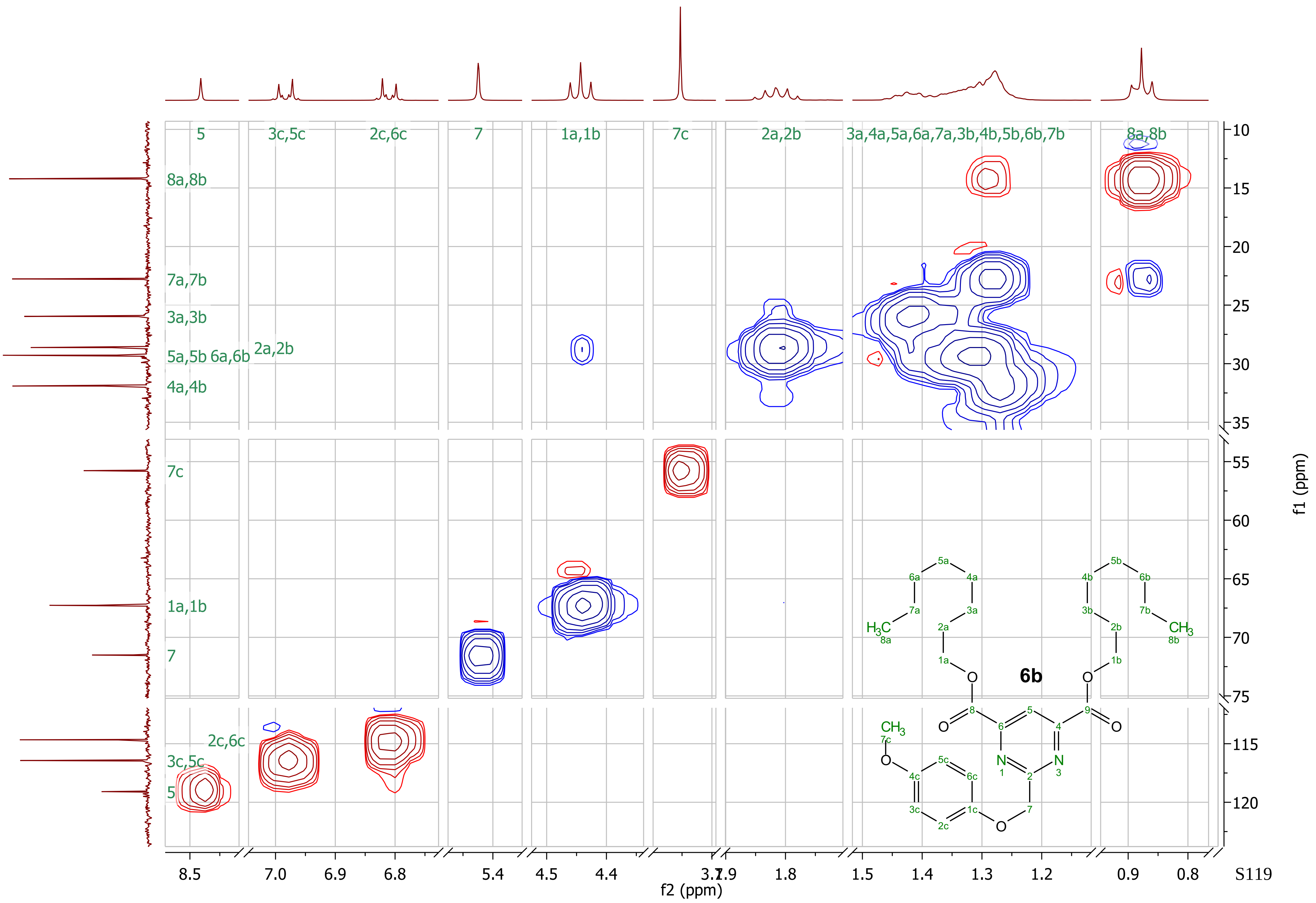

5
3c,5c
2c,6c
7
1a,1b
7c
2a,2b
3a,4a,5a,6a,7a,3b,4b,5b,6b,7b
8a,8b
7a,7b
3a,3b
5a,5b 6a,6b
4a,4b
1a,1b
2c,6c
3c,5c
6b
f1 (ppm)
f2 (ppm)

13C HMBC

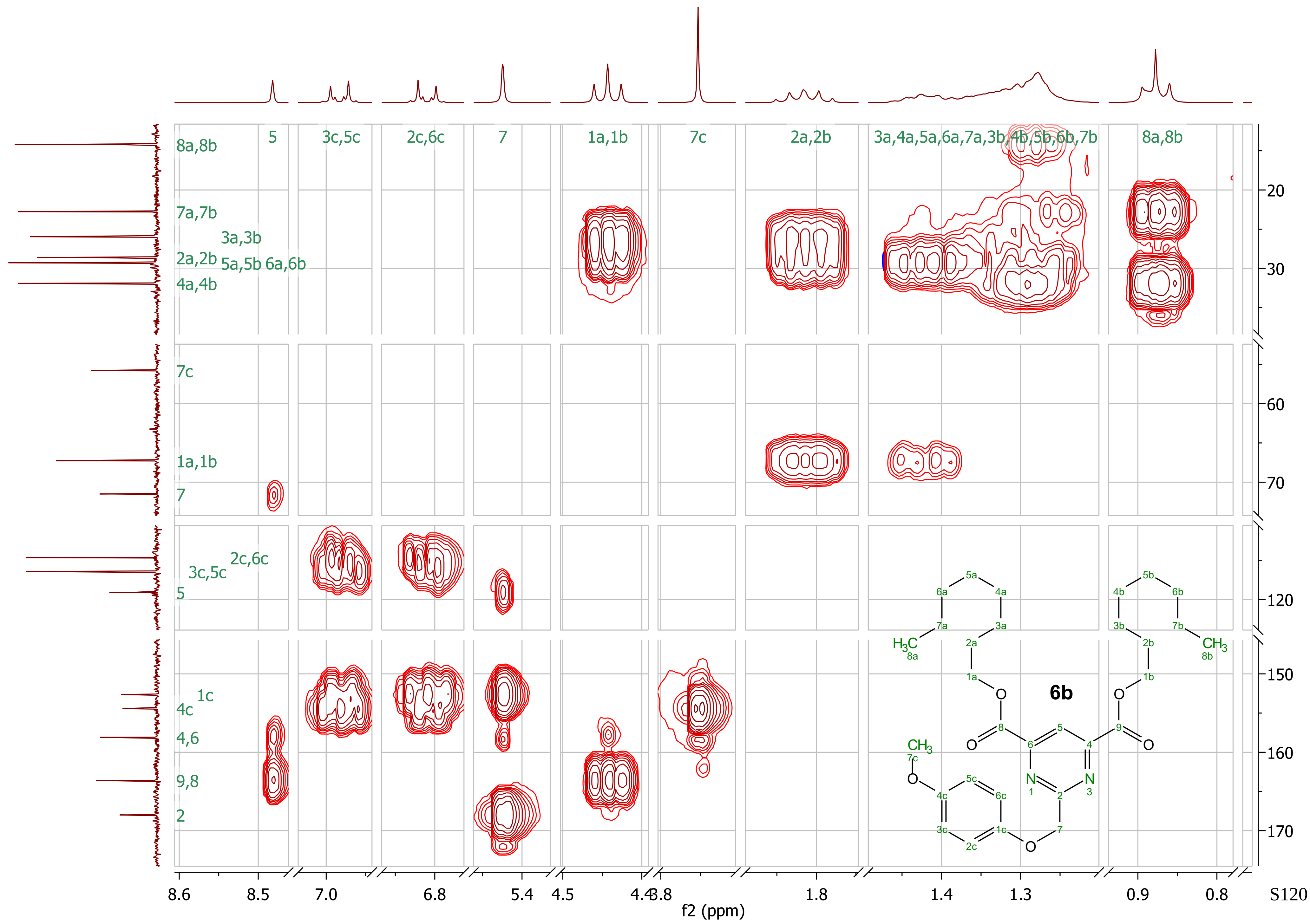

8a,8b
7a,7b
3a,3b
2a,2b
5a,5b 6a,6b
4a,4b
7c
1a,1b
7
2c,6c
3c,5c
5
1c
4c
4,6
9,8
2
5
3c,5c
2c,6c
7
1a,1b
7c
2a,2b
3a,4a,5a,6a,7a,3b,4b,5b,6b,7b
8a,8b
6b
f2 (ppm)
f1 (ppm)

15N HMBC

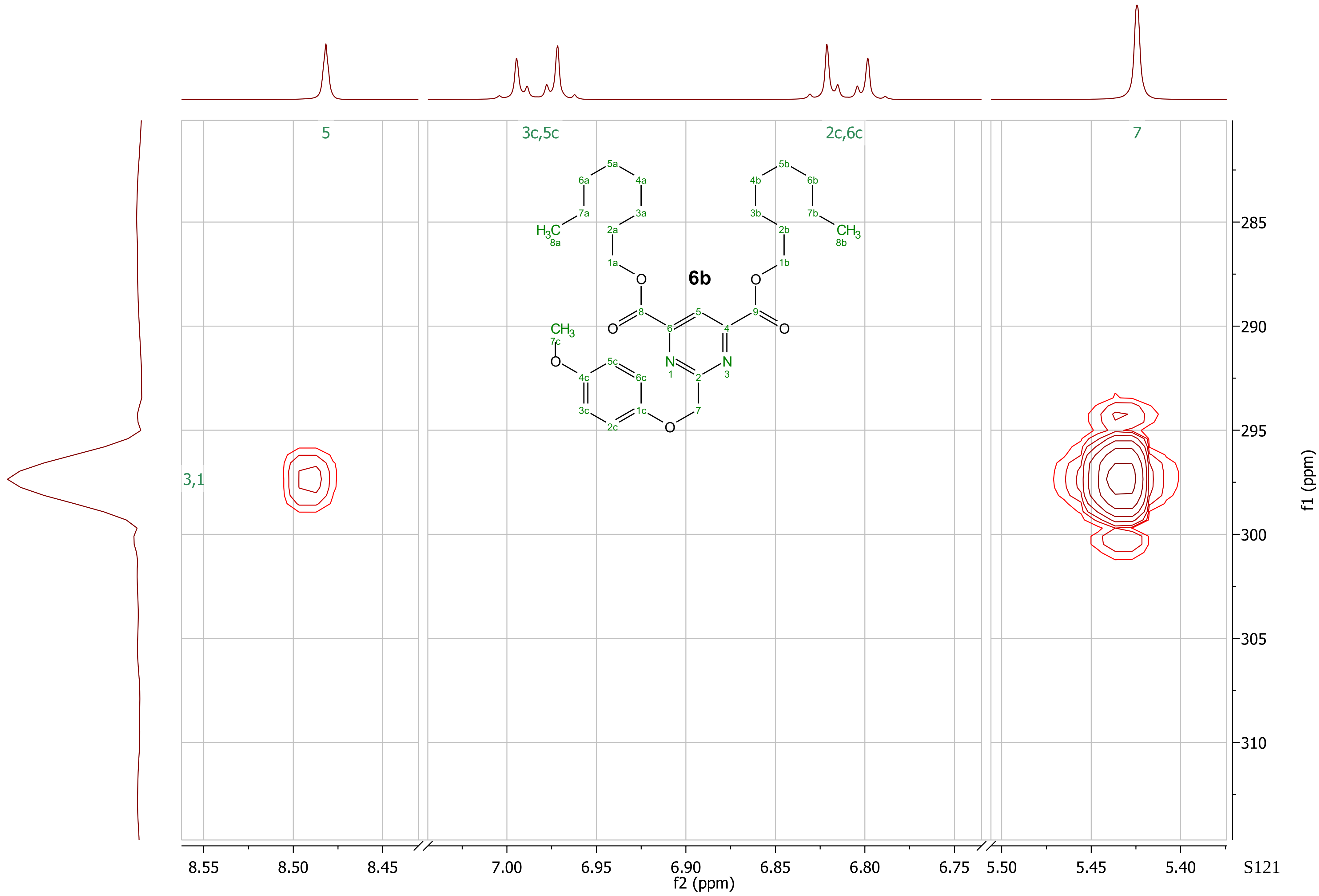

15N HMBC f1 Projection ^{15}N NMR (41 MHz, $CDCl_3$) δ 297.32.

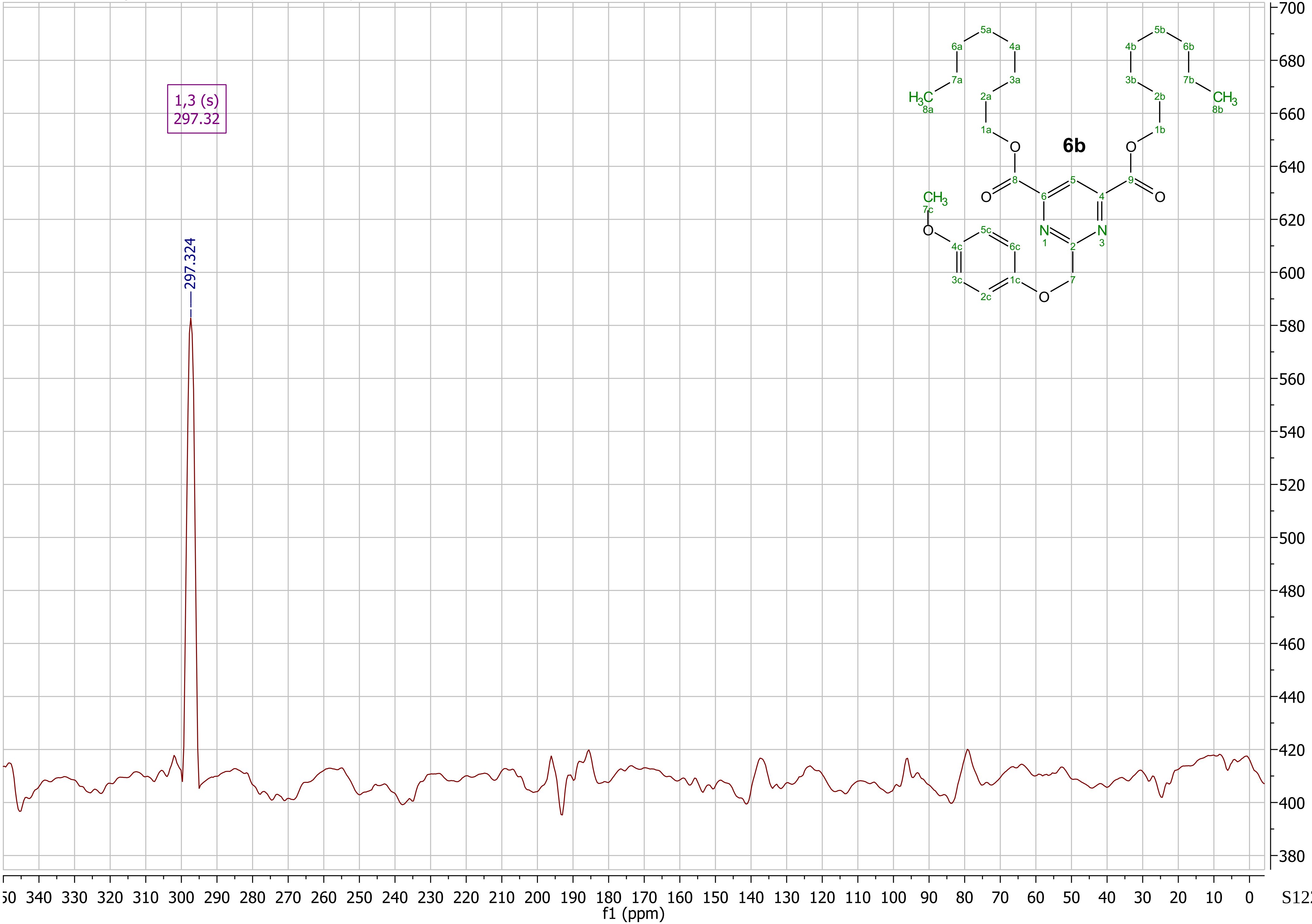

1H ^{1}H NMR (400 MHz, $CDCl_3$) δ 8.48 (s, 1H), 7.02 – 6.95 (m, 2H), 6.85 – 6.78 (m, 2H), 5.43 (s, 2H), 4.44 (t, *J* = 6.9 Hz, 4H), 3.76 (s, 3H), 1.82 (quint, *J* = 6.9 Hz, 4H), 1.48 – 1.17 (m, 32H), 0.87 (app t, *J* = 6.7 Hz, 6H).

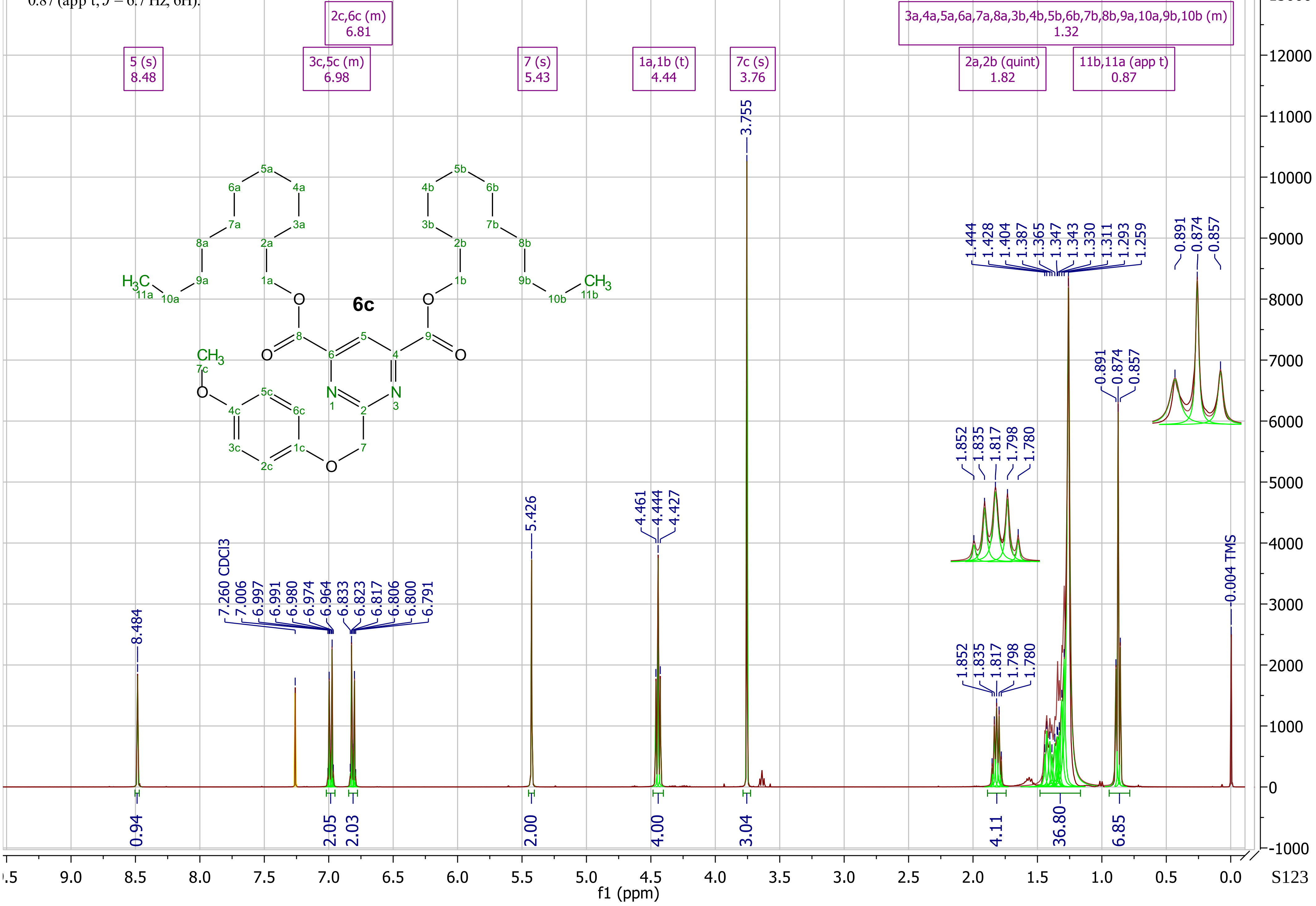

13C ^{13}C NMR (101 MHz, $CDCl_3$) δ 168.0, 163.6 (sym, 2C), 158.1 (sym, 2C), 154.4, 152.6, 119.1, 116.4 (sym, 2C), 114.7 (sym, 2C), 71.5, 67.3 (sym, 2C), 55.8, 32.0 (sym, 2C), 29.74 (sym, 2C), 29.72 (sym, 2C), 29.6 (sym, 2C), 29.5 (sym, 2C), 29.4 (sym, 2C), 28.6 (sym, 2C), 26.0 (sym, 2C), 22.8 (sym, 2C), 14.3 (sym, 2C).

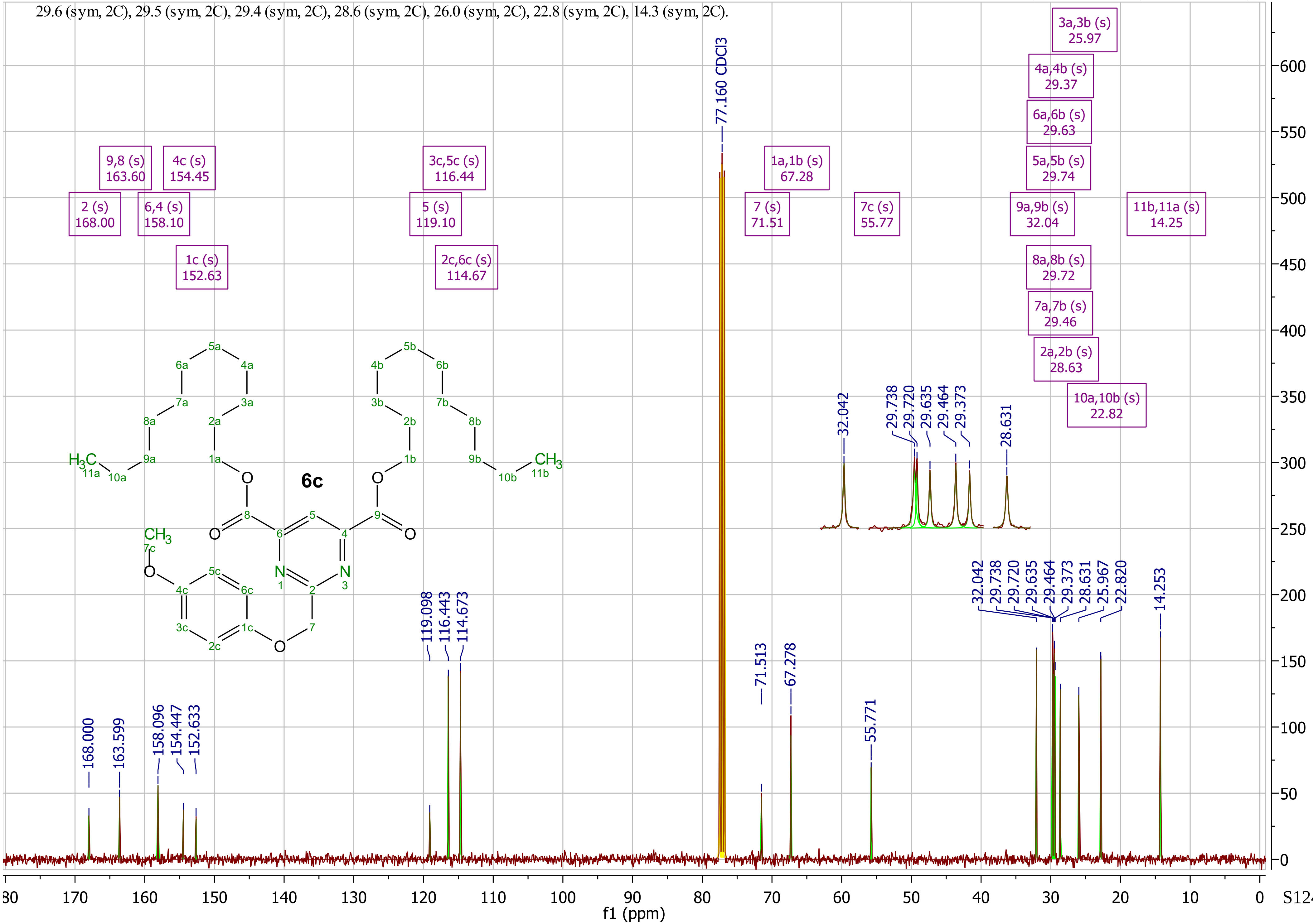

S124

13C HSQC

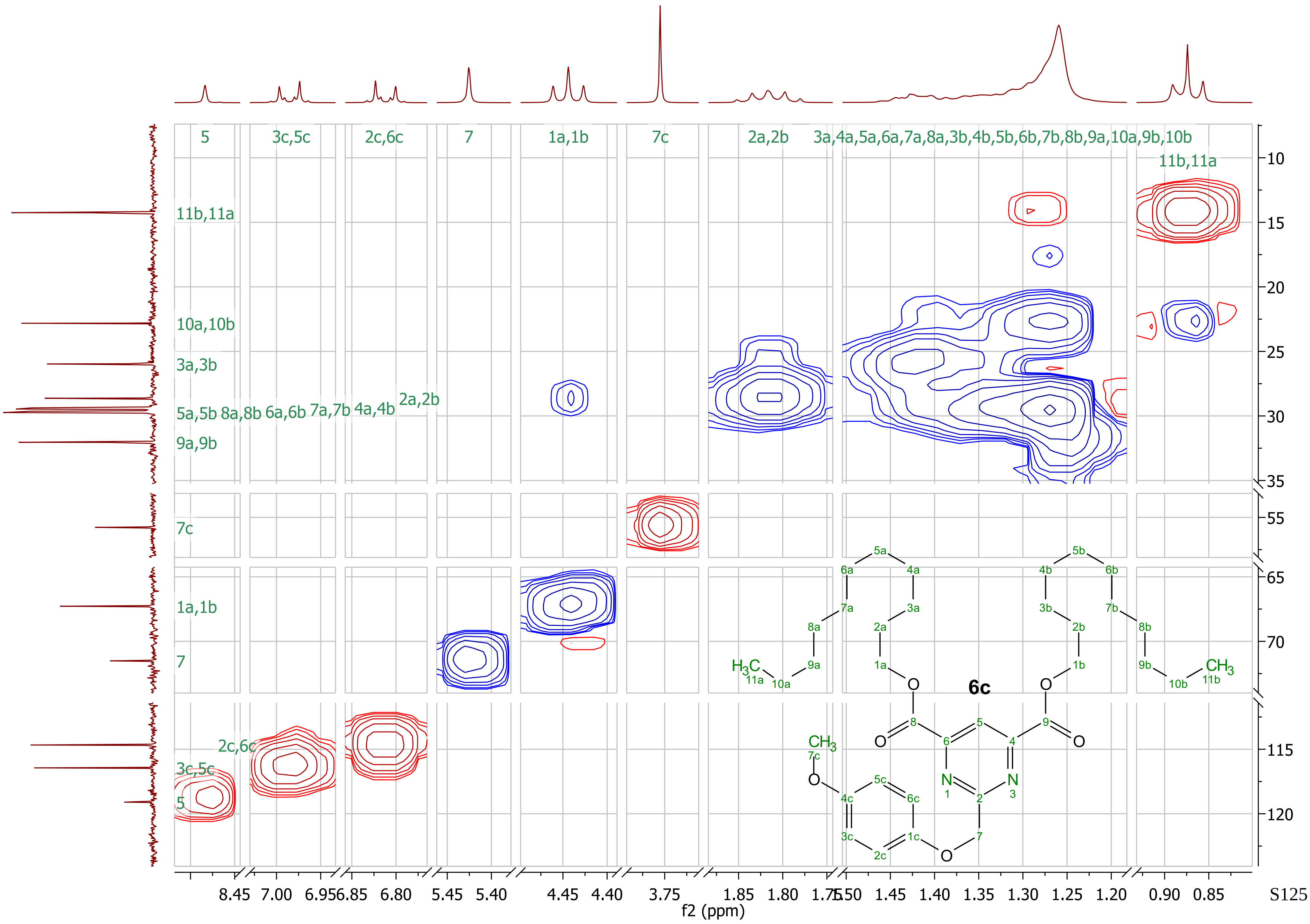

5
3c,5c
2c,6c
7
1a,1b
7c
2a,2b
3a,4a,5a,6a,7a,8a,3b,4b,5b,6b,7b,8b,9a,10a,9b,10b
11b,11a
10a,10b
3a,3b
5a,5b 8a,8b 6a,6b 7a,7b 4a,4b 2a,2b
9a,9b
7c
1a,1b
7
2c,6c
3c,5c
5
6c
f2 (ppm)
f1 (ppm)

13C HMBC

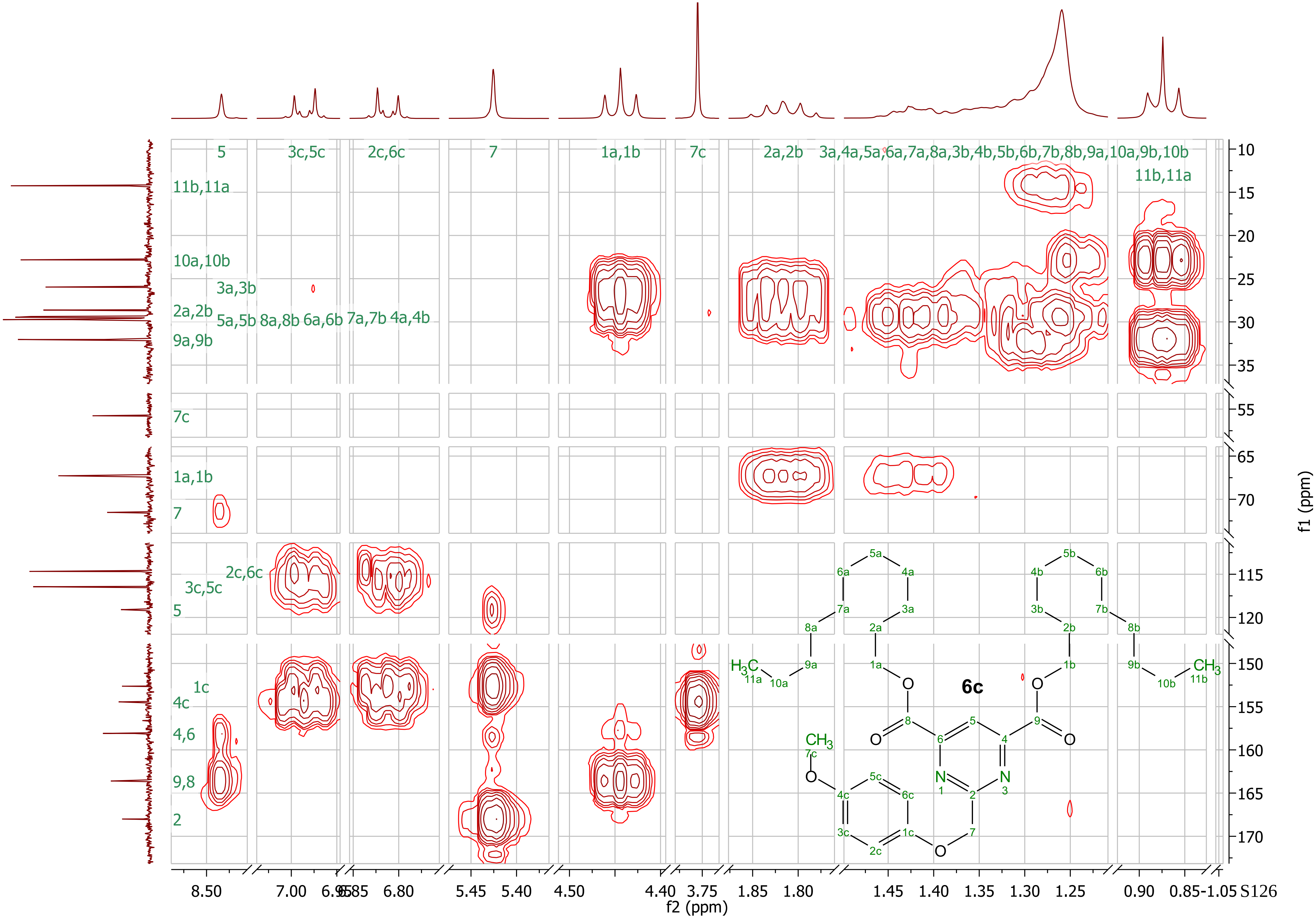

5
3c,5c
2c,6c
7
1a,1b
7c
2a,2b
3a,4a,5a,6a,7a,8a,3b,4b,5b,6b,7b,8b,9a,10a,9b,10b
11b,11a
10a,10b
3a,3b
2a,2b
5a,5b 8a,8b 6a,6b 7a,7b 4a,4b
9a,9b
7c
1a,1b
7
2c,6c
3c,5c
5
1c
4c
4,6
9,8
2
6c
f2 (ppm)
f1 (ppm)

15N HMBC

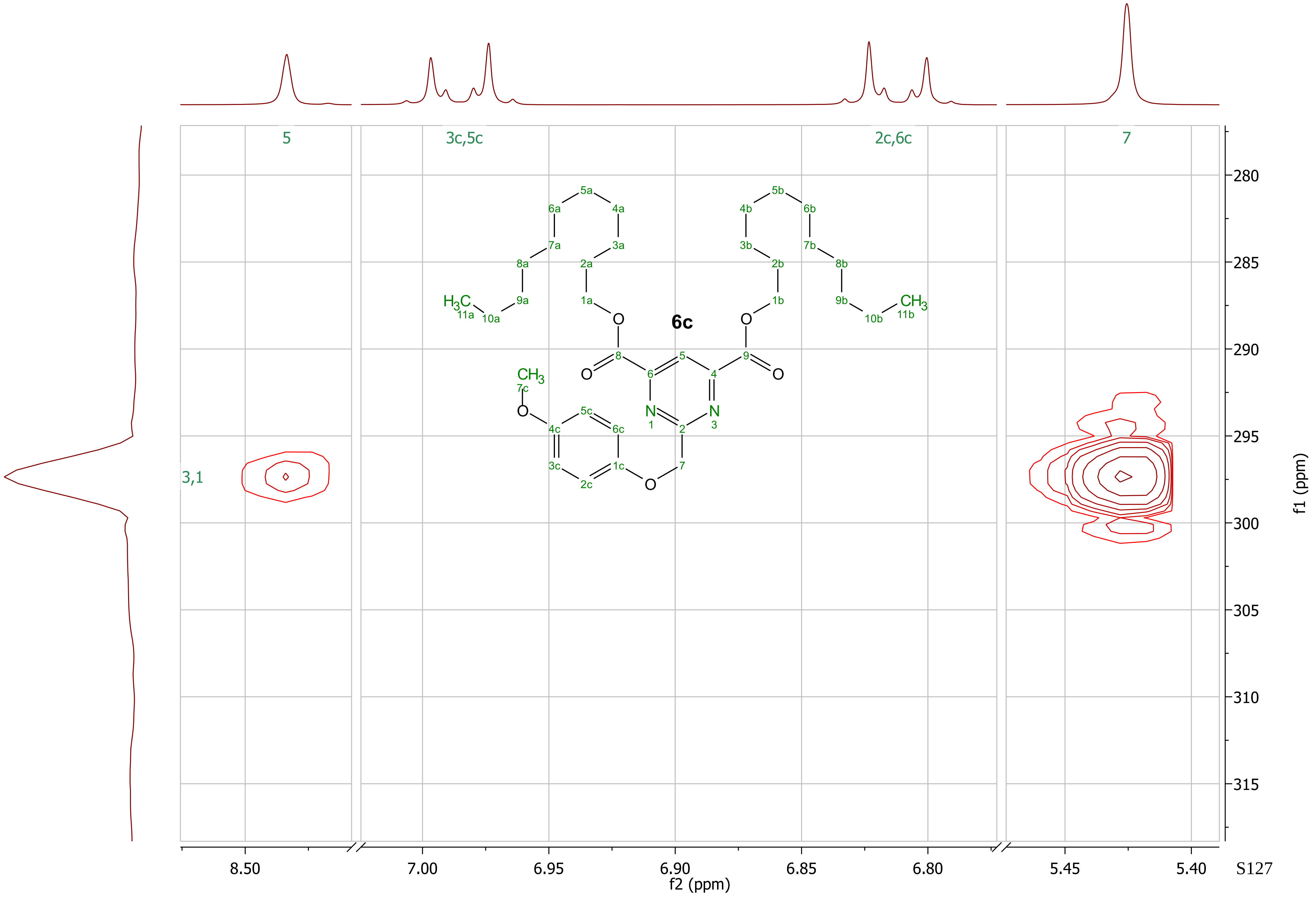

5
3c,5c
2c,6c
7
3,1
6c
f2 (ppm)
f1 (ppm)
8.50
7.00
6.95
6.90
6.85
6.80
5.45
5.40
280
285
290
295
300
305
310
315

15N HMBC f1 Projection ^{15}N NMR (41 MHz, $CDCl_3$) δ 297.32.

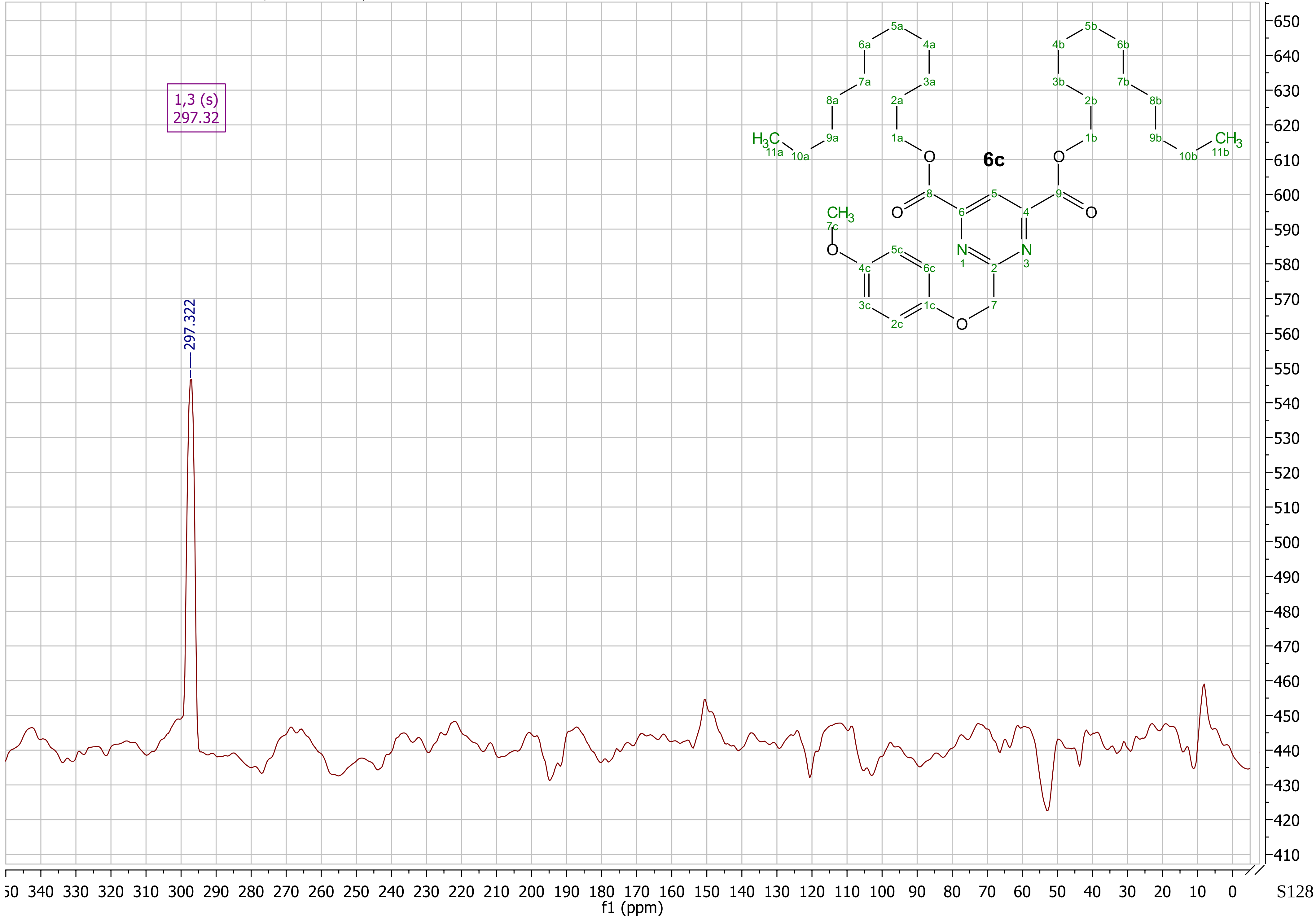

^{1}H NMR (400 MHz, $CDCl_3$) δ 8.47 (app t, J = 0.6 Hz, 1H), 7.05 – 6.90 (m, 2H), 6.88 – 6.76 (m, 2H), 5.43 (s, 2H), 5.17 (app quint, J = 6.1 Hz, 1H), 4.53 (q, J = 7.1 Hz, 2H), 3.75 (s, 3H), 1.85 – 1.57 (m, 4H), 1.46 (t, J = 7.1 Hz, 3H), 1.45 – 1.21 (m, 4H), 0.95 (t, J = 7.4 Hz, 3H), 0.89 (app t, J = 7.0 Hz, 3H).

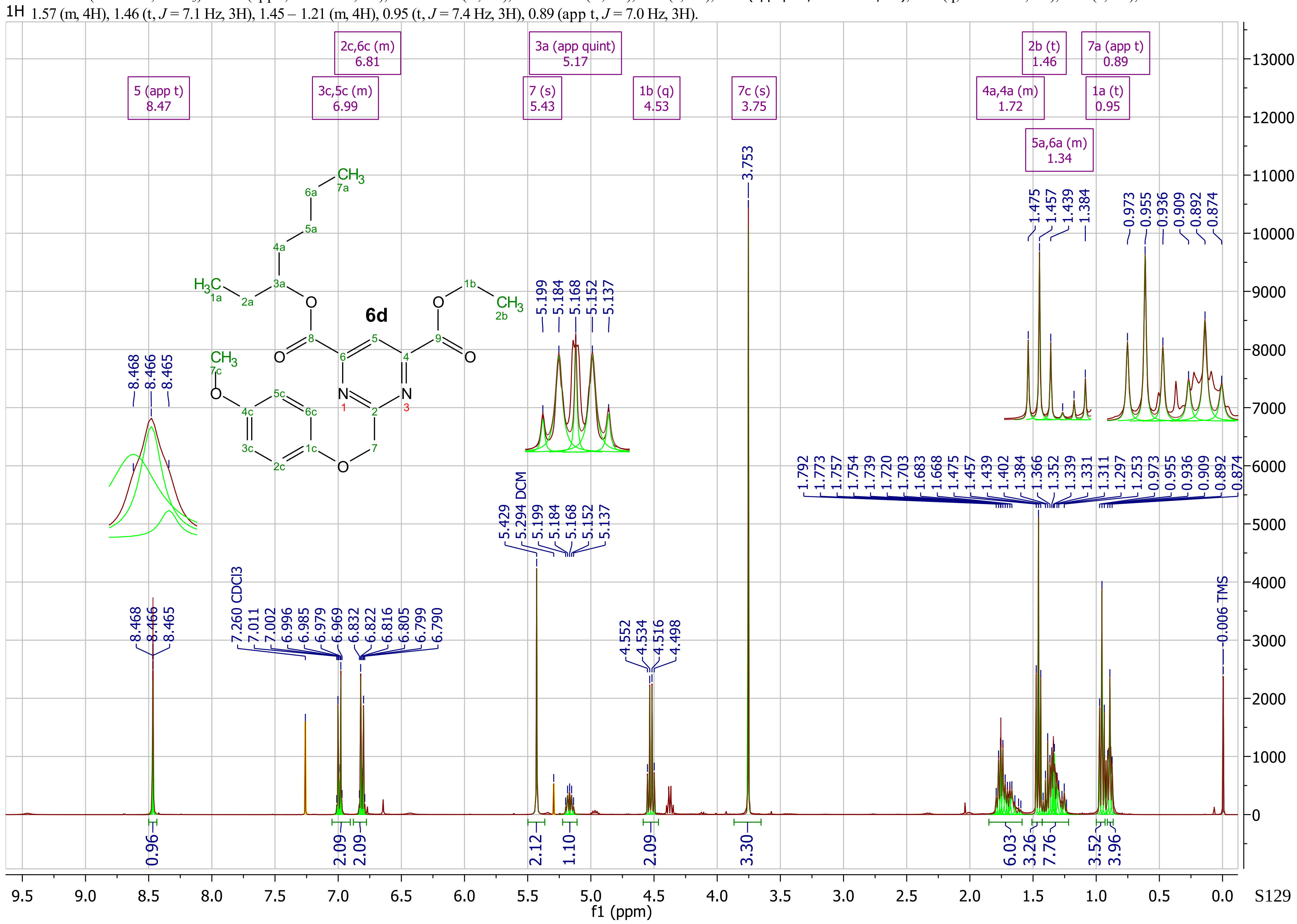

13C ^{13}C NMR (101 MHz, $CDCl_3$) δ 168.0, 163.7, 163.2, 158.4, 158.0, 154.4, 152.7, 119.0, 116.5 (sym, 2C), 114.7 (sym, 2C), 79.2, 71.5, 63.2, 55.8, 33.3, 27.6, 27.1, 22.7, 14.3, 14.1, 9.8.

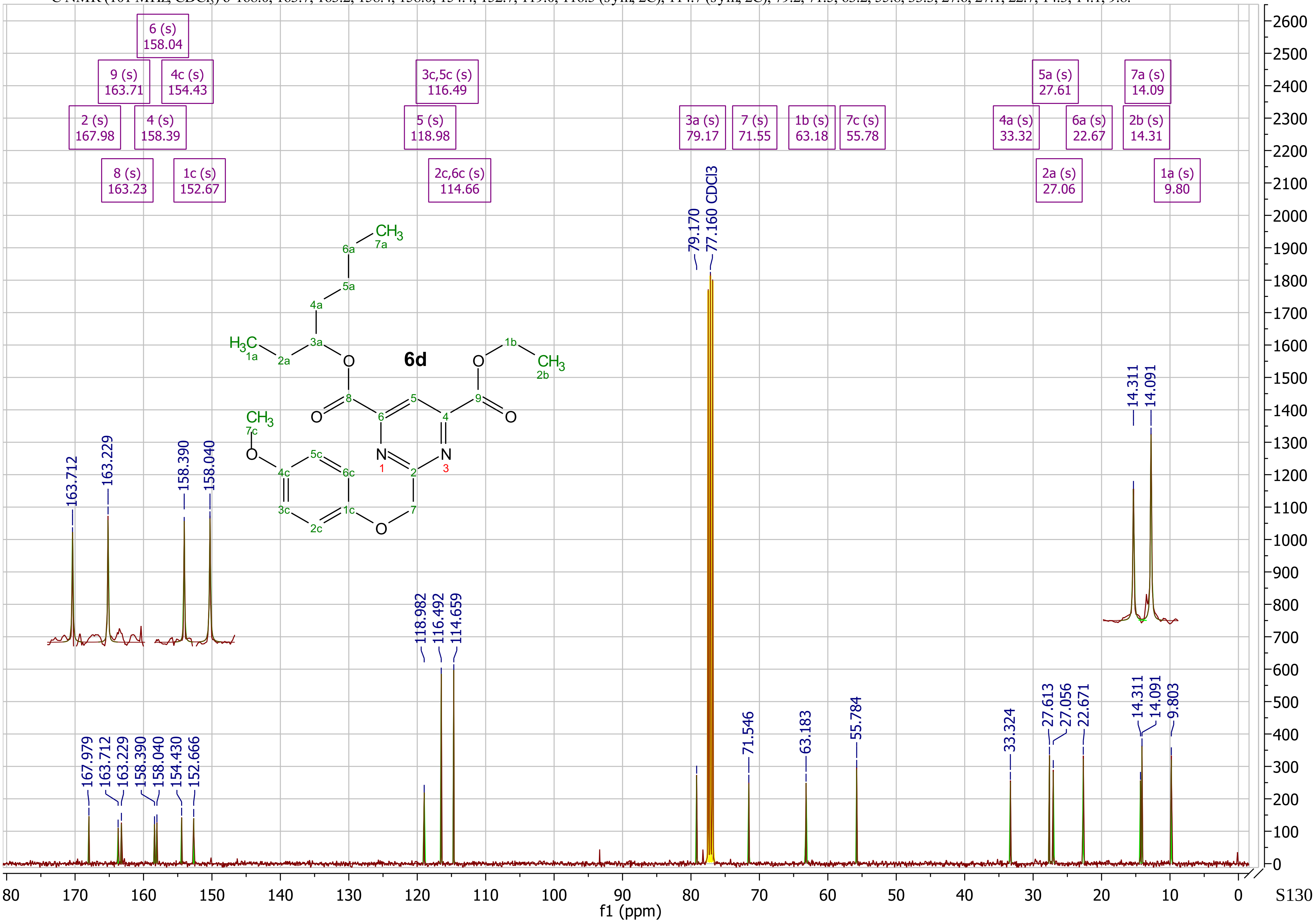

S130

13C HSQC

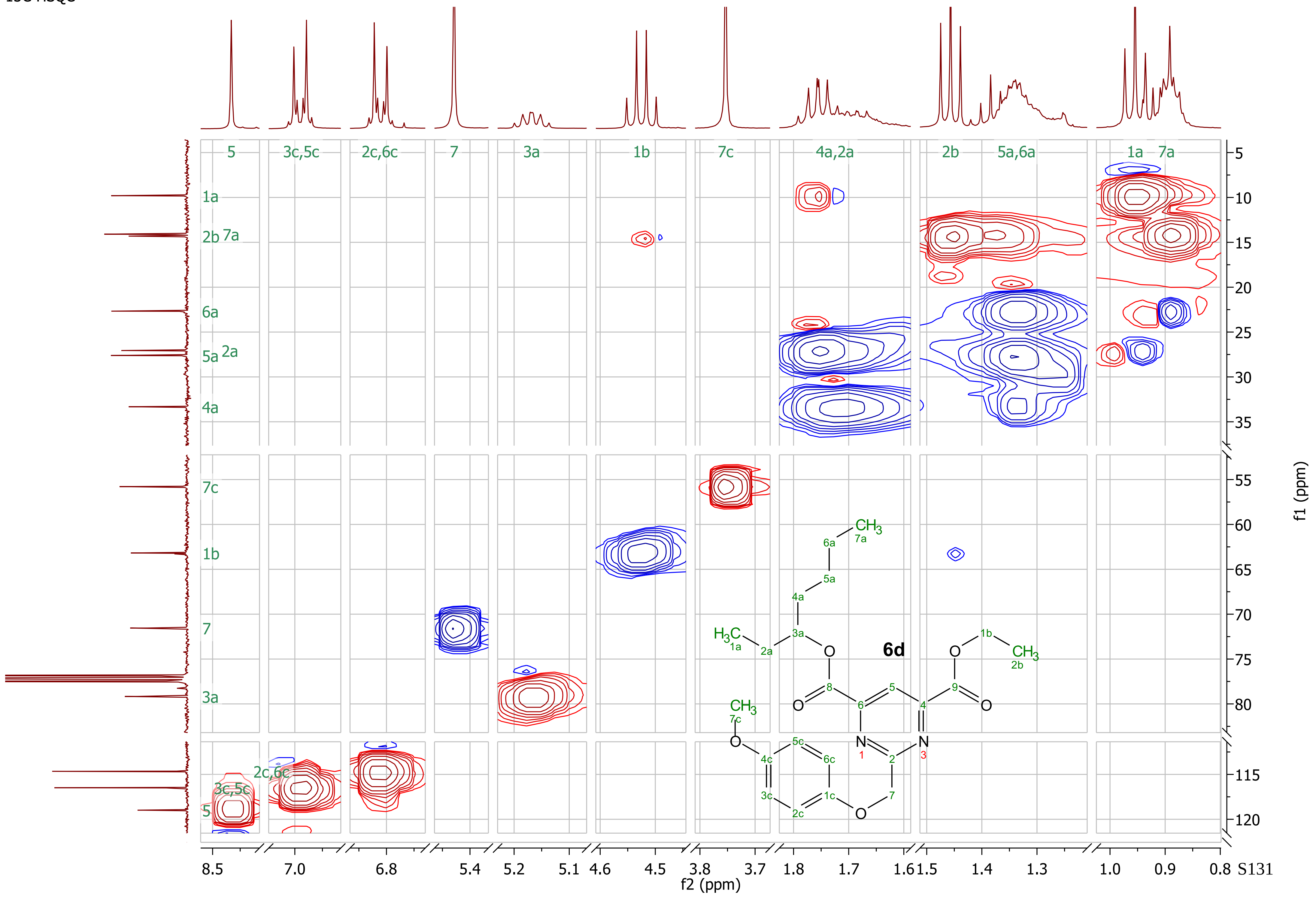

5
3c,5c
2c,6c
7
3a
1b
7c
4a,2a
2b
5a,6a
1a
7a
1a
2b 7a
6a
5a 2a
4a
7c
1b
7
3a
2c,6c
3c,5c
5
6d
f1 (ppm)
f2 (ppm)

13C HBMC

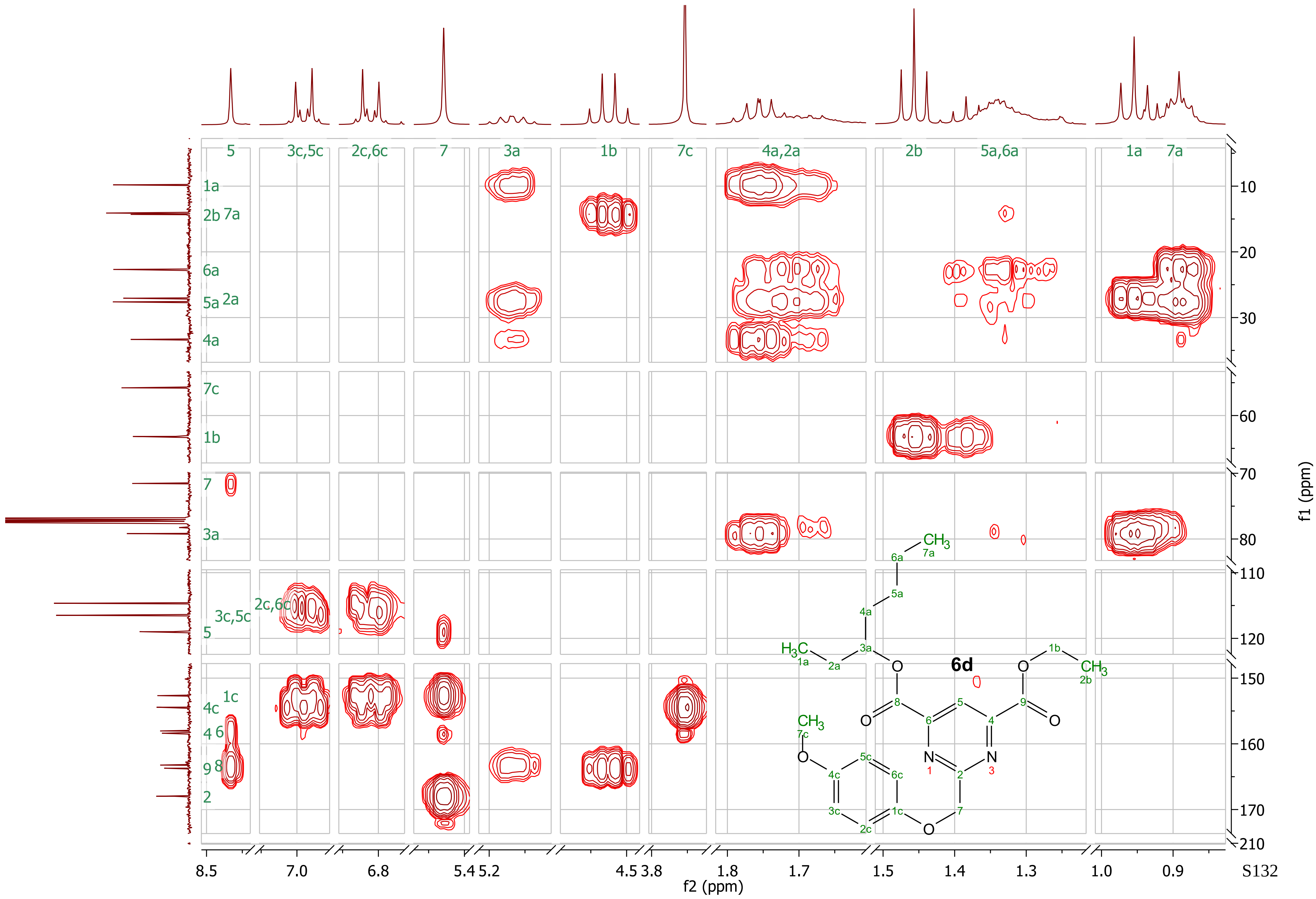

1H ^{1}H NMR (400 MHz, $CDCl_3$) δ 8.42 (app t, J = 0.6 Hz, 1H), 7.07 – 6.92 (m, 2H), 6.88 – 6.70 (m, 2H), 5.44 (s, 2H), 5.16 (app quint, J = 6.1 Hz, 2H), 3.75 (s, 3H), 1.92 – 1.55 (m, 8H), 1.42 – 1.22 (m, 8H), 0.95 (t, J = 7.4 Hz, 6H), 0.89 (app t, J = 7.1 Hz, 6H).

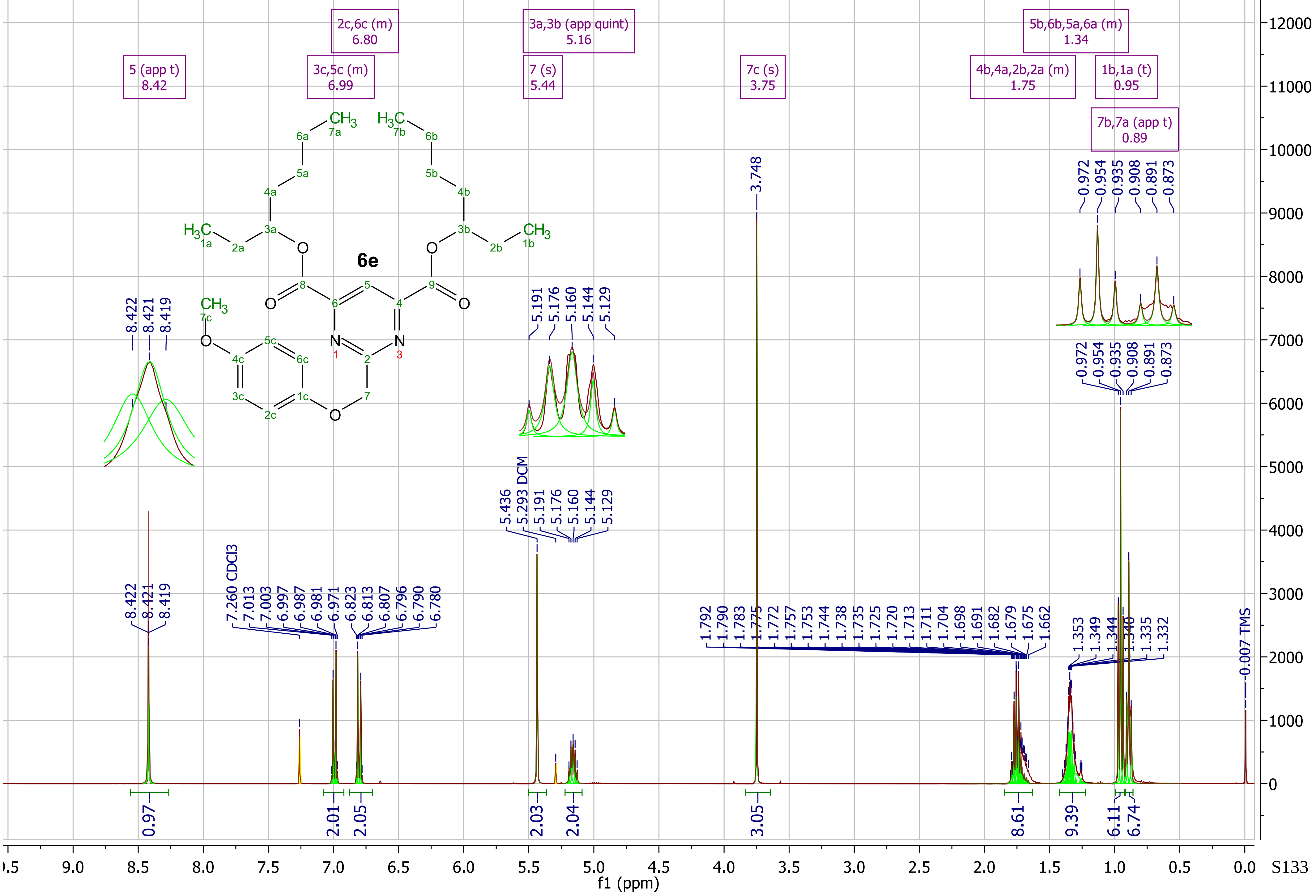

13C ^{13}C NMR (101 MHz, $CDCl_3$) δ 168.0, 163.4 (sym, 2C), 158.3 (sym, 2C), 154.4, 152.7, 118.8, 116.6 (sym, 2C), 114.6 (sym, 2C), 79.1 (sym, 2C), 71.5, 55.8, 33.3 (sym, 2C), 27.6 (sym, 2C), 27.0 (sym, 2C), 22.7 (sym, 2C), 14.1 (sym, 2C), 9.8 (sym, 2C).

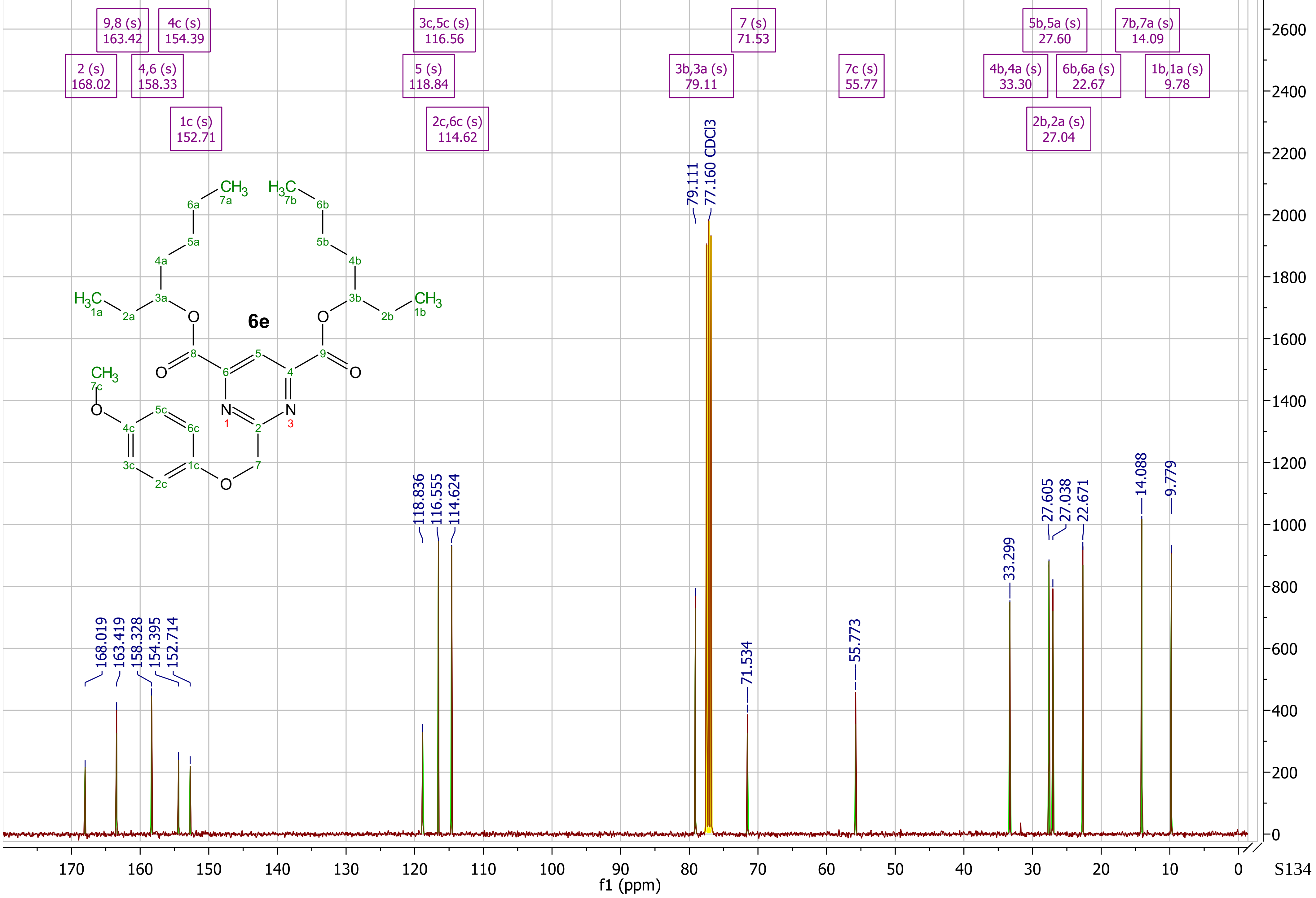

13C HSQC

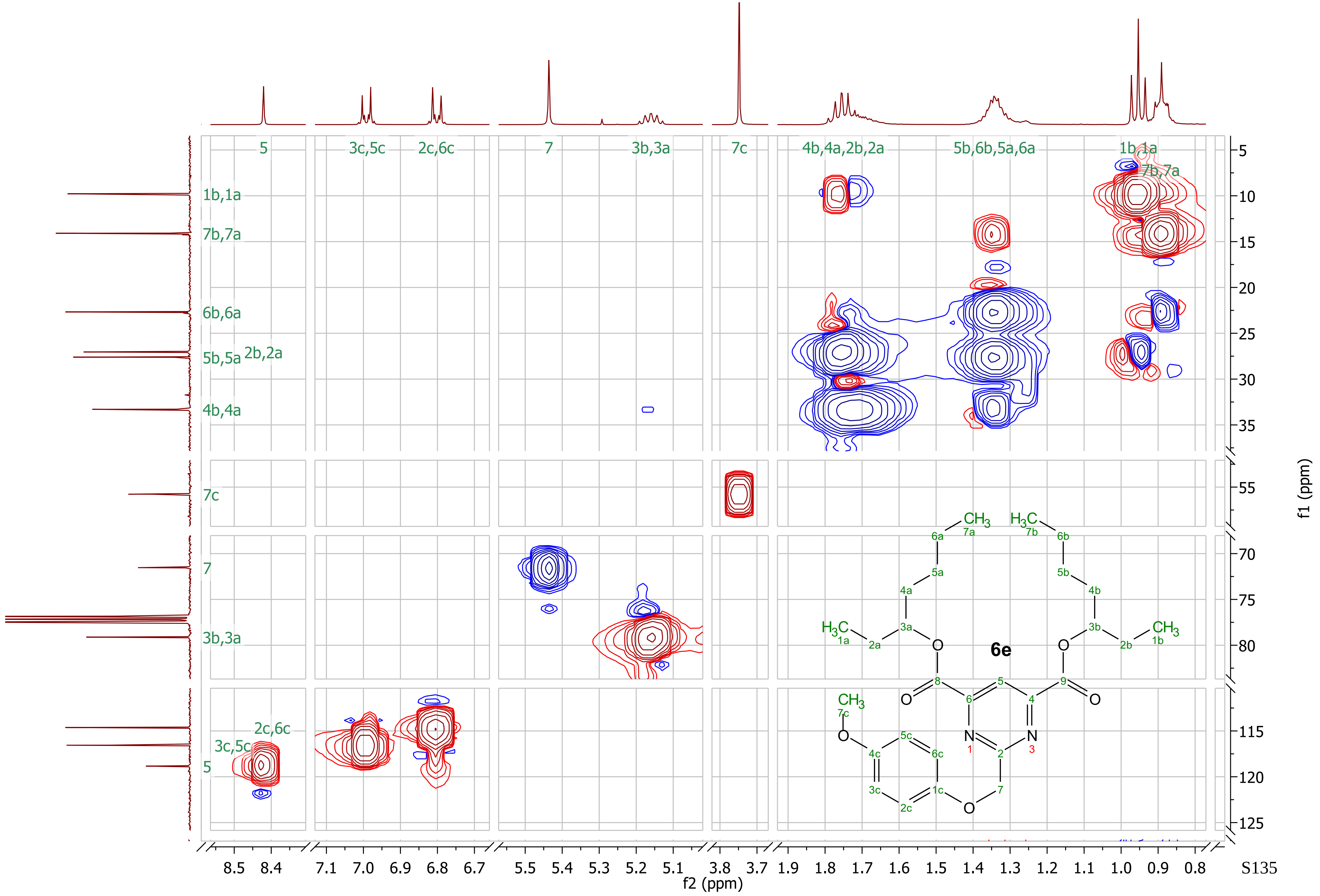

5
3c,5c
2c,6c
7
3b,3a
7c
4b,4a,2b,2a
5b,6b,5a,6a
1b,1a
7b,7a
6b,6a
5b,5a
2b,2a
4b,4a
f2 (ppm)
f1 (ppm)
6e

^{1}H NMR (400 MHz, $CDCl_3$) δ 8.42 (app t, J = 0.6 Hz, 1H), 7.03 – 6.95 (m, 2H), 6.84 – 6.77 (m, 2H), 5.44 (s, 2H), 5.16 (app quint, J = 6.2 Hz, 2H), 3.75 (s, 3H), 1.83 – 1.59 (m, 8H), 1.46 – 1.21 (m, 12H), 0.95 (t, J = 7.4 Hz, 6H), 0.87 (app t, J = 6.8 Hz, 6H).

1H

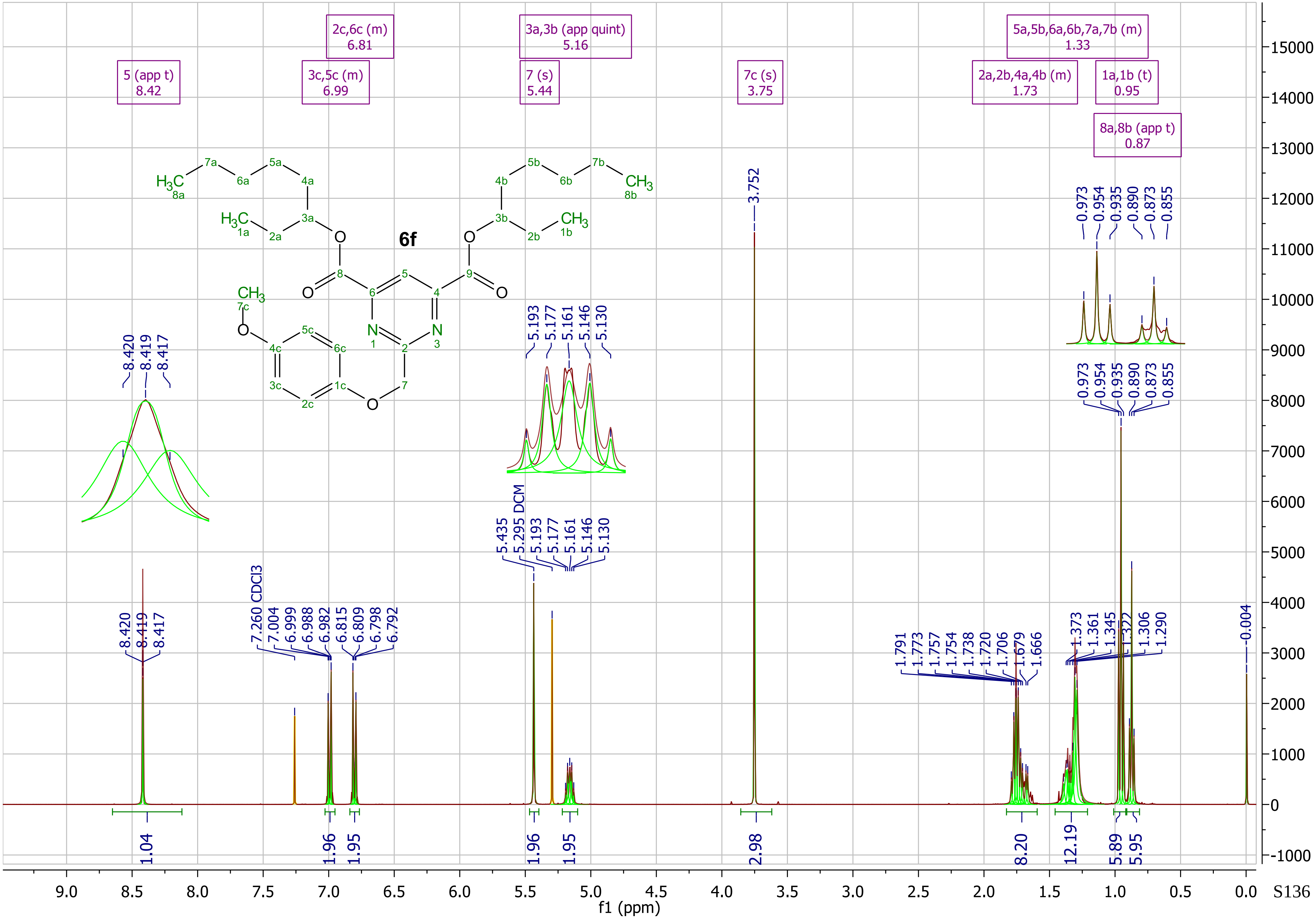

13C ^{13}C NMR (101 MHz, $CDCl_3$) δ 168.0, 163.4 (sym, 2C), 158.3 (sym, 2C), 154.4, 152.7, 118.8, 116.6 (sym, 2C), 114.6 (sym, 2C), 79.1 (sym, 2C), 71.6, 55.8, 33.6 (sym, 2C), 31.8 (sym, 2C), 27.0 (sym, 2C), 25.1 (sym, 2C), 22.6 (sym, 2C), 14.1 (sym, 2C), 9.8 (sym, 2C).

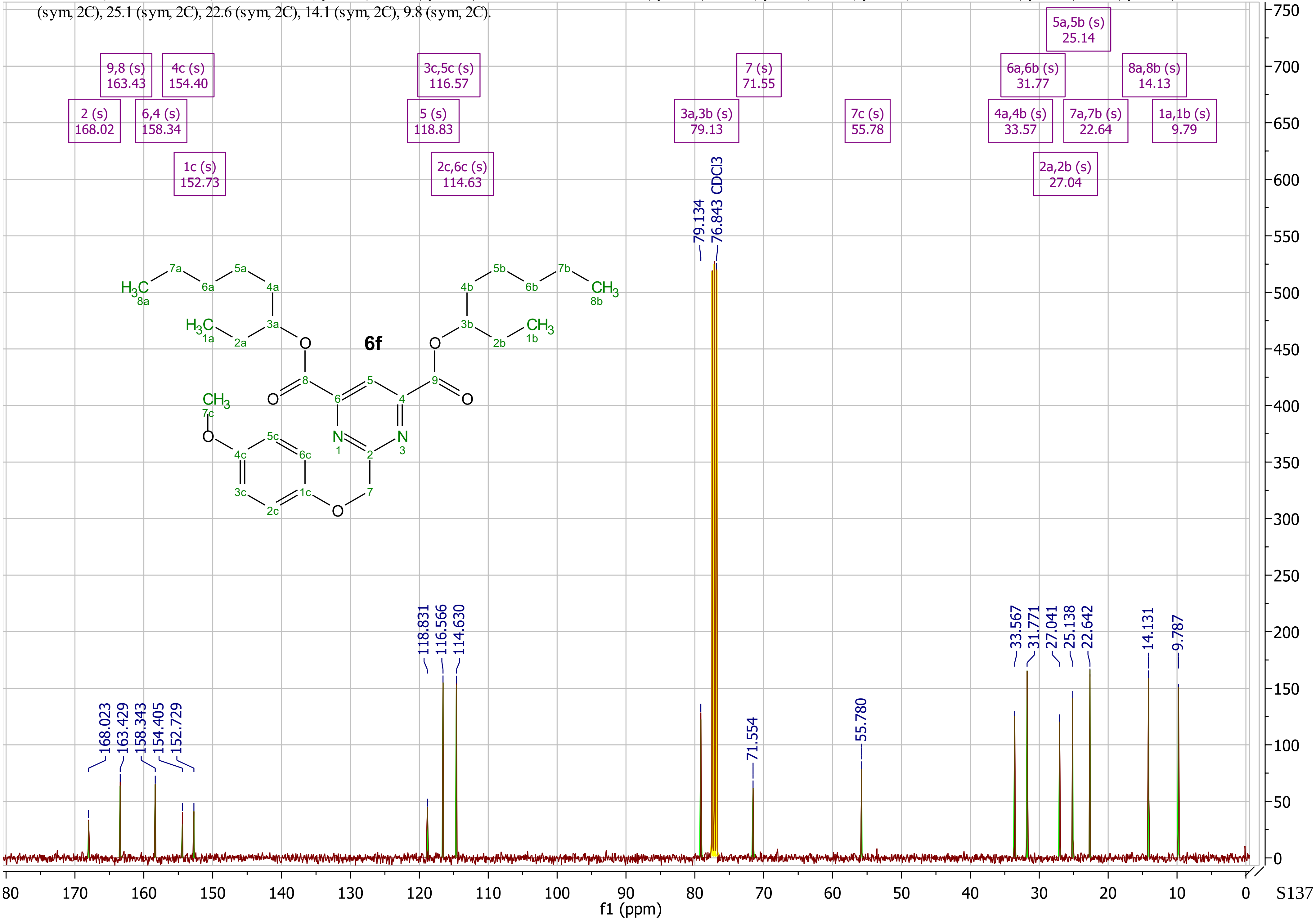

13C HSQC

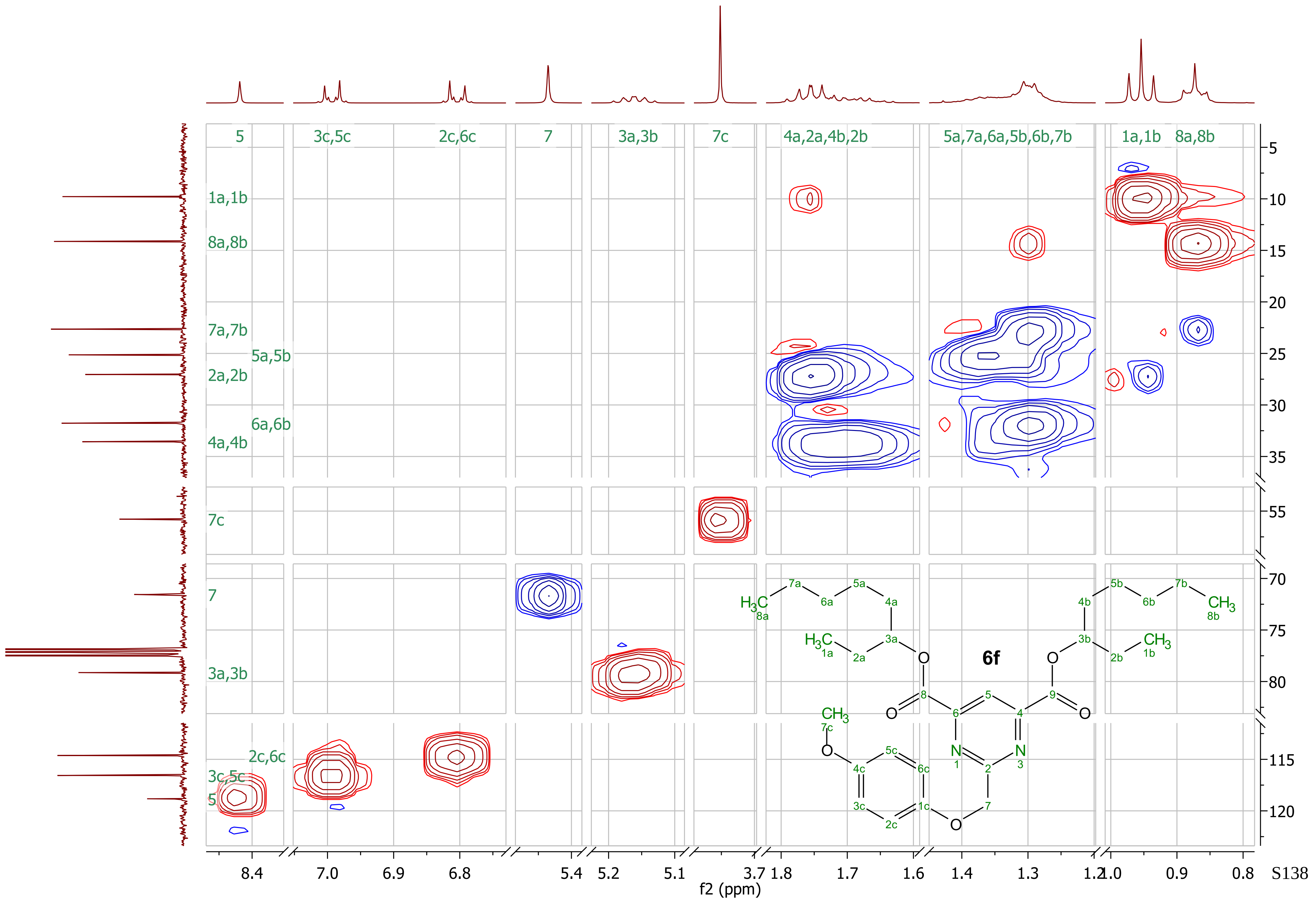

5
3c,5c
2c,6c
7
3a,3b
7c
4a,2a,4b,2b
5a,7a,6a,5b,6b,7b
1a,1b
8a,8b
1a,1b
8a,8b
7a,7b
5a,5b
2a,2b
6a,6b
4a,4b
7c
7
3a,3b
2c,6c
3c,5c
5
6f
f2 (ppm)
f1 (ppm)

13C HMBC

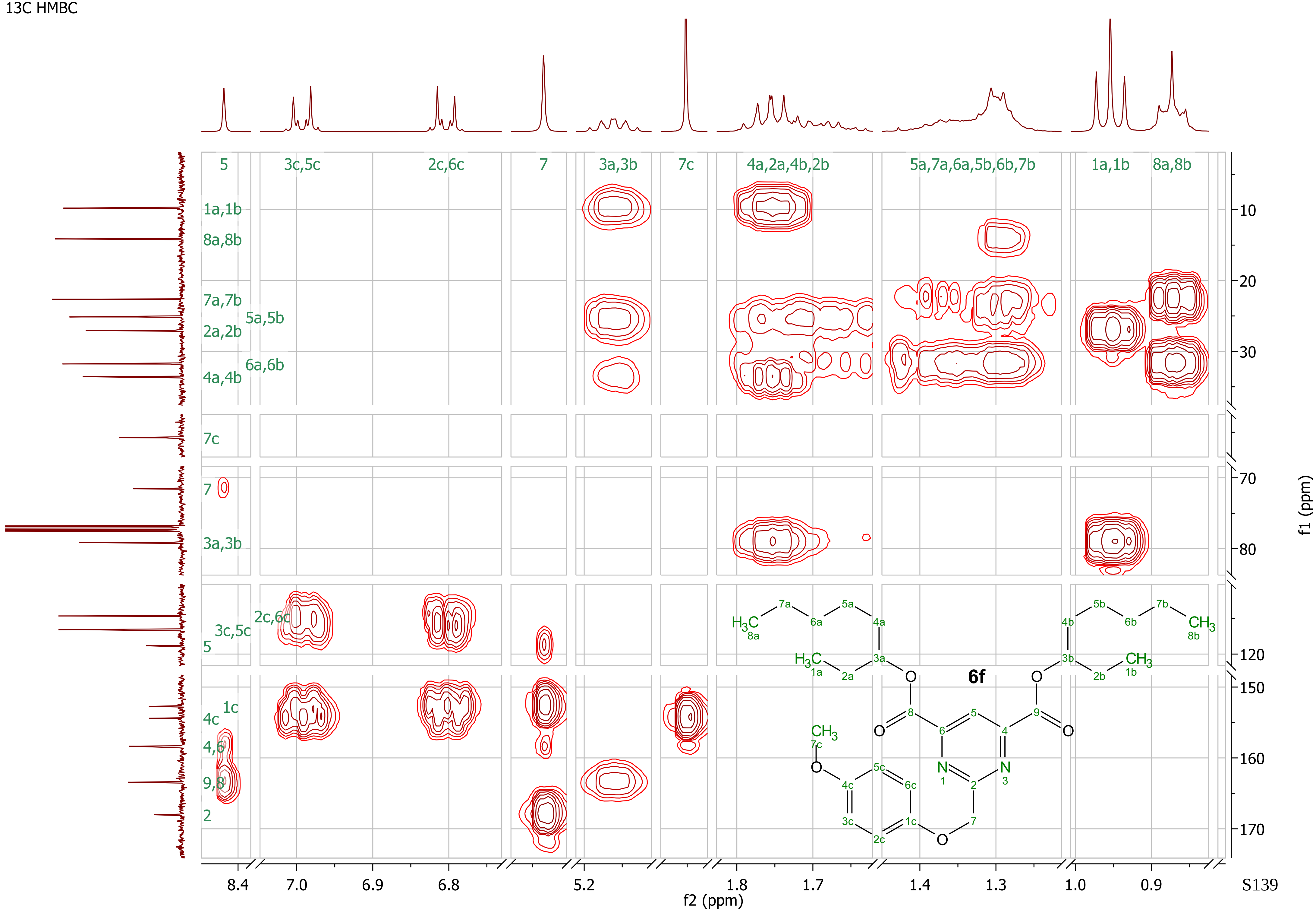

5
3c,5c
2c,6c
7
3a,3b
7c
4a,2a,4b,2b
5a,7a,6a,5b,6b,7b
1a,1b
8a,8b
1a,1b
8a,8b
7a,7b
5a,5b
2a,2b
6a,6b
4a,4b
7c
7
3a,3b
2c,6c
3c,5c
5
1c
4c
4,6
9,8
2
6f
f2 (ppm)
f1 (ppm)

ALI-009_Fr1-3_CDCl3.5.ser — 15N HMBC

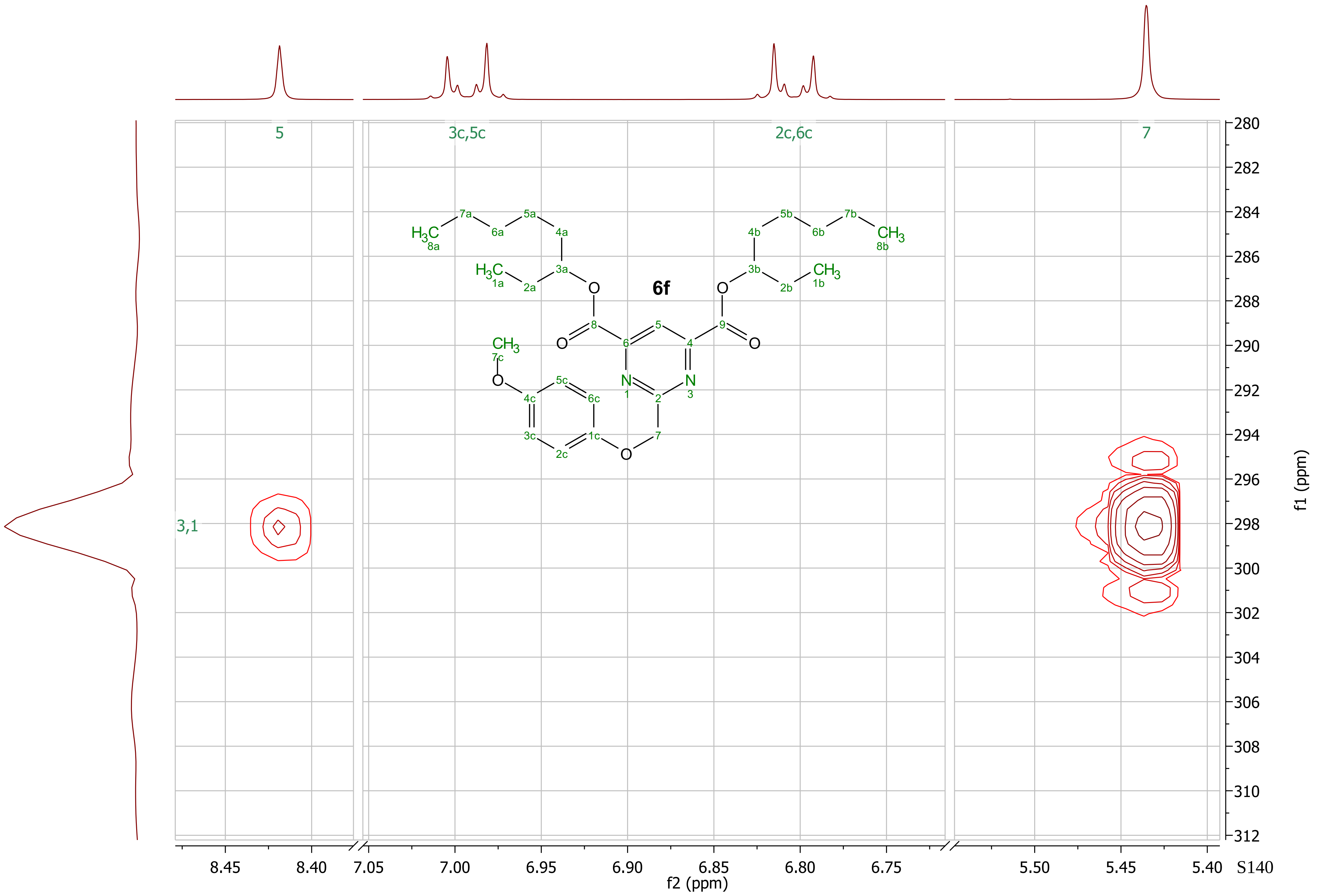

15N HMBC f1 Projection ^{15}N NMR (41 MHz, $CDCl_3$) δ 298.06.

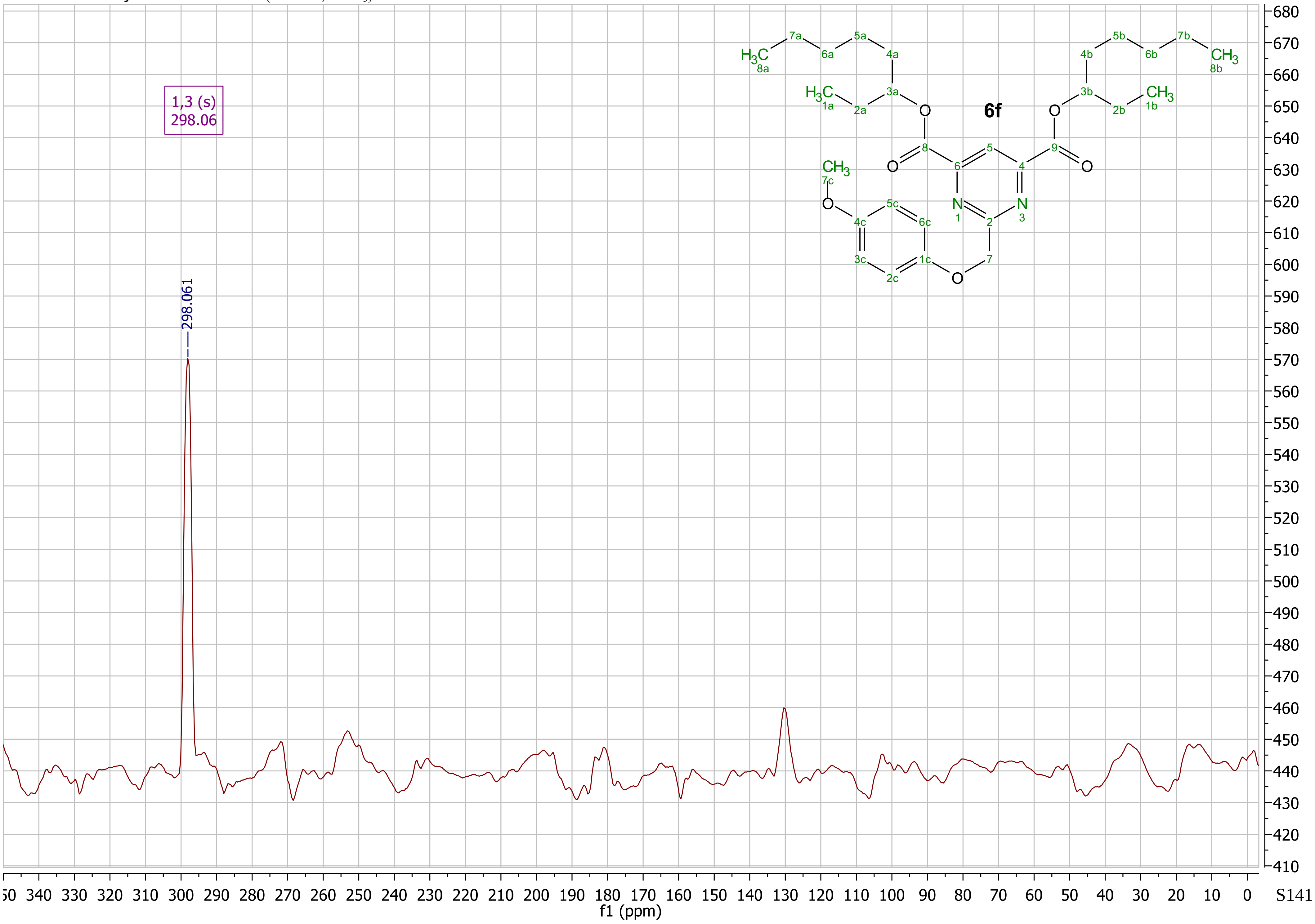

1H ^{1}H NMR (400 MHz, $CDCl_3$) δ 8.53 (app t, J = 0.6 Hz, 1H), 7.73 (s, 2H), 7.69 – 7.60 (m, 4H), 7.52 (t, J = 7.7 Hz, 2H), 7.02 – 6.89 (m, 2H), 6.83 – 6.74 (m, 2H), 5.51 (s, 4H), 5.43 (s, 2H), 3.74 (s, 3H).

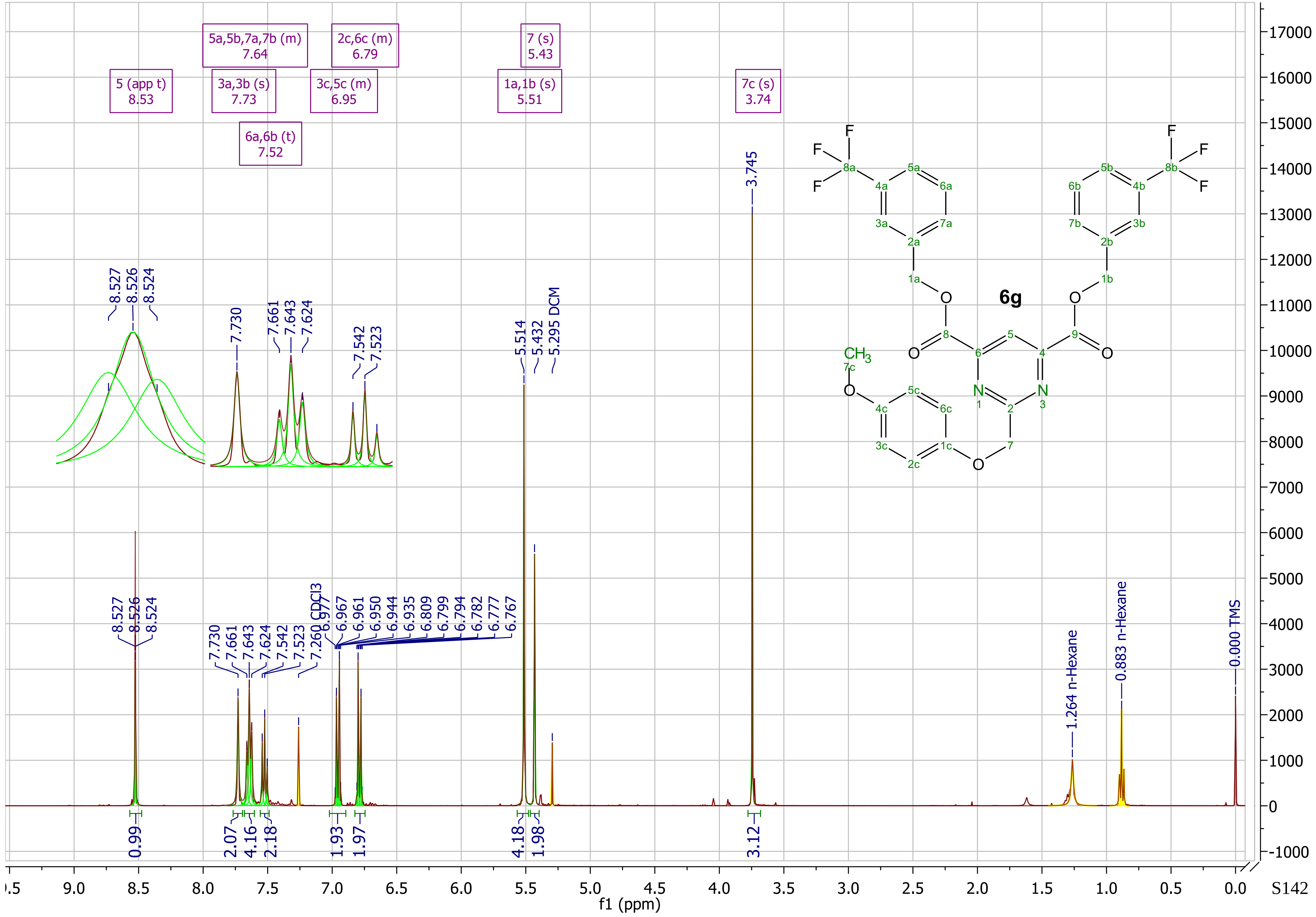

^{13}C NMR (101 MHz, CDCl₃) δ 168.3, 163.2 (sym, 2C), 157.6 (sym, 2C), 154.5, 152.5, 135.7 (sym, 2C), 132.1 (app q, *J* = 1.1 Hz, sym, 2C), 131.3 (q, *J* = 32.5 Hz, sym, 2C), 129.5 (sym, 2C), 125.8 (q, *J* = 3.7 Hz, sym, 2C), 125.5 (q, *J* = 3.8 Hz, sym, 2C), 124.0 (q, *J* = 272.3 Hz, sym, 2C), 119.4, 116.4 (sym, 2C), 114.7 (sym, 2C), 71.3, 67.7 (sym, 2C), 55.8.

13C

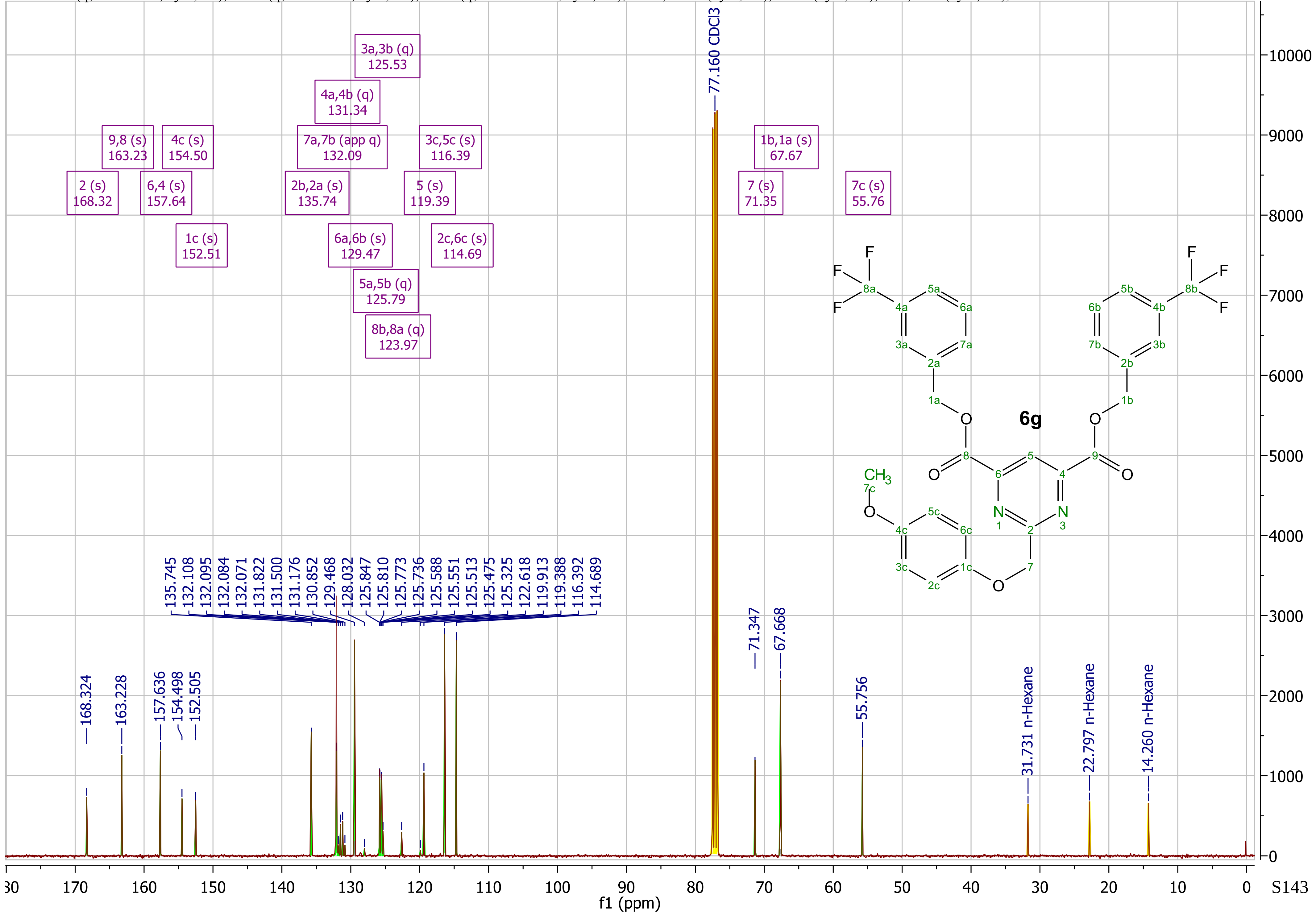

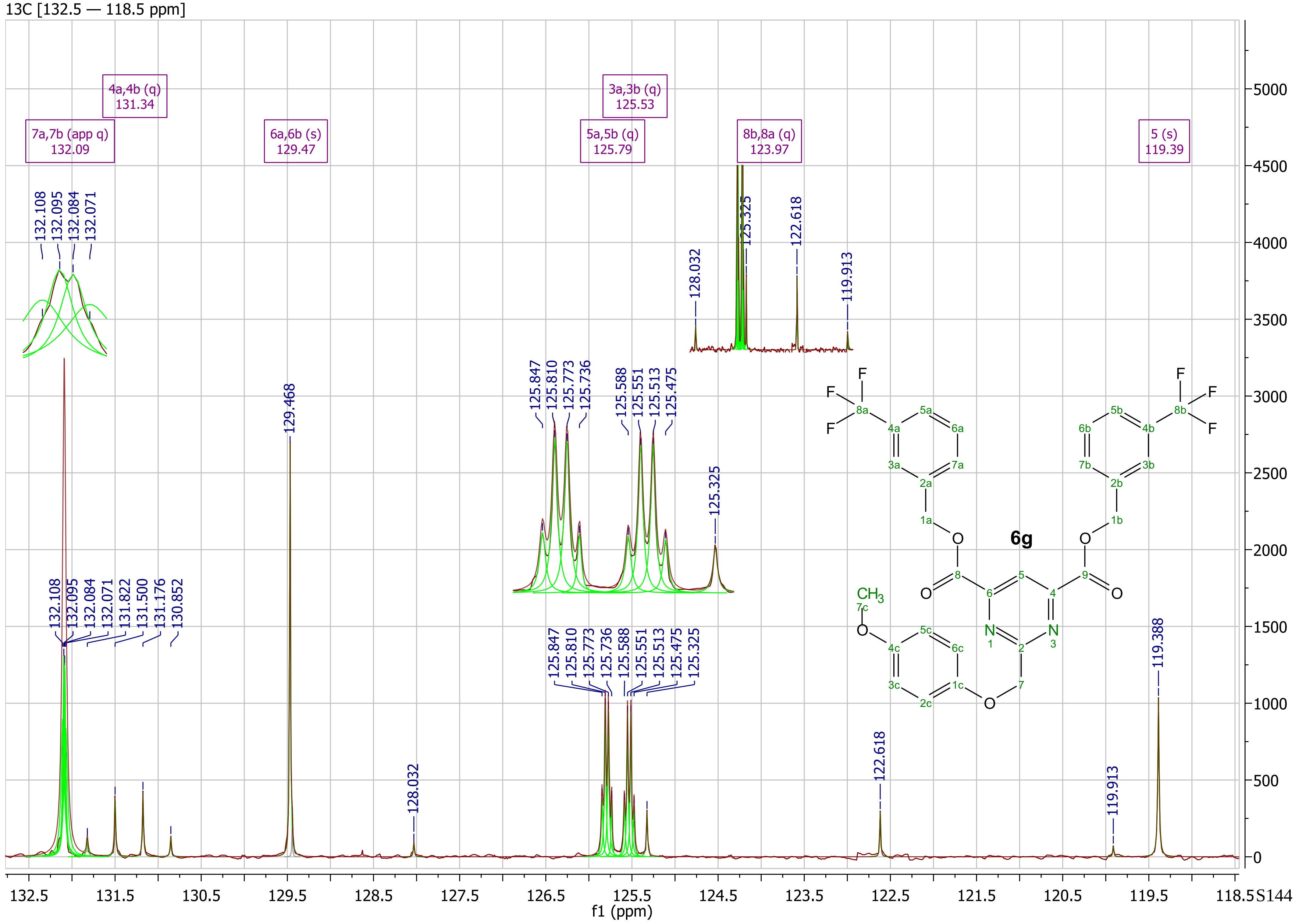

13C [132.5 — 118.5 ppm]
7a,7b (app q) 132.09
4a,4b (q) 131.34
6a,6b (s) 129.47
5a,5b (q) 125.79
3a,3b (q) 125.53
8b,8a (q) 123.97
5 (s) 119.39
132.108
132.095
132.084
132.071
131.822
131.500
131.176
130.852
129.468
128.032
125.847
125.810
125.773
125.736
125.588
125.551
125.513
125.475
125.325
122.618
119.913
119.388
6g
f1 (ppm)

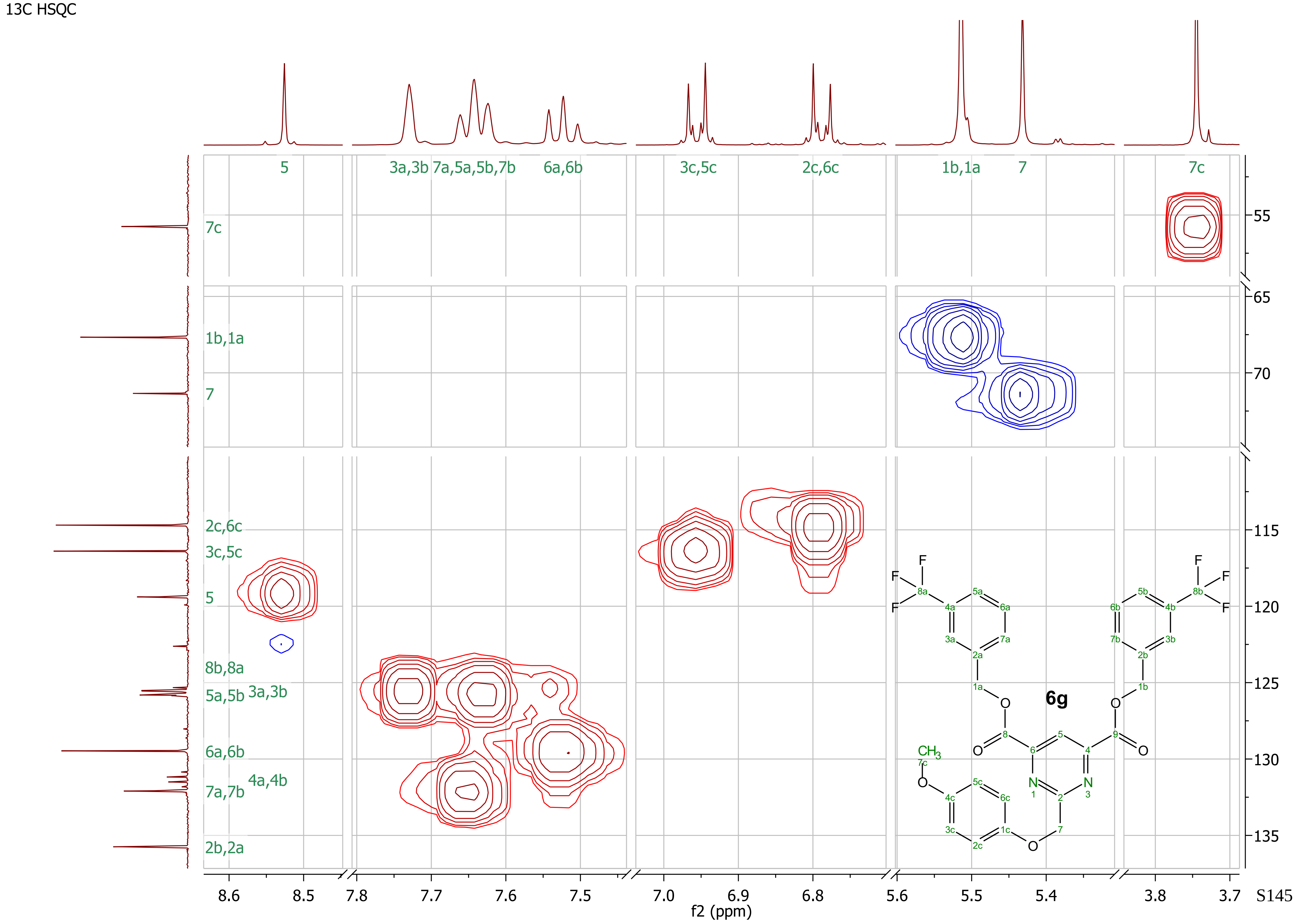
13C HSQC
5
3a,3b 7a,5a,5b,7b
6a,6b
3c,5c
2c,6c
1b,1a
7
7c
1b,1a
2c,6c
3c,5c
8b,8a
5a,5b 3a,3b
6a,6b
4a,4b
7a,7b
2b,2a
6g
f1 (ppm)
f2 (ppm)

13C HMBC

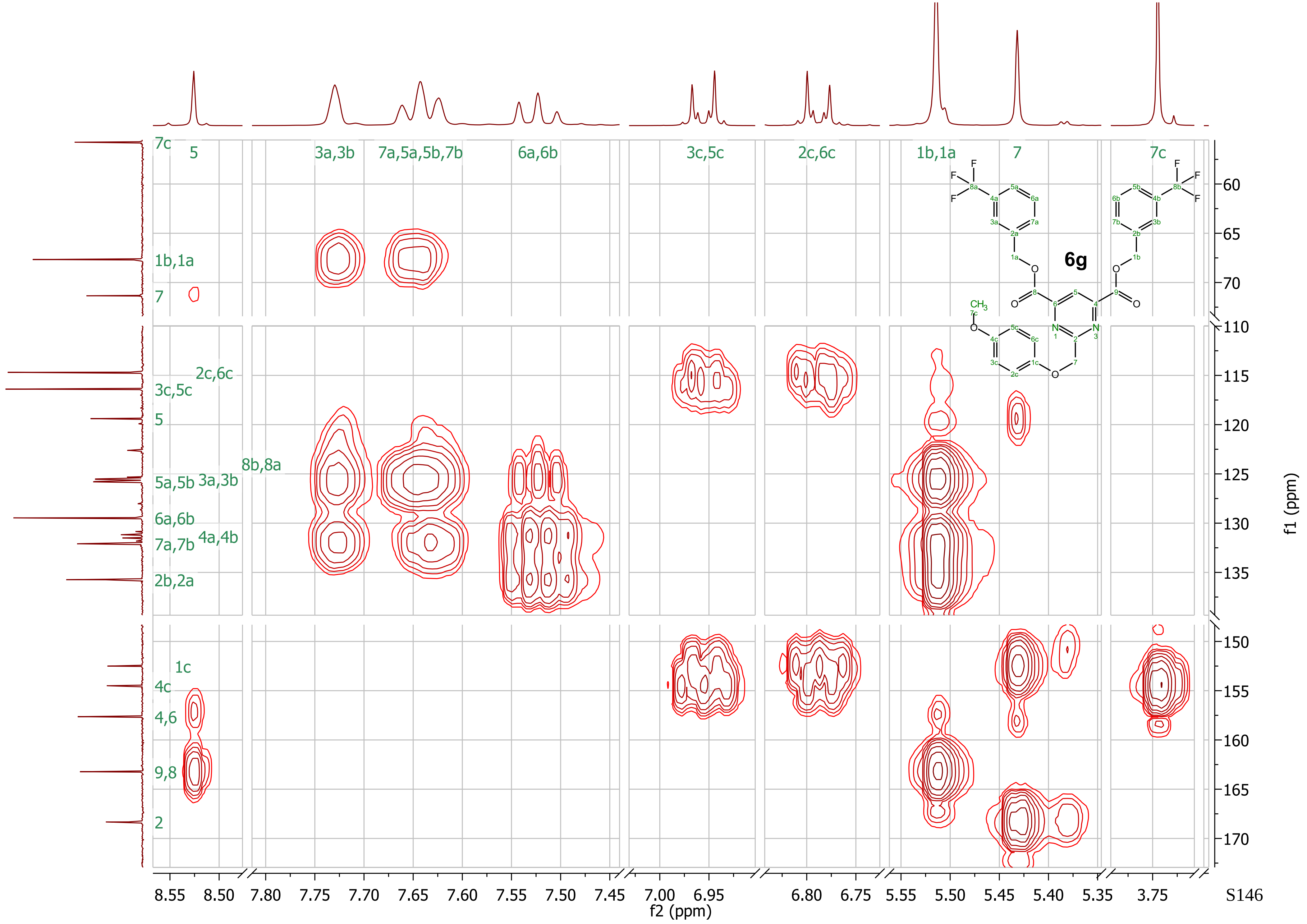

5
3a,3b
7a,5a,5b,7b
6a,6b
3c,5c
2c,6c
1b,1a
7
7c
6g
7c
1b,1a
7
2c,6c
3c,5c
5
8b,8a
5a,5b
3a,3b
6a,6b
7a,7b
4a,4b
2b,2a
1c
4c
4,6
9,8
2
f2 (ppm)
f1 (ppm)

15N HMBC

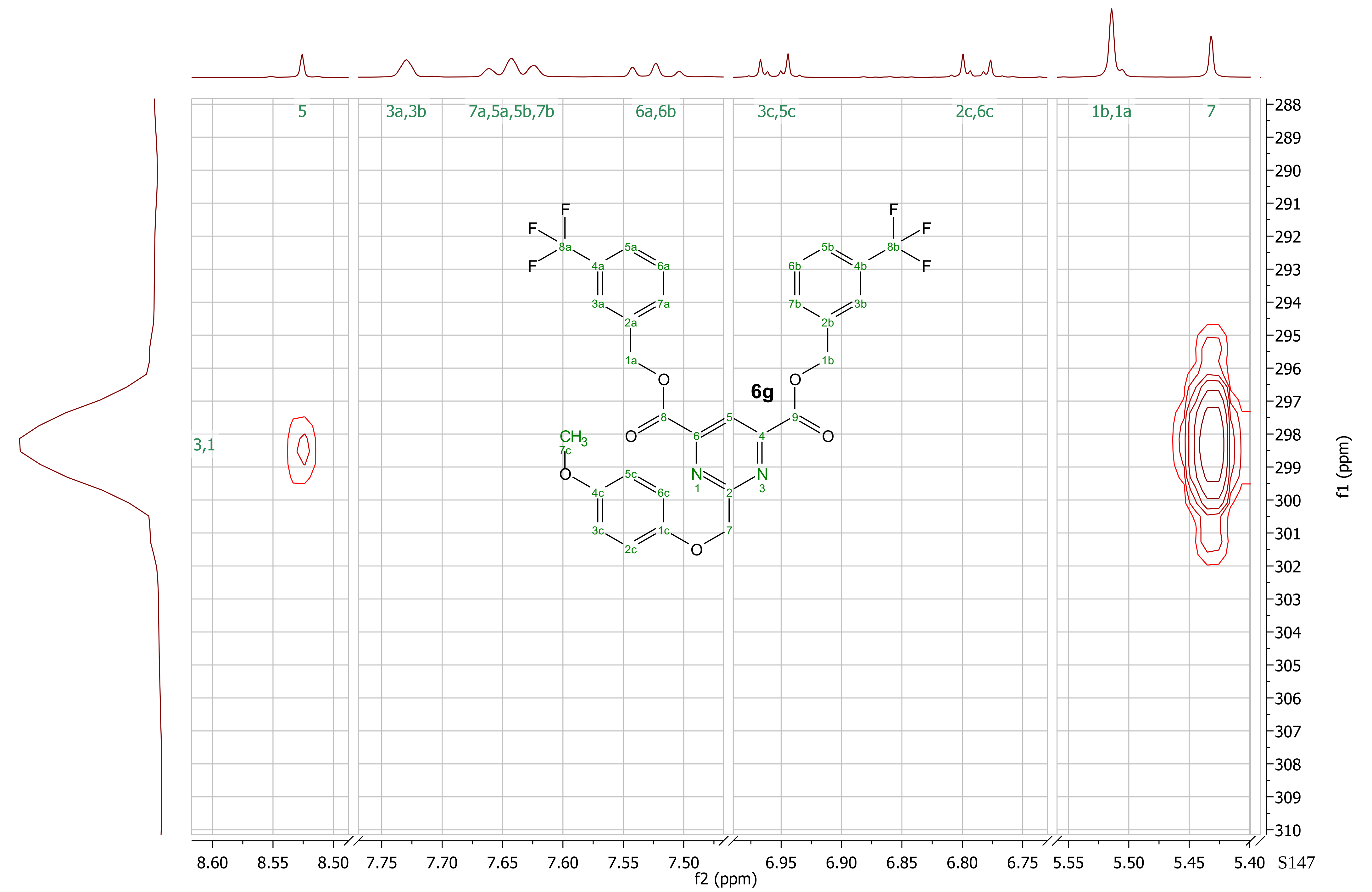

15N HMBC f1 Projection ^{15}N NMR (41 MHz, $CDCl_3$) δ 298.30.

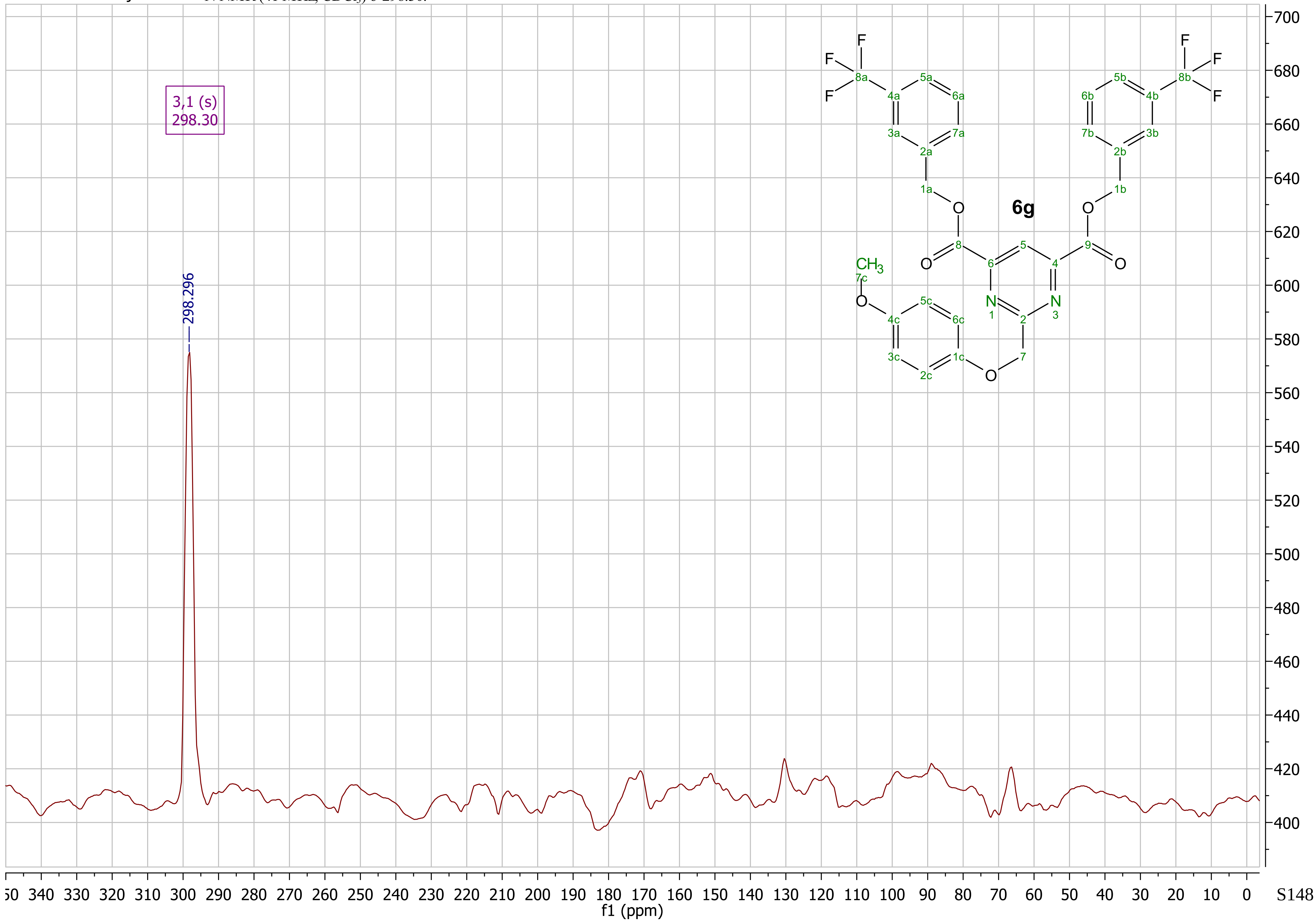

1H ^{1}H NMR (400 MHz, $CDCl_3$) δ 11.05 (br s, 1H), 6.98 – 6.89 (m, 2H), 6.89 – 6.79 (m, 2H), 4.98 (s, 2H), 4.43 (q, J = 7.1 Hz, 2H), 3.76 (s, 3H), 2.25 (s, 3H), 1.41 (t, J = 7.1 Hz, 3H).

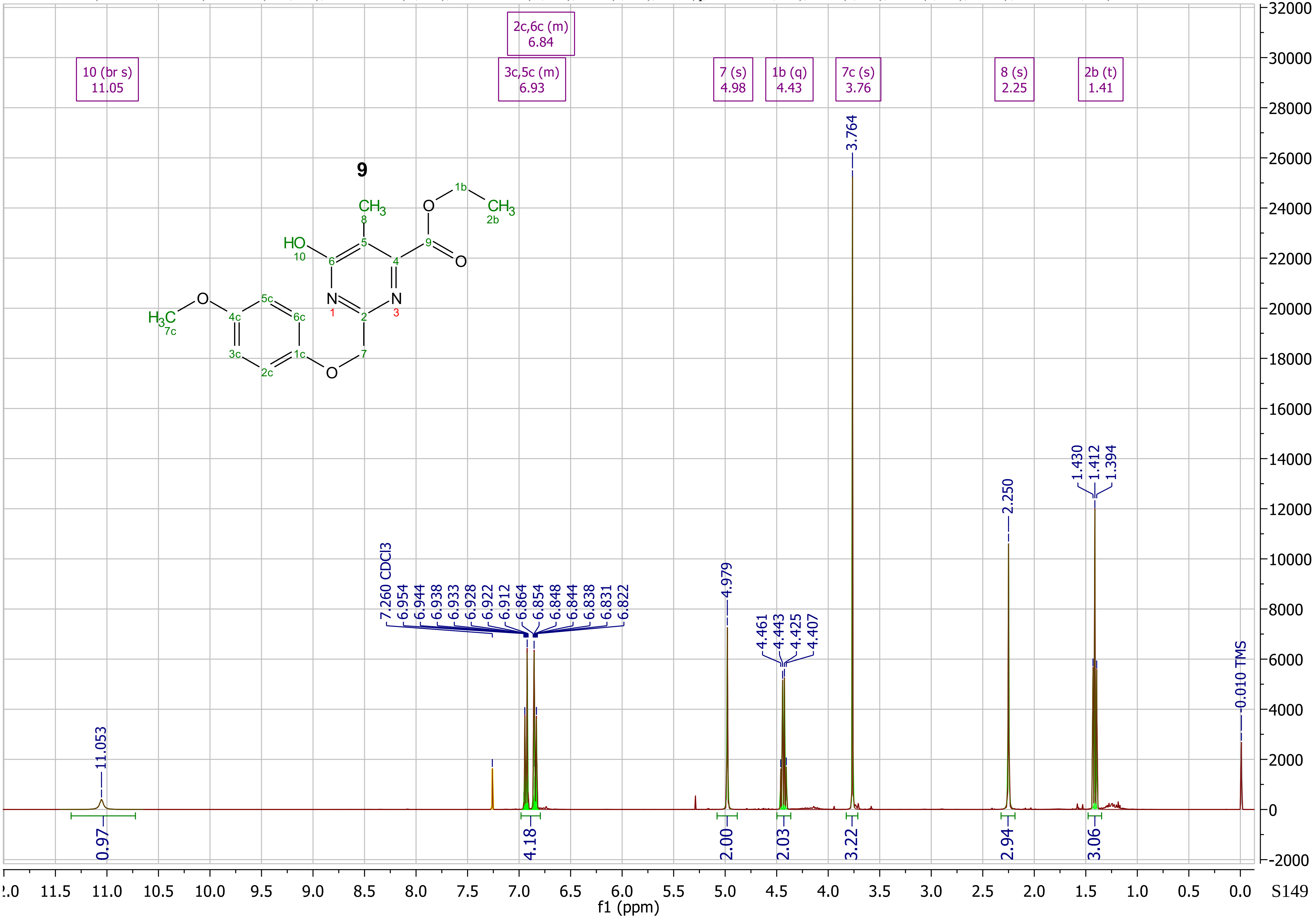

13C ^{13}C NMR (101 MHz, $CDCl_3$) δ 165.5, 163.5, 155.1, 154.7, 151.1, 150.0, 125.3, 115.9 (sym, 2C), 115.0 (sym, 2C), 67.3, 62.4, 55.8, 14.3, 11.8.

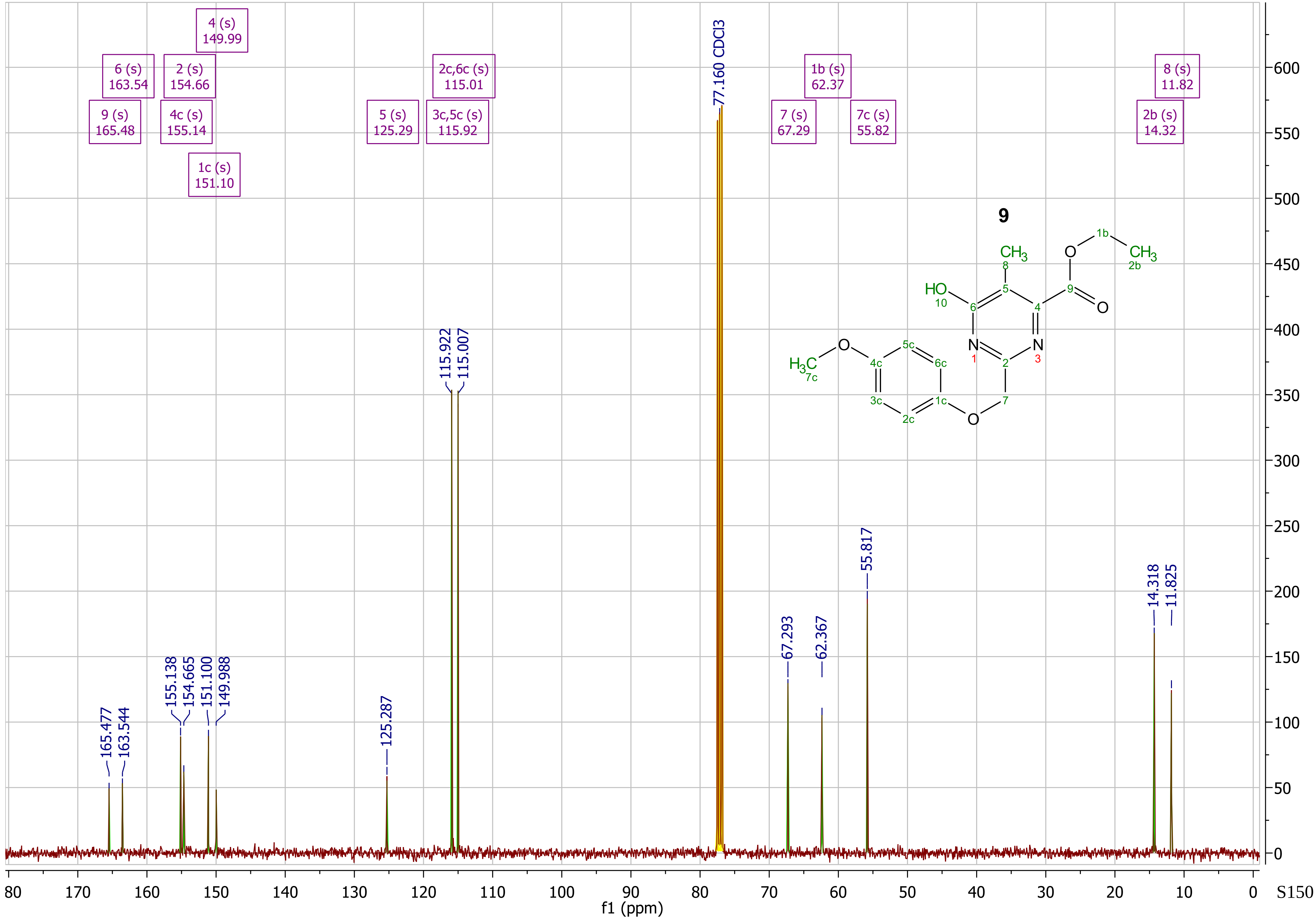

13C HSQC

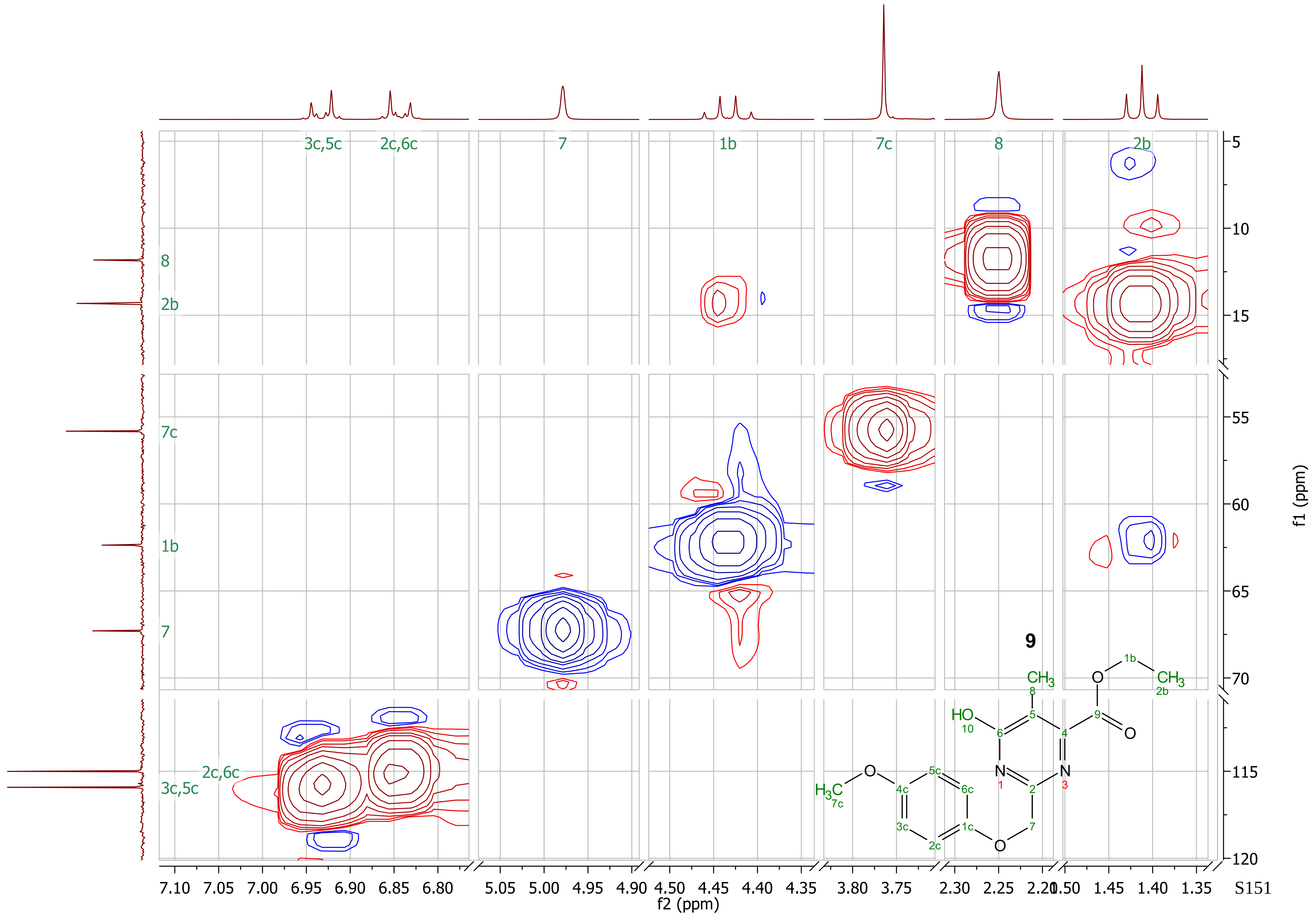
3c,5c
2c,6c
7
1b
7c
8
2b
8
2b
7c
1b
7
2c,6c
3c,5c
9
f1 (ppm)
f2 (ppm)
7.10 7.05 7.00 6.95 6.90 6.85 6.80
5.05 5.00 4.95 4.90
4.50 4.45 4.40 4.35
3.80 3.75
2.30 2.25 2.20
1.50 1.45 1.40 1.35
5
10
15
55
60
65
70
115
120

13C HMBC

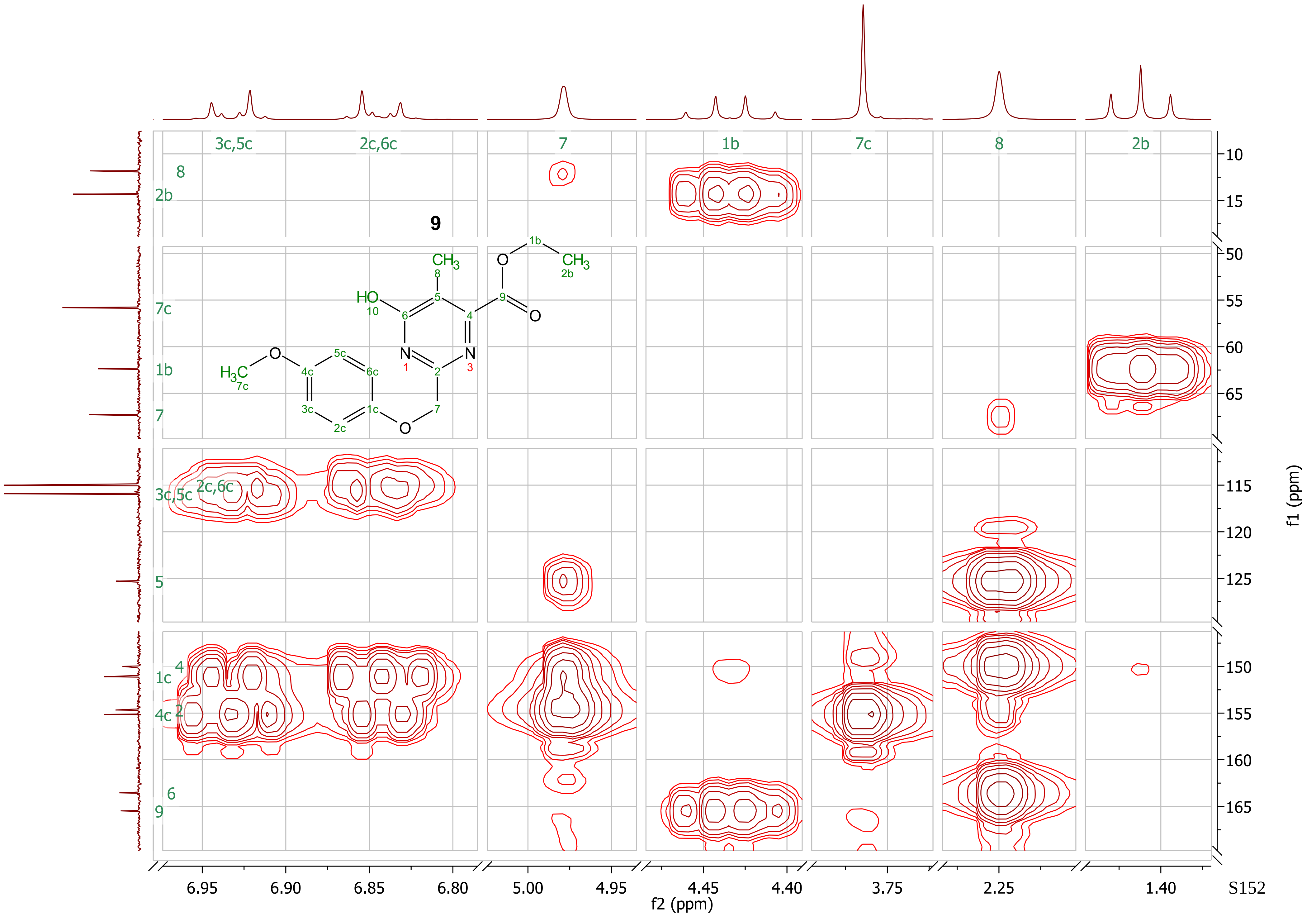
3c,5c
2c,6c
7
1b
7c
8
2b
9
HO
10
CH3
O
O
N
N
1
3
2
4
5
6
H3C
7c
4c
5c
6c
3c
2c
1c
O
1c
4
2
4c
6
f1 (ppm)
f2 (ppm)
6.95
6.90
6.85
6.80
5.00
4.95
4.45
4.40
3.75
2.25
1.40

1H ^{1}H NMR (400 MHz, $CDCl_3$) δ 7.04 – 6.88 (m, 2H), 6.88 – 6.73 (m, 2H), 5.20 (s, 2H), 4.47 (q, *J* = 7.2 Hz, 2H), 3.76 (s, 3H), 2.50 (s, 3H), 1.42 (t, *J* = 7.2 Hz, 3H).

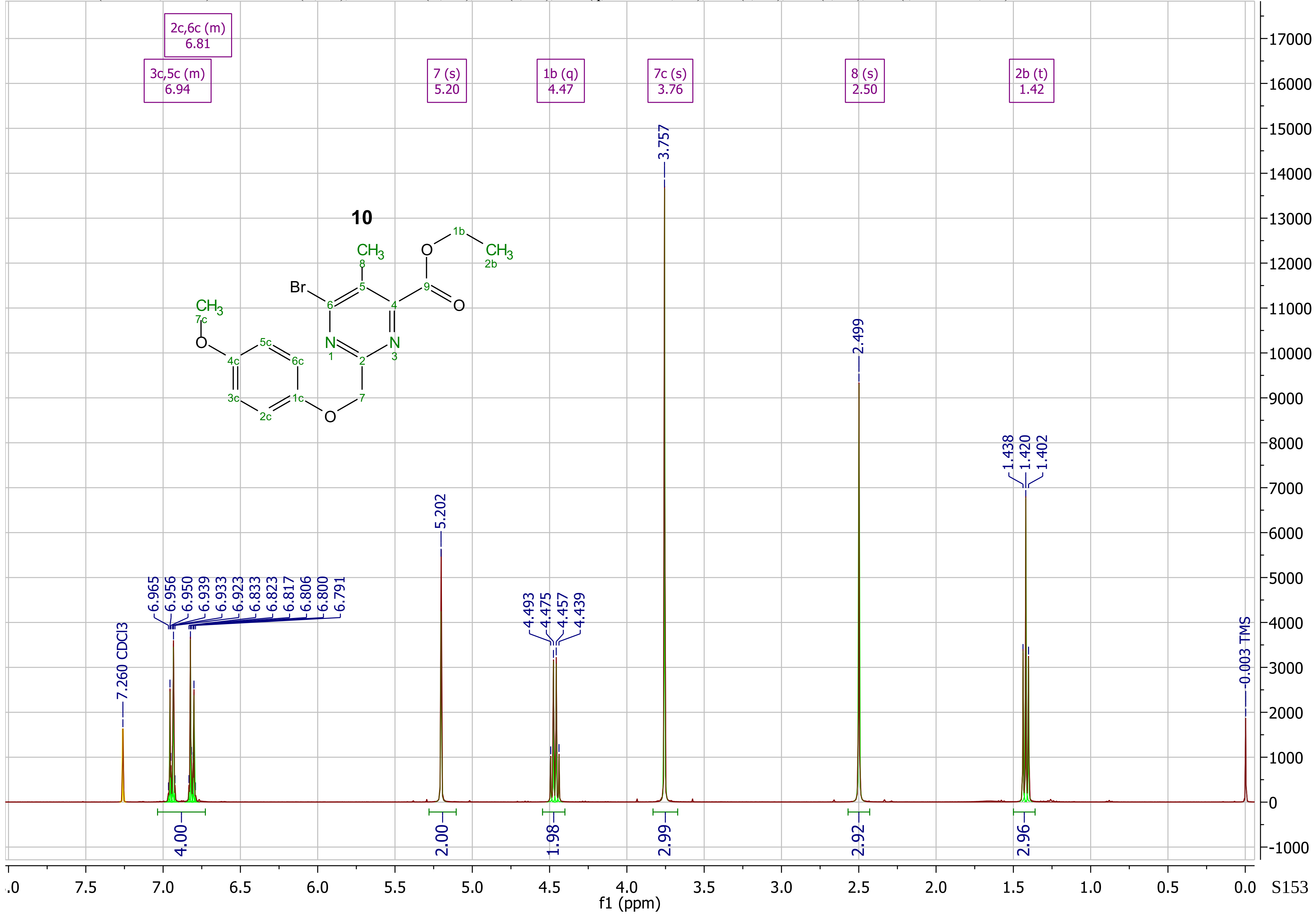

13C ^{13}C NMR (101 MHz, $CDCl_3$) δ 164.6, 164.0, 158.2, 156.9, 154.5, 152.6, 130.2, 116.4 (sym, 2C), 114.7 (sym, 2C), 71.0, 62.9, 55.8, 17.8, 14.2.

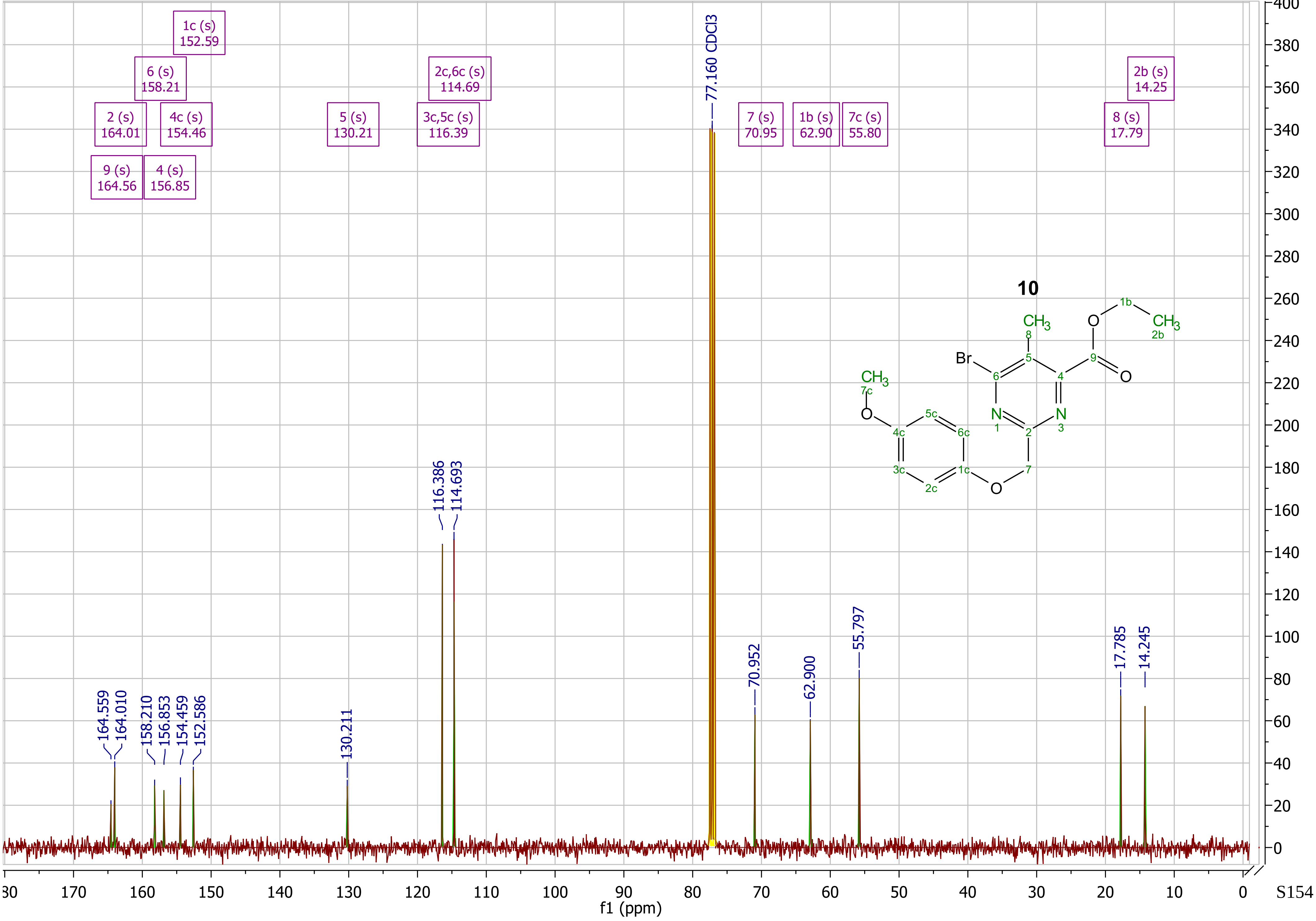

13C HSQC

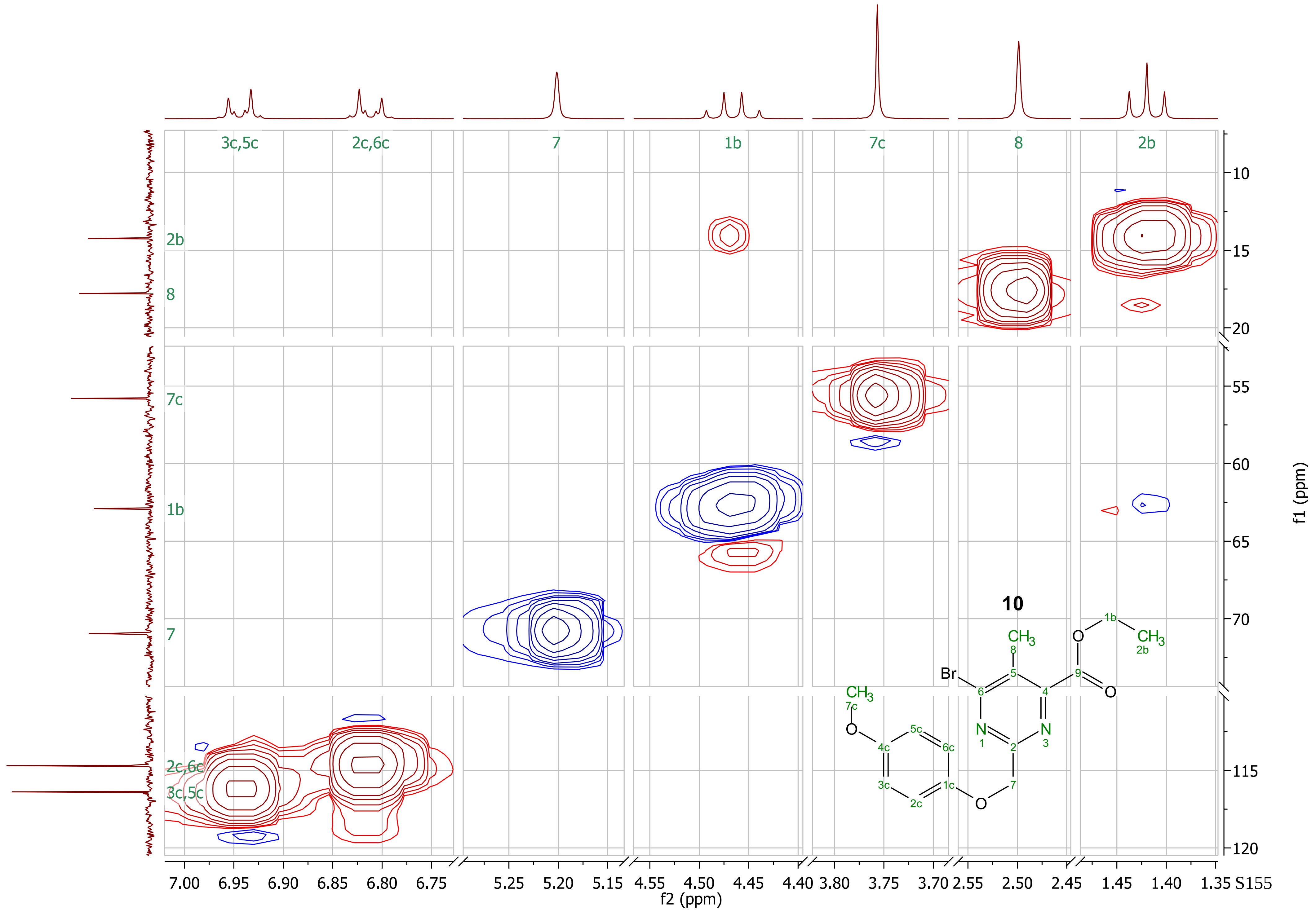

13C HMBC

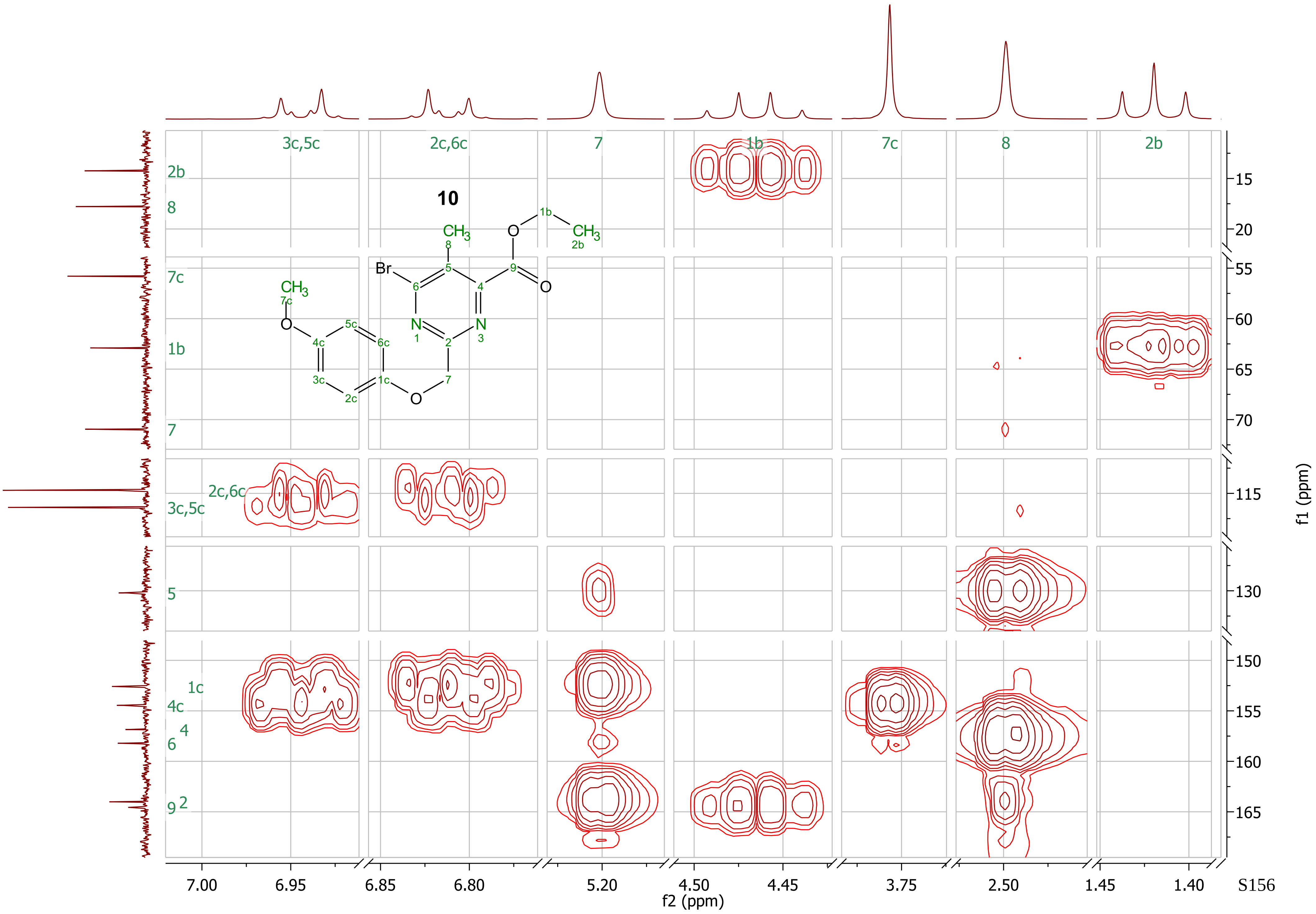
3c,5c
2c,6c
7
1b
7c
8
2b
10
CH3
8
O
1b
CH3
2b
Br
5
9
6
4
O
CH3
7c
O
5c
N
1
2
N
3
4c
6c
3c
1c
7
2c
O
2b
8
7c
1b
7
2c,6c
3c,5c
5
1c
4c
4
6
9
2
7.00
6.95
6.85
6.80
5.20
4.50
4.45
3.75
2.50
1.45
1.40
f2 (ppm)
15
20
55
60
65
70
115
130
150
155
160
165
f1 (ppm)

15N HMBC

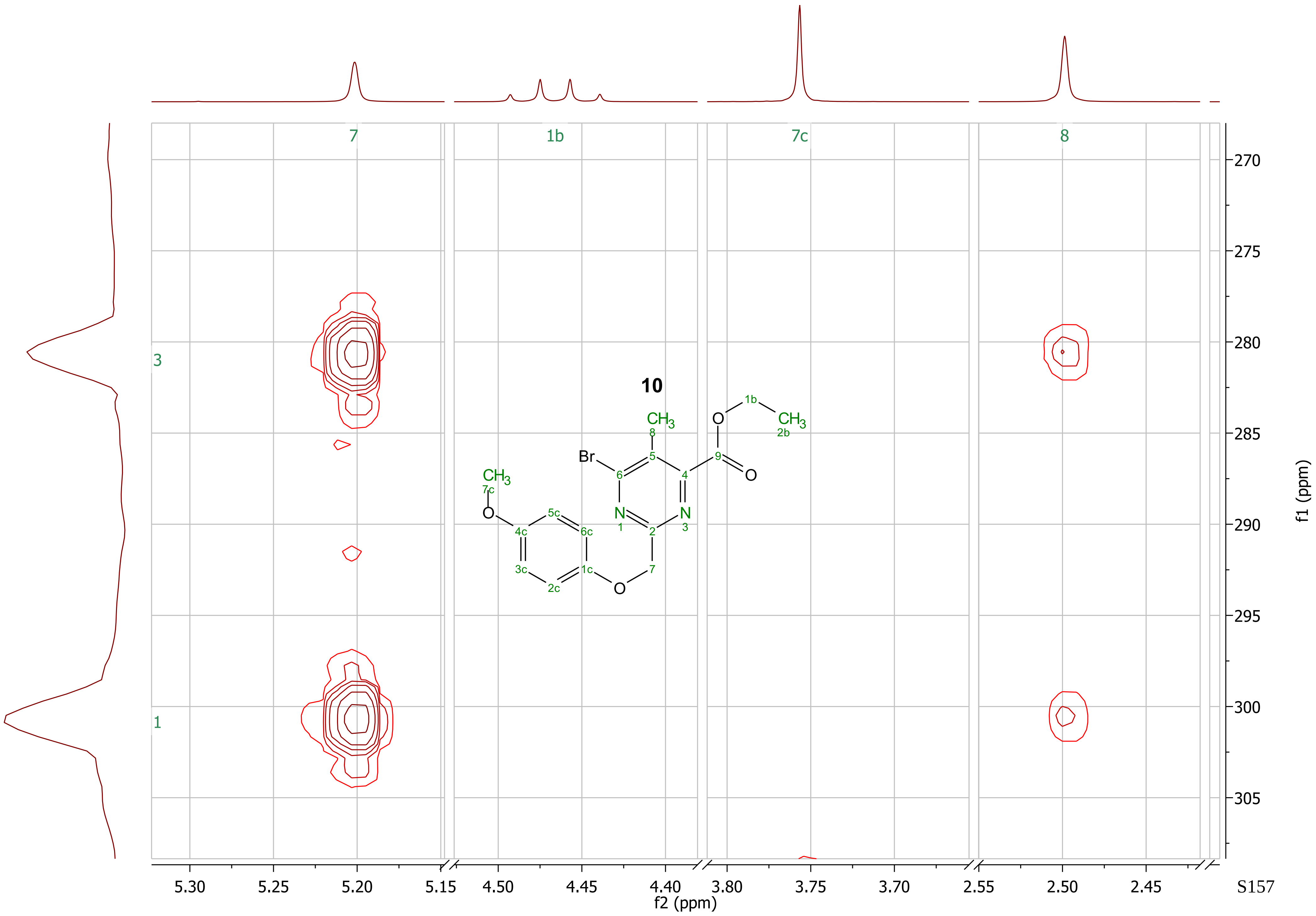

7
1b
7c
8
3
1
10
Br
f2 (ppm)
f1 (ppm)
5.30
5.25
5.20
5.15
4.50
4.45
4.40
3.80
3.75
3.70
2.55
2.50
2.45
270
275
280
285
290
295
300
305

15N HMBC f1 Projection ^{15}N NMR (41 MHz, $CDCl_3$) δ 300.81, 280.95.

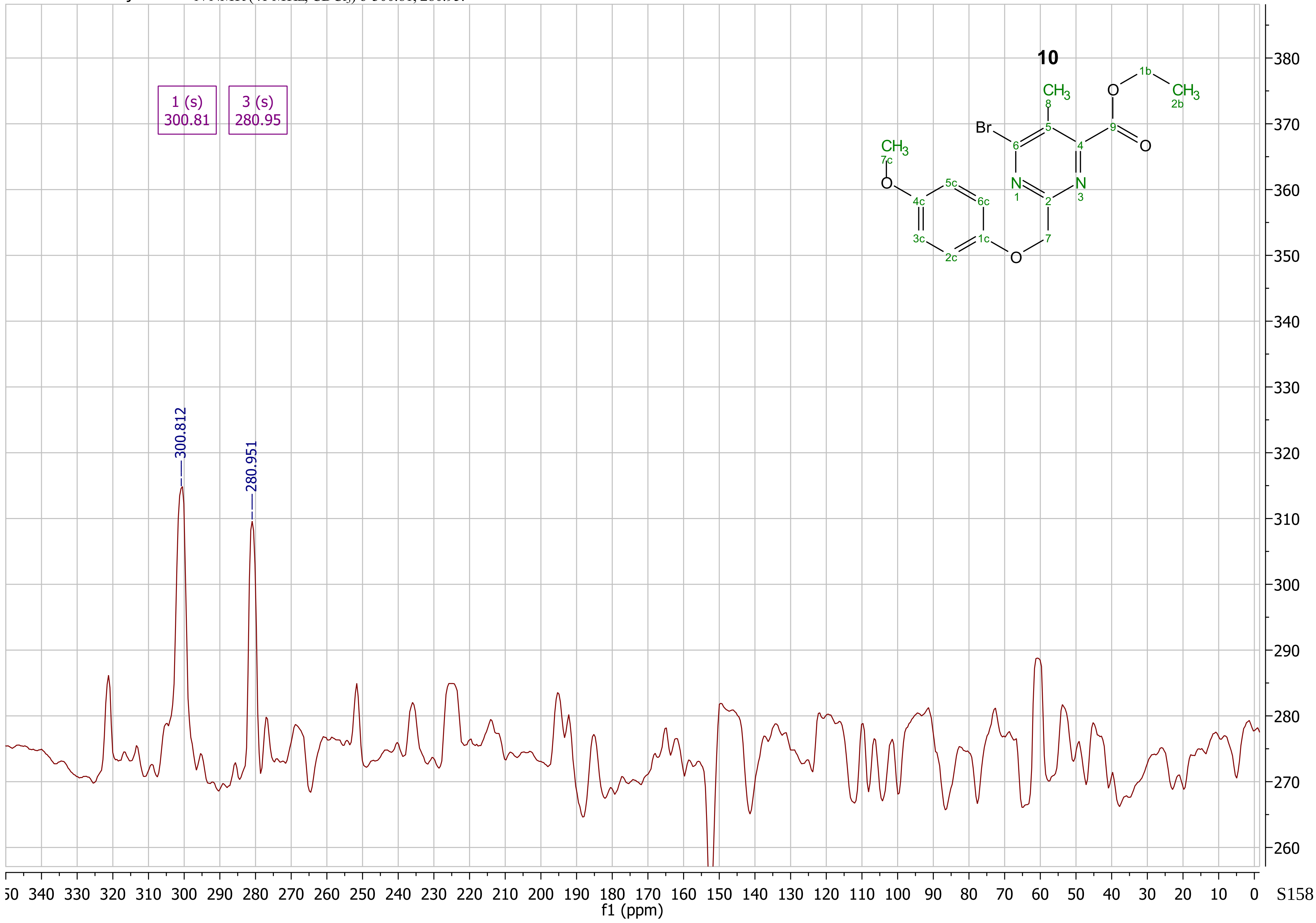

^{1}H NMR (400 MHz, $CDCl_3$) δ_{ppm} 6.98 – 6.86 (m, 2H), 6.86 – 6.75 (m, 2H), 5.14 (s, 2H), 4.37 (t, J = 6.9 Hz, 2H), 4.35 (t, J = 6.7 Hz, 2H), 3.75 (s, 3H), 2.27 (s, 3H), 1.78 (quint, J = 6.9 Hz, 2H), 1.71 (quint, J = 7.6, 6.8 Hz, 2H), 1.47 – 1.17 (m, 16H), 0.89 (app t, J = 6.9 Hz, 3H), 0.88 (app t, J = 6.8 Hz, 3H).

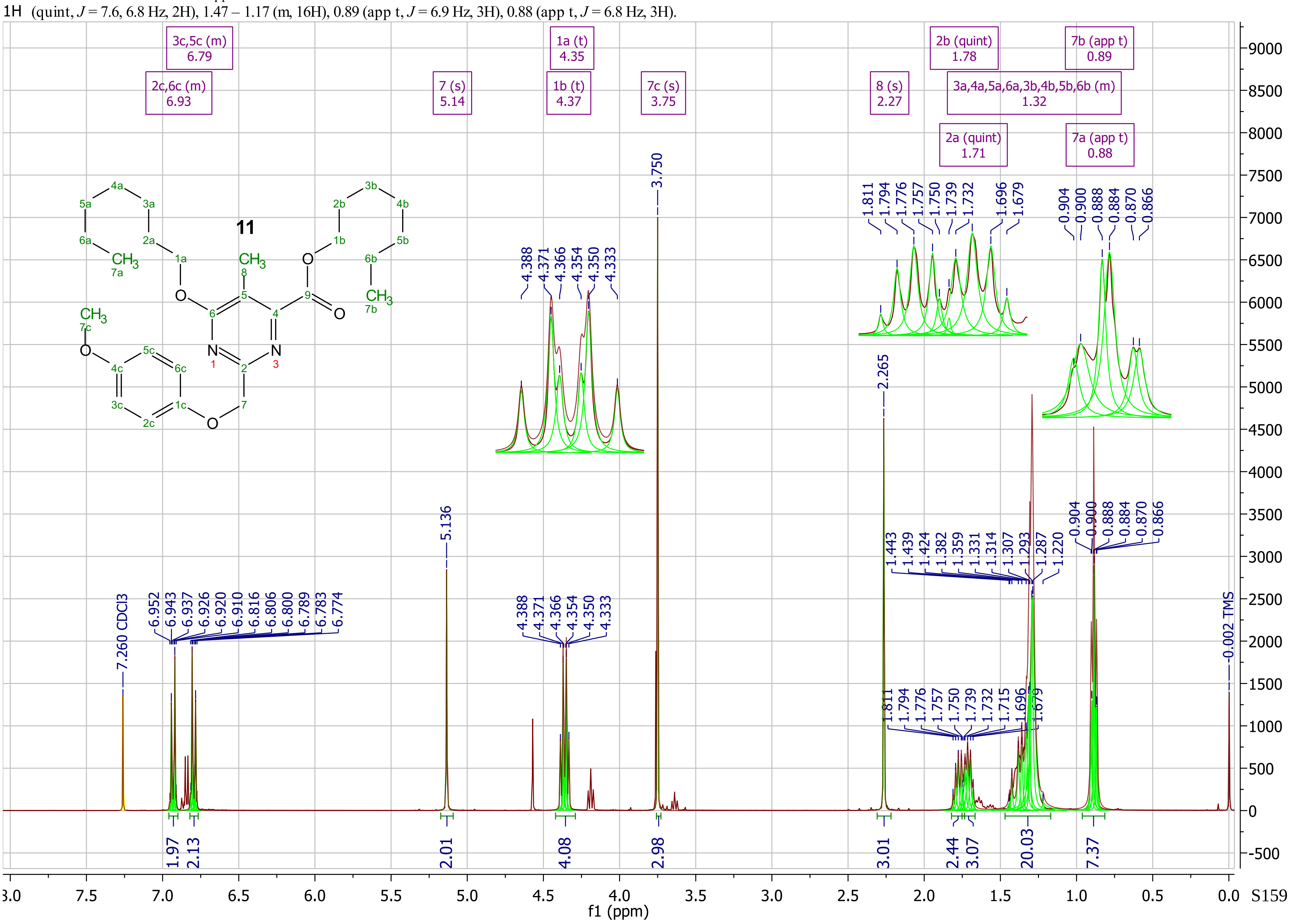

13C ^{13}C NMR (101 MHz, $CDCl_3$) δ 169.3, 165.8, 163.1, 155.0, 154.1, 152.9, 116.6, 116.1 (sym, 2C), 114.6 (sym, 2C), 71.2, 67.7, 66.4, 55.8, 31.9, 31.8, 29.1, 29.0, 28.74, 28.67, 26.04, 25.98, 22.73, 22.71, 14.22, 14.20, 11.1.

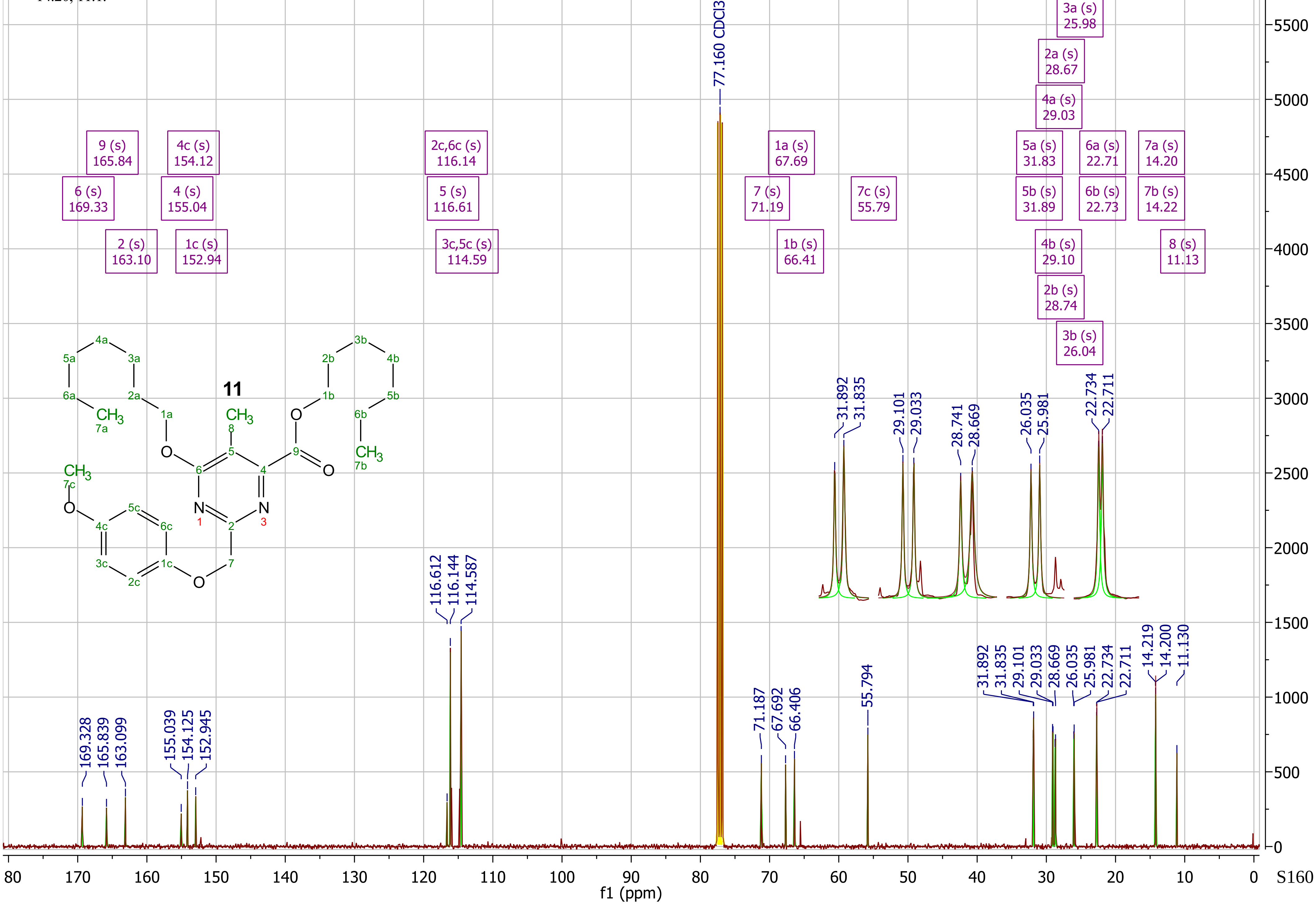

13C HSQC

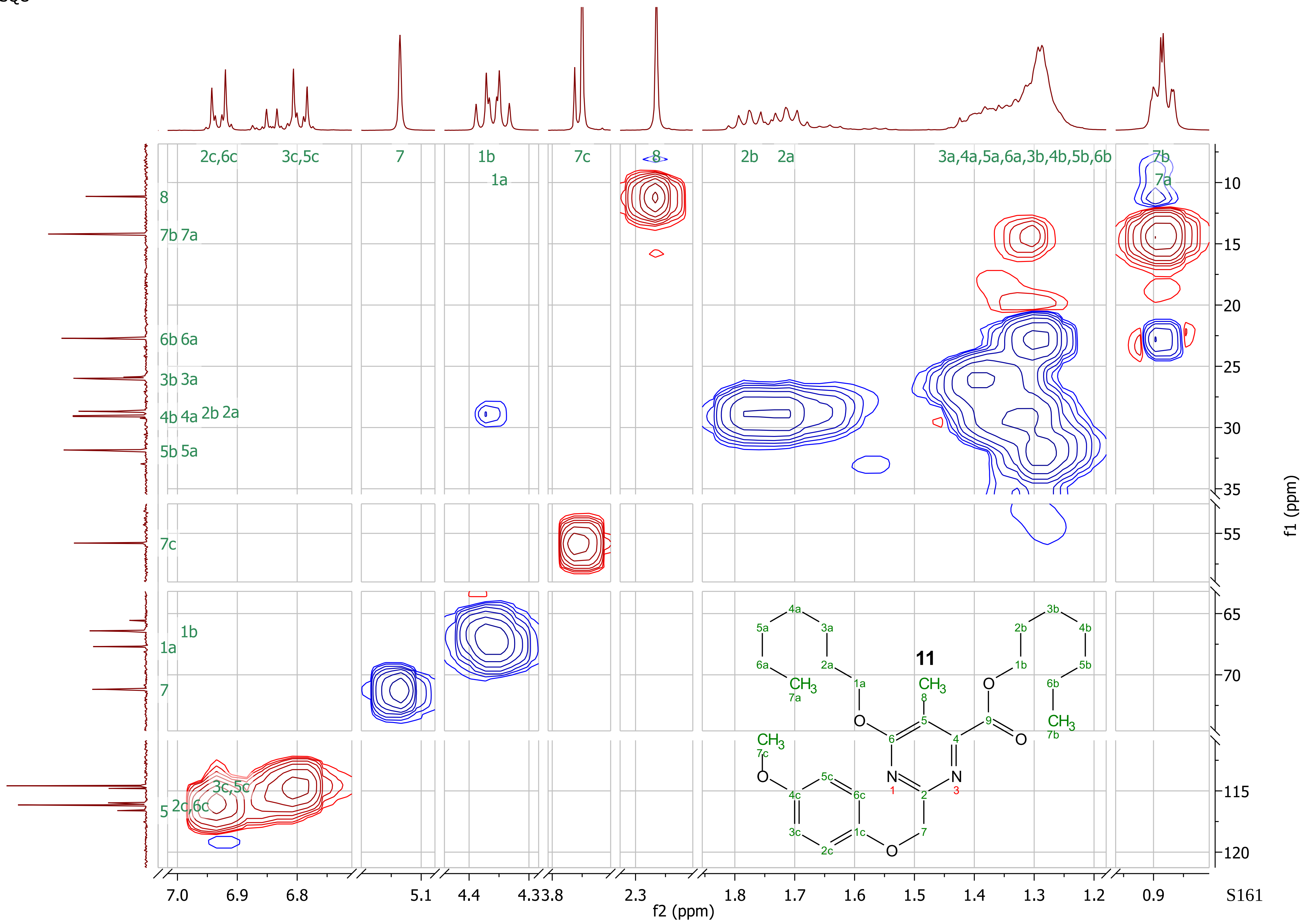
2c,6c
3c,5c
7
1b
1a
7c
8
2b
2a
3a,4a,5a,6a,3b,4b,5b,6b
7b
7a
7b 7a
6b 6a
3b 3a
4b 4a 2b 2a
5b 5a
7c
1b
1a
7
3c,5c
5 2c,6c
11
f2 (ppm)
f1 (ppm)

13C HMBC

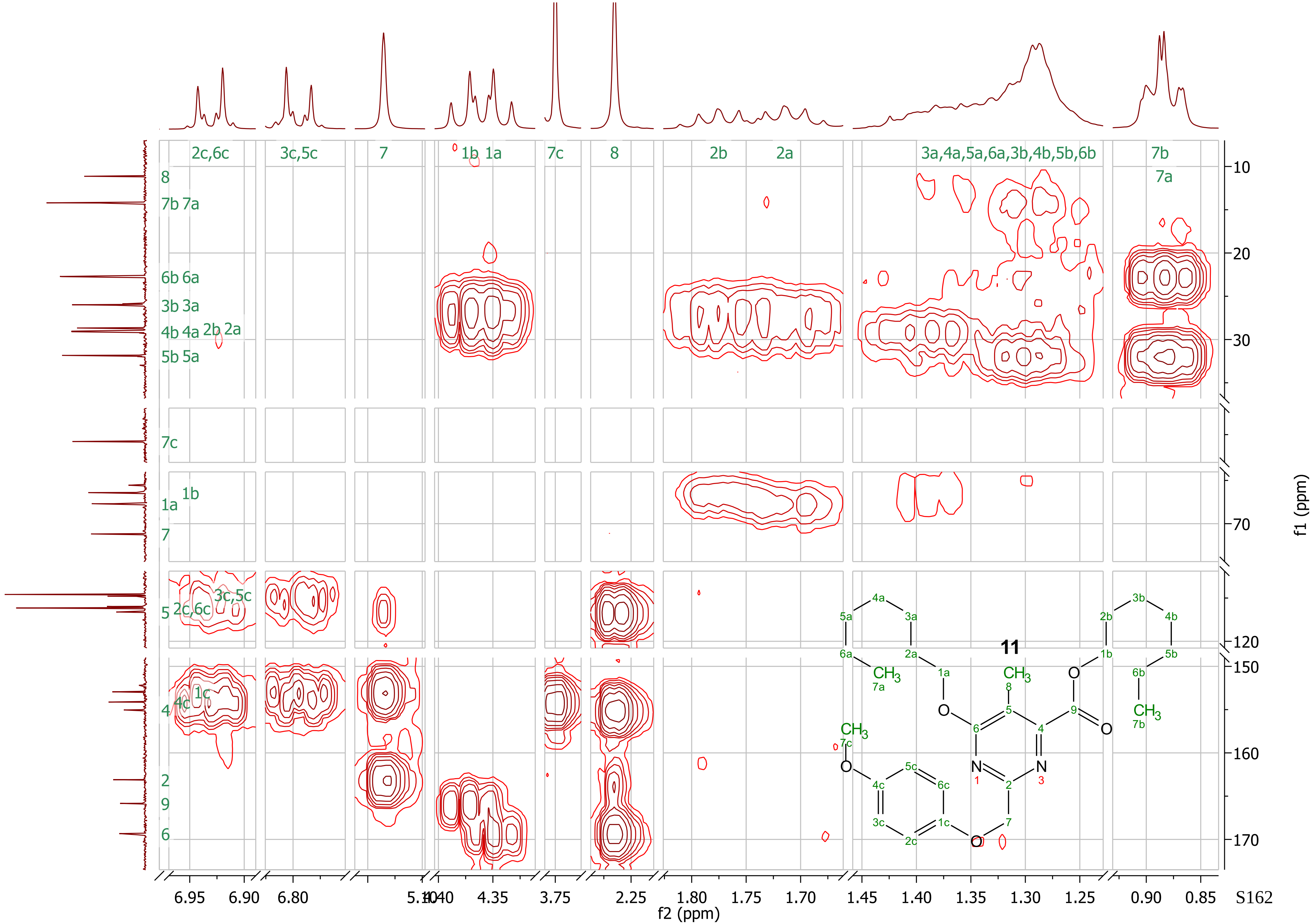

2c,6c
3c,5c
7
1b 1a
7c
8
2b
2a
3a,4a,5a,6a,3b,4b,5b,6b
7b
7a
8
7b 7a
6b 6a
3b 3a
4b 4a 2b 2a
5b 5a
7c
1a 1b
7
5 2c,6c 3c,5c
4 4c 1c
2
9
6
11
f2 (ppm)
f1 (ppm)

1H ^{1}H NMR (400 MHz, $CDCl_3$) δ 7.02 – 6.86 (m, 2H), 6.86 – 6.69 (m, 2H), 5.13 (s, 2H), 4.37 (t, *J* = 6.8 Hz, 2H), 4.35 (t, *J* = 6.6 Hz, 2H), 3.75 (s, 3H), 2.27 (s, 3H), 1.77 (quint, *J* = 7.0 Hz, 2H), 1.71 (quint, *J* = 7.1 Hz, 2H), 1.47 – 1.33 (m, 4H), 1.35 – 1.19 (m, 16H), 0.88 (app t, *J* = 6.7 Hz, 3H), 0.88 (app t, *J* = 6.9 Hz, 3H).

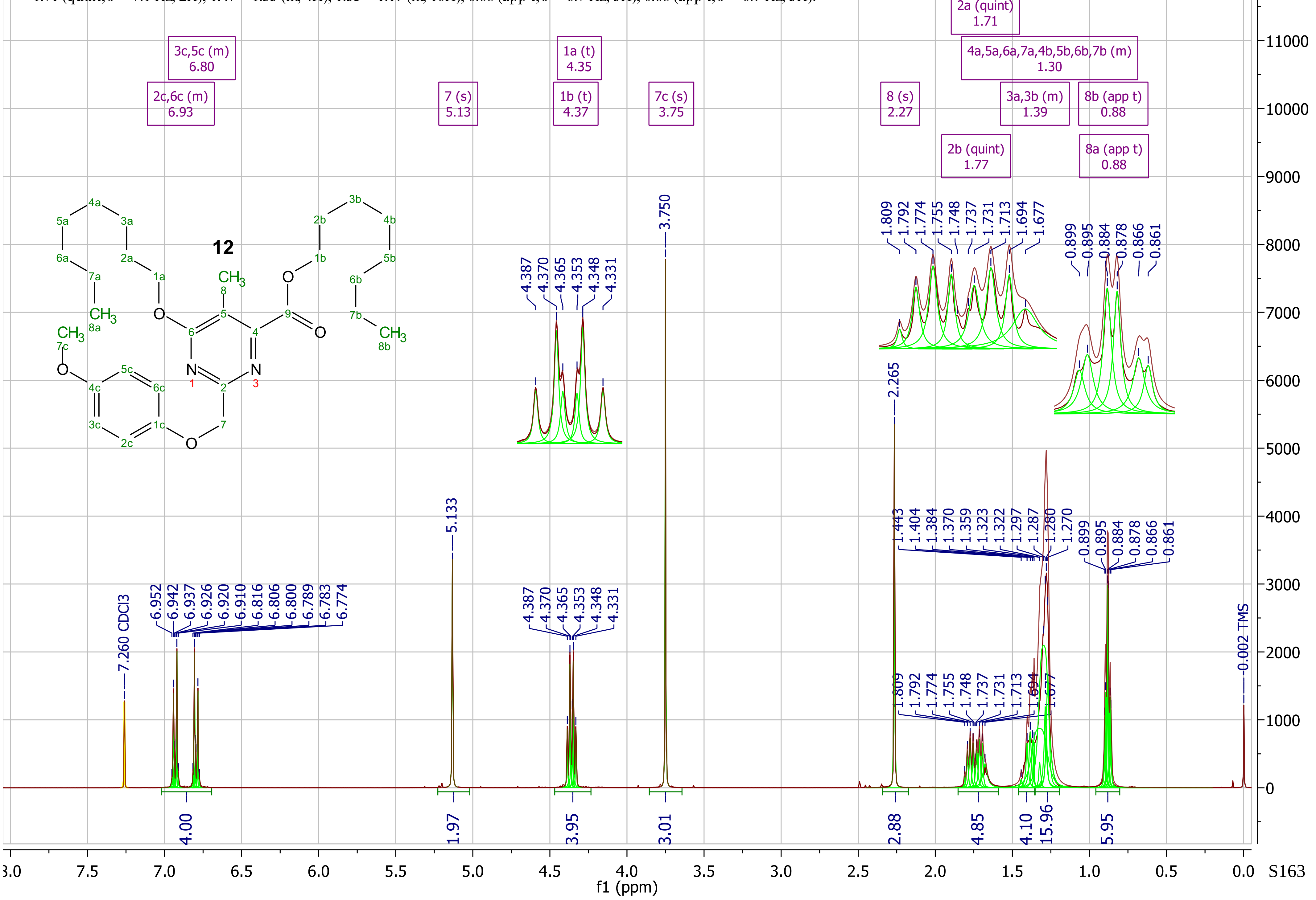

13C ^{13}C NMR (101 MHz, $CDCl_3$) δ 169.3, 165.9, 163.1, 155.1, 154.1, 152.9, 116.6, 116.1 (sym, 2C), 114.6 (sym, 2C), 71.2, 67.7, 66.4, 55.8, 31.94, 31.91, 29.39, 29.35, 29.33, 29.30, 28.74, 28.66, 26.1, 26.0, 22.79, 22.77, 14.24, 14.23, 11.1.

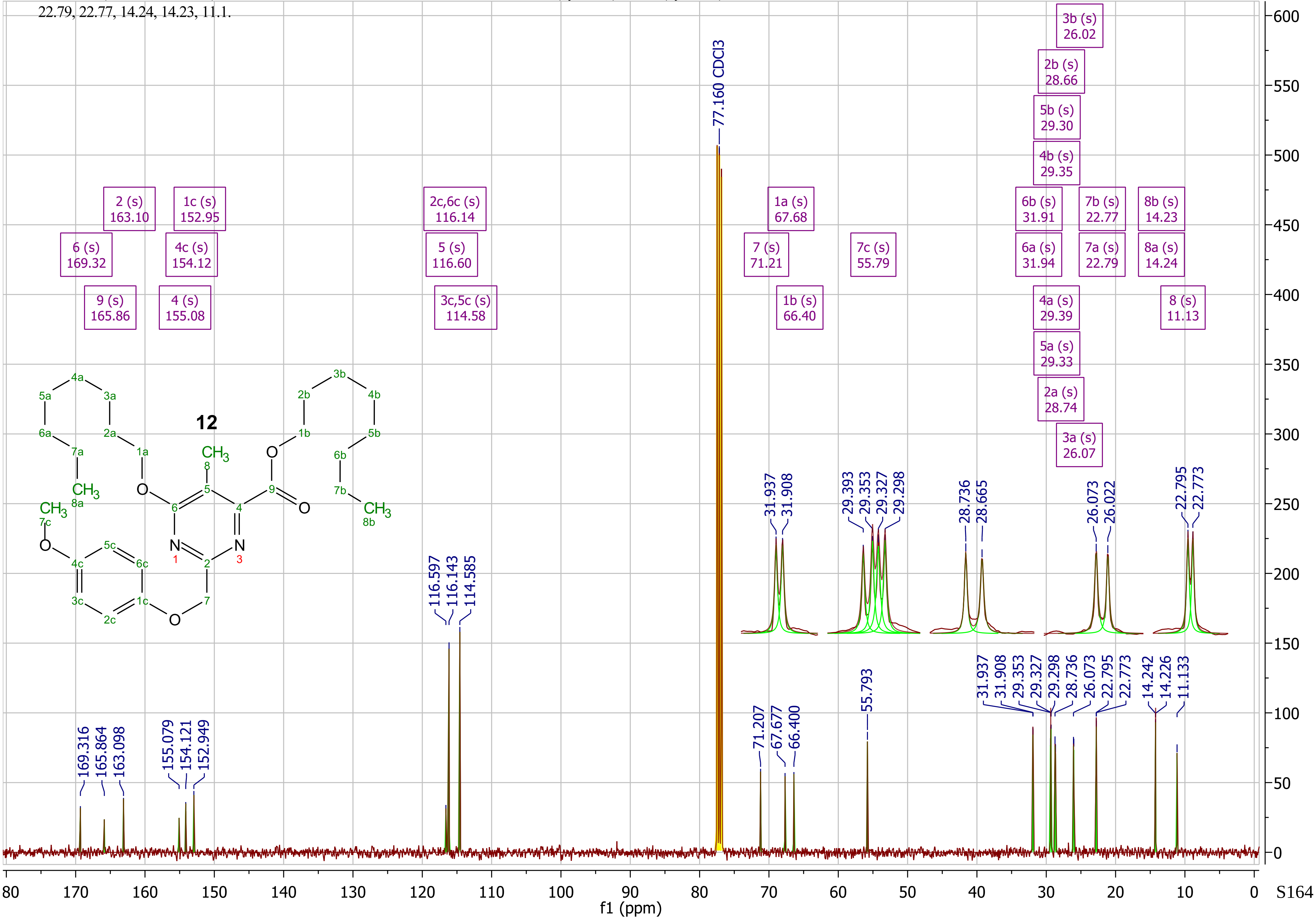

13C HSQC

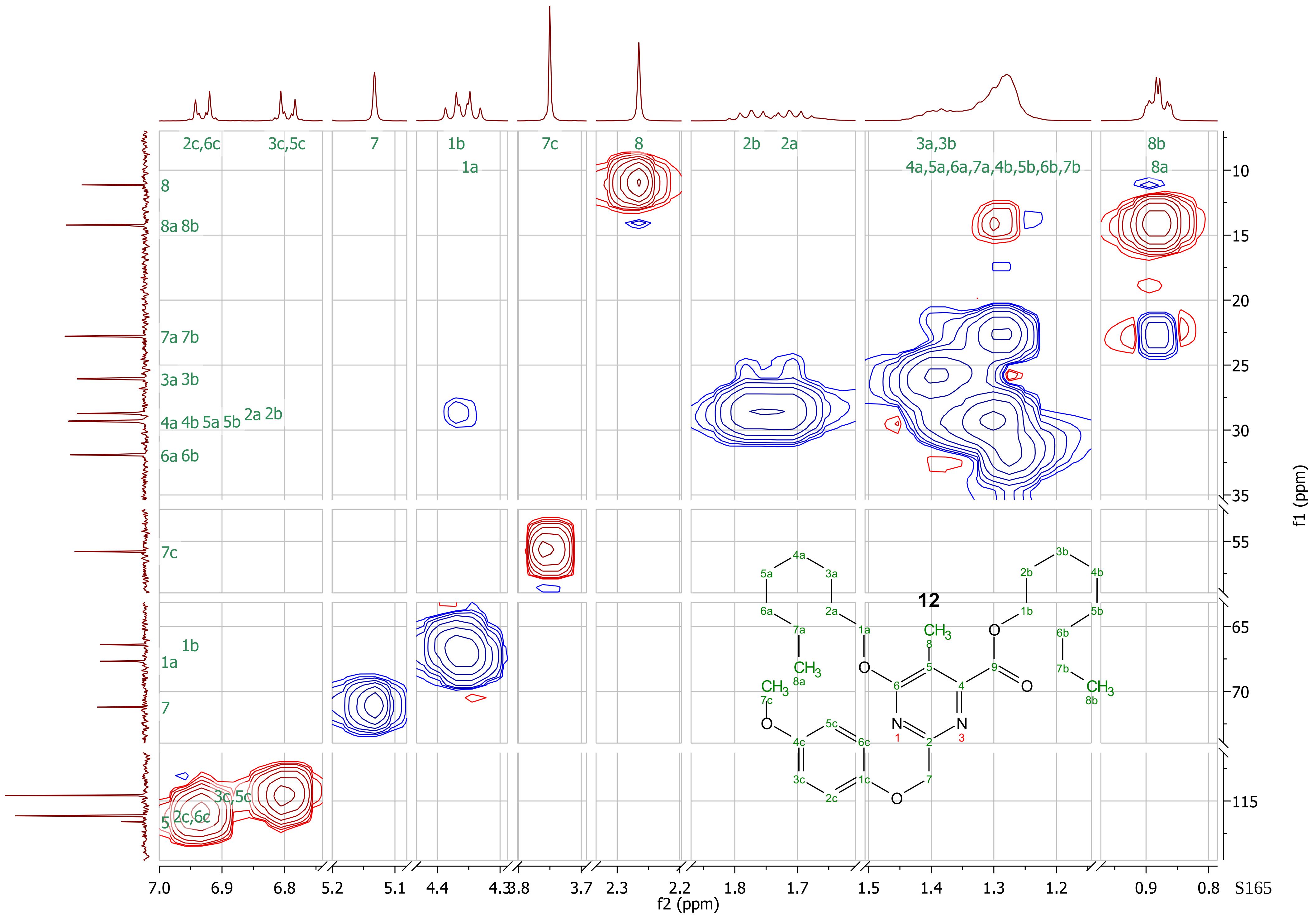
2c,6c
3c,5c
7
1b
1a
7c
8
2b
2a
3a,3b
4a,5a,6a,7a,4b,5b,6b,7b
8b
8a
8a 8b
7a 7b
3a 3b
4a 4b 5a 5b
6a 6b
1b
1a
12
f2 (ppm)
f1 (ppm)

13C HMBC

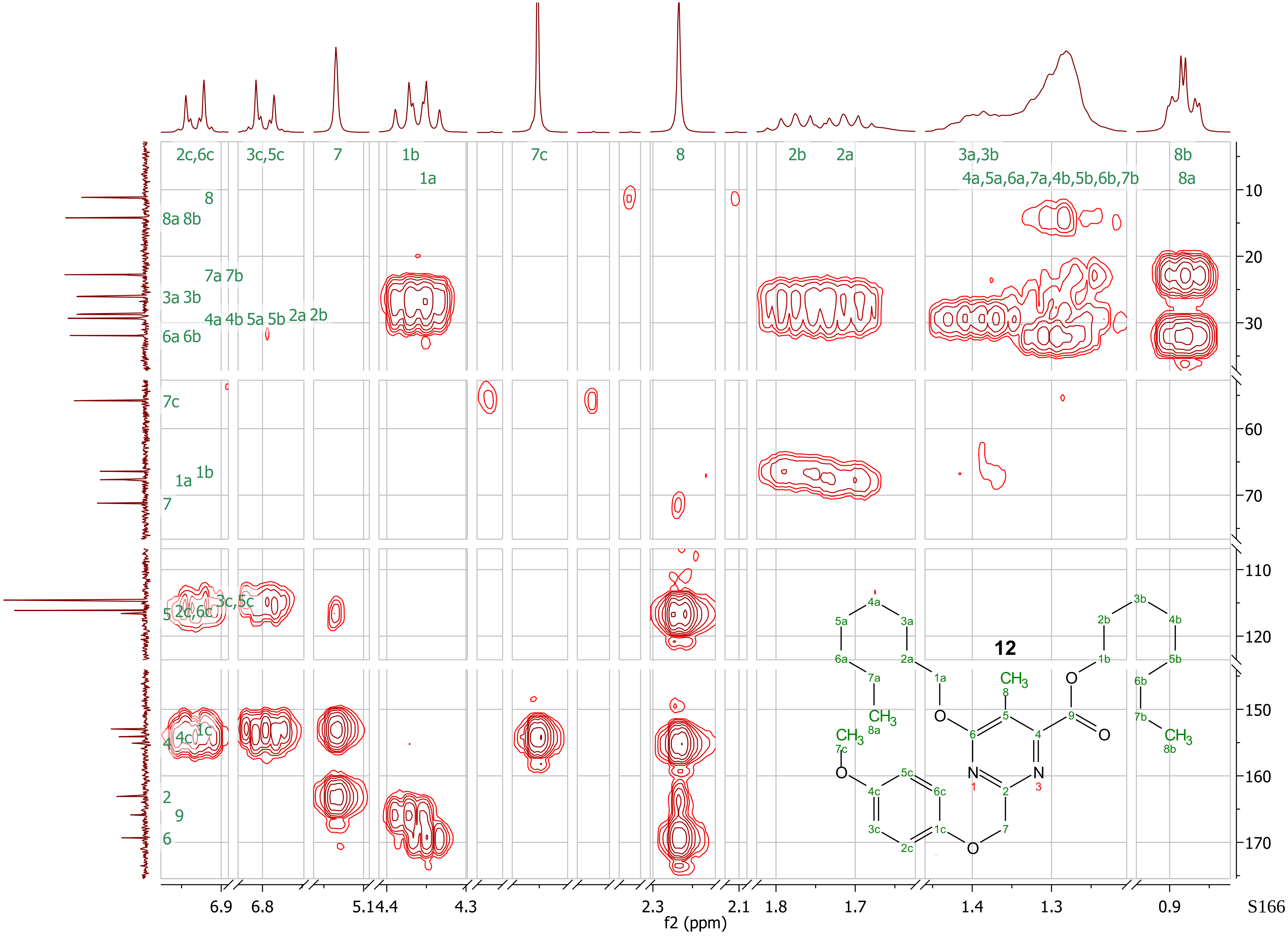

2c,6c
3c,5c
7
1b
1a
7c
8
2b
2a
3a,3b
4a,5a,6a,7a,4b,5b,6b,7b
8b
8a
8a 8b
7a 7b
3a 3b
4a 4b 5a 5b 2a 2b
6a 6b
7c
1a 1b
7
5 2c,6c
3c,5c
4 4c 1c
2
9
6
12
f2 (ppm)
f1 (ppm)

^{1}H NMR (400 MHz, $CDCl_3$) δ 6.98 – 6.84 (m, 2H), 6.84 – 6.69 (m, 2H), 5.19 (quint, J = 5.9 Hz, 1H), 5.13 (s, 2H), 5.12 (app quint, J = 6.0 Hz, 1H), 3.74 (s, 3H), 2.22 (s, 3H), 1.79 – 1.63 (m, 4H), 1.67 – 1.51 (m, 4H), 1.47 – 1.29 (m, 4H), 1.32 – 1.13 (m, 4H), 0.98 (t, J = 7.4 Hz, 3H), 0.91 (app t, J = 7.0 Hz, 3H), 0.85 (app t, J = 6.8 Hz, 3H), 0.84 (t, J = 7.4 Hz, 3H).

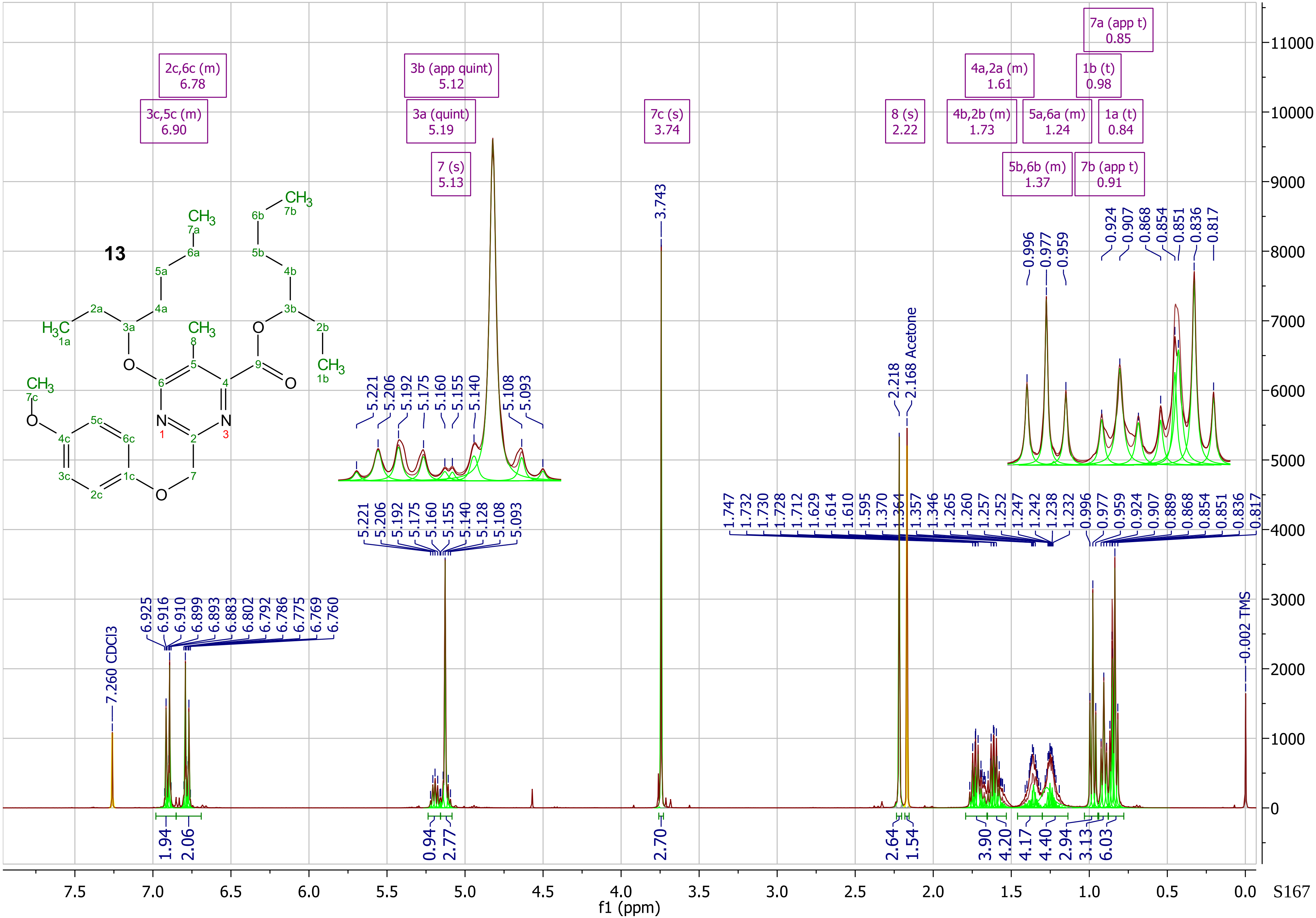

13C ^{13}C NMR (101 MHz, $CDCl_3$) δ 169.2, 166.1, 163.1, 156.2, 154.0, 153.0, 116.0 (sym, 2C), 115.5, 114.6 (sym, 2C), 78.2, 78.1, 70.9, 55.8, 33.3, 33.0, 27.6, 27.4, 27.0, 26.6, 22.74, 22.72, 14.1 (2C), 11.1, 9.8, 9.5.

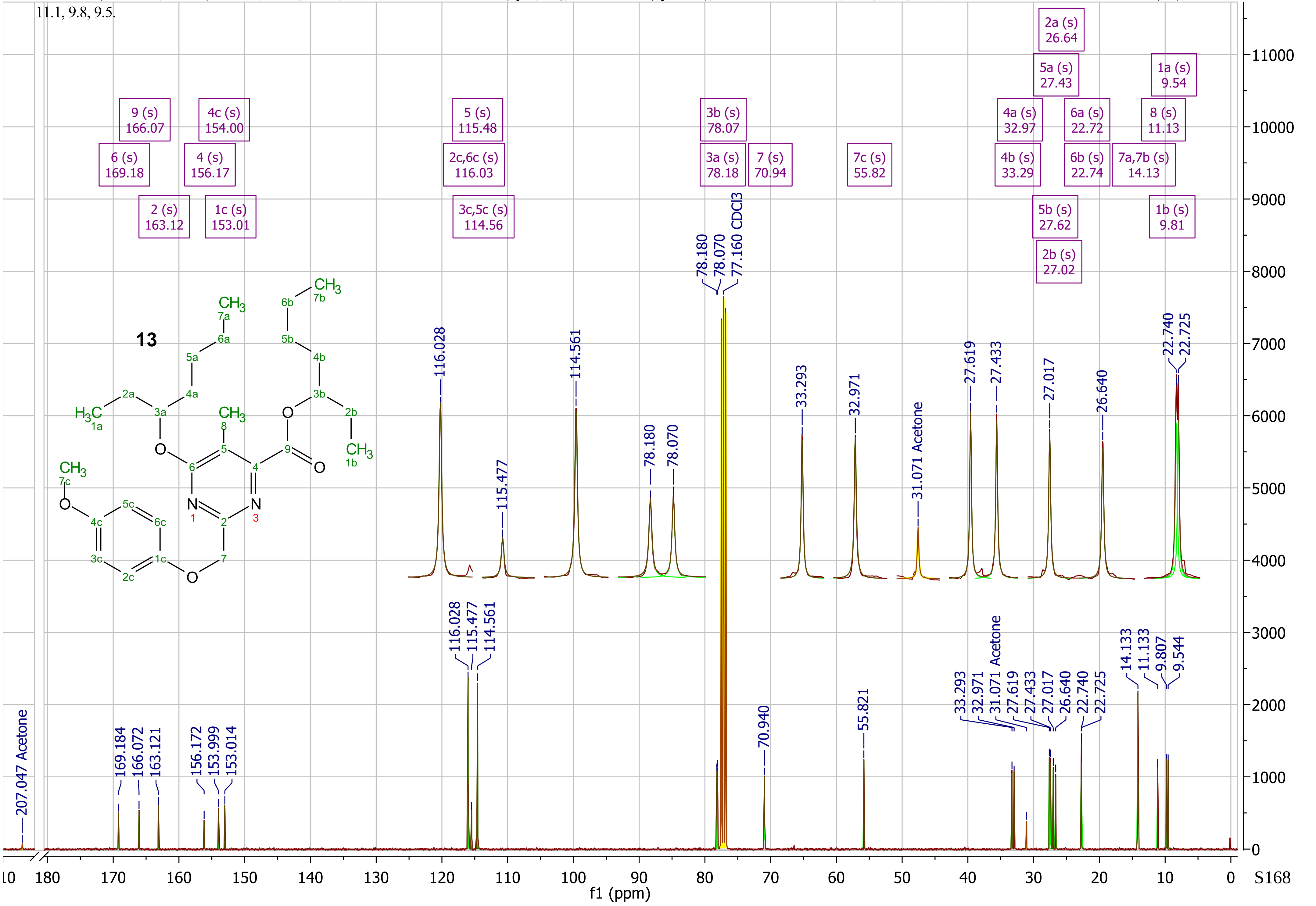

13C HSQC

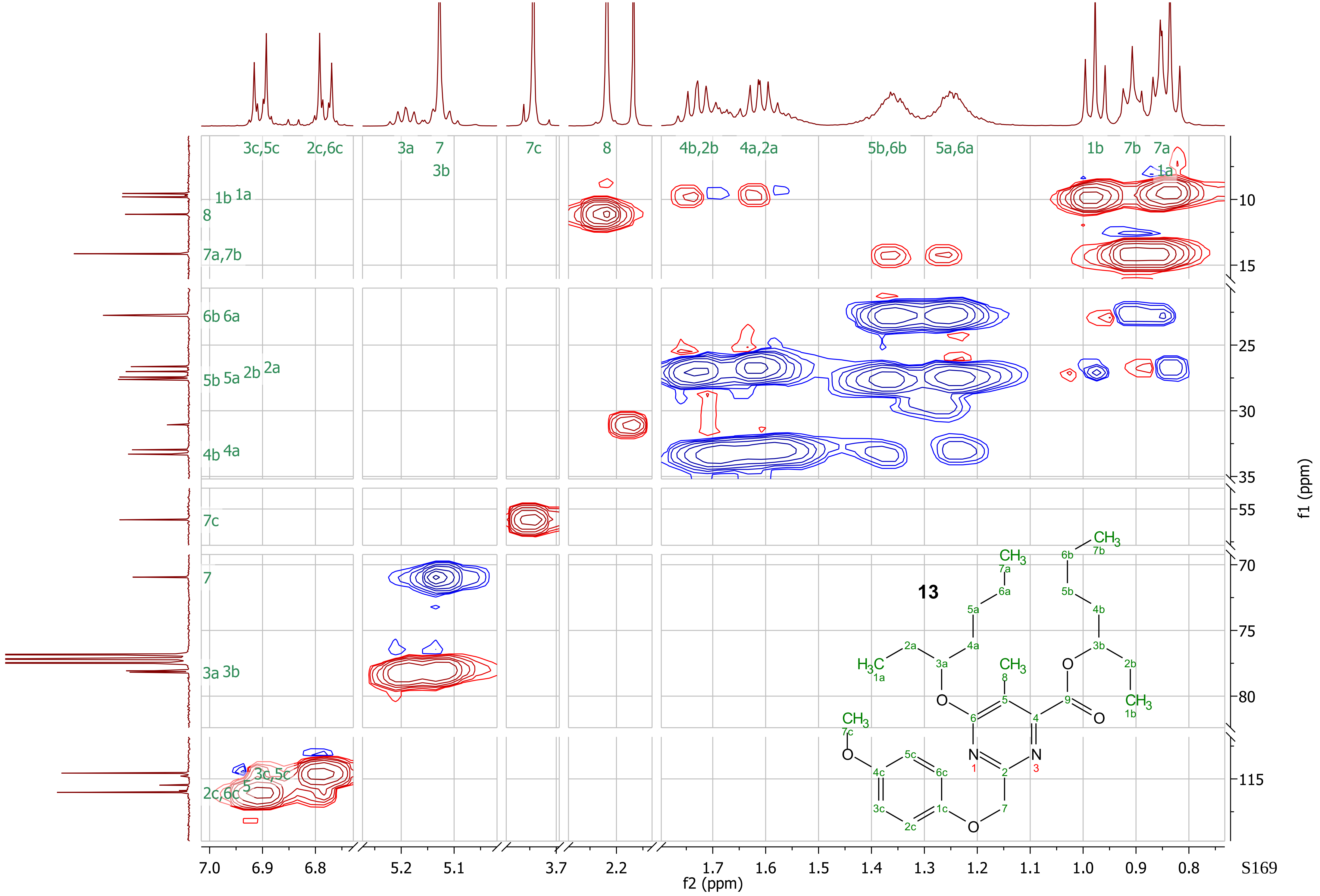
3c,5c
2c,6c
3a
7
3b
7c
8
4b,2b
4a,2a
5b,6b
5a,6a
1b
7b
7a
1a
1b 1a
8
7a,7b
6b 6a
5b 5a 2b 2a
4b 4a
7c
7
3a 3b
2c,6c 3c,5c
13
f1 (ppm)
f2 (ppm)

13C HMBC

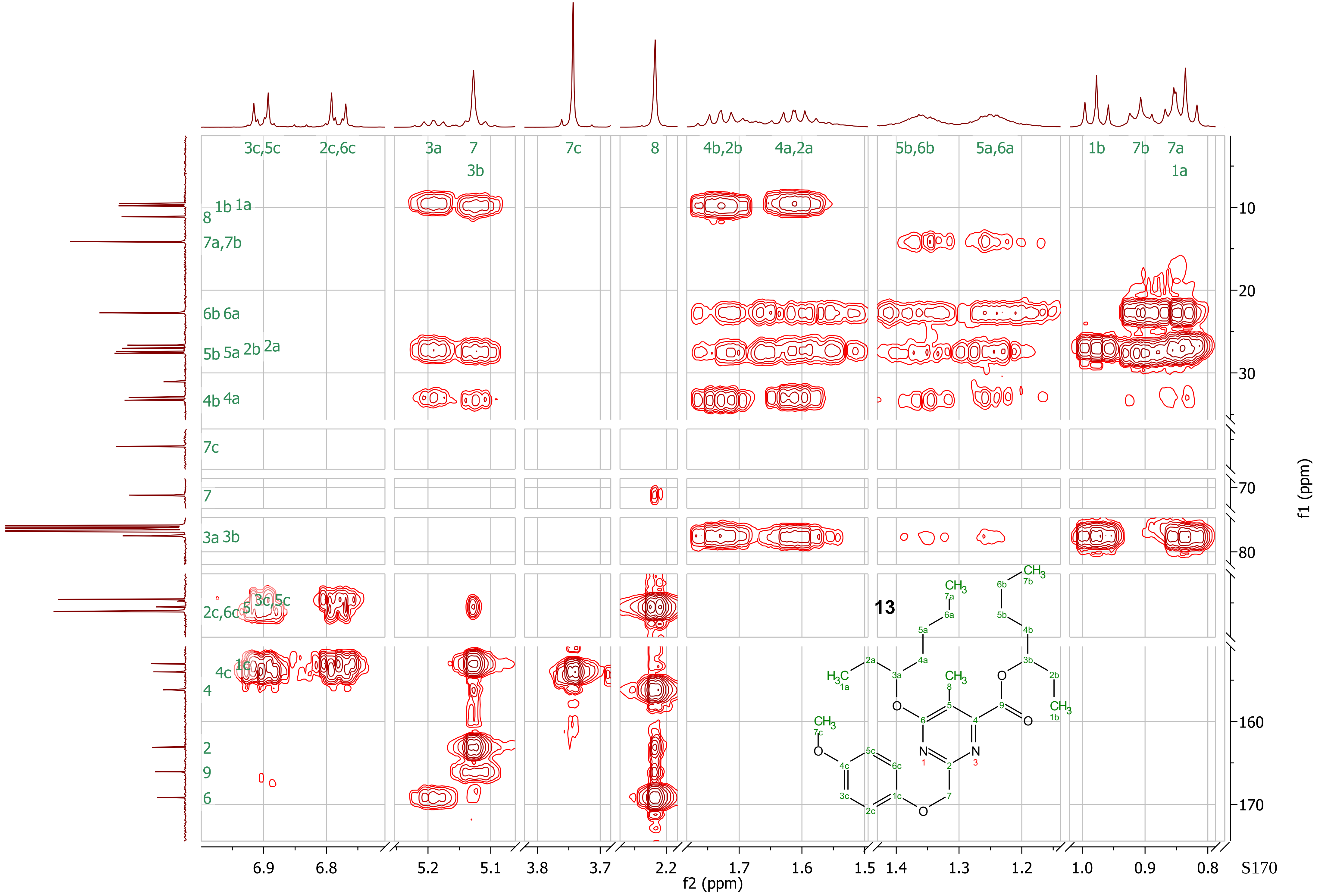
3c,5c
2c,6c
3a
7
3b
7c
8
4b,2b
4a,2a
5b,6b
5a,6a
1b
7b
7a
1a
8
1b 1a
7a,7b
6b 6a
5b 5a 2b 2a
4b 4a
7c
7
3a 3b
2c,6c
4c
4
2
9
6
13
f2 (ppm)
f1 (ppm)

1H ^{1}H NMR (400 MHz, $CDCl_3$) δ 9.25 (br s, 1H), 7.02 – 6.85 (m, 2H), 6.85 – 6.68 (m, 2H), 5.12 (s, 2H), 4.40 (t, J = 6.6 Hz, 2H), 3.76 (s, 3H), 2.57 (s, 3H), 1.77 (quint, J = 6.8 Hz, 2H), 1.53 – 1.20 (m, 6H), 0.90 (app t, J = 6.8 Hz, 3H).

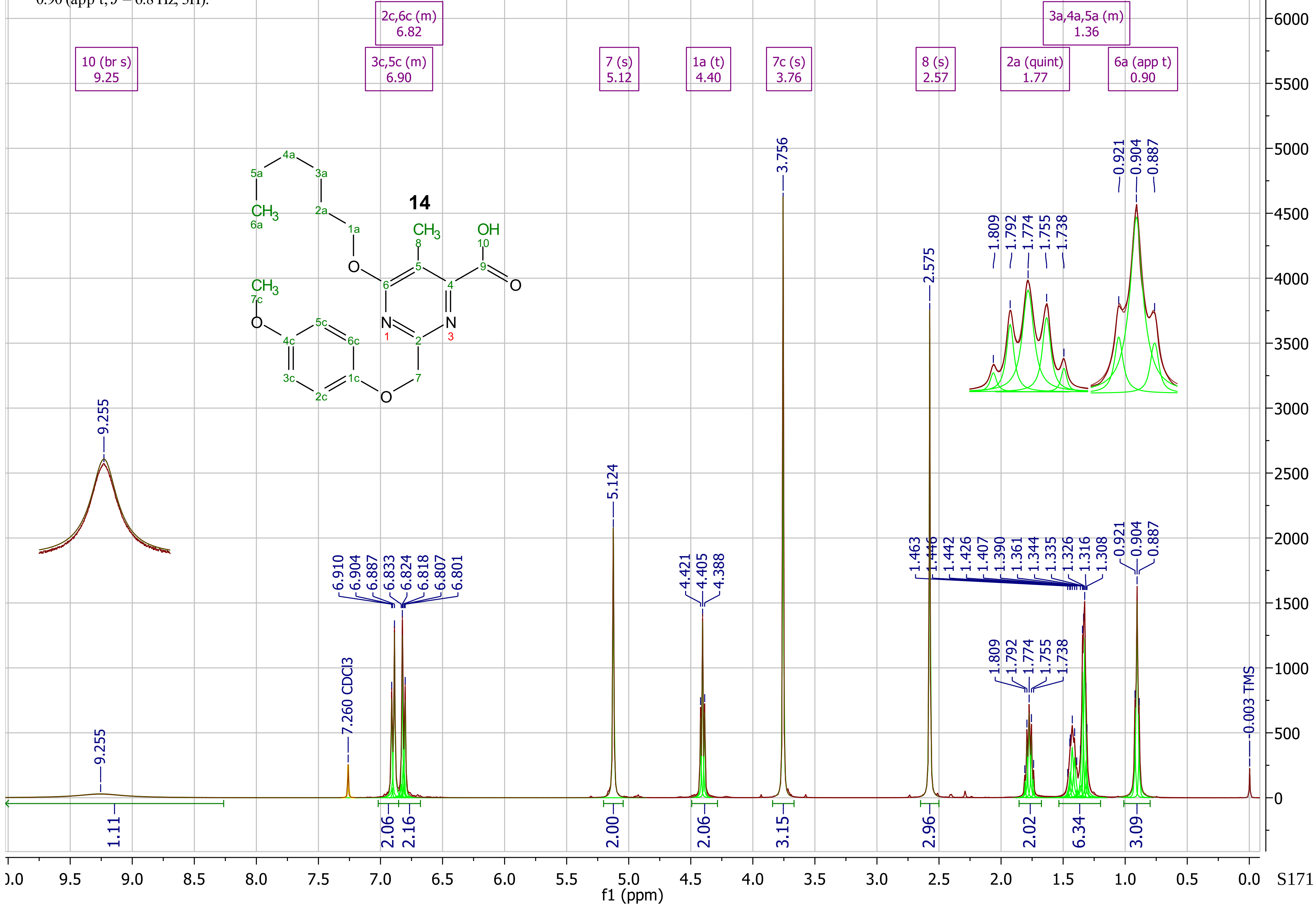

13C ^{13}C NMR (101 MHz, $CDCl_3$) δ 170.6, 163.6, 161.7, 154.4, 152.5, 148.4, 120.8, 115.9 (sym, 2C), 114.8 (sym, 2C), 70.3, 68.5, 55.8, 31.6, 28.6, 25.7, 22.7, 14.1, 10.7.

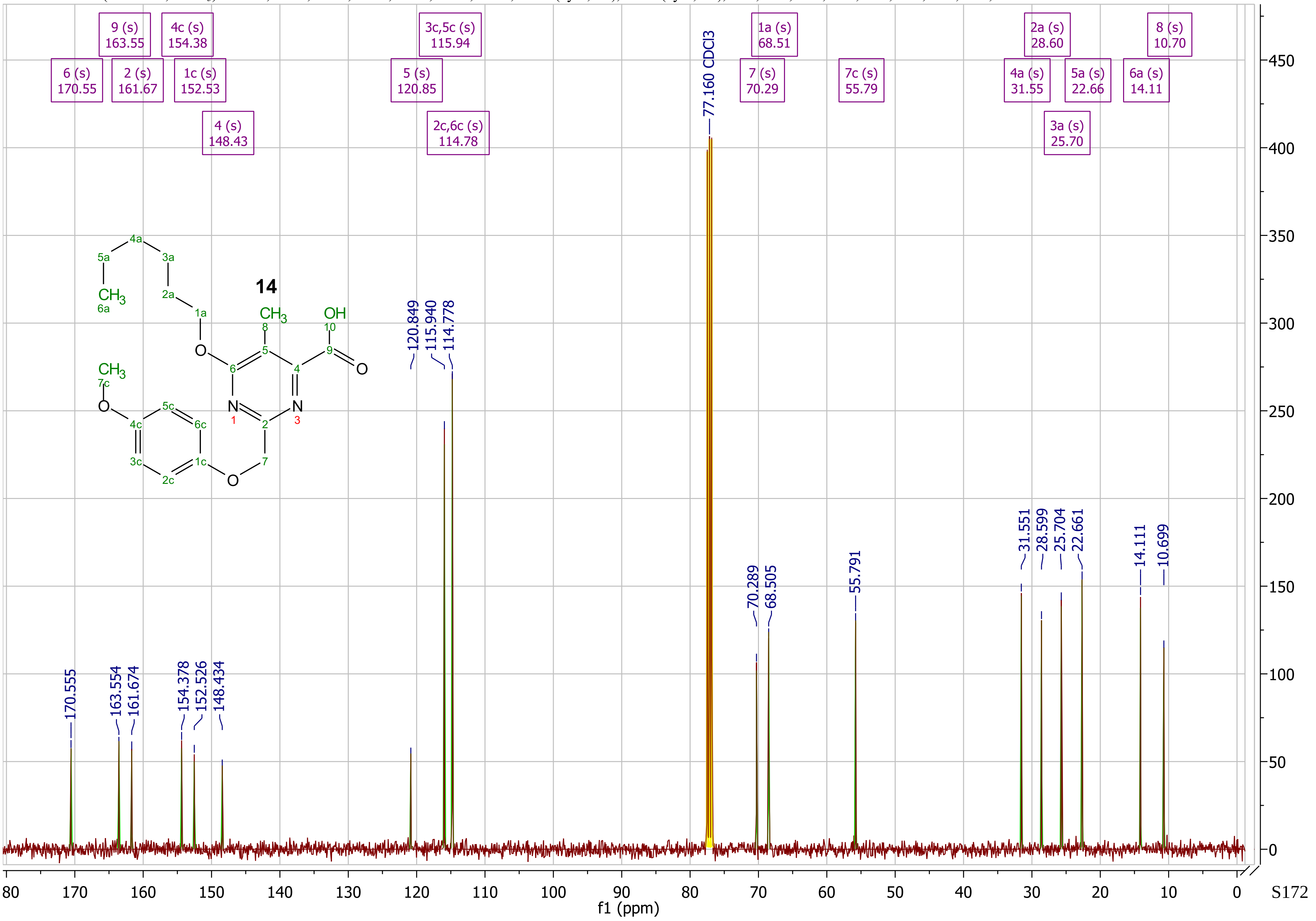

13C HSQC

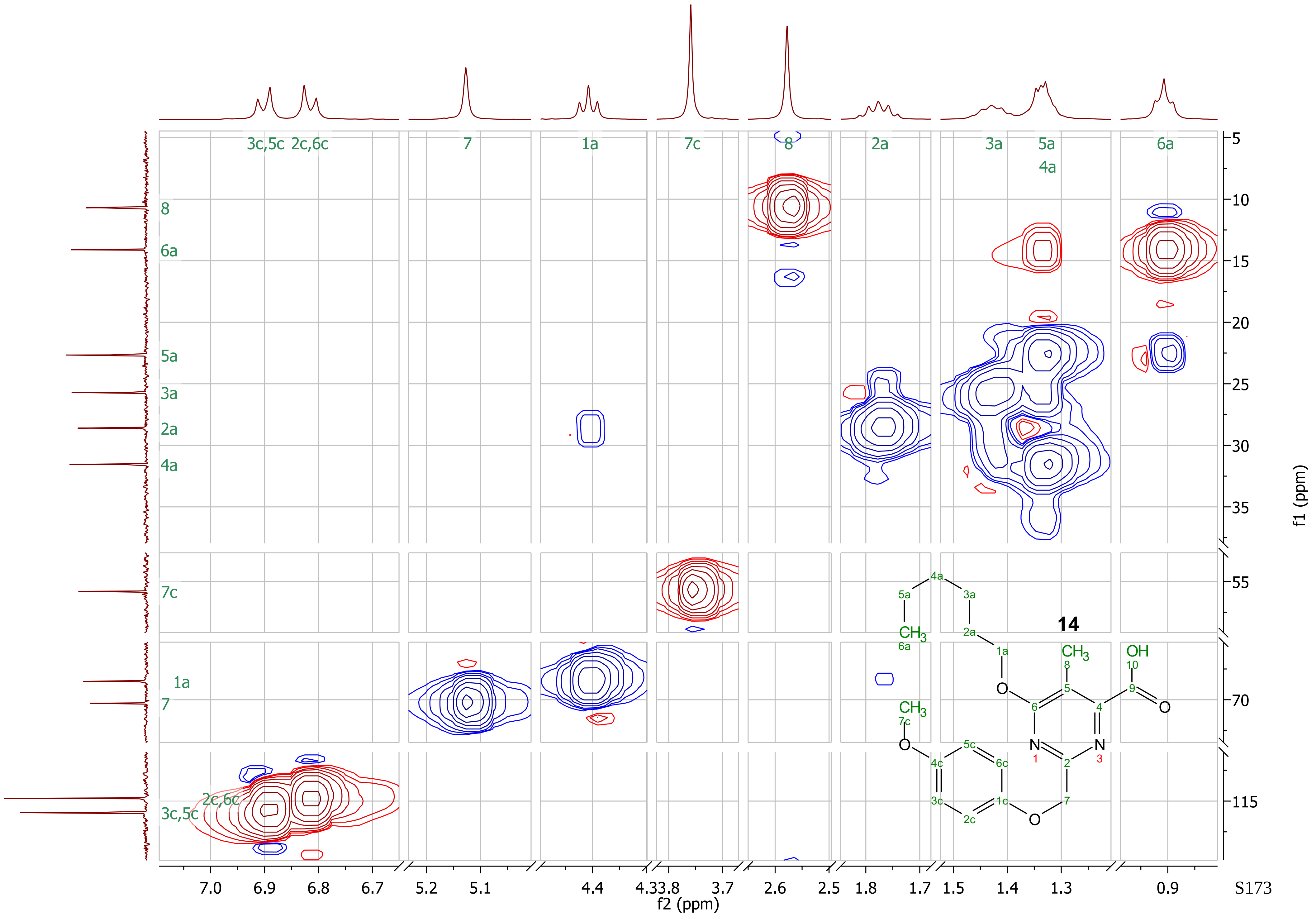

13C HMBC

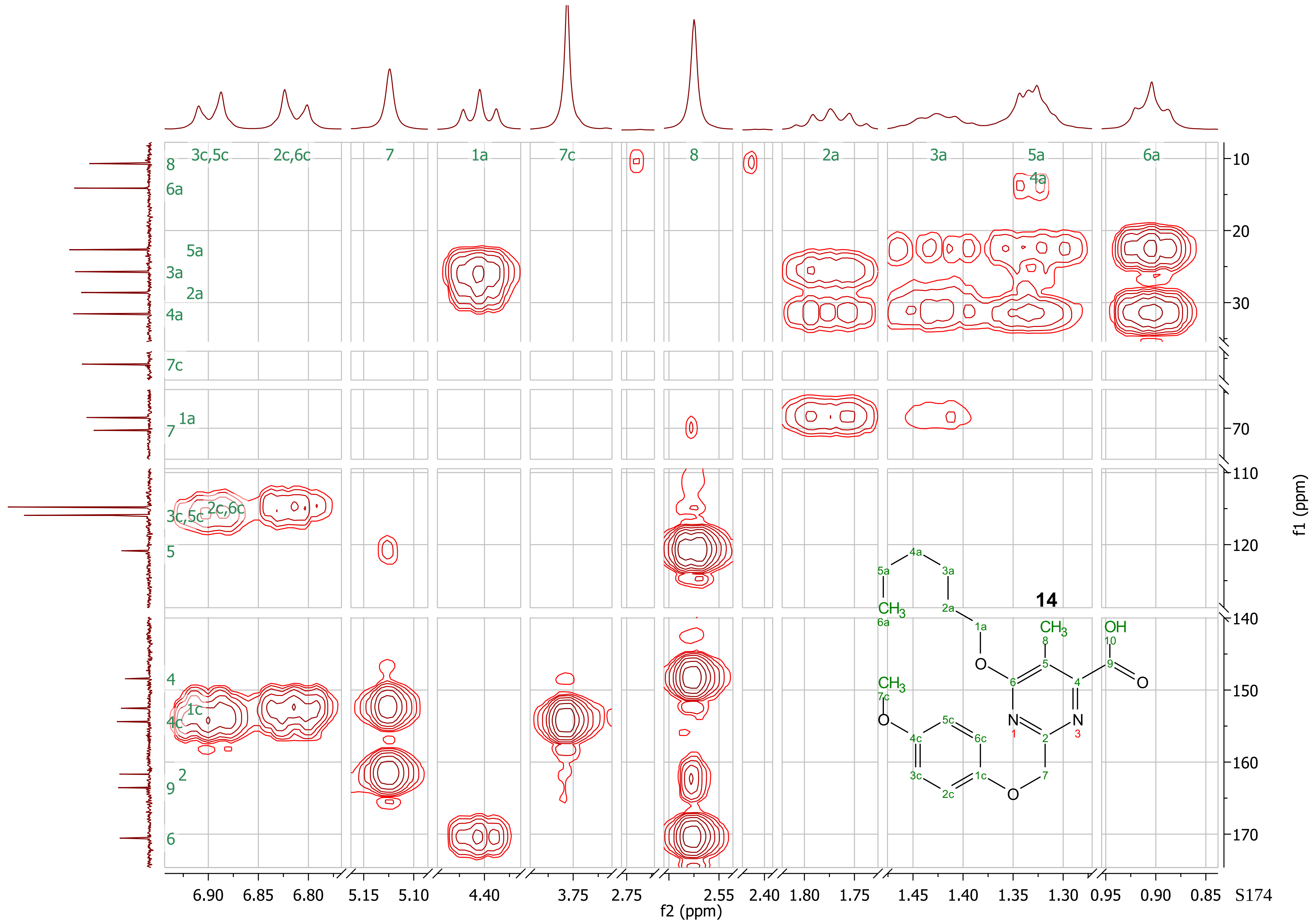

S174

1H ^{1}H NMR (400 MHz, $CDCl_3$) δ 8.90 (br s, 1H), 6.94 – 6.86 (m, 2H), 6.85 – 6.77 (m, 2H), 5.12 (s, 2H), 4.30 (d, *J* = 5.6 Hz, 2H), 3.76 (s, 3H), 2.57 (s, 3H), 1.91 – 1.75 (m, 1H), 1.46 – 1.28 (m, 8H), 0.96 – 0.86 (m, 6H).

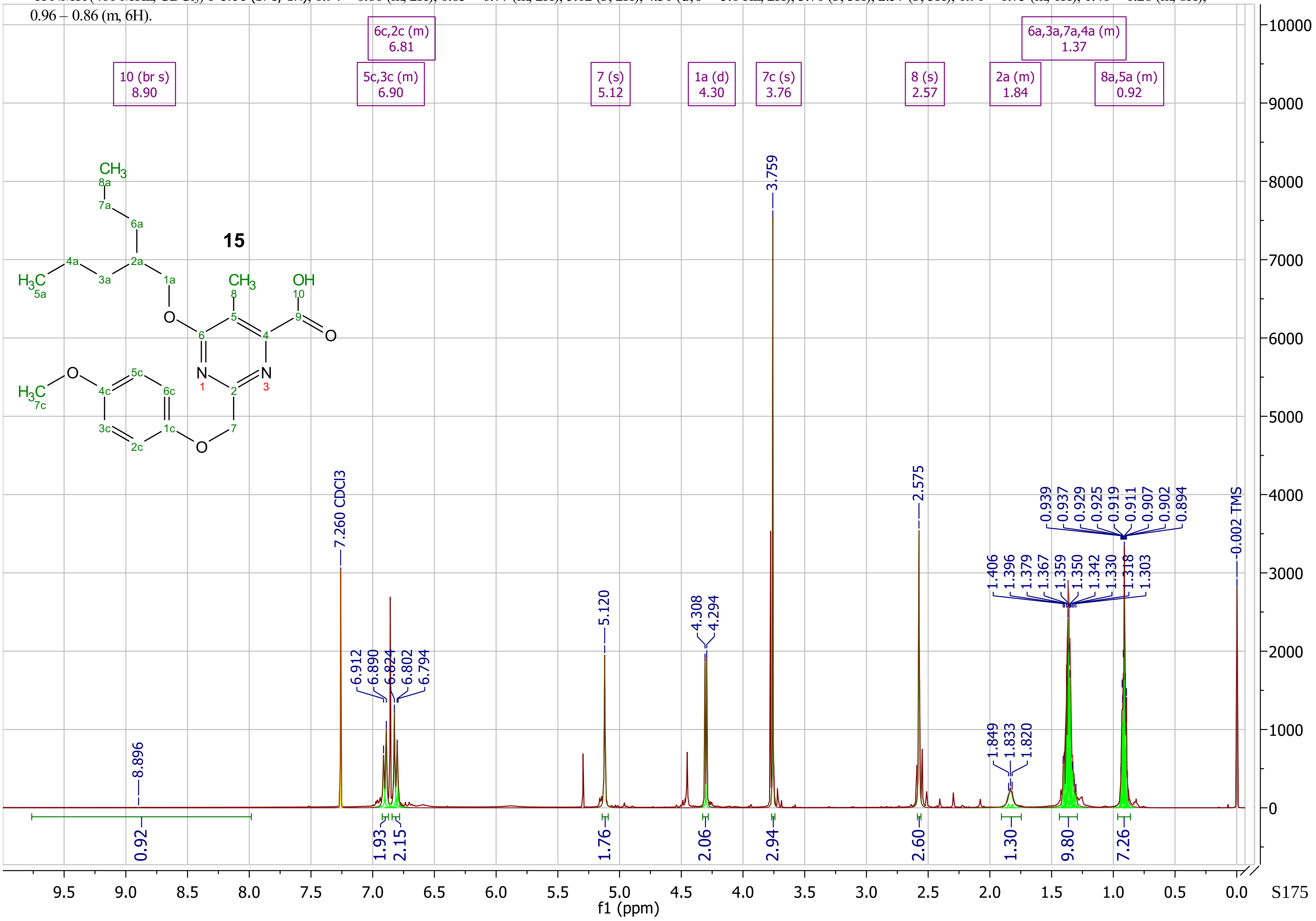

13C ^{13}C NMR (101 MHz, $CDCl_3$) δ 170.7, 163.8, 161.7, 154.4, 152.5, 148.7, 120.8, 116.0 (sym, 2C), 114.8 (sym, 2C), 71.1, 70.3, 55.8, 37.1, 33.8 (sym, 2C), 20.1 (sym, 2C), 14.5 (sym, 2C), 10.7.

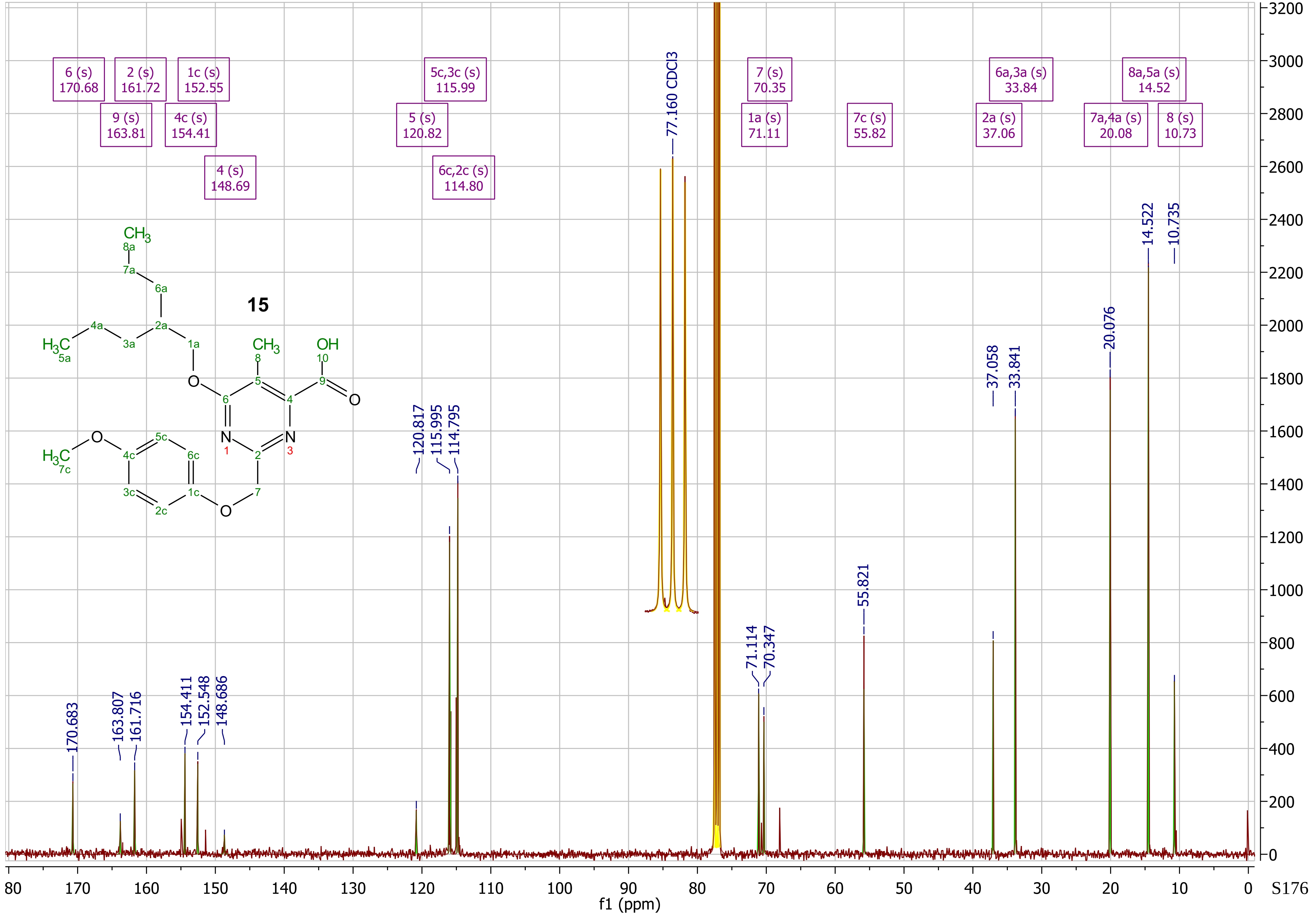

13C HSQC

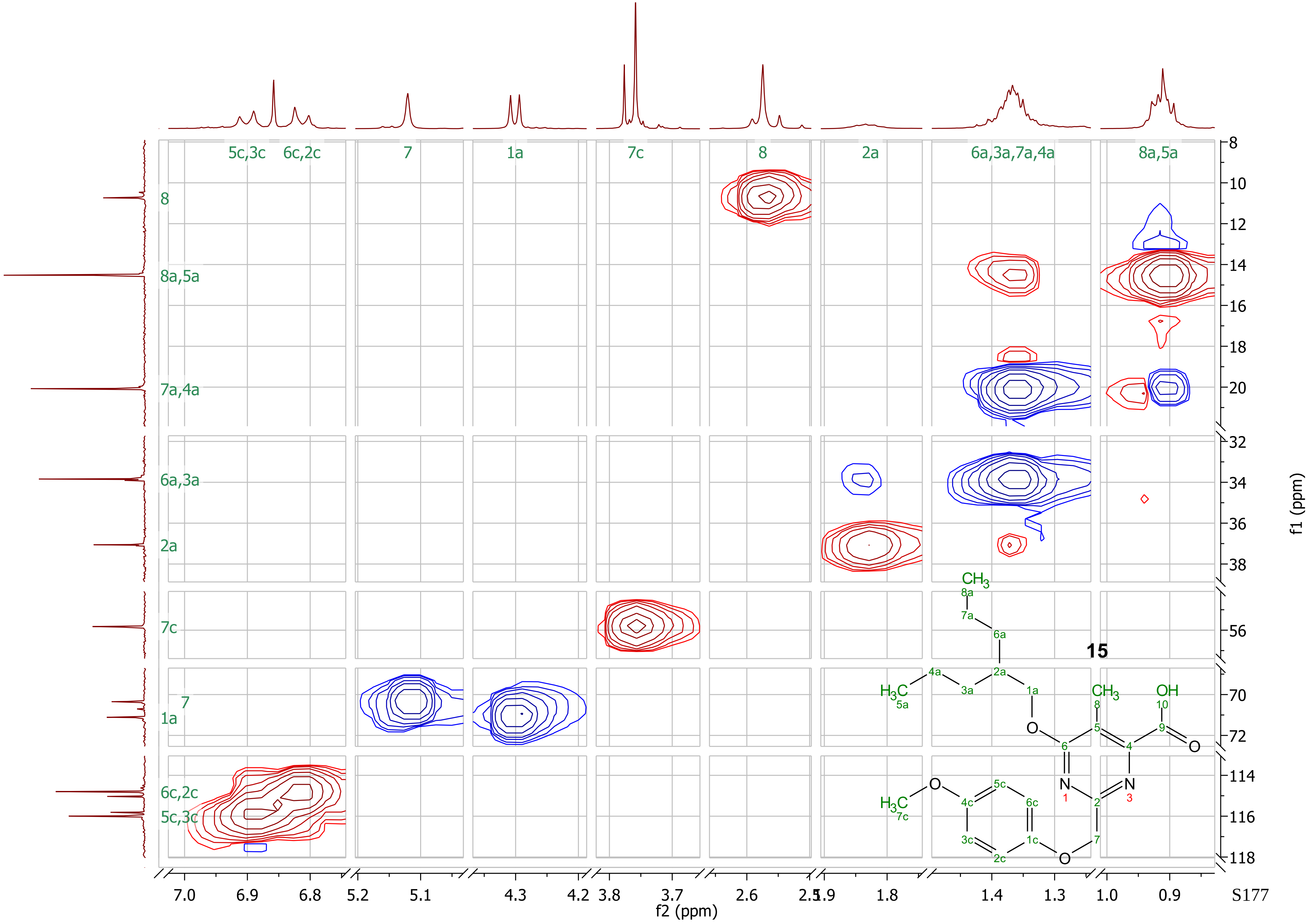

5c,3c
6c,2c
7
1a
7c
8
2a
6a,3a,7a,4a
8a,5a
8
8a,5a
7a,4a
6a,3a
2a
7c
7
1a
6c,2c
5c,3c
15
f1 (ppm)
f2 (ppm)

13C HMBC

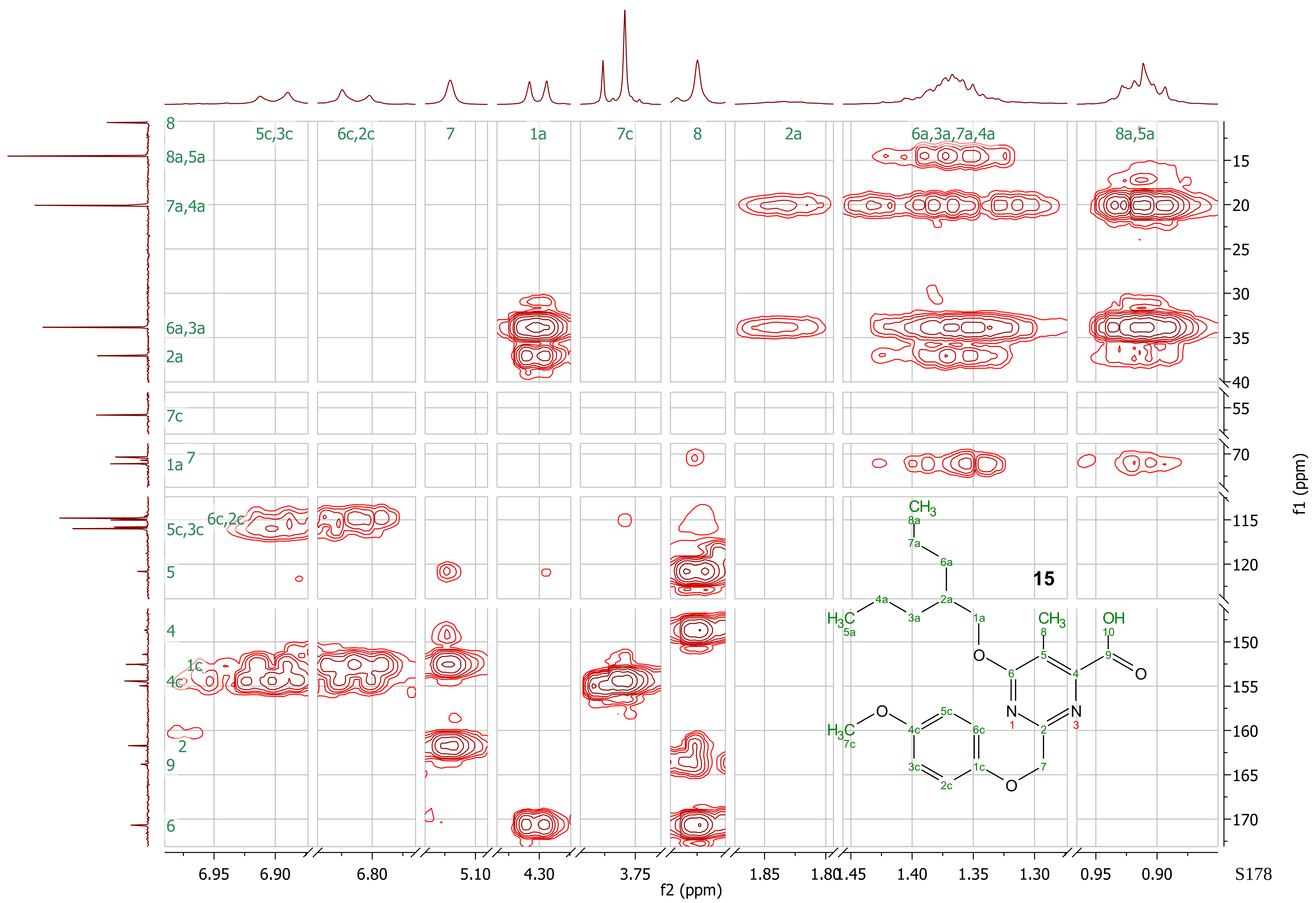
5c,3c
6c,2c
7
1a
7c
8
2a
6a,3a,7a,4a
8a,5a
7a,4a
6a,3a
2a
7c
1a
5c,3c
5
4
1c
4c
2
9
6
15
f2 (ppm)
f1 (ppm)

1H ^{1}H NMR (400 MHz, $CDCl_3$) δ 9.47 (br s, 1H), 7.71 (s, 1H), 7.62 (d, J = 7.6 Hz, 2H), 7.50 (t, J = 7.7 Hz, 1H), 6.99 – 6.86 (m, 2H), 6.86 – 6.76 (m, 2H), 5.52 (s, 2H), 5.16 (s, 2H), 3.77 (s, 3H), 2.63 (s, 3H).

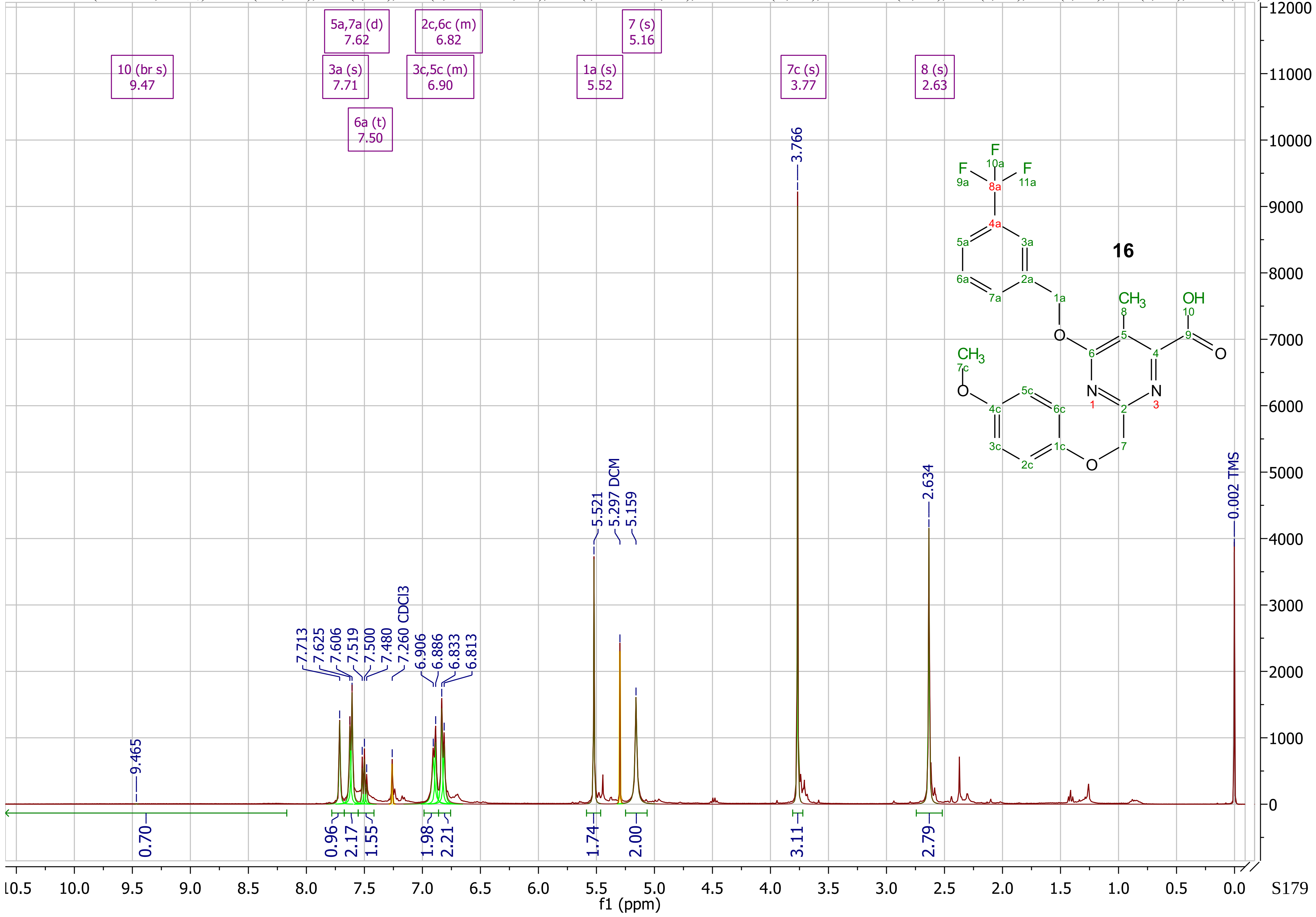

13C ^{13}C NMR (101 MHz, $CDCl_3$) δ 170.0, 163.2, 161.8, 154.5, 152.5, 149.0, 136.6, 131.7 (d, J = 1.1 Hz), 129.3, 125.4 (d, J = 3.9 Hz), 125.2 (d, J = 3.7 Hz), 121.1, 115.9 (sym, 2C), 114.9 (sym, 2C), 70.2, 68.8, 55.8, 10.8.

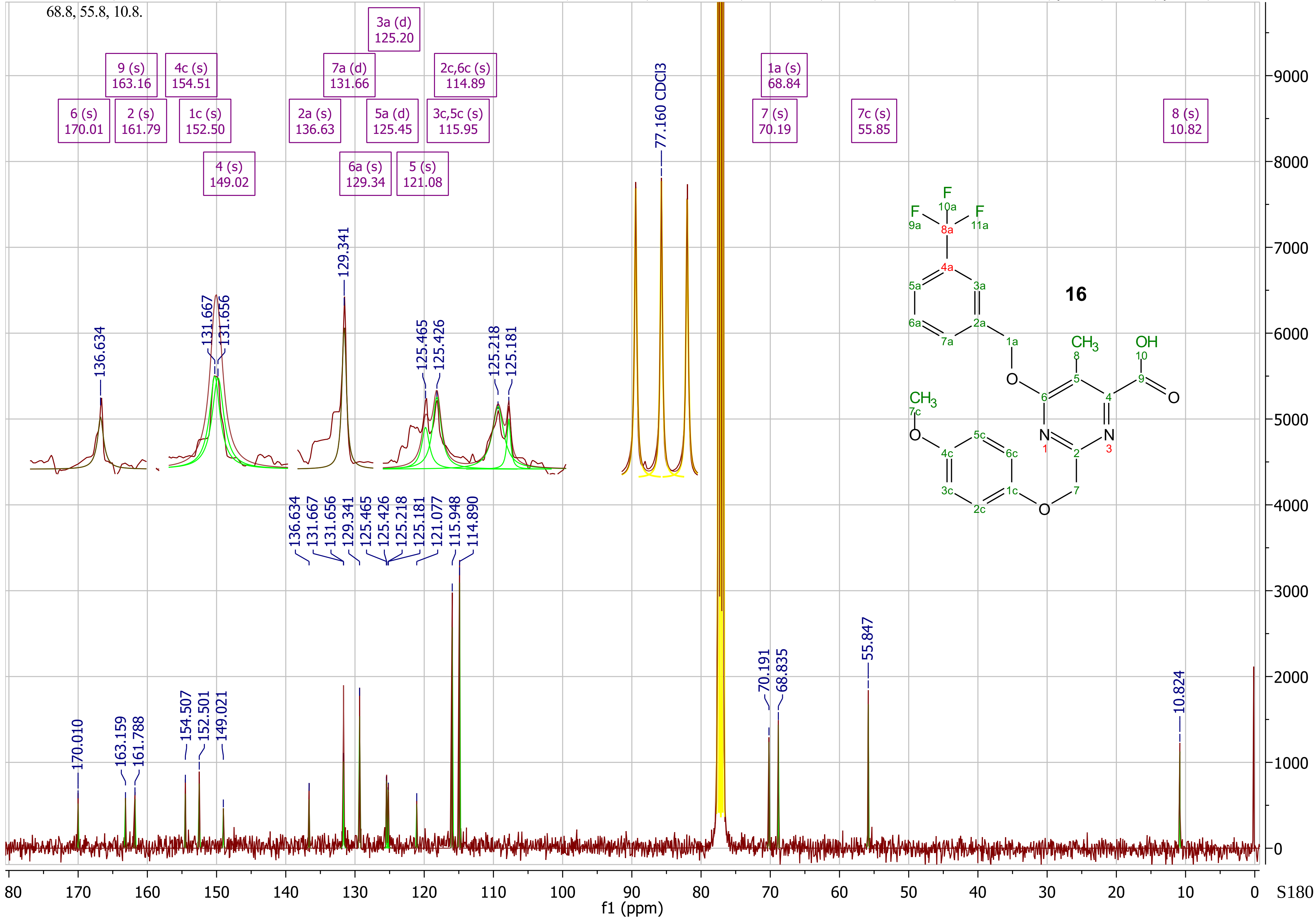

13C HSQC

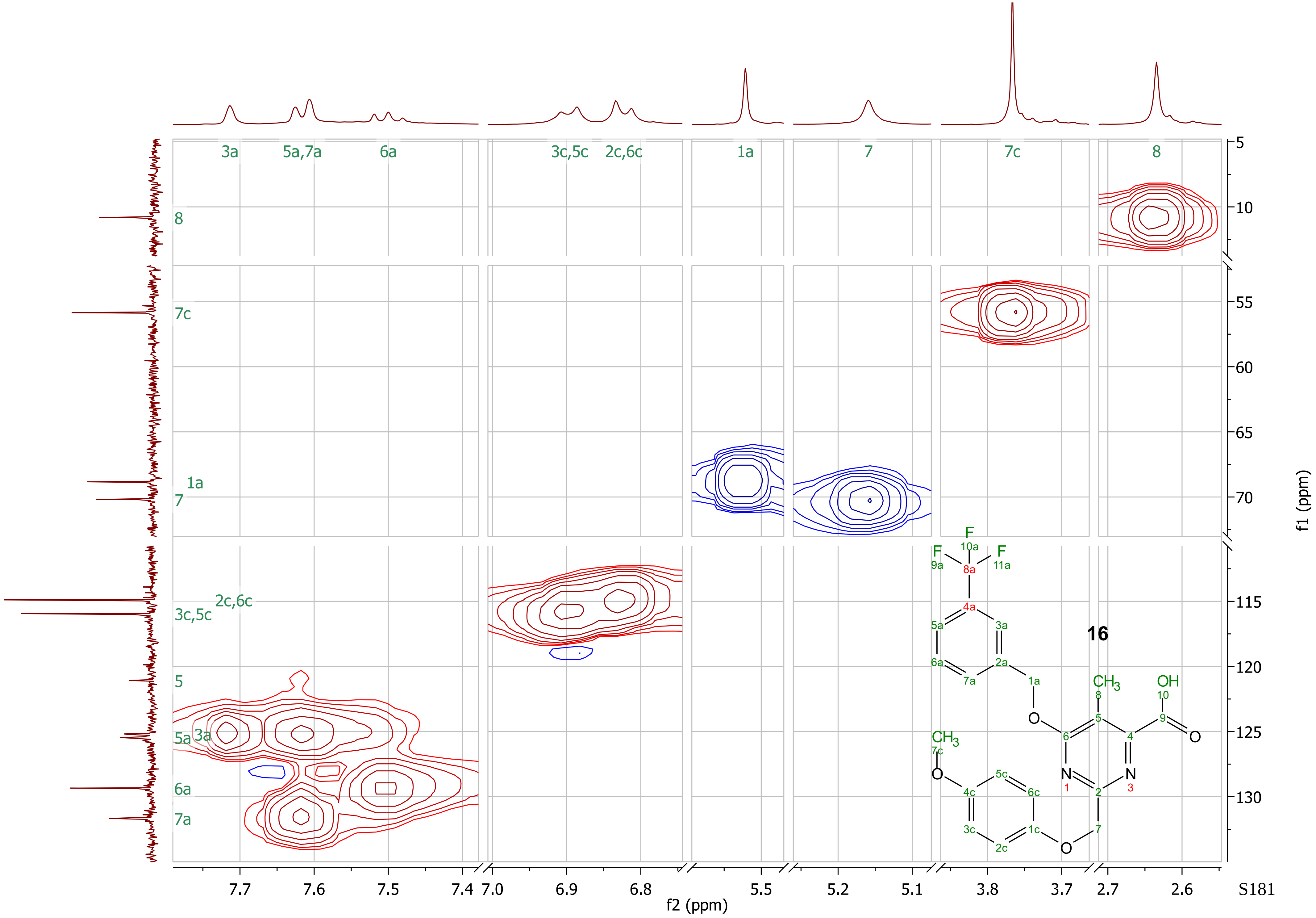

13C HMBC

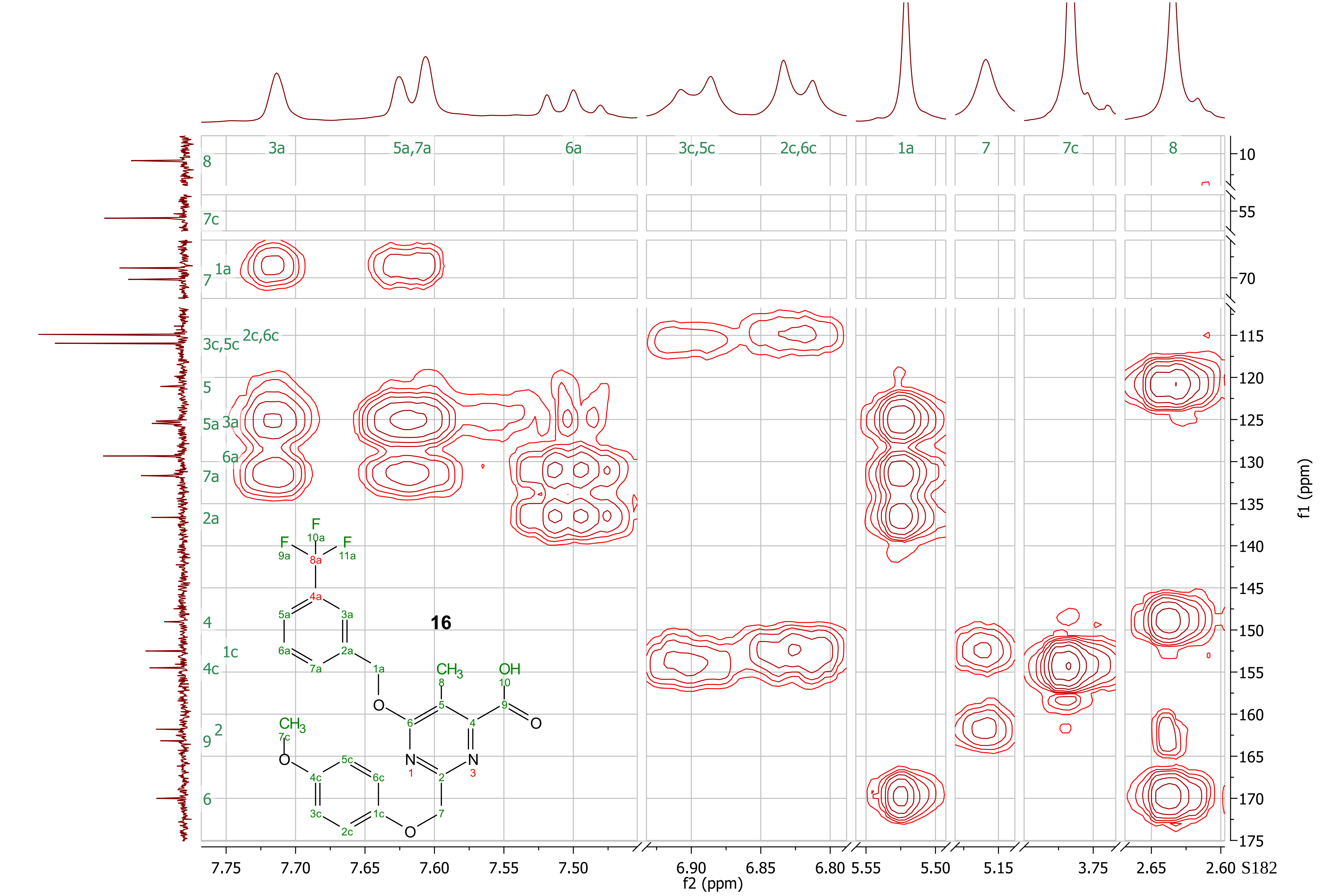

3a
5a,7a
6a
3c,5c
2c,6c
1a
7
7c
8
7c
1a
7
2c,6c
3c,5c
5
5a 3a
6a
7a
2a
4
1c
4c
9 2
6
16
f2 (ppm)
f1 (ppm)
S182

19F ^{19}F NMR (376 MHz, CDCl3) δ -62.69.

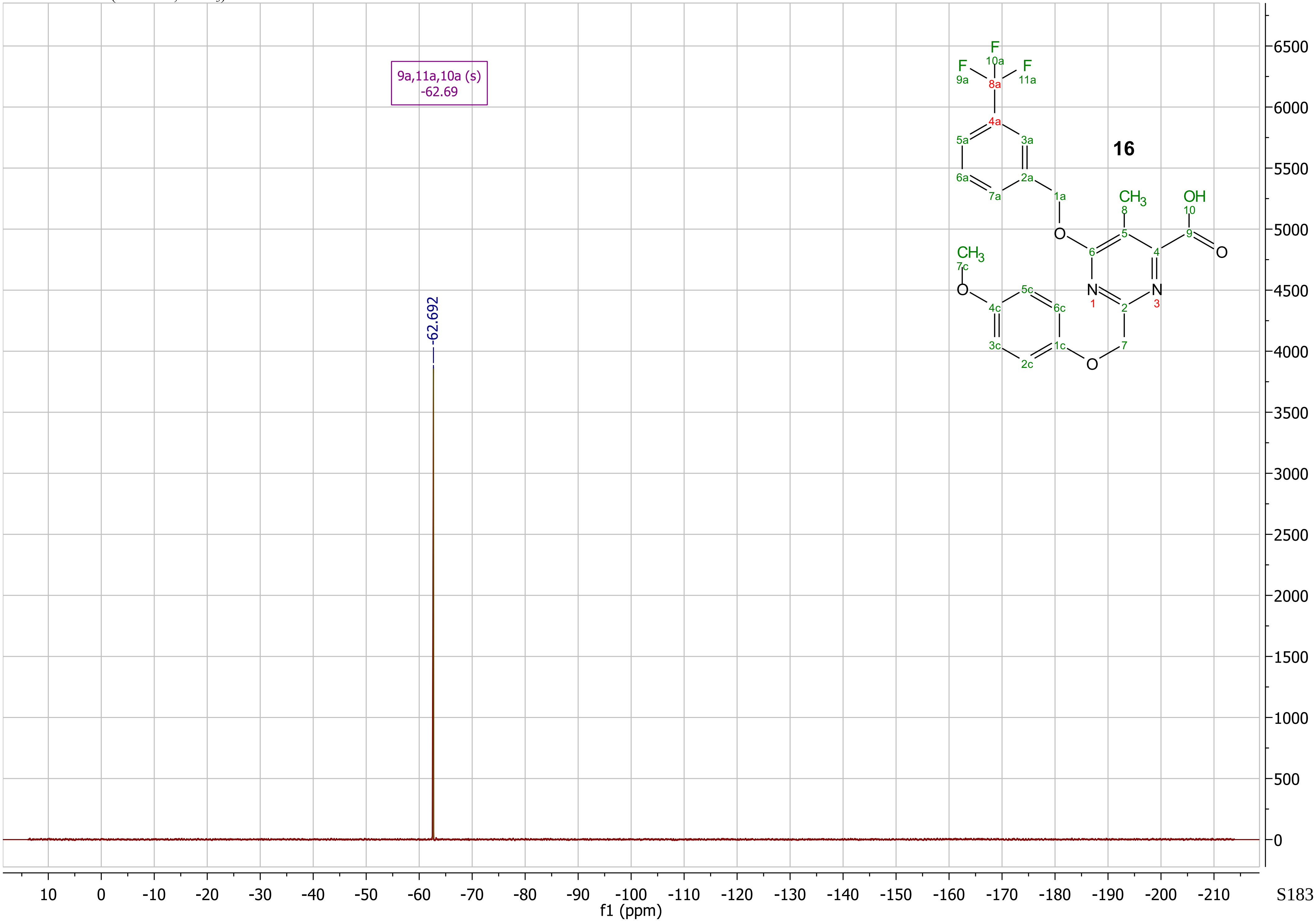

1H ^{1}H NMR (400 MHz, $CDCl_3$) δ 9.37 (br s, 1H), 6.93 – 6.87 (m, 2H), 6.85 – 6.78 (m, 2H), 5.13 (s, 2H), 4.41 (t, J = 6.6 Hz, 2H), 3.76 (s, 3H), 2.58 (s, 3H), 1.78 (quint, J = 7.2 Hz, 2H), 1.49 – 1.21 (m, 8H), 0.90 (app t, J = 7.0 Hz, 3H).

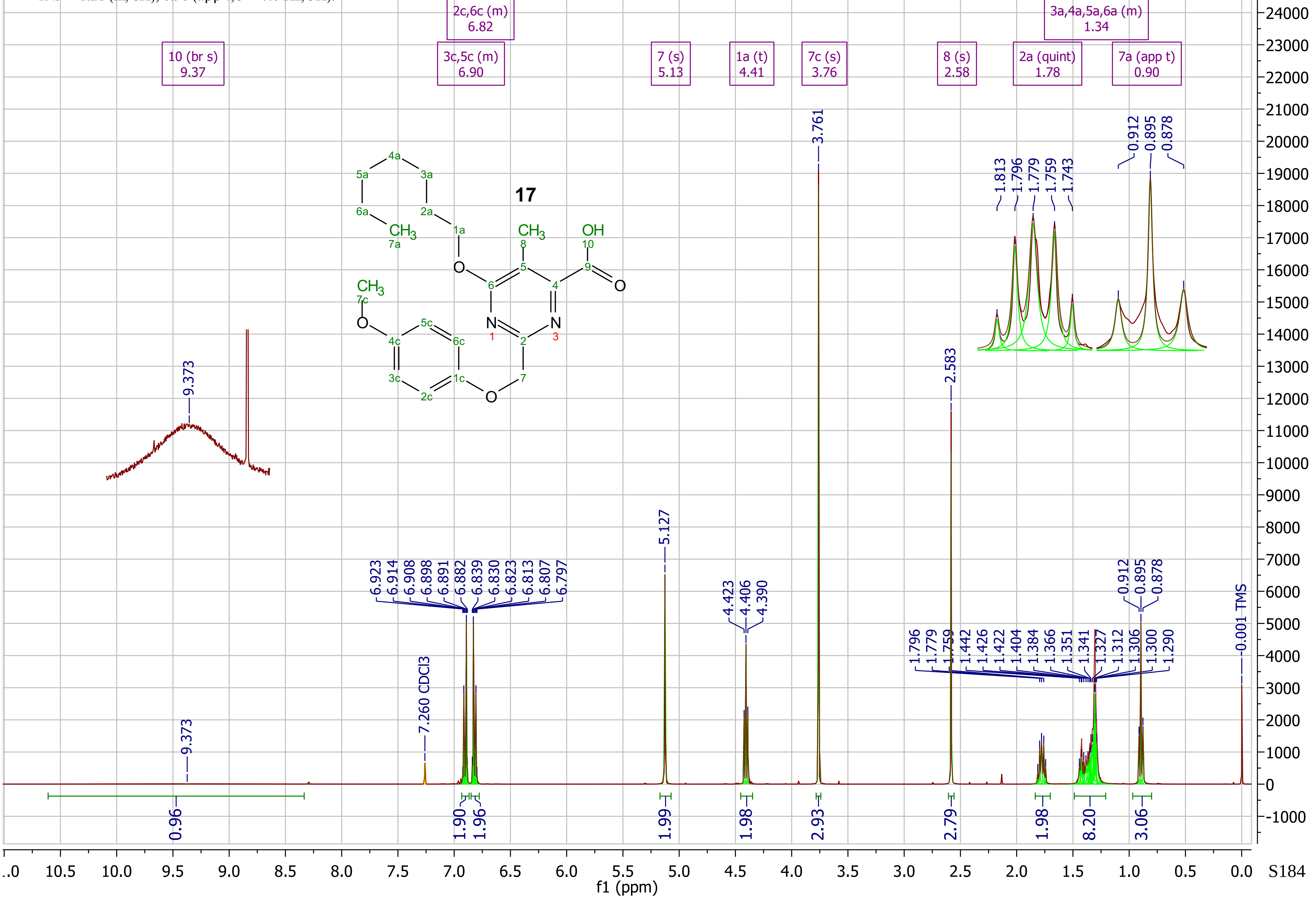

13C ^{13}C NMR (101 MHz, $CDCl_3$) δ 170.6, 163.5, 161.7, 154.4, 152.6, 148.4, 120.9, 116.0 (sym, 2C), 114.8 (sym, 2C), 70.3, 68.5, 55.8, 31.9, 29.1, 28.7, 26.0, 22.7, 14.2, 10.7.

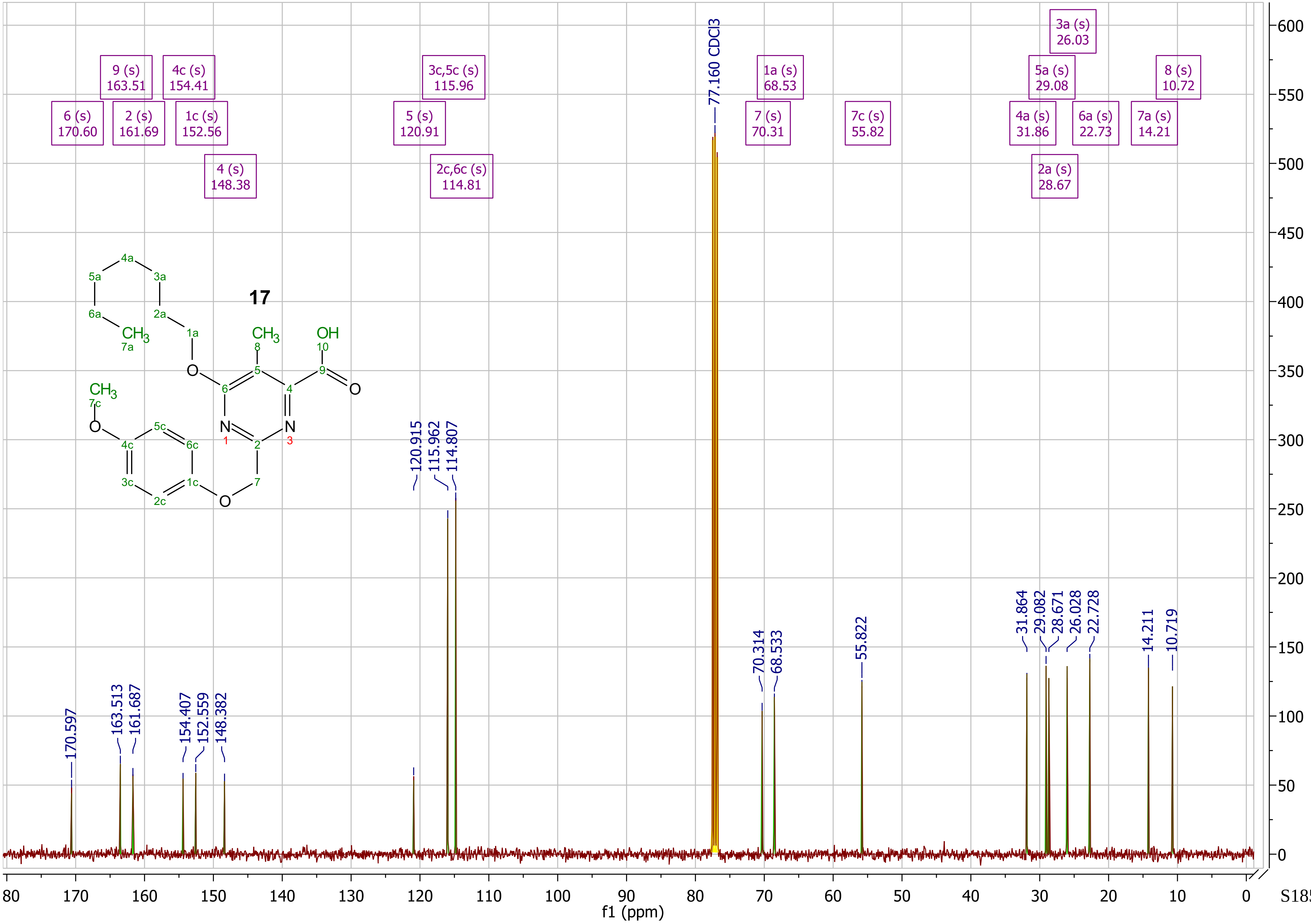

13C HSQC

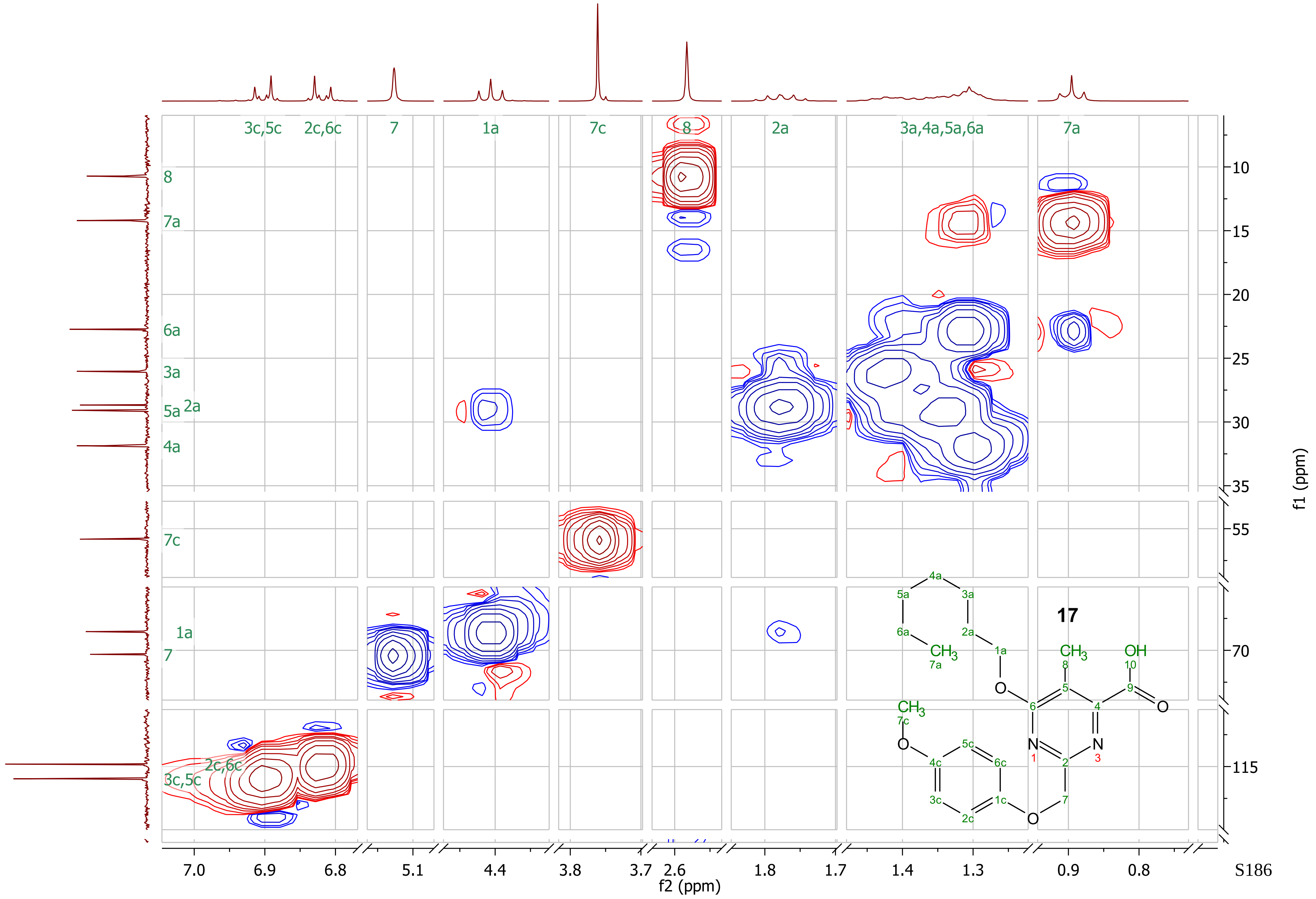

3c,5c
2c,6c
7
1a
7c
8
2a
3a,4a,5a,6a
7a
6a
3a
5a
2a
4a
1a
17
CH3
OH
O
N
f1 (ppm)
f2 (ppm)

13C HMBC

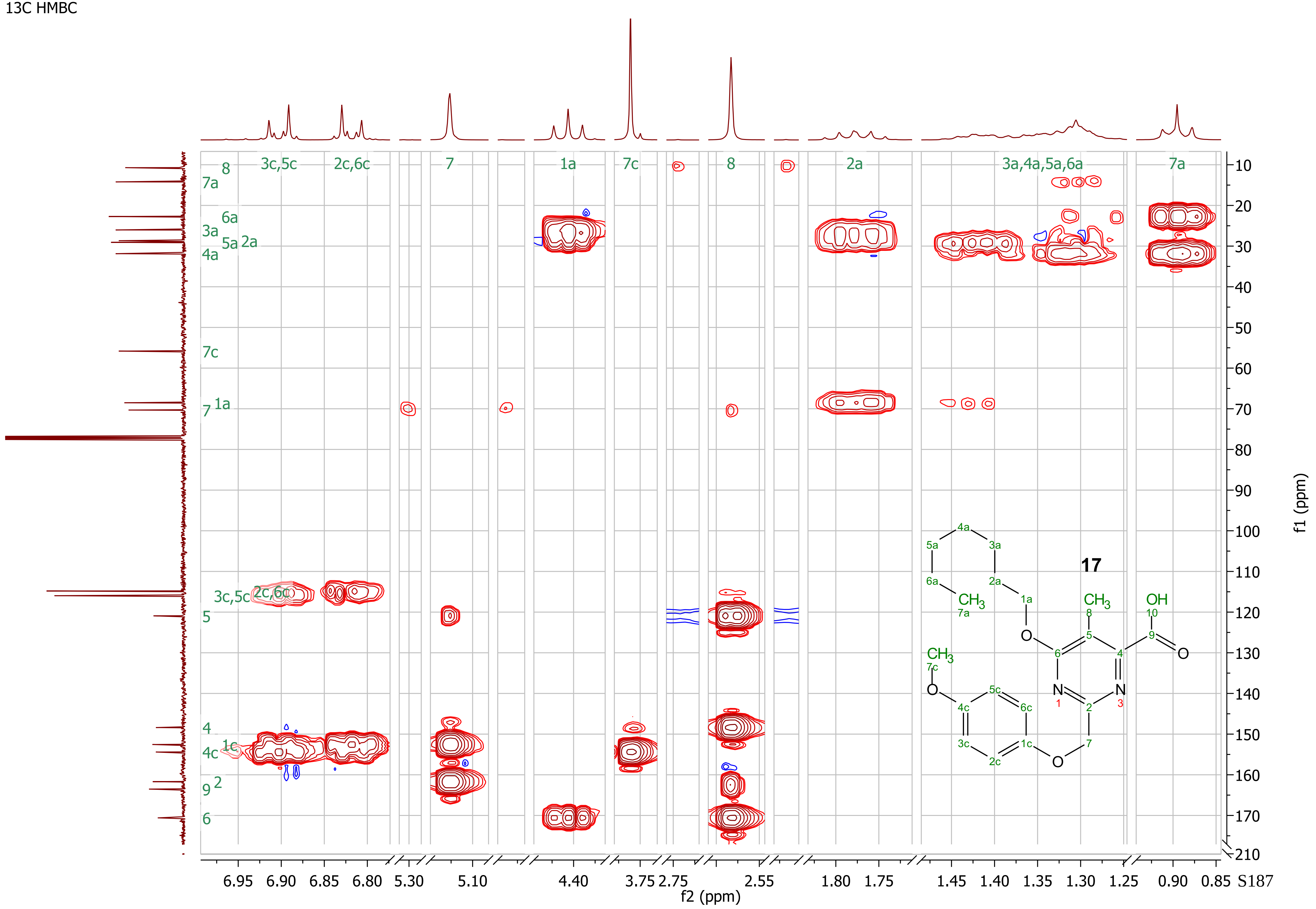

S187

1H ^1H NMR (400 MHz, $CDCl_3$) δ 9.04 (br s, 1H), 6.98 – 6.85 (m, 2H), 6.85 – 6.71 (m, 2H), 5.25 (quint, J = 5.9 Hz, 1H), 5.11 (s, 2H), 3.76 (s, 3H), 2.57 (s, 3H), 1.78 – 1.59 (m, 4H), 1.38 – 1.17 (m, 4H), 0.97 – 0.80 (m, 6H).

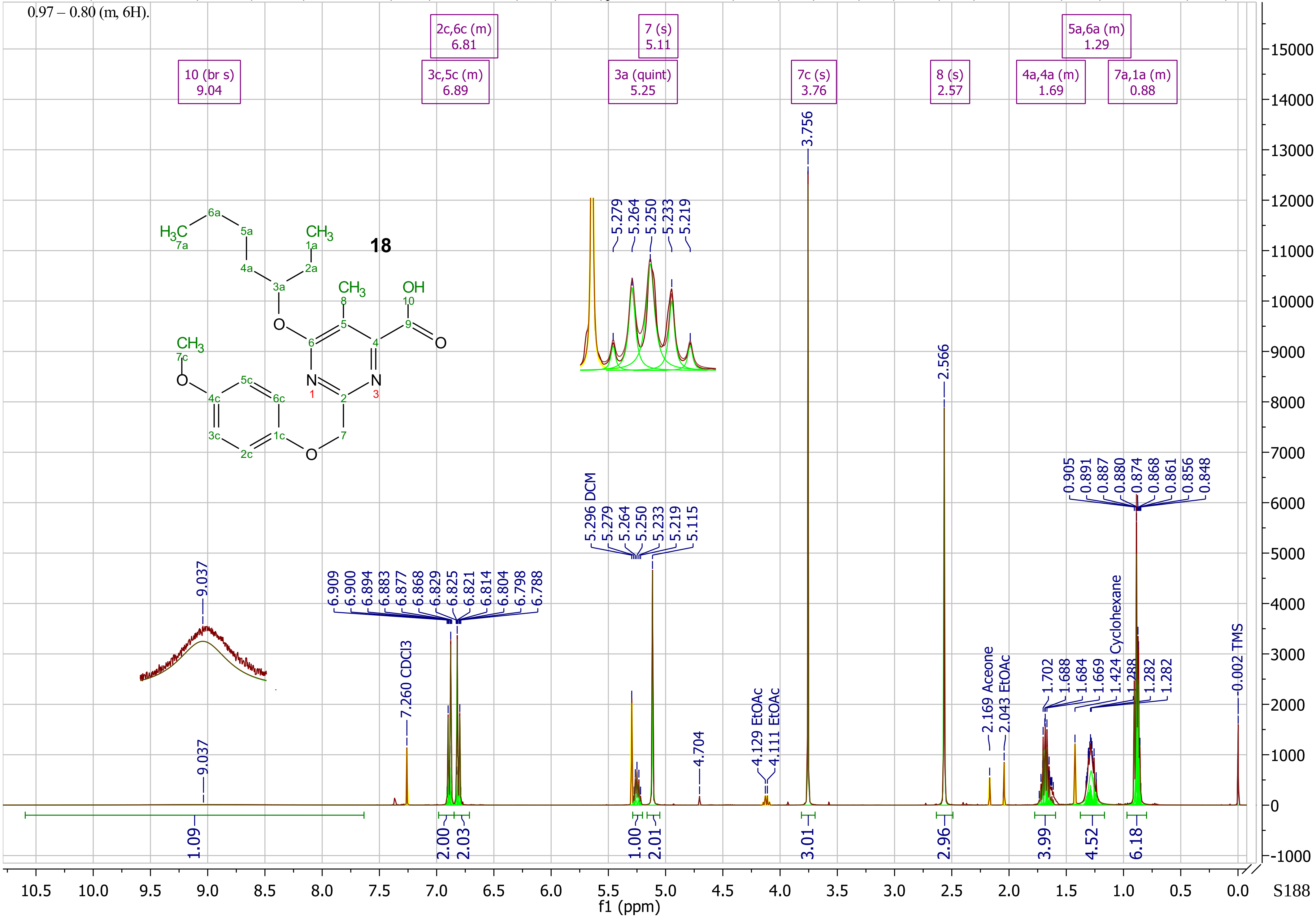

13C ^{13}C NMR (101 MHz, $CDCl_3$) δ 170.6, 163.7, 161.6, 154.4, 152.6, 148.6, 120.9, 115.9 (sym, 2C), 114.8 (sym, 2C), 79.5, 70.2, 55.8, 32.9, 27.5, 26.6, 22.7, 14.1, 10.7, 9.6.

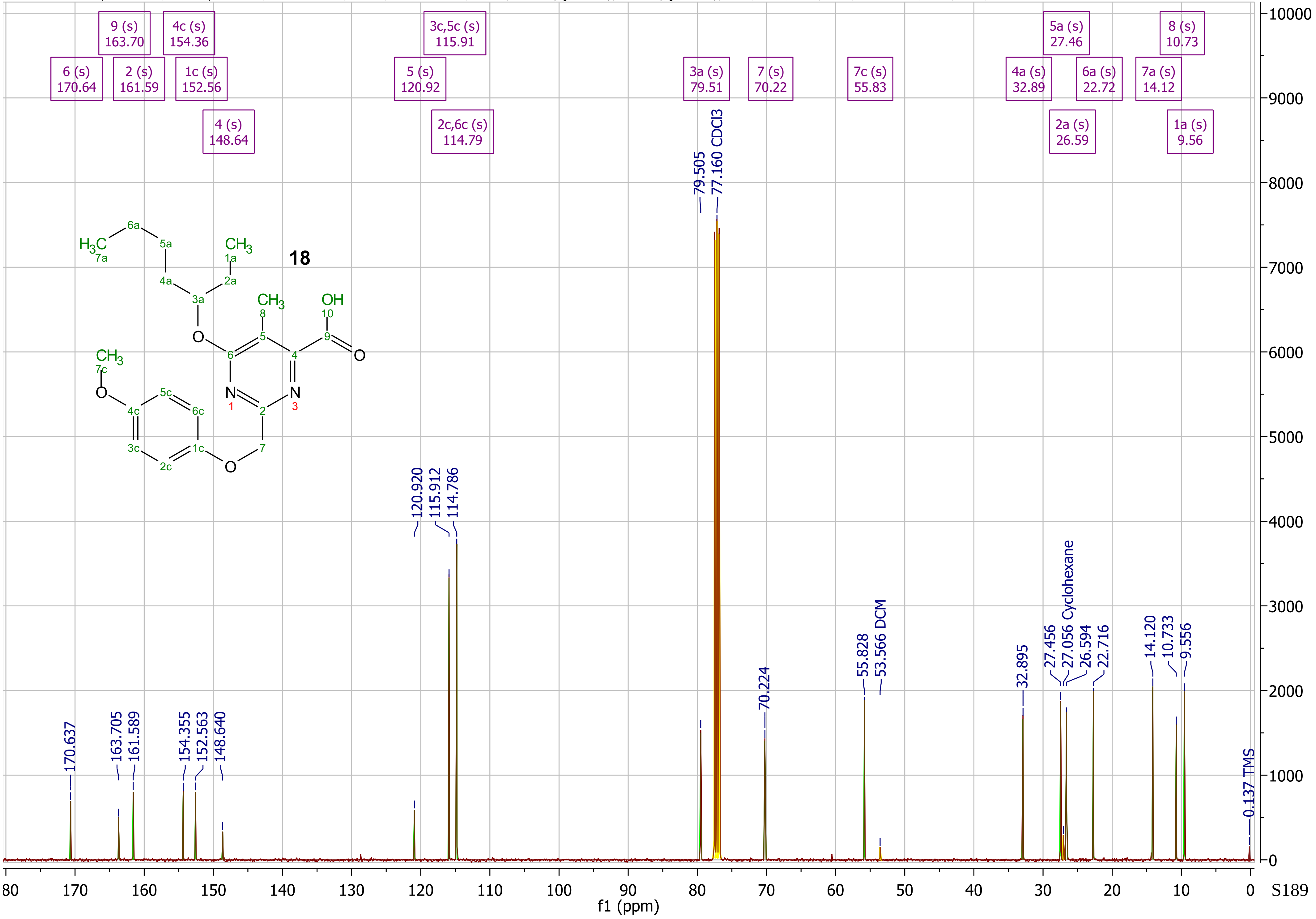

13C HSQC

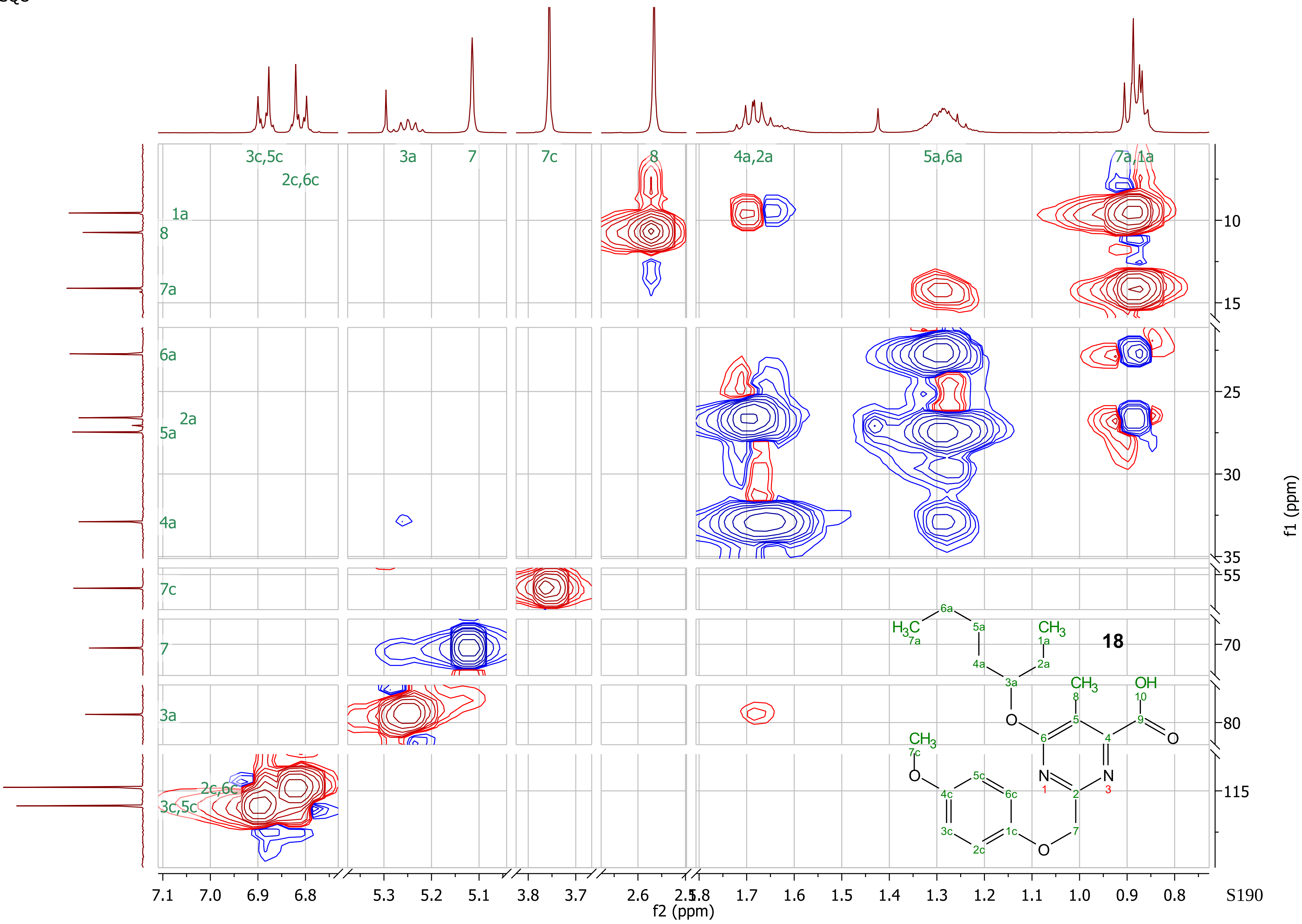

3c,5c
2c,6c
3a
7
7c
8
4a,2a
5a,6a
7a,1a
1a
8
7a
6a
2a
5a
4a
7c
7
3a
2c,6c
3c,5c
18
f2 (ppm)
f1 (ppm)

13C HMBC

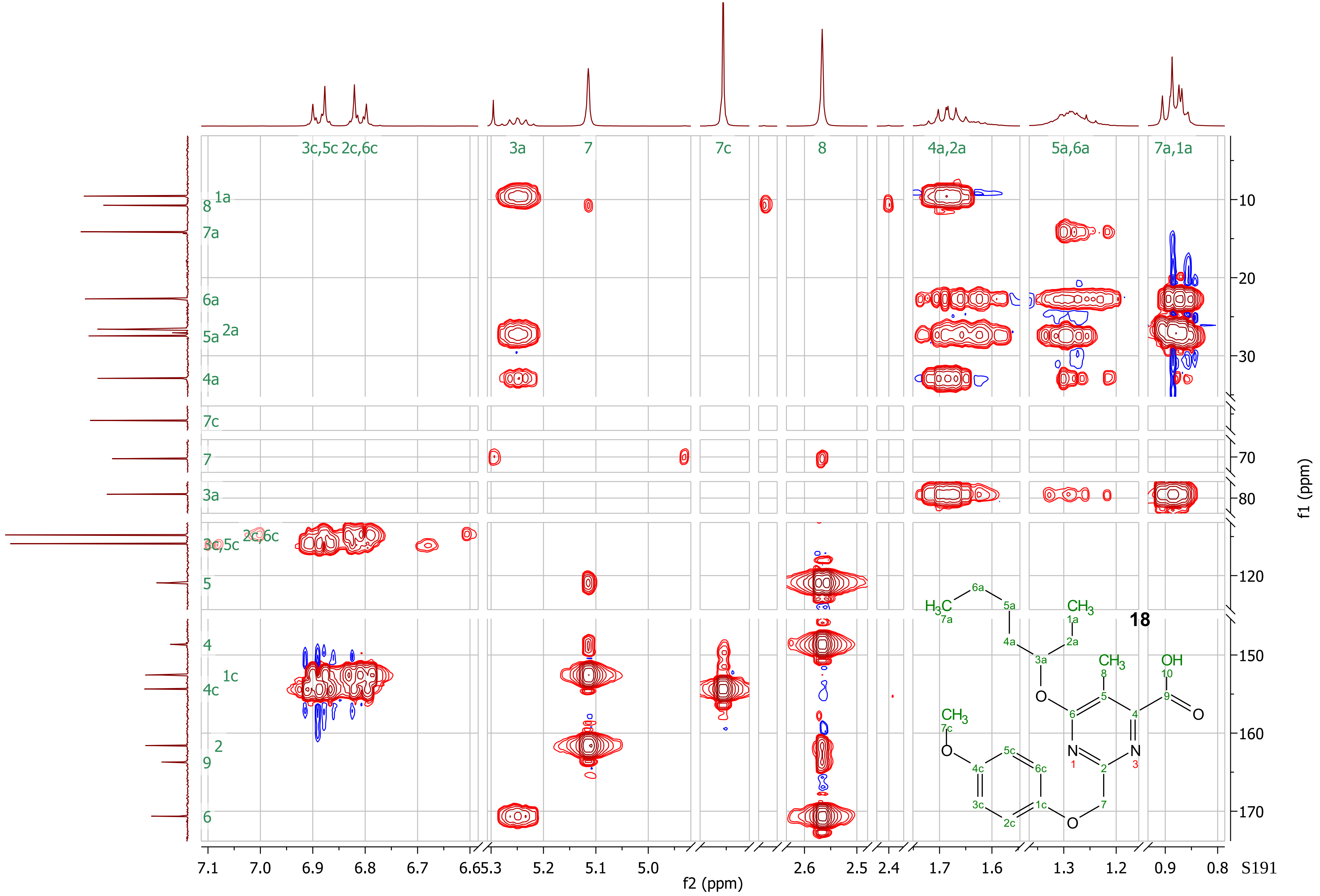

3c,5c 2c,6c
3a
7
7c
8
4a,2a
5a,6a
7a,1a
8 1a
7a
6a
5a 2a
4a
7c
7
3a
3c,5c 2c,6c
5
4
1c
4c
2
9
6
18
f2 (ppm)
f1 (ppm)
S191

1H ^{1}H NMR (400 MHz, $CDCl_3$) δ 7.00 – 6.86 (m, 2H), 6.86 – 6.72 (m, 2H), 5.13 (s, 2H), 4.37 (t, J = 6.9 Hz, 2H), 4.35 (t, J = 6.7 Hz, 2H), 3.75 (s, 3H), 2.26 (s, 3H), 1.77 (quint, J = 7.2 Hz, 2H), 1.71 (quint, J = 7.1 Hz, 2H), 1.48 – 1.36 (m, 4H), 1.36 – 1.24 (m, 8H), 0.89 (app t, J = 7.2 Hz, 6H).

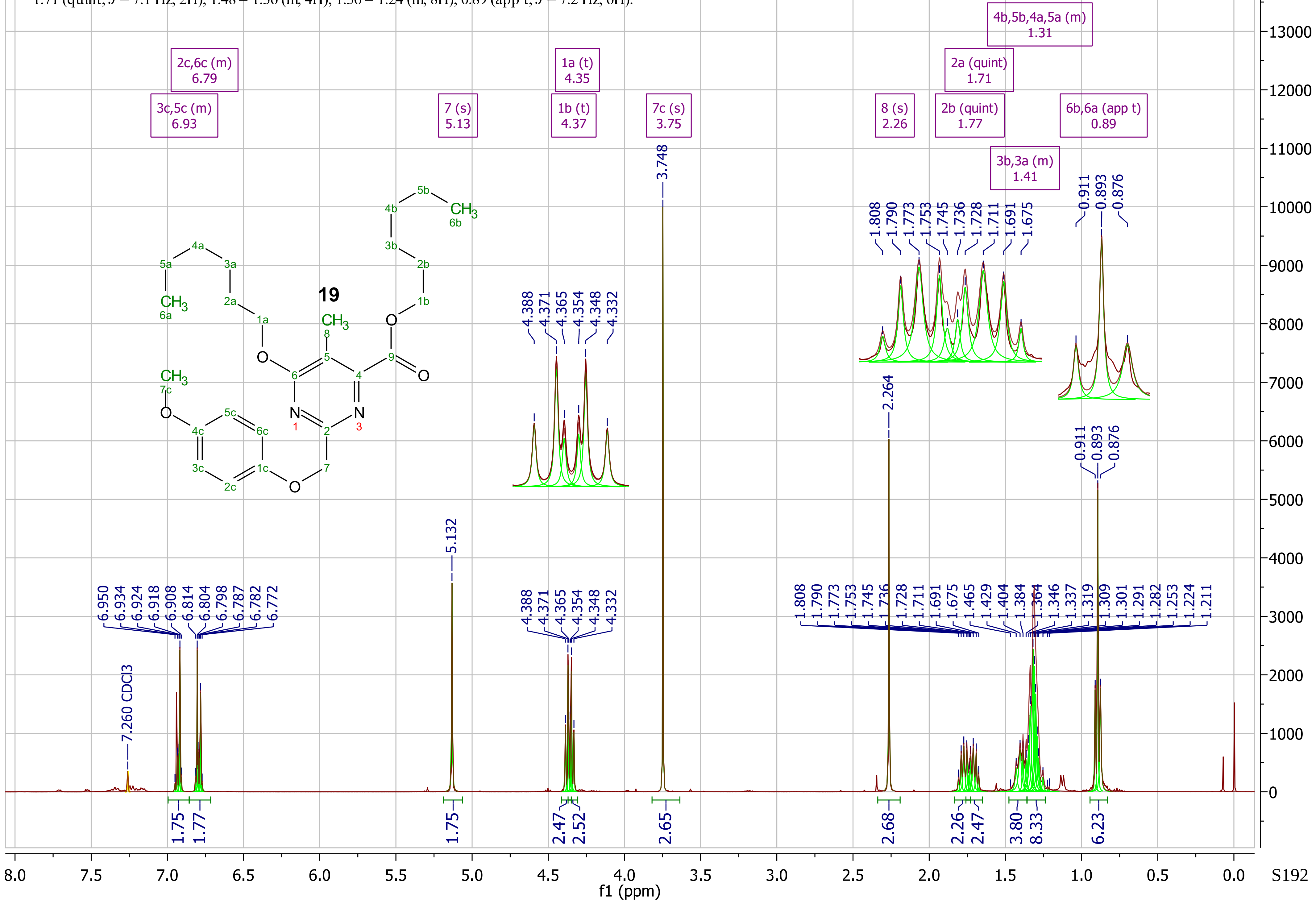

13C ^{13}C NMR (101 MHz, $CDCl_3$) δ 169.3, 165.9, 163.1, 155.1, 154.1, 153.0, 116.6, 116.1 (sym, 2C), 114.6 (sym, 2C), 71.2, 67.6, 66.4, 55.8, 31.6, 31.5, 28.7, 28.6, 25.73, 25.68, 22.7, 22.6, 14.1 (2C), 11.1.

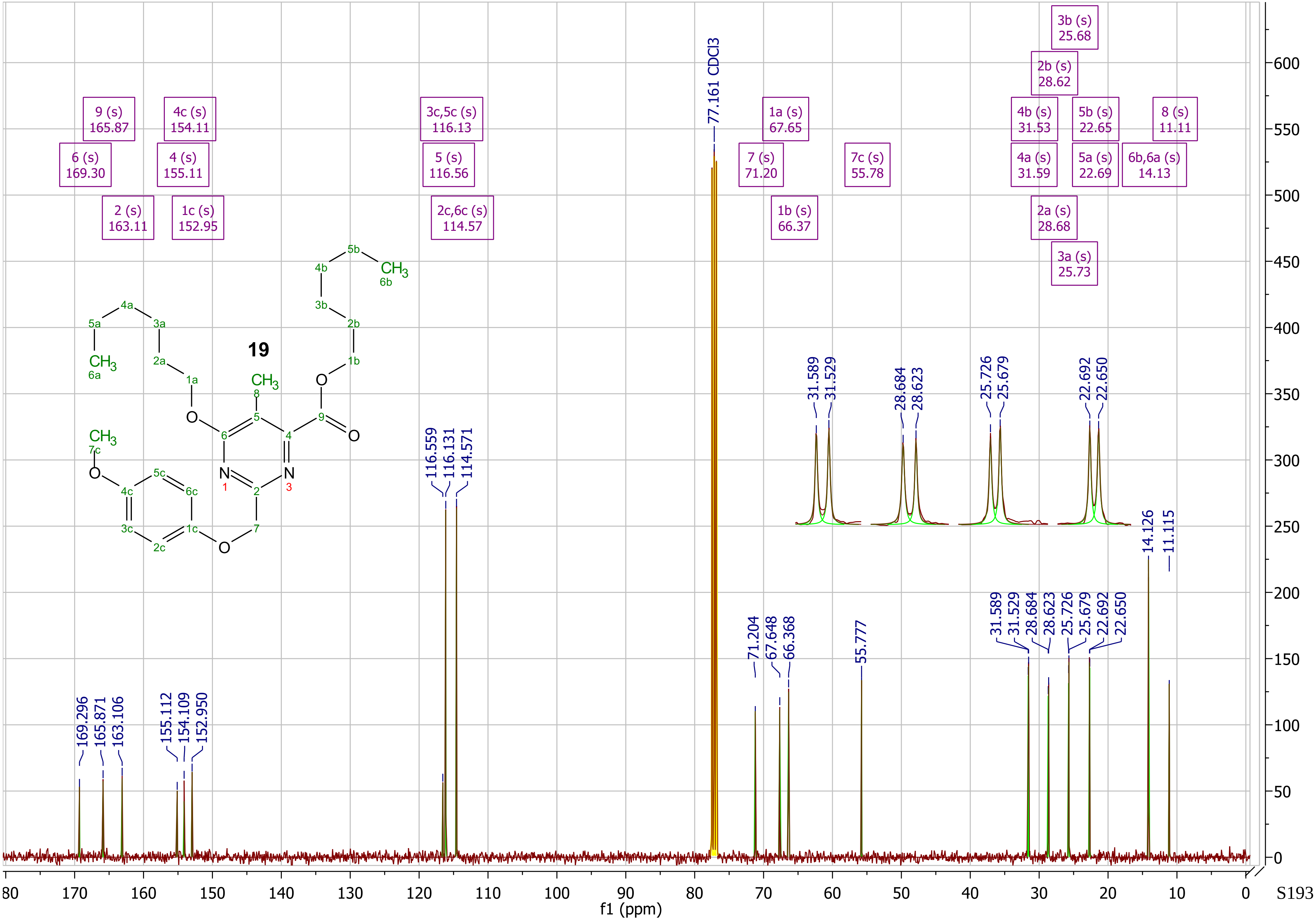

13C HSQC

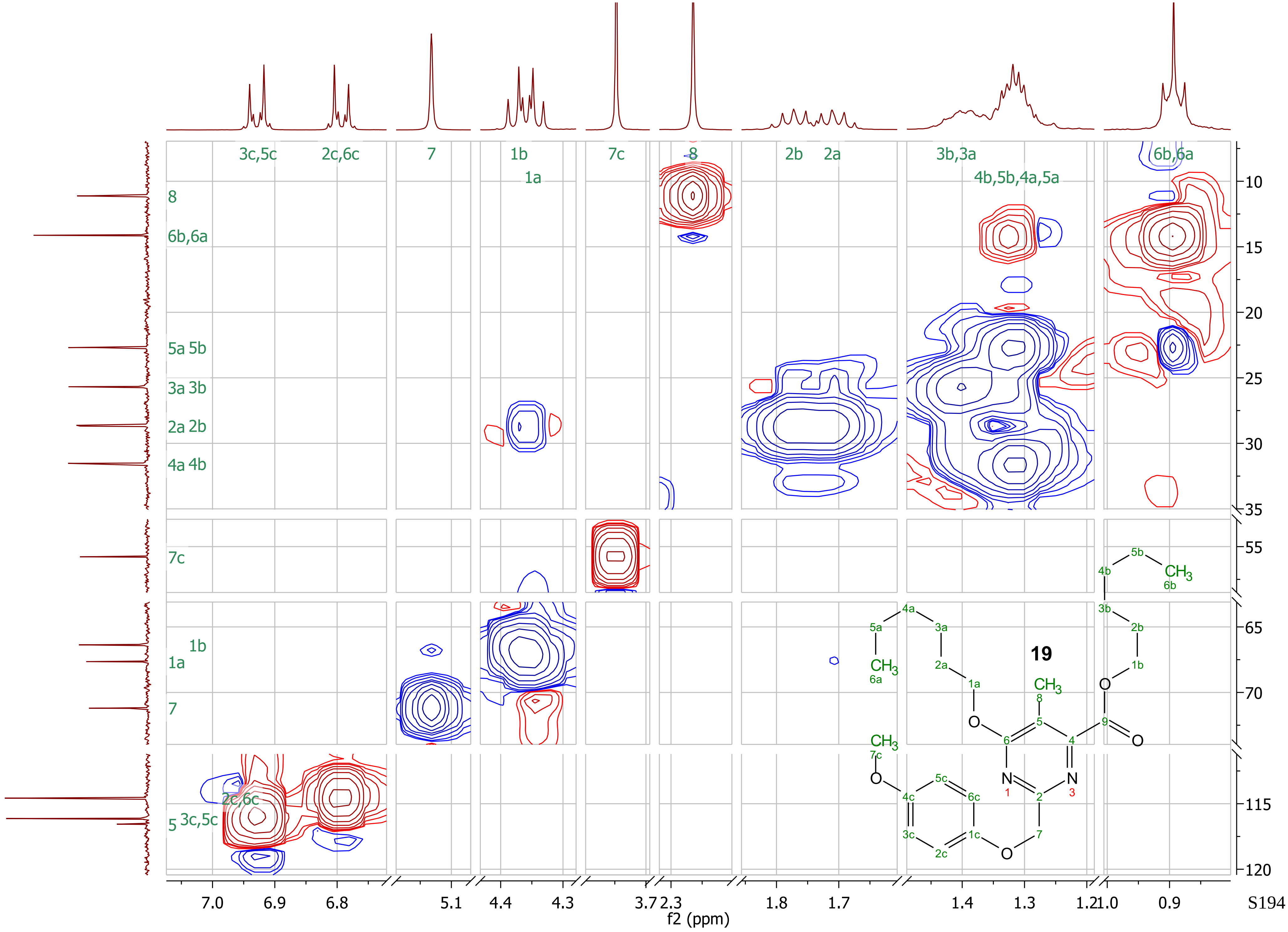

3c,5c
2c,6c
7
1b
1a
7c
8
2b
2a
3b,3a
4b,5b,4a,5a
6b,6a
8
6b,6a
5a 5b
3a 3b
2a 2b
4a 4b
7c
1b
1a
7
2c,6c
5 3c,5c
19
f1 (ppm)
f2 (ppm)

13C HMBC

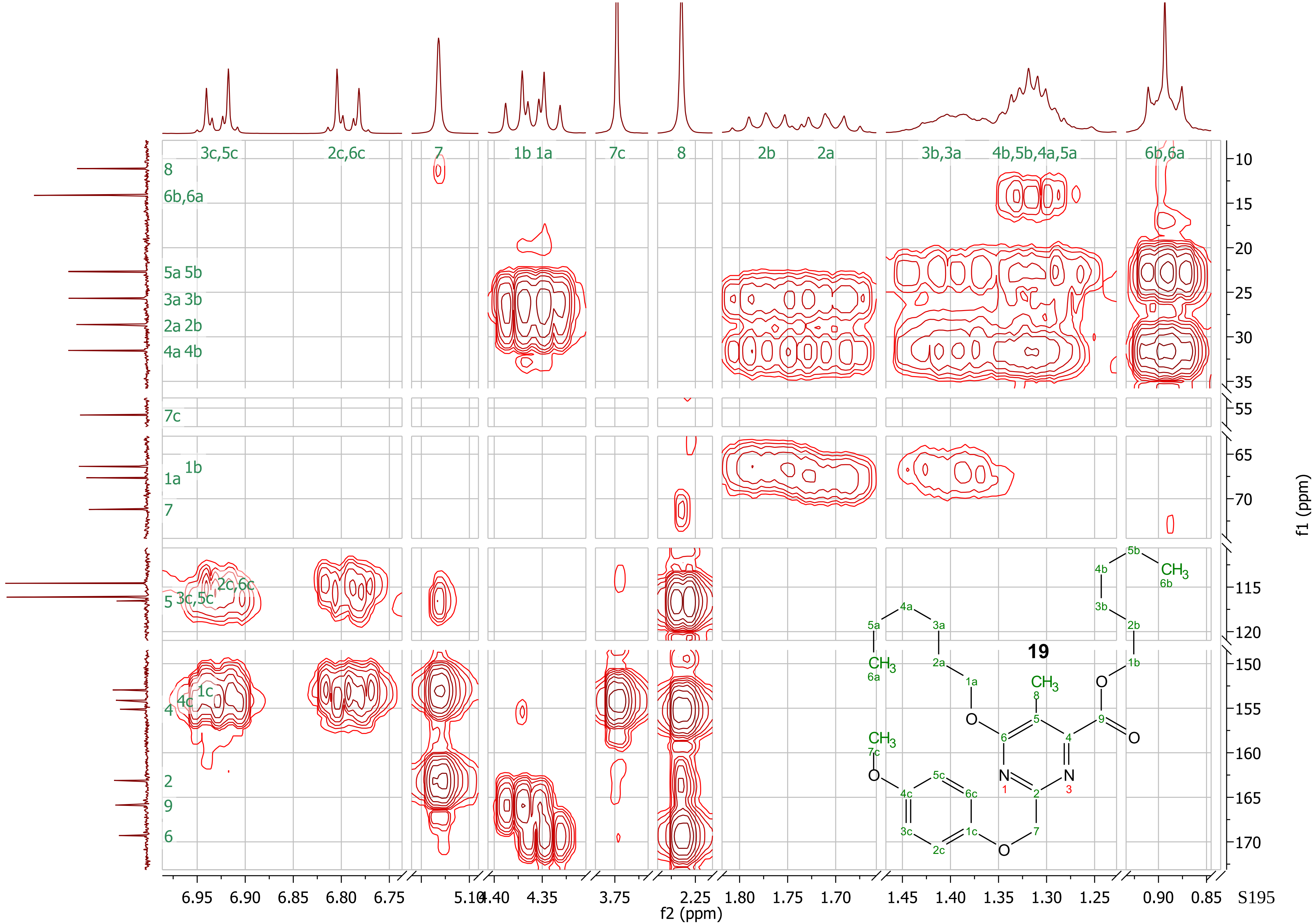

3c,5c
2c,6c
7
1b 1a
7c
8
2b
2a
3b,3a
4b,5b,4a,5a
6b,6a
8
6b,6a
5a 5b
3a 3b
2a 2b
4a 4b
7c
1b
1a
7
5
3c,5c
2c,6c
4
4c
1c
2
9
6
19
f2 (ppm)
f1 (ppm)

^{1}H NMR (400 MHz, $CDCl_3$) δ 6.98 – 6.86 (m, 2H), 6.86 – 6.74 (m, 2H), 5.13 (s, 2H), 4.29 (d, *J* = 5.8 Hz, 2H), 4.25 (d, *J* = 5.6 Hz, 2H), 3.75 (s, 3H), 2.26 (s, 3H), 1.93 – 1.67 (m, 2H), 1.44 – 1.24 (m, 16H), 0.98 – 0.81 (m, 12H).

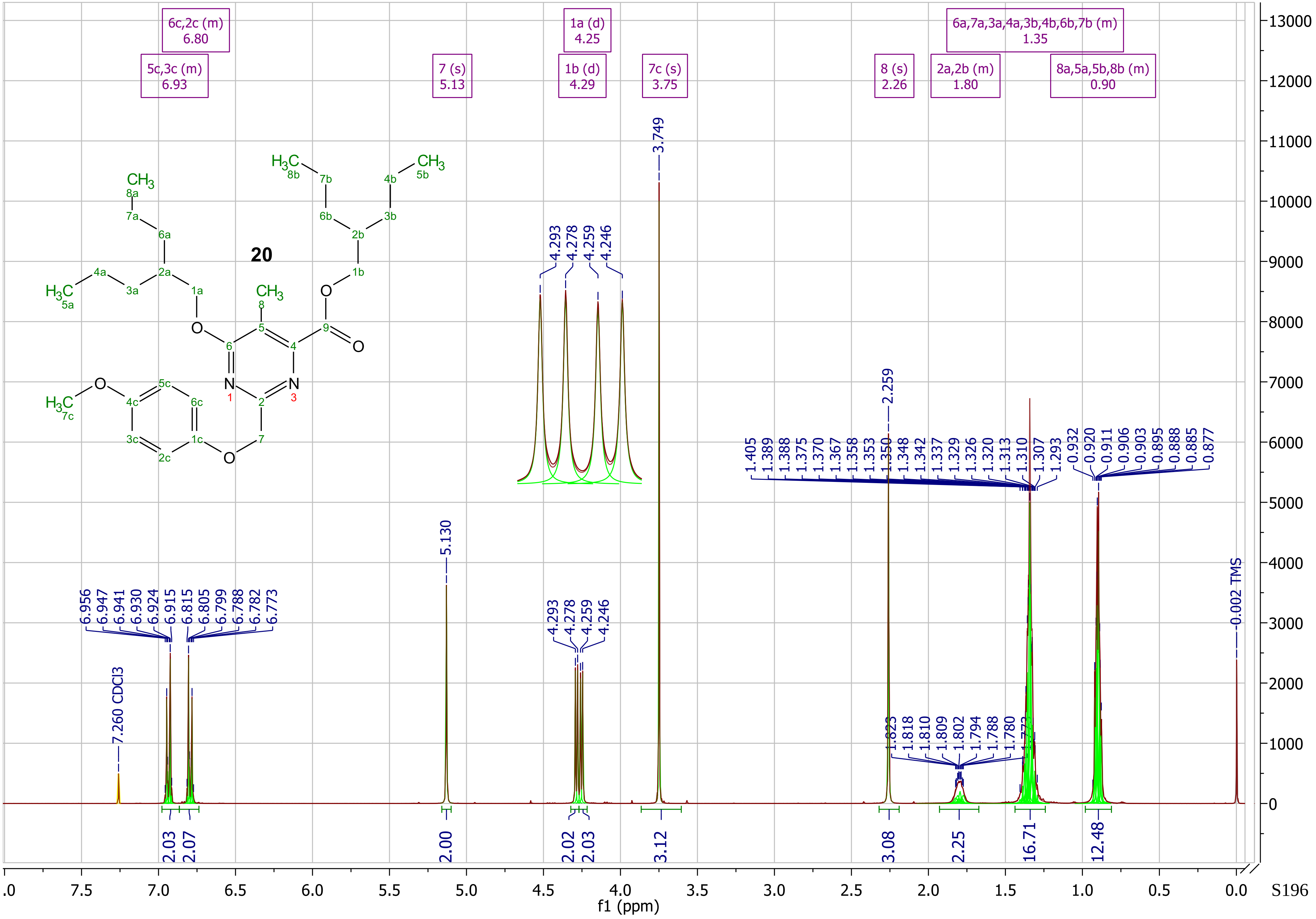

^{13}C NMR (101 MHz, $CDCl_3$) δ 169.4, 166.1, 163.1, 155.3, 154.1, 153.0, 116.3, 116.2 (sym, 2C), 114.6 (sym, 2C), 71.3, 70.2, 69.0, 55.8, 37.1, 37.0, 33.9 (sym, 2C), 33.6 (sym, 2C), 20.1 (sym, 2C), 20.0 (sym, 2C), 14.54 (sym, 2C), 14.50 (sym, 2C), 11.1.

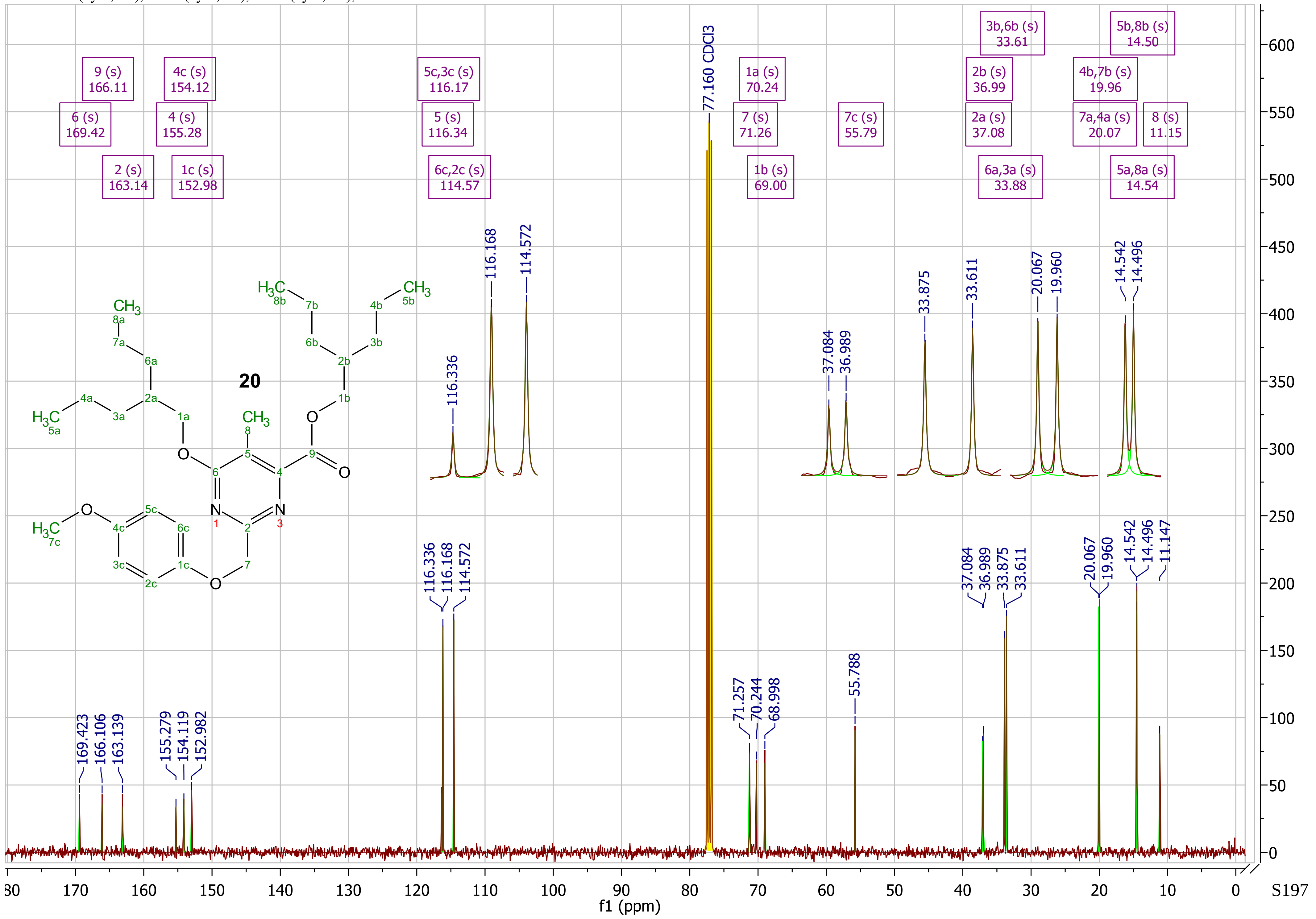

13C HSQC

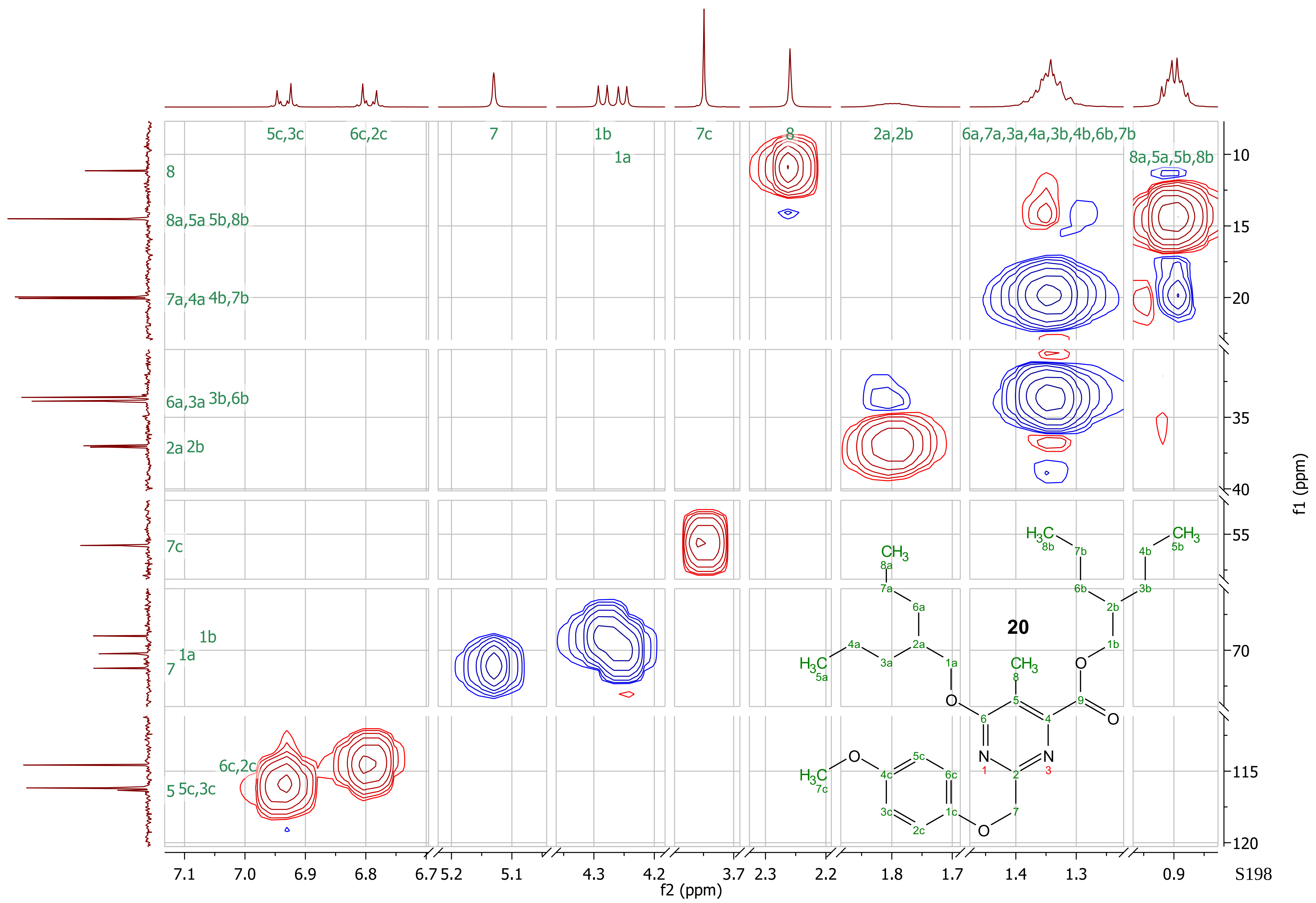

5c,3c
6c,2c
7
1b
1a
7c
8
2a,2b
6a,7a,3a,4a,3b,4b,6b,7b
8a,5a,5b,8b
8
8a,5a 5b,8b
7a,4a 4b,7b
6a,3a 3b,6b
2a 2b
7c
1b
1a
7
6c,2c
5 5c,3c
20
f1 (ppm)
f2 (ppm)

13C HMBC

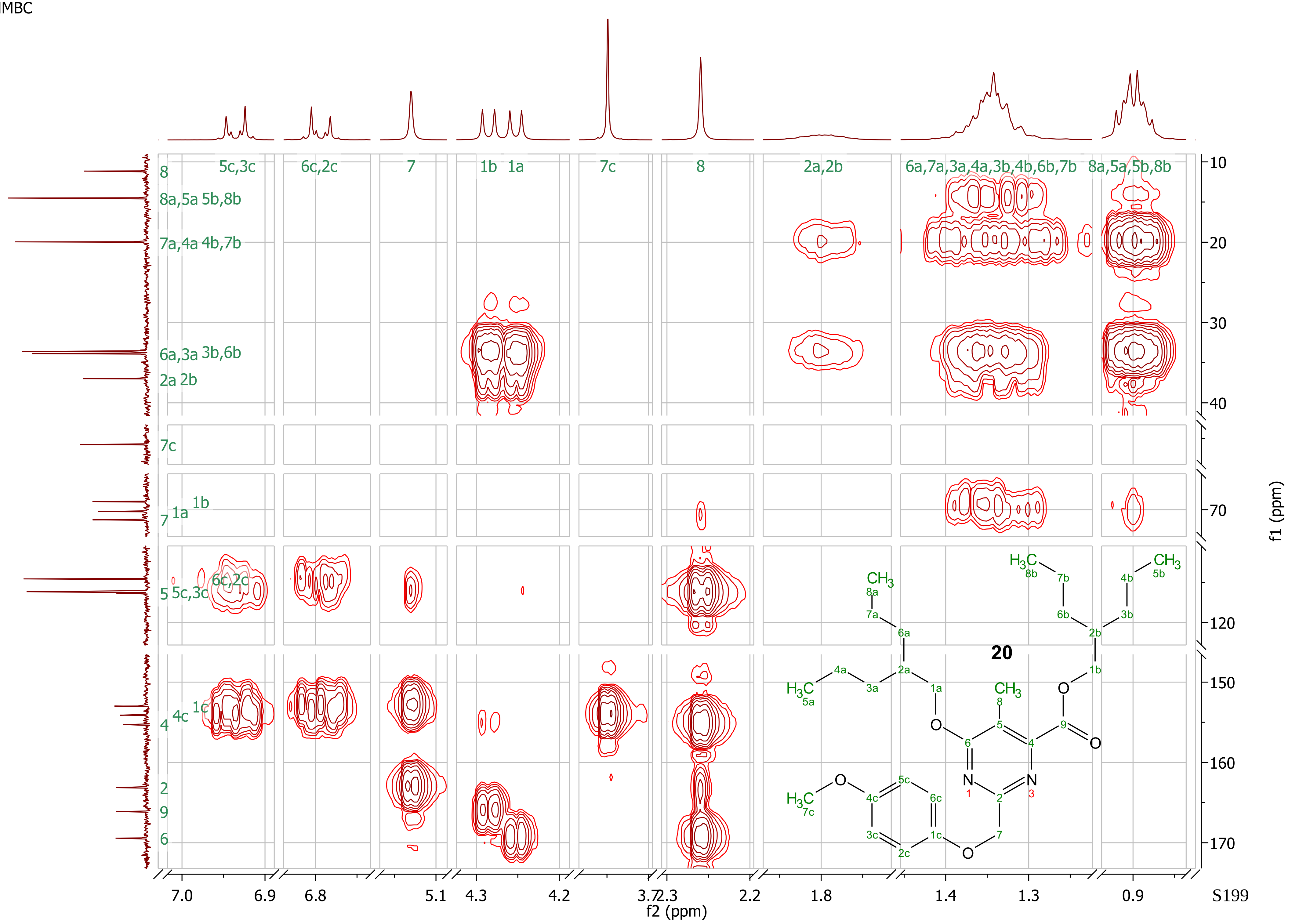
5c,3c
6c,2c
7
1b 1a
7c
8
2a,2b
6a,7a,3a,4a,3b,4b,6b,7b
8a,5a,5b,8b
8
8a,5a 5b,8b
7a,4a 4b,7b
6a,3a 3b,6b
2a 2b
7c
7 1a 1b
5 5c,3c
6c,2c
4 4c 1c
2
9
6
20
f1 (ppm)
f2 (ppm)

^{1}H NMR (400 MHz, $CDCl_3$) δ 7.72 (s, 1H), 7.68 (s, 1H), 7.67 – 7.48 (m, 4H), 7.51 (t, *J* = 7.7 Hz, 1H), 7.45 (t, *J* = 7.7 Hz, 1H), 7.00 – 6.85 (m, 2H), 6.85 – 6.70 (m, 2H), 5.47 (s, 2H), 5.46 (s, 2H), 5.17 (s, 2H), 3.75 (s, 3H), 2.31 (s, 3H).

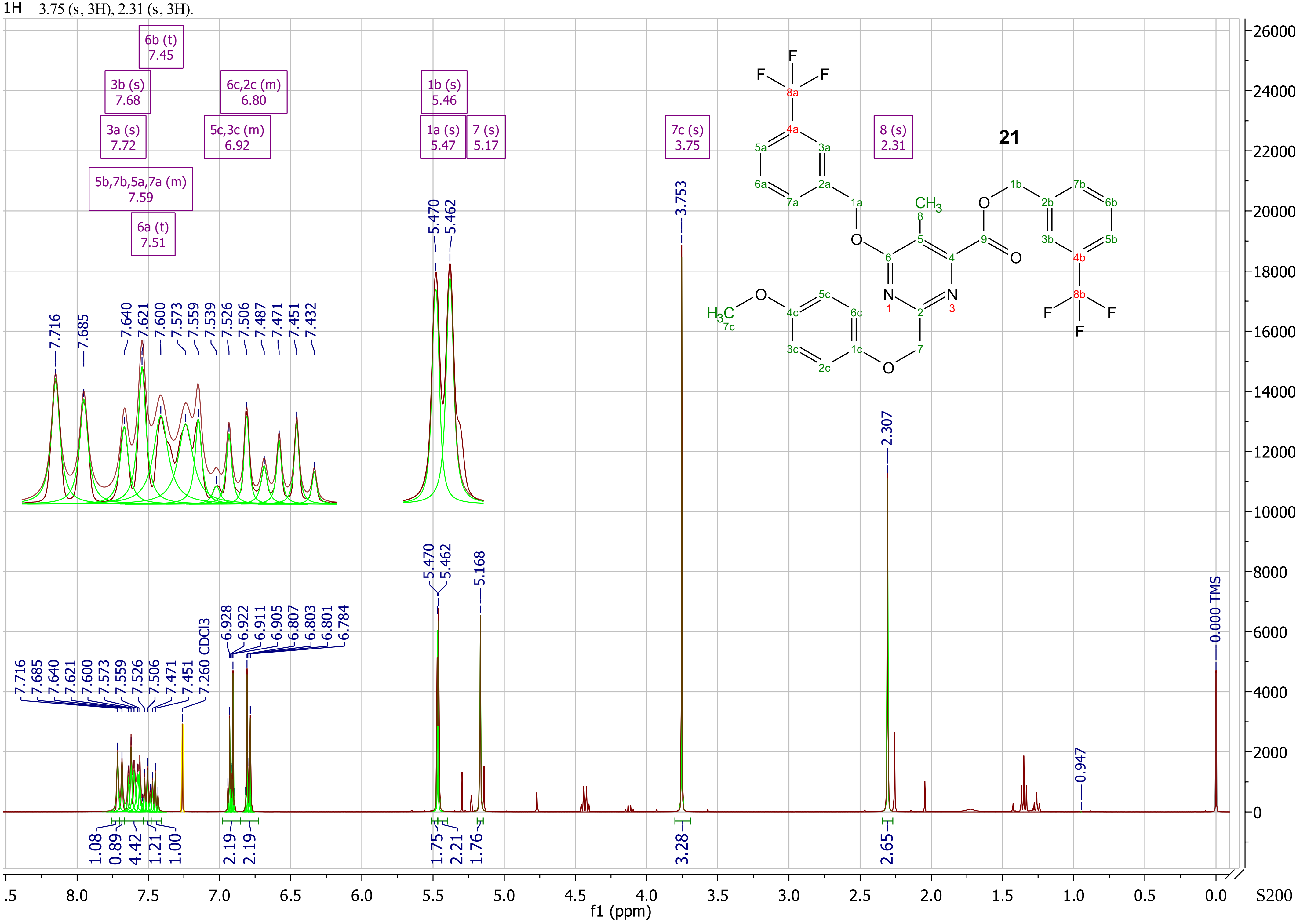

13C ^{13}C NMR (101 MHz, $CDCl_3$) δ 168.7, 165.1, 163.3, 154.7, 154.2, 152.8, 137.0, 136.3, 131.7 (2C), 129.4, 129.2, 125.6 – 125.3 (m, 2C), 125.3 – 125.0 (m, 2C), 117.5, 116.0 (sym, 2C), 114.7 (sym, 2C), 70.9, 68.1, 66.8, 55.8, 11.1.

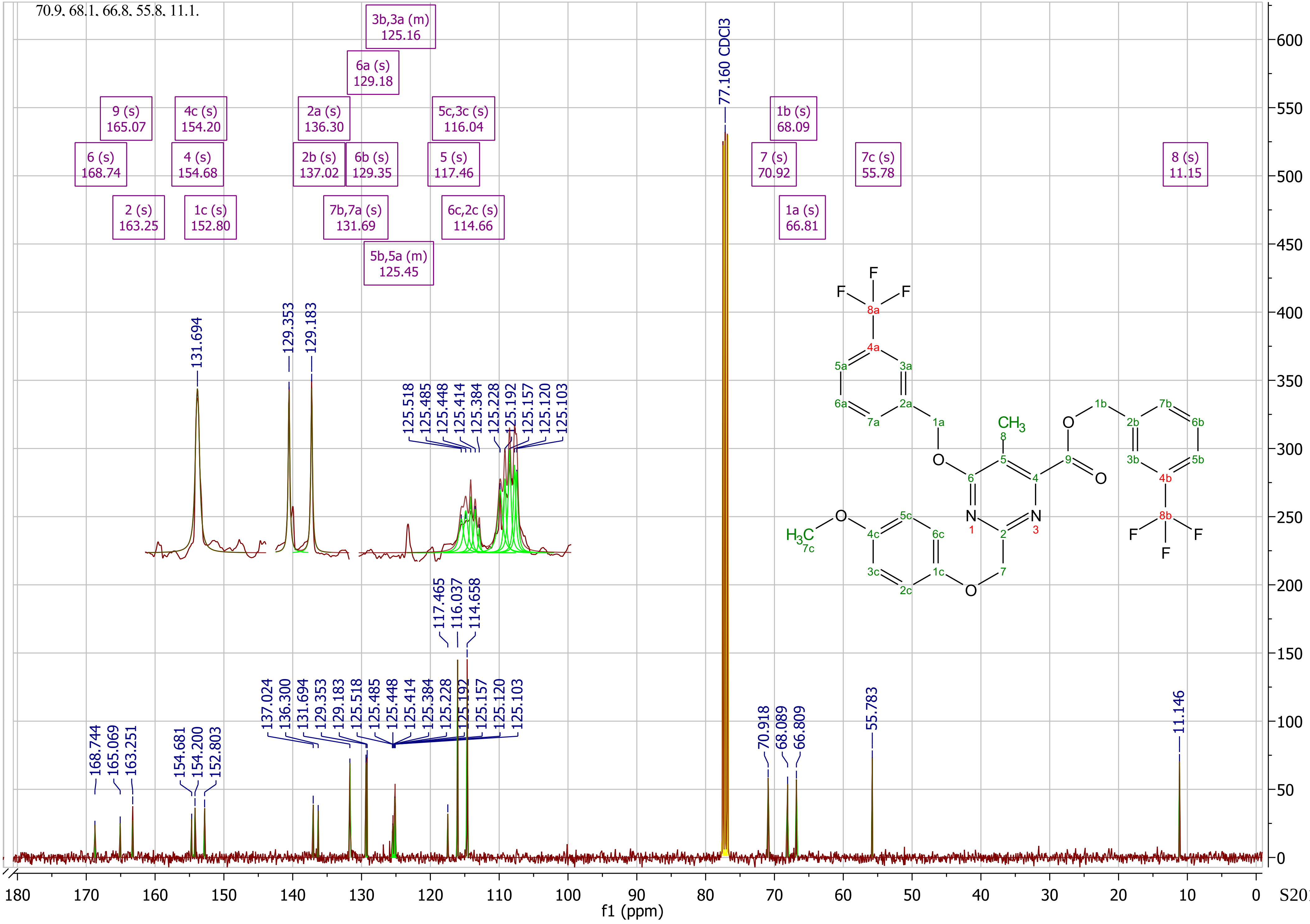

13C HSQC

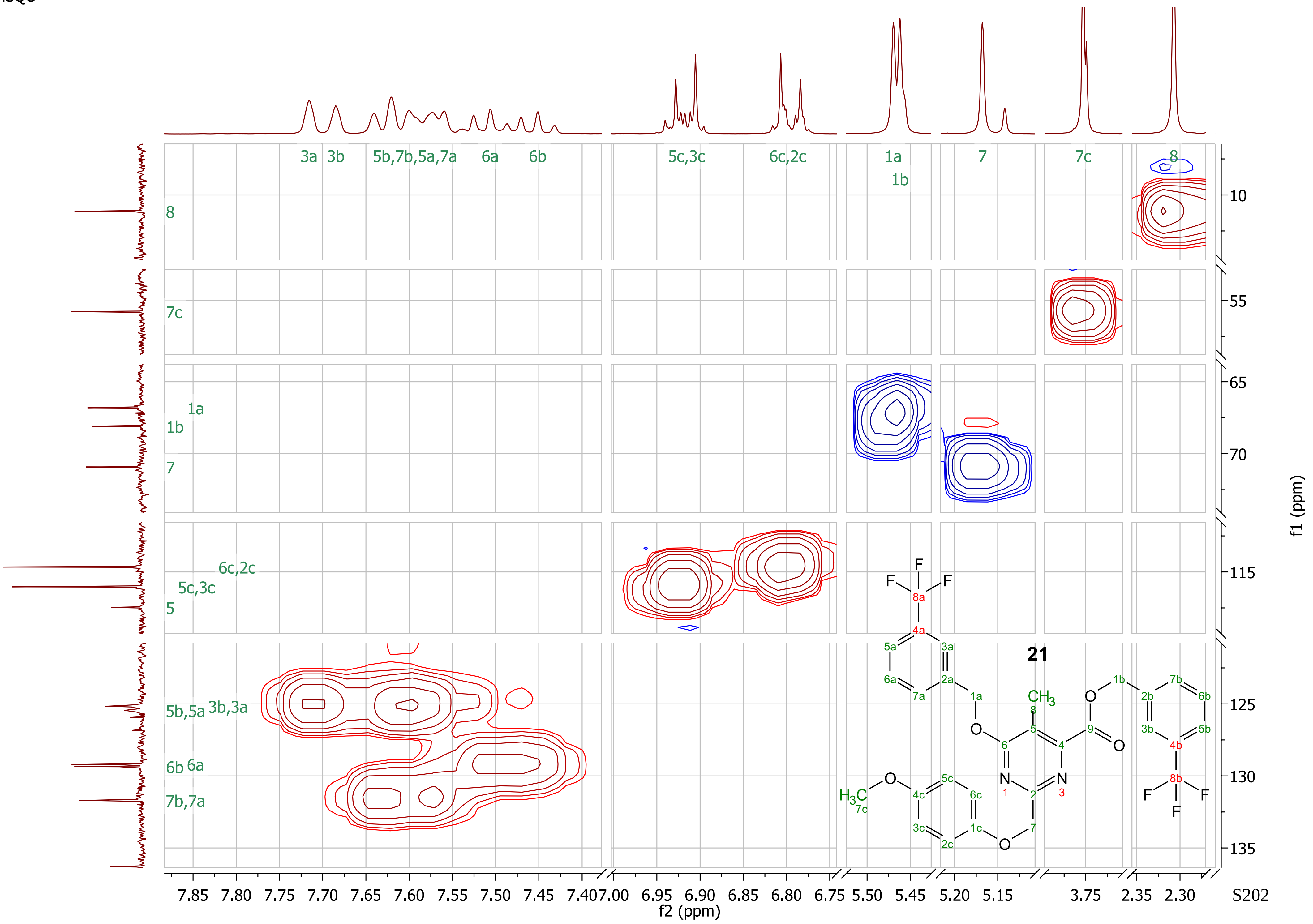

3a 3b
5b,7b,5a,7a
6a
6b
5c,3c
6c,2c
1a
1b
7
7c
8
5
5b,5a 3b,3a
6b 6a
7b,7a
21
f2 (ppm)
f1 (ppm)

13C HMBC

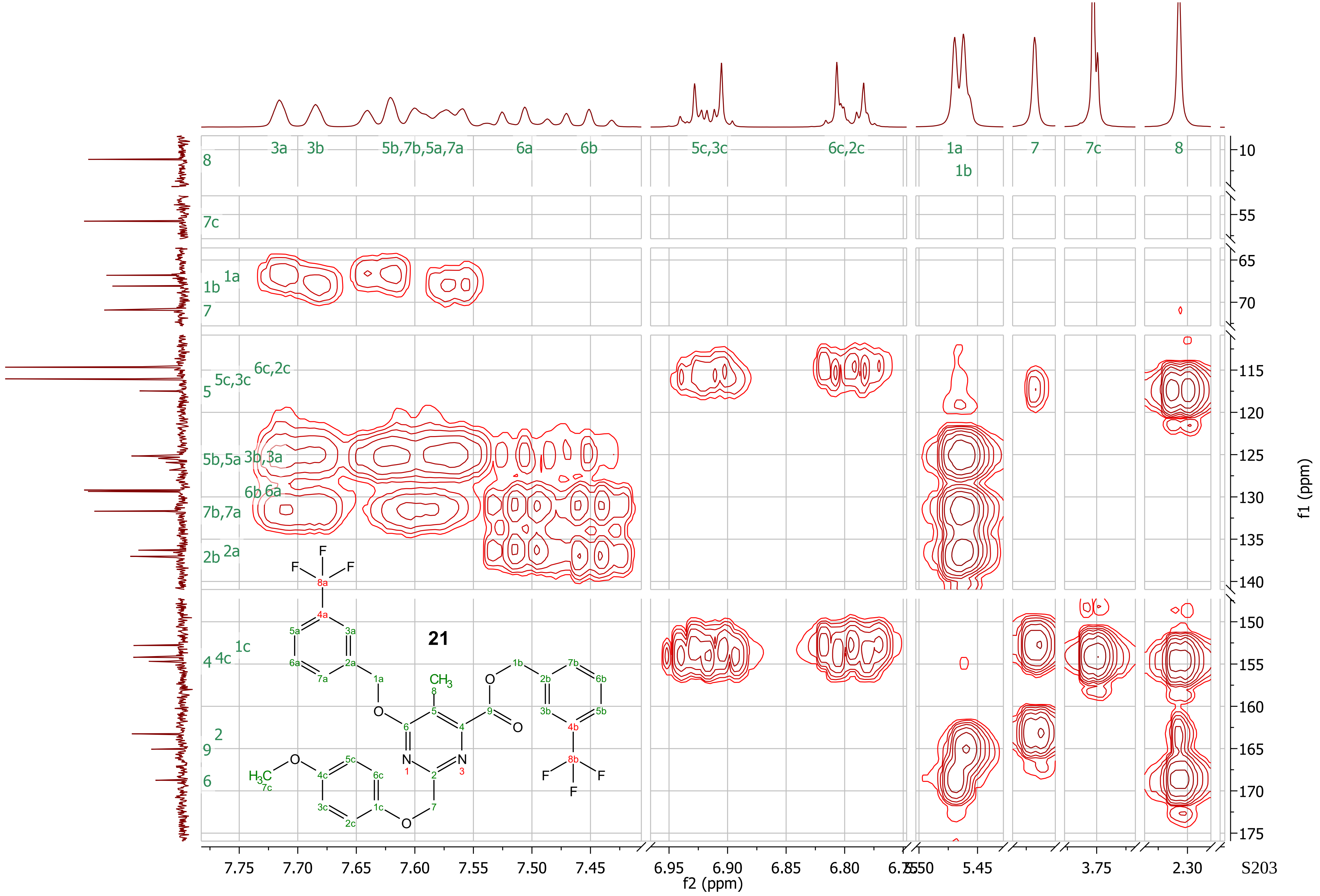

3a
3b
5b,7b,5a,7a
6a
6b
5c,3c
6c,2c
1a
1b
7
7c
8
5c,3c
6c,2c
5
5b,5a
3b,3a
6b
6a
7b,7a
2b
2a
4
4c
1c
2
9
6
21
CH3
H3C
7.75
7.70
7.65
7.60
7.55
7.50
7.45
6.95
6.90
6.85
6.80
5.50
5.45
3.75
2.30
f2 (ppm)
f1 (ppm)
10
55
65
70
115
120
125
130
135
140
150
155
160
165
170
175

1H ^{1}H NMR (400 MHz, $CDCl_3$) δ 7.69 (s, 1H), 7.64 – 7.51 (m, 2H), 7.45 (t, J = 7.7 Hz, 1H), 6.97 – 6.86 (m, 2H), 6.85 – 6.75 (m, 2H), 5.47 (s, 2H), 5.17 (s, 2H), 3.98 (s, 3H), 3.76 (s, 3H), 2.36 (s, 3H).

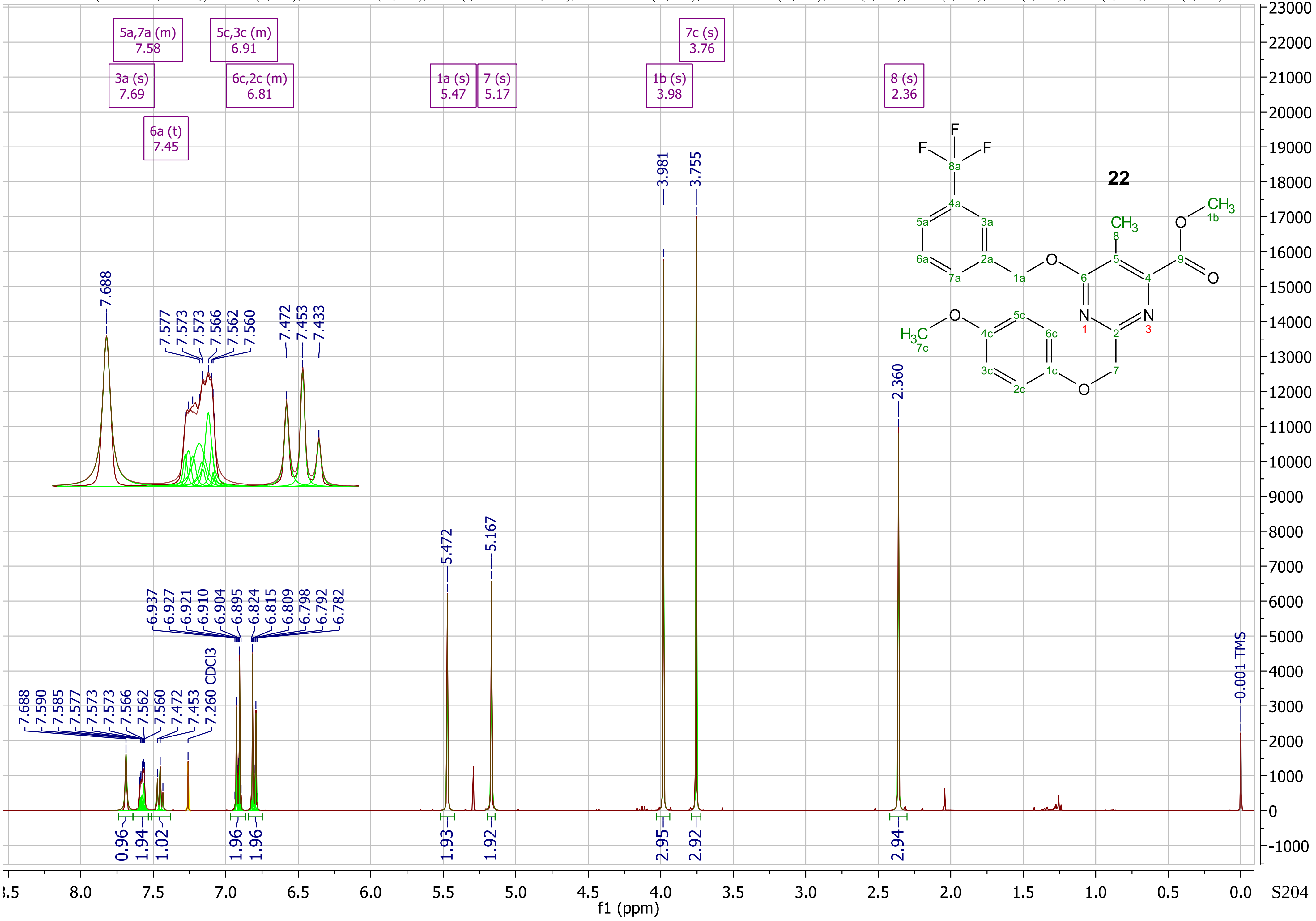

^{13}C NMR (101 MHz, $CDCl_3$) δ 168.8, 165.7, 163.1, 154.7, 154.2, 152.8, 137.1, 131.7 (app q, J = 1.2 Hz), 131.1 (d, J = 32.4 Hz), 129.2, 126.8 (d, J = 271.1 Hz), 125.2 (q, J = 3.9 Hz), 125.1 (q, J = 3.9 Hz), 117.7, 116.0 (sym, 2C), 114.7 (sym, 2C), 71.0, 68.1, 55.8, 53.2, 11.2.

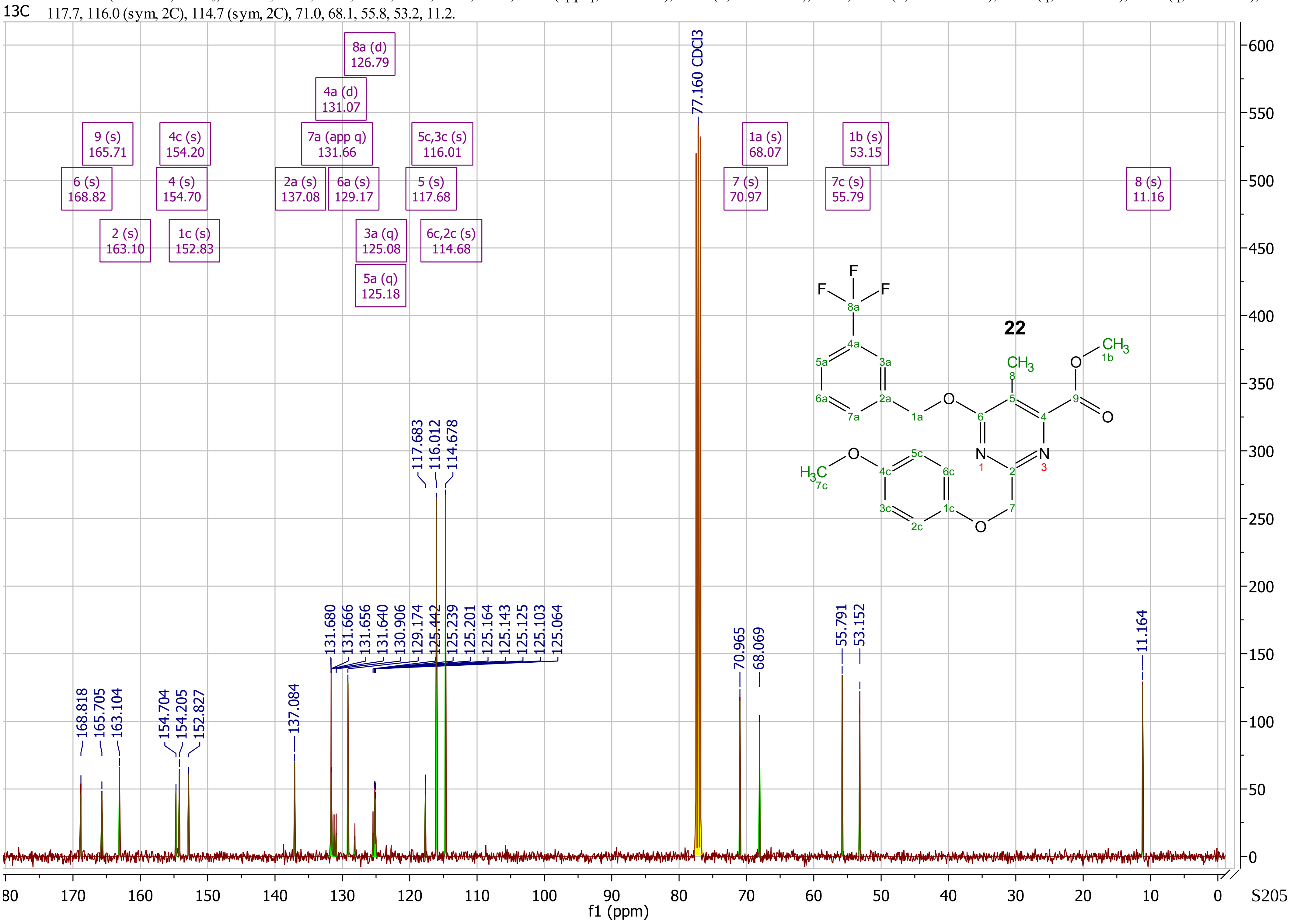

13C [123.5 — 132.5 ppm]

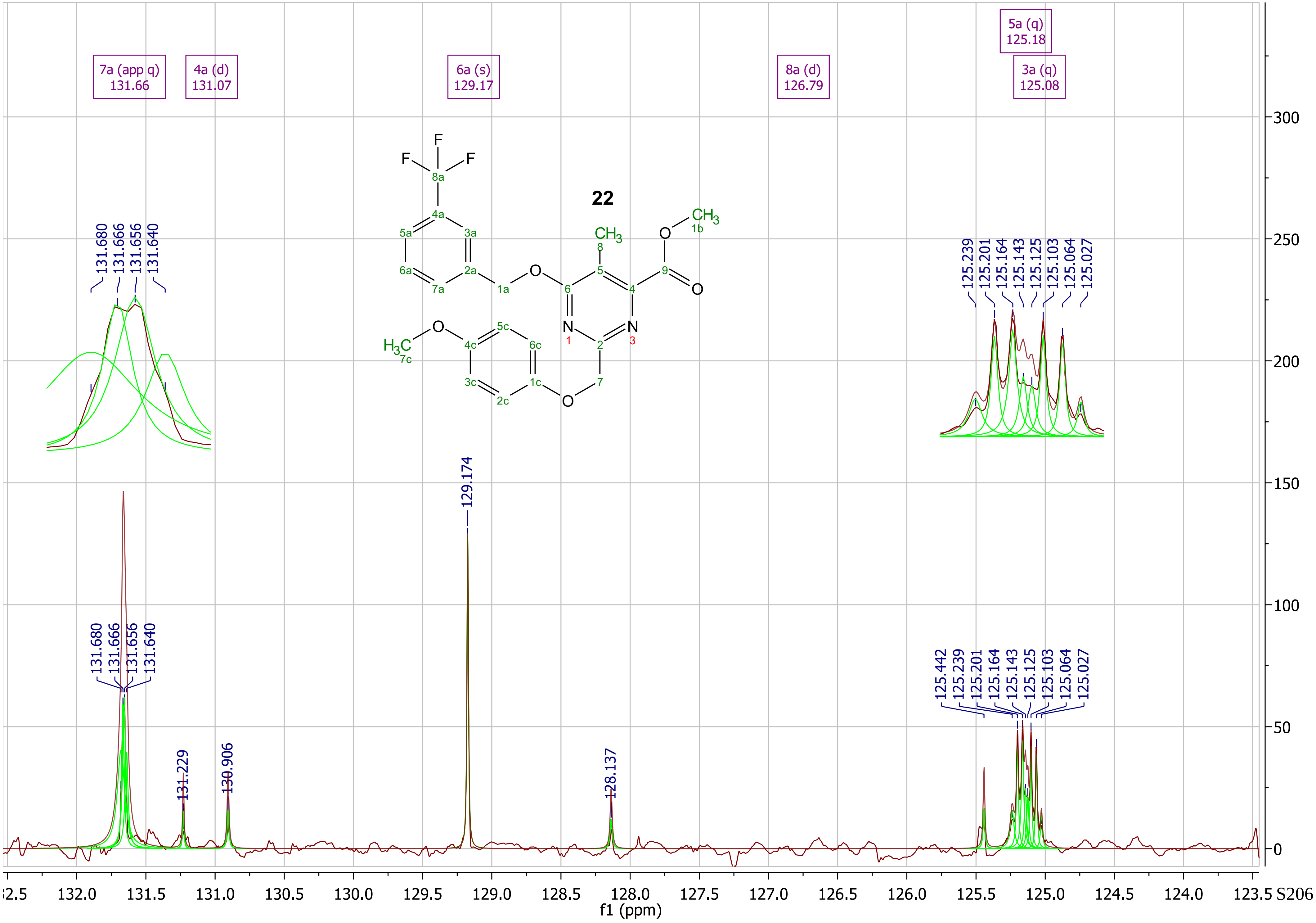

13C HSQC

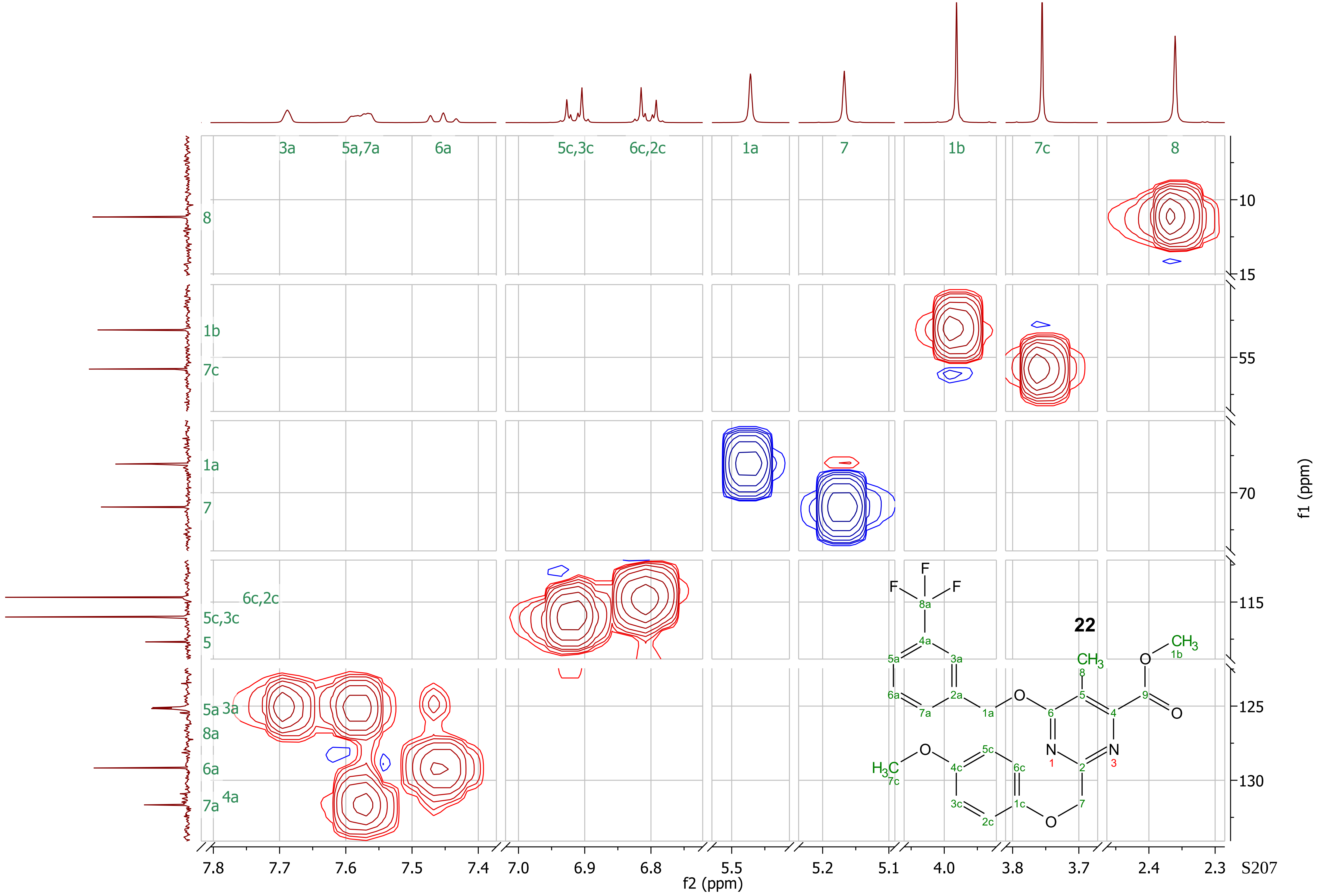

13C HMBC

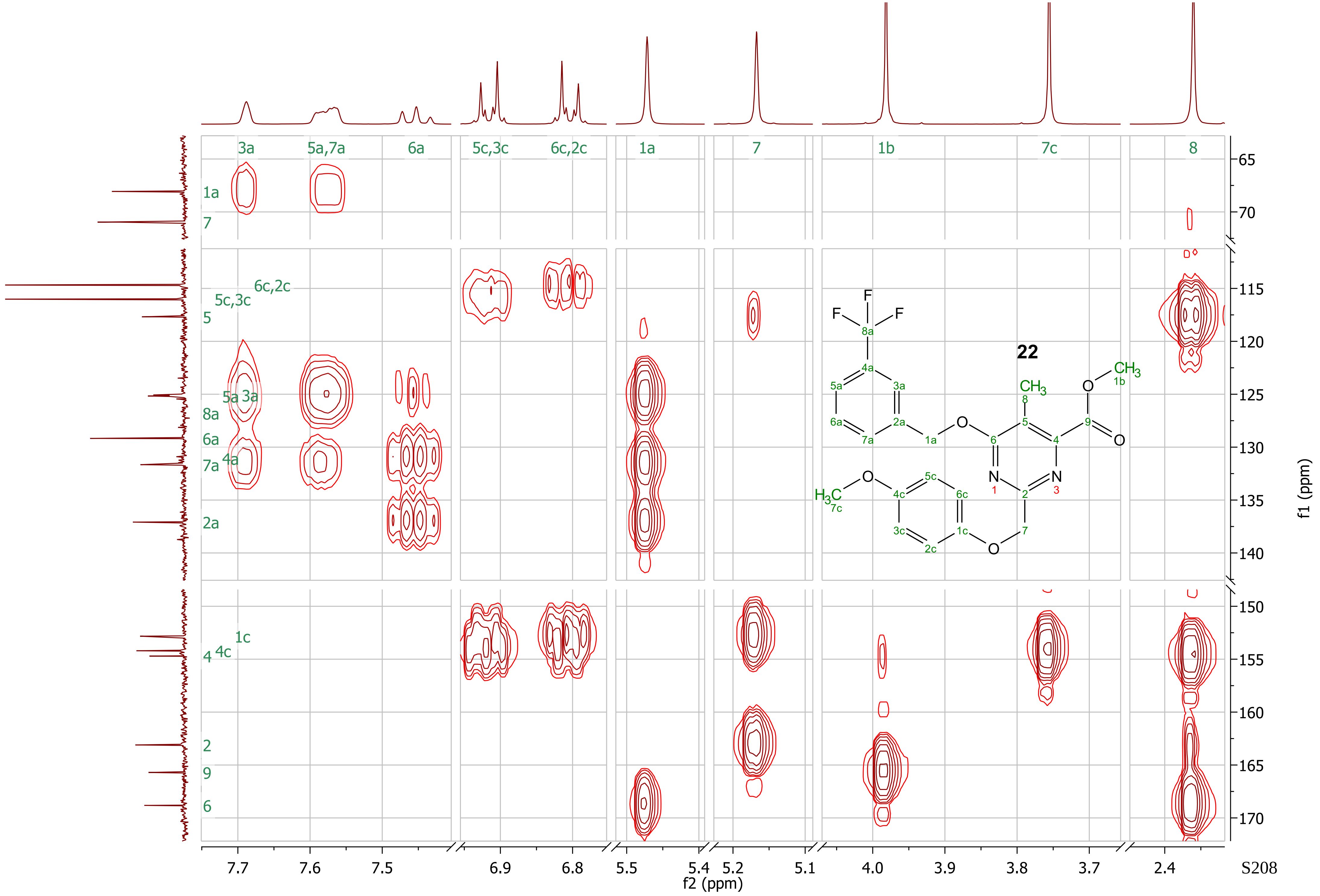

3a
5a,7a
6a
5c,3c
6c,2c
1a
7
1b
7c
8
1a
7
6c,2c
5c,3c
5
5a 3a
8a
6a
7a 4a
2a
1c
4 4c
2
9
6
22
f1 (ppm)
f2 (ppm)

^{1}H NMR (400 MHz, $CDCl_3$) δ 7.69 (s, 1H), 7.61 – 7.52 (m, 2H), 7.45 (t, J = 7.7 Hz, 1H), 6.99 – 6.88 (m, 2H), 6.86 – 6.75 (m, 2H), 5.46 (s, 2H), 5.16 (s, 2H), 5.13 (app quint, J = 6.5 Hz, 1H), 3.76 (s, 3H), 2.30 (s, 3H), 1.81 – 1.59 (m, 4H), 1.45 – 1.23 (m, 4H), 0.97 (t, J = 7.4 Hz, 3H), 0.91 (app t, J = 7.1 Hz, 3H).

1H

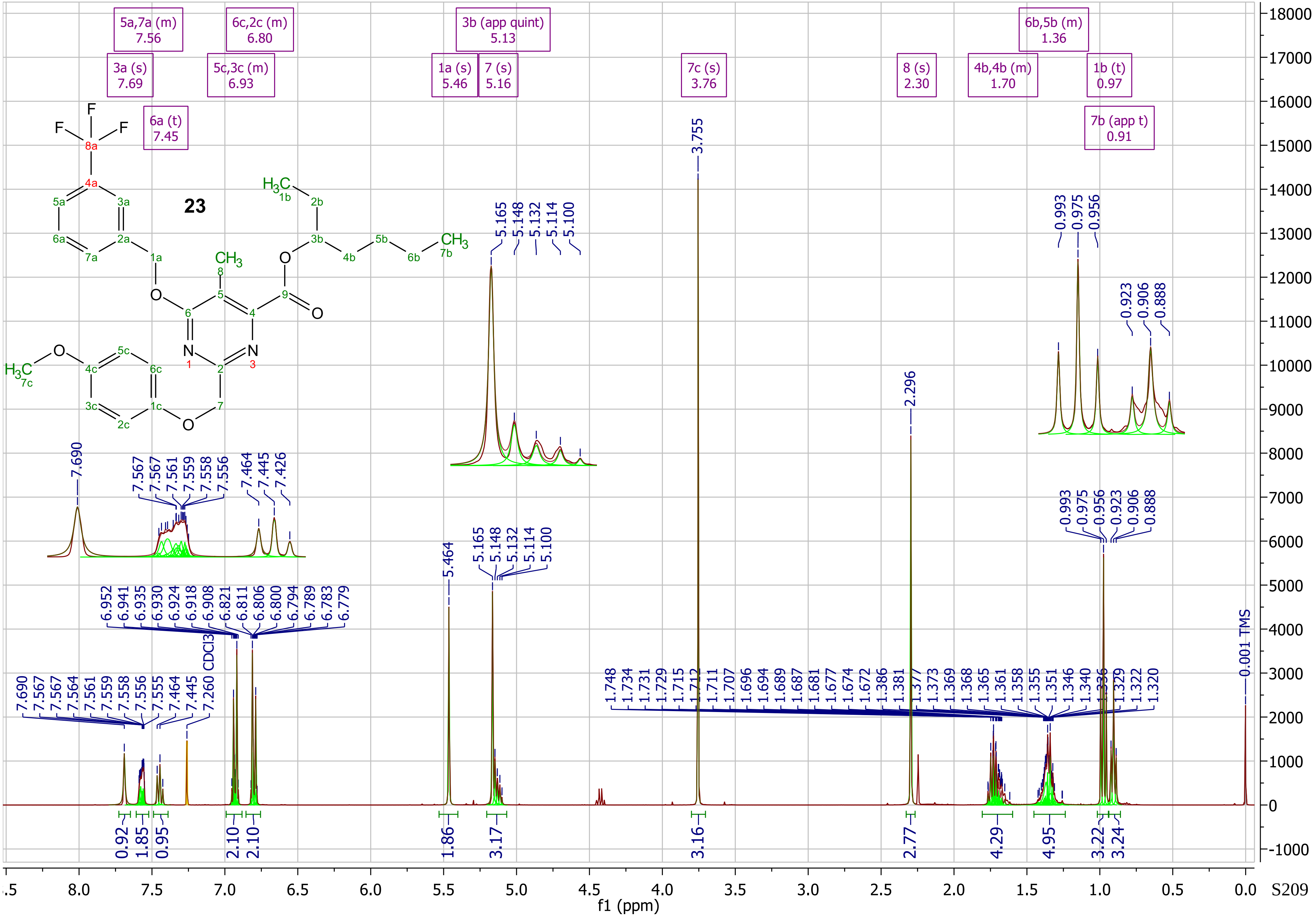

^{13}C NMR (101 MHz, $CDCl_3$) δ 168.6, 165.6, 163.2, 156.6, 154.1, 152.9, 137.2, 131.7 (app q, *J* = 1.3, 0.9 Hz), 129.1, 125.2 – 125.0 (m, 2C), 116.1 (sym, 2C), 115.8, 114.6 (sym, 2C), 78.3, 71.0, 67.9, 55.8, 33.3, 27.6, 27.0, 22.7, 14.1, 11.1, 9.8.

13C

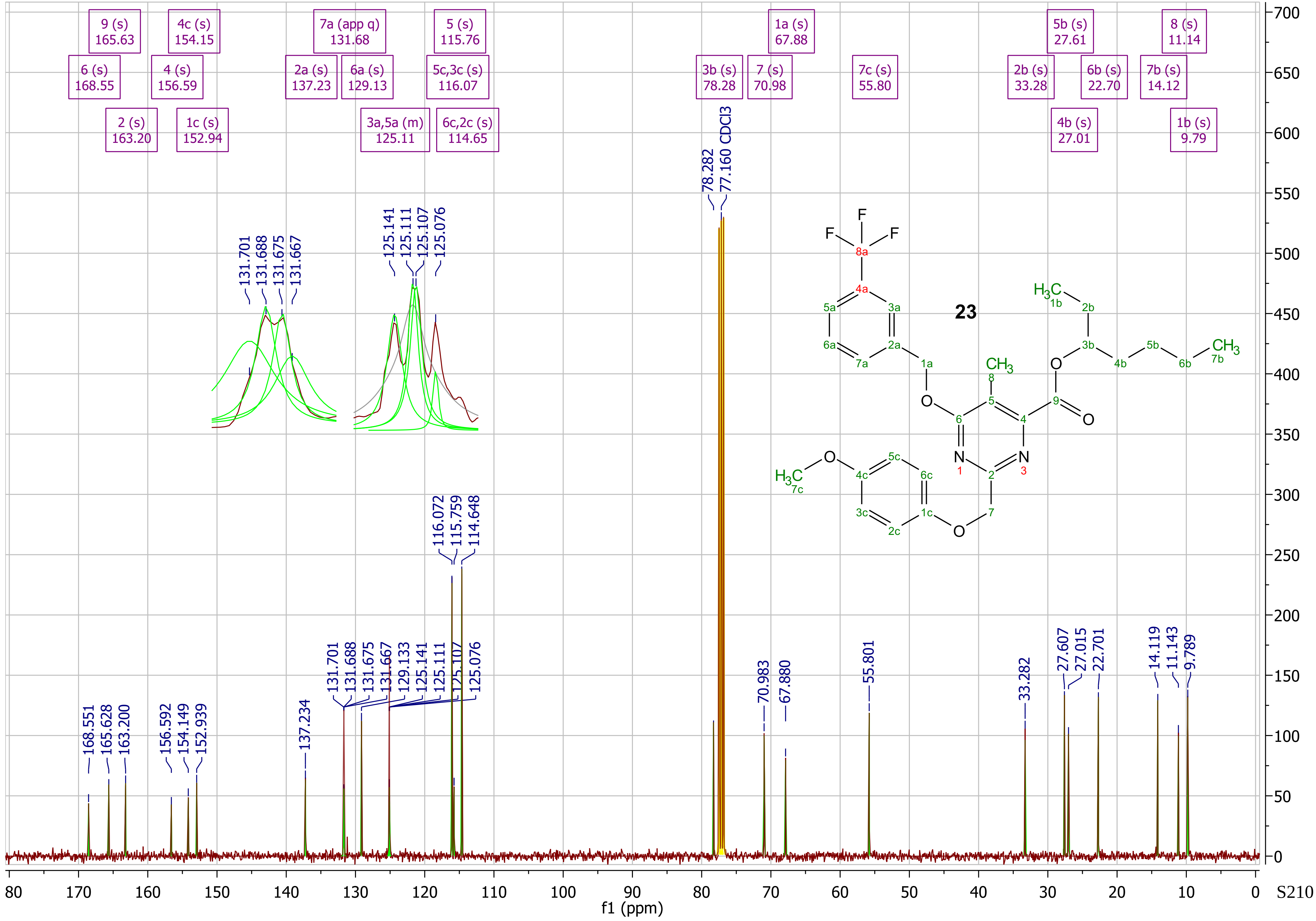

13C HSQC

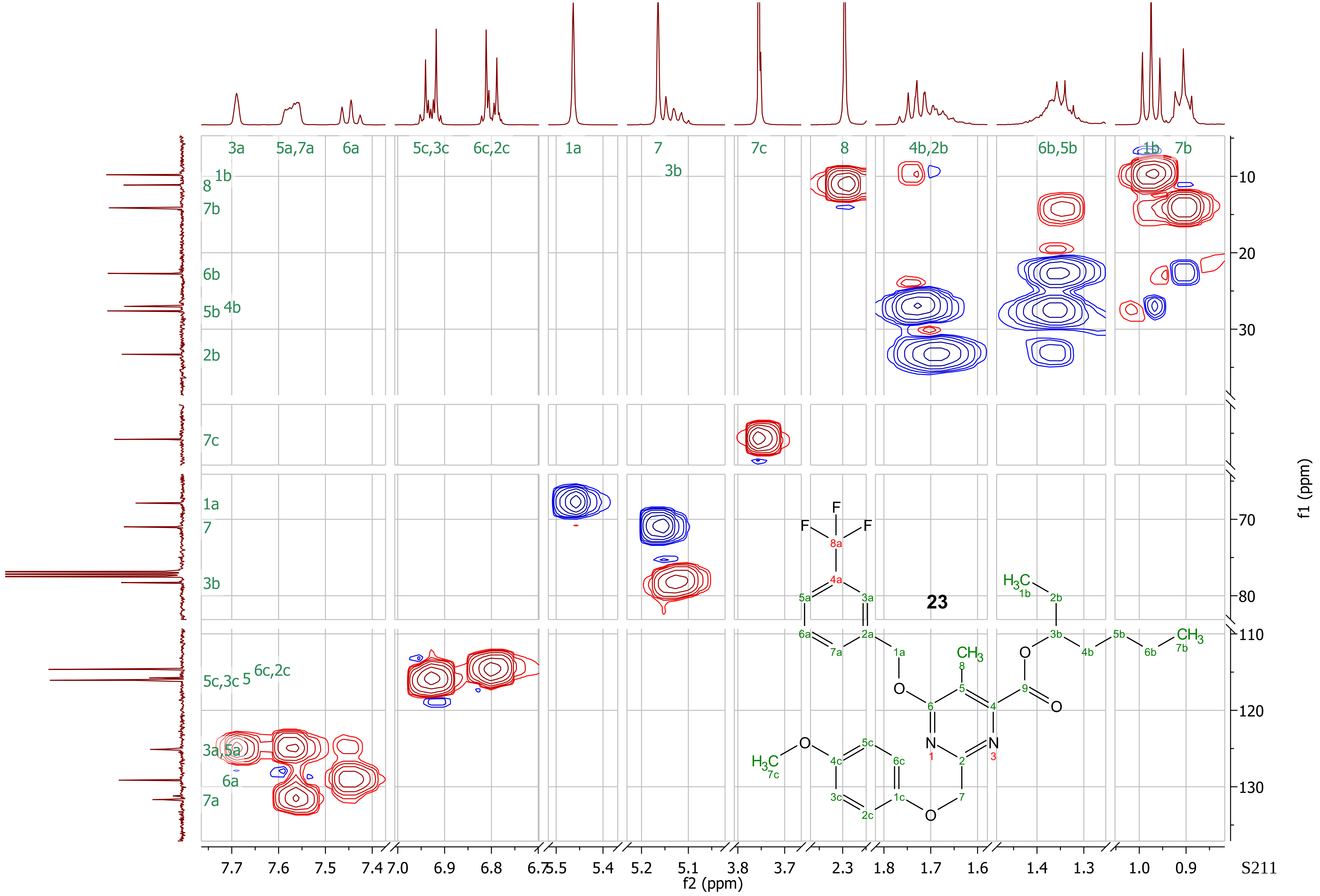
3a
5a,7a
6a
5c,3c
6c,2c
1a
7
3b
7c
8
4b,2b
6b,5b
1b
7b
8
1b
7b
6b
5b
4b
2b
7c
1a
7
3b
5c,3c
5
6c,2c
3a,5a
6a
7a
23
f2 (ppm)
f1 (ppm)

13C HMBC

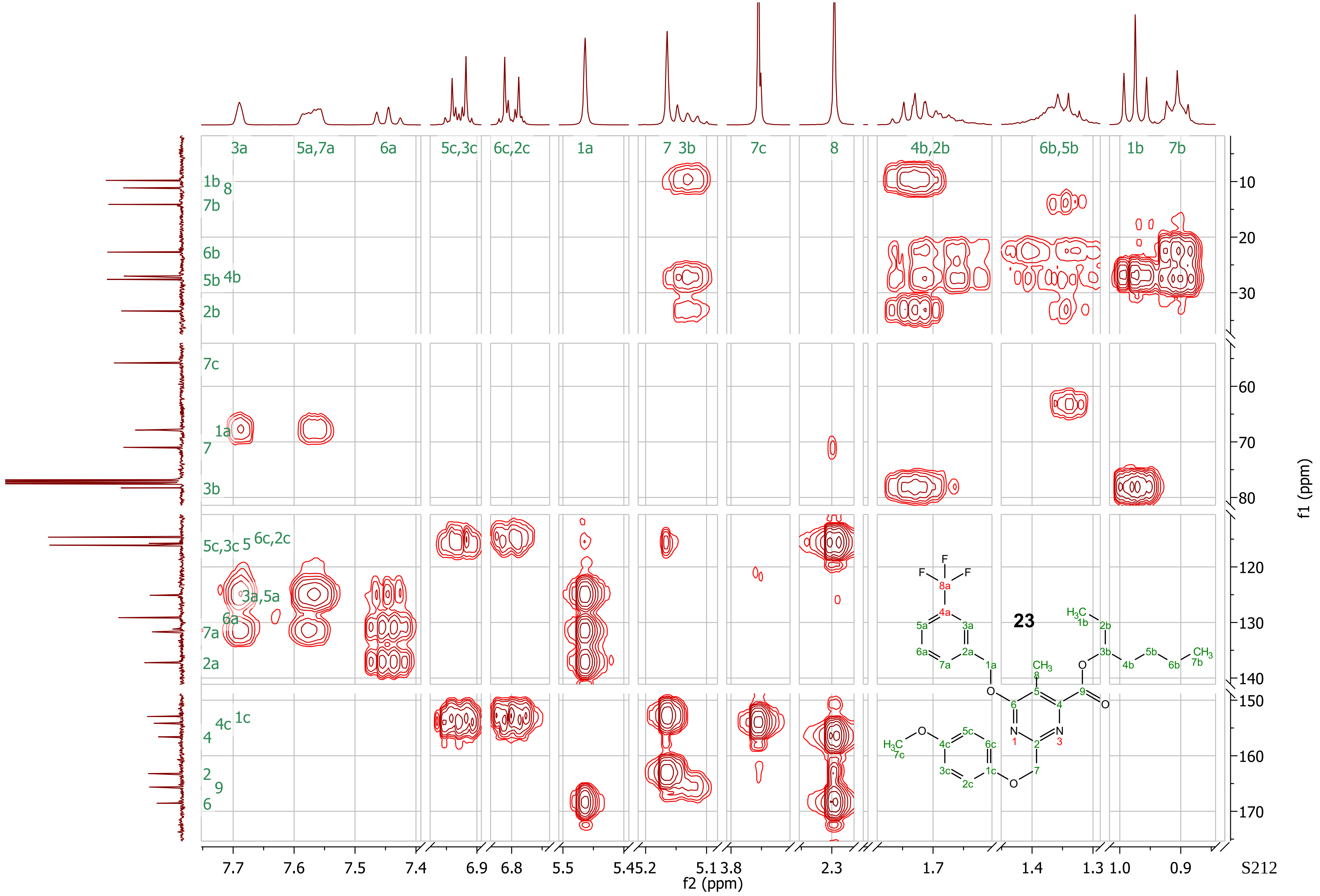

1H ^{1}H NMR (400 MHz, $CDCl_3$) δ 6.98 – 6.89 (m, 2H), 6.84 – 6.74 (m, 2H), 5.13 (s, 2H), 5.12 (app quint, J = 6.1 Hz, 1H), 4.34 (t, J = 6.6 Hz, 2H), 3.75 (s, 3H), 2.24 (s, 3H), 1.79 – 1.59 (m, 6H), 1.45 – 1.20 (m, 12H), 0.97 (t, J = 7.4 Hz, 3H), 0.90 (app t, J = 6.6 Hz, 3H), 0.89 (app t, J = 6.7 Hz, 3H).

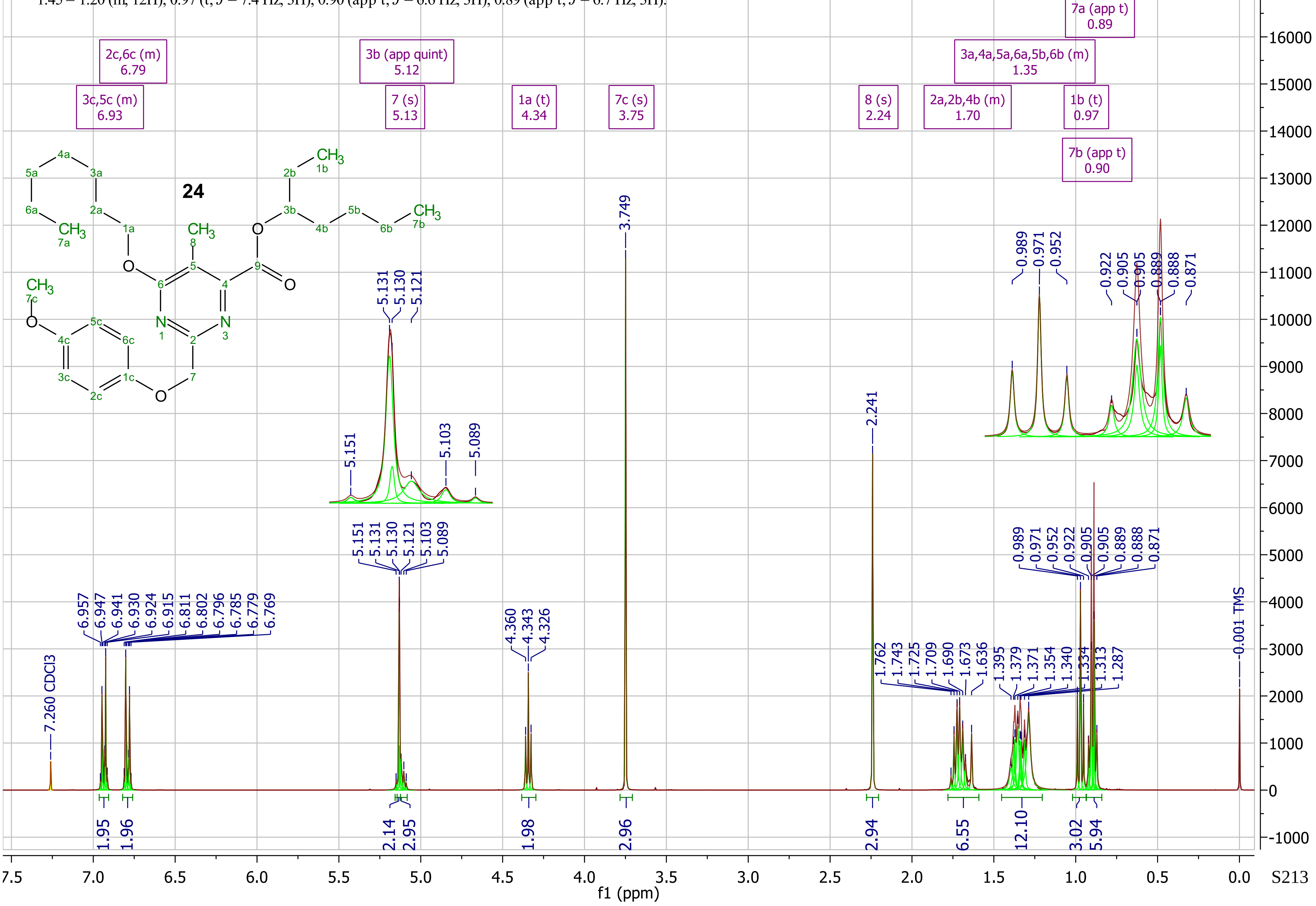

13C ^{13}C NMR (101 MHz, $CDCl_3$) δ 169.2, 165.9, 163.1, 156.0, 154.1, 153.0, 116.2 (sym, 2C), 115.7, 114.6 (sym, 2C), 78.1, 71.2, 67.6, 55.8, 33.3, 31.9, 29.1, 28.8, 27.6, 27.0, 26.0, 22.74, 22.72, 14.2, 14.1, 11.1, 9.8.

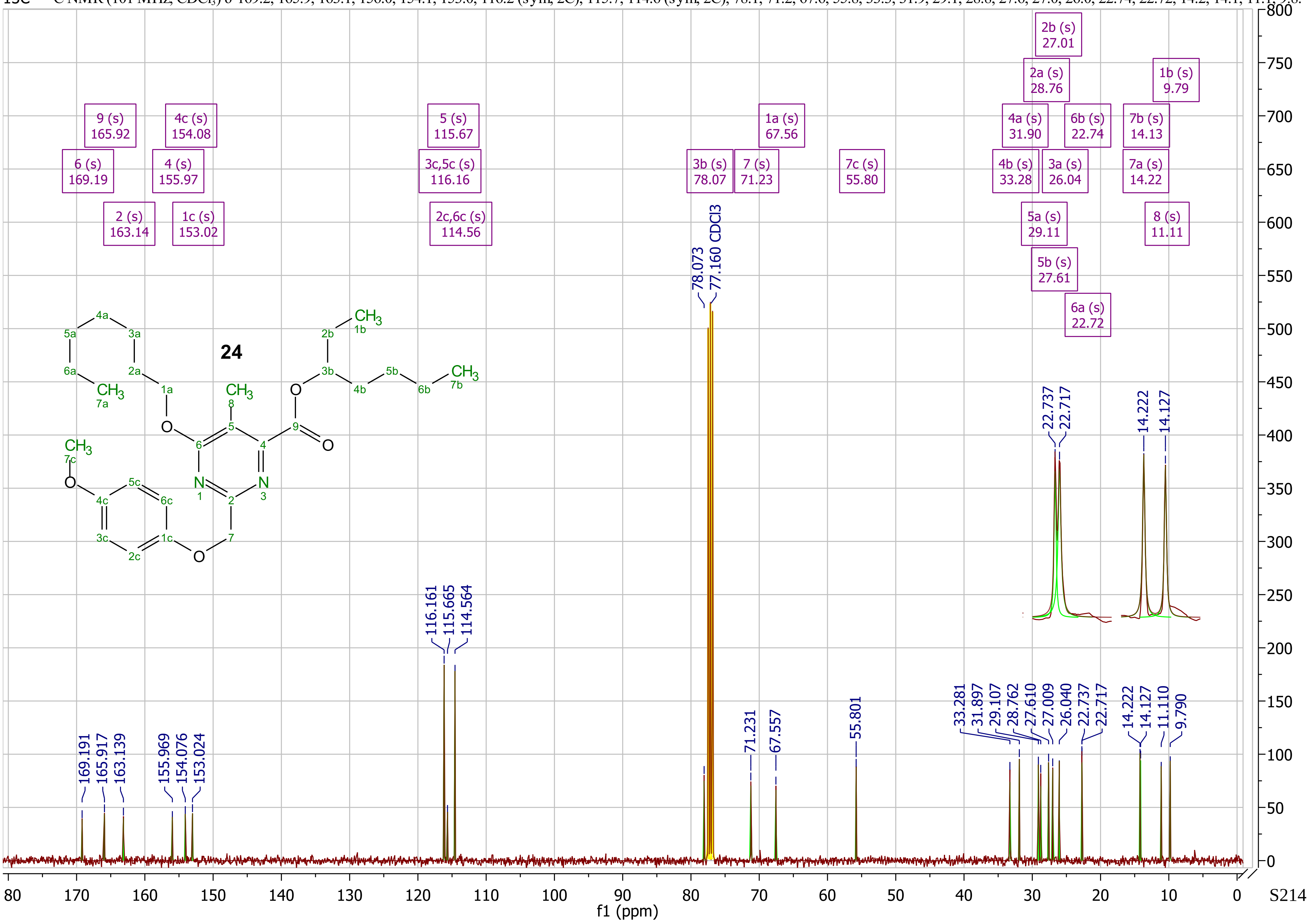

13C HSQC

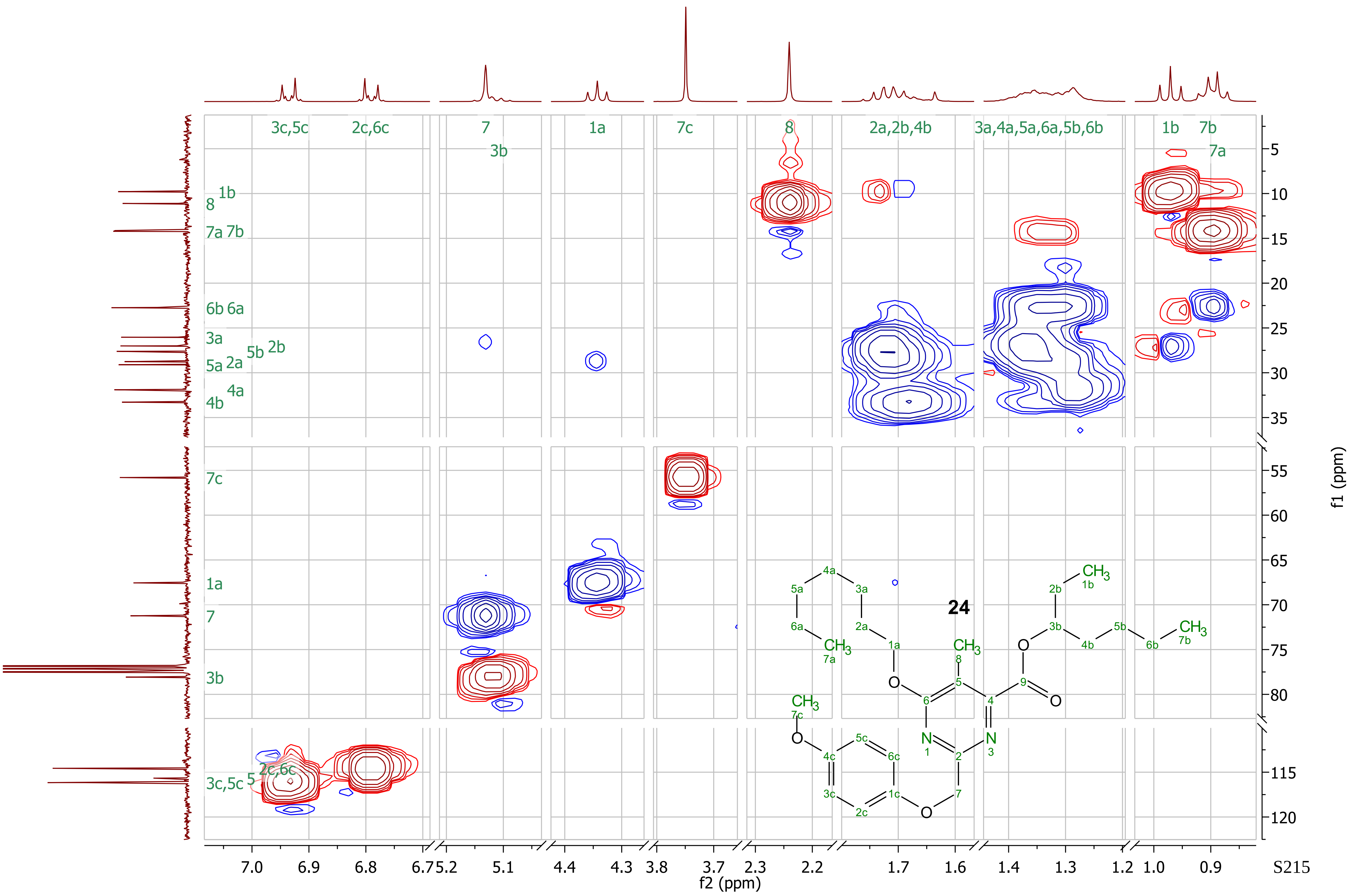
3c,5c
2c,6c
7
3b
1a
7c
8
2a,2b,4b
3a,4a,5a,6a,5b,6b
1b
7b
7a
8 1b
7a 7b
6b 6a
3a
5b 2b
5a 2a
4a
4b
7c
1a
7
3b
3c,5c 5
2c,6c
24
f1 (ppm)
f2 (ppm)

13C HMBC

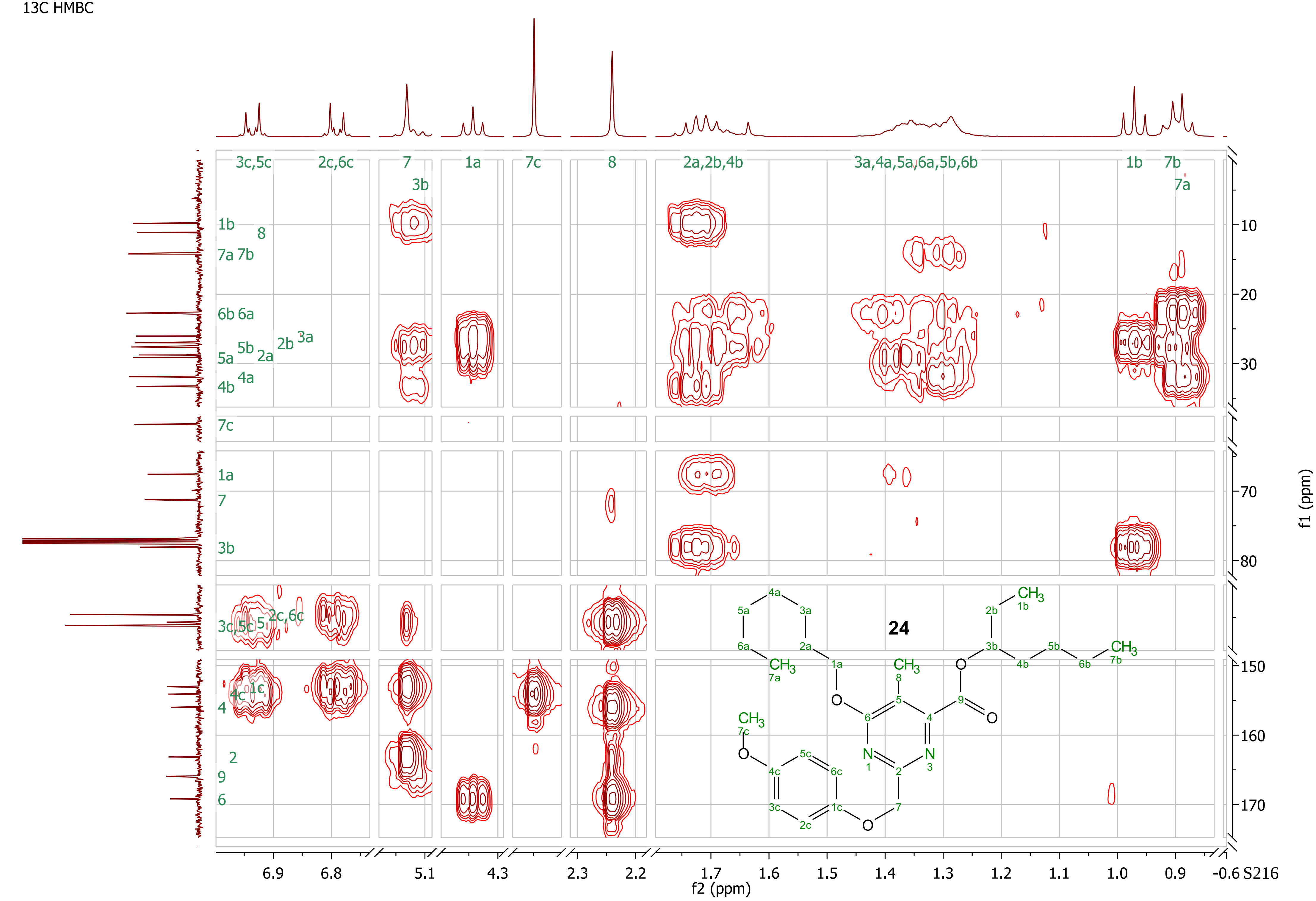

S216

15N HMBC

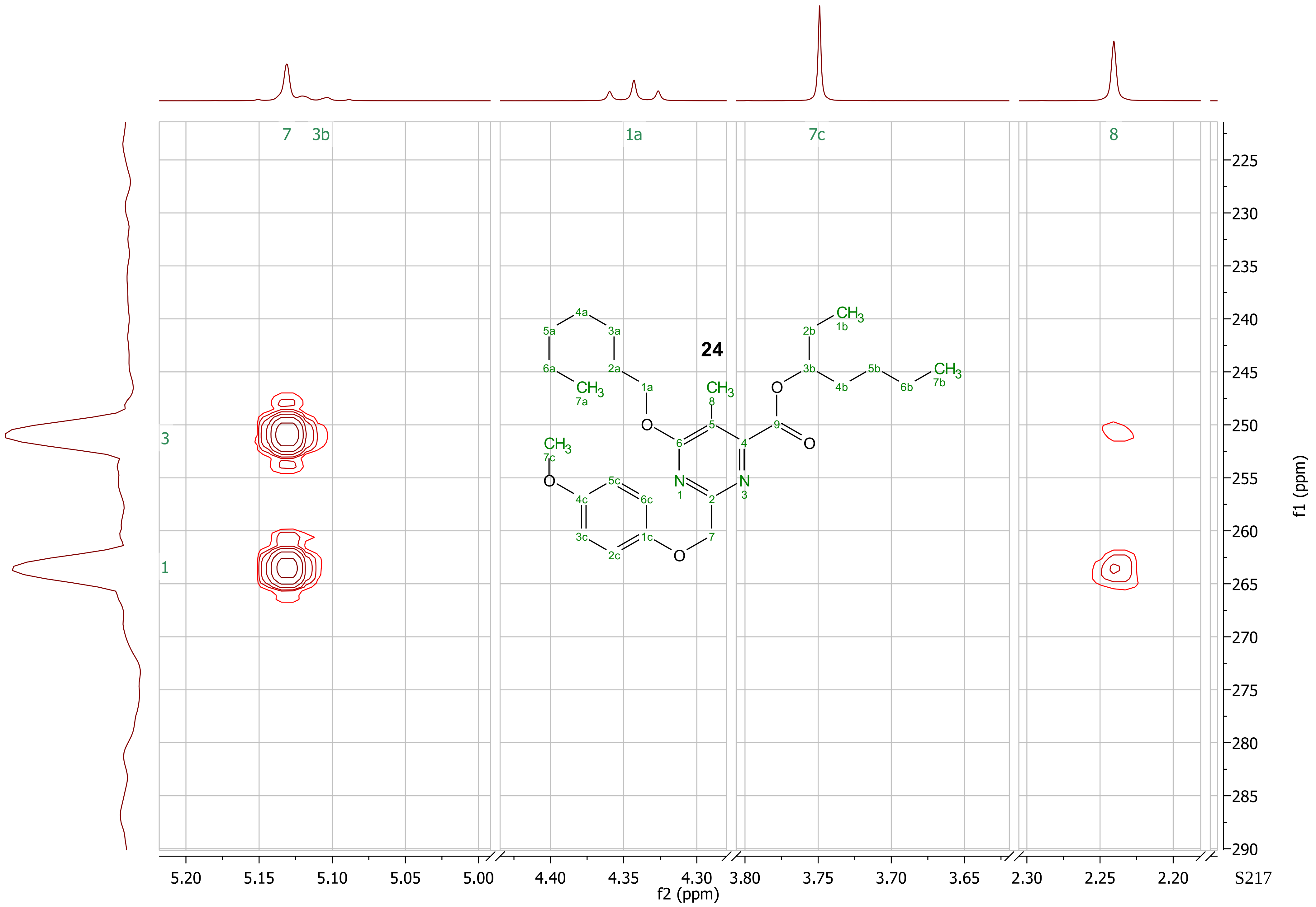

15N HMBC f1 Projection ^{15}N NMR (41 MHz, $CDCl_3$) δ263.35, 251.21.

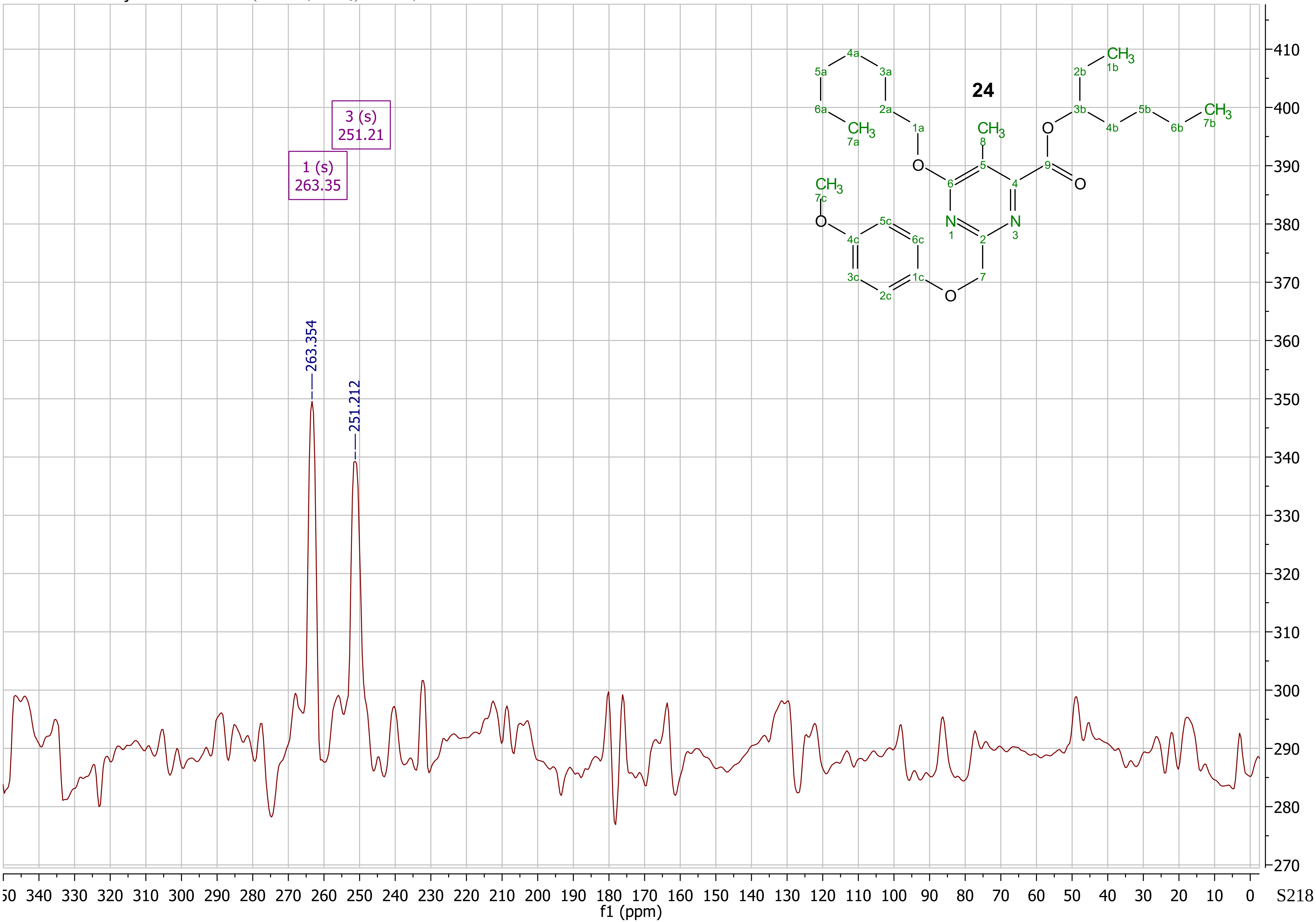

^{1}H NMR (400 MHz, $CDCl_3$) δ 7.72 (s, 1H), 7.65 – 7.58 (m, 2H), 7.50 (t, *J* = 7.7 Hz, 1H), 6.96 – 6.89 (m, 2H), 6.83 – 6.75 (m, 2H), 5.46 (s, 2H), 5.14 (s, 2H), 4.36 (t, *J* = 6.6 Hz, 2H), 3.75 (s, 3H), 2.26 (s, 3H), 1.72 (quint, *J* = 6.7 Hz, 2H), 1.45 – 1.16 (m, 8H), 0.89 (app t, *J* = 7.0 Hz, 3H).

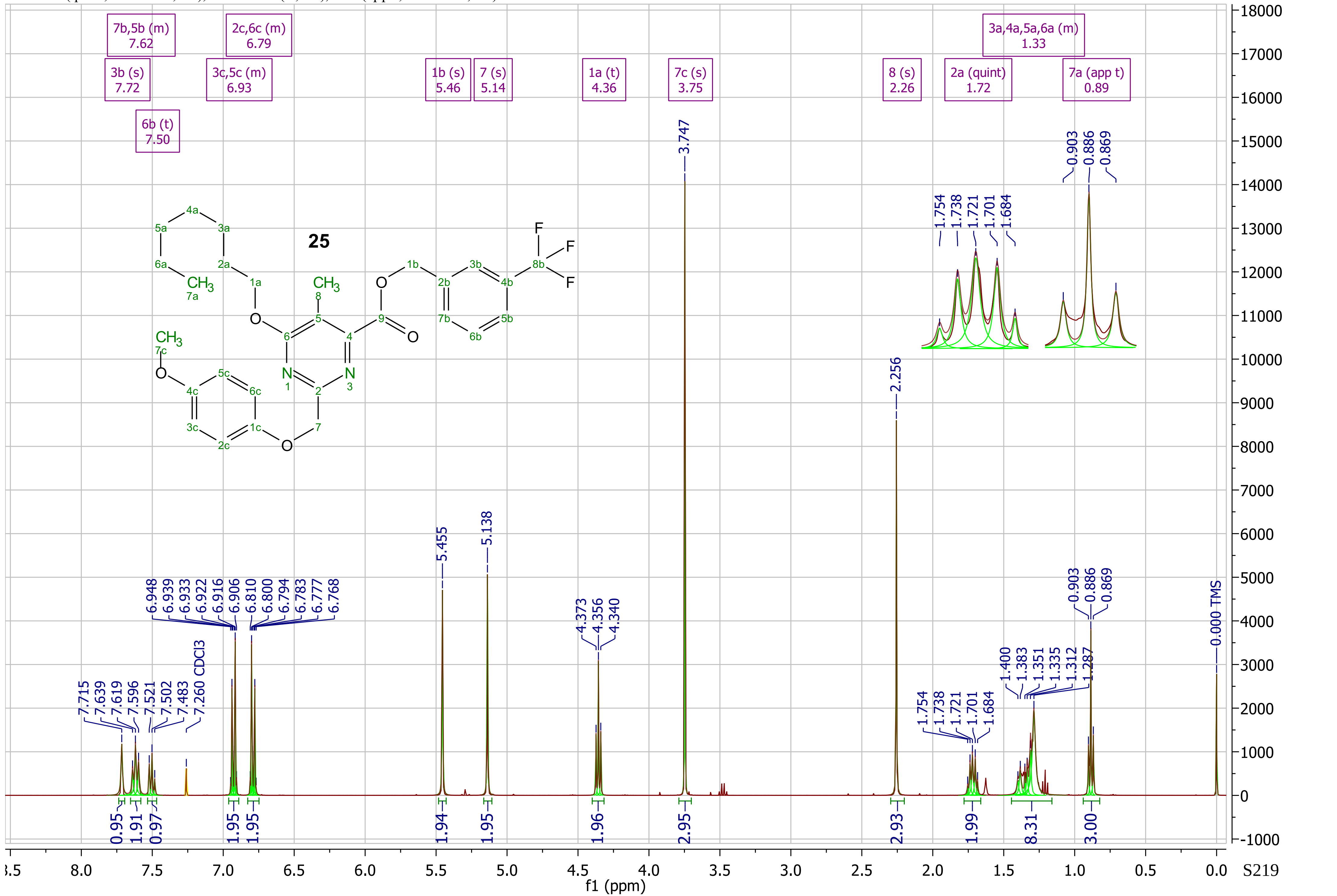

^{13}C NMR (101 MHz, $CDCl_3$) δ 169.4, 165.3, 163.2, 154.2, 154.1, 152.9, 136.4, 131.7 (app q, J = 1.5 Hz), 131.2 (q, J = 32.6 Hz), 129.3, 125.4 (q, J = 3.6 Hz), 125.1 (q, J = 3.7 Hz), 124.3 (q, J = 272.5 Hz), 117.4, 116.1 (sym, 2C), 114.6 (sym, 2C), 71.2, 67.8, 66.7, 55.8, 31.9, 29.1, 28.7, 26.0, 22.7, 14.2, 11.1.

13C

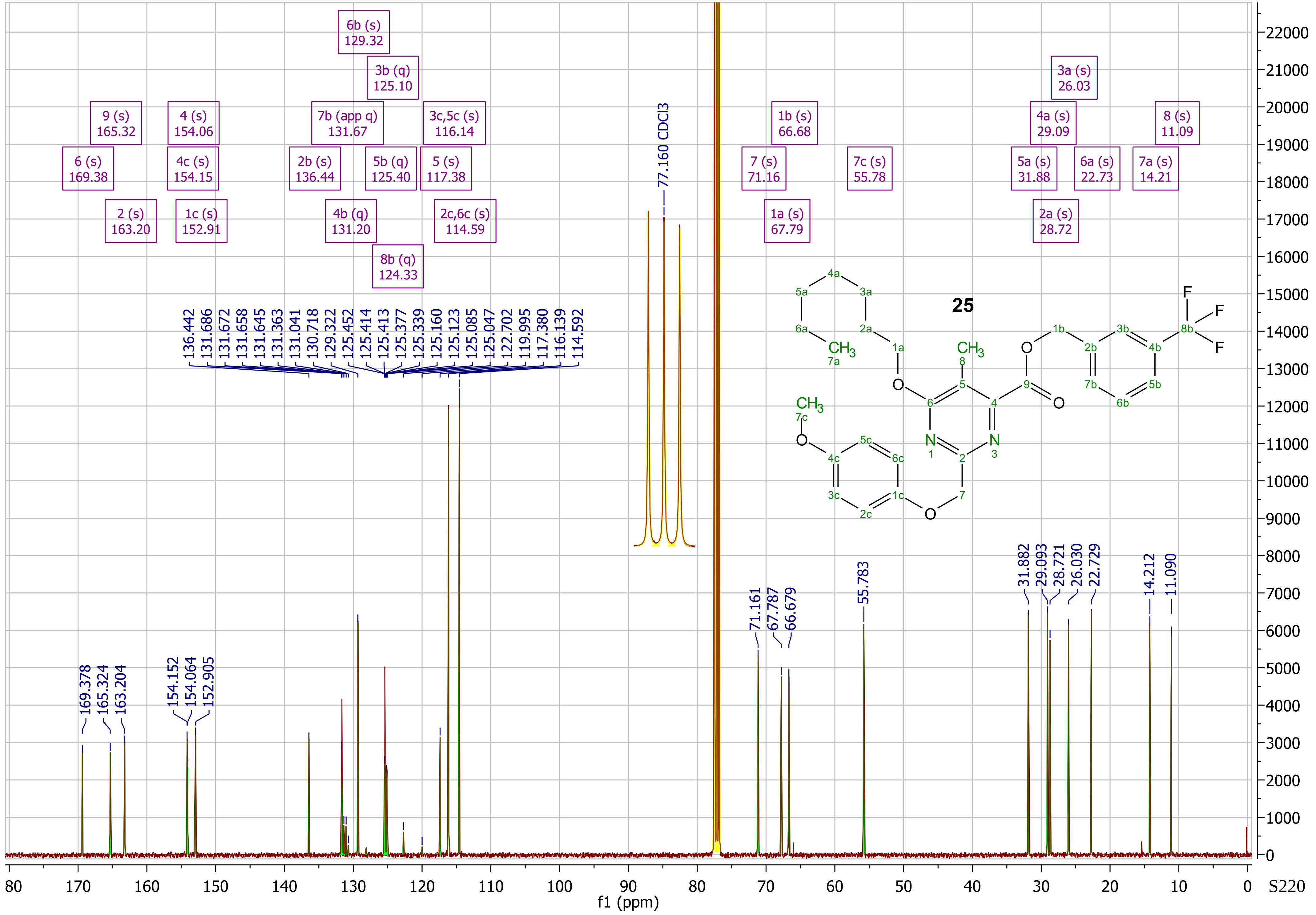

S220

13C [118.5 — 132.5 ppm]

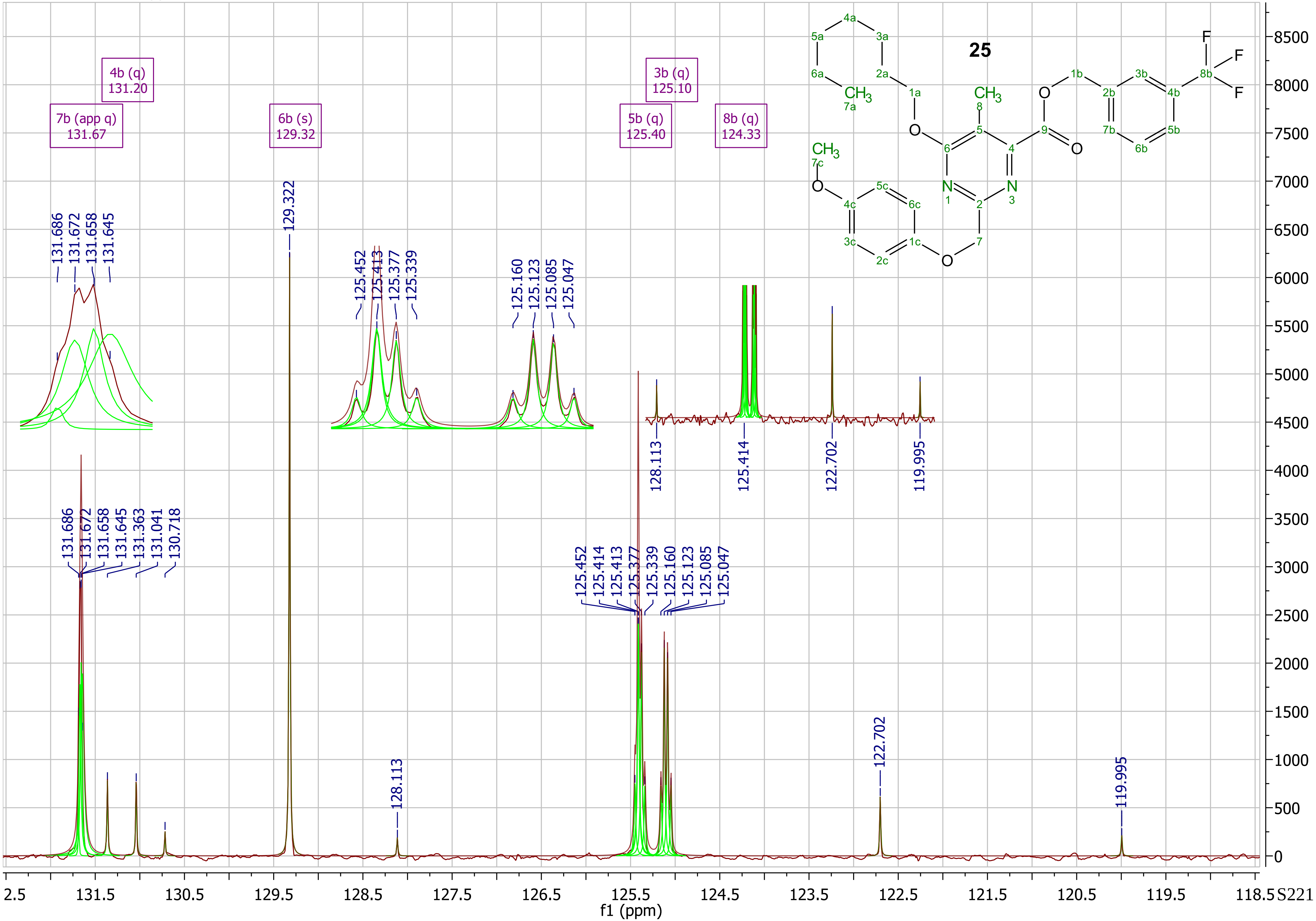

13C HSQC

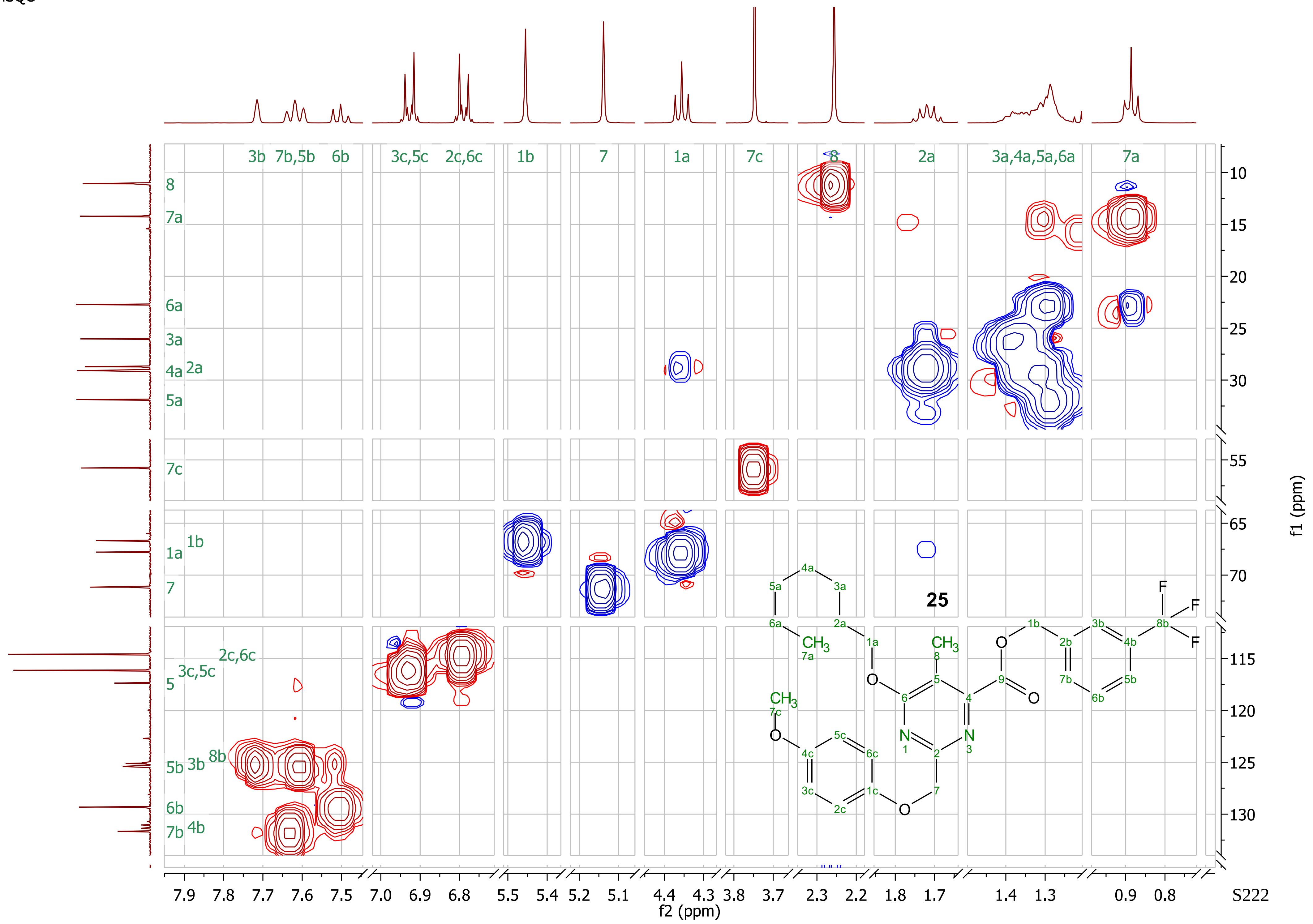

3b
7b,5b
6b
3c,5c
2c,6c
1b
7
1a
7c
8
2a
3a,4a,5a,6a
7a
8
7a
6a
3a
4a
2a
5a
7c
1b
1a
7
2c,6c
3c,5c
5
8b
5b
3b
6b
7b
4b
25
f2 (ppm)
f1 (ppm)

13C HMBC

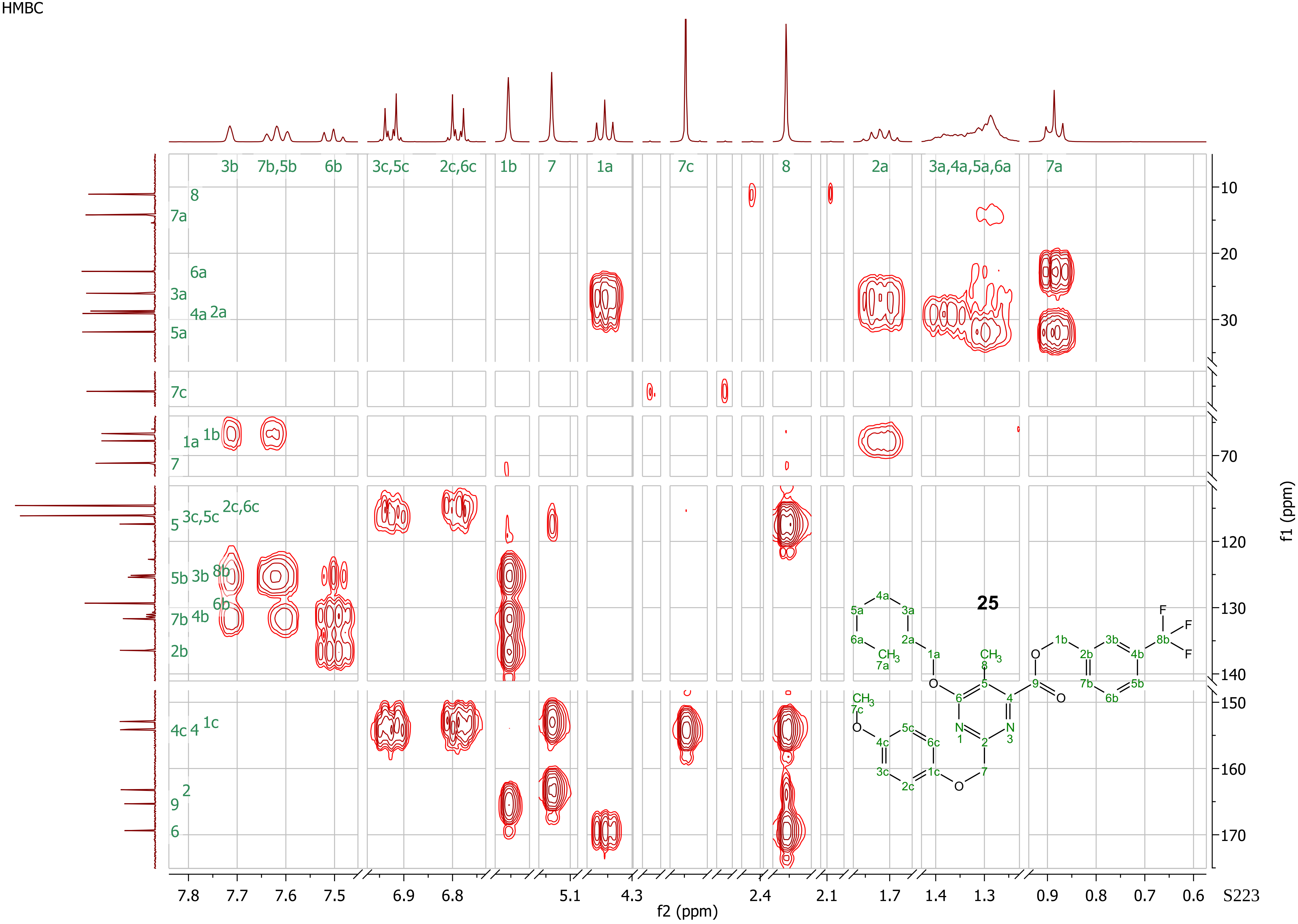

15N HMBC

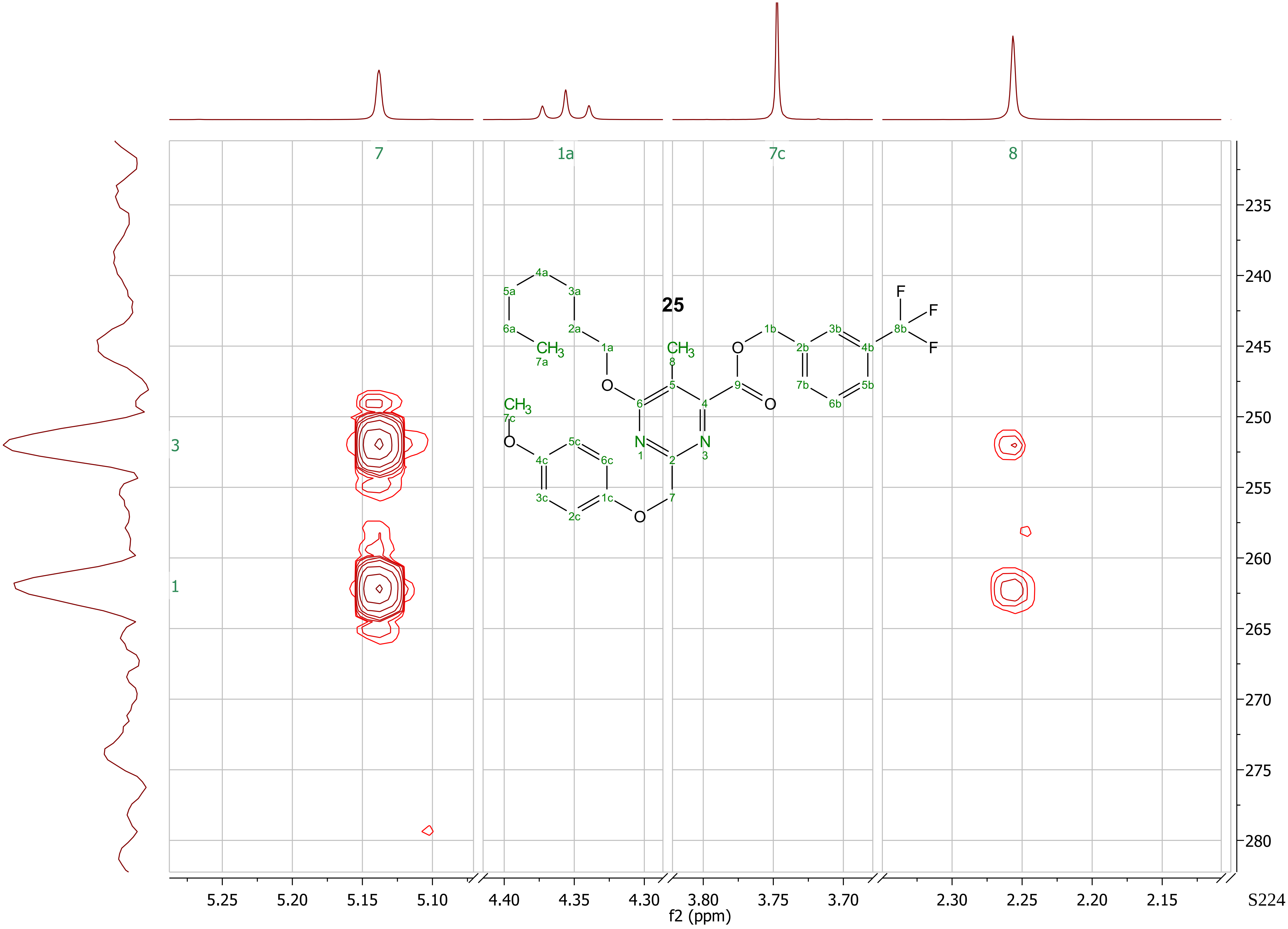

15N HMBC f1 Projection ^{15}N NMR (41 MHz, $CDCl_3$) δ 261.95, 251.97.

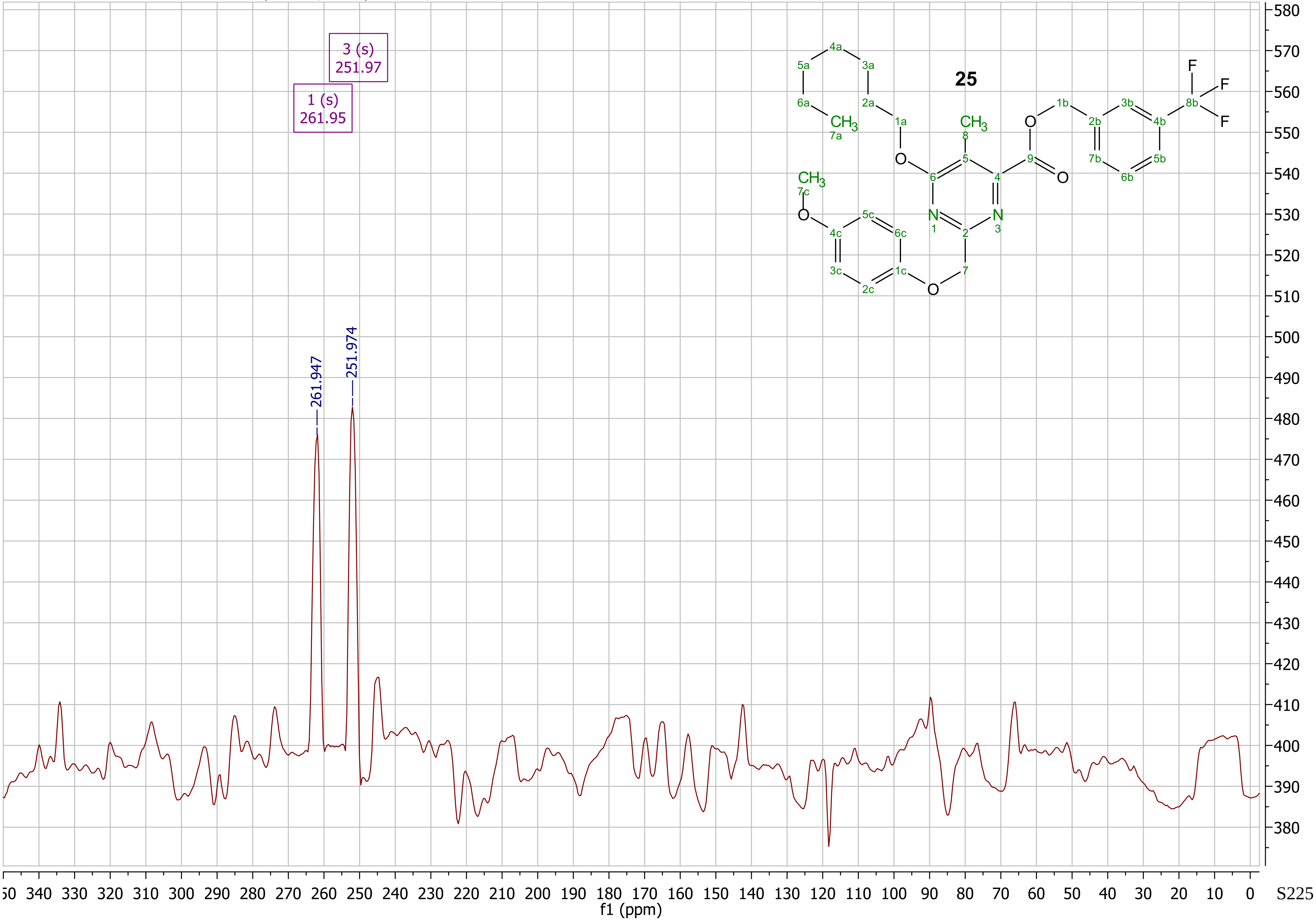

1H ^{1}H NMR (400 MHz, $CDCl_3$) δ 6.93 – 6.84 (m, 2H), 6.82 – 6.73 (m, 2H), 5.19 (quint, J = 6.0 Hz, 1H), 5.12 (s, 2H), 3.96 (s, 3H), 3.73 (s, 3H), 2.28 (s, 3H), 1.76 – 1.48 (m, 4H), 1.34 – 1.12 (m, 4H), 0.92 – 0.77 (m, 6H).

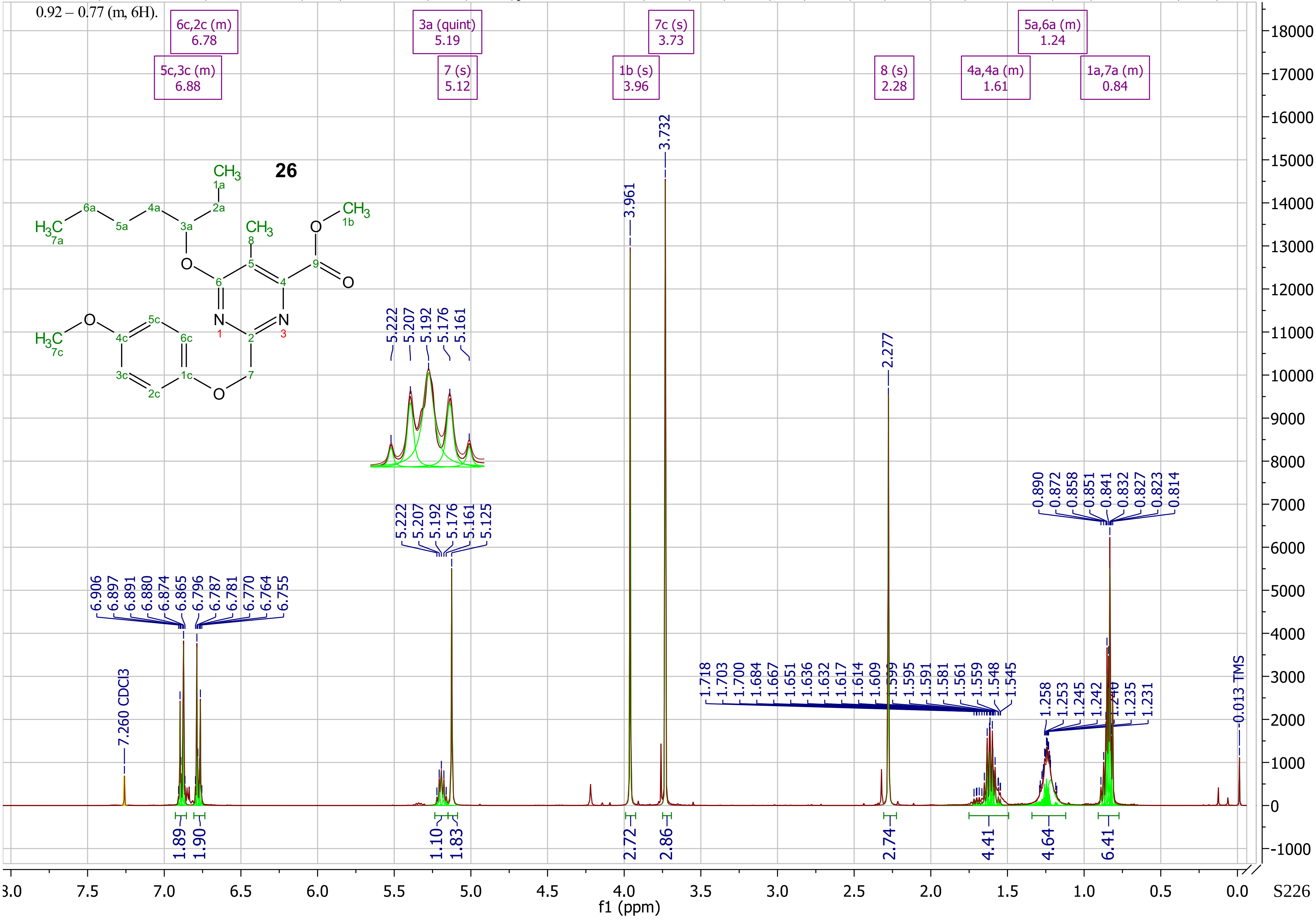

13C ^{13}C NMR (101 MHz, $CDCl_3$) δ 169.4, 166.1, 163.0, 154.3, 154.0, 152.9, 117.4, 115.9 (sym, 2C), 114.6 (sym, 2C), 78.4, 70.9, 55.8, 53.0, 32.9, 27.4, 26.6, 22.7, 14.1, 11.1, 9.5.

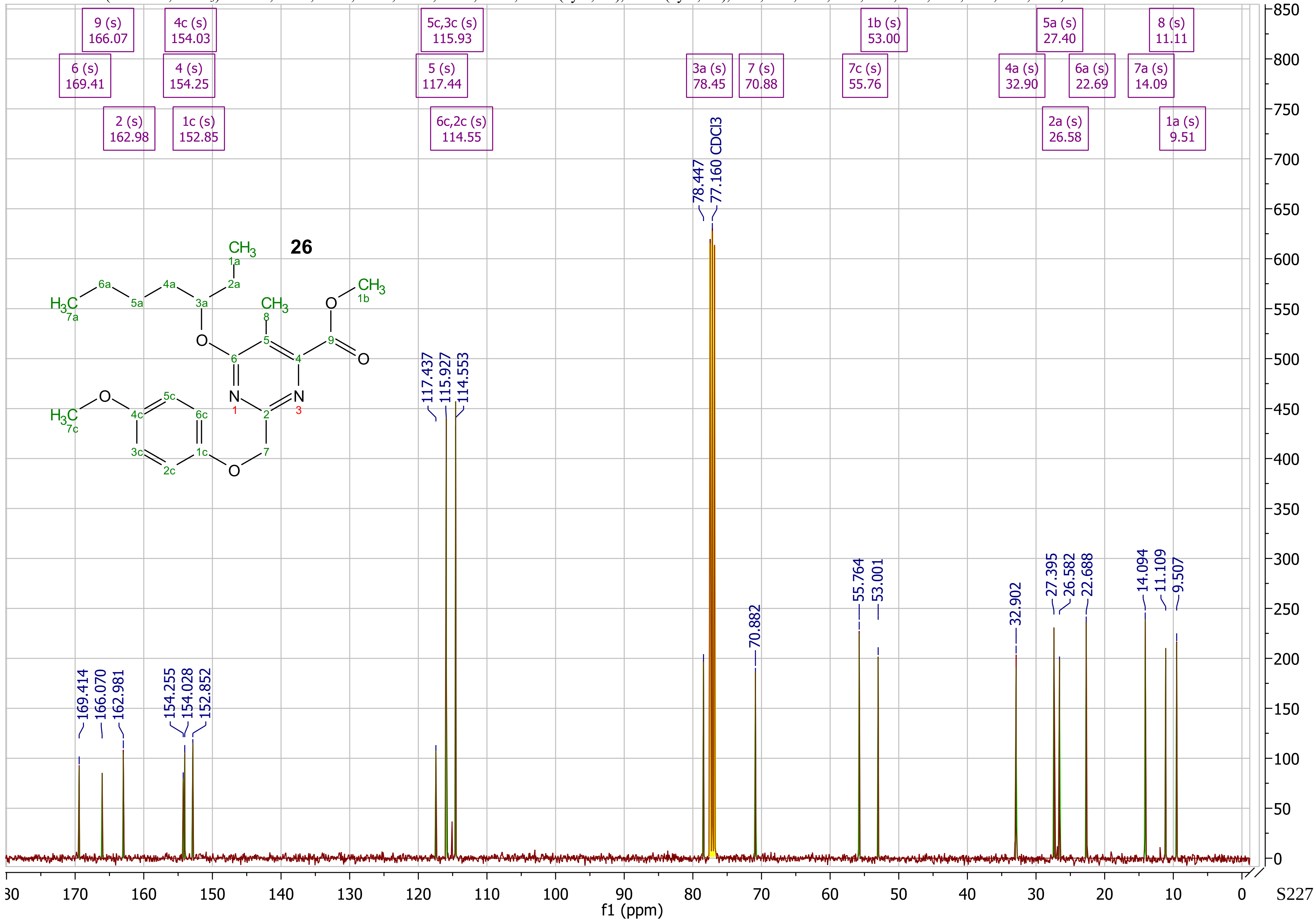

13C HSQC

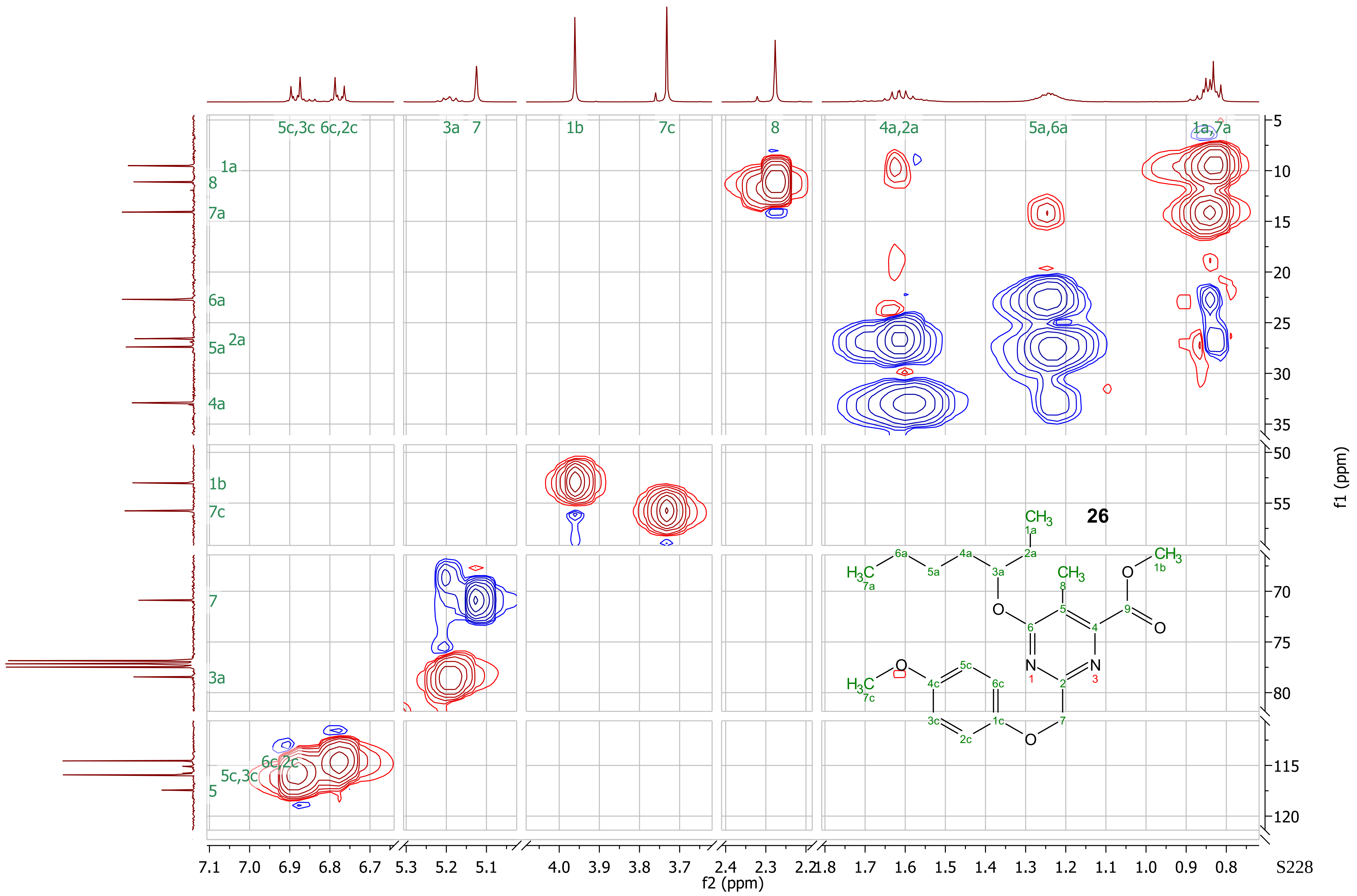

5c,3c 6c,2c
3a
7
1b
7c
8
4a,2a
5a,6a
1a,7a
1a
8
7a
6a
5a
2a
4a
1b
7c
7
3a
6c,2c
5c,3c
5
26
f2 (ppm)
f1 (ppm)

13C HMBC

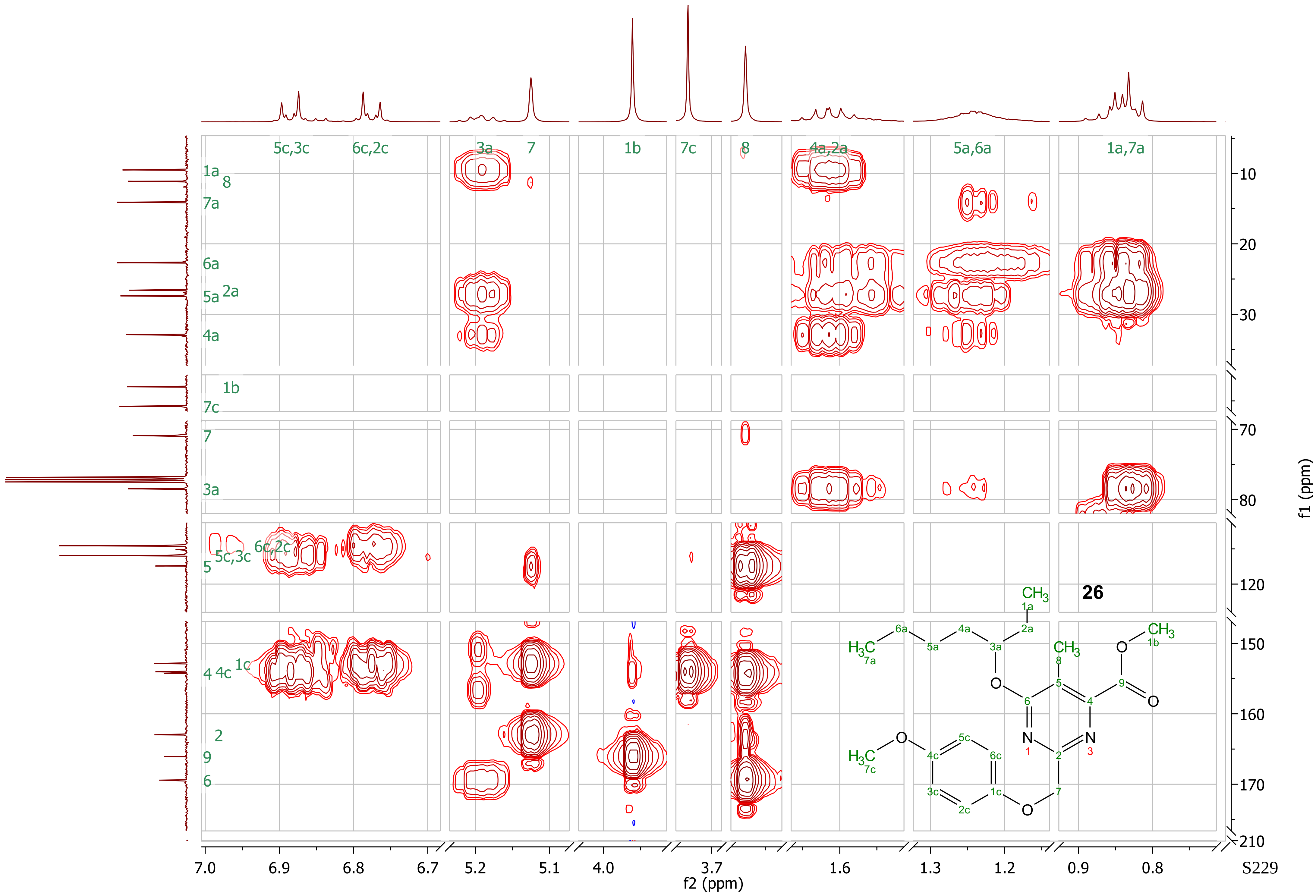

5c,3c
6c,2c
3a
7
1b
7c
8
4a,2a
5a,6a
1a,7a
1a
8
7a
6a
5a
2a
4a
1b
7c
7
3a
5c,3c
6c,2c
5
4
4c
1c
2
9
6
26
f1 (ppm)
f2 (ppm)

^{1}H NMR (400 MHz, $CDCl_3$) δ 7.72 (s, 1H), 7.64 (d, J = 7.6 Hz, 1H), 7.61 (d, J = 7.9 Hz, 1H), 7.51 (t, J = 7.7 Hz, 1H), 6.97 – 6.84 (m, 2H), 6.84 – 6.71 (m, 2H), 5.46 (s, 2H), 5.21 (quint, J = 5.9 Hz, 1H), 5.14 (s, 2H), 3.74 (s, 3H), 2.23 (s, 3H), 1.76 – 1.48 (m, 4H), 1.33 – 1.11 (m, 4H), 0.92 – 0.74 (m, 6H).

1H

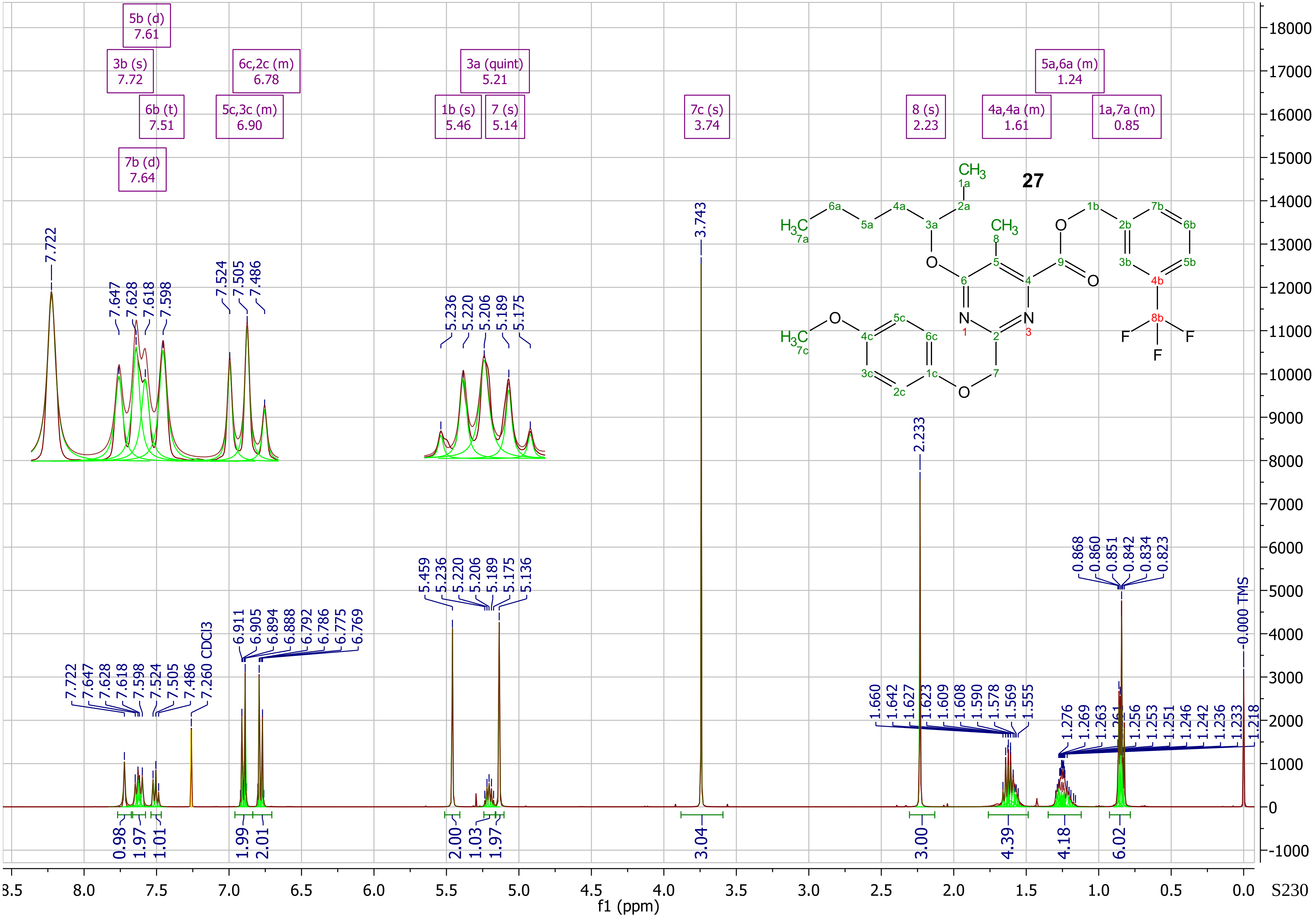

^{13}C NMR (101 MHz, $CDCl_3$) δ 169.4, 165.5, 163.2, 154.3, 154.1, 152.9, 136.4, 131.7 (app q, J = 0.9 Hz), 129.3, 125.4 (q, J = 3.8 Hz), 125.2 (q, J = 3.8 Hz), 117.2, 116.0 (sym, 2C), 114.6 (sym, 2C), 78.5, 70.9, 66.7, 55.8, 32.9, 27.4, 26.6, 22.7, 14.1, 11.1, 9.5.

13C

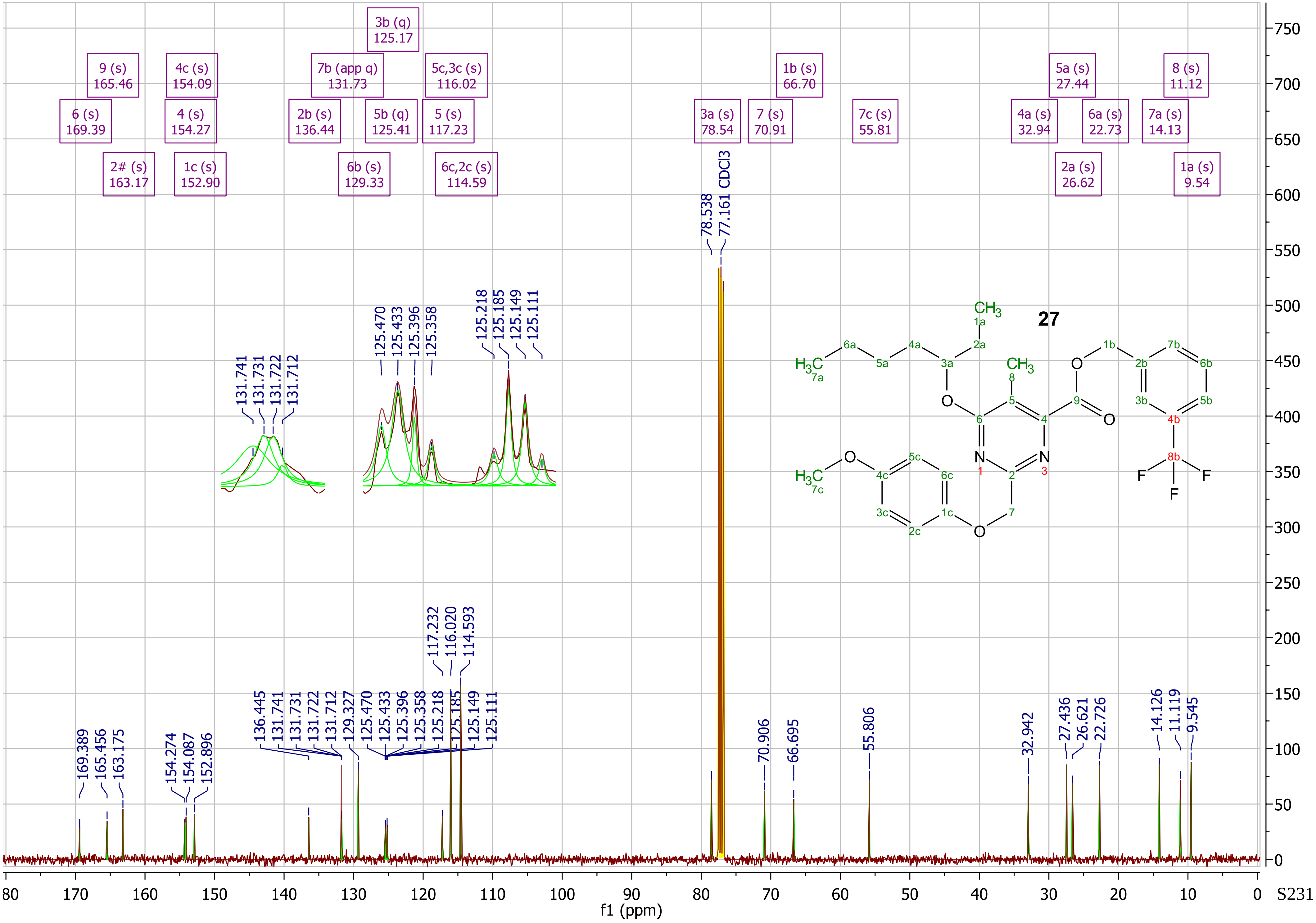

13C HSQC

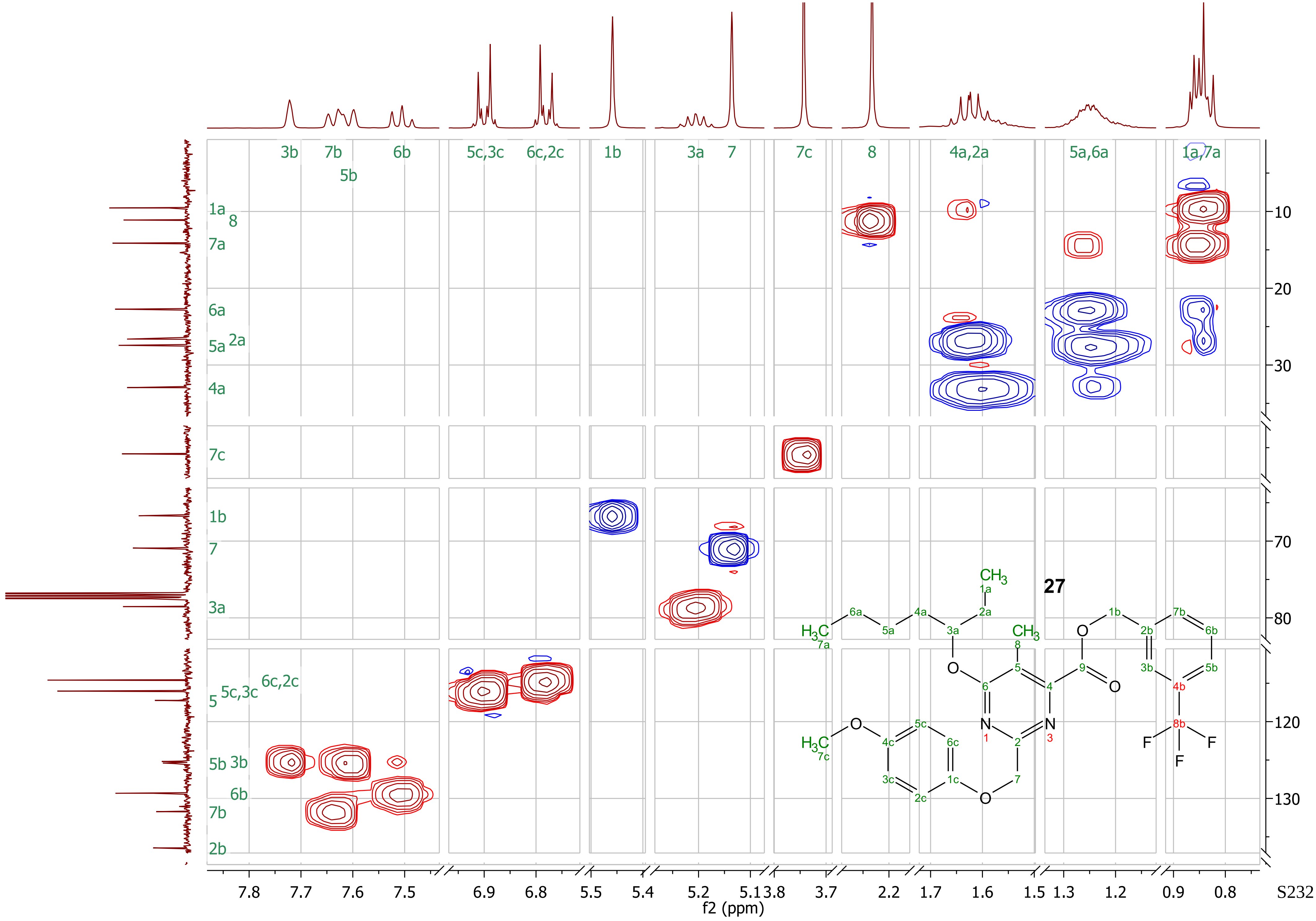

3b
7b
5b
6b
5c,3c
6c,2c
1b
3a
7
7c
8
4a,2a
5a,6a
1a,7a
1a
8
7a
6a
5a
2a
4a
7c
1b
7
3a
5
5c,3c
6c,2c
5b
3b
6b
7b
2b
27
f1 (ppm)
f2 (ppm)

13C HMBC

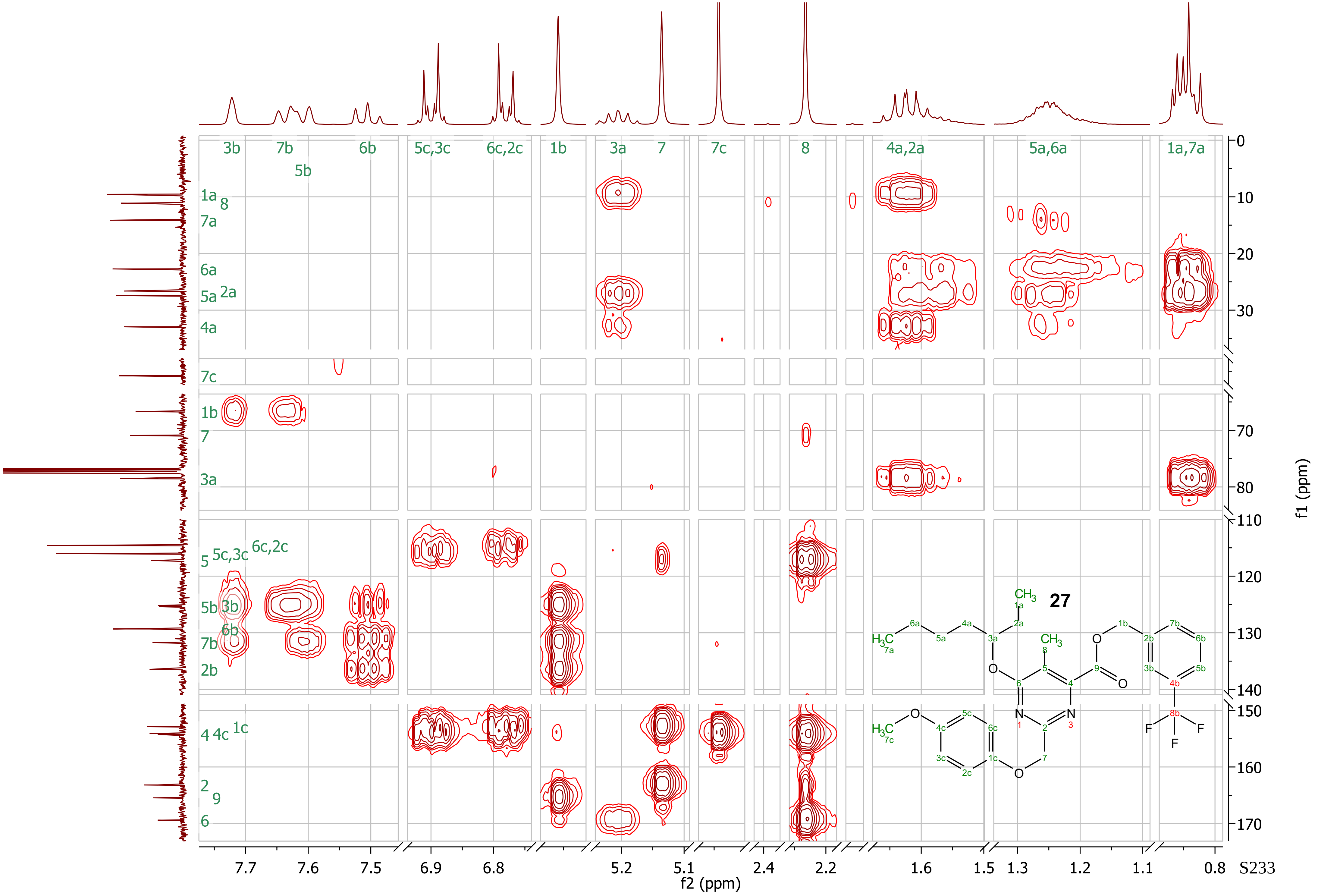

3b
7b
5b
6b
5c,3c
6c,2c
1b
3a
7
7c
8
4a,2a
5a,6a
1a,7a
1a
8
7a
6a
5a
2a
4a
7c
1b
7
3a
5
5c,3c
6c,2c
5b
3b
6b
7b
2b
4
4c
1c
2
9
6
27
f2 (ppm)
f1 (ppm)
